# Scalable Synthesis of Versatile Rare Deoxyamino Sugar Building Blocks from d-Glucosamine

**DOI:** 10.1021/acs.joc.2c03016

**Published:** 2023-05-04

**Authors:** Debashis Dhara, Marion Bouchet, Laurence A. Mulard

**Affiliations:** , Institut Pasteur, Université Paris Cité, UMR CNRS3523, Chemistry of Biomolecules Laboratory, 8 rue du Dr Roux, 75015 Paris, France

## Abstract

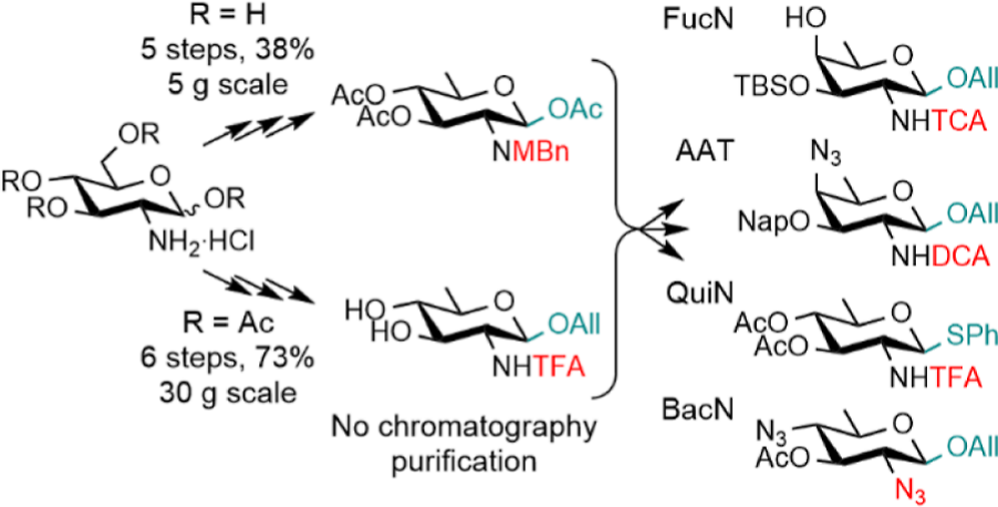

We report the syntheses of 1,3,4-tri-*O*-acetyl-2-amino-2,6-dideoxy-β-d-glucopyranose and allyl 2-amino-2,6-dideoxy-β-d-glucopyranoside from d-glucosamine hydrochloride. The potential of these two versatile scaffolds as key intermediates to a diversity of orthogonally protected rare deoxyamino hexopyranosides is exemplified in the context of fucosamine, quinovosamine, and bacillosamine. The critical C-6 deoxygenation step to 2,6-dideoxy aminosugars is performed at an early stage on a precursor featuring an imine moiety or a trifluoroacetamide moiety in place of the 2-amino group, respectively. Robustness and scalability are demonstrated for a combination of protecting groups and incremental chemical modifications that sheds light on the promise of the yet unreported allyl 2,6-dideoxy-2-*N*-trifluoroacetyl-β-d-glucopyranoside when addressing the feasibility of synthetic zwitterionic oligosaccharides. In particular, allyl 3-*O*-acetyl-4-azido-2,4,6-trideoxy-2-trifluoroacetamido-β-d-galactopyranoside, an advanced 2-acetamido-4-amino-2,4,6-trideoxy-d-galactopyranose building block, was achieved on the 30 g scale from 1,3,4,6-tetra-*O*-acetyl-β-d-glucosamine hydrochloride in 50% yield and nine steps, albeit only two chromatography purifications.

## Introduction

Carbohydrates are ubiquitous cell surface components. They play major roles in a myriad of complex biological processes governed by cell–cell or cell–environment interactions including host–pathogen recognition. Going far beyond the structural diversity seen within the human glycome, the prokaryote glycome is highly varied.^1,2^ Diversity stems for a large part from the large number of unique monosaccharides composing its alphabet. It expands as novel monosaccharides are revealed paralleling the structural analysis of natural glycans of increasing complexity.^3^ Among distinct monosaccharides not found in mammalian glycans are several rare deoxyamino sugars often present in glycans from pathogenic bacteria but essentially absent from the human microbiota.^4,5^ Whether as components of zwitterionic polysaccharides (ZPSs),^6,7^ substrates for selective metabolic labeling,^4^ key biosynthetic intermediates, and potential targets for novel antibiotics^8^ or in the context of epitope mapping and vaccine design,^7,9^ 2-amino-2,6-dideoxy-hexoses (ADDH) and 2,4-diamino-2,4,6-trideoxy-hexoses (DATDH) are the subject of wide interest.^10^

Mostly identified in ZPSs, AAT (d-Fuc*p*NAc4N) is among the most studied DATDH. Being α-linked in glycans from the well-established immunomodulators *Streptococcus pneumoniae* 1 (Sp1)^11^ and *Bacteroides fragilis* (PS A1),^12^ in the enterobacterial common antigen^13^ and in several other ZPSs, it is β-linked in the surface polysaccharides from *Shigella sonnei*,^14^*Plesiomonas shigelloides,*^15^ and other bacteria.^16−19^ Otherwise, it is occasionally found in both forms within the same glycans such as in the *S. pneumoniae* lipoteichoic acid (Sp LTA).^20^ AAT also occurs in various 4-acylamino forms.^21^ It may even be present as both 4-amino and 4-acetylamino residues within selected strains.^22^ Obviously, the different *N*-substitutions at C-2 and C-4 increase the synthetic complexity.

**Figure 1:**
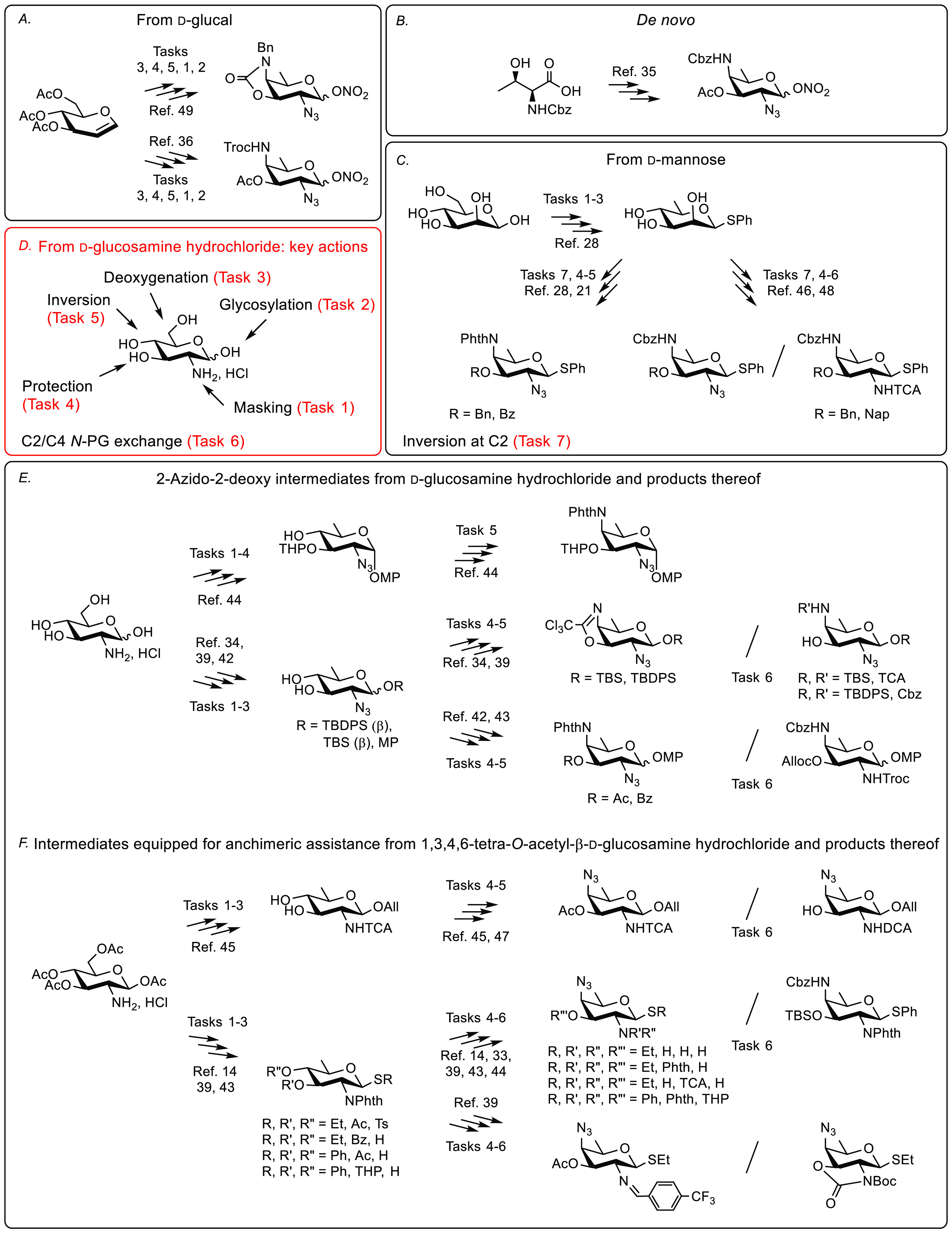
Main representative strategies to 2-*N*,4-*N*-protected AAT building blocks (panels A–C,E,F) and key actions when starting from d-glucosamine hydrochloride (panel D). All: allyl, Alloc: allyloxycarbonyl, Boc: *tert*-butoxycarbonyl, Cbz: benzyloxycarbonyl, DCA: dichloroacetyl, MP: 4-methoxyphenyl, PG: protecting group; Phth: phthalimidoyl, TBS: *tert*-butyldimethylsilyl, TBDPS: *tert*-butyldiphenylsilyl, TCA: trichloroacetyl, THP: tetrahydropyranyl, Troc: 2,2,2-trichloroethoxycarbonyl, Ts: tosyl.

Going beyond the original paths to 2-acetamido^14,23−27^ and 2,4-diacetamido glycosides,^27−29^ or post-glycosylation functionalization routes,^30−32^ a large variety of advanced AAT intermediates featuring 2,4-orthogonal protecting groups were designed (Figure 1).^10^ Most focus was on precursors to α-linked AAT for use in the synthesis of PS A1,^33−35^ Sp1,^11,24,30,36−40^ and Sp LTA.^41,42^ Except for a precursor bearing a 2-imino group^39^ and stereotunable 2-*N*,3-*O*-oxazolidinone donors,^32,39^ intermediates set up for use as glycosylating agents basically feature a 2-azido moiety.^43^ Otherwise, interest in β-linked AAT led to donors equipped for anchimeric assistance at position 2 including *N*-phthaloyl,^14^*N*-2,2,2-trichloroethoxycarbonyl,^42^ and *N*-trichloroacetyl^14,44−48^ derivatives. Besides routes based on an azidonitration step^35^ (Figure 1A) and original concepts such as a de novo path from l-threonine^34^ (Figure 1B) or a 4-amino group introduction by means of an intramolecular displacement^38,49^ (Figure 1A,E), most syntheses start from d-glucosamine^14,25,33,38,42,43,45^ (Figure 1E,F) or involve an elegant one-pot sequential inversion of 2,4-bistriflate d-thiorhamnoside intermediates (Figure 1C).^11,21,27,43,50^ They comprise 10–15 steps in average with the orthogonal masking/unmasking of the amino/acetamido groups being common concerns owing to the harsh conditions employed for the C-6 deoxygenation step. Briefly, five interdependent key actions can be identified to build 2-amino-2,6-dideoxy-hexoses and 2,4-diamino-2,4,6-trideoxy-hexoses from d-glucosamine hydrochloride: 2-NH_2_ masking, aglycon insertion, C-6 deoxygenation, 3-OH protection, and C-4 inversion, possibly complemented by protecting group exchange at positions 2 and/or 4 (Figure 1D). These five actions can be organized in multiple ways, several of which were already explored (Figure 1E,F).

The growing interest in large homogeneous segments of ZPSs^6,11,47^ has underlined the importance of robust and scalable versatile strategies to orthogonally protected ADDH. Herein, going beyond the existing paths, we propose two handy 2-amino-2,6-dideoxy scaffolds easily achievable in high yield on a large scale from the naturally abundant d-glucosamine hydrochloride. We exemplify versatility through their conversion into diverse protected ADDH and DATDH. Moreover, we demonstrate robustness for the 30 g scale synthesis of a key orthogonally protected AAT brick used in the assembly of *S. sonnei* glycans of interest for vaccine development.^47^

## Results and Discussion

**Scheme 1:**
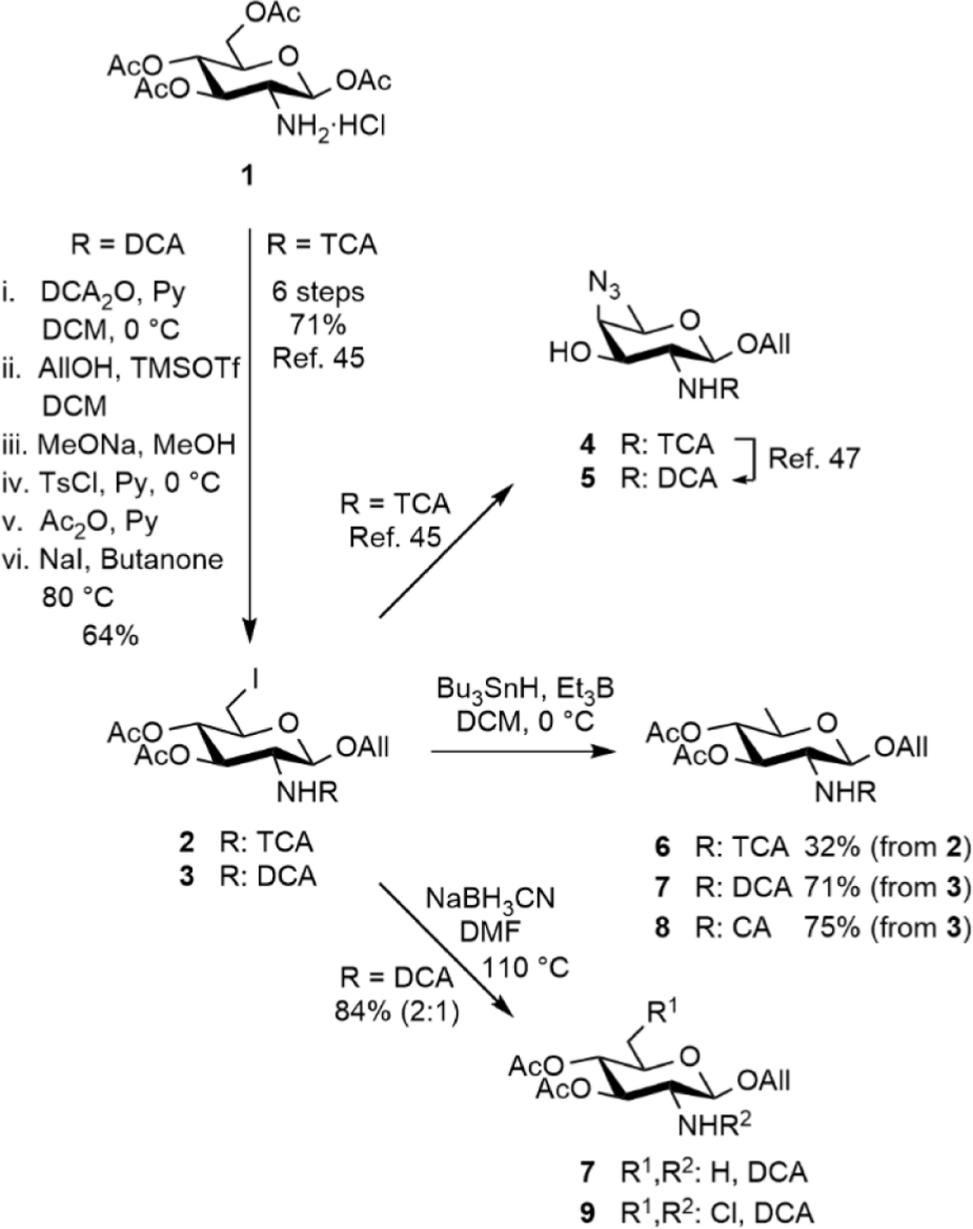
Initial Route to the 2-*N*-Trichloroacetyl and 2-*N*-Dichloroacetyl AAT (**4** and **5**) and Quinovosamine (QuiN, **6** and **7**) Building Blocks CA: chloroacetyl, Py: pyridine.

Starting from d-glucosamine hydrochloride, the limiting C-6 deoxygenation step is commonly performed on a 2-azido or a 2-phthalimido intermediate (Figure 1E,F).^38^ Instead, we have reported the 2-trichloroacetamide **2**, easily obtained from 1,3,4,6-tetra-*O*-acetyl-β-d-glucosamine hydrochloride **1** (Scheme S1), as a precursor to the AAT building block **4** (Scheme 1).^45^

In this work, the C-6 reduction step of the 6-iodo precursor **2** into trichloroacetamide **6** met with issues. Variations around the initial conditions did not fulfill the efficiency criteria either (Scheme S2). In particular, the use of NaBH_3_CN/CuX (X = Cl, Br, or I) was found less effective.^51^ Radical-mediated reduction by means of (TMS)_3_SiH/AIBN,^52^ Bu_3_SnH/Et_3_B,^53^ or (TMS)_3_SiH/Et_3_B did not overcome limitations.^54^ Concomitant hydrodechlorination yielding dichloroacetamide **7** could not be avoided, and chloroacetamide **8** was also observed under certain conditions. Similar observations stemmed from the Seeberger group^55^ and the Codée group,^56^ which reported that the thiol- and phosphine-mediated azide reduction of unrelated 2-trichloroacetamido derivatives had generated the corresponding 2-dichloroacetamido side products, respectively. We have previously achieved dichloroacetamide **5** from trichloroacetamide **4** (Scheme 1).^47^ Herein, the more straightforward synthesis of the AAT acceptor **5** from precursor **3** also met with limitations. In particular, chloroacetamide **8** and the 6-chloro derivative **9** were observed repeatedly upon radical^51,53^ or hydride-mediated reduction,^45^ respectively (Schemes 1 and S3). Having reconsidered the interdependence between the five key actions identified to build ADDH and DATDH from d-glucosamine (Figure 1D), we now propose two original alternatives to the existing paths. The critical C-6 deoxygenation step is performed on a precursor featuring either an imine moiety at position 2 in place of the starting 2-amino group (generic route 1) or a 2-trifluoroacetamide moiety to mask that same amino group (generic route 2).

### Generic Route 1

**Scheme 2:**
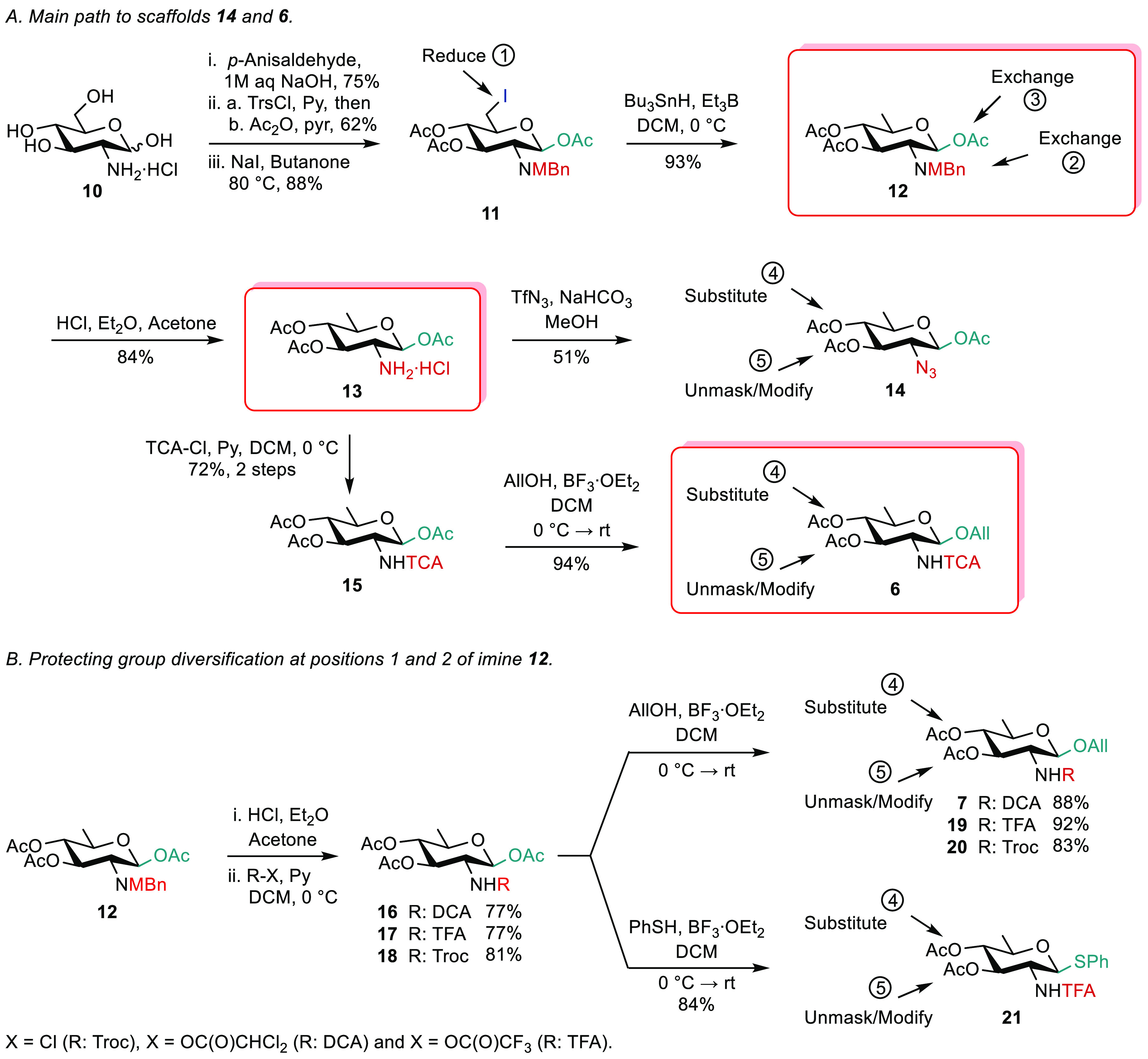
From d-Glucosamine Hydrochloride to Orthogonally Protected ADDH and DATDH Precursors via the QuiN Imine **12** MBn: *para*-methoxybenzylidene, Tf: trifluoromethanesulfonyl, TFA: trifluoroacetyl, Trs: triisopropylbenzenesulfonyl.

Aiming to achieve **4** while avoiding interference of the TCA moiety during C-6 deoxygenation, we set to investigate a route featuring a late-stage suitable *N*-protection step. We reasoned that a route encompassing an early deoxygenation step would offer a faster and more versatile access to intermediates bearing any protecting group combination. Satisfactorily, treatment of the upstream 6-iodo imine **11** with Bu_3_SnH/Et_3_B provided the 6-deoxy analogue **12** in high yield (Scheme 2). Varying the tosylation conditions had little influence (Scheme S4). In contrast, changing the tosyl group for the bulkier triisopropylbenzenesulfonyl moiety was beneficial. Acidic hydrolysis of the C-2 imine gave the versatile 1,3,4-tri-*O*-acetyl-β-d-quinovosamine (QuiN) **13** from d-glucosamine hydrochloride in five steps and 32% yield on a 5 g scale. Conversion of triacetate **13** into the 2-azido analogue **14** paved the way to a panel of AAT and QuiN donors ready for 1,2-*cis* glycosylation. Otherwise, starting from imine **12**, *N*-unmasking and subsequent trichloroacetylation of intermediate **13** furnished triacetate **15**, in turn converted into allyl glycoside **6**, a well-established AAT precursor.^45^ This efficient three-step protocol provides an access to the known **6** in eight steps from d-glucosamine hydrochloride (Scheme 2A). The process was adapted to give the *N*-dichloroacetyl (**7**), *N*-trifluoroacetyl (**19**), and *N*-Troc (**20**) analogues with excellent anomeric control (Scheme 2B). Likewise, the thiophenyl QuiN **21** was easily achieved from imine **12** by means of trifluoroacetamide **17**, demonstrating that aglycon diversification post-*N*-protection is straightforward. Subjected to improving the path to iodide **11**, imine **12** represents a highly versatile precursor to ADDH and DATDH.

**Scheme 3:**
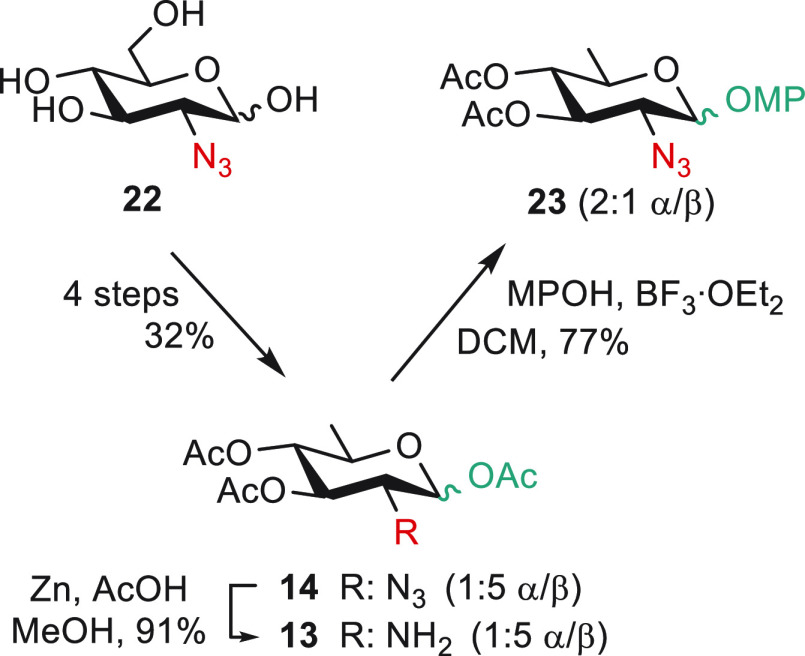
2-Azido QuiN **14** as a Scaffold

With the azido moiety fulfilling all orthogonality criteria, the more stable 2-azido-2,6-dideoxy derivative **14**, easily obtained as a 1:5 α/β mixture from the commercially available 2-azido-2-deoxy-d-glucose^57^**22** (Scheme S5), was envisioned as a possible alternative to imine **12**. Similarly to the original route whereby glycosylation preceded deoxygenation,^42^ conventional glycosylation of triacetate **14** with *para*-methoxyphenol gave the known AAT precursor **23** as a 2:1 α/β mixture (Scheme 3). Otherwise, reduction of the azido moiety in **14** was high yielding, providing an expedite access to scaffold **13**. Of note, this route delivered triacetate **13** as a 1:5 α/β mixture.

### Generic Route 2

**Scheme 4:**
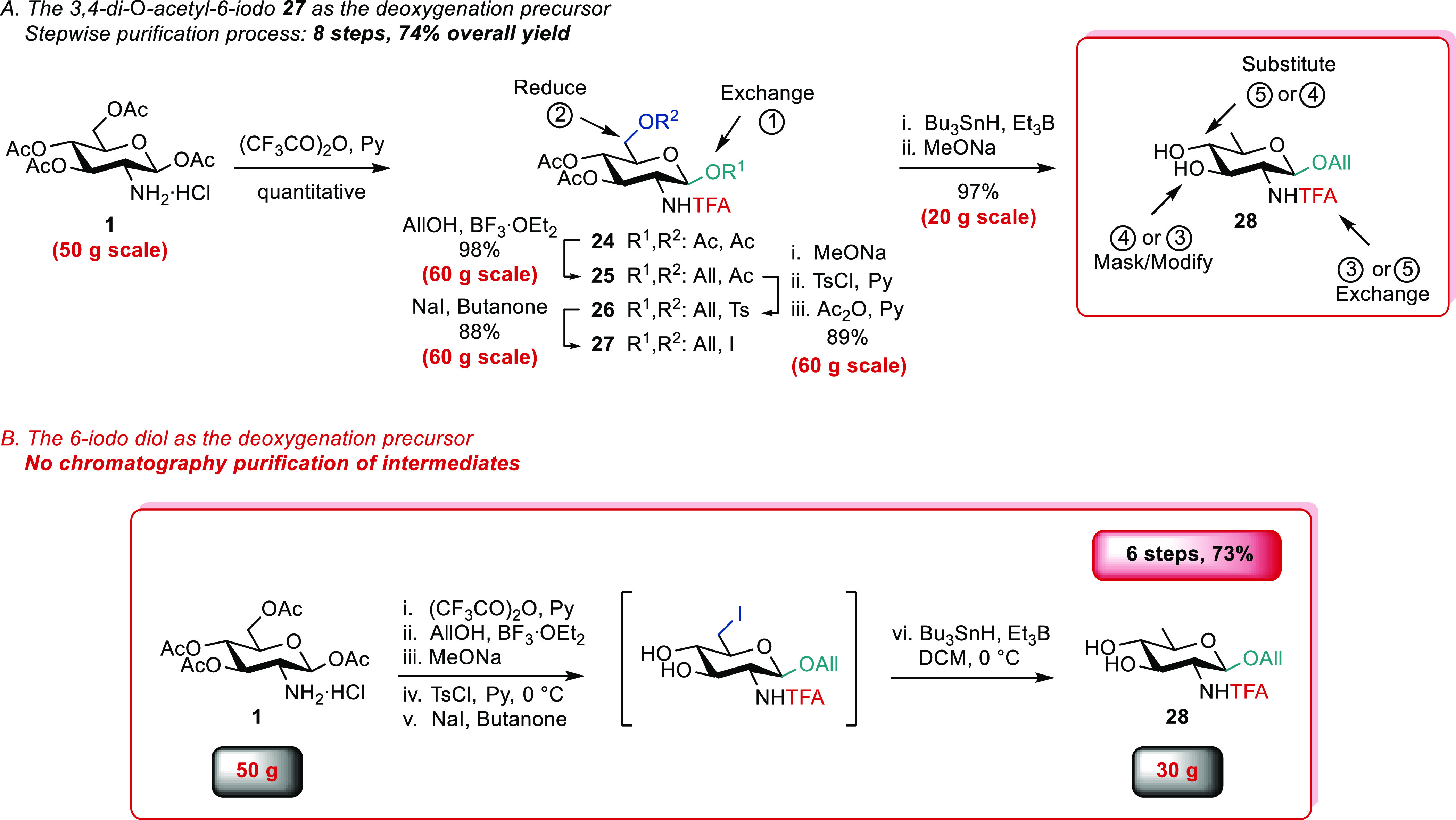
From 1,3,4,6-Tetra-*O*-acetyl-β-d-glucosamine Hydrochloride **1** to Trifluoroacetamide QuiN **28**

Aiming at improving robustness, we identified the more advanced allyl 2,6-dideoxy-2-trifluoroacetamido-β-d-glucopyranoside **28** as a promising alternative to imine **12** and azide **14** (Scheme 4). The trifluoroacetyl moiety was envisioned as an easy-to-introduce participating *N*-protecting group stable under harsh reductive conditions, albeit seemingly cleavable under relatively mild basic conditions.^58^ The synthesis of diol **28** from **1** by means of **24**(58) followed that reported for the trichloroacetamide analogue (Scheme 1).^45^ The conversion of tetra-acetate **1** into the 6-iodo **27** went smoothly to deliver the latter intermediate in 77% yield over six steps (Scheme 4A). It is noteworthy that the key reduction step furnished diacetate **19** in excellent yield (96%, 40 g scale) when using an optimized Bu_3_SnH/Et_3_B combination (Scheme S6). Robustness was confirmed as reduction at C-6 of **27** and subsequent transesterification gave diol **28** in a 90–97% yield on a 20 g scale (Scheme S7). Moreover, investigation toward scale-up demonstrated that re-*O*-acetylation post-tosylation was not mandatory. This step and the related transesterification of diacetate **19** into diol **28** were removed from the process. As a reward, scaffold **28**, serving as a versatile intermediate to protected ADDH and DATDH, was obtained in six steps and 78% overall yield from the commercially available **1** on a 150 mmol scale (Schemes 4B and S8). Modification at positions 2, 3, and 4 of diol **28** can follow multiple paths (Scheme 4A), in part exemplified below.

**Scheme 5:**
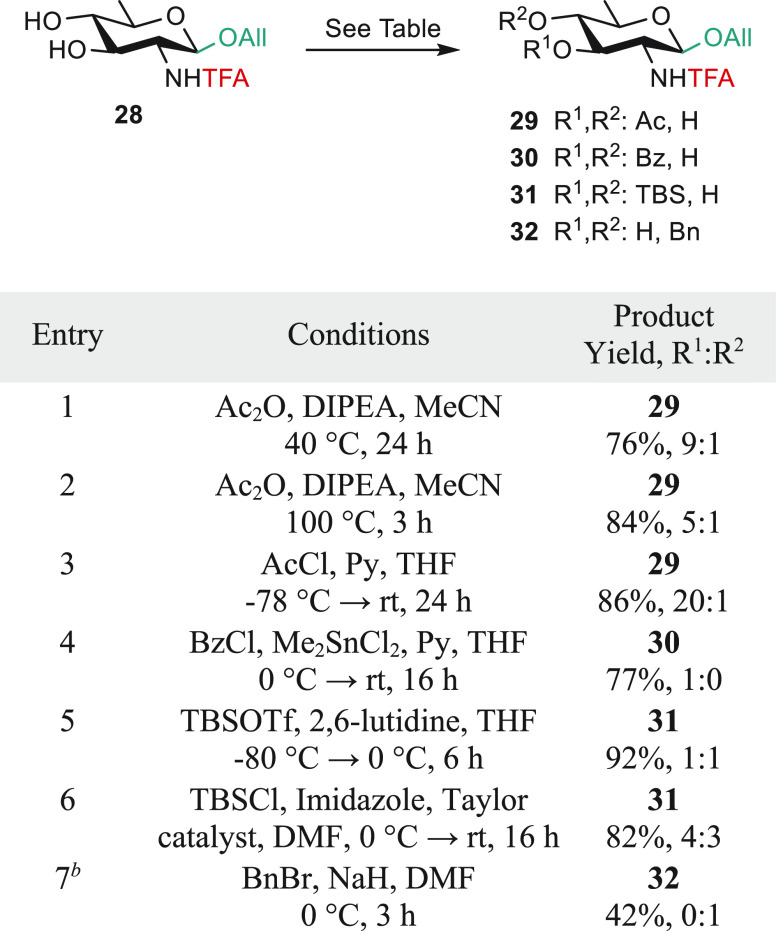
Regioselective *O*-Protection of the QuiN Diol **28** Isolated yields. ^b^Corrected yield: 79%. DIPEA: *N*,*N*-diisopropylethylamine.

### From the *N*-Trifluoroacetyl QuiN **28** to AAT and d-FucNAc Building Blocks: 3-*O*-Protection and C-4 Inversion

Applying the diisopropylethylamine (DIPEA)-triggered self-catalyzed regioselective acetylation conditions^59^ to diol **28** provided acetate **29** together with its regioisomer (Scheme 5, entries 1 and 2). Aiming at avoiding formation of the latter, diol **28** was treated with acetyl chloride under conditions previously established for the trichloroacetamide analogue.^45^ This more demanding protocol delivered the expected **29** with an improved regioselectivity (entry 3). This option was adopted on a multigram scale. Otherwise, the 3-*O*-benzoyl **30** was achieved smoothly (77%) in the presence of Me_2_SnCl_2_ (entry 4).^60^ In contrast, *tert*-butyldimethylsilylation required attention to furnish the expected **31**. The 4-*O*-*tert*-butyldimethylsilyl isomer was always present (entries 5 and 6). Moreover, the 4-*O*-benzyl **32** was formed preferentially while the reaction was stopped before completion to avoid extensive *N*-benzylation in the presence of sodium hydride (entry 7). To our knowledge, the observed regioselectivity was not reported previously for *N*-protected QuiN derivatives. Further attempts at achieving a better regioselectivity by use of a diaryl boronic acid catalyst^61^ or an iron catalyst^62^ were unsuccessful.

**Scheme 6:**
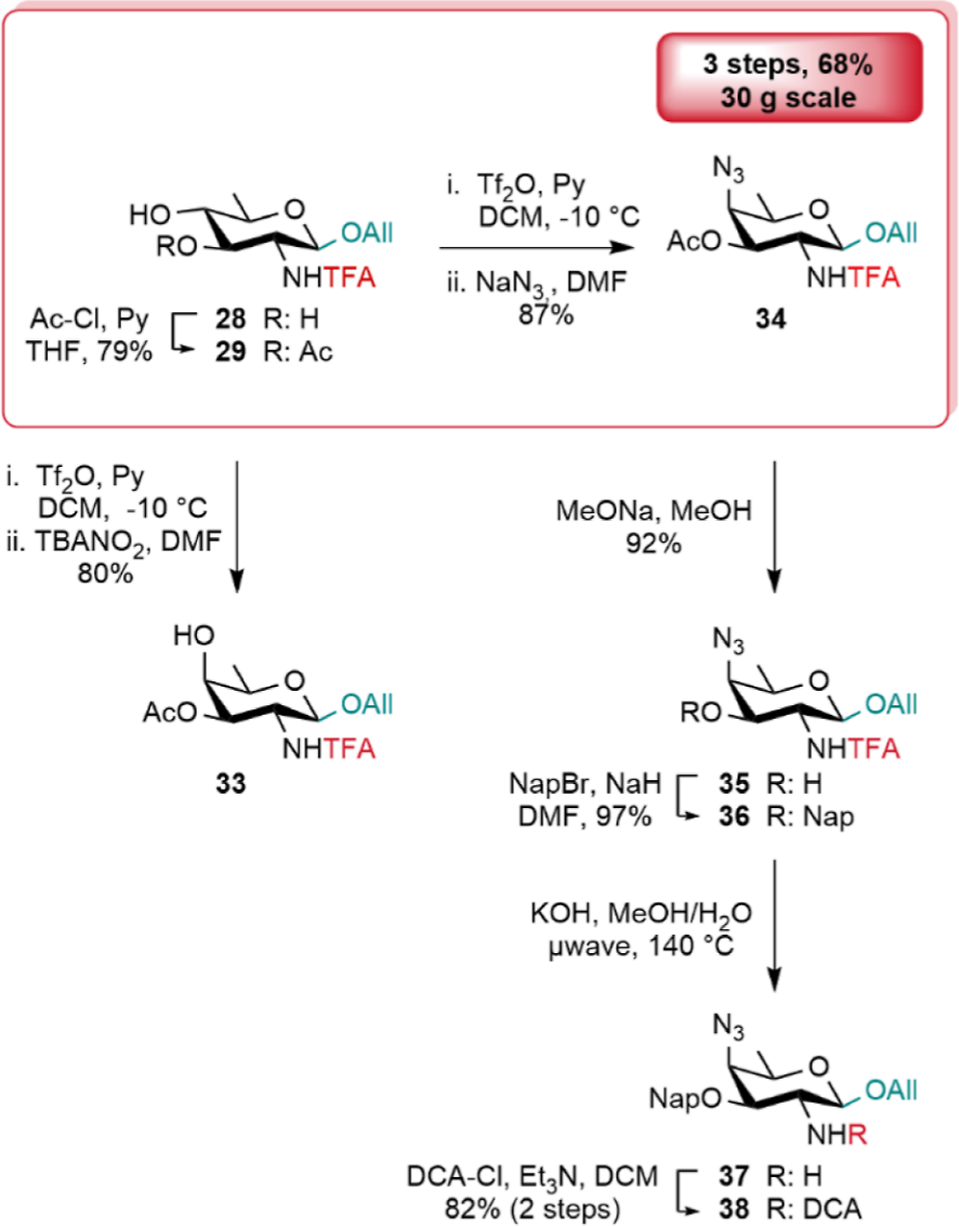
From the *N*-Trifluoroacetyl QuiN **28** to d-FucNAc (**33**) and AAT (**34–38**) Building Blocks TBANO_2_: tetrabutylammonium nitrite.

Alcohol **29** was next elaborated into d-FucNAc **33** in high yield via triflate-mediated inversion using the nitrite-mediated Lattrell-Dax method.^63^ Alternatively, azide-mediated substitution of the intermediate triflate readily generated upon reaction of **29** with triflic anhydride in the presence of pyridine delivered the fully protected AAT **34** in excellent yield (Scheme 6). Moreover, scale-up toward a robust synthesis of AAT was in agreement with expectations. In particular, the key intermediate **34** was achieved in 68% yield from diol **28** on the 30 g scale by means of the 3-*O*-acetyl QuiN **29**, readily obtained as a >15:1 mix with its 4-*O*-acetyl regioisomer (Scheme 6).

Selective unmasking of 3-OH in **34** provided acceptor **35** on the way to diversely 3-*O*-protected analogues. Going beyond the unsuccessful 3-*O*-benzylation of the QuiN diol **28**, the 3-*O*-Nap **36** was easily achieved from the 4-azido AAT precursor **35**. As exemplified with the conversion of AAT **36** into its dichloroacetamide counterpart **38** by way of amine **37** (Scheme 6), AAT derivatives bearing a 3-*O*-ether protecting group are achievable in high yield from their fully protected trifluoroacetamide equivalents. Nevertheless, in contrast to 3-*O*-ester-equipped analogues, the cleavage of the trifluoroacetamide moiety in these substrates requires rather harsh conditions.

### From the *N*-Trifluoroacetyl QuiN **28** and AAT **34** to Diversely 2-*N*-Protected AAT Building Blocks

**Scheme 7:**
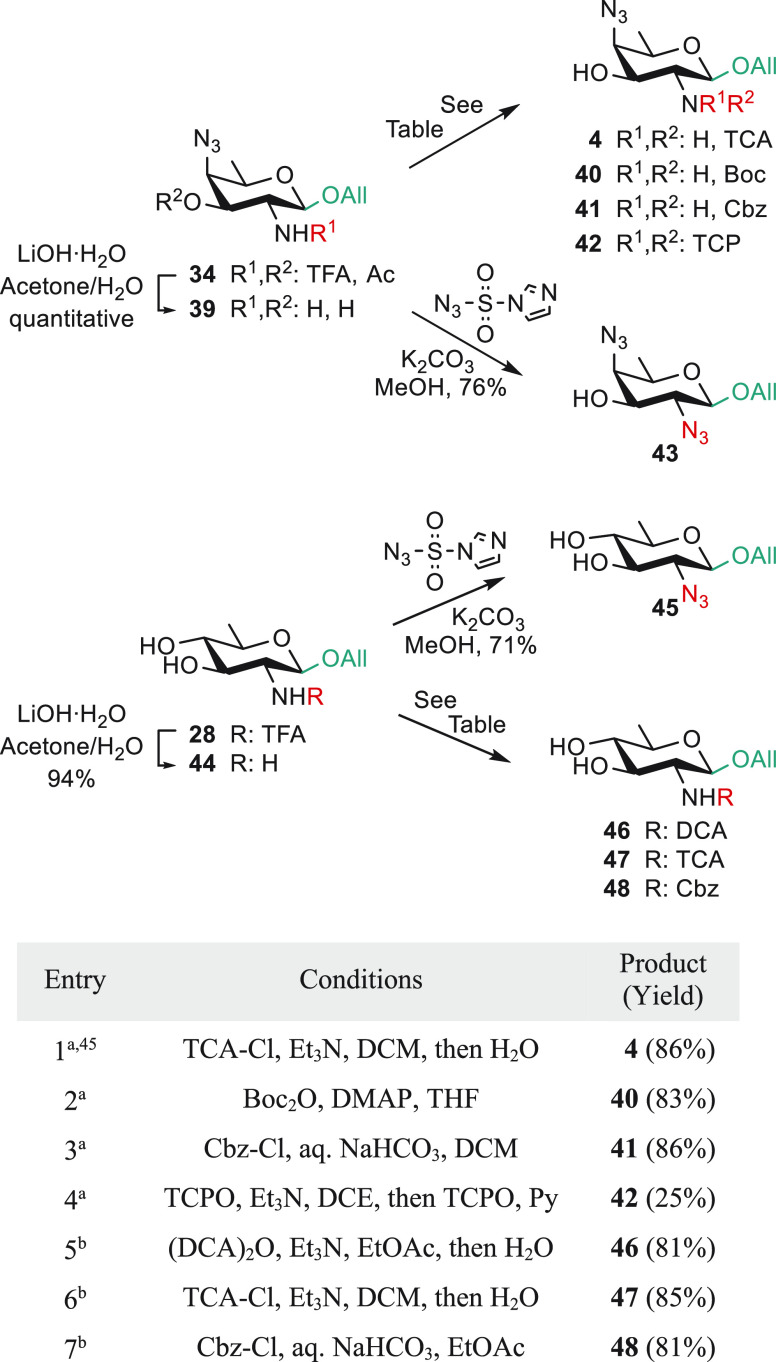
*N*-Protecting Group Manipulation from the *N*-Trifluoroacetyl AAT and QuiN Precursors, **34** and **28**, Respectively From **39**. ^b^From **44**. TCPO: tetrachlorophthalic anhydride, (DCA)_2_O: dichloroacetic anhydride.

The next step ensuring a versatile strategy consisted in establishing that trifluoroacetamide **34** was a suitable intermediate to a large variety of AAT bricks differing by the 2-acetamido masking, which may find interest in the synthesis of glycans obeying stringent orthogonality criteria. Thus, AAT **34** was subjected to a smooth *N*-trifluoroacetyl cleavage upon treatment with lithium hydroxide (Scheme 7). Subsequent selective *N*-protection of amino alcohol **39** delivered the known trichloroacetamide acceptor **4**(45) (entry 1) and carbamates **40** and **41** (entries 2 and 3), all in high yield in contrast to the tetrachlorophthalimide **42** (entry 4). Trichloroacetamide **4**, a well-established AAT acceptor in the synthesis of *S. sonnei* oligosaccharides, was achieved in 13 g amounts from 1,3,4,6-tetra-*O*-acetyl-β-d-glucosamine hydrochloride **1** in 11 steps, three chromatography purifications, and 45% overall yield. Otherwise, starting from the QuiN diol **28**, *N*-trifluoroacetyl cleavage delivered amine **44**, facilitating selective *N*-masking, as required. Accordingly, trichloroacetamide **47**(45) (entry 6), the novel dichloroacetamide **46** (entry 5), and carbamate **48** (entry 7) QuiN derivatives, C-2-equipped for anchimeric assistance, are easily accessible from diol **28**. Similarly, analogues **43** and **45** bearing a C-2 azide were achieved by an azido transfer reaction from amines **39** and **44**, respectively (Scheme 7).

**Scheme 8:**
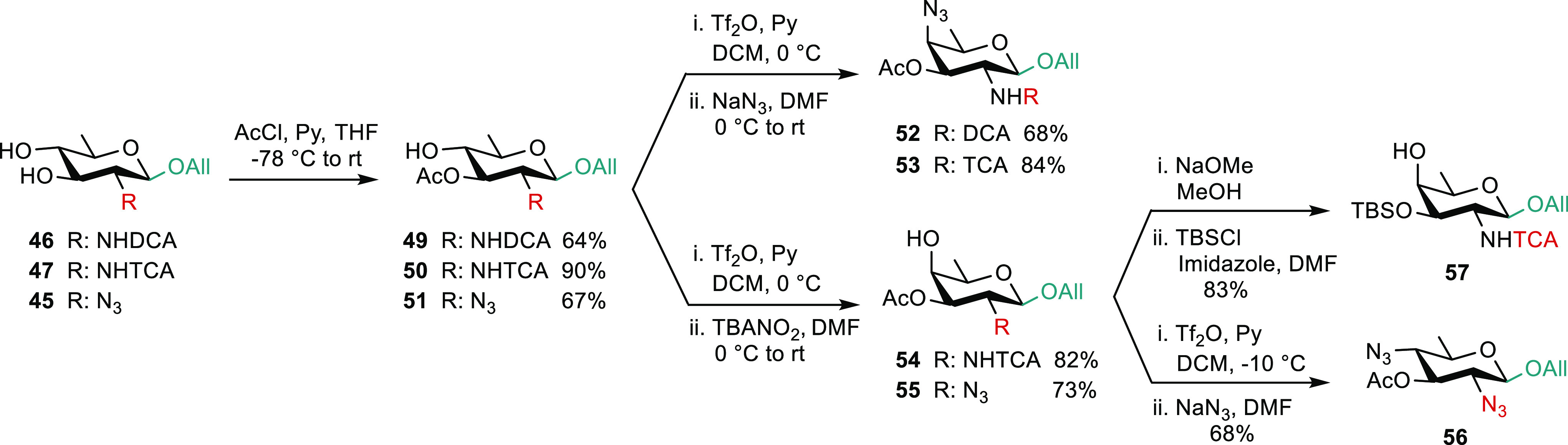
From *N*-Protected QuiN **45–47** to ADDH and DATDH Bricks

Alike the *N*-trifluoroacetyl diol **28**, the *N*-protected QuiN **45**(45)**–47** provided an additional set of orthogonally protected d-FucNAc and AAT derivatives when subjected to regioselective protection at OH-3, triflation, and subsequent nucleophilic substitution at C-4 (Scheme 8). This is exemplified for QuiN **49**, **50,**(45) and **51** and next converted in good to excellent yields into the corresponding AAT (**52** and **53**(45)) and FucNAc (**54** and **55**) bricks, respectively. Overall, the trifluoroacetyl protecting group was found superior. However, as for the trifluoroacetamide QuiN **28**, attempted 3-*O*-silylation of diol **47** met with issues. Alternatively, the 3-*O*-silylated FucNAc **57** was easily obtained from acetate **54**. Illustrating further the broad scope of building block **28**, the FucNAc intermediate **55** was converted to the 2,4-diazido bacillosamine **56**(Scheme 8).

## Conclusions

The proof of concept is established for two versatile strategies to rare d-deoxyamino sugars from the naturally abundant d-glucosamine hydrochloride and its more advanced commercially available 1,3,4,6-tetra-acetate equivalent. Key features include a temporary 2-*N*-protection in the form of an imine or a trifluoroacetamide, enabling a high yielding early stage C-6 deoxygenation step. The tosylation–iodination–reduction at the primary hydroxyl was achieved in high yield on the 3,4,6-triol substrate. Subsequent diversity-oriented chemical manipulation provided orthogonally protected QuiN, FucNAc, and AAT intermediates equipped for 1,2-*cis* and/or 1,2-*trans* glycosylation. Versatility is illustrated in terms of protecting group selection and sequential introduction to deliver multiple ADDH and DATDH bricks. Some examples are closely related to known compounds as the disclosed routes may be envisioned as alternatives to published syntheses. The novel 6-deoxy-d-glucosamine derivatives, diacetate **19** and its diol equivalent **28**, both of which feature a 2-trifluorocatamide moiety, were shown to be easily achievable on the multidecagram scale. While these allyl glycosides are the main model scaffolds in this study, the proposed strategies are easily applicable to other substrates, especially thioglycosides, thus enabling greener C-6 reduction conditions. Beyond novelty, this study adds to previous demonstrations of the potential of fine-tuned generic scaffolds for the synthesis of rare deoxyamino sugars, the interest for which is manifest. Synthesis robustness and scalability are demonstrated for the pivotal QuiN diol **28** (30 g, six steps, 73%, no chromatography purification) and the more advanced AAT **34** (30 g, nine steps, 53%, two chromatography purifications) proposed for use in the synthesis of zwitterionic glycans of relevance in adjuvant and vaccine development.

## Experimental Section

### General Procedures

Anhydrous (Anhyd.) solvents—including DCM, 1,2-dichloroethane (DCE), tetrahydrofuran (THF), *N*,*N*-dimethylformamide (DMF), MeOH, MeCN, Py, and toluene (Tol)—were delivered over molecular sieves (MS) and used as received. Reactions requiring anhyd. conditions were run under an argon (Ar) atmosphere using dried glassware. Freshly activated 4 Å MS were prepared before use by heating under high vacuum. Reactions were performed at a concentration of 100–200 mmol/L with respect to the limiting starting material unless indicated otherwise. The amount of added reagents with respect to the limiting starting material is according to specific indications in the experimental procedures. Reactions run under microwave conditions used a Biotage Initiator+ Fourth Generation Microwave Synthesizer and high-precision 20 mL microwave vials. Analytical thin-layer chromatography (TLC) was performed with silica gel 60 F254, 0.25 mm pre-coated TLC aluminum foil plates. Compounds were visualized using UV (λ = 254 nm) and/or orcinol (1 mg·mL^–1^) in 10% aq H_2_SO_4_ with charring. Flash column chromatographies were carried out using silica gel (25 or 40–63 μm particle size). Reactions that required heating were run in flasks equipped with an air flux condenser using a heat-on block equipped with an external temperature probe and filled in with sand whenever necessary to ensure proper heat transfer. NMR spectra were recorded at 303 K on a Bruker AVANCE spectrometer equipped with a BBO probe at 400 MHz (^1^H) and 100 MHz (^13^C). Spectra were recorded in CDCl_3_, CD_3_CN, CD_3_OD, and DMSO-*d*_6_. Chemical shifts are reported in ppm (δ) relative to residual solvent peaks at 7.28/77.0 ppm for CDCl_3_, 1.39/1.32 ppm for CD_3_CN, 3.33/49.0 ppm for CD_3_OD, and 2.50/39.5 ppm for DMSO-*d*_6_ for the ^1^H and ^13^C spectra, respectively. Coupling constants are reported in hertz (Hz). Elucidation of chemical structures is based on ^1^H, COSY, DEPT-135, HSQC, decoupled HSQC, ^13^C, decoupled ^13^C, and HMBC spectra. Signals are reported as s (singlet), d (doublet), t (triplet), dd (doublet of doublet), q (quartet), dt (doublet of triplet), dq (doublet of quartet), ddd (doublet of doublet of doublet), m (multiplet), and broad (prefix br). Of the two magnetically non-equivalent geminal protons at C-6, the one resonating at a lower field is denoted as H-6a, and the one at a higher field is denoted as H-6b. HRMS spectra were recorded in the positive-ion electrospray ionization (ESI^+^) mode on a WATERS QTOF Micromass instrument or on a Q exactive mass spectrometer (Thermo Fisher Scientific) equipped with a H-ESI II Probe source. Solutions were prepared using 1:1 MeCN/H_2_O containing 0.1% formic acid. In the case of sensitive compounds, solutions were prepared using 1:1 MeOH/H_2_O to which was added 10 mM ammonium acetate.

#### Allyl 3,4-Di-*O*-acetyl-2-deoxy-2-dichloroacetamido-6-iodo-β-d-glucopyranoside (**3**)

Allyl 3,4-di-*O*-acetyl-2-deoxy-2-dichloroacetamido-6-*O*-toluenesulfonyl-β-d-glucopyranoside (9.8 g, 17.2 mmol, 1.0 equiv) was dissolved in butanone (122 mL), and sodium iodide (6.8 g, 45.3 mmol, 2.5 equiv) was added. After stirring at 80 °C for 6 h, TLC monitoring (Tol/EtOAc 4:1) showed the complete consumption of the starting material (*R*_*f*_ 0.25) and the presence of a less polar material (*R*_*f*_ 0.4). Volatiles were evaporated, and the residue was diluted with dichloromethane (300 mL). The organic phase was washed with water and brine, dried over Na_2_SO_4_, filtered, and concentrated in vacuo. Flash chromatography of the residue (Tol/EtOAc 86:14 → 80:20) gave the 6-iodo derivative **3** as a white solid (7.6 g, 14.1 mmol, 82%). The desired **3** had *R*_*f*_ 0.3 (Tol/EtOAc 4:1). ^1^H NMR (400 MHz, CDCl_3_): δ 6.53 (d, 1H, *J*_NH,2_ = 8.8 Hz, NH), 5.95–5.85 (m, 1H, CH_All_), 5.89 (s, 1H, CHCl_2_), 5.39–5.32 (m, 2H, H-3, CH_2All_), 5.27–5.23 (m, 1H, CH_2All_), 4.92 (dd, 1H, *J*_4,3_ = 9.2 Hz, *J*_4,5_ = 9.6 Hz, H-4), 4.77 (d, 1H, *J*_1,2_ = 8.0 Hz, H-1), 4.44–4.39 (m, 1H, CH_2All_), 4.23–4.17 (m, 1H, CH_2All_), 3.95 (dt, 1H, *J*_2,3_ = 10.8 Hz, H-2), 3.59 (ddd, 1H, H-5), 3.33 (dd, 1H, *J*_5,6a_ = 2.7 Hz, *J*_6a,6b_ = 10.9 Hz, H-6a), 3.19 (dd, 1H, *J*_5,6b_ = 8.8 Hz, H-6b), 2.09, 2.04 (2s, 6H, CH_3Ac_). ^13^C{^1^H} NMR (100 MHz, CDCl_3_): δ 170.74, 169.45 (2C, CO_Ac_), 164.44 (CO_NH_), 133.18 (CH_All_), 118.43 (CH_2All_), 99.88 (C-1), 73.87 (C-5), 72.36 (C-4), 71.19 (C-3), 70.15 (CH_2All_), 66.19 (CHCl_2_), 55.33 (C-2), 20.76, 20.55 (2C, C_Ac_), 2.91 (C-6). HRMS (ESI^+^) *m*/*z*: [M + Na]^+^ calcd for C_15_H_20_Cl_2_INO_7_Na, 545.9559; found, 545.9572.

#### Allyl 3,4-Di-*O*-acetyl-2,6-dideoxy-2-trichloroacetamido-β-d-glucopyranoside (**6**)^45^

A solution of triacetate **15** (200 mg, 462 μmol, 1.0 equiv) in anhyd. DCM (5 mL) containing allyl alcohol (94 μL, 1.38 mmol, 3.0 equiv) was stirred with 4 Å MS (300 mg) under an Ar atmosphere at rt for 1 h before being cooled to 0 °C. BF_3_·OEt_2_ (116 μL, 924 μmol, 2.0 equiv) was slowly added dropwise. After stirring for 1 h at 0 °C, the reaction mixture was allowed to reach rt slowly and was stirred for 8 h at this temperature. A TLC analysis (Tol/EtOAc 3:1) showed the consumption of the starting material (*R*_*f*_ 0.55) and the presence of a new spot (*R*_*f*_ 0.65). The suspension was filtered and washed with DCM (2 × 5 mL). The filtrate was washed with water (20 mL) and brine (20 mL). The organic phase was dried over Na_2_SO_4_ and concentrated, and the residue was purified by flash chromatography (Tol/EtOAc 70:30 → 65:35) to give the known allyl glycoside **6** as a white solid (188 mg, 436 μmol, 94%). The 6-deoxy derivative **6** had *R*_*f*_ 0.45 (Tol/EtOAc 4:1). HRMS (ESI^+^) *m*/*z*: [M + Na]^+^ calcd for C_15_H_20_NO_7_Cl_3_Na, 454.0203; found, 454.0222. Other analytical data were as published.^45^

#### Allyl 3,4-Di-*O*-acetyl-2-dichloroacetamido-2,6-dideoxy-β-d-glucopyranoside (**7**)

Route a: Bu_3_SnH (48 μL, 210 μmol, 1.1 equiv) followed by Et_3_B (1.0 M in hexane, 19 μL, 19 μmol, 0.1 equiv) was added to a solution of the starting material **3** (100 mg, 191 μmol, 1.0 equiv) in anhyd. DCM at 0 °C. After stirring for 1 h at this temperature, a TLC analysis (Tol/EtOAc, 7:3) showed the consumption of the starting material and the presence of two more polar spots. MeOH was added, and after stirring for another 10 min, volatiles were evaporated. Flash chromatography purification (Tol/EtOAc 85:15 → 60:40) gave by order of elution the desired product **7** (54 mg, 71%) and the chloroacetamide derivative **8** (8 mg, 11%), both isolated as white solids.

Route b: A solution of triacetate **16** (150 mg, 376 μmol, 1.0 equiv) in anhyd. DCE (5.0 mL) containing allyl alcohol (76 μL, 1.12 mmol, 3.0 equiv) was stirred with 4 Å MS (200 mg) under an Ar atmosphere at rt for 1 h before being cooled to 0 °C. BF_3_·OEt_2_ (92 μL, 752 μmol, 2.0 equiv) was added dropwise over 5 min. The suspension was stirred overnight at this temperature, at which time a TLC follow-up (Tol/EtOAc, 3:1) indicated completion. The suspension was filtered and rinsed with DCM (10 mL). The combined filtrates were washed with water (10 mL) and brine (15 mL), dried over Na_2_SO_4_, and concentrated. Flash chromatography (Tol/EtOAc 70:30 → 55:45) gave allyl glycoside **7** as a white solid (132 mg, 286 μmol, 88%). Dichloroacetamide **7** had *R*_*f*_ 0.4 (Tol/EtOAc 4:1). ^1^H NMR (400 MHz, CDCl_3_): δ 6.69 (d, 1H, *J*_NH,2_ = 8.8 Hz, NH), 5.91 (s, 1H, CHCl_2_), 5.89–5.81 (m, 1H, CH_All_), 5.34–5.25 (m, 2H, H-3, CH_2All_), 5.21–5.17 (m, 1H, CH_2All_), 4.85 (t, 1H, *J*_3,4_ = *J*_4,5_ = 9.6 Hz, H-4), 4.67 (d, 1H, *J*_1,2_ = 8.0 Hz, H-1), 4.37–4.31 (m, 1H, CH_2All_), 4.12–4.06 (m, 1H, CH_2All_), 3.97 (dt, 1H, *J*_2,3_ = 8.4 Hz, H-2), 3.60 (dq, 1H, H-5), 2.05, 2.03 (2s, 6H, CH_3Ac_), 1.27 (d, 3H, *J*_5,6_ = 6.0 Hz, H-6). ^13^C{^1^H} NMR (100 MHz, CDCl_3_): δ 170.90, 169.55 (2C, CO_Ac_), 164.43 (CO_NH_), 133.48 (CH_All_), 117.81 (CH_2All_), 99.21 (C-1), 73.52 (C-4), 71.78 (C-3), 70.12 (C-5), 69.96 (CH_2All_), 66.26 (CHCl_2_), 55.28 (C-2), 20.66, 20.58 (2C, C_Ac_), 17.41 (C-6). HRMS (ESI^+^) *m*/*z*: [M + Na]^+^ calcd for C_15_H_21_Cl_2_NO_7_Na, 420.0593; found, 420.0593.

#### Allyl 3,4-Di-*O*-acetyl-2-chloroacetamido-2,6-dideoxy-β-d-glucopyranoside (**8**)

The 6-iodo derivative **3** (75 mg, 143 μmol, 1.0 equiv) in DCM (3 mL) was cooled to 0 °C. Bu_3_SnH (165 μL, 717 μmol, 5.0 equiv) was added, followed by Et_3_B (1 M in hexane, 29 μL, 29 μmol, 0.2 equiv). After stirring for 1 h at 0 °C, a TLC analysis (Tol/EtOAc 6:4) showed the absence of **3** (*R*_*f*_ 0.7) and appearance of a single more polar spot (*R*_*f*_ 0.5). MeOH was added, and volatiles were eliminated under reduced pressure. Flash chromatography (Tol/EtOAc 60:40 → 50:50) of the residue gave chloroacetamide **8** (39 mg, 107 μmol, 75%) as a white solid. Chloroacetamide **8** had *R*_*f*_ 0.55 (Tol/EtOAc 3:2). ^1^H NMR (400 MHz, CDCl_3_): δ 6.58 (d, 1H, *J*_2,NH_ = 8.8 Hz, NH), 5.92–5.82 (m, 1H, CH_All_), 5.33–5.25 (m, 2H, H-3, CH_2All_), 5.22–5.19 (m, 1H, CH_2All_), 4.85 (t, 1H, *J*_3,4_ = *J*_4,5_ = 9.4 Hz, H-4), 4.72 (d, 1H, *J*_1,2_ = 8.1 Hz, H-1), 4.38–4.32 (m, 1H, CH_2All_), 4.12–4.06 (m, 1H, CH_2All_), 4.04 (d, 1H, *J* = 15.1 Hz, CH_2CA_), 3.99 (d, 1H, CH_2CA_), 3.90 (ddd, 1H, H-2), 3.59 (dq, 1H, H-5), 2.05, 2.04 (2s, 6H, CH_3Ac_), 1.27 (d, 3H, *J*_5,6_ = 6.0 Hz, H-6). ^13^C{^1^H} NMR (100 MHz, CDCl_3_): δ 170.75, 169.62 (CO_Ac_), 166.23 (CO_NH_), 133.58 (CH_All_), 117.75 (CH_2All_), 99.18 (C-1), 73.58 (C-4), 71.96 (C-3), 70.07 (C-5), 69.88 (CH_2All_), 55.39 (C-2), 42.46 (CH_2CA_), 20.67, 20.61 (2C, CH_3Ac_), 17.45 (C-6). HRMS (ESI^+^) *m*/*z*: [M + Na]^+^ calcd for C_15_H_22_ClNO_7_Na, 386.0982; found, 386.0984.

#### Allyl 3,4-Di-*O*-acetyl-6-chloro-2,6-dideoxy-2-dichloroacetamido-β-d-glucopyranoside (**9**)

Sodium cyanoborohydride (180 mg, 28.6 mmol, 3.0 equiv) was added to a solution of the dichloroacetamide **3** (500 mg, 9.56 mmol, 1.0 equiv) in anhyd. DMF (9.6 mL) under an Ar atmosphere. After heating at 110 °C for 24 h, the reaction mixture was left to return to rt and diluted with water. The aq phase was extracted with DCM (3 × 30 mL), and the combined organic phases were dried over Na_2_SO_4_, filtered, and concentrated. Flash chromatography of the crude (Tol/EtOAc 85:15 → 80:20) gave a 1:2 mixture of the 6-chloro derivative **9** and the targeted 6-deoxy analogue **7** as a white solid (330 mg, 84%). An analytical sample of the 6-chlorinated side product **9** had *R*_*f*_ 0.4 (Tol/EtOAc 4:1). ^1^H NMR (400 MHz, CDCl_3_): δ 6.76 (d, 1H, *J*_2,NH_ = 8.8 Hz, NH), 5.92 (s, 1H, CHCl_2_), 5.90–5.82 (m, 1H, CH_All_), 5.40 (dd, 1H, *J*_2,3_ = 10.3 Hz, *J*_3,4_ = 9.2 Hz, H-3), 5.33–5.28 (m, 1H, CH_2All_), 5.24–5.21 (m, 1H, CH_2All_), 5.04 (t, 1H, *J*_4,5_ = 9.4 Hz, H-4), 4.77 (d, 1H, *J*_1,2_ = 8.0 Hz, H-1), 4.40–4.35 (m, 1H, CH_2All_), 4.17–4.12 (m, 1H, CH_2All_), 3.99 (ddd, 1H, H-2), 3.77 (ddd, 1H, H-5), 3.67–3.58 (m, 2H, H-6a, H-6b), 2.07, 2.05 (2s, 6H, CH_3Ac_). ^13^C{^1^H} NMR (100 MHz, CDCl_3_): δ 170.82, 169.42 (CO_Ac_), 164.51 (CO_NH_), 133.20 (CH_All_), 118.26 (CH_2All_), 99.05 (C-1), 73.88 (C-5), 71.46 (C-3), 70.38 (C-4), 70.14 (CH_2All_), 66.20 (CHCl_2_), 55.02 (C-2), 43.30 (C-6), 20.66, 20.57 (2C, CH_3Ac_). HRMS (ESI^+^) *m*/*z*: [M + NH_4_]^+^ calcd for C_15_H_24_Cl_3_N_2_O_7_ 449.0644; found, 449.0639.

#### 1,3,4-Tri-*O*-acetyl-2,6-dideoxy-2-(4-methoxybenzylidene)amino-6-iodo-β-d-glucopyranose (**11**)

Route a: 1,3,4-Tri-*O*-acetyl-2-deoxy-2-*N*-(4-methoxybenzylidene)-6-*O*-toluenesulfonyl-β-d-glucopyranose (8.0 g, 13.8 mmol, 1.0 equiv) was dissolved in butanone (69 mL), and sodium iodide (6.2 g, 41.5 mmol, 3.0 equiv) was added. After stirring at 80 °C until complete disappearance of the starting material (*R*_*f*_ 0.25) as monitored by TLC (Tol/EtOAc, 4:1), the reaction mixture was cooled to rt and concentrated to one-third (∼20 mL) under reduced pressure. DCM (200 mL) and water (300 mL) were added. The organic layer was separated, dried over Na_2_SO_4_, and concentrated in vacuo. Flash chromatography (Tol/EtOAc 90:10 → 80:20) of the residue gave iodide **11** as a white solid (6.6 g, 12.3 mmol, 89%).

Route b: Sodium iodide (1.0 g, 7.25 mmol, 2.5 equiv) was added to a solution of 1,3,4-tri-*O*-acetyl-2-deoxy-2-*N*-(4-methoxybenzylidene)-6-*O*-(2,4,6-triisopropylbenzenesulfonyl)-β-d-glucopyranose (2.0 g, 2.90 mmol, 1.0 equiv) in butanone (20 mL). The reaction was stirred at 80 °C for 16 h at which time TLC (Tol/EtOAc 7:3) showed reaction completion. The reaction mixture was allowed to reach rt and concentrated to dryness under reduced pressure. DCM (50 mL) and water (50 mL) were added. The DCM layer was separated, dried over Na_2_SO_4_, and concentrated. The crude was purified (Tol/EtOAc 85:15 → 80:20) to give iodide **11** (1.37 g, 2.57 mmol, 88%). The desired **11** had *R*_*f*_ 0.5 (Tol/EtOAc 4:1). ^1^H NMR (400 MHz, CDCl_3_): δ 8.17 (s, 1H, CH=N), 7.68–7.66 (m, 2H, H_Ar_), 6.94–6.92 (m, 2H, H_Ar_), 5.99 (d, 1H, *J*_1,2_ = 8.4 Hz, H-1), 5.45 (t, 1H, *J*_4,3_ = *J*_2,3_ = 9.6 Hz, H-3), 5.03 (t, 1H, *J*_4,5_ = 9.6 Hz, H-4), 3.85 (s, 3H, OCH_3_), 3.68 (ddd, H-5), 3.46 (dd, 1H, H-2), 3.40 (dd, 1H, *J*_5,6a_ = 2.8 Hz, *J*_6a,6b_ = 11.2 Hz, H-6a), 3.24 (dd, 1H, *J*_5,6b_ = 6.1 Hz, H-6b), 2.08, 2.05, 1.90 (3s, 9H, CH_3Ac_). ^13^C{^1^H} NMR (100 MHz, CDCl_3_): δ 169.73, 169.54, 168.69 (3C, CO_Ac_), 164.28 (CH=N), 162.33 (C_q,Ar_), 130.24 (C_Ar_), 128.38 (C_q,Ar_), 114.08 (C_Ar_), 92.92 (C-1, ^1^*J*_C,H_ = 167 Hz), 73.28 (C-5), 73.02 (C-3), 72.99 (C-2), 72.47 (C-4), 55.40 (OCH_3_), 20.76, 20.48 (3C, C_Ac_), 3.38 (C-6). HRMS (ESI^+^) *m*/*z*: [M + H]^+^ calcd for C_20_H_25_INO_8_, 534.0625; found, 534.0628.

#### 1,3,4-Tri-*O*-acetyl-2,6-dideoxy-2-(4-methoxybenzylidene)amino-β-d-glucopyranose (**12**)

A solution of the 6-iodo derivative **11** (6.4 g, 12.0 mmol, 1.0 equiv) in anhyd. DCM (100 mL) was cooled to 0 °C. Tributyltin hydride (3.04 mL, 13.2 mmol, 1.1 equiv) was added, followed by the addition of Et_3_B (1 M in hexane, 1.2 mL, 1.2 mmol, 1.0 equiv). After stirring for 1 h at 0 °C, a TLC monitoring (DCM/EtOAc 9:1) showed the absence of the starting material (*R*_*f*_ 0.65) and the presence of a new spot (*R*_*f*_ 0.6). MeOH (2 mL) was added, and after 15 min, volatiles were evaporated. Flash chromatography of the crude residue (Tol/EtOAc 81:18 → 80:20) gave the 6-deoxy derivative **12** as a white solid (4.55 g, 11.1 mmol, 93%). Triacetate **12** had *R*_*f*_ 0.5 (Tol/EtOAc 4:1). ^1^H NMR (400 MHz, CDCl_3_): δ 8.17 (s, 1H, CH=N), 7.69–7.66 (m, 2H, H_Ar_), 6.95–6.91 (m, 2H, H_Ar_), 5.95 (d, 1H, *J*_1,2_ = 8.4 Hz, H-1), 5.41 (t, 1H, *J*_3,4_ = *J*_2,3_ = 9.6 Hz, H-3), 4.89 (t, 1H, *J*_4,5_ = 9.6 Hz, H-4), 3.89–3.85 (m, 4H, H-5, OCH_3_), 3.42 (dd, 1H, H-2), 2.07, 2.03, 1.89 (3s, 9H, CH_3Ac_), 1.30 (d, 3H, *J*_5,6_ = 6.2 Hz, H-6). ^13^C{^1^H} NMR (100 MHz, CDCl_3_): δ 170.11, 169.53, 168.84 (3C, CO_Ac_), 163.92 (CH = N), 162.25 (C_q,Ar_), 130.19 (C_Ar_), 128.50 (C_q,Ar_), 114.06 (C_Ar_), 93.11 (C-1, ^1^*J*_C,H_ = 168 Hz), 73.46 (C-4), 73.40 (C-3), 73.30 (C-2), 70.92 (C-5), 55.39 (OCH_3_), 20.79, 20.76, 20.52 (3C, C_Ac_), 17.47 (C-6). HRMS (ESI^+^) *m*/*z*: [M + H]^+^ calcd for C_20_H_26_NO_8_, 408.1658; found, 408.1654.

#### 1,3,4-Tri-*O*-acetyl-2-amino-2,6-dideoxy-β-d-glucopyranose (**13**)

Route a: Zn (108 mg, 1.65 mmol, 8.0 equiv) and AcOH (94 μL, 1.65 mmol, 8.0 equiv) were added to a solution of azide **14** (65 mg, 206 μmol, 1.0 equiv) in anhyd. THF (4 mL). The reaction mixture was stirred for 6 h at rt. The suspension was filtered over a bed of Celite and washed with THF (2 × 5 mL). The combined filtrate was concentrated to give the crude amine **13** as a 1:5 α/β mixture (54 mg, 189 μmol, 91%). The target **13** had HRMS (ESI^+^) *m*/*z*: [M + Na]^+^ calcd for C_12_H_19_NO_7_Na, 312.1054; found, 312.1051.

Route b: Imine **12** (6.0 g, 14.7 mmol, 1.0 equiv) was dissolved in acetone (60 mL), and commercially available 2 N HCl in Et_2_O (8.1 mL, 16.2 mmol, 1.1 equiv) was added. The reaction mixture was heated for 30 min at 60 °C and a precipitate appeared. After precipitation completion at 0 °C, the precipitate was filtered and washed thoroughly with Et_2_O. The target **13** was isolated as a white solid (4.04 g, 12.4 mmol, 84%). Amine **13**, isolated in the form of the hydrochloride salt, had *R*_*f*_ 0.1 (EtOAc/MeOH 4:1). ^1^H NMR (400 MHz, DMSO-*d*_6_): δ 8.77 (brs, 3H, NH_3_^+^), 5.85 (d, 1H, *J*_1,2_ = 8.4 Hz, H-1), 5.29 (dd, 1H, *J*_4,3_ = 9.2 Hz, *J*_2,3_ = 10.0 Hz, H-3), 4.69 (t, 1H, H-4), 3.83–3.76 (m, 1H, H-5), 3.53 (dd, 1H, H-2), 2.16, 2.03, 2.01 (3s, 9H, CH_3Ac_), 1.12 (d, 3H, *J*_5,6_ = 6.1 Hz, H-6). ^13^C{^1^H} NMR (100 MHz, DMSO-*d*_6_): δ 170.22, 169.99, 169.17 (3C, CO_Ac_), 90.53 (C-1), 73.24 (C-4), 70.79 (C-3), 70.46 (C-5), 52.87 (C-2), 21.41, 21.31, 20.87 (3C, C_Ac_), 17.44 (C-6). HRMS (ESI^+^) *m*/*z*: [M + H]^+^ calcd for C_12_H_20_NO_7_, 290.1240; found, 290.1250.

#### 1,3,4-Tri-*O*-acetyl-2-azido-2-deoxy-α/β-d-glucopyranose (**14**)

Route a: Bu_3_SnH (230 μL, 998 μmol, 1.1 equiv) and Et_3_B (1 M in Hexane, 91 μL, 91 μmol, 0.1 equiv) were successively added to a solution of 1,3,4-tri-*O*-acetyl-2-azido-2,6-dideoxy-6-iodo-α/β-d-glucopyranose (400 mg, 907 μmol, 1.0 equiv) in anhyd. DCM (5 mL) cooled to 0 °C. After 30 min at this temperature and following a TLC analysis, more Bu_3_SnH (42 μL, 181 μmol, 0.2 equiv) was added. The reaction was left for another 30 min at 0 °C, at which point a TLC follow-up (Tol/EtOAc 4:1) revealed completion. MeOH was added, and after 10 min, volatiles were evaporated. Flash chromatography (Tol/EtOAc 90:10 → 88:12) gave the 6-deoxy derivative **14** as a 1:5 α/β mixture (250 mg, 793 μmol, 87%). HRMS (ESI^+^) *m*/*z*: [M + NH_4_]^+^ calcd for C_19_H_27_N_4_O_10_S, 333.1407; found, 333.1405.

Route b: Sodium azide (144 mg, 2.21 mmol, 8.0 equiv) was dissolved in water (3 mL). DCM (6 mL) was added, and the biphasic mixture was cooled to 0 °C. Trifluoromethane-sulfonic anhydride (69 μL, 415 μmol, 1.5 equiv) was added dropwise. After stirring the reaction mixture for 2 h at 0 °C, the organic phase was separated and washed with satd aq NaHCO_3_. Amine hydrochloride **13** (90 mg, 277 μmol, 1.0 equiv) was dissolved in MeOH, and NaHCO_3_ (23 mg, 277 μmol, 1.0 equiv) was added. The reaction mixture was cooled to 0 °C, and the DCM extract was immediately added to the solution. The reaction mixture was allowed to reach rt and stirred overnight at this temperature. Following a TLC analysis (Tol/EtOAc 1:1), the mixture was concentrated in vacuo. Flash chromatography (Tol/EtOAc 90:10 → 88:12) of the crude gave **14** as a colorless oil (45 mg, 143 μmol, 51%).

The β isomer had *R*_*f*_ 0.6 (Tol/EtOAc 4:1). ^1^H NMR (400 MHz, CDCl_3_): δ 5.55 (d, 1H, *J*_1,2_ = 8.4 Hz, H-1), 5.06 (dd, 1H, *J*_2,3_ = 10.0 Hz, *J*_3,4_ = 9.6 Hz, H-3), 4.78 (t, 1H, *J*_4,5_ = 9.6 Hz, H-4), 3.69 (dq, 1H, H-5), 3.64 (dd, 1H, H-2), 2.20, 2.10, 2.06 (3s, 9H, CH_3Ac_), 1.24 (d, 3H, *J*_5,6_ = 6.2 Hz, H-6). ^13^C{^1^H} NMR (100 MHz, CDCl_3_): δ 169.81, 169.77, 168.64 (3C, CO_Ac_), 92.50 (C-1, ^1^*J*_C,H_ = 168 Hz), 73.09 (C-4), 72.68 (C-3), 70.96 (C-5), 62.94 (C-2), 20.84, 20.60 (3C, C_Ac_), 17.22 (C-6).

#### 1,3,4-Tri-*O*-acetyl-2,6-dideoxy-2-trichloroacetamido-β-d-glucopyranose (**15**)

The 6-deoxy derivative **12** (300 mg, 737 μmol, 1.0 equiv) was dissolved in acetone (6 mL), and 1 N HCl in Et_2_O (0.81 mL, 810 μmol, 1.1 equiv) was added. The reaction mixture was heated to reflux for 10 min, at which time a precipitate appeared. The system was cooled to 0 °C to allow complete precipitation. The precipitate was filtered and washed with Et_2_O (2 × 5 mL). Pyridine (297 μL, 3.68 mmol, 5.0 equiv) and then trichloroacetyl chloride (164 μL, 1.47 mmol, 2.0 equiv) were added to a suspension of the precipitate in DCM (6 mL) at 0 °C. After 30 min, the suspension had turned into a clear solution. At completion, MeOH (1 mL) was added. Volatiles were evaporated and coevaporated with toluene (3 × 5 mL). Flash chromatography (Tol/EtOAc 85:15 → 80:20) gave the triacetate **15** as a white solid (230 mg, 531 μmol, 72%). Trichloroacetamide **15** had *R*_*f*_ 0.5 (Tol/EtOAc 4:1). ^1^H NMR (400 MHz, CDCl_3_): δ 7.03 (d, 1H, *J*_2,NH_ = 9.6 Hz, NH), 5.80 (d, 1H, *J*_1,2_ = 10.0 Hz, H-1), 5.30 (dd, 1H, *J*_4,3_ = 9.6 Hz, *J*_2,3_ = 10.8 Hz, H-3), 4.95 (t, 1H, *J*_4,5_ = 9.6 Hz, H-4), 4.28 (ddd, 1H, H-2), 3.76 (dq, 1H, H-5), 2.13, 2.10, 2.07 (3s, 9H, CH_3Ac_), 1.30 (d, 3H, *J*_5,6_ = 6.2 Hz, H-6). ^13^C{^1^H} NMR (100 MHz, CDCl_3_): δ 171.32, 169.44, 169.39 (3C, CO_Ac_), 162.18 (CO_NH_), 92.18 (CCl_3_), 92.12 (C-1), 72.74 (C-4), 71.86 (C-3), 71.45 (C-5), 55.03 (C-2), 20.70, 20.60, 20.52 (3C, C_Ac_), 17.30 (C-6). HRMS (ESI^+^) *m*/*z*: [M + NH_4_]^+^ calcd for C_14_H_22_Cl_3_N_2_O_8_, 451.0442; found, 451.0444.

#### 1,3,4-Tri-*O*-acetyl-2,6-dideoxy-2-dichloroacetamido-β-d-glucopyranose (**16**)

Imine **12** (330 mg, 810 μmol, 1.0 equiv) was dissolved in acetone (9 mL), and HCl (1 N in Et_2_O, 0.89 mL, 892 μmol, 1.1 equiv) was added. The reaction mixture was stirred at 60 °C for 30 min while becoming turbid over time. The system was cooled to 0 °C to allow complete precipitation. The precipitate was filtered and washed thoroughly with Et_2_O (2 × 5 mL). Anhyd. pyridine (327 μL, 4.05 mmol, 5.0 equiv) was added to a suspension of the crude amine **13** in DCM (10 mL). The suspension was cooled to 0 °C, and dichloroacetic anhydride (117 μL, 1.21 mmol, 1.5 equiv) was added slowly. After stirring for 1 h at this temperature, a TLC follow-up (Tol/EtOAc 4:1) revealed reaction completion. MeOH (1.0 mL) was added, and stirring was pursued at rt for an additional 5 min. After dilution with DCM (10 mL), the organic layer was washed with 1 N aq HCl (20 mL), satd aq NaHCO_3_ (20 mL), and brine (20 mL). The organic phase was dried over Na_2_SO_4_, filtered, and concentrated to dryness. Flash chromatography (Tol/EtOAc 80:20 → 75:25) gave the desired **16** as a white solid (250 mg, 626 μmol, 77%). The dichloroacetamide **16** had *R*_*f*_ 0.4 (Tol/EtOAc 4:1). ^1^H NMR (400 MHz, CDCl_3_): δ 6.92 (d, 1H, *J*_2,NH_ = 9.6 Hz, NH), 5.88 (s, 1H, CHCl_2_), 5.80 (d, 1H, *J*_1,2_ = 8.8 Hz, H-1), 5.31 (dd, 1H, *J*_4,3_ = 9.2 Hz, *J*_2,3_ = 10.8 Hz, H-3), 4.90 (t, 1H, *J*_4,5_ = 9.6 Hz, H-4), 4.27 (ddd, 1H, H-2), 3.76 (_dq_, 1H, H-5), 2.12, 2.09, 2.06 (3s, 9H, CH_3Ac_), 1.29 (d, 3H, *J*_5,6_ = 6.2 Hz, H-6). ^13^C{^1^H} NMR (100 MHz, CDCl_3_): δ 171.24, 169.53, 169.42 (3C, CO_Ac_), 164.70 (CO_NH_), 92.10 (C-1, ^1^*J*_C,H_ = 165.8 Hz), 72.96 (C-4), 71.90 (C-3), 71.33 (C-5), 66.10 (CHCl_2_), 53.80 (C-2), 20.72, 20.61, 20.55 (3C, C_Ac_), 17.31 (C-6). HRMS (ESI^+^) *m*/*z*: [M + NH_4_]^+^ calcd for C_14_H_23_Cl_2_N_2_O_8_, 417.0832; found, 417.0835.

#### 1,3,4-Tri-*O*-acetyl-2,6-dideoxy-2-trifluoroacetamido-β-d-glucopyranose (**17**)

To a solution of triacetate **12** (400 mg, 982 μmol, 1.0 equiv) in acetone (12 mL) was added 1 N HCl in Et_2_O (2.94 mL, 2.94 mmol, 3.0 equiv). The solution was stirred at 80 °C for 2 h and a white precipitate appeared. The suspension was allowed to reach rt, and anhyd. DCM (10 mL) was added. The solution was cooled to 0 °C, and pyridine (396 μL, 4.91 mmol, 5.0 equiv) was added, followed by trifluoroacetic anhydride (273 μL, 1.96 mmol, 2.0 equiv). After stirring at 0 °C for 1 h, MeOH (1 mL) was added to the obtained yellowish solution. After another 20 min while the temperature reached rt, volatiles were removed. The residue was diluted with DCM (30 mL), and the organic phase was washed with water, dried over Na_2_SO_4_, and concentrated. Flash chromatography (Tol/MeCN 88:12 → 86:14) gave the desired **17** as a white solid (290 mg, 629 μmol, 77%). Trifluoroacetamide **17** had *R*_*f*_ 0.35 (Tol/MeCN 6:1). ^1^H NMR (400 MHz, CDCl_3_): δ 7.42 (d, 1H, *J*_2,NH_ = 9.7 Hz, NH), 5.74 (d, 1H, *J*_1,2_ = 8.8 Hz, H-1), 5.31 (dd, 1H, *J*_4,3_ = 9.5 Hz, *J*_2,3_ = 10.7 Hz, H-3), 4.88 (t, 1H, *J*_4,5_ = 9.6 Hz, H-4), 4.38–4.31 (m, 1H, H-2), 3.76 (dq, 1H, H-5), 2.12, 2.08, 2.06 (3s, 9H, CH_3Ac_), 1.28 (d, 3H, *J*_5,6_ = 6.2 Hz, H-6). ^13^C{^1^H} NMR (100 MHz, CDCl_3_): δ 171.80, 169.64, 169.61 (3C, CO_Ac_), 157.50 (CO_NH_, *J*_C,F_ = 37.4 Hz), 115.62 (CF_3_, *J*_C,F_ = 286.1 Hz), 91.79 (C-1, ^1^*J*_C,H_ = 166.0 Hz), 73.05 (C-4), 72.24 (C-3), 71.18 (C-5), 53.38 (C-2), 20.59, 20.52, 20.31 (3C, C_Ac_), 17.22 (C-6). HRMS (ESI^+^) *m*/*z*: [M + NH_4_]^+^ calcd for C_14_H_22_F_3_N_2_O_8_, 403.1322; found, 403.1323.

#### 1,3,4-Tri-*O*-acetyl-2,6-dideoxy-2-(2,2,2-trichloroethoxycarbonylamino)-β-d-glucopyranose (**18**)

2 N HCl in Et_2_O (1.62 mL, 3.24 mmol, 1.1 equiv) was added to a solution of imine **12** (1.2 g, 2.94 mmol, 1.0 equiv) in acetone, and the reaction mixture was refluxed for 1 h. A white precipitate was formed, which was filtered off and washed thoroughly with Et_2_O. Pyridine (476 μL, 5.89 mmol, 2.0 equiv) and 2,2,2-trichloroethoxycarbonyl chloride (609 μL, 4.42 mmol, 1.5 equiv) were added to a suspension of the crude amine in DCM (20 mL) at 0 °C. After stirring for 1 h while allowing the reaction to reach rt, a TLC (Tol/EtOAc 4:1) follow-up revealed reaction completion. MeOH (1.0 mL) was added, and after stirring for another 10 min, volatiles were evaporated. Flash chromatography (Tol/EtOAc 85:15 → 80:20) gave triacetate **18** as a white solid (1.1 g, 2.37 mmol, 81%). Triacetate **18** had *R*_*f*_ 0.45 (Tol/EtOAc, 4:1). ^1^H NMR (400 MHz, CDCl_3_): δ 5.72 (d, 1H, *J*_1,2_ = 8.7 Hz, H-1), 5.47 (d, 1H, *J*_2,NH_ = 9.5 Hz, NH), 5.21 (dd, 1H, *J*_4,3_ = 9.6 Hz, *J*_2,3_ = 10.3 Hz, H-3), 4.86 (t, 1H, *J*_4,5_ = 9.6 Hz, H-4), 4.77–4.71 (m, 2H, CH_2Troc_), 4.00–3.93 (m, 1H, H-2), 3.74 (dq, 1H, H-5), 2.11, 2.07, 2.05 (3s, 9H, CH_3Ac_), 1.26 (d, 3H, *J*_5,6_ = 6.2 Hz, H-6). ^13^C{^1^H} NMR (100 MHz, CDCl_3_): δ 171.94, 169.66, 169.38 (3C, CO_Ac_), 154.23 (CO_NH_), 95.48 (CCl_3_), 92.34 (C-1), 74.48 (CH_2Troc_), 73.23 (C-4), 72.24 (C-3), 71.01 (C-5), 55.39 (C-2), 20.82, 20.64, 20.61 (3C, C_Ac_), 17.31 (C-6). HRMS (ESI^+^) *m*/*z*: [M + NH_4_]^+^ calcd for C_15_H_24_Cl_3_N_2_O_9_, 481.0542; found, 481.0544.

#### Allyl 3,4-Di-*O*-acetyl-2,6-dideoxy-2-trifluoroacetamido-β-d-glucopyranoside (**19**)

Route a: Et_3_B (1 M in hexane, 8.5 mL, 8.5 mmol, 0.1 equiv) was added dropwise to a solution of the 6-iodo precursor **27** (43.4 g, 85.2 mmol, 1.0 equiv) and Bu_3_SnH (21.5 mL, 93.7 mmol, 1.1 equiv) in anhyd. DCM (600 mL) at 0 °C. The solution was stirred for 1 h at 0 °C, at which time a follow-up by TLC (Tol/EtOAc 7:3) indicated the absence of the starting material (*R*_*f*_ 0.5) and the presence of a less polar product (*R*_*f*_ 0.4). Volatiles were evaporated, and the crude was purified by flash chromatography (Tol/EtOAc 5:1 → 3:1). The desired **19** (31.4 g, 81.9 mmol, 96%) was obtained as a white solid. Analytical data of **19** were as described below.

Route b: Allyl alcohol (52 μL, 779 μmol, 3.0 equiv) was added to triacetate **17** (100 mg, 260 μmol, 1.0 equiv) in anhyd. DCM (3 mL). The mixture was stirred with activated 4 Å MS (200 mg) under Ar for 1 h at rt and cooled to 0 °C. BF_3_·OEt_2_ (70 μL, 571 mmol, 2.2 equiv) was added. The suspension was stirred for 12 h while slowly reaching rt. Following a TLC control (DCM/EtOAc, 9:1), the suspension was filtered and thoroughly washed with DCM. The combined filtrates were washed with water (10 mL) and brine (10 mL). Flash chromatography (Tol/EtOAc 80:20 → 75:25) of the crude gave the desired **19** as a white solid (92 mg, 240 μmol, 92%). Allyl glycoside **19** had *R*_*f*_ 0.4 (Tol/EtOAc 7:3). ^1^H NMR (400 MHz, CDCl_3_): δ 6.82 (d, 1H, *J*_NH,2_ = 9.0 Hz, NH), 5.89–5.79 (m, 1H, CH_All_), 5.30–5.23 (m, 2H, H-3, CH_2All_), 5.22–5.18 (m, 1H, CH_2All_), 4.86 (t, 1H, *J*_4,3_ = *J*_4,5_ = 9.5 Hz, H-4), 4.63 (d, 1H, *J*_1,2_ = 8.3 Hz, H-1), 4.37–4.32 (m, 1H, CH_2All_), 4.11–4.03 (m, 2H, H-2, CH_2All_), 3.61 (dq, 1H, H-5), 2.05, 2.04 (2s, 6H, CH_3Ac_), 1.28 (d, 3H, *J*_5,6_ = 6.2 Hz, H-6). ^13^C{^1^H} NMR (100 MHz, CDCl_3_): δ 171.30, 169.52 (2C, CO_Ac_), 157.35 (CO_NH_, q, *J*_C,F_ = 37.3 Hz), 133.25 (CH_All_), 117.81 (CH_2All_), 115.55 (CF_3_, q, *J*_C,F_ = 286.1 Hz), 98.99 (C-1, ^1^*J*_C,H_ = 161.0 Hz), 73.42 (C-4), 72.02 (C-3), 70.11 (C-5), 69.87 (CH_2All_), 54.99 (C-2), 20.61, 20.39 (2C, CH_3Ac_), 17.36 (C-6). HRMS (ESI^+^) *m*/*z*: [M + NH_4_]^+^ calcd for C_15_H_24_F_3_N_2_O_7_, 401.1536; found, 401.1530.

#### Allyl 3,4-Di-*O*-acetyl-2,6-dideoxy-2-(2,2,2-trichloroethoxyacetamido)-β-d-glucopyranoside (**20**)

A solution of triacetate **18** (1.0 g, 2.16 mmol, 1.0 equiv) in anhyd. DCM (10 mL) containing allyl alcohol (440 μL, 6.47 mmol, 3.0 equiv) was stirred with 4 Å MS (500 mg) in an Ar atmosphere at rt for 1 h before being cooled to 0 °C. BF_3_·OEt_2_ (533 μL, 4.31 mmol, 2.0 equiv) was added dropwise, and the reaction mixture was allowed to reach rt. After 36 h, a TLC (Tol/EtOAc, 3:1) follow-up indicated complete conversion. The suspension was filtered and rinsed with DCM (10 mL). The combined filtrates were washed with water (20 mL) and 50% aq NaHCO_3_ (20 mL), dried over Na_2_SO_4_, and concentrated to dryness. Flash chromatography (Tol/EtOAc 90:10 → 88:12) gave the desired **20** as a white solid (800 mg, 1.80 mmol, 83%). The allyl glycoside **20** had *R*_*f*_ 0.15 (Tol/EtOAc, 9:1). ^1^H NMR (400 MHz, CDCl_3_): δ 5.91–5.83 (m, 1H, CH_All_), 5.32–5.27 (m, 1H, CH_2All_), 5.24–5.20 (m, 2H, CH_2All_, H-3), 5.13 (d, 1H, *J*_NH,2_ = 8.4 Hz, NH), 4.84 (t, 1H, *J*_4,5_ = *J*_3,4_ = 9.6 Hz, H-4), 4.79 (d, 1H, CH_2,Troc_), 4.70 (d, 1H, *J* = 11.9 Hz, CH_2,Troc_), 4.61 (d, 1H, *J*_1,2_ = 8.4 Hz, H-1), 4.40–4.35 (m, 1H, CH_2All_), 4.12–4.07 (m, 1H, CH_2All_), 3.71–3.64 (m, 1H, H-2), 3.57 (dq, 1H, H-5), 2.06, 2.05 (2s, 6H, CH_3Ac_), 1.25 (d, 3H, *J*_5,6_ = 6.2 Hz, H-6). ^13^C{^1^H} NMR (100 MHz, CDCl_3_): δ 170.72, 169.69 (2C, CO_Ac_), 154.10 (CO_NH_), 133.57 (CH_All_), 117.68 (CH_2All_), 99.63 (C-1, *J*_C,H_ = 162.4 Hz), 95.50 (CCl_3_), 74.44 (CH_2,Troc_), 73.74 (C-4), 72.04 (C-3), 70.00 (CH_2All_), 69.94 (C-5), 56.51 (C-2), 20.68, 20.65 (2C, C_Ac_), 17.41 (C-6). HRMS (ESI^+^) *m*/*z*: [M + NH_4_]^+^ calcd for C_16_H_26_Cl_3_N_2_O_8_, 479.0749; found, 479.0740.

#### Phenyl 3,4-Di-*O*-acetyl-2,6-dideoxy-2-trifluoroacetamido-1-thio-β-d-glucopyranoside (**21**)

A solution of triacetate **17** (200 mg, 519 μmol, 1.0 equiv) and thiophenol (158 μL, 1.55 mmol, 3.0 equiv) in anhyd. DCM (6 mL) was stirred with activated 4 Å MS (300 mg) for 1 h and cooled to −10 °C. BF_3_·OEt_2_ (128 μL, 1.03 mmol, 2.0 equiv) was added, and after reaching rt slowly, the reaction mixture was stirred overnight. The suspension was filtered and washed thoroughly with DCM (15 mL). The organic phase was washed with water (10 mL), satd aq NaHCO_3_ (10 mL), and brine (10 mL); dried over Na_2_SO_4_; and concentrated to dryness. Flash chromatography (Tol/EtOAc 90:10 → 86:14) gave the desired **21** as a white solid (190 mg, 436 μmol, 84%). The phenyl thioglycoside **21** had *R*_*f*_ 0.5 (Tol/EtOAc 4:1). ^1^H NMR (400 MHz, CDCl_3_): δ 7.52–7.48 (m, 2H, H_Ar_), 7.35–7.30 (m, 3H, H_Ar_), 7.07 (d, 1H, *J*_2,NH_ = 9.6 Hz, NH), 5.27 (dd, 1H, *J*_4,3_ = 9.8 Hz, *J*_2,3_ = 10.0 Hz, H-3), 4.82 (t, 1H, *J*_4,5_ = 9.6 Hz, H-4), 4.75 (d, 1H, *J*_1,2_ = 10.4 Hz, H-1), 4.14–4.06 (m, 1H, H-2), 3.64 (dq, 1H, H-5), 2.01, 1.93 (2s, 6H, CH_3Ac_), 1.29 (d, 3H, *J*_5,6_ = 6.2 Hz, H-6). ^13^C{^1^H} NMR (100 MHz, CDCl_3_): δ 171.64, 169.39 (2C, CO_Ac_), 157.15 (CO_NH_, *J*_C,F_ = 37.4 Hz), 133.42 (C_Ar_), 131.43 (C_q,Ar_), 129.06, 128.65 (C_Ar_), 115.64 (CF_3_, *J*_C,F_ = 286.1 Hz), 85.98 (C-1, ^1^*J*_C,H_ = 156 Hz), 74.47 (C-5), 73.61 (C-3), 73.15 (C-4), 53.47 (C-2), 20.46, 20.36 (2C, C_Ac_), 17.64 (C-6). HRMS (ESI^+^) *m*/*z*: [M + NH_4_]^+^ calcd for C_18_H_24_F_3_N_2_O_6_S, 453.1301; found, 453.1289.

#### 4-Methoxyphenyl 3,4-Di-*O*-acetyl-2-azido-2-deoxy-α/β-d-glucopyranoside (**23**)

A solution of triacetate **14** (120 mg, 312 μmol, 1.0 equiv) and 4-methoxyphenol (116 mg, 935 μmol, 3.0 equiv) in anhyd. DCM (5 mL) was stirred with activated MS 4 Å (250 mg) for 1 h. The suspension was cooled to 0 °C, and BF_3_·OEt_2_ (115 μL, 935 mmol, 3.0 equiv) was added. After warming up to rt, the reaction mixture was heated at 40 °C for 24 h. A TLC analysis (Tol/EtOAc 4:1) showed the presence of a new spot (*R*_*f*_ 0.65) migrating closely to the starting material (*R*_*f*_ 0.6). After reaching rt, the reaction mixture was filtered and washed with DCM (2 × 5 mL). The organic phase was washed with water, satd aq NaHCO_3_, and brine; dried over Na_2_SO_4_; filtered; and concentrated to dryness. Flash chromatography (Tol/EtOAc 90:10) gave the target glycoside **23** as a 2:1 α/β mixture (105 mg, 273 μmol, 77%). The obtained white solid had *R*_*f*_ 0.7 (Tol/Acetone 4:1). The α anomer had ^1^H NMR (400 MHz, CDCl_3_): δ 7.08–7.05 (m, 2H, H_Ar_), 6.89–6.85 (m, 2H, H_Ar_), 5.66 (dd, 1H, *J*_3,4_ = 9.6 Hz, H-3), 5.46 (d, 1H, *J*_1,2_ = 3.6 Hz, H-1), 4.88 (t, 1H, *J*_4,5_ = 9.6 Hz, H-4), 4.11 (dq, 1H, H-5), 3.80 (s, 3H, OCH_3_), 3.41 (dd, 1H, *J*_2,3_ = 10.8 Hz, H-2), 2.14, 2.08 (2s, 6H, CH_3Ac_), 1.21 (d, 3H, *J*_5,6_ = 6.3 Hz, H-6). ^13^C{^1^H} NMR (CDCl_3_): δ 170.10, 169.98 (2C, CO_Ac_), 155.51, 150.32, 118.73, 117.84, 114.84, 114.68 (C_Ar_), 97.41 (C-1, ^1^*J*_C,H_ = 174.4 Hz), 73.80 (C-4), 70.25 (C-3), 66.16 (C-5), 61.16 (C-2), 55.67 (OCH_3_), 20.71, 20.67 (CH_3Ac_), 17.28 (C-6). HRMS (ESI^+^) *m*/*z*: [M + NH_4_]^+^ calcd for C_17_H_25_N_4_O_7_, 397.1718; found, 397.1711.

The β anomer had ^1^H NMR (400 MHz, CDCl_3_): δ 7.08–7.05 (m, 2H, H_Ar_), 6.89–6.85 (m, 2H, H_Ar_), 5.03 (dd, 1H, *J*_3,4_ = 9.6 Hz, *J*_2,3_ = 10.4 Hz, H-3), 4.91–4.82 (m, 2H, H-4, H-1), 3.78 (s, 3H, OCH_3_), 3.75 (dd, 1H, *J*_1,2_ = 8.1 Hz, H-2), 3.65(dq, 1H, *J*_4,5_ = 9.6 Hz, H-5), 2.12, 2.06 (2s, 6H, CH_3Ac_), 1.30 (d, 3H, *J*_5,6_ = 6.2 Hz, H-6). ^13^C{^1^H} NMR (CDCl_3_): δ 169.94, 169.82 (2C, CO_Ac_), 155.90, 150.86, 118.73, 117.85, 116.02, 114.84, 114.77, 114.68 (C_Ar_), 101.41 (C-1, ^1^*J*_C,H_ = 162.0 Hz), 73.31 (C-4), 72.41 (C-3), 70.17 (C-5), 63.92 (C-2), 55.77 (OCH_3_), 20.71, 20.67 (CH_3Ac_), 17.42 (C-6).

#### 1,3,4,6-Tetra-*O*-acetyl-2-deoxy-2-trifluoroacetamido-β-d-glucopyranose (**24**)^58^

Anhyd. pyridine (42.1 mL, 521 mmol, 4.0 equiv) was added to a suspension of commercially available tetraacetate **1** (50.0 g, 130 mmol, 1.0 equiv) in anhyd. DCM (370 mL). The suspension was cooled to 0 °C. Trifluoroacetic anhydride (36.7 mL, 260 mmol, 2.0 equiv) was added slowly, and the mixture was stirred for 1 h while the bath temperature was allowed to reach rt. A follow-up by TLC (Tol/EtOAc 7:3) indicated the absence of **1** and the presence of a less polar product (*R*_*f*_ 0.25). MeOH (30 mL) was added, and after an additional 30 min at rt, DCM (200 mL) was added. The organic layer was washed with 1 N aq HCl (800 mL) and satd aq NaHCO_3_ (600 mL), dried over Na_2_SO_4_, filtered, and concentrated to dryness. The crude **24** (57.7 g, quant.) was obtained as a pale yellow solid. An analytical sample of the trifluoroacetamide target had *R*_*f*_ 0.35 (Tol/MeCN 4:1). ^1^H NMR (400 MHz, CDCl_3_): δ 7.15 (d, 1H, *J*_NH,2_ = 9.6 Hz, NH), 5.77 (d, 1H, *J*_1,2_ = 8.7 Hz, H-1), 5.34 (dd, 1H, *J*_3,2_ = 10.6 Hz, *J*_3,4_ = 9.4 Hz, H-3), 5.15 (t, 1H, *J*_4,5_ = 9,6 Hz, H-4), 4.41–4.33 (m, 1H, H-2), 4.30 (dd, 1H, *J*_6a,5_ = 4.8 Hz, H-6a), 4.18 (dd, 1H, *J*_6b,5_ = 2.3 Hz, *J*_6a,6b_ = 12.5 Hz, H-6b), 3.91 (ddd, 1H, H-5), 2.14, 2.12, 2.08, 2.07 (4s, 12H, CH_3Ac_). ^13^C{^1^H} NMR (100 MHz, CDCl_3_): δ 171.63, 170.59, 169.37, 169.30 (4C, CO_Ac_), 157.50 (CO_NH_, q, *J*_C,F_ = 37.6 Hz), 115.65 (CF_3_, q, *J*_C,F_ = 286.1 Hz), 91.86 (C-1, ^1^*J*_C,H_ = 166.2 Hz), 73.04 (C-5), 72.08 (C-3), 67.91 (C-4), 61.62 (C-6), 53.26 (C-2), 20.61, 20.46, 20.33 (4C, C_Ac_). HRMS (ESI^+^) *m*/*z*: [M + Na]^+^ calcd for C_16_H_20_F_3_NO_10_Na, 466.0937; found, 466.0931.

#### Allyl 3,4,6-Tri-*O*-acetyl-2-deoxy-2-trifluoroacetamido-β-d-glucopyranoside (**25**)

A solution of the crude trifluoroacetamide **24** (57.7 g, 130 mmol, 1.0 equiv) and allyl alcohol (26.4 mL, 387 mmol, 3.0 equiv) in anhyd. DCM (120 mL) was stirred with freshly activated MS 4 Å (17 g) for 30 min under an Ar atmosphere. The suspension was cooled to 0 °C, and BF_3_·OEt_2_ (35 mL, 284 mmol, 2.2 equiv) was added dropwise. The bath was allowed to reach rt, and the reaction mixture was stirred for 48 h at this temperature. A TLC (DCM/EtOAc 9:1) follow-up indicated the conversion of the starting material (*R*_*f*_ 0.25) into a less polar compound (*R*_*f*_ 0.35). The suspension was passed through a fitted funnel, and solids were rinsed with DCM (2 × 50 mL). The organic layer was washed with water and satd aq NaHCO_3_, dried over Na_2_SO_4_, filtered, and concentrated in vacuo. Flash chromatography (DCM/EtOAc 98:2 → 90:10) of the residue gave the desired allyl glycoside **25** as a white solid (56.3 g, 127 mmol, 98%) and had *R*_*f*_ 0.6 (Tol/EtOAc 2:1). ^1^H NMR (400 MHz, CDCl_3_): δ 6.63 (bs, 1H, NH), 5.90–5.80 (m, 1H, CH_All_), 5.35–5.30 (t, 1H, *J*_2,3_ = 10.2 Hz, H-3), 5.26–5.22 (m, 2H, CH_2All_), 5.12 (t, 1H, *J*_4,3_ = *J*_4,5_ = 9.7 Hz, H-4), 4.70 (d, 1H, *J*_1,2_ = 8.3 Hz, H-1), 4.39–4.34 (m, 1H, CH_2All_), 4.30 (dd, 1H, *J*_6a,6b_ = 12.4 Hz, *J*_6a,5_ = 4.9 Hz, H-6a), 4.18 (dd, 1H, *J*_6b,5_ = 2.5 Hz, H-6b), 4.12–4.01 (m, 2H, CH_2All_, H-2), 3.75 (ddd, 1H, H-5), 2.11 (s, 3H, CH_3Ac_), 2.05 (s, 6H, CH_3Ac_). ^13^C{^1^H} NMR (100 MHz, CDCl_3_): δ 171.07, 170.65, 169.27 (CO_Ac_), 157.33 (CO_NH_, q, *J*_C,F_ = 37.4 Hz), 133.02 (CH_All_), 118.16 (CH_2All_), 115.57 (CF_3_, q, *J*_C,F_ = 286.5 Hz), 99.06 (C-1, ^1^*J*_C,H_ = 160.5 Hz), 71.98 (C-5), 71.76 (C-3), 70.12 (CH_2All_), 68.48 (C-4), 62.02 (C-6), 54.88 (C-2), 20.67, 20.53, 20.37 (3C, CH_3Ac_). HRMS (ESI^+^) *m*/*z*: [M + NH_4_]^+^ calcd for C_17_H_26_F_3_N_2_O_9_, 459.1590; found, 459.1586.

#### Allyl 3,4-Di-*O*-acetyl-2-deoxy-6-*O*-toluenesulfonyl-2-trifluoroacetamido-β-d-glucopyranoside (**26**)

NaOMe (25% in MeOH, 8.15 mL, 37.7 mmol, 0.3 equiv) was added to a solution of triacetate **25** (57.4 g, 130 mmol, 1.0 equiv) in MeOH (600 mL), and the mixture was stirred at rt for 1 h at which time a TLC follow-up (Tol/EtOAc 7:3) indicated reaction completion. A minimal amount of Dowex-H^+^ resin was added portionwise to reach neutral pH. The suspension was filtered, and the resin was thoroughly washed with MeOH. Volatiles were evaporated, and the crude material was dried under high vacuum before being dissolved in anhyd. pyridine (800 mL). Tosyl chloride (59.9 g, 314.5 mmol, 2.5 equiv) was added to the resulting solution at 0 °C. After 2.5 h at this temperature, acetic anhydride (59.4 mL, 629 mmol, 5.0 equiv) was added, and the reaction mixture was stirred for another 1 h at 0 °C and then for 2 h at rt. A follow-up by TLC (Tol/EtOAc 7:3) indicated the presence of a major product (*R*_*f*_ 0.5). MeOH (40 mL) was added, and after 30 min, volatiles were evaporated. The crude was dissolved in DCM (500 mL), and the organic layer was washed with 1 N aq HCl and satd aq NaHCO_3_, dried over Na_2_SO_4_, filtered, and concentrated. Flash chromatography (Tol/EtOAc 80:20 → 75:25) gave the desired **26** (64.0 g, 89% over three steps). The tosylate **26** had *R*_*f*_ 0.35 (Tol/EtOAc 4:1). ^1^H NMR (400 MHz, CDCl_3_): δ 7.80–7.78 (m, 2H, H_Ar_), 7.38–7.36 (m, 2H, H_Ar_), 7.04 (d, 1H, *J*_NH,2_ = 9.1 Hz, NH), 5.86–5.76 (m, 1H, CH_All_), 5.34 (dd, 1H, *J*_2,3_ = 10.6 Hz, *J*_3,4_ = 9.3 Hz, H-3), 5.27–5.22 (m, 1H, CH_2All_), 5.21–5.18 (m, 1H, CH_2All_), 4.96 (t, 1H, *J*_4,5_ = 9.8 Hz, H-4), 4.66 (d, 1H, *J*_1,2_ = 8.3 Hz, H-1), 4.30–4.24 (m, 1H, CH_2All_), 4.17 (dd, 1H, *J*_5,6a_ = 3.2 Hz, *J*_6a,6b_ = 11.2 Hz, H-6a), 4.12 (dd, 1H, *J*_5,6b_ = 5.8 Hz, H-6b), 4.06–3.99 (m, 2H, H-2, CH_2All_), 3.85 (ddd, 1H, H-5), 2.48 (s, 3H, CH_3Ts_), 2.03 (s, 6H, CH_3Ac_). ^13^C{^1^H} NMR (CDCl_3_): δ 171.20, 169.47 (2C, CO_Ac_), 157.42 (CO_NH_, q, *J*_C,F_ = 37.4 Hz), 145.29 (C_Ar_), 132.98 (CH_All_), 132.44, 129.95, 128.00 (C_Ar_), 118.00 (CH_2All_), 115.60 (CF_3_, q, *J*_C,F_ = 286.0 Hz), 98.91 (C-1, ^1^*J*_C,H_ = 161.5 Hz), 71.76 (2C, C-5, C-3), 69.96 (CH_2All_), 68.91 (C-4), 68.01 (C-6), 54.48 (C-2), 21.62 (CH_3Ts_), 20.51, 20.33 (2C, CH_3Ac_). HRMS (ESI^+^) *m*/*z*: [M + NH_4_]^+^ calcd for C_22_H_30_F_3_N_2_O_10_S, 571.1573; found, 571.1570.

#### Allyl 3,4-Di-*O*-acetyl-2-deoxy-6-iodo-2-trifluoroacetamido-β-d-glucopyranoside (**27**)

To a solution of tosylate **26** (64.0 g, 115.6 mmol, 1.0 equiv) in butanone (770 mL) was added sodium iodide (43.3 g, 289.2 mmol, 2.5 equiv). The reaction mixture was heated to reflux for 4 h. A TLC (Tol/EtOAc 7:3) follow-up indicated reaction completion with conversion of the starting material (*R*_*f*_ 0.5) into a less polar compound (*R*_*f*_ 0.6). The suspension was filtered over a pad of Celite and washed with Et_2_O, and the filtrate was concentrated to dryness. Flash chromatography (Tol/EtOAc 6:1 → 4:1) gave the desired **27** (52.0 g, 88%) as a white solid. The 6-iodo derivative had *R*_*f*_ 0.75 (Tol/EtOAc 1:1). ^1^H NMR (400 MHz, CDCl_3_): δ 6.61 (bd, 1H, *J*_NH,2_ = 7.0 Hz, NH), 5.93–5.83 (m, 1H, CH_All_), 5.36–5.30 (m, 2H, H-3, CH_2All_), 5.28–5.24 (m, 1H, CH_2All_), 4.93 (t, 1H, *J*_4,3_ = *J*_4,5_ = 9.4 Hz, H-4), 4.72 (d, 1H, *J*_1,2_ = 8.3 Hz, H-1), 4.44–4.39 (m, 1H, CH_2All_), 4.21–4.16 (m, 1H, CH_2All_), 4.09–4.02 (m, 1H, H-2), 3.63–3.57 (ddd, 1H, H-5), 3.33 (dd, 1H, *J*_5,6a_ = 2.7 Hz, H-6a), 3.20 (dd, 1H, *J*_5,6b_ = 8.8 Hz, *J*_6a,6b_ = 10.9 Hz, H-6b), 2.10, 2.06 (2s, 6H, CH_3Ac_). ^13^C{^1^H} NMR (100 MHz, CDCl_3_): δ 171.08, 169.39 (2C, CO_Ac_), 157.42 (CO_NH_, q, *J*_C,F_ = 37.3 Hz), 132.94 (CH_All_), 118.55 (CH_2All_), 115.55 (CF_3_, q, *J*_C,F_ = 285.9 Hz), 98.64 (C-1, ^1^*J*_C,H_ = 162.5 Hz), 73.88 (C-5), 72.22 (C-4), 71.39 (C-3), 70.08 (CH_2All_), 55.10 (C-2), 20.72, 20.37 (2C, CH_3Ac_), 2.64 (C-6). HRMS (ESI^+^) *m*/*z*: [M + NH_4_]^+^ calcd for C_15_H_23_F_3_IN_2_O_7_, 527.0502; found, 527.0510.

#### Allyl 2,6-Dideoxy-2-trifluoroacetamido-β-d-glucopyranoside (**28**)

Route a: Sodium methoxide (25% NaOMe in MeOH, 340 μL, 1.56 mmol, 0.2 equiv) was added to a solution of diacetate **19** (3.0 g, 7.83 mmol, 1.0 equiv) in MeOH (70 mL). The solution was stirred for 2 h at rt. More sodium methoxide (25% NaOMe in MeOH, 170 μL, 0.78 mmol, 0.1 equiv) was added, and after another 2 h, a TLC analysis (Tol/EtOAc 3:1) indicated reaction completion. Dowex-H^+^ resin was added portionwise under gentle stirring until neutral pH was reached. The suspension was filtered and washed with MeOH (3 × 10 mL), and the filtrate was concentrated in vacuo. The crude was purified by flash chromatography (cHex/EtOAc 40:60 → 10:90) to get the desired diol **28** (2.2 g, 7.35 mmol, 94%) as a white solid. Trifluoroacetamide **28** had *R*_*f*_ 0.25 (Tol/EtOAc 1:1). ^1^H NMR (400 MHz, CD_3_OD): δ 5.92–5.83 (m, 1H, CH_All_), 5.29–5.23 (m, 1H, CH_2All_), 5.16–5.13 (m, 1H, CH_2All_), 4.51 (d, 1H, *J*_1,2_ = 8.4 Hz, H-1), 4.32–4.27 (m, 1H, CH_2All_), 4.07–4.02 (m, 1H, CH_2All_), 3.73 (dd, 1H, *J*_2,3_ = 10.4 Hz, H-2), 3.51 (dd, 1H, *J*_3,4_ = 8.8 Hz, H-3), 3.37–3.30 (m, 1H, H-5), 3.07 (t, 1H, *J*_4,5_ = 8.8 Hz, H-4), 1.32 (d, 3H, *J*_5,6_ = 6.1 Hz, H-6). ^13^C{^1^H} NMR (100 MHz, CD_3_OD): δ 158.00 (CO_NH_, q, *J*_C,F_ = 36.5 Hz), 133.89 (CH_All_), 116.65 (CF_3_, q, *J*_C,F_ = 284.9 Hz), 114.79 (CH_2All_), 99.70 (C-1, ^1^*J*_C,H_ = 160.8 Hz), 76.13 (C-4), 73.44 (C-3), 72.00 (C-5), 69.43 (CH_2All_), 56.61 (C-2), 16.61 (C-6). HRMS (ESI^+^) *m*/*z*: [M + Na]^+^ calcd for C_11_H_16_F_3_NO_5_Na, 322.0878; found, 322.0874.

Route b: Et_3_B (1 M in hexane, 7.8 mL, 7.8 mmol, 0.2 equiv) was added dropwise to a solution of iodide **27** (20 g, 39.3 mmol, 1.0 equiv) and Bu_3_SnH (10.7 mL, 47.1 mmol, 1.2 equiv) in anhyd. DCM (390 mL) cooled to 0 °C. The solution was stirred for 1 h at 0 °C, at which time a follow-up by TLC (Tol/EtOAc 7:3) indicated the total consumption of the starting material (*R*_*f*_ 0.5) and the presence of a more polar product (*R*_*f*_ 0.4). The mixture was concentrated in vacuo. The crude was taken in MeOH (200 mL) and treated with sodium methoxide (25% in MeOH, 13.4 mL, 58.9 mmol, 1.5 equiv) to reach pH 9. After stirring at rt for 4 h, a TLC follow-up (Tol/EtOAc 7:3) indicated the presence of a single more polar product. A minimal amount of Dowex-H^+^ resin was added portionwise until neutral pH was reached. The suspension was filtered, and the resin was washed thoroughly with MeOH. Solvents were evaporated. Flash chromatography of the crude material (Tol/EtOAc, 55:45 → 20:80) gave the desired diol **28** (11.5 g, 97%) as a white solid.

Route c: The crude allyl 2,6-dideoxy-6-iodo-2-trifluoroacetamido-β-d-glucopyranoside (50 g, 117 mmol, 1.0 equiv, see the Supporting Information, Scheme S8) was dissolved in anhyd. THF (590 mL) and cooled to 0 °C. Bu_3_SnH (29.7 mL, 129 mmol, 1.1 equiv) was added, followed by the addition of Et_3_B (1.0 M in hexane, 11.7 mL, 11.7 mmol, 0.1 equiv). A careful follow-up by TLC (Tol/EtOAc 2:3) showed completion after stirring for 1 h at this temperature. MeOH (10 mL) was added, and after another 15 min, volatiles were evaporated, and the residue was dissolved in DCM (400 mL). The precipitate was filtered and washed with DCM (2 × 50 mL) to give the expected QuiN derivative **28** (31.0 g, 103 mmol, 88%). The filtrate was concentrated, and the residue was purified by chromatography eluting with (cHex/EtOAc 40:60 → 20:80) to give an additional amount (2.5 g, 8.3 mmol) of **28** as a white solid to reach an isolated yield of 95%. Analytical data for **28** were as described above.

#### Allyl 3-*O*-Acetyl-2,6-dideoxy-2-trifluoroacetamido-β-d-glucopyranoside (**29**)

Route a: A solution of diol **28** (11.5 g, 38.4 mmol, 1.0 equiv) in anhyd. THF (350 mL) was cooled to −78 °C. Anhyd. pyridine (6.2 mL, 76.9 mmol, 2.0 equiv) was added, followed by acetyl chloride (3.0 mL, 42.3 mmol, 1.1 equiv). The reaction mixture was allowed to reach rt over 8 h and stirred for another 16 h. A follow-up by TLC (Tol/EtOAc 7:3) indicated the conversion of the starting material into a less polar product (*R*_*f*_ 0.25). MeOH (3.0 mL) was added, and after stirring at rt for 30 min, solvents were evaporated. Flash chromatography (Tol/EtOAc 65:35 → 55:45) gave by order of elution diacetate **19** (600 mg, 1.56 mmol, 4.0%) as a white solid and the 3-*O*-acetyl derivative **29** (11.3 g, 32.2 mmol, 86%) as a white solid, albeit as a 20:1 mix with the 4-*O*-acetyl isomer.

Scale-Up: A solution of diol **28** (33.5 g, 112 mmol, 1.0 equiv) in anhyd. THF (560 mL) was cooled to −78 °C. Anhyd. pyridine (18.1 mL, 224 mmol, 2.0 equiv) was added, followed by the addition of acetyl chloride (8.7 mL, 123 mmol, 1.1 equiv). The reaction mixture was allowed to reach rt over 8 h and stirred overnight at this temperature. A follow-up by TLC (Tol/EtOAc 1:1) indicated conversion of the starting **28** into a less polar product (*R*_*f*_ 0.35). MeOH (15 mL) was added, and after stirring at rt for another 30 min, volatiles were evaporated. Flash chromatography using Tol/EtOAc (65:35 → 55:45) gave first the undesired diacetate **19** (3.6 g, 9.39 mmol, 8.3%) and then a non-separable >15:1 mix of the desired **29** and its regioisomer (30 g, 87.9 mol, 79%), both as a white solid. The target **29** had *R*_*f*_ 0.5 (Tol/EtOAc 1:1). ^1^H NMR (400 MHz, CDCl_3_): δ 6.93 (d, 1H, *J*_NH,2_ = 9.0 Hz, NH), 5.88–5.80 (m, 1H, CH_All_), 5.29–5.24 (m, 1H, CH_2All_), 5.22–5.16 (m, 2H, CH_2All_, H-3), 4.60 (d, 1H, *J*_1,2_ = 8.4 Hz, H-1), 4.37–4.32 (m, 1H, CH_2All_), 4.10–4.04 (m, 1H, CH_2All_), 4.02–3.95 (m, 1H, *J*_2,3_ = 10.5 Hz, H-2), 3.48–3.38 (m, 2H, H-4, H-5), 2.72 (d, 1H, *J*_4,OH_ = 6.0 Hz, OH-4), 2.11 (s, 3H, CH_3Ac_), 1.38 (d, 3H, *J*_5,6_ = 5.8 Hz, H-6). ^13^C{^1^H} NMR (100 MHz, CDCl_3_): δ 172.43 (CO_Ac_), 157.49 (CO_NH_, q, *J*_C,F_ = 37.3 Hz), 133.31 (CH_All_), 117.79 (CH_2All_), 115.68 (CF_3_, q, *J*_C,F_ = 286.1 Hz), 98.94 (C-1, ^1^*J*_C,H_ = 160.5 Hz), 75.13 (C-3), 74.21 (C-4), 72.02 (C-5), 69.95 (CH_2All_), 55.08 (C-2), 20.75 (C, CH_3Ac_), 17.51 (C-6). HRMS (ESI^+^) *m*/*z*: [M + Na]^+^ calcd for C_13_H_18_F_3_NO_6_Na, 364.0978; found, 364.0974.

Route b: DIPEA (20 μL, 114 μmol, 0.2 equiv) and acetic anhydride (54 μL, 574 μmol, 1.01 equiv) were added to diol **28** (170 mg, 568 μmol, 1.0 equiv) dissolved in dry MeCN (6.0 mL) and stirred vigorously for 3 h at 100 °C under an Ar atmosphere. The resulting mixture was allowed to reach rt, and volatiles were evaporated. Flash chromatography of the crude gave the 3-*O*-acetyl derivative **29** and its regioisomer as a non-separable 5:1 mixture (164 mg, 480 μmol, 84%). Analytical data were as described above.

#### Allyl 3-*O*-Benzoyl-2,6-dideoxy-2-trifluoroacetamido-β-d-glucopyranoside (**30**)

Dimethyltin dichloride (38 mg, 174 μmol, 0.2 equiv) was added to a solution of diol **28** (260 mg, 869 μmol, 1.0 equiv) in anhyd. THF containing pyridine (140 μL, 1.73 mmol, 2.0 equiv) at 0 °C. Benzoyl chloride (111 μL, 956 μmol, 1.1 equiv) was added dropwise, and the reaction mixture was stirred overnight while it slowly reached rt. At completion, MeOH was added, and after stirring for another 15 min, volatiles were removed under reduced pressure. Flash chromatography (Tol/EtOAc 75:25 → 70:30) gave the 3-*O*-benzoylated product **30** as a white solid (270 mg, 669 μmol, 77%). Compound **30** had *R*_*f*_ 0.4 (Tol/EtOAc 7:3). ^1^H NMR (400 MHz, CDCl_3_): δ 8.01–7.99 (m, 2H, H_Ar_), 7.63–7.60 (m, 1H, H_Ar_), 7.51–7.44 (m, 2H, H_Ar_), 7.14 (d, 1H, *J*_NH,2_ = 9.2 Hz, NH), 5.90–5.82 (m, 1H, CH_All_), 5.53 (dd, 1H, *J*_3,4_ = 8.4 Hz, *J*_2,3_ = 10.2 Hz, H-3), 5.30–5.25 (m, 1H, CH_2All_), 5.20–5.17 (m, 1H, CH_2All_), 4.69 (d, 1H, *J*_1,2_ = 8.3 Hz, H-1), 4.39–4.34 (m, 1H, CH_2All_), 4.24–4.17 (m, 1H, H-2), 4.11–4.06 (m, 1H, CH_2All_), 3.60–3.55 (m, 2H, H-4, H-5), 1.42 (d, 3H, *J*_5,6_ = 5.4 Hz, H-6). ^13^C{^1^H} NMR (100 MHz, CDCl_3_): δ 167.98 (CO_Bz_), 157.57 (CO_NH_, q, *J*_C,F_ = 37.3 Hz), 133.92 (C_Ar_), 133.35 (CH_All_), 129.90, 128.61 (4C, C_Ar_), 117.75 (CH_2All_), 115.63 (CF_3_, q, *J*_C,F_ = 286.4 Hz), 99.11 (C-1, ^1^*J*_C,H_ = 160.5 Hz), 75.98 (C-3), 74.58 (C-4), 72.10 (C-5), 70.00 (CH_2All_), 55.09 (C-2), 17.54 (C-6). HRMS (ESI^+^) *m*/*z*: [M + NH_4_]^+^ calcd for C_18_H_24_F_3_N_2_O_6_, 421.1581; found, 421.1572.

#### Allyl 3-*O*-*tert*-Butyldimethylsilyl-2,6-dideoxy-2-trifluoroacetamido-β-d-glucopyranoside (**31**)

2,6-Lutidine (138 μL, 1.44 mmol, 1.2 equiv) and TBSOTf (304 μL, 1.32 mmol, 1.1 equiv) were added to a solution of diol **28** (360 mg, 669 μmol, 1.0 equiv) in anhyd. THF (12 mL) at −78 °C. The reaction was allowed to attain rt slowly over 6 h. MeOH was added, and volatiles were evaporated. Flash chromatography (Tol/EtOAc 90:10 → 80:20) gave by order of elution the 4-*O*-silylated analogue (220 mg, 532 μmol, 44%) and the desired **30**, both as a white solid (240 mg, 580 μmol, 48%). The 3-*O*-silyl ether **31** had *R*_*f*_ 0.3 (Tol/EtOAc 4:1). ^1^H NMR (400 MHz, CDCl_3_): δ 6.39 (d, 1H, *J*_2,NH_ = 8.2 Hz, NH), 5.89–5.79 (m, 1H, CH_All_), 5.28–5.23 (m, 1H, CH_2All_), 5.21–5.18 (m, 1H, CH_2All_), 4.75 (d, 1H, *J*_1,2_ = 8.4 Hz, H-1), 4.36–4.31 (m, 1H, CH_2All_), 4.07–4.02 (m, 1H, CH_2All_), 3.92 (dd, 1H, *J*_3,4_ = 8.3 Hz, *J*_2,3_ = 10.0 Hz, H-3), 3.56–3.49 (m, 1H, H-2), 3.41 (dq, 1H, *J*_4,5_ = 9.4 Hz, H-5), 3.23–3.18 (m, 1H, H-4), 2.04 (d, 1H, *J*_4,OH_ = 3.4 Hz, OH), 1.36 (d, 3H, *J*_5,6_ = 6.2 Hz, H-6), 0.89 (s, 9H, C(CH_3_)_TBS_), 0.14 (s, 3H, CH_3,TBS_), 0.06 (s. 3H, CH_3,TBS_). ^13^C NMR (100 MHz, CDCl_3_): δ 157.27 (CO_NH_, q, *J*_C,F_ = 36.8 Hz), 133.50 (CH_2All_), 117.75 (CH_2All_), 115.65 (CF_3_, q, *J*_C,F_ = 287.0 Hz), 98.22 (C-1, ^1^*J*_C,H_ = 161 Hz), 77.29 (C-4), 74.37 (C-3), 71.38 (C-5), 70.02 (CH_2All_), 58.78 (C-2), 25.66 (CH_3,TBS_), 18.04 (C_q,TBS_), 17.66 (C-6), −4.03, −4.84 (2C, CH_3,TBS_). HRMS (ESI^+^) *m*/*z*: [M + H]^+^ calcd for C_17_H_30_F_3_NO_5_Si, 414.1924; found, 414.1934.

#### Allyl 4-*O*-Benzyl-2,6-dideoxy-2-trifluoroacetamido-β-d-glucopyranoside (**32**)

NaH (60% in mineral oil, 32 mg, 669 μmol, 1.0 equiv) was added portionwise to a solution of diol **28** (200 mg, 0.67 mmol, 1.0 equiv) and benzyl bromide (87 μL, 0.74 mmol, 1.1 equiv) in anhyd. DMF (5.0 mL) under vigorous stirring at 0 °C. After 3 h at 0 °C, 0.5 M aq NH_4_Cl (10 mL) was added, and the aqueous phase was extracted with EtOAc (20 mL). The organic phases were pooled and washed with brine, dried over Na_2_SO_4_, and concentrated. Flash chromatography (Tol/EtOAc 65:35 → 40:60) gave by order of elution the *N*,*N*-dibenzylated side product (12 mg, 31 μmol, 4.6%), the 4-*O*-benzyl ether **32** (110 mg, 282 μmol, 42%) as a white solid, and the unreacted **28** (95 mg, 317 μmol, 47%). The target **32** had *R*_*f*_ 0.55 (Tol/EtOAc 1:1). ^1^H NMR (400 MHz, CDCl_3_): δ 7.39–7.30 (m, 5H, H_Ar_), 6.75 (d, 1H, *J*_NH,2_ = 7.2 Hz, NH), 5.92–5.82 (m, 1H, CH_All_), 5.30–5.26 (m, 1H, CH_2All_), 5.24–5.21 (m, 1H, CH_2All_), 4.81 (d, 1H, *J* = 11.6 Hz, CH_2Bn_), 4.74 (d, 1H, CH_2Bn_), 4.64 (d, 1H, *J*_1,2_ = 8.1 Hz, H-1), 4.37–4.32 (m, 1H, CH_2All_), 4.09–3.98 (m, 2H, CH_2All_, H-3), 3.62–3.56 (m, 1H, H-2), 3.52–3.46 (dq, 1H, *J*_4,5_ = 9.0 Hz, H-5), 3.15–3.13 (m, 2H, H-4, OH), 1.37 (d, 3H, *J*_5,6_ = 6.2 Hz, H-6). ^13^C{^1^H} NMR (100 MHz, CDCl_3_): δ 157.76 (CO_NH_, q, *J*_C,F_ = 36.9 Hz), 138.01 (C_q,Ar_), 133.34 (CH_All_), 128.57, 128.04, 127.98 (C_Ar_), 118.06 (CH_2All_), 115.73 (CF_3_, q, *J*_C,F_ = 286.6 Hz), 98.43 (C-1, ^1^*J*_C,H_ = 161.4 Hz), 84.01 (C-4), 75.08 (CH_2Bn_), 73.13 (C-3), 71.45 (C-5), 69.96 (CH_2All_), 58.24 (C-2), 18.08 (C-6). HRMS (ESI^+^) *m*/*z*: [M + NH_4_]^+^ calcd for C_18_H_26_F_3_N_2_O_5_, 407.1788; found, 407.1778.

#### Allyl 3-*O*-Acetyl-2,6-dideoxy-2-trifluoroacetamido-β-d-galactopyranoside (**33**)

Pyridine (71 μL, 880 μmol, 1.5 equiv) and triflic anhydride (118 μL, 704 μmol, 1.2 equiv) were added to a solution of allyl glycoside **29** (200 mg, 586 μmol, 1.0 equiv) in anhyd. DCM (6 mL) at −10 °C. After stirring for 1 h at this temperature, DCM (10 mL) was added, and the solution was washed with 1 N aq HCl (10 mL), 0.5 M aq NaHCO_3_ (10 mL), and 50% aq NaCl (10 mL). The organic layer was dried over Na_2_SO_4_ and concentrated under reduced pressure, and the crude was extensively dried under high vacuum. TBANO_2_ (253 mg, 880 μmol, 1.5 equiv) was added to a solution of the crude material in anhyd. DMF (3.0 mL) at rt. After stirring overnight, a TLC analysis (Tol/EtOAc 7:3) indicated the absence of the triflate intermediate (*R*_*f*_ 0.8) and the presence of a polar spot (*R*_*f*_ 0.15). DCM (10 mL) was added, followed by water (40 mL). The DCM layer was separated, and the aqueous phase was extracted with DCM (2 × 10 mL). The organic layers were combined, washed with 50% aq NaCl (50 mL), separated, dried over Na_2_SO_4_, and concentrated under vacuum. Flash chromatography of the crude eluting with Tol/EtOAc (70:30 → 60:40) gave the expected **33** as a white solid (160 mg, 469 mmol, 80%). Alcohol **33** had *R*_*f*_ 0.25 (Tol/EtOAc 7:3). ^1^H NMR (400 MHz, CDCl_3_): δ 6.51 (d, 1H, *J*_NH,2_ = 8.7 Hz, NH), 5.89–5.81 (m, 1H, CH_All_), 5.30–5.25 (m, 1H, CH_2All_), 5.23–5.20 (m, 1H, CH_2All_), 5.16 (dd, 1H, *J*_3,4_ = 3.1 Hz, *J*_2,3_ = 11.2 Hz, H-3), 4.62 (d, 1H, *J*_1,2_ = 8.4 Hz, H-1), 4.40–4.34 (m, 1H, CH_2All_), 4.24–4.17 (m, 1H, H-2), 4.12–4.06 (m, 1H, CH_2All_), 3.86 (brs, 1H, H-4), 3.74 (dq, 1H, *J*_4,5_ = 0.8 Hz, H-5), 2.31 (d, 1H, *J*_4,OH_ = 5.1 Hz, OH), 2.12 (s, 3H, CH_3Ac_), 1.37 (d, 3H, *J*_5,6_ = 6.5 Hz, H-6). ^13^C{^1^H} NMR (100 MHz, CDCl_3_): δ 170.95 (C_Ac_), 157.51 (CO_NH_), 133.33 (CH_All_), 117.89 (CH_2All_), 114.22 (CF_3_), 99.38 (C-1, ^1^*J*_C,H_ = 161 Hz), 72.54 (C-3), 70.60 (C-5), 69.94 (CH_2All_), 69.60 (C-4), 51.71 (C-2), 20.66 (CH_3Ac_), 16.17 (C-6). HRMS (ESI^+^) *m*/*z*: [M + NH_4_]^+^ calcd for C_13_H_22_F_3_N_2_O_6_, 359.1424; found, 359.1417.

#### Allyl 3-*O*-Acetyl-4-azido-2,4,6-trideoxy-2-trifluoroacetamido-β-d-galactopyranoside (**34**)

Alcohol **29** (10.3 g, 30.1 mmol, 1.0 equiv), containing traces of the 4-*O*-acetyl isomer, was dissolved in anhyd. DCM (280 mL) under an Ar atmosphere. The solution was cooled to −10 °C. Triflic anhydride (6.5 mL, 39 mmol, 1.3 equiv) was added, followed by the addition of anhyd. pyridine (4.84 mL, 60 mmol, 2.0 equiv). The solution was stirred for 1 h at −10 °C. A follow-up by TLC (Tol/EtOAc 2:1) indicated the absence of the starting material (*R*_*f*_ 0.25) and the presence of a less polar product (*R*_*f*_ 0.8). The reaction mixture was diluted with DCM (100 mL). The organic layer was washed with 1 N aq HCl (200 mL) and 0.5 M aq NaHCO_3_ (200 mL), dried over Na_2_SO_4_, filtered, and concentrated in vacuo. The crude was dried under high vacuum for 2 h.

The residue was dissolved in anhyd. DMF (100 mL), and sodium azide (9.8 g, 150 mmol, 5.0 equiv) was added. After stirring at rt overnight, TLC (Tol/EtOAc 4:1) showed the total conversion of the intermediate triflate (*R*_*f*_ 0.45) and the presence of a more polar product (*R*_*f*_ 0.35). The reaction mixture was diluted with DCM (200 mL) and washed with H_2_O (300 mL) and brine (300 mL). The organic phase was dried over Na_2_SO_4_, filtered, and concentrated under reduced pressure. Flash chromatography (Tol/EtOAc 80:20 → 75:25) gave by order of elution the unwanted 3-azido allopyranoside (100 mg, 273 μmol, 1%) and the azido derivative **34** (9.1 g, 82%), both as a white solid.

Scale-Up: Pyridine (14.1 mL, 175 mmol, 2.0 equiv) was added to the contaminated **29** (30.0 g, 87.9 mmol, 1.0 equiv) in anhyd. DCM (550 mL). The solution was cooled to 0 °C, and triflic anhydride (17.7 mL, 105 mmol, 1.2 equiv) was added. After 2 h at 0 °C, DCM (200 mL) was added. The organic phase was washed with 1 N aq HCl (600 mL), 0.5 M aq NaHCO_3_ (600 mL), and brine (600 mL); then dried over Na_2_SO_4_; filtered; and concentrated. After extensive drying under high vacuum, the crude was dissolved in anhyd. DMF (250 mL). The solution was cooled to 0 °C, and sodium azide (28.5 g, 439 mmol, 5.0 equiv) was added. After stirring for 8 h while allowing the bath to reach rt, the mixture was concentrated, and the crude was diluted with DCM (700 mL) and water (700 mL). The organic layer was separated, dried over Na_2_SO_4_, and concentrated under reduced pressure. Flash chromatography (Tol/EtOAc 80:20 → 70:30) gave the desired AAT derivative **34** (28.2 g, 87.5%) obtained as a white solid. The targeted **34** had *R*_*f*_ 0.5 (Tol/EtOAc 7:3). ^1^H NMR (400 MHz, CDCl_3_): δ 6.41 (d, 1H, *J*_NH,2_ = 8.0 Hz, NH), 5.89–5.79 (m, 1H, CH_All_), 5.42 (dd, 1H, *J*_3,4_ = 3.6 Hz, *J*_2,3_ = 11.2 Hz, H-3), 5.29–5.23 (m, 1H, CH_2All_), 5.22–5.19 (m, 1H, CH_2All_), 4.68 (d, 1H, *J*_1,2_ = 8.4 Hz, H-1), 4.37–4.32 (m, 1H, CH_2All_), 4.10–4.04 (m, 2H, H-2, CH_2All_), 3.85 (dd, 1H, H-4), 3.62 (dq, 1H, *J*_4,5_ = 1.0 Hz, H-5), 2.14 (s, 3H, CH_3Ac_), 1.39 (d, 3H, *J*_5,6_ = 6.3 Hz, H-6). ^13^C{^1^H} NMR (100 MHz, CDCl_3_): δ 170.53 (C_Ac_), 157.35 (CO_NH_, q, *J*_C,F_ = 37.3 Hz), 133.24 (CH_All_), 118.04 (CH_2All_), 115.53 (CF_3_, q, *J*_C,F_ = 286.6 Hz), 98.70 (C-1, ^1^*J*_C,H_ = 160.4 Hz), 71.72 (C-3), 69.85 (CH_2All_), 69.30 (C-5), 63.27 (C-4), 52.34 (C-2), 20.31 (CH_3Ac_), 17.29 (C-6). HRMS (ESI^+^) *m*/*z*: [M + NH_4_]^+^ calcd for C_13_H_21_F_3_N_5_O_5_, 384.1489; found, 384.1481.

#### Allyl 4-Azido-2,4,6-trideoxy-2-trifluoroacetamido-β-d-galactopyranoside (**35**)

NaOMe (25% in MeOH, 273 μmol, 0.1 equiv) was added to a solution of azide **34** (1.0 g, 2.73 mmol, 1.0 equiv) in MeOH (25 mL). After stirring for 1 h at rt and a TLC follow-up (Tol/EtOAc 3:2), the reaction was quenched by adding Dowex-H^+^ resin under gentle stirring to reach pH ∼ 7.0. The suspension was filtered and thoroughly washed with MeOH. The combined filtrate was concentrated in vacuo. Flash chromatography (Tol/EtOAc 70:30 → 60:40) of the crude gave **35** as a white solid (810 mg, 2.49 mmol, 92%). Alcohol **35** had *R*_*f*_ 0.4 (Tol/EtOAc 3:2). ^1^H NMR (400 MHz, DMSO-*d*_6_): δ 9.17 (d, 1H, *J*_NH,2_ = 9.2 Hz, NH), 5.84–5.75 (m, 2H, CH_All_, OH), 5.22–5.16 (m, 1H, CH_2All_), 5.12–5.08 (m, 1H, CH_2All_), 4.38 (d, 1H, *J*_1,2_ = 8.4 Hz, H-1), 4.20–4.14 (m, 1H, CH_2All_), 3.97–3.92 (m, 2H, CH_2All_, H-3), 3.79 (dd, 1H, *J*_3,4_ = 3.6 Hz, *J*_4,5_ = 1.2 Hz, H-4), 3.76–3.69 (m, 1H, H-2), 3.64 (dq, 1H, H-5), 1.21 (d, 3H, *J*_5,6_ = 6.3 H, H-6). ^13^C{^1^H} NMR (100 MHz, DMSO-*d*_6_): δ 157.02 (CO_NH_, q, *J*_C,F_ = 36.2 Hz), 133.90 (CH_2All_), 116.47 (CF_3_, q, *J*_C,F_ = 286.8 Hz), 116.45 (CH_2All_), 100.16 (C-1, ^1^*J*_C,H_ = 160.5 Hz), 70.47 (C-3), 69.19 (CH_2All_), 68.92 (C-5), 66.02 (C-4), 53.37 (C-2), 17.76 (C-6). HRMS (ESI^+^) *m*/*z*: [M + NH_4_]^+^ calcd for C_11_H_19_F_3_N_5_O_4_, 342.1384; found, 342.1382.

#### Allyl 4-Azido-3-*O*-(2-naphthylmethyl)-2,4,6-trideoxy-2-trifluoroacetamido-β-d-galactopyranoside (**36**)

Route a: 2-Naphthylmethyl bromide (171 mg, 741 μmol, 1.2 equiv) was added to a solution of alcohol **35** (200 mg, 617 μmol, 1.0 equiv) in anhyd. DMF (6 mL) at 0 °C. NaH (60% in oil, 61 mg, 1.54 mmol, 2.5 equiv) was then added portionwise to the solution stirred vigorously at this temperature. The suspension was allowed to reach rt within 1 h and then heated at 40 °C for 2 h. At completion, 5% aq NH_4_Cl (15 mL) was added. The aqueous phase was extracted with DCM (3 × 10 mL). The combined DCM layers were washed with brine (20 mL), dried over Na_2_SO_4_, and concentrated. Flash chromatography (cHex/EtOAc 80:20 → 70:30) gave the desired **36** (280 mg, 603 μmol, 97%).

Route b: A solution of acetate **34** (2.0 g, 5.46 mmol, 1.0 equiv) in MeOH (40 mL) containing NaOMe (25% in MeOH, 132 μL, 617 μmol, 0.1 equiv) was stirred at rt for 1 h. TLC (Tol/EtOAc 2:1) indicated reaction completion, and Dowex-H^+^ resin was added under gentle stirring until neutral pH. The resin was filtered and washed thoroughly, and volatiles were evaporated and co-evaporated with toluene (2 × 10 mL). The crude product was dissolved in anhyd. DMF (20 mL) and cooled to 0 °C. 2-Naphthylmethyl bromide (1.51 g, 6.55 mmol, 1.2 equiv) was added, followed by the portionwise addition of NaH (60% in oil, 436 mg, 10.9 mmol, 2.0 equiv). After stirring for 3 h while the reaction mixture had reached rt, 5% aq NH_4_Cl (60 mL) was added, and the organics were extracted with DCM (3 × 40 mL). The combined DCM layers were washed with brine (100 mL), dried over Na_2_SO_4_, and concentrated. Flash chromatography (Tol/EtOAc 90:10 → 88:12) gave by order of elution the 2-napthylmethyl ether **36** (1.3 g, 2.80 mmol, 51%) obtained as a white solid and the intermediate alcohol **35** (600 mg, 1.63 mmol, 35%) as a white solid. The napthylmethyl ether **36** had *R*_*f*_ 0.6 (Tol/EtOAc 9:1). ^1^H NMR (400 MHz, CDCl_3_): δ 7.88–7.78 (m, 4H, H_Ar_), 7.54–7.51 (m, 2H, H_Ar_), 7.48–7.45 (m, 1H, H_Ar_), 6.48 (d, 1H, *J*_NH,2_ = 6.8 Hz, NH), 5.89–5.79 (m, 1H, CH_All_), 5.27–5.22 (m, 1H, CH_2All_), 5.21–5.18 (m, 1H, CH_2All_), 4.89 (d, 1H, *J*_1,2_ = 8.4 Hz, H-1), 4.85 (d, 1H, *J* = 11.6 Hz, CH_2Nap_), 4.73 (d, 1H, CH_2Nap_), 4.49 (dd, 1H, *J*_3,4_ = 3.6 Hz, *J*_2,3_ = 10.8 Hz, H-3), 4.35–4.29 (m, 1H, CH_2All_), 4.07–4.02 (m, 1H, CH_2All_), 3.79 (dd, 1H, *J*_4,5_ = 1.0 Hz, H-4), 3.68–3.58 (m, 2H, H-2, H-5), 1.38 (d, 3H, *J*_5,6_ = 6.3 Hz, H-6). ^13^C{^1^H} NMR (100 MHz, CDCl_3_): δ 157.46 (CO_NH_, q, *J*_C,F_ = 36.9 Hz), 134.16, 133.39, 133.28 (C_q,Ar_), 133.20 (CH_All_), 128.72, 127.96, 127.75, 127.43, 126.39. 126.36, 125.84 (C_Ar_), 118.15 (CH_2All_), 115.51 (CF_3_, q, *J*_C,F_ = 286.7 Hz), 97.55 (C-1, ^1^*J*_C,H_ = 164.0 Hz), 75.92 (C-3), 72.51 (CH_2Bn_), 70.03 (CH_2All_), 69.12 (C-5), 62.75 (C-4), 55.32 (C-2), 17.55 (C-6). HRMS (ESI^+^) *m*/*z*: [M + NH_4_^+^]^+^ calcd for C_22_H_27_F_3_N_5_O_4_, 482.2010; found, 482.2010.

#### Allyl 2-Amino-4-azido-3-*O*-(2-naphthylmethyl)-2,4,6-trideoxy-β-d-galactopyranoside (**37**)

KOH (241 mg, 4.30 mmol, 5.0 equiv) was added portionwise to a suspension of the 2-naphthylmethyl ether **36** (400 mg, 862 μmol, 1.0 equiv) in MeOH/H_2_O (1:1, 18 mL), and the solution was set into a 20 mL microwave vial. The reaction was set up in a Biotage Initiator+ instrument. After heating to 140 °C for 3 h, during which time the pressure reached 10 bar, a TLC analysis (EtOAc/MeCN 4:1) indicated the absence of the starting material and the presence of a more polar spot (*R*_*f*_ 0.5). Volatiles were eliminated, and DCM (50 mL) followed by water (50 mL) was added. The organic phase was separated, dried over Na_2_SO_4_, and concentrated to give the crude amine following extensive drying. Amine **37** had ^1^H NMR (400 MHz, CDCl_3_): δ 7.89–7.84 (m, 4H, H_Ar_), 7.56–7.49 (m, 3H, H_Ar_), 5.97–5.90 (m, 1H, CH_All_), 5.31–5.27 (m, 1H, CH_2All_), 5.21–5.18 (m, 1H, CH_2All_), 4.95 (d, 1H, *J* = 11.2 Hz, CH_2Nap_), 4.76 (d, 1H, *J* = 11.8 Hz, CH_2Nap_), 4.39–4.35 (m, 1H, CH_2All_), 4.21 (d, 1H, *J*_1,2_ = 7.9 Hz, H-1), 4.10–4.05 (m, 1H, CH_2All_), 3.72 (d, 1H, H-4), 3.62 (dd, 1H, *J*_3,4_ = 3.4 Hz, H-3), 3.59 (q, 1H, H-5), 3.21 (dd, 1H, *J*_2,3_ = 9.8 Hz, H-2), 2.39 (brs, 2H, NH_2_), 1.25 (d, 3H, *J*_5,6_ = 6.3 Hz, H-6). ^13^C{^1^H} NMR (100 MHz, CDCl_3_): δ 134.62 (C_q,Ar_), 133.99 (CH_All_), 133.25, 133.22 (C_q,Ar_), 128.61, 127.97, 127.75, 127.18, 126.31, 126.22, 125.88 (C_Ar_), 117.66 (CH_2All_), 102.84 (C-1), 82.14 (C-3), 72.25 (CH_2Nap_), 69.95 (CH_2All_), 69.30 (C-5), 61.40 (C-4), 52.59 (C-2), 17.69 (C-6). HRMS (ESI^+^) *m*/*z*: [M + H]^+^ calcd for C_20_H_24_N_4_O_3_, 369.1927; found, 369.1927.

#### Allyl 4-Azido-2-dichloroacetamido-3-*O*-(2-naphthylmethyl)-2,4,6-trideoxy-β-d-galactopyranoside (**38**)

The crude **37** was dissolved in anhyd. DCM (18 mL) containing pyridine (600 μL, 4.30 mmol, 5.0 equiv). Dichloroacetyl chloride (165 μL, 1.72 mmol, 2.0 equiv) was added to the solution stirred at 0 °C. After 2 h while the bath reached rt, a TLC (Tol/EtOAc 6:4) follow-up indicated reaction completion. MeOH (1.0 mL) was added, and after stirring for another 10 min, volatiles were evaporated. Flash chromatography of the crude eluting with Tol/EtOAc (90:10 → 85:15) gave dichloroacetamide **38** as a yellowish solid (340 mg, 711 μmol, 82%). Compound **38** had *R*_*f*_ 0.5 (Tol/EtOAc 4:1). ^1^H NMR (400 MHz, CDCl_3_): δ 7.87–7.81 (m, 4H, H_Ar_), 7.53–7.49 (m, 3H, H_Ar_), 6.72 (d, 1H, *J*_NH,2_ = 8.0 Hz, NH), 5.91–5.82 (m, 1H, CH_All_), 5.81 (s, 1H, CHCl_2_), 5.28–5.23 (m, 1H, CH_2All_), 5.19–5.16 (m, 1H, CH_2All_), 4.92 (d, 1H, *J*_1,2_ = 8.3 Hz, H-1), 4.85 (d, 1H, *J* = 11.8 Hz, CH_2Nap_), 4.78 (d, 1H, *J* = 11.8 Hz, CH_2Nap_), 4.54 (dd, 1H, *J*_3,4_ = 3.4 Hz, *J*_3,2_ = 10.6 Hz, H-3), 4.35–4.29 (m, 1H, CH_2All_), 4.08–4.03 (m, 1H, CH_2All_), 3.74 (dd, 1H, *J*_4,5_ = 1.2 Hz, H-4), 3.65 (dq, 1H, H-5), 3.61–3.54 (m, 1H, H-2), 1.34 (d, 3H, *J*_5,6_ = 6.4 Hz, H-6). ^13^C{^1^H} NMR (100 MHz, CDCl_3_): δ 164.47 (CO_NH_), 134.53 (C_q,Ar_), 133.70 (CH_All_), 133.21 (C_q,Ar_), 128.53, 127.96, 127.73, 127.36, 126.33, 126.27, 126.07 (C_Ar_), 117.91 (CH_2All_), 97.74 (C-1), 76.04 (C-3), 72.68 (CH_2Nap_), 70.14 (CH_2All_), 69.01 (C-5), 66.40 (CHCl_2_), 63.16 (C-4), 55.64 (C-2), 17.59 (C-6). HRMS (ESI^+^) *m*/*z*: [M + NH_4_]^+^ calcd for C_22_H_28_Cl_2_N_5_O_4_, 496.1518; found, 496.1514.

#### Allyl 2-Amino-4-azido-2,4,6-trideoxy-β-d-galactopyranoside (**39**)

LiOH·H_2_O (6.87 g, 163 mmol, 3.0 equiv) was added to a solution of azide **34** (20.0 g, 54.6 mmol, 1.0 equiv) in acetone/water (2:1, 320 mL). After stirring for 2 h at rt, a follow-up by TLC (Tol/EtOAc 1:1) indicated the total conversion of the starting **34** (*R*_*f*_ 0.7). Volatiles were evaporated, and the crude was purified by flash chromatography eluting with DCM/MeOH (20:1 → 12:1). The expected amine **39** (13.9 g, quant.), isolated as a yellowish oil, had *R*_*f*_ 0.15 (EtOAc/MeOH 9:1). ^1^H NMR (400 MHz, CDCl_3_): δ 5.97–5.87 (m, 1H, CH_All_), 5.32–5.28 (m, 1H, CH_2All_), 5.25–5.22 (m, 1H, CH_2All_), 4.61 (brs, 3H, OH, NH_2_), 4.38–4.34 (m, 1H, CH_2All_), 4.23 (d, 1H, *J*_1,2_ = 8.0 Hz, H-1), 4.09–4.04 (m, 1H, CH_2All_), 3.84 (dd, 1H, *J*_3,4_ = 3.6 Hz, H-3), 3.67–3.64 (m, 2H, H-4, H-5), 2.95 (dd, 1H, *J*_2,3_ = 10.2 Hz, H-2), 1.36 (d, 3H, *J*_5,6_ = 6.4 Hz, H-6). ^13^C{^1^H} NMR (100 MHz, CDCl_3_): δ 133.42 (CH_All_), 118.41 (CH_2All_), 101.31 (C-1), 72.49 (C-3), 70.18 (CH_2All_), 69.72 (C-5), 65.51 (C-4), 53.90 (C-2), 17.34 (C-6). HRMS (ESI^+^) *m*/*z*: [M + H]^+^ calcd for C_20_H_24_N_4_O_3_, 369.1927; found, 369.1926.

#### Allyl 4-Azido-2-trichloroacetamido-2,4,6-trideoxy-β-d-galactopyranoside (**4**)^45^

Amine **39** (13.9 g, 54.6 mmol, 1.0 equiv) in DCM (60 mL) was treated with triethylamine (22.8 mL, 163 mmol, 3.0 equiv) and trichloroacetyl chloride (12.1 mL, 109 mmol, 2.0 equiv) at rt. The reaction mixture was stirred for 30 min. A follow-up by TLC (Tol/EtOAc 7:3) indicated the presence of a major less polar product (*R*_*f*_ 0.45) and a minor one (*R*_*f*_ 0.7). DCM (50 mL) and water (50 mL) were added, and the biphasic mixture was stirred for another 1 h. The organic layer was separated, and the aqueous layer was extracted with DCM. The combined DCM layers were dried over Na_2_SO_4_, filtered, and concentrated under reduced pressure. Flash chromatography (Tol/EtOAc 80:20 → 70:30) gave the known **4** (17.5 g, 86%) as a white solid. Analytical data were as reported.^45^

#### Allyl 4-Azido-2-*N*-(*tert*-butyloxycarbonyl)-2,4,6-trideoxy-β-d-galactopyranoside (**40**)

DMAP (10.7 mg, 88 μmol, 0.1 equiv) and *tert*-butyl dicarbonate (Boc_2_O, 287 mg, 13.1 mmol, 1.5 equiv) were added to a solution of amine **39** (200 mg, 877 μmol, 1.0 equiv) in anhyd. THF (6.0 mL). After heating at 50 °C for 2 h, a follow-up by TLC (Tol/EtOAc 7:3) indicated completion. The reaction mixture was cooled to rt and concentrated under reduced pressure. The residue was purified by flash chromatography (cHex/EtOAc 70:30 → 60:40) to give by order of elution the 2-*N*,3-*O*-di-*tert*-butyloxycarbonyl side product (16 mg, 37 μmol, 4%) and the desired alcohol **40** (185 mg, 5.63 mmol, 83%), both as a white solid. Carbamate **40** had *R*_*f*_ 0.25 (Tol/EtOAc 7:3). ^1^H NMR (400 MHz, CDCl_3_): δ 5.98–5.88 (m, 1H, CH_All_), 5.33–5.28 (m, 1H, CH_2All_), 5.25–5.22 (m, 1H, CH_2All_), 5.00 (brs, 1H, OH), 4.80 (brs, 1H, NH), 4.39–4.33 (m, 2H, CH_2All_, H-1), 4.09–4.04 (m, 1H, CH_2All_), 4.00 (bd, 1H, *J*_2,3_ = 8.4 Hz, H-3), 3.68 (brd, 1H, *J*_3,4_ = 3.4 Hz, H-4), 3.64 (q, 1H, H-5), 3.56–3.50 (m, 1H, H-2), 1.47 (brs, 9H, CH_3tBu_), 1.37 (d, 3H, *J*_5,6_ = 6.2 Hz, H-6). ^13^C{^1^H} NMR (100 MHz, CDCl_3_): δ 157.77 (CO_NH_), 133.62 (CH_All_), 118.09 (CH_2All_), 99.47 (C-1), 81.05 (C_qtBu_), 74.40 (C-3), 69.57 (CH_2All_), 69.56 (C-5), 65.23 (C-4), 55.40 (C-2), 28.26 (CH_3tBu_), 17.48 (C-6). HRMS (ESI^+^) *m*/*z*: [M + Na]^+^ calcd for C_14_H_24_N_4_O_5_Na, 351.1638; found, 351.1636.

#### Allyl 4-Azido-2-*N*-carboxybenzyl-2,4,6-trideoxy-β-d-galactopyranoside (**41**)

Benzyl chloroformate (150 μL, 1.05 mmol, 1.2 equiv) was added to the DCM layer of a biphasic solution of amine **39** (200 mg, 877 μmol, 1.0 equiv) in DCM/satd aq NaHCO_3_ (2:1, 15 mL) at 0 °C. After stirring at this temperature for 2 h, a TLC (Tol/EtOAc 1:1) follow-up indicated completion. DCM (10 mL) and water (10 mL) were added. The DCM layer was separated, dried over Na_2_SO_4_, and concentrated. Flash chromatography (cHex/EtOAc 65:35 → 60:40) of the residue gave the *N*-protected derivative **41** (275 mg, 759 μmol, 86%) as a white solid. Azide **41** had *R*_*f*_ 0.4 (Tol/EtOAc 1:1). ^1^H NMR (400 MHz, DMSO-*d*_6_): δ 7.36–7.29 (m, 5H, H_Ar_), 7.11 (d, 1H, *J*_2,NH_ = 9.2 Hz, NH), 5.84–5.76 (m, 1H, CH_All_), 5.56 (d, 1H, *J*_3,OH_ = 4.4 Hz, OH), 5.25–5.19 (m, 1H, CH_2All_), 5.10–4.98 (m, 3H, CH_2Bn_, CH_2All_), 4.25 (d, 1H, *J*_1,2_ = 8.6 Hz, H-1), 4.20–4.14 (m, 1H, CH_2All_), 3.96–3.91 (m, 1H, CH_2All_), 3.80–3.77 (m, 1H, *J*_2,3_ = 8.8 Hz, H-3), 3.74 (brd, 1H, *J*_3,4_ = 3.2 Hz, H-4), 3.60 (q, 1H, H-5), 3.45–3.40 (m, 1H, H-2), 1.18 (d, 3H, *J*_5,6_ = 6.3 Hz, H-6). ^13^C{^1^H} NMR (100 MHz, DMSO-*d*_6_): δ 156.64 (CO), 137.81 (C_q,Ar_), 135.14 (CH_2All_), 128.72, 128.10, 128.04 (C_Ar_), 116.40 (CH_2All_), 101.26 (C-1), 71.27 (C-3), 69.08 (CH_2All_), 68.72 (C-5), 66.34 (C-4), 65.51 (CH_2Bn_), 54.37 (C-2), 17.86 (C-6). HRMS (ESI^+^) *m*/*z*: [M + NH_4_]^+^ calcd for C_17_H_26_N_5_O_5_, 380.1928; found, 380.1924.

#### Allyl 4-Azido-2-tetrachlorophthalimido-2,4,6-trideoxy-β-d-galactopyranoside (**42**)

Tetrachlorophthalic anhydride (TCPO, 150 mg, 525 μmol, 0.6 equiv) was added to a solution of amine **39** (200 mg, 877 μmol, 1.0 equiv) in anhyd. DCE (6.0 mL). After stirring for 30 min at rt under an Ar atmosphere, Et_3_N (146 μL, 1.05 mmol, 1.2 equiv) was added, followed by more TCPO (150 mg, 525 μmol, 0.6 equiv). After 1 h, a TLC (EtOAc/MeOH 9:1) follow-up indicated the absence of the starting amine. Volatiles were evaporated, and the residue was dried under high vacuum. The crude was dissolved in anhyd. pyridine (60 mL), and more TCPO (50 mg, 175 μmol, 0.2 equiv) was added. After heating at 50 °C for 1 h, a TLC (cHex/EtOAc 3:2) follow-up indicated completion. Volatiles were evaporated and co-evaporated with toluene (2 × 5 mL). A solution of the crude in DCM (20 mL) was washed with water (30 mL) and brine (30 mL), dried over Na_2_SO_4_, filtered, and concentrated under reduced pressure. Flash chromatography (cHex/EtOAc 75:25 → 70:30) of the residue gave the tetrachlorophthalimide **42** (110 mg, 223 μmol, 25%) as a white solid. The target **42** had *R*_*f*_ 0.65 (Tol/EtOAc 7:3). ^1^H NMR (400 MHz, CDCl_3_): δ 5.80–5.70 (m, 1H, CH_All_), 5.19–5.14 (m, 2H, CH_2All_, H-1), 5.12–5.08 (m, 1H, CH_2All_), 5.50 (brs, 1H, H-3), 4.31–4.26 (m, 1H, CH_2All_), 4.23 (dd, 1H, *J*_1,2_ = 8.4 Hz, *J*_2,3_ = 11.0 Hz, H-2), 4.06–4.00 (m, 1H, CH_2All_), 3.88 (dq, 1H, H-5), 3.80 (dd, 1H, *J*_3,4_ = 3.8 Hz, *J*_4,5_ = 0.9 Hz, H-4), 2.40 (d, 1H, *J*_3,OH_ = 8.0 Hz, OH), 1.48 (d, 3H, *J*_5,6_ = 6.4 Hz, H-6). ^13^C{^1^H} NMR (100 MHz, CDCl_3_): δ 163.47 (CO), 140.36 (C_Ar_), 133.51 (CH_All_), 127.22 (C_Ar_), 117.68 (CH_2All_), 96.87 (C-1), 70.25 (C-5), 69.68 (CH_2All_), 68.37 (C-3), 66.99 (C-4), 55.20 (C-2), 17.43 (C-6). HRMS (ESI^+^) *m*/*z*: [M + NH_4_]^+^ calcd for C_17_H_18_Cl_4_N_5_O_5_, 512.0062; found, 512.0071.

#### Allyl 2,4-Diazido-2,4,6-trideoxy-β-d-galactopyranoside (**43**)

K_2_CO_3_ (242 mg, 1.75 mmol, 2.0 equiv) and *N*-diazoimidazole-1-sulfonamide; sulfuric acid (356 mg, 1.31 mmol, 1.5 equiv) were added to a solution of the amine **39** (200 mg, 877 μmol, 1.0 equiv) in MeOH (10 mL). After stirring overnight at rt, a TLC (Tol/EtOAc 6:4) follow-up indicated reaction completion. Volatiles were evaporated, and the residue in DCM (20 mL) was washed with water (20 mL) and brine (20 mL), dried over Na_2_SO_4_, filtered, and concentrated. Flash chromatography (Tol/EtOAc 75:25 → 70:30) of the residue gave the diazide **43** (155 mg, 610 μmol, 76%) as a white solid. The galactoside analogue **43** had *R*_*f*_ 0.65 (Tol/EtOAc 6:4). ^1^H NMR (400 MHz, CDCl_3_): δ 6.00–5.90 (m, 1H, CH_All_), 5.37–5.31 (m, 1H, CH_2All_), 5.25–5.22 (m, 1H, CH_2All_), 4.44–4.39 (m, 1H, CH_2All_), 4.30 (d, 1H, *J*_1,2_ = 7.6 Hz, H-1), 4.15–4.10 (m, 1H, CH_2All_), 3.68 (dd, 1H, *J*_3,4_ = 3.6 Hz, *J*_4,5_ = 1.0 Hz, H-4), 3.66–3.61 (m, 2H, H-3, H-5), 3.56 (dd, 1H, *J*_2,3_ = 10.0 Hz, H-2), 2.53 (d, 1H, *J*_3,OH_ = 4.4 Hz, OH-3), 1.38 (d, 3H, *J*_5,6_ = 6.4 Hz, H-6). ^13^C{^1^H} NMR (100 MHz, CDCl_3_): δ 133.39 (CH_All_), 117.72 (CH_2All_), 101.06 (C-1), 72.65 (C-3), 70.17 (CH_2All_), 69.67 (C-5), 64.76 (C-4), 63.97 (C-2), 17.36 (C-6). HRMS (ESI^+^) *m*/*z*: [M + H]^+^ calcd for C_9_H_15_N_6_O_3_, 255.1200; found, 255.1199.

#### Allyl 2-Amino-2,6-dideoxy-β-d-glucopyranoside (**44**)

LiOH·H_2_O (1.32 g, 3.14 mmol, 2.0 equiv) was added to a solution of diacetate **19** (6.0 g, 1.57 mmol, 1.0 equiv) in acetone/water (2:1, 15 mL) at rt. After stirring for 3 h at rt, the mixture was concentrated under reduced pressure. The crude was passed through a short silica gel column eluting with DCM/MeOH (90:10 → 85:15) to give the expected amine **44** (3.0 g, 1.47 mmol, 94%) as a yellowish oil. The amine **44** had *R*_*f*_ 0.2 (DCM/MeOH 9:1). ^1^H NMR (400 MHz, CDCl_3_): δ 6.06 (brs, 4H, OH, NH_2_), 5.97–5.87 (m, 1H, CH_All_), 5.32–5.28 (m, 1H, CH_2All_), 5.25–5.22 (m, 1H, CH_2All_), 4.44 (d, 1H, *J*_1,2_ = 8.0 Hz, H-1), 4.37–4.32 (m, 1H, CH_2All_), 4.11–4.06 (m, 1H, CH_2All_), 3.66–3.61 (m, 1H, H-3), 3.38–3.35 (m, 1H, H-5), 3.22–3.18 (m, 1H, H-4), 2.94–2.89 (m, 1H, H-2), 1.32 (d, 3H, *J*_5,6_ = 6.4 Hz, H-6). ^13^C{^1^H} NMR (100 MHz, CDCl_3_): δ 133.19 (CH_All_), 118.84 (CH_2All_), 99.11 (C-1), 75.69 (C-4), 73.56 (C-3), 72.24 (C-5), 70.39 (CH_2All_), 56.86 (C-2), 17.33 (C-6). HRMS (ESI^+^) *m*/*z*: [M + H]^+^ calcd for C_9_H_18_NO_4_, 204.1230; found, 204.1226.

#### Allyl 2-Azido-2,6-dideoxy-β-d-glucopyranoside (**45**)

K_2_CO_3_ (2.0 g, 14.7 mmol, 1.5 equiv) and imidazole-1-sulfonyl azide and sulfuric acid (2.4 g, 1.18 mmol, 1.2 equiv) were added to a solution of the amine **44** (2.0 g, 9.84 mmol, 1.0 equiv) in MeOH (30 mL) at 0 °C. The reaction was run overnight at rt. A TLC follow-up (Tol/EtOAc 6:4) revealed the presence of a major product (*R*_*f*_ 0.3). The reaction mixture was concentrated. The residue was dissolved in DCM (30 mL), and the organic phase was washed with water. The organic layer was separated, dried over Na_2_SO_4_, filtered, and concentrated. Flash chromatography (Tol/EtOAc 70:30 → 60:40) of the residue gave the expected azide **45** (1.6 g, 6.98 mmol, 71%) as a yellowish oil. Azide **45** had *R*_*f*_ 0.3 (Tol/EtOAc 1:1). ^1^H NMR (400 MHz, CDCl_3_): δ 6.01–5.92 (m, 1H, CH_All_), 5.38–5.34 (m, 1H, CH_2All_), 5.26–5.24 (m, 1H, CH_2All_), 4.44–4.37 (m, 2H, CH_2All_, H-1), 4.18–4.13 (m, 1H, CH_2All_), 3.49 (brs, 1H, OH), 3.37–3.30 (m, 3H, H-2, H-4, H-5), 3.25–3.23 (m, 2H, H-3, OH), 1.35 (d, 3H, *J*_5,6_ = 6.0 Hz, H-6). ^13^C{^1^H} NMR (100 MHz, CDCl_3_): δ 133.37 (CH_All_), 117.86 (CH_2All_), 101.82 (C-1), 75.33 (C-3), 74.93 (C-5), 71.67 (C-4), 70.36 (CH_2All_), 66.21 (C-2), 17.48 (C-6). HRMS (ESI^+^) *m*/*z*: [M + H]^+^ calcd for C_9_H_16_N_3_O_4_, 230.1136; found, 230.1136.

#### Allyl 2-Dichloroacetamido-2,6-dideoxy-β-d-glucopyranoside (**46**)

Route a: 25% Methanolic NaOMe (0.76 μL, 0.35 mmol, 0.2 equiv) was added to a solution of diacetate **7** (700 mg, 1.76 mmol, 1.0 equiv) in MeOH (9 mL). After stirring at rt for 1 h, Dowex-H^+^ resin was added slowly until neutral pH is reached. The suspension was filtered and washed thoroughly with MeOH. Volatiles were evaporated, and the crude was purified by passing through a short pack of silica gel eluting with cHex/EtOAc (20:80 → 10:90). The desired diol **46** (500 mg, 1.25 mmol, 90%) was isolated as a white solid.

Route b: Triethylamine (1.3 mL, 9.84 mmol, 5.0 equiv) and dichloroacetic anhydride (0.9 mL, 5.90 mmol, 3.0 equiv) were added to a solution of amine **44** (400 mg, 1.96 mmol, 1.0 equiv) in EtOAc (10 mL) at 0 °C. The reaction mixture was stirred for 2 h while reaching rt. Following a TLC analysis (EtOAc/MeCN 4:1), water (5 mL) was added, and after another 3 h at rt, a TLC analysis (EtOAc) showed the presence of one major spot. Volatiles were evaporated, and the crude was purified by column chromatography (cHex/EtOAc, 10:90 → 0:100) to give the target **46** as a white solid (500 mg, 1.59 mmol, 81%). Dichloroacetamide **46** had *R*_*f*_ 0.4 (EtOAc/MeCN, 4:1). ^1^H NMR (400 MHz, DMSO-*d*_6_): δ 8.41 (d, 1H, *J*_NH,2_ = 8.8 Hz, NH), 6.37 (s, 1H, CHCl_2_), 5.83–5.75 (m, 1H, CH_All_), 5.25–5.19 (m, 1H, CH_2All_), 5.10–5.07 (m, 2H, CH_2All_, OH-4), 5.00 (d, 1H, *J*_OH,3_ = 6.0 Hz, OH-3), 4.37 (d, 1H, *J*_1,2_ = 8.4 Hz, H-1), 4.19–4.14 (m, 1H, CH_2All_), 3.97–3.92 (m, 1H, CH_2All_), 4.08 (dt, 1H, H-2), 3.35–3.30 (ddd, 1H, *J*_2,3_ = 10.2 Hz, H-3), 3.21–3.17 (dq, 1H, *J*_4,5_ = 9.3 Hz, H-5), 2.85 (dt, 1H, *J*_3,4_ = 9.0 Hz, H-4), 1.18 (d, 3H, *J*_5,6_ = 6.2 Hz, H-6). ^13^C{^1^H} NMR (100 MHz, DMSO-*d*_6_): δ 163.34 (CO_NH_), 134.52 (CH_All_), 116.12 (CH_2All_), 99.92 (C-1), 75.89 (C-4), 73.20 (C-3), 71.67 (C-5), 68.76 (CH_2All_), 67.19 (CHCl_2_), 56.38 (C-2), 17.83 (C-6). HRMS (ESI^+^) *m*/*z*: [M + Na]^+^ calcd for C_11_H_17_Cl_2_NO_5_Na, *m*/*z* 336.0381; found, 336.0388.

#### Allyl 2,6-Dideoxy-2-trichloroacetamido-β-d-glucopyranoside **(47**)^45^

The amine **44** (80 mg, 394 μmol, 1.0 equiv) was dissolved in DCM (5 mL). The solution was cooled to 0 °C, and Et_3_N (326 μL, 2.36 μmol, 6.0 equiv) was added, followed by the dropwise addition of trichloroacetyl chloride (131 μL, 1.18 mmol, 3.0 equiv). After stirring for 2 h at 0 °C, a TLC analysis (Tol/EtOAc, 5:1) showed complete conversion. DCM (5 mL) and water (10 mL) were added at rt and stirring went on for 2 h. At this time, a TLC analysis (Tol/EtOAc, 1:4) revealed the presence of one major spot (*R*_*f*_ 0.6). The DCM layer was collected, and the aq layer was extracted with DCM (285 mL). The DCM parts were combined, dried over Na_2_SO_4_, and concentrated in vacuo. Flash chromatography (Tol/EtOAc, 40:60 → 20:80) of the crude gave **47** as a white solid (101 mg, 85%). Analytical data were as published.^45^

#### Allyl 2-*N*-Carboxybenzyl-2,6-dideoxy-β-d-glucopyranoside (**48**)

NaHCO_3_ (414 mg, 1.96 mmol, 2.0 equiv) in water (2 mL) was added to a solution of the amine **44** (200 mg, 985 mmol, 1.0 equiv) in EtOAc (6.0 mL) at 0 °C. Benzyl chloroformate (154 μL, 1.08 mmol, 1.1 equiv) was added dropwise. After stirring for 1 h at 0 °C, MeOH (200 μL) was added. After stirring for another 5 min at rt, EtOAc (10 mL) was added, and the organic phase was washed with brine (20 mL), dried over Na_2_SO_4_, and concentrated. Flash chromatography (Tol/EtOAc 20:80 → 10:90) gave the expected carbamate **48** (240 mg, 711 μmol, 81%) as a white solid. The diol **48** had *R*_*f*_ 0.15 (Tol/EtOAc 1:4). ^1^H NMR (400 MHz, DMSO-*d*_6_): δ 7.38–7.29 (m, 5H, H_Ar_), 7.14 (d, 1H, *J*_2,NH_ = 7.2 Hz, NH), 5.87–5.77 (m, 1H, CH_All_), 5.26–5.20 (m, 1H, CH_2All_), 5.10–4.94 (m, 5H, CH_2All_, CH_2Cbz_, OH-4, OH-3), 4.28 (d, 1H, *J*_1,2_ = 7.2 Hz, H-1), 4.21–4.17 (m, 1H, CH_2All_), 3.98–3.93 (m, 1H, CH_2All_), 3.31–3.20 (m, 2H, H-2, H-3), 3.17–3.10 (dq, 1H, *J*_4,5_ = 9.3 Hz, H-5), 2.87–2.83 (m, 1H, H-4), 1.17 (d, 3H, *J*_5,6_ = 6.1 Hz, H-6). ^13^C{^1^H} NMR (100 MHz, DMSO-*d*_6_): δ 156.55 (CO_NH_), 137.87 (C_q,Ar_), 135.22 (CH_All_), 128.71, 128.02 (C_Ar_), 116.35 (CH_2All_), 101.16 (C-1, ^1^*J*_C,H_ = 163.0 Hz), 76.44 (C-4), 74.18 (C-3), 72.08 (C-5), 69.11 (CH_2All_), 65.43 (CH_2,Cbz_), 57.98 (C-2), 18.40 (C-6). HRMS (ESI^+^) *m*/*z*: [M + Na]^+^ calcd for C_17_H_23_NO_6_Na, 360.1418; found, 360.1403.

#### Allyl 3-*O*-Acetyl-2-dichloroacetamido-2,6-dideoxy-β-d-glucopyranoside (**49**)

A solution of diol **46** (400 mg, 1.27 mmol, 1.0 equiv) in anhyd. THF (30 mL) was cooled to −78 °C. Anhyd. pyridine (206 μL, 2.55 mmol, 2.0 equiv) was added, followed by the addition of acetyl chloride (95 μL, 1.34 mmol, 1.05 equiv). The reaction mixture slowly reached rt over 8 h and was stirred overnight at this temperature. A follow-up by TLC (cHex/EtOAc 3:7) indicated the conversion of the starting **46** and the presence of less polar products. MeOH (0.1 mL) was added, and after stirring at rt for 10 min, solvents were evaporated. The crude was purified by flash chromatography using cHex/EtOAc (60:40 → 50:50). The 3-*O*-acetyl derivative **49** (292 mg, 822 μmol, 64%), isolated as a white solid, had R_*f*_ 0.4 (cHex/EtOAc 1:1). ^1^H NMR (400 MHz, DMSO-*d*_6_): δ 8.62 (d, 1H, *J*_NH,2_ = 9.2 Hz, NH), 6.36 (s, 1H, CHCl_2_), 5.86–5.77 (m, 1H, CH_All_), 5.40 (d, 1H, *J*_OH,4_ = 6.0 Hz, OH-4), 5.23–5.18 (m, 1H, CH_2All_), 5.11–5.08 (m, 1H, CH_2All_), 4.88 (dd, 1H, *J*_3,4_ = 9.0 Hz, *J*_3,2_ = 10.6 Hz, H-3), 4.56 (d, 1H, *J*_1,2_ = 8.4 Hz, H-1), 4.21–4.16 (m, 1H, CH_2All_), 4.01–3.96 (m, 1H, CH_2All_), 3.71–3.64 (m, 1H, H-2), 3.38–3.31 (dq, 1H, *J*_4,5_ = 9.3 Hz, H-5), 3.13–3.07 (m, 1H, H-4), 1.94 (s, 3H, CH_3Ac_), 1.21 (d, 3H, *J*_5,6_ = 6.0 Hz, H-6). ^13^C NMR (100 MHz, DMSO-*d*_6_): δ 170.10 (CO_Ac_), 164.13 (CO_NH_), 134.84 (CH_All_), 116.77 (CH_2All_), 99.71 (C-1, ^1^*J*_C,H_ = 162 Hz), 75.36 (C-3), 73.55 (C-4), 72.05 (C-5), 69.40 (CH_2All_), 67.24 (CHCl_2_), 54.66 (C-2), 21.19 (CH_3Ac_), 18.11 (C-6). HRMS (ESI^+^) *m*/*z*: [M + Na]^+^ calcd for C_13_H_19_Cl_2_NO_6_Na, 378.0487; found, 378.0492.

#### Allyl 3-*O*-Acetyl-2-azido-2,6-dideoxy-β-d-glucopyranoside (**51**)

Acetyl chloride (102 μL, 14.4 mmol, 1.1 equiv) was added dropwise to a solution of the diol **45** (300 mg, 1.30 mmol, 1.0 equiv) and pyridine (210 μL, 26.1 mmol, 2.0 equiv) in anhyd. THF (15 mL) at −78 °C. The reaction mixture slowly reached rt. After 16 h, a TLC analysis (Tol/EtOAc 4:1) indicated reaction completion. MeOH (0.1 mL) was added, and after 10 min, volatiles were evaporated. Flash chromatography (Tol/EtOAc 84:16 → 80:20) gave the 3-*O*-acetylated product **51** (240 mg, 0.88 mmol, 67%) along with the regioisomer (65 mg, 0.23 mmol, 18%). The target **51** had *R*_*f*_ 0.55 (Tol/EtOAc 4:1). ^1^H NMR (400 MHz, CDCl_3_): δ 5.99–5.91 (m, 1H, CH_All_), 5.38–5.33 (m, 1H, CH_2All_), 5.26–5.23 (m, 1H, CH_2All_), 4.74 (dd, 1H, *J*_3,4_ = 9.2 Hz, H-3), 4.43–4.38 (m, 2H, CH_2All_, H-1), 4.19–4.14 (m, 1H, CH_2All_), 3.46 (dd, 1H, *J*_1,2_ = 8.1 Hz, *J*_3,2_ = 10.1 Hz, H-2), 3.41–3.35 (dq, 1H, *J*_4,5_ = 9.4 Hz, H-5), 3.34–3.26 (m, 1H, H-4), 2.62 (d, 1H, *J*_4,OH_ = 5.6 Hz, OH), 2.18 (s, 3H, CH_3Ac_), 1.36 (d, 3H, *J*_5,6_ = 6.0 Hz, H-6). ^13^C{^1^H} NMR (100 MHz, CDCl_3_): δ 172.04 (CO_Ac_), 133.29 (CH_All_), 117.97 (CH_2All_), 100.56 (C-1), 76.33 (C-3), 74.60 (C-4), 72.21 (C-5), 70.39 (CH_2All_), 63.93 (C-2), 20.94 (CH_3Ac_), 17.42 (C-6). HRMS (ESI^+^) *m*/*z*: [M + Na]^+^ calcd for C_11_H_17_N_3_O_5_Na, 294.1060; found, 294.1055.

#### Allyl 3-*O*-Acetyl-4-azido-2-dichloroacetamido-2,4,6-trideoxy-β-d-galactopyranoside (**52**)

Alcohol **49** (280 mg, 789 μmol, 1.0 equiv) was dissolved in anhyd. DCM (7.0 mL) under an Ar atmosphere. The solution was cooled to −10 °C. Anhyd. pyridine (113 μL, 1.41 mmol, 2.0 equiv) was added, followed by addition of triflic anhydride (177 μL, 105 mmol, 1.5 equiv). After stirring for 1 h at −10 °C, a follow-up by TLC (Tol/EtOAc 3:1) revealed the absence of the starting **49** (*R*_*f*_ 0.2) and the presence of a less polar product (R_*f*_ 0.8). The reaction mixture was diluted with DCM (10 mL). The organic layer was washed with 1 N aq HCl (20 mL) and NaHCO_3_ (20 mL), dried over Na_2_SO_4_, filtered, and concentrated in vacuo. The crude was dried under high vacuum for 2 h. The residue was dissolved in anhyd. DMF (7 mL), and sodium azide (229 mg, 3.52 mmol, 5.0 equiv) was added. After overnight stirring, TLC (Tol/EtOAc 2:1) revealed the absence of the intermediate triflate (*R*_*f*_ 0.85) and the presence of a less polar product (*R*_*f*_ 0.65). The reaction mixture was diluted with DCM (20 mL) and washed with water (70 mL) and brine (70 mL). The organic phase was dried over Na_2_SO_4_, filtered, and concentrated under reduced pressure. Flash chromatography (Tol/EtOAc 80:20 → 70:30) gave the azido derivative **52** (205 mg, 539 μmol, 68%) as a white solid. The azide **52** had *R*_*f*_ 0.35 (Tol/EtOAc 4:1). ^1^H NMR (400 MHz, CDCl_3_): δ 6.51 (d, 1H, *J*_NH,2_ = 8.0 Hz, NH), 5.89 (s, 1H, CHCl_2_), 5.91–5.81 (m, 1H, CH_All_), 5.49 (dd, 1H, *J*_3,4_ = 4.0 Hz, *J*_3,2_ = 11.2 Hz, H-3), 5.30–5.24 (m, 1H, CH_2All_), 5.21–5.17 (m, 1H, CH_2All_), 4.72 (d, 1H, *J*_1,2_ = 8.4 Hz, H-1), 4.37–4.32 (m, 1H, CH_2All_), 4.11–4.05 (m, 1H, CH_2All_), 4.02 (dt, 1H, H-2), 3.85 (dd, 2H, *J*_4,5_ = 1.2 Hz, H-4), 3.80 (dq, 1H, H-5), 2.14 (s, 3H, CH_3Ac_), 1.41 (d, 3H, *J*_5,6_ = 6.4 Hz, H-6). ^13^C{^1^H} NMR (100 MHz, CDCl_3_): δ 170.42 (CO_Ac_), 164.47 (CO_NH_), 133.52 (CH_All_), 117.91 (CH_2All_), 98.91 (C-1), 71.62 (C-3), 69.91 (CH_2All_), 69.24 (C-5), 66.31 (CHCl_2_), 63.44 (C-4), 52.45 (C-2), 20.44 (CH_3Ac_), 17.34 (C-6). HRMS (ESI^+^) *m*/*z*: [M + Na]^+^ calcd for C_13_H_18_Cl_2_N_4_O_5_Na, 403.0552; found, 403.0552.

#### Allyl 3-*O*-Acetyl-2,6-dideoxy-2-trichloroacetamido-β-d-galactopyranoside (**54**)

A solution of alcohol **50** (1.1 g, 2.82 mmol, 1.0 equiv) in anhyd. DCM (15 mL) was cooled to −10 °C. Pyridine (454 μL, 5.65 mmol, 2.0 equiv) and triflic anhydride (569 μL, 3.39 mmol, 1.2 equiv) were added. The mixture was stirred for 1 h at this temperature and then diluted with DCM (20 mL). The organic phase was washed with 1 N aq HCl (40 mL), satd aq NaHCO_3_ (40 mL), and brine (40 mL); passed through a phase separator filter; and concentrated to dryness. The crude was dissolved in anhyd. DMF (8.0 mL) and cooled to 0 °C. TBANO_2_ (2.0 g, 7.06 mmol, 2.5 equiv) was added, and the reaction mixture was stirred for 12 h after having reached rt slowly. A TLC analysis (Tol/EtOAc 7:3) showed the absence of the intermediate triflate (*R*_*f*_ 0.7) and the presence of a polar spot (*R*_*f*_ 0.1). The mixture was diluted with DCM (30 mL) and washed with water (100 mL) and brine (60 mL). The organic layer was separated, dried over Na_2_SO_4_, and concentrated under reduced pressure. Flash chromatography (Tol/EtOAc 70:30 → 65:35) gave the fucosaminide derivative **54** (910 mg, 2.33 mmol, 82%) as a white solid. The latter had *R*_*f*_ 0.35 (Tol/EtOAc, 7:3). ^1^H NMR (400 MHz, CDCl_3_): δ 6.92 (d, 1H, *J*_2,NH_ = 8.8 Hz, NH), 5.89–5.81 (m, 1H, CH_All_), 5.30–5.16 (m, 3H, H-3, CH_2All_), 4.68 (d, 1H, *J*_1,2_ = 8.4 Hz, H-1), 4.40–4.34 (m, 1H, CH_2All_), 4.25–4.18 (m, 1H, H-2), 4.12–4.06 (m, 1H, CH_2All_), 3.84 (d, 1H, *J*_3,4_ = 2.9 Hz, H-4), 3.74 (q, 1H, H-5), 2.49 (brs, 1H, OH), 2.12 (s, 3H, CH_3Ac_), 1.36 (d, 3H, *J*_5,6_ = 6.3 Hz, H-6). ^13^C{^1^H} NMR (100 MHz, CDCl_3_): δ 170.95 (CO_Ac_), 162.16 (CO_NH_), 133.57 (CH_All_), 117.65 (CH_2All_), 99.77 (C-1), 92.48 (CCl_3_), 72.50 (C-3), 70.65 (C-5), 70.10 (CH_2All_), 69.77 (C-4), 52.80 (C-2), 20.84 (CH_3Ac_), 16.22 (C-6). HRMS (ESI^+^) *m*/*z*: [M + NH_4_]^+^ calcd for C_13_H_22_Cl_3_N_2_O_6_, 407.0538; found, 407.0530.

#### Allyl 3-*O*-Acetyl-2-azido-2,6-dideoxy-β-d-galactopyranoside (**55**)

Pyridine (236 μL, 2.95 mmol, 2.0 equiv) and triflic anhydride (346 μL, 2.06 mmol, 1.4 equiv) were added to alcohol **51** (400 mg, 1.47 mmol, 1.0 equiv) in anhyd. DCM (10 mL) at −10 °C. After stirring for 1 h at −10 °C, a TLC analysis (Tol/EtOAc 4:1) indicated the conversion of the starting **51** (*R*_*f*_ 0.35) to a less polar compound (*R*_*f*_ 0.95). DCM (10 mL) was added, and the reaction mixture was washed with 20% aq CuSO_4_ (10 mL) and brine (10 mL). The organic layer was separated, dried over Na_2_SO_4_, concentrated, and extensively dried under high vacuum. The crude was dissolved in anhyd. DMF (8.0 mL), and TBANO_2_ (851 mg, 2.95 mmol, 2.0 equiv) was added. After stirring overnight, DCM (50 mL) was added, and the reaction was washed with 50% aq NaCl (50 mL). The aq phase was extracted with DCM (2 × 20 mL). The combined organic phases were dried over Na_2_SO_4_ and concentrated. Flash chromatography (cHex/EtOAc 65:35 → 60:40) of the crude gave the expected alcohol **55** (290 mg, 1.06 mmol, 73%) as a white solid. Compound **55** had *R*_*f*_ 0.2 (Tol/EtOAc 9:1). ^1^H NMR (400 MHz, CDCl_3_): δ 5.99–5.91 (m, 1H, CH_All_), 5.38–5.33 (m, 1H, CH_2All_), 5.26–5.23 (m, 1H, CH_2All_), 4.70 (dd, 1H, *J*_3,4_ = 3.0 Hz, *J*_2,3_ = 10.4 Hz, H-3), 4.45–4.40 (m, 1H, CH_2All_), 4.36 (d, 1H, *J*_1,2_ = 8.0 Hz, H-1), 4.19–4.14 (m, 1H, CH_2All_), 3.81 (brs, 1H, H-4), 3.71 (dd, 1H, H-2), 3.65 (q, 1H, H-5), 2.18 (s, 3H, CH_3Ac_), 2.11 (brs, 1H, OH), 1.33 (d, 3H, *J*_5,6_ = 6.6 Hz, H-6). ^13^C{^1^H} NMR (100 MHz, CDCl_3_): δ 170.11 (CO_Ac_), 133.35 (CH_All_), 117.92 (CH_2All_), 101.07 (C-1), 74.07 (C-3), 70.39 (C-5), 70.35 (CH_2All_), 69.34 (C-4), 60.70 (C-2), 20.93 (CH_3Ac_), 16.10 (C-6). HRMS (ESI^+^) *m*/*z*: [M + NH_4_]^+^ calcd for C_11_H_21_N_4_O_5_, 289.1506; found, 289.1494.

#### Allyl 3-*O*-Acetyl-2,4-diazido-2,4,6-trideoxy-β-d-glucopyranoside (**56**)

Pyridine (26 μL, 336 μmol, 1.3 equiv) and triflic anhydride (52 μL, 310 μmol, 1.2 equiv) were added to a solution of alcohol **55** (70 mg, 258 μL, 1.0 equiv) in anhyd. DCM (3.0 mL) cooled to −10 °C. After stirring for 2 h at this temperature, DCM (5 mL) was added. The organic phase was washed with 20% aq CuSO_4_ (10 mL), aq NaHCO_3_ (10 mL), and brine (10 mL). The DCM part was separated, dried over Na_2_SO_4_, concentrated, and dried under high vacuum. NaN_3_ (149 mg, 516 μmol, 2.0 equiv) was added to the crude in anhyd. DMF (3.0 mL). After stirring for 6 h at rt, DCM was added, and the DCM layer was washed with water and brine. The organic layer was separated, dried, and concentrated. Flash chromatography (Tol/EtOAc 95:5 → 90:10) of the crude gave the diazide **56** (52 mg, 175 μmol, 68%) as a white solid. Compound **56** had *R*_*f*_ 0.75 (Tol/EtOAc 9:1). ^1^H NMR (400 MHz, CDCl_3_): δ 5.98–5.90 (m, 1H, CH_All_), 5.38–5.33 (m, 1H, CH_2All_), 5.28–5.24 (m, 1H, CH_2All_), 4.95 (dd, 1H, *J*_3,4_ = *J*_2,3_ = 10.0 Hz, H-3), 4.42–4.38 (m, 2H, CH_2All_, H-1), 4.19–4.13 (m, 1H, CH_2All_), 3.44 (dd, 1H, *J*_1,2_ = 8.0 Hz, H-2), 3.34 (dq, 1H, *J*_4,5_ = 9.8 Hz, H-5), 3.18 (t, 1H, *J*_3,4_ = 10.0 Hz, H-4), 2.19 (s, 3H, CH_3Ac_), 1.40 (d, 3H, *J*_5,6_ = 6.1 Hz, H-6). ^13^C{^1^H} NMR (400 MHz, CDCl_3_): δ 169.63 (CO_Ac_), 133.11 (CH_All_), 118.16 (CH_2All_), 100.54 (C-1), 72.89 (C-3), 70.68 (C-5), 70.48 (CH_2All_), 65.81 (C-4), 64.18 (C-2), 20.71 (CH_3Ac_), 18.23 (C-6). HRMS (ESI^+^): calcd for C_11_H_17_N_6_O_4_, [M + H]^+^*m*/*z* 297.1306; found, 297.1303.

#### Allyl 3-*O*-*tert*-Butyldimethylsilyl-2,6-dideoxy-2-trichloroacetamido-β-d-galactopyranoside (**57**)

NaOMe (25% NaOMe in MeOH, 36 μL, 167 μmol, 0.1 equiv) was added to a solution of acetate **54** (650 mg, 1.67 mmol, 1.0 equiv) in MeOH (12 mL). After stirring for 1 h at rt, Dowex-H^+^ resin was added to reach pH ∼ 7.0. The suspension was filtered and washed thoroughly with MeOH, and the filtrate was concentrated to dryness. The crude diol was dissolved in anhyd. DMF (10 mL). TBSCl (277 mg, 1.83 mmol, 1.1 equiv) was added, followed by the addition of imidazole (170 mg, 2.50 mmol, 1.5 equiv). After stirring overnight at rt, a TLC analysis (Tol/EtOAc, 1:1) showed complete conversion of the starting material (*R*_*f*_ 0.2) into a less polar product (*R*_*f*_ 0.6). MeOH was added, and after an additional hour, volatiles were evaporated. Flash chromatography of the crude (Tol/EtOAc 15:85 → 50:50) gave the silylated derivative **57** as a white solid (640 mg, 1.38 mmol, 83%). Alcohol **57** had *R*_*f*_ 0.5 (Tol/EtOAc 4:1). ^1^H NMR (400 MHz, CDCl_3_): δ 6.87 (d, 1H, *J*_2,NH_ = 7.2 Hz, NH), 5.93–5.83 (m, 1H, CH_All_), 5.29–5.24 (m, 1H, CH_2All_), 5.19–5.15 (m, 1H, CH_2All_), 4.95 (d, 1H, *J*_1,2_ = 8.4 Hz, H-1), 4.42 (dd, 1H, *J*_2,3_ = 10.2 Hz, *J*_3,4_ = 3.4 Hz, H-3), 4.38–4.33 (m, 1H, CH_2All_), 4.11–4.05 (m, 1H, CH_2All_), 3.69 (dq, 1H, *J*_4,5_ = 0.8 Hz, H-5), 3.66 (d, 1H, *J*_3,4_ = 3.4 Hz, H-4), 3.54–3.48 (m, 1H, H-2), 2.45 (brs, 1H, OH), 1.41 (d, 3H, *J*_5,6_ = 6.5 Hz, H-6), 0.92 (s, 9H, CH_3*t*Bu_), 0.13, 0.12 (2s, 6H, CH_3TBS_). ^13^C{^1^H} NMR (100 MHz, CDCl_3_): δ 161.80 (CO_NH_), 133.79 (CH_All_), 117.80 (CH_2All_), 97.45 (C-1), 92.49 (CCl_3_), 71.68 (C-4), 70.45 (C-3), 70.11 (CH_2All_), 69.92 (C-5), 57.12 (C-2), 25.67 (CH_3tBu_), 17.87 (C_q*t*Bu_), 16.44 (C-6), −4.53, −4.73 (2C, CH_3TBS_). HRMS (ESI^+^) *m*/*z*: [M + NH_4_]^+^ calcd for C_17_H_34_Cl_3_N_2_O_5_Si, 479.1297; found, 479.1291.

## Data Availability

The data underlying this study are available in the published article and its Supporting Information.

## Supporting Information Available

Abbreviations, experimental procedures, analytical data of the synthesized compounds, as well as ^1^H and ^13^C NMR spectra of novel compounds (PDF)
