# Supplementary material for: Scalable Synthesis of Versatile Rare Deoxyamino Sugar Building Blocks from d-Glucosamine
Source: J Org Chem. 2023 May 4;88(11):6645–63. doi: 10.1021/acs.joc.2c03016 (PMC10242766; doi:10.1021/acs.joc.2c03016)
Supplement: Supplementary file 1 — jo2c03016_si_001.pdf [file jo2c03016_si_001.pdf]

Supporting information

Scalable synthesis of versatile rare deoxyamino sugar building blocks from D-glucosamine

Debashis Dhara,[a] Marion Bouchet,[a] and Laurence A Mulard[a]*

[a] Institut Pasteur, Université Paris Cité, CNRS UMR3523, Chemistry of Biomolecules Laboratory, 28 rue du Dr Roux, 75015 Paris, France

Table of contents

1. Abbreviations p S2

2. Supplementary schemes showing S1-S15 and related protocols p S2

3. Supplementary figure showing side-products S16-S22 p S15

4. Experimental protocols and analytics for side-products S16-S22 p S15

5. References p S18

6. ^{1}H and ^{13}C NMR spectra of novel compounds p S19
3, 7-9, 11-21, 23-44, 46-49, 51-52, 54-57, S2, S4, S6, S7, S9-S22

1. Abbreviations

Ac: acetyl, All: allyl, anhyd.: anhydrous, aq.: aqueous, Ar: argon, $BF_3 \cdot OEt_2$: boron trifluoride diethyl etherate, Bn: benzyl, Boc: *tert*-butyloxycarbonyl, Boc_2O: di-*tert*-butyl dicarbonate, CA: chloroacetyl, calc.: calculated, Cbz: benzyloxycarbonyl, CD_3CN: deuterated acetonitrile, $CDCl_3$: deuterated chloroform, cHex: cyclohexane, CSA: camphorsulfonic acid, DCA: dichloroacetyl, $(DCA)_2O$: dichloroacetic anhydride, D_2O: deuterated water, DCA: dichloroacetyl, DCE: 1,2-dichloroethane, DCM: dichloromethane, DMAP: 4-(dimethylamino)pyridine, DMF: *N*,*N*-dimethylformamide, DMSO-d_6*:* deuterated dimethylsulfoxide, equiv.: equivalent, EtOAc: ethyl acetate, MBn: *para*-methoxybenzylidene, MeCN: acetonitrile, MeOH: methanol, MeOH-d_4: deuterated methanol, Ms: mesyl, MS: molecular sieves, TCA: trichloroacetyl, TFA: trifluoroacetyl, Py: pyridine, rt: room temperature, satd: saturated, TBDPS: *tert*-butyldiphenylsilyl, TBS: *tert*-butyldimethylsilyl, TCP: tetrachlorophthaloyl, TCPO: tetrachlorophthalic anhydride, Tf: trifluoromethanesulfonyl, Tf_2O: triflic anhydride, TfN_3: trifluoromethanesulfonyl azide, THF: tetrahydrofuran, TLC: thin-layer chromatography, TMS: trimethysilyl, TMSOTf: trimethylsilyl trifluoromethanesulfonate, Tol: toluene, Troc: 2,2,2-trichloroethoxycarbonyl, Trs: trisyl, Ts: tosyl.

2. Supplementary schemes showing S1-S15 and related protocols

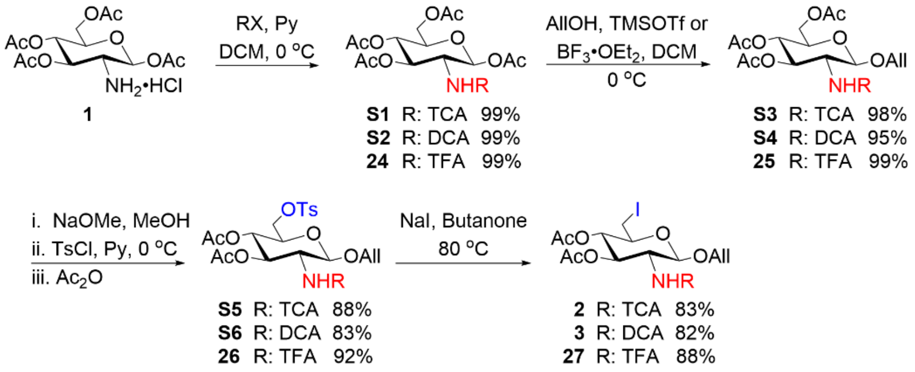

Scheme S1. Synthesis of allyl glycosides **2**, **3** and **27** from 1,2,3,4-tetra-*O*-acetyl-D-glucosamine hydrochloride.[1] X = Cl (for R: TCA), X = OC(O)$CHCl_2$ (for R: DCA) and X = OC(O)CF_3 (for R: TFA).

1,3,4,6-Tetra-*O*-acetyl-2-deoxy-2-dichloroacetamido-*β*-D-glucopyranose (S2). Commercially available 1,3,4,6-tetra-*O*-acetyl-2-amino-2-deoxy-*β*-D-glucopyranose hydrochloride (10.0 g, 26.1 mmol, 1.0 equiv.) was solubilized in anhyd. DCM (100 mL) at 0 °C. Anhyd. pyridine (10.5 mL, 130 mmol, 5.0 equiv.) and then dichloroacetic anhydride (7.5 mL, 78.3 mmol, 3.0 equiv.) were added slowly. The mixture was stirred for 1 h during which time it reached rt. TLC (Tol/EtOAc 2:1) indicated the total conversion of the starting material to less polar products (R_f 0.4). MeOH (5 mL) was added and after an additional 5 min, DCM (100 mL) was added. The organic layer was washed with 1 N aq. HCl (200 mL) and satd aq. $NaHCO_3$ (200 mL), dried over Na_2SO_4, filtered, and concentrated *in vacuo*. Flash chromatography of the residue (Tol/EtOAc 2:1→1:1) gave the desired **S2** (11.8 g, 99%). The

dichloroacetamide **S2** had R_f 0.4 (Tol/EtOAc 2:1). ^{1}H NMR (400 MHz, $CDCl_3$) δ 6.90 (d, 1H, $J_{NH,2}$ = 9.4 Hz, NH), 5.88 (s, 1H, $CHCl_2$), 5.82 (d, 1H, $J_{1,2}$ = 8.2 Hz, H-1), 5.38 (dd, 1H, $J_{3,2}$ = 9.2 Hz, $J_{3,4}$ = 10.6 Hz, H-3), 5.16 (t, 1H, $J_{4,3}$ = $J_{4,5}$ = 9.7 Hz, H-4), 4.34-4.26 (m, 2H, H-2, H-6a), 4.18 (dd, 1H, $J_{6a,6b}$ = 2.4 Hz, $J_{6b,5}$ = 12.8 Hz, H-6b), 3.91 (ddd, 1H, $J_{6a,5}$ = 4.8 Hz, H-5), 2.12, 2.11, 2.08, 2.07 (4s, 12H, CH_{3Ac}). ^{13}C{^{1}H} NMR (100 MHz, $CDCl_3$) δ 171.20, 170.58, 169.29, 169.24 (4C, CO_{Ac}), 164.73 (CO_{NHDCA}), 92.04 (C-1), 73.12 (C-5), 71.82 (C-3), 67.96 (C-4), 66.06 ($CHCl_2$), 61.69 (C-6), 53.45 (C-2), 20.70, 20.66, 20.51 (4C, C_{Ac}). HRMS (ESI$^+$): *m/z* [M+Na]$^+$ calc. for $C_{16}H_{21}Cl_2NO_{10}Na$ 480.0440; found 480.0441.

Allyl 3,4,6-tri-*O*-acetyl-2-deoxy-2-dichloroacetamido-*β*-D-glucopyranoside (S4). A solution of the dichloroacetamide derivative **S2** (10.0 g, 21.8 mmol, 1.0 equiv.) and allyl alcohol (4.4 mL, 65.6 mmol, 3.0 equiv.) in anhyd. DCM (110 mL) was stirred with freshly activated MS 4 Å (5.0 g) for 1 h under an Ar atmosphere. The suspension was cooled to 0 °C and TMSOTf (4.35 mL, 24.0 mmol, 1.1 equiv.) was added dropwise. After stirring for 1 h at this temperature, a TLC analysis (DCM/EtOAc 9:1) indicated the conversion of the starting material (R_f 0.25) into a closely migrating compound (R_f 0.3). Et_3N (1.0 equiv.) was added. The suspension was filtered and solids were washed with DCM (2×50 mL). The organic layer was washed with H_2O and brine, dried over Na_2SO_4, filtered, and concentrated *in vacuo*. Flash chromatography of the crude solid (Tol/EtOAc 75:25→65:35) gave the desired product **S4** (9.5 g, 20.8 mmol, 95%). Allyl glycoside **S4** had R_f 0.3 (DCM/EtOAc 9:1). ^{1}H NMR (400 MHz, $CDCl_3$) δ 6.88 (d, 1H, $J_{NH,2}$ = 8.9 Hz, NH), 5.91 (s, 1H, $CHCl_2$), 5.89-5.79 (m, 1H, CH_{All}), 5.39 (dd, 1H, $J_{3,4}$ = 9.3 Hz, $J_{3,2}$ = 10.7 Hz, H-3), 5.29-5.24 (m, 1H, CH_{2All}), 5.20-5.17 (m, 1H, CH_{2All}), 5.09 (t, 1H, $J_{4,3}$ = $J_{4,5}$ = 9.6 Hz, H-4), 4.73 (d, 1H, $J_{1,2}$ = 8.4 Hz, H-1), 4.36-4.30 (m, 1H, CH_{2All}), 4.27 (dd, 1H, $J_{6a,6b}$ = 12.3 Hz, $J_{6a,5}$ = 5.0 Hz, H-6a), 4.16 (dd, 1H, $J_{6b,5}$ = 2.4 Hz, H-6b), 4.12-4.06 (m, 1H, CH_{2All}), 3.99 (m, 1H, H-2), 3.75 (ddd, 1H, H-5), 2.08 (s, 3H, CH_{3Ac}), 2.02 (s, 6H, CH_{3Ac}). ^{13}C{^{1}H} NMR (100 MHz, $CDCl_3$) δ 170.82, 170.64, 169.29 (3C, CO_{Ac}), 164.58 (CO_{NHDCA}), 133.33 (CH_{All}), 117.93 (CH_{2All}), 99.30 (C-1), 71.90 (C-5), 71.71 (C-3), 70.12 (CH_{2All}), 68.75 (C-4), 66.23 ($CHCl_2$), 62.16 (C-6), 54.92 (C-2), 20.67, 20.55 (3C, C_{Ac}). HRMS (ESI$^+$): *m/z* [M+Na]$^+$ calc. for $C_{17}H_{23}Cl_2NO_9Na$ 478.0648; found 478.0648.

Allyl 3,4-di-*O*-acetyl-2-deoxy-2-dichloroacetamido-6-*O*-tosyl-*β*-D-glucopyranoside (S6). NaOMe (338 mg, 6.2 mmol, 0.3 equiv.) was added to a solution of allyl glycoside **S4** (9.5 g, 20.8 mmol, 1.0 equiv.) in MeOH (100 mL). The reaction mixture was stirred for 1 h, at which time a TLC analysis (EtOAc/MeOH 9:1) indicated the total consumption of the starting material (R_f 0.95) and the presence of a more polar product (R_f 0.2). Dowex-H$^+$ resin was added to reach pH ~7. The suspension was filtered and the resin washed thoroughly with MeOH (2×15 mL). The filtrate was concentrated, dried over Na_2SO_4, filtered, and concentrated to dryness. The crude was dissolved in anhyd. pyridine (120 mL), cooled to 0 °C, and tosyl chloride (11.9 g, 62.6 mmol, 3.0 equiv.) was added. After stirring for 3 h at 0 °C, acetic anhydride (9.8 mL, 104.3 mmol, 5.0 equiv.) was added. The reaction mixture was stirred for another 3 h while reaching rt. MeOH (10 mL) was added and volatiles were co-evaporated with toluene (2×20 mL). Flash chromatography of the crude (Tol/EtOAc 85:15→80:20) gave diacetate **S6** as a white solid (9.8 g, 17.2 mmol, 83%). The 6-*O*-tosyl derivative **S6** had R_f 0.25 (Tol/EtOAc 4:1). ^{1}H NMR (400 MHz, $CDCl_3$) δ 7.80 (d, 2H, J = 8.4 Hz, H_{Ar}), 7.37 (d, 2H,

H_{Ar}), 6.64 (d, 1H, $J_{NH,2}$ = 9.2 Hz, NH), 5.89 (s, 1H, $CHCl_2$), 5.87-5.77 (m, 1H, CH_{All}), 5.35 (dd, 1H, $J_{4,3}$ = 9.2 Hz, $J_{2,3}$ = 10.6 Hz, H-3), 5.30-5.24 (m, 1H, CH_{2All}), 5.22-5.19 (m, 1H, CH_{2All}), 4.95 (t, 1H, $J_{4,3}$ = $J_{4,5}$ = 9.3 Hz, H-4), 4.68 (d, 1H, $J_{1,2}$ = 8.3 Hz, H-1), 4.30-4.25 (m, 1H, CH_{2All}), 4.18-4.10 (m, 2H, H-6a, H-6b), 4.07-4.02 (m, 1H, CH_{2All}), 3.95-3.88 (m, 1H, H-2), 3.81 (ddd, 1H, $J_{5,6a}$ = 3.2 Hz, $J_{5,6b}$ = 5.6 Hz, H-5), 2.47 (s, 3H, CH_{3Ts}), 2.03 (s, 6H, CH_{3Ac}). $^{13}C\{^1H\}$ NMR (100 MHz, $CDCl_3$) δ 170.74, 169.41 (2C, CO_{Ac}), 164.42 (CO_{NHDCA}), 145.18 (C_{Ar}), 133.11 (CH_{All}),132.54, 129.91, 128.04 (C_{Ar}), 118.12 (CH_{2All}), 99.12 (C-1), 71.82 (C-5), 71.34 (C-3), 70.08 (CH_{2All}), 68.85 (C-4), 67.96 (C-6), 66.15 ($CHCl_2$), 54.87 (C-2), 21.64 (C_{Me}), 20.53 (2C, C_{Ac}). HRMS (ESI$^+$): *m/z* [M+Na]$^+$ calc. for $C_{22}H_{27}Cl_2NO_{10}SNa$ 590.0630; found 590.0663.

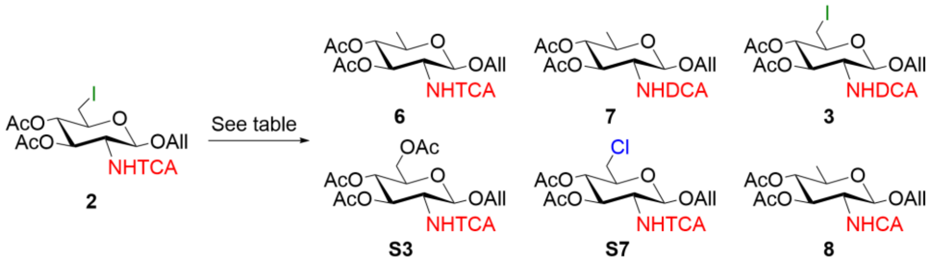

Entry	Scale (g)	Reducing agent (equiv.)	Conditions	Products (Yield)
1	5.0	$NaCNBH_3$ (3.0)	DMF, 110 °C, 24 h	**6** (57%), **7** (8%) **S7** (7%)
2	1.0	$NaCNBH_3$ (3.0)	THF, 70 °C, 6 h	**2** (recovered)
3[2]	1.0	$NaCNBH_3$ (3.0) CuI (0.5)	THF, 80 °C, 5 h	**6** (20%), **7** (24%)
4[2]	1.0	$NaCNBH_3$ (3.0) CuCl (0.5)	THF, 70 °C, 5 h	**6** (10%), **7** (33%) **3** (14%)
5	1.0	$NaBH(OAc)_3$ (3.0)	DMF, 110 °C, 16 h	**6** (25%), **S3**[3] (27%)
6[4]	0.5	Bu_3SnH (5.0) Et_3B (0.3)	Tol, 0 °C, 1 h	**8** (74%)
7	0.5	Bu_3SnH (1.2) Et_3B (0.1)	Tol, 0 °C, 1 h	**6** (32%), **7** (22%) **8** (12%)
8[5]	0.2	$(TMS)_3SiH$ (1.5) AIBN (0.1)	Tol, 80 °C, 3 h	**6** (33%), **7** (25%)
9[6]	0.5	$(TMS)_3SiH$ (1.2) Et_3B (0.1)	Tol, 0 °C→ rt, 1 h	**6** (36%), **2** (8%)
10	0.5	$(TMS)_3SiH$ (1.5) Et_3B (0.2)	Tol, 0 °C→ rt, 2 h	**6** (33%), **7** (11%) **2** (6%)
11[b]	0.5	$(TMS)_3SiH$ (1.2) Et_3B (0.1)	THF, -78 °C → 0 °C, 1 h	**6** (14%), **2** (54%)

[a] Reported yields are based on isolated products. [b] Longer reaction time led to additional side-product formation. $(TMS)_3SiH$: Tris(trimethyl)silane, AIBN: 2,2'-azobis(2-methylpropionitrile).

Scheme S2. C-6 reduction of diacetate **2** by means of hydrides (Entries 1, 2 and 5) or radical-mediated conditions (Entries 3, 4 and 6-11).

Allyl 3,4-di-*O*-acetyl-2,6-dideoxy-2-trichloroacetamido-*β*-D-glucopyranoside (6)[7] **and allyl 3,4-di-*O*-acetyl-6-chloro-2,6-dideoxy-2-trichloroacetamido-*β*-D-glucopyranoside (S7).** The known 6-iodo derivative[7] **2** (5.0 g, 8.97 mmol, 1.0 equiv.) was dissolved in anhyd. DMF (90 mL). Sodium cyanoborohydride (1.6 g, 26.9 mmol, 3.0 equiv.) was added and the reaction mixture was stirred at 110 °C for 24 h under an Ar atmosphere. At completion, the reaction mixture was left to reach rt, diluted with water (100 mL) and extracted with DCM (3 × 100 mL). The organic phases were pooled, washed with brine, dried over Na_2SO_4, filtered and concentrated *in vacuo*. The crude was purified by flash chromatography (Tol/EtOAc 4:1) to give by order of elution the known 6-deoxy target **6**[7] (2.2 g, 5.1 mmol, 57%), the dichloroacetamide **7** (300 mg, 755 µmol, 8%) and the 6-chloro derivative **S7** (280 mg, 650 µmol, 7%), all as white solids. Analytical data for compound **6** were as described.[7]

The 6-chloro derivative **S7** had R_f 0.35 (Tol/EtOAc 4:1). ^{1}H NMR (400 MHz, $CDCl_3$) δ 6.74 (d, 1H, $J_{2,NH}$ = 8.8 Hz, NH), 5.92-5.82 (m, 1H, CH_{2All}), 5.38 (dd, 1H, $J_{2,3}$ = 10.2 Hz, $J_{3,4}$ = 9.2 Hz, H-3), 5.34-5.29 (m, 1H, CH_{2All}), 5.25-5.22 (m, 1H, CH_{2All}), 5.06 (t, 1H, $J_{4,5}$ = 9.2 Hz, H-4), 4.78 (d, 1H, $J_{1,2}$ = 8.2 Hz, H-1), 4.43-4.37 (m, 1H, CH_{2All}), 4.18-4.13 (m, 1H, CH_{2All}), 4.02-3.95 (m, 1H, H-2), 3.76 (ddd, 1H, H-5), 3.68-3.58 (m, 2H, H-6a, H-6b), 2.08 (s, 3H, CH_{3Ac}), 2.05 (s, 3H, CH_{3Ac}). ^{13}C{^{1}H} NMR (100 MHz, $CDCl_3$) δ 170.70, 169.37 (2C, CO_{Ac}), 161.88 (CO_{NHTCA}), 133.03 (CH_{All}), 118.41 (CH_{2All}), 98.99 (C-1), 92.23 (CCl_3), 73.95 (C-5), 71.26 (C-3), 70.21 (C-4), 70.16 (CH_{2All}), 56.12 (C-2), 43.23 (C-6), 20.63, 20.52 (2C, CH_{3Ac}). HRMS (ESI$^+$): *m/z* [M+Na]$^+$ calc. for $C_{15}H_{19}Cl_4NO_7Na$ *m/z* 487.9813; found 487.9789.

Allyl 3,4-di-*O*-acetyl-2,6-dideoxy-2-trichloroacetamido-*β*-D-glucopyranoside (6) and allyl 3,4-di-*O*-acetyl-2-dichloroacetamido-2,6-dideoxy-6-iodo-*β*-D-glucopyranoside (3). The 6-iodo derivative **2** (1.0 g, 1.79 mmol, 1.0 equiv.) was dissolved in anhyd. THF (18 mL). $NaCNBH_3$ (338 mg, 5.38 mmol, 3.0 equiv.) and CuCl (89 mg, 898 µmol, 0.5 equiv.) were added. After heating at 80 °C for 5 h under an Ar atmosphere (TLC analysis: Tol/EtOAc, 4:1), the reaction mixture was cooled to rt and quenched by addition of aq. NH_4Cl. The aq. layer was extracted with DCM (40 mL) twice. The combined DCM parts were dried over Na_2SO_4 and concentrated under *vacuo*. Flash chromatography (Tol/EtOAc 85:15→75:25) of the crude gave compounds **3** (130 mg, 248 µmol, 14%), **6** (80 mg, 186 µmol, 10%) and **7** (240 mg, 604 µmol, 33%). Analytical data for **3** and **7** were as described in the main document. Those for **6** were as published.[7]

Allyl 3,4-di-*O*-acetyl-2,6-dideoxy-2-trichloroacetamido-*β*-D-glucopyranoside (6), allyl 3,4-di-*O*-acetyl-2-dichloroacetamido-2,6-dideoxy-*β*-D-glucopyranoside (7) and allyl 3,4-di-*O*-acetyl-2-chloroacetamido-2,6-dideoxy-*β*-D-glucopyranoside (8). *Route a.* Bu_3SnH (289 µL, 1.07 mmol, 1.2 equiv.) followed by Et_3B (1 M in Hexane, 90 µL, 90 µmol, 0.1 equiv.) were added to a solution of **2** (500 mg, 90 µmol, 1.0 equiv.) in anhyd. toluene (18 mL) at 0 °C. After stirring for 1 h at 0 °C, a follow up by TLC (Tol/EtOAc 4:1) indicated the absence of the starting material (R_f 0.4) and the presence of more polar products (R_f 0.35, 0.25, 0.15). Flash chromatography of the crude material (Tol/EtOAc 55:45→20:80) gave the desired 6-deoxy product **6** (130 mg, 290 µmol, 32%), the dichloroacetamide **7** (80 mg, 201 µmol, 22%), and the chloroacetamide **8** (40 mg, 110 µmol, 12%), all isolated as white solids. Analytical data for side-products **7** and **8** were as described in the main document.

Route b. The 6-iodo derivative **2** (500 mg, 989 µmol, 1.0 equiv.) was dissolved in anhyd. toluene and cooled to 0 °C keeping the mixture under an Ar atmosphere. Tris(trimethylsilyl) silane (TMS_3SiH, 415 µL, 1.34 mmol, 1.5 equiv.) was added followed by the slow addition of triethyl borane (Et_3B, 1 M in hexane, 180 µL, 180 µmol, 0.2 equiv.) at 0 °C. The bath was allowed to reach rt within 1 h. After another 1 h at rt, (TLC analysis: Tol/EtOAc 4:1), MeOH (0.5 mL) was added and volatiles were evaporated. Flash chromatography (Tol/EtOAc 80:20→70:30) of the crude gave by order of elution the starting material **2** (30 mg, 53.8 µmol, 6%), the desired 6-deoxy product **6** (130 mg, 301 µmol, 33%) and the dichloroacetamide **7** (40 mg, 100 µmol, 11%). Analytical data for side-product **7** were as described in the main document.

Allyl 3,4-di-*O*-acetyl-2,6-dideoxy-2-trichloroacetamido-*β*-D-glucopyranoside (6) and allyl 3,4,6-tri-*O*-acetyl-2-deoxy-2-trichloroacetamido-*β*-D-glucopyranoside (S3). Sodium triacetoxyborohydride ($NaBH(OAc)_3$, 1.14 g, 5.38 mmol, 3.0 equiv.) was added to a solution of the 6-iodo derivative[7] **2** (1.0 g, 1.79 mmol, 1.0 equiv.) in anhyd. DMF (12 mL) at rt. The reaction mixture was heated at 110 °C for 16 h. A TLC analysis (Tol/EtOAc 4:1) indicated the presence of more polar compounds (R_f 0.4, 0.0). Volatiles were eliminated. Flash chromatography of the crude (Tol/EtOAc 80:20→50:50) gave by order of elution the desired 6-deoxy product **6** (200 mg, 464 µmol, 25%) and the known triacetate **S3**[3] (240 mg, 490 µmol, 27%). Analytical data were as described.[3]

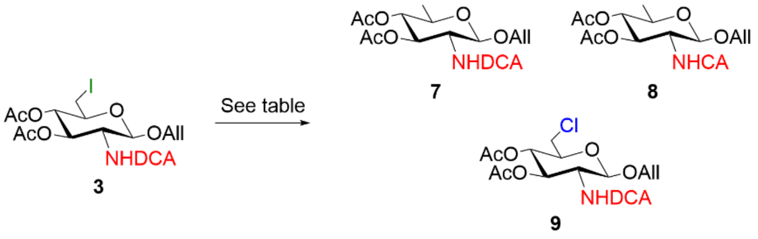

Entry	Scale (mg)	Reducing agent (equiv.)	Conditions	Products (ratio, Yield)[a]
1[7]	500	$NaBH_3CN$ (3.0)	DMF, 110 °C, 20 h	**7**+**9** (~2:1, 84%)
2	4,000	$NaBH_3CN$ (3.0)	DMF, 110 °C, 24 h	**7**+**9** (~2:1, 23%)
3[2]	250	$NaBH_3CN$ (3.0)<br>CuI (0.5)	DMF, 80 °C, 24 h	**7**+**9** (~2:1, 60%)
4[4]	75	Bu_3SnH (5.0)<br>Et_3B (0.1)	DCM, 0 °C, 1 h	**8** (75%)
5	100	Bu_3SnH (1.1)<br>Et_3B (0.1)	DCM, 0 °C, 1 h	**7** (71%), **8** (11%)

[a] Reported yields are based on isolated products.

Scheme S3. C-6 reduction on diacetate **3** by means of hydrides (Entries 1-2) or radical-mediated conditions (Entries 3-5).

Allyl 3,4-di-*O*-acetyl-2-chloroacetamido-2,6-dideoxy-*β*-D-glucopyranoside (8). The 6-iodo derivative **3** (75 mg, 143 µmol, 1.0 equiv.) in DCM (3 mL) was cooled to 0 °C. Bu_3SnH (165 µL, 717 µmol, 5.0 equiv.) was added, followed by Et_3B (1 M in hexane, 29 µL, 29 µmol, 0.2 equiv.). After stirring for 1 h at 0 °C, a TLC analysis (Tol/EtOAc 6:4) showed the absence of **3** (R_f 0.7) and appearance of a single more polar spot (R_f 0.5). MeOH was added and volatiles were eliminated under reduced pressure. Flash chromatography (Tol/EtOAc 60:40→50:50) of the residue gave chloroacetamide **8** (39 mg, 107 µmol, 75%) as a white solid. Analytical data were as described in the main manuscript.

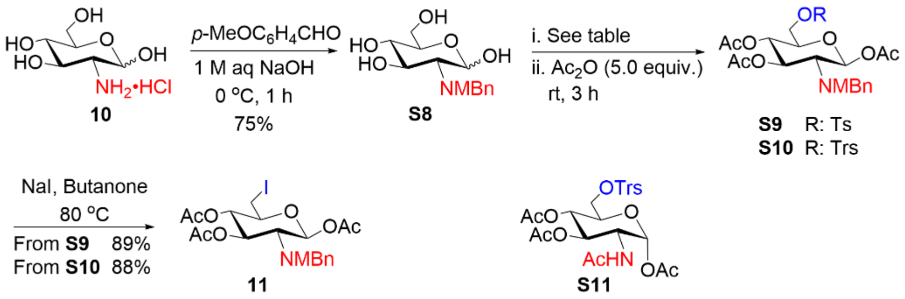

Entry	Scale (g)	Reagent (equiv.)	Conditions	Products (yield)[a]
1	1.0	TsCl (1.0)	Py, 0 °C, 3 h	**S9** (43%)
2	1.0	TsCl (1.5)	Py, 0 °C, 3 h	**S9** (40%)
3	2.0	TsCl (2.0)	Py, rt, 6 h	**S9** (31%)
4	0.5	TrsCl (1.5)	Py, 0 °C → rt, 24 h	**S10** (43%)
5	1.0	TrsCl (3.0)	Py, rt, 3 h	**S10** (38%)
6	2.0	TrsCl (4.0)	Py, rt, 3 h	**S10** (50%)
7	1.0	TrsCl (1.5)	Py, 0 °C, 40 h	**S10** (57%)
**8**	**5.0**	**TrsCl (1.0)**	**Py, -10 °C, 24 h**	**S10 (62%), S11[b] (11%)**
**9**	1.0	TrsCl (1.1)	Py, -15 °C, 40 h	**S10** (30%)

[a] Yields are reported for the isolated products over two steps. [b] **S11** may also be present in reactions corresponding to other entries. As reflected during follow up and by the isolated yields of **S9** and **S10**, diverse side-products may also form during the two-step conversions.

Scheme S4. Synthesis of the 2-amino-2,6-dideoxy derivative **11** from glucosamine hydrochloride. MBn: *para*-methoxybenzylidene, Ts: tosyl; Trs: trisyl = 2,4,6-triisopropylbenzenesulfonyl.

1,3,4-Tri-*O*-acetyl-2-deoxy-2-*N*-(*para*-methoxybenzylidene)-6-*O*-tosyl-*β*-D-glucopyranose (S9). A suspension of the crude imine[8] **S8** prepared as described[8] from **10** (16.8 mmol, 1.0 equiv.) in anhyd. pyridine (84 mL) was cooled to 0 °C. Tosyl chloride (3.2 g, 16.8 mmol, 1.0 equiv.) was added portionwise over 1 h. After stirring for 24 h at 0 °C, a TLC (EtOAc/MeOH, 5:1) follow up showed the full starting material consumption and the presence

of one major spot (R_f 0.3). Acetic anhydride (6.68 mL, 70.6 mmol, 4.2 equiv.) was added dropwise to the reaction mixture maintained at 0 °C. After completion of the addition, the reaction mixture was allowed to reach rt and stirred at this temperature for another 3 h. MeOH (5.0 mL) was added and after an additional 15 min, volatiles were eliminated under reduced pressure. The crude was taken in DCM (200 mL) and washed successively with 1 M aq. $CuSO_4$ (200 mL), satd aq. $NaHCO_3$ (200 mL) and brine (200 mL). The organic layer was dried over Na_2SO_4, filtered and concentrated to dryness. Flash chromatography (Tol/EtOAc 85:15→80:20) gave the 6-*O*-tosyl target **S9** as a white solid (4.27 g, 7.39 mmol, 44%). The tosylate **S9** had R_f 0.7 (Tol/EtOAc 2:1). ^{1}H NMR (400 MHz, $CDCl_3$) δ 8.14 (s, 1H, CH=N), 7.81 (d, 2H, $H_{Ar,Ts}$), 7.65 (d, 2H, $H_{Ar,MBn}$), 7.37 (d, 2H, $H_{Ar,Ts}$), 6.93 (d, 2H, $H_{Ar,MBn}$), 5.89 (d, 1H, $J_{1,2}$ = 9.0 Hz, H-1), 5.38 (t, 1H, $J_{4,3}$ = $J_{2,3}$ = 9.7 Hz, H-3), 5.10 (t, 1H, $J_{4,5}$ = 9.7 Hz, H-4), 4.21-4.18 (m, 2H, H-6a, H-6b), 3.95 (ddd, $J_{5,6a}$ = 3.0 Hz, $J_{5,6b}$ = 4.0 Hz, H-5), 3.86 (s, 3H, OCH_3), 3.40 (dd, 1H, H-2), 2.48 (s, 3H, CH_{3Ts}), 2.01, 1.99, 1.88 (3s, 9H, CH_{3Ac}). ^{13}C{^{1}H} NMR (100 MHz, $CDCl_3$) δ 169.60, 169.55, 168.56 (3C, CO_{Ac}), 164.31 (CH=N), 162.34 ($C_{q,Ar}$), 144.95, 132.63 (2C, $C_{q,Ar}$), 130.23, 129.81, 128.17 (C_{Ar}), 128.3 ($C_{q,Ar}$), 114.07 (C_{Ar}), 93.06 (C-1, $^1J_{C,H}$ = 167 Hz), 73.14 (C-3), 72.70 (C-2), 72.18 (C-5), 68.20 (C-4), 67.11 (C-6), 55.39 (OCH_3), 21.63 (C_{Me}), 20.69, 20.54, 20.44 (3C, C_{Ac}). HRMS (ESI$^+$): *m/z* $[M+Na]^+$ calc. for $C_{27}H_{31}NO_{11}SNa$ 600.1516; found 600.1516.

1,3,4-Tri-*O*-acetyl-2-deoxy-2-*N*-(*para*-methoxybenzylidene)-6-*O*-(2,4,6-triisopropylbenzenesulfonyl)-*β*-D-glucopyranose (S10) and 2-acetamido-1,3,4-tri-*O*-acetyl-2-deoxy-6-*O*-(2,4,6-triisopropylbenzenesulfonyl)-*α*-D-glucopyranose (S11). A suspension of the crude imine **S8** prepared as described[8] from **10** (16.8 mmol, 1.0 equiv.) in anhyd. pyridine (168 mL) was cooled to -10 °C and 2,4,6-triisopropylbenzenesulfonyl chloride (5.0 g, 16.8 mmol, 1.0 equiv.) was added portionwise. After being stirred for 24 h at -10 °C, a TLC (DCM/MeOH, 5:1) follow up showed the presence of a major spot (R_f 0.5) and the absence of the starting **S8**. The reaction mixture reached rt and acetic anhydride (6.6 mL, 70.6 mmol, 4.2 equiv.) was added. After another 3 h, MeOH (5.0 mL) was added and stirring was pursued for 15 min. Volatiles were eliminated. The residue was dissolved in DCM (200 mL) washed successively with water (200 mL), 50% aq. $CuSO_4$ (200 mL) and brine (200 mL). The DCM layer was dried over Na_2SO_4, filtered, and concentrated to dryness. Flash chromatography of the crude residue (Tol/EtOAc 85:15→80:20) furnished by order of elution the sulfonate **S10** (7.2 g, 10.4 mmol, 62%) and the acetamide **S11** (1.1 g, 1.79 mmol, 11%) both as white solids. The target **S10** had R_f 0.55 (Tol/EtOAc 4:1). ^{1}H NMR (400 MHz, $CDCl_3$) δ 8.17 (s, 1H, CH=N), 7.68 (d, 2H, J = 8.8 Hz Hz, H_{Ar}), 6.94 (d, 2H, J = 8.8 Hz, H_{Ar}), 5.95 (d, 1H, $J_{1,2}$ = 8.4 Hz, H-1), 5.37 (dd, 1H, $J_{4,3}$ = 9.6 Hz, $J_{2,3}$ = 10.0 Hz, H-3), 4.97 (t, 1H, $J_{4,5}$ = 9.6 Hz, H-4), 3.92-3.78 (m, 6H, H-6a, H-6b, OCH_3, H-5), 3.95 (dd, 1H, H-2), 2.03, 2.00, 1.89 (3s, 9H, CH_{3Ac}), 1.08-1.06 (m, 21H, $CH(CH_3)_2$). ^{13}C{^{1}H} NMR (100 MHz, $CDCl_3$) δ 169.72, 169.64, 168.76 (CO_{Ac}), 163.89 (CH=N), 162.21 ($C_{q,Ar}$), 130.17 (C_{Ar}), 128.56 ($C_{q,Ar}$), 114.04 (C_{Ar}), 93.18 (C-1), 75.42 (C-5), 73.80 (C-3), 73.10 (C-2), 68.56 (C-4), 62.39 (C-6), 55.37 (OCH_3), 20.71, 20.55 (3C, C_{Ac}), 17.85, 17.82 (2C, CH_{TIPPS}), 12.27, 11.97 ($C_{Me,TIPPS}$). HRMS (ESI$^+$): *m/z* $[M+H]^+$ calc. for $C_{35}H_{47}NO_{11}S$ 690.2948; found *m/z* 690.2960.

The acetamide **S11** had R_f 0.35 (Tol/EtOAc 7:3). ^{1}H NMR (400 MHz, $CDCl_3$) δ 6.06 (d, 1H, $J_{1,2}$ = 3.6 Hz, H-1), 5.43 (dd_{po}, 1H, $J_{3,4}$ = 10.0 Hz, $J_{2,3}$ = 9.6 Hz, H-3), 5.13 (t, 1H, $J_{4,5}$ = 9.6

Hz, H-4), 4.72 (d, 1H, $J_{2,NH}$ = 9.6 Hz, NH), 4.26 (dd, 1H, $J_{5,6a}$ = 4.4 Hz, $J_{6a,6b}$ = 12.4 Hz, H-6a), 4.06-3.93 (m_{po}, 5H, H-2, H-5, H-6b, $2CH_{Pr}$), 2.90 (sep, 1H, J = 6.8 Hz, CH_{Pr}), 2.15, 2.08, 2.02, 1.19 (4s, 12H, $CH_{3,NHAc}$, CH_{3Ac}), 1.29-1.24 (m, 18H, CH_{3Pr}). $^{13}C\{^1H\}$ NMR (100 MHz, $CDCl_3$) δ 171.45, 170.55, 169.11, 168.19 (4C, CO_{Ac}), 153.15, 149.86, 133.70 ($C_{q,Ar}$), 129.01, 128.20, 125.28 (C_{Ar}), 91.47 (C-1, $^1J_{C,H}$ = 181 Hz), 70.31 (C-3), 69.36 (C-5), 67.93 (C-4), 61.53 (C-6), 54.42 (C-2), 34.15, 30.00 (3C, C_{Pr}), 24.84, 24.68, 23.50 (6C, C_{Pr}), 20.78, 20.61, 20.48, 20.36 (4C, C_{Ac}). HRMS (ESI^+): m/z $[M+NH_4]^+$ calc. for $C_{29}H_{47}N_2O_{11}S$ 631.2895; found 631.2892.

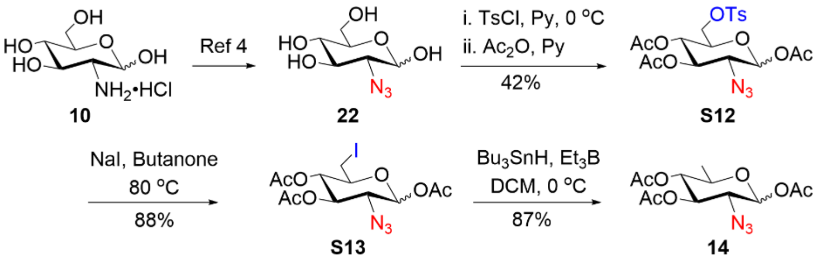

Scheme S5. Synthesis of the triacetate **14** from glucosamine hydrochloride **10** by means of azide **22**.[9]

1,3,4-Tri-*O*-acetyl-2-azido-2-deoxy-6-*O*-tosyl-*α*/*β*-D-glucopyranose (S12). Tosyl chloride (2.78 g, 14.6 mmol, 3.0 equiv.) was added to a suspension of commercially available 2-azido-2-deoxy-D-glucose[9] **22** (1.0 g, 4.87 mmol, 1.0 equiv.) in pyridine at 0 °C. After stirring for 4 h at this temperature, TLC (EtOAc) monitoring showed the presence of a main spot (R_f 0.75). Acetic anhydride (2.76 mL, 29.2 mmol, 6.0 equiv.) was added and the reaction was left for another 4 h while the temperature reached rt. A TLC analysis (Tol/EtOAc 4:1) showed the presence of a main less polar spot (R_f 0.6). MeOH (5 mL) was added and after stirring for another 15 min, volatiles were evaporated. The residue was diluted with DCM (50 mL) and the organic phase was washed with 1 N aq. HCl (100 mL), satd aq. $NaHCO_3$ (100 mL), and brine (100 mL). The DCM layer was dried over Na_2SO_4 and concentrated. Flash chromatography (Tol/EtOAc 90:10→80:20) furnished a ~1:7 α/*β* mix of tosylate **S12** as a white solid (1.0 g, 2.06 mmol, 42%). The *β*-isomer had R_f 0.55 (Tol/EtOAc 4:1). ^1H NMR (400 MHz, $CDCl_3$) δ 7.77 (d, 2H, J = 8.4 Hz, $H_{Ar,Ts}$), 7.35 (d, 2H, J = 8.0 Hz, $H_{Ar,Ts}$), 5.50 (d, 1H, $J_{1,2}$ = 8.4 Hz, H-1), 5.07-4.97 (m, 2H, H-3, H-4), 4.17-4.08 (m, 2H, H-6a, H-6b), 3.80 (m, 1H, H-5), 3.60 (m, 1H, H-2), 2.46 (s, 3H, CH_{3Ts}), 2.18, 2.09, 1.97 (3s, 9H, CH_{3Ac}). $^{13}C\{^1H\}$ NMR (100 MHz, $CDCl_3$) δ 169.78, 169.33, 168.30 (3C, CO_{Ac}), 145.10, 132.45 (2C, $C_{q,Ar}$), 129.84, 128.13 (C_{Ar}), 92.39 (C-1), 72.60 (C-4), 72.11 (C-5), 67.86 (C-3), 66.55 (C-6), 62.38 (C-2), 21.63 (C_{Me}), 20.74, 20.56, 20.40 (3C, C_{Ac}). HRMS (ESI^+): m/z $[M+NH_4]^+$ calc. for $C_{19}H_{27}N_4O_{10}S$ 503.1448; found 503.1444.

The *α*-isomer had R_f 0.5 (Tol/EtOAc 4:1). ^1H NMR (400 MHz, $CDCl_3$) δ 7.77 (d, J = 8.4 Hz, $H_{Ar,Ts}$), 7.35 (d, J = 8.0 Hz, $H_{Ar,Ts}$), 6.21 (d, $J_{1,2}$ = 3.6 Hz, H-1), 5.41 (dd, $J_{4,3}$ = 9.8 Hz, $J_{2,3}$ = 9.6 Hz, H-3), 5.07-4.97 (m, H-4), 4.17-4.08 (m, H-6a, H-6b, H-5), 3.63-3.57 (m, H-2), 2.46 (s, CH_{3Ts}), 2.17, 2.10, 2.01 (3s, CH_{3Ac}). $^{13}C\{^1H\}$ NMR (100 MHz, $CDCl_3$) δ 170.0, 169.3, 168.3 (3C, CO_{Ac}), 145.1 132.4 (2C, $C_{q,Ar}$), 129.8, 128.1 (C_{Ar}), 89.63 (C-1), 70.63 (C-3), 69.51 (C-5),

68.02 (C-4), 66.91 (C-6), 60.14 (C-2), 21.6 (C_{Me}), 20.8, 20.5, 20.4 (3C, C_{Ac}). HRMS (ESI^+): *m/z* $[M+NH_4]^+$ calc. for $C_{19}H_{27}N_4O_{10}S$ 503.1448; found 503.1444.

1,3,4-Tri-*O*-acetyl-2-azido-2,6-dideoxy-6-iodo-*α/β*-D-glucopyranose (S13). Sodium iodide (502 mg, 3.35 mmol, 2.5 equiv.) was added to tosylate **S12** (650 mg, 1.34 mmol, 1.0 equiv.) in butanone (10 mL). After heating for 3 h at 80 °C, a TLC (Tol/EtOAc, 4:1) follow up showed that the reaction was completed. The mixture was concentrated to 1/4th of the original volume, the resulting suspension was diluted with DCM (20 mL) and water (20 mL) was added. The DCM part was separated, dried over Na_2SO_4 and the filtrate was concentrated to dryness. Flash chromatography (Tol/EtOAc 90:10→85:15) gave the target **S13** as a white solid (520 mg, 1.17 mmol, 88%). The iodide **S13**, isolated as a 1:5 *α/β* mixture, had R_f 0.65 (Tol/EtOAc 4:1). HRMS (ESI^+): *m/z* $[M+Na]^+$ calc. for $C_{12}H_{16}IN_3O_7Na$ 463.9925; found 463.9913. The *β*-isomer had ^1H NMR (400 MHz, $CDCl_3$) δ 5.61 (d, 1H, $J_{1,2}$ = 8.4 Hz, H-1), 5.11 (dd, 1H, $J_{2,3}$ = 10.2 Hz, $J_{4,3}$ = 9.1 Hz, H-3), 4.92 (t, 1H, $J_{4,5}$ = 9.2 Hz, H-4), 3.67 (dd, 1H, H-2), 3.51 (ddd, 1H, H-5), 3.33 (dd, 1H, H-6a), 3.17 (m, 1H, H-6b), 2.21, 2.09, 2.05 (3s, 9H, CH_{3Ac}). $^{13}C\{^1H\}$ NMR (100 MHz, $CDCl_3$) δ 169.74, 169.44, 168.44 (3C, CO_{Ac}), 92.26 (C-1, $^1J_{C,H}$ = 169 Hz), 73.19 (C-5), 72.40 (C-3), 72.11 (C-4), 62.62 (C-2), 20.83, 20.61, 20.59 (3C, C_{Ac}), 2.54 (C-6).

The *α*-isomer had ^1H NMR (400 MHz, $CDCl_3$) δ 6.30 (d, $J_{1,2}$ = 3.6 Hz, H-1), 5.41 (dd, 1H, $J_{4,3}$ = 10.2 Hz, $J_{2,3}$ = 9.2 Hz, H-3), 4.97 (t, 1H, J = 9.6 Hz, H-4), 3.79-3.74 (m, 1H, H-5), 3.79-3.65 (m, 1H, H-2), 3.35-3.30 (m, 1H, H-6a), 3.18-3.13 (m, 1H, H-6b), 2.19, 2.11, 2.08 (3s, 9H, CH_{3Ac}). $^{13}C\{^1H\}$ NMR (100 MHz, $CDCl_3$) δ 170.01, 169.36, 168.35 (3C, CO_{Ac}), 89.78 (C-1, $^1J_{C,H}$ = 180 Hz), 72.28 (C-4), 70.41 (C-3), 60.33 (C-2), 20.8, 20.6, 20.5 (3C, C_{Ac}), 3.43 (C-6).

1,3,4-Tri-*O*-acetyl-2-azido-2-deoxy-*α/β*-D-glucopyranose (14). *Route a*: Bu_3SnH (230 µL, 998 µmol, 1.1 equiv.) and Et_3B (1 M in Hexane, 91 µL, 91 µmol, 0.1 equiv.) were successively added to a solution of iodide **S13** (400 mg, 907 µmol, 1.0 equiv.) in anhyd. DCM (5 mL) cooled to 0 °C. After 30 min at this temperature and following a TLC analysis, more Bu_3SnH (42 µL, 181 µmol, 0.2 equiv.) was added. The reaction was left for another 30 min at 0 °C, at which point a TLC follow up (Tol/EtOAc 4:1) revealed completion. MeOH was added, and after 10 min, volatiles were evaporated. Flash chromatoghy (Tol/EtOAc 90:10→88:12) gave the 6-deoxy derivative **14** as a 1:5 *α/β* mixture (250 mg, 793 µmol, 87%). HRMS (ESI^+): *m/z* $[M+NH_4]^+$ calc. for $C_{19}H_{27}N_4O_{10}S$ 333.1407; found 333.1405.

Route b: Sodium azide (144 mg, 2.21 mmol, 8.0 equiv.) was dissolved in water (3 mL). DCM (6 mL) was added and the biphasic mixture was cooled to 0 °C. Trifluoromethane-sulfonic anhydride (69 µL, 415 µmol, 1.5 equiv.) was added dropwise. After stirring the reaction mixture for 2 h at 0 °C, the organic phase was separated and washed with satd aq. $NaHCO_3$. Amine hydrochloride **13** (90 mg, 277 µmol, 1.0 equiv.) was dissolved in MeOH and $NaHCO_3$ (23 mg, 277 µmol, 1.0 equiv.) was added. The reaction mixture was cooled to 0 °C and the DCM extract was immediately added to the solution. The reaction mixture was allowed to reach rt and stirred overnight at this temperature. Following a TLC analysis (Tol/EtOAc 1:1), the mixture was concentrated *in vacuo*. Flash chromatography (Tol/EtOAc 90:10→88:12) of the crude gave the **14*β*** as a colorless oil (45 mg, 143 µmol, 51%).

The *β* isomer had R_f 0.6 (Tol/EtOAc 4:1). ^1H NMR (400 MHz, $CDCl_3$) δ 5.53 (d, 1H, $J_{1,2}$ = 8.7 Hz, H-1), 5.04 (dd$_{po}$, 1H, $J_{2,3}$ = 10.1 Hz, $J_{3,4}$ = 9.4 Hz, H-3), 4.76 (t, 1H, $J_{4,5}$ = 9.6 Hz, H-4),

3.71-3.58 (m_{po}, 2H, H-5, H-2), 2.16, 2.07, 2.02 (3s, 9H, CH_{3Ac}), 1.21 (d, 3H, $J_{5,6}$ = 6.0 Hz, H-6). $^{13}C\{^1H\}$ NMR (100 MHz, $CDCl_3$) δ 169.7, 169.7, 168.5 (3C, CO_{Ac}), 92.2 (C-1, $^1J_{C,H}$ = 168 Hz), 73.0 (C-4), 72.6 (C-3), 70.9 (C-5), 62.9 (C-2), 20.8, 20.8, 20.5 (3C, C_{Ac}), 17.1 (C-6).

The α-isomer had R_f 0.6 (Tol/EtOAc 4:1). ^1H NMR (400 MHz, $CDCl_3$) δ 6.22 (d, $J_{1,2}$ = 3.6 Hz, H-1), 5.40 (dd, $J_{3,4}$ = 10.2 Hz, $J_{2,3}$ = 9.2 Hz, H-3), 4.80 (t, H-4), 3.96 (m, 1H, H-5), 3.71-3.58 (m, H-2), 2.16, 2.09, 2.05 (3s, CH_{3Ac}), 1.18 (d, $J_{5,6}$ = 6.4 Hz, H-6). $^{13}C\{^1H\}$ NMR (100 MHz, $CDCl_3$) δ 169.9, 169.6, 168.6 (3C, CO_{Ac}), 90.3 (C-1, $^1J_{C,H}$ = 180 Hz), 73.2 (C-4), 70.6 (C-3), 67.7 (C-5), 60.5 (C-2), 20.8, 20.8, 20.5 (3C, C_{Ac}), 17.2 (C-6).

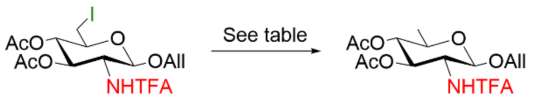

27 19

Entry	Scale (g)	Reducing agent (equiv.)	Conditions	19 (yield)[a]
1	0.25	$NaBH_3CN$ (3.0)	DMF, 100 °C, 16 h	64%
2[b]	2.0	$NaBH_3CN$ (3.0)	DMF, 100 °C, 24 h	73%
3	0.075	$(TMS)_3SiH$ (1.2)<br>Et_3B (0.2)	DCM, 0 °C→ rt, 2 h	46%
4	0.25	Bu_3SnH (1.5)<br>Et_3B (0.3)	Tol, 0 °C, 1 h	79%
5	2.0	Bu_3SnH (1.5)<br>Et_3B (0.3)	DCM, 0 °C, 1 h	76%
**6**	**43.5**	**Bu_3SnH (1.1)**<br>**Et_3B (0.1)**	**DCM, 0 °C, 1 h**	**96%**

[a] Reported yields are based on isolated products. [b] Recovered **27** (5%).

Scheme S6. C-6 reduction on diacetate **27** by means of hydrides (Entries 1-2) or radical-mediated conditions (Entries 3-6).

Allyl 3,4-di-*O*-acetyl-2,6-dideoxy-2-trifluoroacetamido-*β*-D-glucopyranoside (19). *Route a.* The 6-iodo derivative **27** (2.0 g, 3.92 mmol, 1.0 equiv.) was dissolved in anhyd. DMF (20 mL) and $NaBH_3CN$ (741 mg, 11.7 mmol, 3.0 equiv.) was added. The reaction mixture was heated at 100 °C and stirred for 24 h under an Ar atmosphere. After cooling to rt, water (150 mL) was added and the aq. phase was washed with DCM (50 mL) three times. The DCM parts were combined, washed with brine (100 mL), dried over Na_2SO_4 and concentrated *in vacuo*. Flash chromatography of the crude mixture gave diacetate **19** as a white solid (1.1 g, 2.87 mmol, 73%). Analytical data were as described in the main document.

Route b. Iodide **27** (75 mg, 135 µmol, 1.0 equiv.) was dissolved in anhyd. DCM (4 mL) and cooled to 0 °C. TMS_3SiH (50 µL, 162 µmol, 1.2 equiv.) followed by Et_3B (1 M in hexane, 27 µL, 27 µmol, 0.2 equiv.) was added. The reaction mixture was stirred for 2 h under an Ar atmosphere while slowly allowed to reach rt. MeOH (~0.2 mL) was added and volatiles were

eliminated under vacuum. Flash chromatography of the crude gave **19** as a white solid (26 mg, 67 µmol, 46%). Analytical data of **19** were as described in the main document.

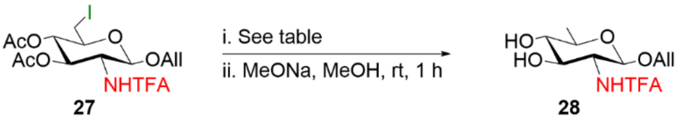

Entry	Scale (g)	Reducing agent (equiv.)	Conditions	Yield (%)
1	4.5	$NaBH_3CN$ (3.0)	DMF, 100 °C, 26 h	73
2	2.0	Bu_3SnH (1.5), Et_3B (0.2)	Tol, 0 °C, 1 h	81
3	8.0	Bu_3SnH (1.5), Et_3B (0.2)	DCM, 0 °C, 1 h	85
**4**	**20.0**	**Bu_3SnH (1.2), Et_3B (0.2)**	**DCM, 0 °C → rt, 1 h**	**97**
**5**	**20.0**	**Bu_3SnH (1.2), Et_3B (0.2)**	**DCM, 0 °C → rt, 1 h**	**92**
**6**	**22.0**	**Bu_3SnH (1.2), Et_3B (0.2)**	**DCM, 0 °C → rt, 1 h**	**90**

[a] Reported yields are based on isolated products over two steps.

Scheme S7. Two-step conversion of diacetate **27** into the 6-deoxy diol **28**.

Allyl 2,6-dideoxy-2-trifluoroacetamido-*β*-D-glucopyranoside (28). $NaCNBH_3$ (1.66 g, 26.5 mmol, 3.0 equiv.) was added to a solution of the 6-iodo precursor **27** (4.5 g, 8.8 mmol, 1.0 equiv.) in anhyd. DMF (40 mL) under an Ar atmosphere. The reaction mixture was heated at 100 °C for 26 h keeping the reaction under an Ar atmosphere. Following a TLC analysis (Tol/EtOAc 4:1) indicating completion, the reaction mixture was cooled to rt and water (400 mL) was added. The aq. phase was washed with DCM (3×60 mL). The organic phases were combined, washed with brine (100 mL), dried over Na_2SO_4, and concentrated under reduced pressure. The crude product was dissolved in MeOH (40 mL) and 25% NaOMe in MeOH (396 µL, 0.2 equiv.) was added at rt. After 2 h, more NaOMe (0.2 equiv.) was added. After another 2 h, a TLC (Tol/EtOAc 1:1) follow up showed reaction completion. Dowex-H^+ resin was added portionwise under gentle stirring until neutral pH was reached. The suspension was filtered over a bed of Celite® and solids were washed thoroughly with MeOH. The filtrate was concentrated and the crude was purified by flash chromatography (Tol/EtOAc 55:45→45:55) to give diol **28** (1.95 g, 5.08 mmol, 73%) as a white solid. Analytical data were as described in the main document.

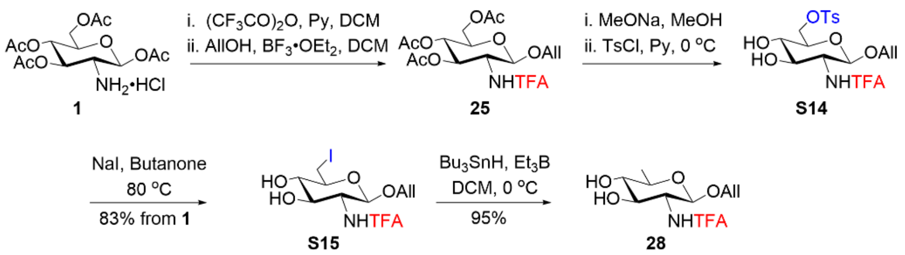

Scheme S8. Large scale synthesis of QuiN **28** from glucosamine **1**.

Allyl 2,6-dideoxy-6-*O*-tosyl-2-trifluoroacetamido-*β*-D-glucopyranoside (S14). Anhyd. pyridine (37.3 mL, 461 mmol, 3.0 equiv.) was added to a suspension of commercially available **1** (59.0 g, 153 mmol, 1.0 equiv.) in DCM (512 mL). The suspension was cooled to 0 °C. Trifluoroacetic anhydride (27.7 mL, 199 mmol, 1.3 equiv.) was added slowly and the mixture was stirred for 1 h while the bath temperature was allowed to reach rt. Follow up by TLC (Tol/EtOAc 6:4) indicated the absence of starting material and the presence of a less polar product (R_f 0.55). MeOH (20 mL) was added and the reaction mixture was stirred at rt for an additional 20 min and diluted with DCM (250 mL). The organic layer was washed with 1 N aq. HCl (600 mL) and satd aq. $NaHCO_3$ (600 mL), dried over Na_2SO_4, filtered, and concentrated *in vacuo*. The crude solid was dried extensively to give trifluoroacetamide **24** (68.0 g) as a yellow solid.

The crude **24** (67.0 g, estimated 151 mmol, 1.0 equiv.) and allyl alcohol (30.8 mL, 453 mmol, 3.0 eq.) in anhyd. DCM (600 mL) were stirred with freshly activated MS 4 Å (30 g) for 1 h under an Ar atmosphere. The suspension was cooled to 0 °C and boron trifluoride diethyl etherate (BF_3·OEt_2, 41.0 mL, 332 mmol, 2.2 equiv.) was slowly added. The bath was allowed to reach rt and the reaction mixture was stirred for 65 h at rt. A TLC (DCM/EtOAc 9:1) follow up indicated conversion of the starting material (R_f 0.25) into a less polar compound (R_f 0.35). The suspension was filtered over a Celite® bed and washed with DCM (3×50 mL). The organic layer was washed with H_2O (600 mL) and satd aq. $NaHCO_3$ (600 mL), dried over Na_2SO_4, filtered, and concentrated to dryness. The crude allyl glycoside **25** (66.0 g) was obtained as a yellowish solid.

NaOMe (25% in MeOH, 9.4 mL, 43 mmol, 0.3 equiv.) was added to the crude allyl glycoside **25** (64.0 g, estimated 145 mmol, 1.0 equiv) in MeOH (900 mL). After stirring at rt for 2 h, a follow up by TLC (Tol/EtOAc 7:3) indicated the total conversion of the starting material (R_f 0.25) into a more polar product. Dowex-H^+ resin was added slowly under gentle stirring until pH 7.0. The suspension was filtered and the resin was washed with MeOH thoroughly. Solvents were evaporated and the crude material was dried under high vacuum. The crude residue dissolved in anhyd. pyridine (725 mL) was cooled to 0 °C. Tosyl chloride (69.1 g, 362 mmol, 2.5 equiv.) was added and the reaction mixture was stirred for 2.5 h at 0 °C. More MeOH (50 mL) was added and after another 30 min, volatiles were removed under vacuum. The crude was dissolved in DCM (600 mL) and washed with 1 N aq. HCl (500 mL) and 50% aq. $NaHCO_3$ (600 mL). The organic phase was dried over Na_2SO_4 and filtered over a fitted funnel. Volatiles were evaporated and co-evaporated with toluene (500 mL) to give the crude product (68 g) as an off-white solid containing a 9:1 mix of the desired **S14** and of di-*O*-tosyl side-products as seen in the ^1H NMR spectrum. The 6-*O*-tosyl **S14** had ^1H NMR (400

MHz, CD_3CN) δ 7.83 (d, 2H, J = 8.4 Hz, $H_{Ar,Ts}$), 7.54 (d, 1H, $J_{NH,2}$ = 9.6 Hz, NH), 7.47 (dd, 2H, J = 8.1, Hz, $H_{Ar,Ts}$), 5.90-5.81 (m, 1H, CH_{All}), 5.26-5.20 (m, 1H, CH_{2All}), 5.18-5.15 (m, 1H, CH_{2All}), 4.49 (d, 1H, $J_{1,2}$ = 8.4 Hz, H-1), 4.32 (dd, 1H, $J_{5,6a}$ = 2.0 Hz, $J_{6a,6b}$ = 11.0 Hz, H-6a), 4.20-4.15 (m, 2H, H-6b, CH_{2All}), 4.00-3.94 (m, 1H, CH_{2All}), 3.68 (d, 1H, $J_{3,OH}$ = 5.1 Hz, OH), 3.62 (ddd, 1H, $J_{2,3}$ = 9.7 Hz, H-2), 3.57 (d, 1H, $J_{4,OH}$ = 4.9 Hz, OH), 3.52-3.45 (m, 2H, H-3, H-5), 3.28 (ddd, 1H, H-4), 2.47 (s, 3H, CH_{3Ts}). $^{13}C\{^1H\}$ NMR (100 MHz, CD_3CN) δ 157.16 (CO_{NHTFA}, q, $J_{C,F}$ = 36.0 Hz), 145.48 ($C_{q,Ar}$), 134.04 (CH_{All}), 132.76 ($C_{q,Ar}$), 130.03 (C_{Ar}), 127.83 ($C_{Ar,Ts}$), 116.94 (CF_3, q, $J_{C,F}$ = 283.7 Hz), 116.34 (CH_{2All}), 99.47 (C-1, $^1J_{C,H}$ = 161.6 Hz), 73.34 (C-3, C-5), 70.05 (C-4), 69.53, 59.52 (2C, C-6, CH_{2All}), 56.10 (C-2), 20.68 (C_{Me}). HRMS (ESI^+): *m/z* $[M+NH_4]^+$ calcd for $C_{18}H_{26}F_3N_2O_8S$ 487.1356; found 487.1351.

Allyl 2,6-dideoxy-6-iodo-2-trifluoroacetamido-*β*-D-glucopyranoside (S15). Sodium iodide (65.1 g, 434 mmol, 3.0 equiv.) was added to a solution of the crude **S14** (68.0 g, estimated 144 mmol, 1.0 equiv., *see above*) in butanone (720 mL). The reaction mixture was heated to reflux for 24 h. After reaching rt, the reaction mixture was concentrated under vacuum. The crude was dissolved in EtOAc (600 mL) and washed with water (500 mL). The organic phase was dried over Na_2SO_4 and concentrated. The residue was washed with DCM (2×250 mL) and the obtained off-white solid was collected and dried extensively to give the targeted 6-iodo derivative **S15** (50 g, 117 mmol, 83% over five steps). Diol **S15** had ^1H NMR (400 MHz, CD_3CN) δ 7.61 (d, 1H, $J_{NH,2}$ = 9.1 Hz, NH), 5.96-5.86 (m, 1H, CH_{All}), 5.31-5.25 (m, 1H, CH_{2All}), 5.21-5.17 (m, 1H, CH_{2All}), 4.58 (d, 1H, $J_{1,2}$ = 8.4 Hz, H-1), 4.33-4.28 (m, 1H, CH_{2All}), 4.13-4.08 (m, 1H, CH_{2All}), 3.72-3.65 (m, 3H, H-2, 2OH), 3.62 (dd, 1H, $J_{5,6a}$ = 2.7 Hz, $J_{6a,6b}$ = 11.0 Hz, H-6a), 3.55 (m, 1H, H-3), 3.36 (dd, 1H, $J_{5,6b}$ = 7.1 Hz, H-6b), 3.24 (m, 1H, H-4), 3.16 (m, 1H, H-5). $^{13}C\{^1H\}$ NMR (100 MHz, CD_3CN) δ 157.21 (CO_{NHTFA}, q, $J_{C,F}$ = 36.7 Hz), 134.05 (CH_{All}), 116.64 (CH_{2All}), 116.13 (CF_3, q, $J_{C,F}$= 287.0 Hz), 99.30 (C-1, $^1J_{C,H}$ = 161.2 Hz), 74.62 (C-5), 74.27 (C-4), 73.13 (C-3), 69.63 (CH_{2All}), 56.41 (C-2), 6.53 (C-6). HRMS (ESI^+): *m/z* $[M+NH_4]^+$ calcd for $C_{11}H_{19}F_3IN_2O_5$ 443.0285; found 443.0281.

3. Supplementary figure showing S16-S22

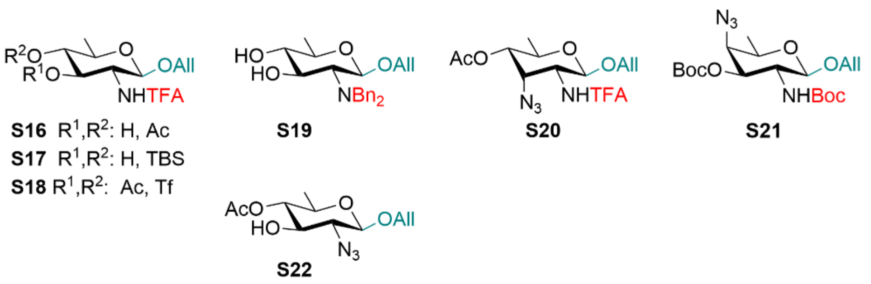

Figure S1. Additional side-products characterized upon protecting group manipulation and described in the main experimental.

4. Experimental and analytical data for side-products S16-S22

Allyl 4-*O*-acetyl-2,6-dideoxy-2-trifluoroacetamido-*β*-D-glucopyranoside (S16). *Route a.* A solution of diol **28** (11.5 g, 38.4 mmol, 1.0 equiv.) in anhyd. THF (350 mL) was cooled to -78 °C. Anhyd. pyridine (6.2 mL, 76.9 mmol, 2.0 equiv.) was added followed by acetyl chloride (3.0 mL, 42.3 mmol, 1.1 equiv.). The reaction mixture was allowed to reach rt over 8 h and was stirred for another 16 h. A follow up by TLC (Tol/EtOAc 7:3) indicated the conversion of the starting material into a less polar product (R_f 0.25). MeOH (3.0 mL) was added and after stirring at rt for 30 min, solvents were evaporated. Flash chromatography (Tol/EtOAc 65:35→55:45) gave by order of elution diacetate **19** (600 mg, 1.56 mmol, 4.0%) as a white solid and the 3-*O*-acetyl derivative **32** (11.3 g, 32.2 mmol, 86%) as a white solid, albeit as a 25:1 mix with the 4-*O*-acetate **S16**.

Scale up. A solution of diol **28** (33.5 g, 112 mmol, 1.0 equiv.) in anhyd. THF (560 mL) was cooled to -78 °C. Anhyd. pyridine (18.1 mL, 224 mmol, 2.0 equiv.) was added followed by the addition of acetyl chloride (8.7 mL, 123 mmol, 1.1 equiv.). The reaction mixture was allowed to reach rt over 8 h and was stirred overnight at this temperature. A follow up by TLC (Tol/EtOAc 1:1) indicated conversion of the starting **28** into a less polar product (R_f 0.35). MeOH (15 mL) was added and after stirring at rt for another 30 min, volatiles were evaporated. Flash chromatography using Tol/EtOAc (65:35→55:45) gave first the undesired diacetate **19** (3.6 g, 9.39 mmol, 8.3%) and then a non-separable >15:1 mix of the desired **32** and its regioisomer **S16** (30.0 g, 87.9 mol, 79%), both as a white solid. Regioisomer **S16** had R_f 0.5 (Tol/EtOAc 1:1). ^{1}H NMR (400 MHz, $CDCl_3$, extracted from the mixture) δ 6.82 (d, $J_{NH,2}$ = 8.8 Hz, NH), 5.88-5.80 (m, CH_{All}), 5.31-5.26 (m, H-4, CH_{2All}), 5.25-5.22 (m, CH_{2All}), 4.72 (d, $J_{1,2}$ = 8.4 Hz, H-1), 4.67 (dd, $J_{2,3}$ = $J_{3,4}$ = 9.3 Hz, H-3), 4.38-4.32 (m, CH_{2All}), 4.10-4.04 (m, CH_{2All}), 3.77-3.63 (dt, 1H, H-2), 3.60-3.53 (m, H-5), 3.40 (OH), 2.13 (s, CH_{3Ac}-4), 1.27 (d, 3H, $J_{5,6}$ = 5.6 Hz, H-6). $^{13}C\{^1H\}$ NMR (100 MHz, $CDCl_3$, extracted from the mixture) δ 171.0 (CO_{Ac}), 157.2 (CO_{NHTFA}, q, $J_{C,F}$ = 37.7 Hz), 133.1 (CH_{All}), 117.8 (CH_{2All}), 117.0 (CF_3, q, $J_{C,F}$ = 288.6 Hz), 98.3 (C-1, $^1J_{C,H}$ = 160.2 Hz), 76.5 (C-3), 71.3 (C-4), 70.0 (C-5), 70.1 (CH_{2All}), 58.5 (C-2), 20.8 (C, CH_{3Ac}), 17.4 (C-6). HRMS (ESI$^+$): *m/z* $[M+H]^+$ calcd for $C_{13}H_{19}F_3NO_6$ 342.1165; found 342.1181.

Route b. DIPEA (20 µL, 114 µmol, 0.2 equiv.) and acetic anhydride (54 µL, 574 µmol, 1.01 equiv.) were added to diol **28** (170 mg, 568 µmol, 1.0 equiv.) dissolved in dry MeCN (6.0 mL) and stirred vigorously for 3 h at 100 °C under an Ar atmosphere. The resulting mixture was allowed to reach rt and volatiles were evaporated. Flash chromatography of the crude gave the 3-*O*-acetyl derivative **32** and its regioisomer **S16** as a non-separable 5:1 mixture (164 mg, 480 µmol, 84%). Analytical data were as above.

Allyl 4-*O*-*tert*-butyldimethylsilyl-2,6-dideoxy-2-trifluoroacetamido-*β*-D-glucopyranoside (S17). 2,6-Lutidine (138 µL, 1.44 mmol, 1.2 equiv.) and TBSOTf (304 µL, 1.32 mmol, 1.1 equiv.) were added to a solution of diol **28** (360 mg, 669 µmol, 1.0 equiv.) in anhyd. THF (12 mL) at -78 °C. The reaction was allowed to attain rt slowly over 6 h. MeOH was added and volatiles were evaporated. Flash chromatography (Tol/EtOAc 90:10→80:20) gave by order of elution the 4-*O*-silylated analog **S17** (220 mg, 532 µmol, 44%) and the desired **30**, both as a white solid (240 mg, 580 µmol, 48%). Regioisomer **S17** had R_f 0.6 (Tol/EtOAc

4:1). ^{1}H NMR (400 MHz, $CDCl_3$) δ 6.57 (brs, 1H, NH), 5.92-5.83 (m, 1H, CH_{2All}), 5.31-5.26 (m, 1H, CH_{2All}), 5.25-5.22 (m, 1H, CH_{2All}), 4.68 (d, 1H, $J_{1,2}$ = 8.0 Hz, H-1), 4.38-4.33 (m, 1H, CH_{2All}), 4.10-4.05 (m, 1H, CH_{2All}), 3.85 (ddd, 1H, $J_{2,3}$ = 10.0 Hz, H-3), 3.54 (dt, 1H, H-2), 3.49-3.39 (m, 2H, H-4, H-5), 3.29 (t, 1H, $J_{3,4}$ = $J_{4,5}$ = 8.1 Hz, H-4), 2.54 (d, 1H, $J_{4,OH}$ = 4.4 Hz, OH), 1.33 (d, 3H, $J_{5,6}$ = 6.0 Hz, H-6), 0.92 (s. 9H, $CH_{3,TBS}$), 0.16 (s, 3H, $CH_{3,TBS}$), 0.14 (s, 3H, $CH_{3,TBS}$). ^{13}C{^{1}H} NMR ($CDCl_3$) δ 157.58 (CO_{NHTFA}, q, $J_{C,F}$ = 34.1 Hz), 133.31 (CH_{All}), 118.18 (CH_{2All}), 115.71 (CF_3, q, $J_{C,F}$ = 288.0 Hz), 98.29 (C-1, $^1J_{C,H}$ = 161 Hz), 77.39 (C-4), 73.45 (C-3), 72.73 (C-5), 69.88 (CH_{2All}), 58.15 (C-2), 25.87 (C_{TBS}), 18.26 (C-6), 18.17 ($C_{q,TBs}$), -3.82, -4.57 (2C, $CH_{3,TBS}$). HRMS (ESI$^+$): *m/z* [M+NH_4]$^+$ calcd for $C_{17}H_{34}F_3N_2O_5Si$ 431.2189; found 431.2177.

Allyl 2-*N,N*-dibenzyl-2,6-dideoxy-*β*-D-glucopyranoside (S19). NaH (60% in mineral oil, 32 mg, 669 µmol, 1.0 equiv.) was added portionwise to a solution of diol **28** (200 mg, 0.67 mmol, 1.0 equiv.) and benzyl bromide (87 µL, 0.74 mmol, 1.1 equiv.) in anhyd. DMF (5.0 mL) under vigorous stirring at 0 °C. After 3 h at 0 °C, 0.5 M aq. NH_4Cl (10 mL) was added and the aqueous phase was extracted with EtOAc (20 mL). The organic phases were pooled and washed with brine, dried over Na_2SO_4, and concentrated. Flash chromatography (Tol/EtOAc 65:35→40:60) gave by order of elution the *N,N*-dibenzylated side-product **S19** (12 mg, 31 µmol, 4.6%), the 4-*O*-benzyl ether **31** (110 mg, 282 µmol, 42%) as a white solid, and the unreacted **28** (95 mg, 317 µmol, 47%). The side-product **S19** had R_f 0.4 (Tol/EtOAc 1:1). ^{1}H NMR (400 MHz, $CDCl_3$) δ 7.39-7.25 (m, 10H, H_{Ar}), 6.17-6.07 (m, 1H, CH_{All}), 5.49-5.44 (m, 1H, CH_{2All}), 5.35-5.32 (m, 1H, CH_{2All}), 4.72 (d, 1H, $J_{1,2}$ = 8.4 Hz, H-1), 4.55-4.50 (m, 1H, CH_{2All}), 4.21-4.16 (m, 1H, CH_{2All}), 3.95 (d, 2H, J = 12.8 Hz, CH_{2Bn}), 3.80 (d, 2H, CH_{2Bn}), 3.49 (dd, 1H, $J_{3,4}$ = 8.2 Hz, $J_{2,3}$ = 10.2 Hz, H-3), 3.36-3.29 (m, 1H, H-5), 3.26 (s, 1H, OH), 3.08 (t, 1H, $J_{4,5}$ = 8.8 Hz, H-4), 2.65 (brs, 1H, OH), 2.60 (dd, 1H, H-2), 1.30 (d, 3H, $J_{5,6}$ = 6.4 Hz, H-6). ^{13}C{^{1}H} NMR (100 MHz, $CDCl_3$) δ 139.16 ($C_{q,Ar}$), 134.05 (CH_{All}), 129.30, 128.55, 127.38 (C_{Ar}), 117.71 (CH_{2All}), 100.76 (C-1, $^1J_{C,H}$ = 156.6 Hz), 76.48 (C-4), 71.63 (2C, C-3, C-5), 69.83 (CH_{2All}), 62.57 (C-2), 54.52 (2C, CH_{2NBn}), 17.50 (C-6). HRMS (ESI$^+$): *m/z* [M+H]$^+$ calcd for $C_{23}H_{30}NO_4$ 384.2169; found 384.2156.

Allyl 3-*O*-acetyl-2,6-dideoxy-2-trifluoroacetamido-4-*O*-trifluoromethanesulfonyl-*β*-D-glucopyranoside (S18) and allyl 4-*O*-acetyl-3-azido-2,3,6-trideoxy-2-trifluoroacetamido-*β*-D-allopyranoside (S20). Alcohol **32** (10.3 g, 30.1 mmol, 1.0 equiv.), containing traces of the 4-*O*-acetyl isomer (**S16**), was dissolved in anhyd. DCM (280 mL) under an Ar atmosphere. The solution was cooled to -10 °C. Triflic anhydride (6.5 mL, 39 mmol, 1.3 equiv.) was added followed by addition of anhyd. pyridine (4.84 mL, 60 mmol, 2.0 equiv.). The solution was stirred for 1 h at -10 °C. Follow up by TLC (Tol/EtOAc 2:1) indicated the absence of the starting material (R_f 0.25) and the presence of a less polar product (R_f 0.8). The reaction mixture was diluted with DCM (100 mL). The organic layer was washed with 1 N aq. HCl (200 mL) and 0.5 M aq. $NaHCO_3$ (200 mL), dried over Na_2SO_4, filtered and concentrated *in vacuo*. The crude was dried under high vacuum for 2 h. The intermediate allyl 3-*O*-acetyl-2,6-dideoxy-2-trifluoroacetamido-4-*O*-trifluoromethanesulfonyl-*β*-D-glucopyranoside (**S18**) had ^{1}H NMR (400 MHz, $CDCl_3$) δ 7.00 (d, 1H, $J_{NH,2}$ = 8.8 Hz, NH), 5.87-5.79 (m, 1H, CH_{All}), 5.45 (dd, 1H, $J_{2,3}$ = 10.8 Hz, H-3), 5.30-5.21 (m, 2H, CH_{2All}), 4.67 (t, 1H, $J_{3,4}$ = $J_{4,5}$ = 9.6 Hz, H-4), 4.61 (d, 1H, $J_{1,2}$ = 8.1 Hz, H-1), 4.37-4.31 (m, 1H, CH_{2All}), 4.20-4.13 (m, 1H, H-2), 4.11-4.05 (m, 1H, CH_{2All}), 3.72-3.68 (m, 1H, H-5), 2.14 (s, 3H, CH_{3Ac}), 1.43 (d, 3H, $J_{5,6}$ = 6.0 Hz, H-6). ^{13}C{^{1}H}

NMR (100 MHz, $CDCl_3$) δ 171.61 (CO_{Ac}), 157.49 (CO_{NHTFA}, q, $J_{C,F}$ = 38.5 Hz), 133.82 (CH_{All}), 118.41 (CF_3, q, $J_{C,F}$ = 286.6 Hz), 118.33 (CH_{2All}), 98.86 (C-1, $^1J_{C,H}$ = 161.0 Hz), 84.79 (C-4), 71.36 (C-3), 70.24 (CH_{2All}), 69.28 (C-5), 55.03 (C-2), 20.35 (CH_{3Ac}), 17.19 (C-6). HRMS (ESI^+): *m/z* $[M+NH_4]^+$ calcd for $C_{14}H_{21}F_6N_2O_8S$ 491.0917; found 491.0917.

The residue was dissolved in anhyd. DMF (100 mL) and sodium azide (9.8 g, 150 mmol, 5.0 equiv.) was added. After stirring at rt overnight, TLC (Tol/EtOAc 4:1) showed the total conversion of the intermediate triflate (R_f 0.45) and the presence of a more polar product (R_f 0.35). The reaction mixture was diluted with DCM (200 mL) and washed with H_2O (300 mL) and brine (300 mL). The organic phase was dried over Na_2SO_4, filtered, and concentrated under reduced pressure. Flash chromatography (Tol/EtOAc 80:20→75:25) gave by order of elution the unwanted **S20** (100 mg, 273 µmol, 1%) and the azido derivative **34** (9.1 g, 82%), both as a white solid. The side-product **S20** had R_f 0.75 (Tol/EtOAc 7:3). ^{1}H NMR (400 MHz, $CDCl_3$) δ 6.49 (d, 1H, $J_{NH,2}$ = 8.5 Hz, NH), 5.89-4.80 (m, 1H, CH_{All}), 5.30-5.20 (m, 2H, CH_{2All}), 4.83 (dd, 1H, $J_{4,5}$ = 9.1 Hz, $J_{3,4}$ = 3.2 Hz, H-4), 4.58 (d, 1H, $J_{1,2}$ = 7.8 Hz, H-1), 4.39 (t, 1H, $J_{3,2}$ = $J_{3,4}$ = 3.2 Hz, H-3), 4.3-4.30 (m, 1H, CH_{2All}), 4.15 (dt, 1H, H-2), 4.06-3.99 (m, 2H, CH_{2All}, H-5), 2.16 (s, 3H, CH_{3Ac}), 1.29 (d, 3H, $J_{5,6}$ = 6.3 Hz, H-6). $^{13}C\{^1H\}$ NMR (100 MHz, $CDCl_3$) δ 169.39 (CO_{Ac}), 156.71 (CO_{NHTFA}, q, $J_{C,F}$ = 37 Hz), 133.16 (CH_{All}), 117.93 (CH_{2All}), 115.63 (CF_3, q, $J_{C,F}$ = 288 Hz), 97.39 (C-1, $^1J_{C,H}$ = 161 Hz), 73.49 (C-4), 69.71 (CH_{2All}), 68.57 (C-5), 60.38 (C-3), 51.25 (C-2), 20.38 (CH_{3Ac}), 17.48 (C-6). HRMS (ESI^+): *m/z* $[M+NH_4]^+$ calcd for $C_{13}H_{21}F_3N_5O_5$ 384.1489; found 384.1475.

Allyl 4-azido-2-*N*,3-*O*-di-*tert*-butyloxycarbonyl-2,4,6-trideoxy-*β*-D-galactopyranoside (S21). DMAP (10.7 mg, 88 µmol, 0.1 equiv.) and *tert*-butyl dicarbonate (Boc_2O, 287 mg, 13.1 mmol, 1.5 equiv.) were added to a solution of amine **39** (200 mg, 877 µmol, 1.0 equiv.) in anhyd. THF (6.0 mL). After heating at 50 °C for 2 h, a follow up by TLC (Tol/EtOAc 7:3) indicated completion.The reaction mixture was cooled to rt and concentrated under reduced pressure. The residue was purified by flash chromatography (cHex/EtOAc 70:30→60:40) to give by order of elution the fully protected **S21** (16 mg, 37 µmol, 4%) and the desired alcohol **40** (185 mg, 5.63 mmol, 83%), both as a white solid. The side-product **S21** had R_f 0.75 (Tol/EtOAc 7:3). ^{1}H NMR (400 MHz, $CDCl_3$) δ 5.91-5.82 (m, 1H, CH_{2All}), 5.30-5.24 (m, 2H, H-3, CH_{2All}), 5.18-5.15 (m, 1H, CH_{2All}), 4.76 (brs, 2H, H-1, NH), 4.35-4.29 (m, 1H, CH_{2All}), 4.10-4.05 (m, 1H, CH_{2All}), 3.88 (brd, 1H, $J_{3,4}$ = 3.6 Hz, H-4), 3.72 (dq, 1H, H-5), 3.44 (brs, 1H, H-2), 1.49 (brs, 9H, CH_3), 1.42 (brs, 9H, CH_3), 1.37 (d, 3H, $J_{5,6}$ = 6.0 Hz, H-6). $^{13}C\{^1H\}$ NMR (100 MHz, $CDCl_3$) δ 155.03 (CO_{NHBoc}), 152.79 (CO_{Boc}), 133.90 (CH_{All}), 117.40 (CH_{2All}), 99.19 (C-1), 83.11 ($C_{q,Bu}$), 79.74 ($C_{q,Bu}$), 73.98 (C-3), 69.79 (CH_{2All}), 68.94 (C-5), 63.73 (C-4), 52.91 (C-2), 28.23 ($CH_{3,Bu}$), 27.63 ($CH_{3,Bu}$), 17.41 (C-6). HRMS (ESI^+): *m/z* $[M+Na]^+$ calcd for $C_{19}H_{32}N_4O_7Na$ 451.2163; found 451.2160.

Allyl 4-*O*-acetyl-2-azido-2,6-dideoxy-*β*-D-glucopyranoside (S22). Acetyl chloride (102 µL, 14.4 mmol, 1.1 equiv.) was added dropwise to a solution of the diol **47** (300 mg, 1.30 mmol, 1.0 equiv.) and pyridine (210 µL, 26.1 mmol, 2.0 equiv.) in anhyd. THF (15 mL) at -78 °C. The reaction mixture slowly reached rt. After 16 h, a TLC analysis (Tol/EtOAc 4:1) indicated reaction completion. MeOH (0.1 mL) was added and after 10 min, volatiles were evaporated. Flash chromatography (Tol/EtOAc 84:16→80:20) gave the 3-*O*-acetylated product **51** (240 mg, 0.88 mmol, 67%) along with the regioisomer **S22** (65 mg, 0.23 mmol, 18%). Regioisomer **S22** had R_f 0.6 (Tol/EtOAc 4:1). ^{1}H NMR (400 MHz, $CDCl_3$) δ 5.99-5.90

(m, 1H, CH_{2All}), 5.36-5.32 (m, 2H, CH_{2All}), 4.65 (t, 1H, $J_{3,4} = J_{4,5} = 9.6$ Hz, H-4), 4.41-4.36 (m, 1H, CH_{2All}), 4.35 (d, 1H, $J_{1,2} = 8.0$ Hz, H-1), 4.16-4.11 (m, 1H, CH_{2All}), 3.51-3.37 (m, 3H, H-2, H-3, H-5), 2.93 (d, 1H, $J_{3,OH} = 4.0$ Hz, OH), 2.11 (s, 3H, CH_{3Ac}), 1.22 (d, 3H, $J_{5,6} = 6.0$ Hz, H-6). $^{13}C\{^1H\}$ NMR (100 MHz, $CDCl_3$) δ 170.94 (CO_{Ac}), 133.35 (CH_{All}), 117.84 (CH_{2All}), 100.61 (C-1), 75.73 (C-4), 73.28 (C-3), 70.32 (CH_{2All}), 69.94 (C-5), 66.67 (C-2), 20.86 (CH_{3Ac}), 17.35 (C-6). HRMS (ESI^+): *m/z* $[M+NH_4]^+$ calcd for $C_{11}H_{21}N_4O_5$ 289.1506; found 289.1504.

5. References

(1) Medgyes, A.; Farkas, E.; Lipták, A.; Pozsgay, V. Synthesis of the monosaccharide units of the O-specific polysaccharide of *Shigella sonnei*. *Tetrahedron* **1997**, *53*, 4159-4178.

(2) Guiard, J.; Rahali, Y.; Praly, J.-P. $NaBH_3CN$: A Janus Substitute for Tin-Free Radical-Based Reactions. *Eur. J. Org. Chem.* **2014**, *2014*, 4461-4466.

(3) Boutet, J.; Kim, T. H.; Guerreiro, C.; Mulard, L. A. Efficient route to orthogonally protected precursors of 2-acylamino-2-deoxy-3-O-substituted-beta-D-glucopyranose derivatives and use thereof. *Tetrahedron Lett.* **2008**, *49*, 5339-5342.

(4) Zhang, X.; Zhou, Y.; Zuo, J.; Yu, B. Total synthesis of periploside A, a unique pregnane hexasaccharide with potent immunosuppressive effects. *Nat. Commun.* **2015**, *6*, 5879.

(5) Lloyd, D.; Bennett, C. S. Gram-Scale Synthesis of an Armed Colitose Thioglycoside. *J. Org. Chem.* **2014**, *79*, 9826-9829.

(6) Cheng, G.-J.; Drosos, N.; Morandi, B.; Thiel, W. Computational Study of $B(C_6F_5)_3$-Catalyzed Selective Deoxygenation of 1,2-Diols: Cyclic and Noncyclic Pathways. *ACS Catal.* **2018**, *8*, 1697-1702.

(7) Pfister, H. B.; Mulard, L. A. Synthesis of the Zwitterionic Repeating Unit of the O-Antigen from *Shigella sonnei* and Chain Elongation at Both Ends. *Org. Lett.* **2014**, *16*, 4892-4895.

(8) Myszka, H.; Bednarczyk, D.; Najder, M.; Kaca, W. Synthesis and induction of apoptosis in B cell chronic leukemia by diosgenyl 2-amino-2-deoxy-*β*-D-glucopyranoside hydrochloride and its derivatives. *Carbohydr. Res.* **2003**, *338*, 133-141.

(9) Yan, R. B.; Yang, F.; Wu, Y. F.; Zhang, L. H.; Ye, X. S. An efficient and improved procedure for preparation of triflyl azide and application in catalytic diazotransfer reaction. *Tetrahedron Lett.* **2005**, *46*, 8993-8995.

6. ^{1}H, ^{13}C{^{1}H}, ^{1}H-^{1}H COSY and DEPT-HSQC NMR spectra of novel compounds

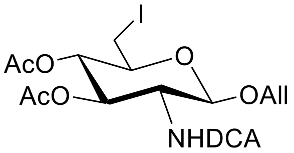

3

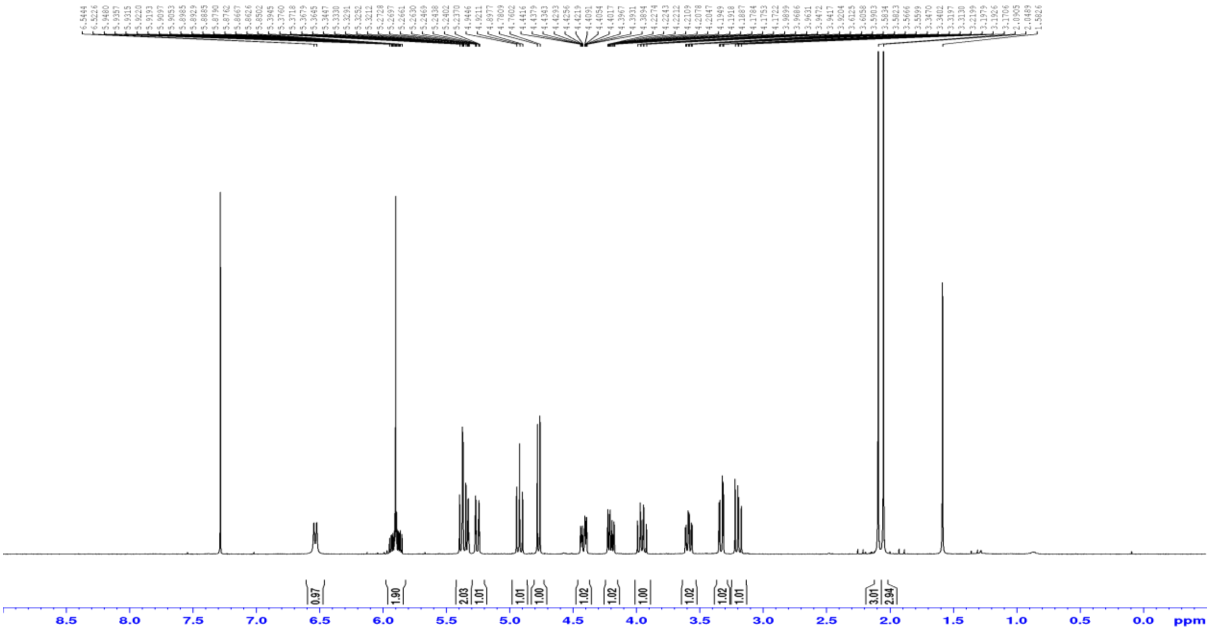

Figure. ^{1}H NMR (400 MHz, $CDCl_3$) spectrum of **3**.

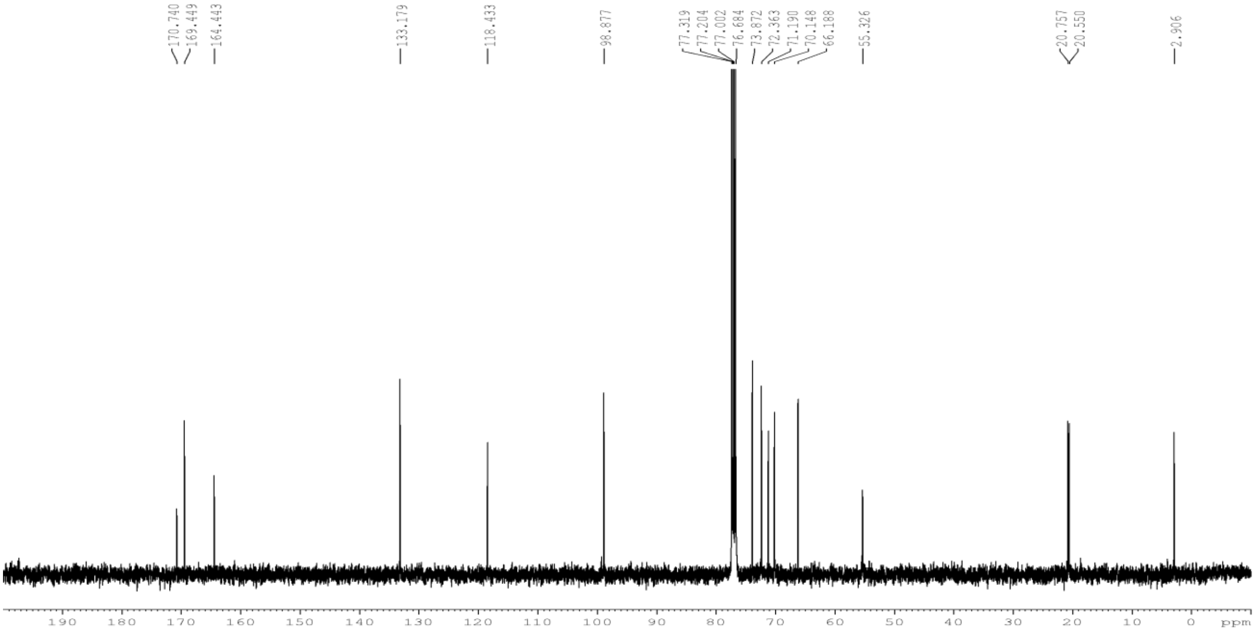

Figure. ^{13}C{^{1}H} NMR (100 MHz, $CDCl_3$) spectrum of **3**.

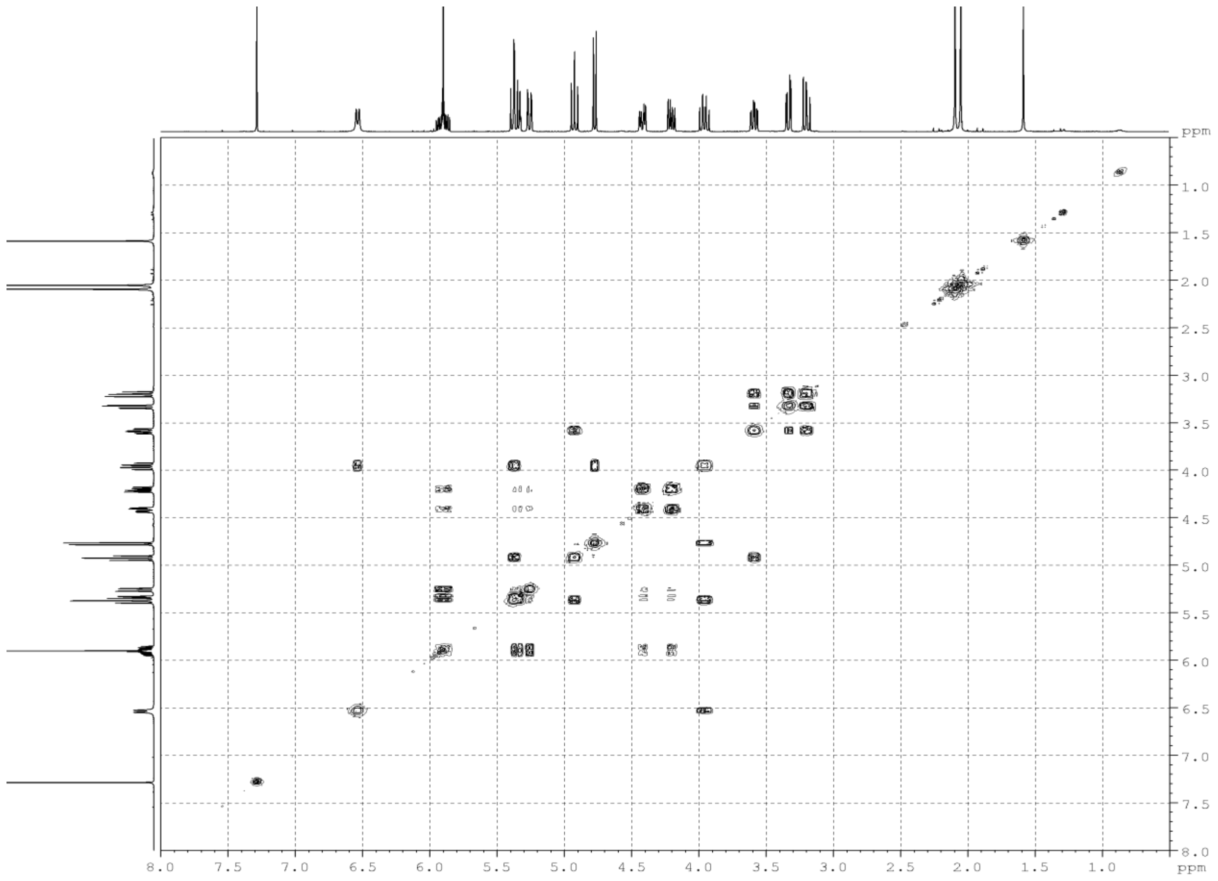

Figure. ^{1}H-^{1}H COSY NMR (400 MHz, $CDCl_3$) spectrum of **3**.

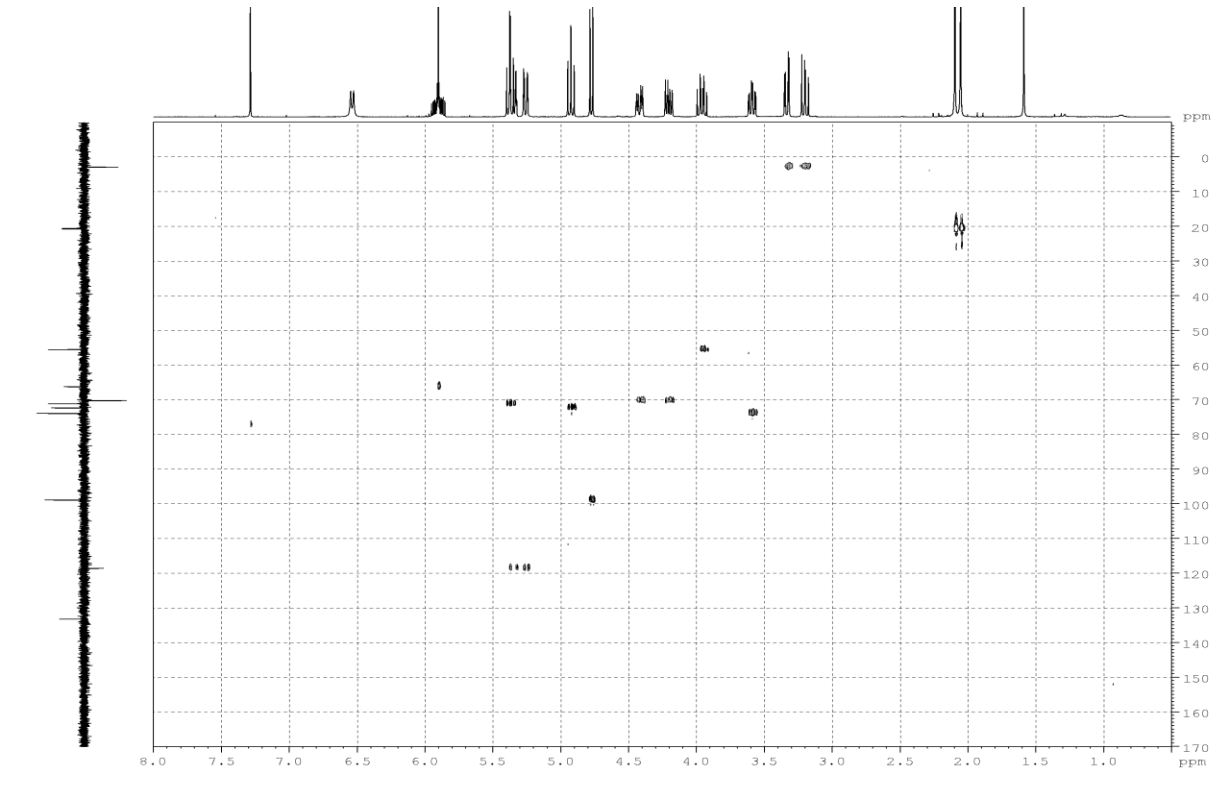

Figure. DEPT-HSQC NMR spectrum of **3**.

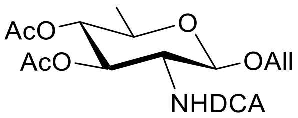

7

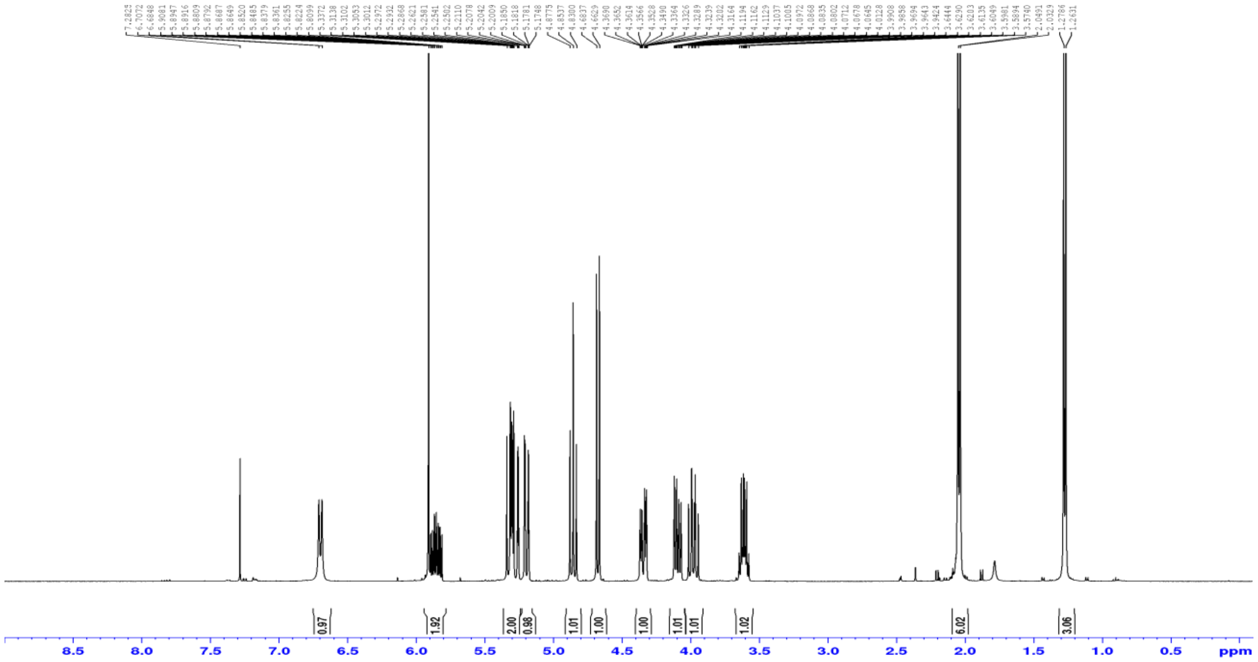

Figure. ^{1}H NMR (400 MHz, $CDCl_3$) spectrum of **7**.

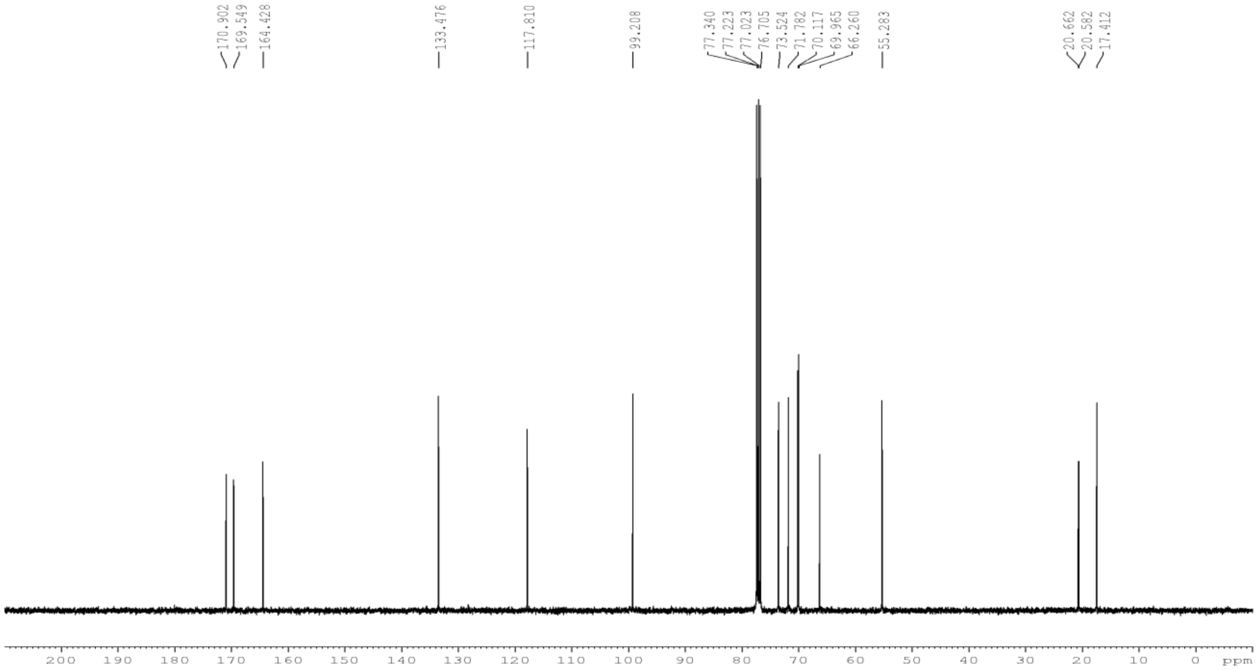

Figure. ^{13}C{^{1}H} NMR (100 MHz, $CDCl_3$) spectrum of **7**.

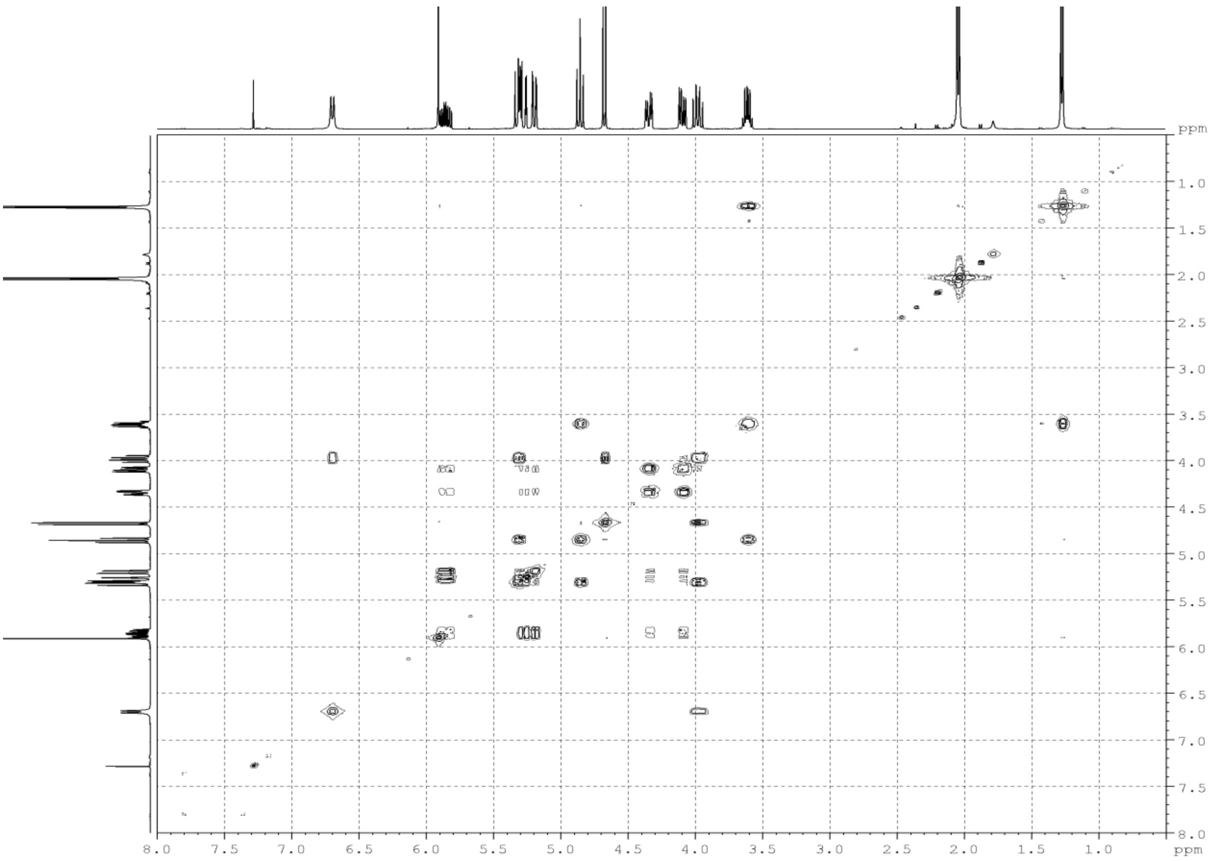

Figure. ^{1}H-^{1}H COSY NMR (400 MHz, $CDCl_3$) spectrum of **7**.

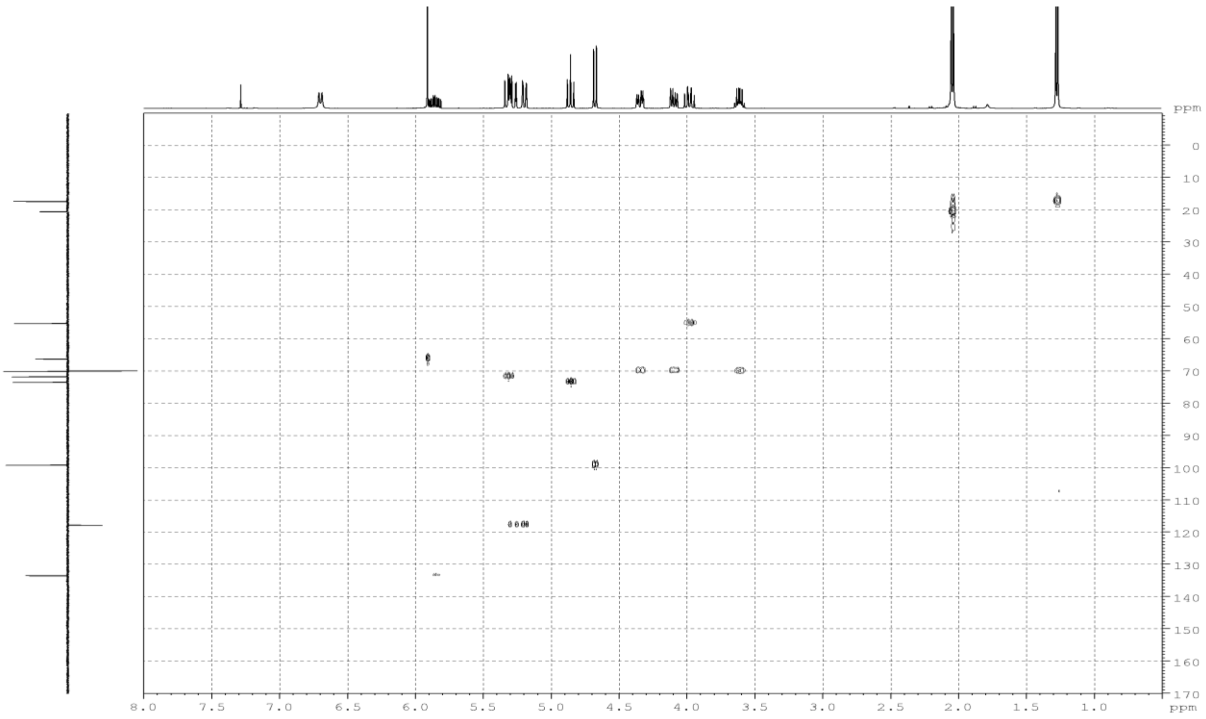

Figure. DEPT-HSQC NMR spectrum of **7**.

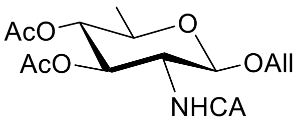

8

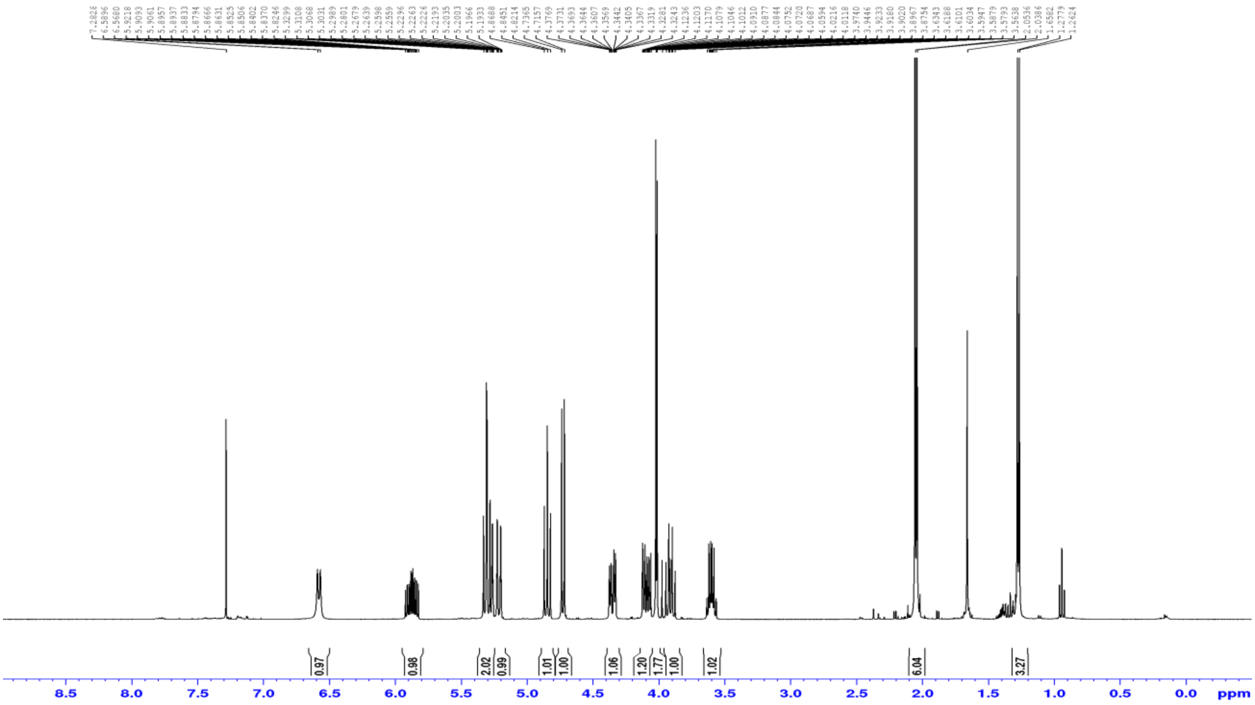

Figure. ^{1}H NMR (400 MHz, $CDCl_3$) spectrum of **8**.

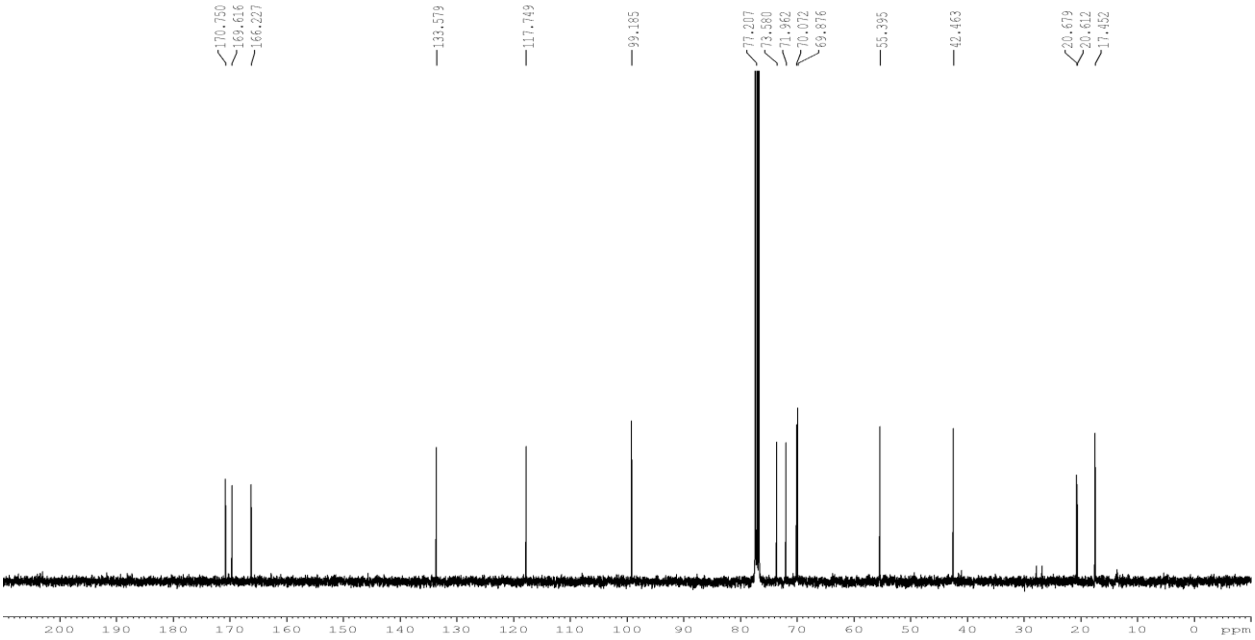

Figure. ^{13}C{^{1}H} NMR (100 MHz, $CDCl_3$) spectrum of **8**.

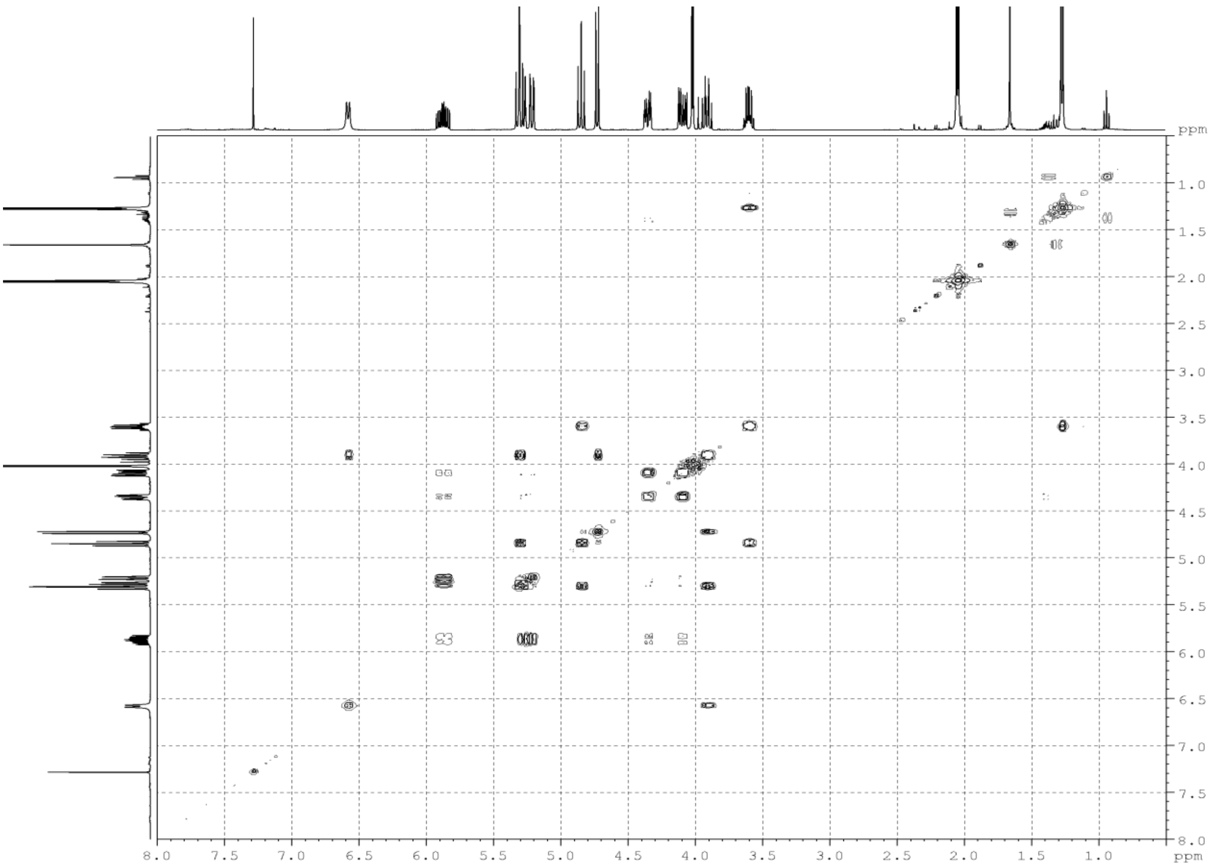

Figure. ^{1}H-^{1}H COSY NMR (400 MHz, $CDCl_3$) spectrum of **8**.

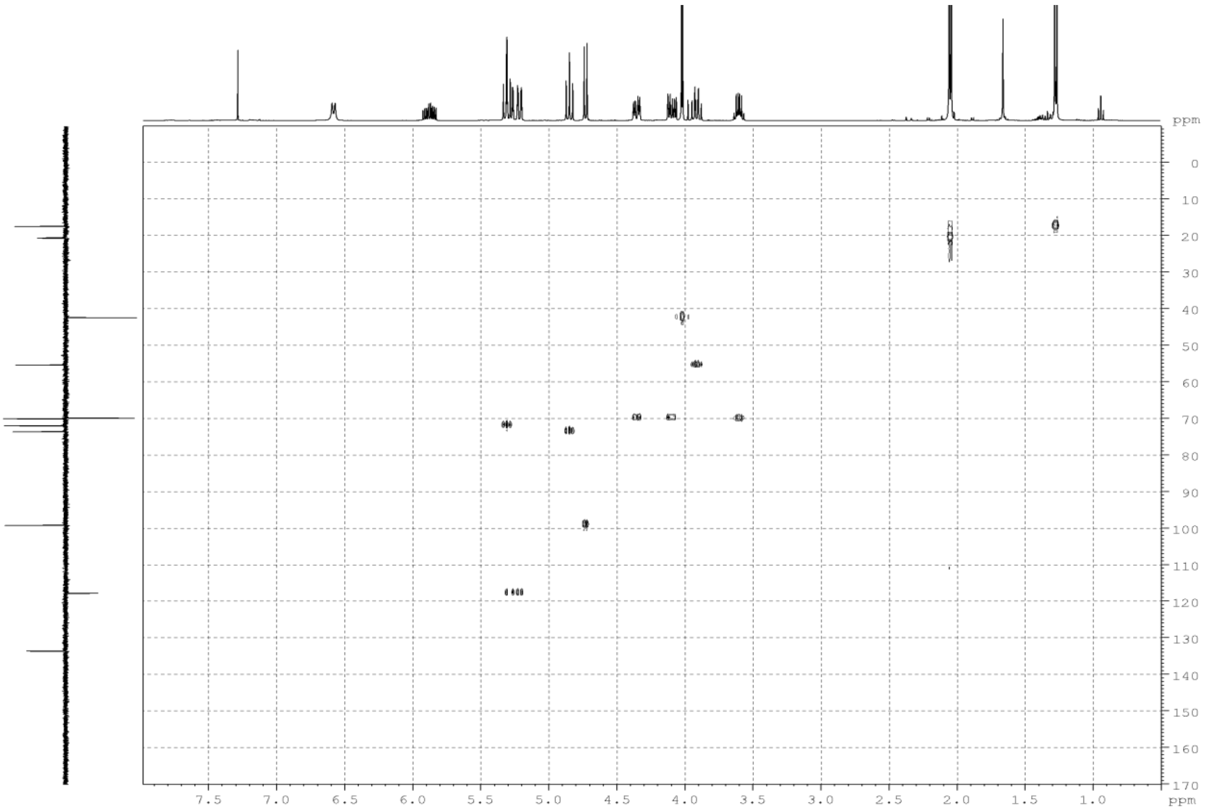

Figure. DEPT-HSQC NMR spectrum of **8**.

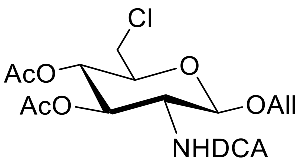

9

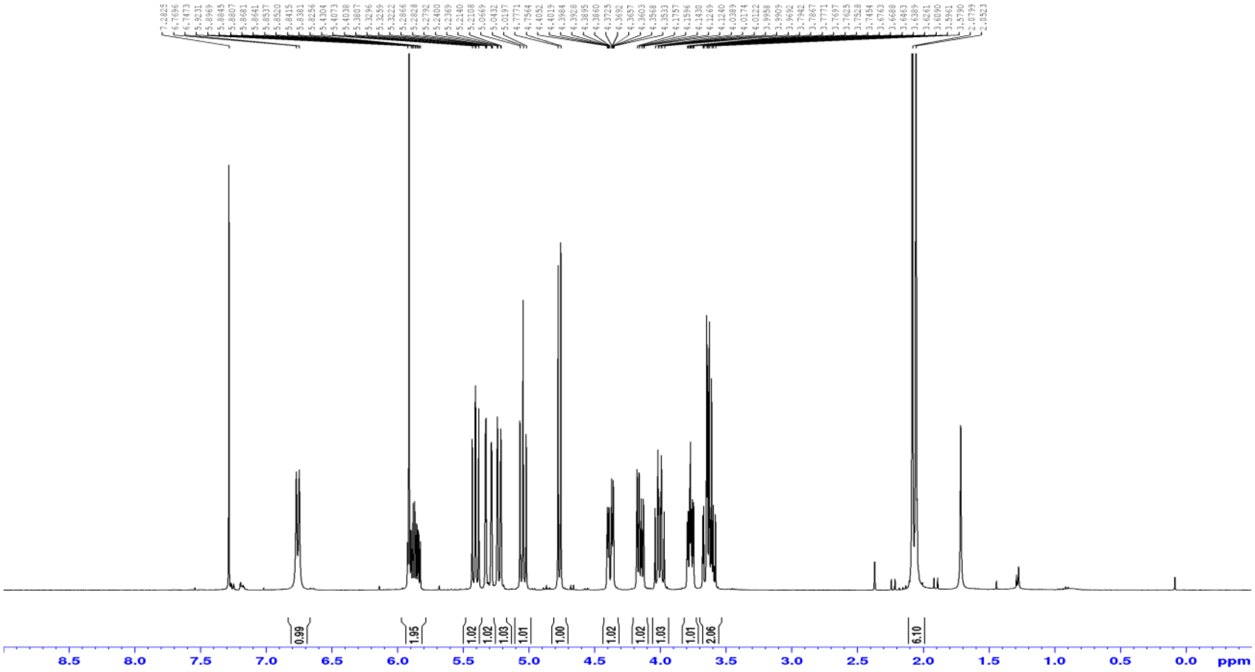

Figure. ^{1}H NMR (400 MHz, $CDCl_3$) spectrum of **9**.

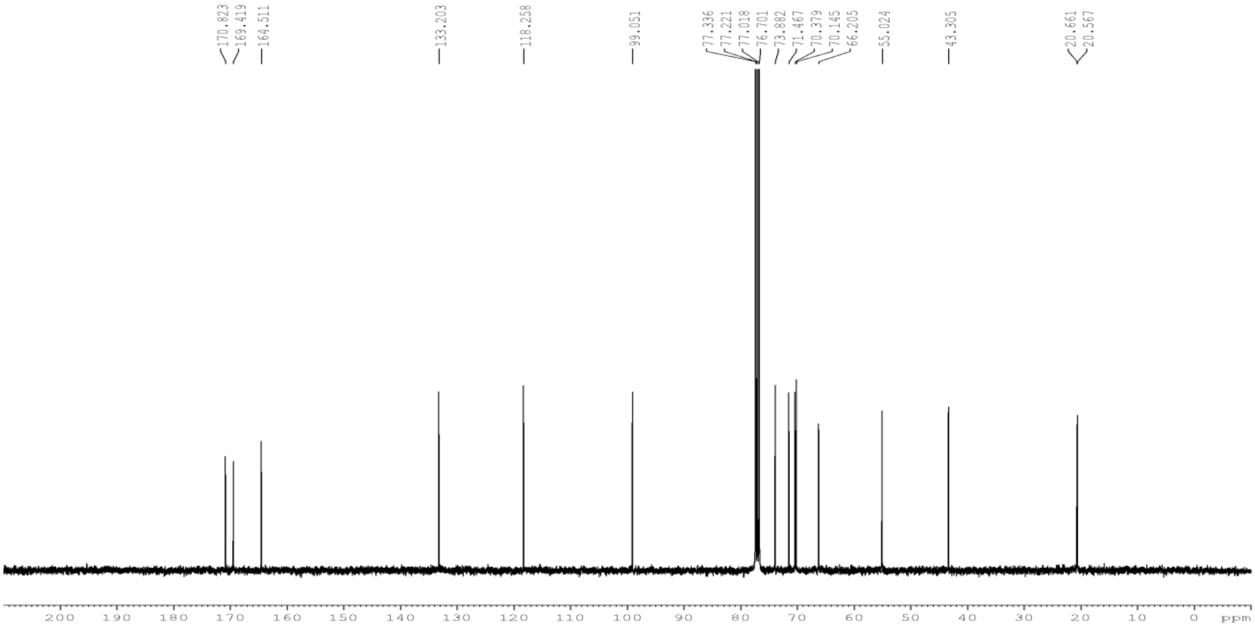

Figure. $^{13}C\{^{1}H\}$ NMR (100 MHz, $CDCl_3$) spectrum of **9**.

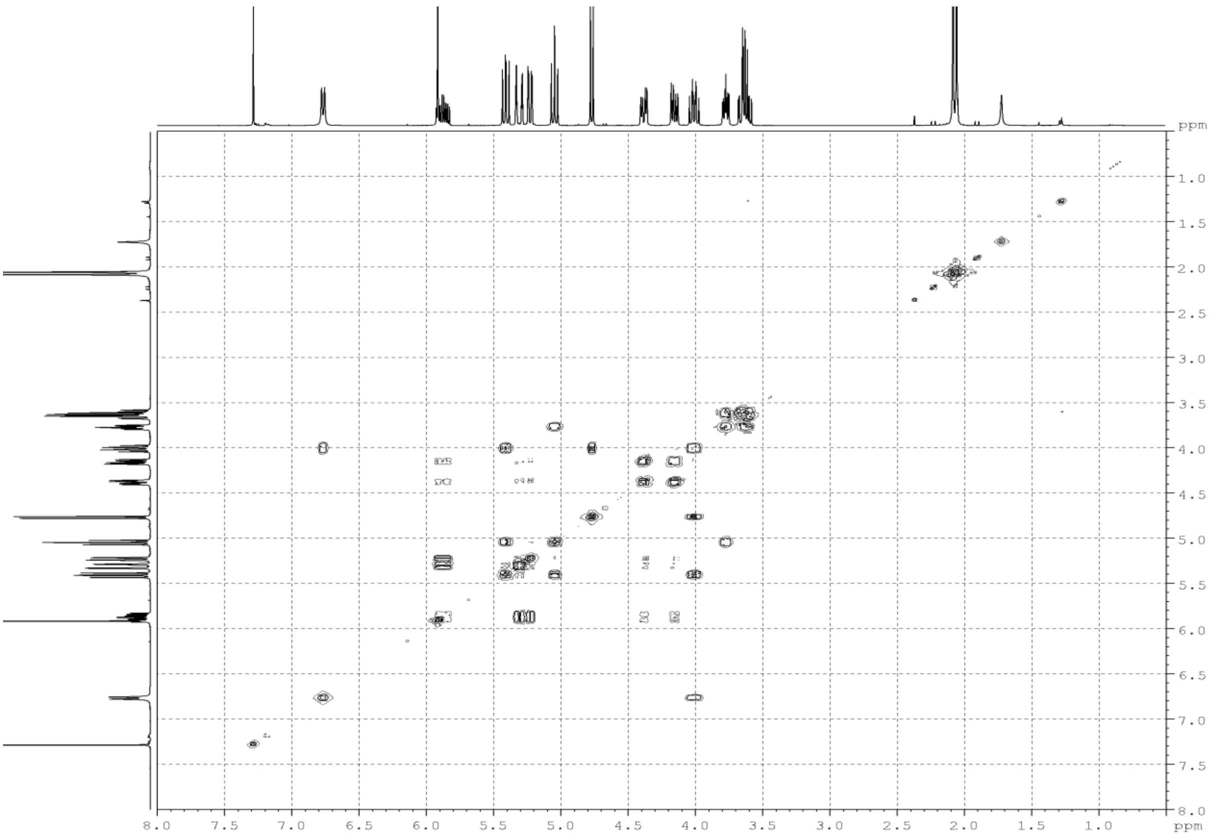

Figure. ^{1}H-^{1}H COSY NMR (400 MHz, $CDCl_3$) spectrum of **9**.

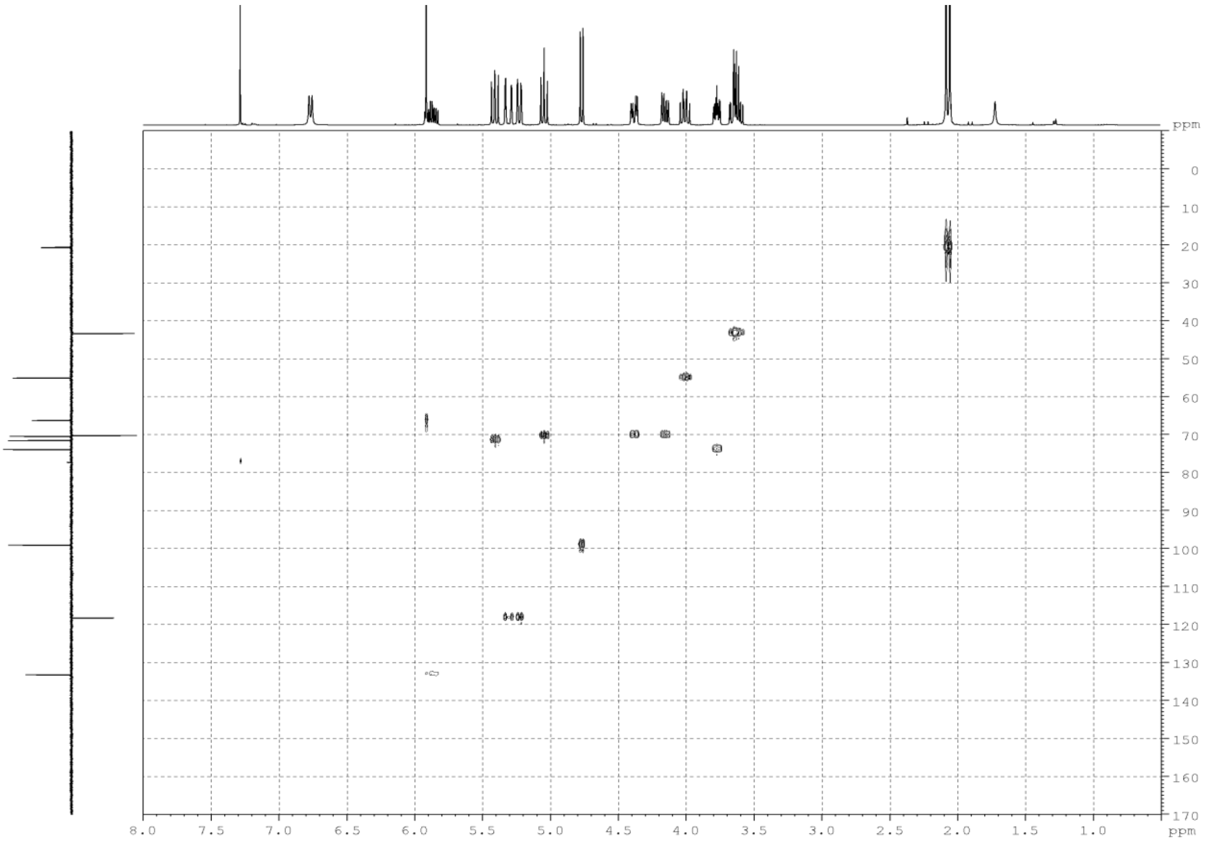

Figure. DEPT-HSQC NMR spectrum of **9**.

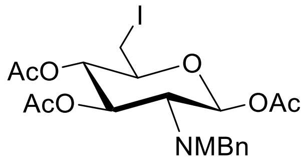

11

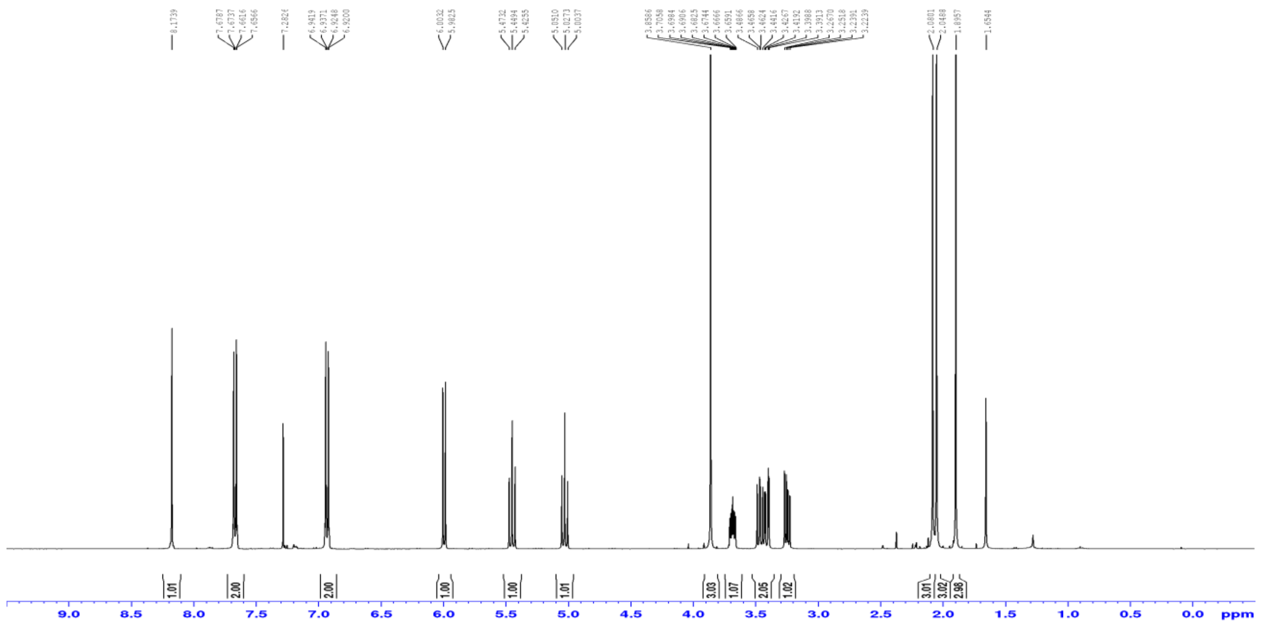

Figure. ^{1}H NMR (400 MHz, CDCl3) spectrum of **11**.

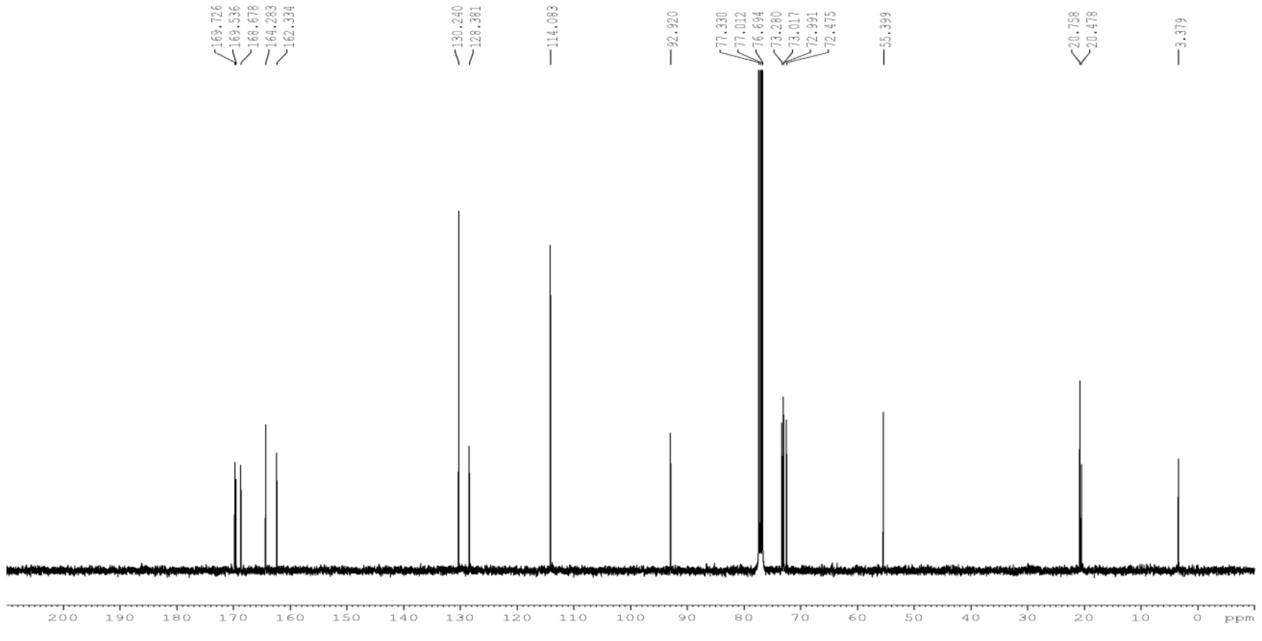

Figure. ^{13}C{^{1}H} NMR (100 MHz, CDCl3) spectrum of **11**.

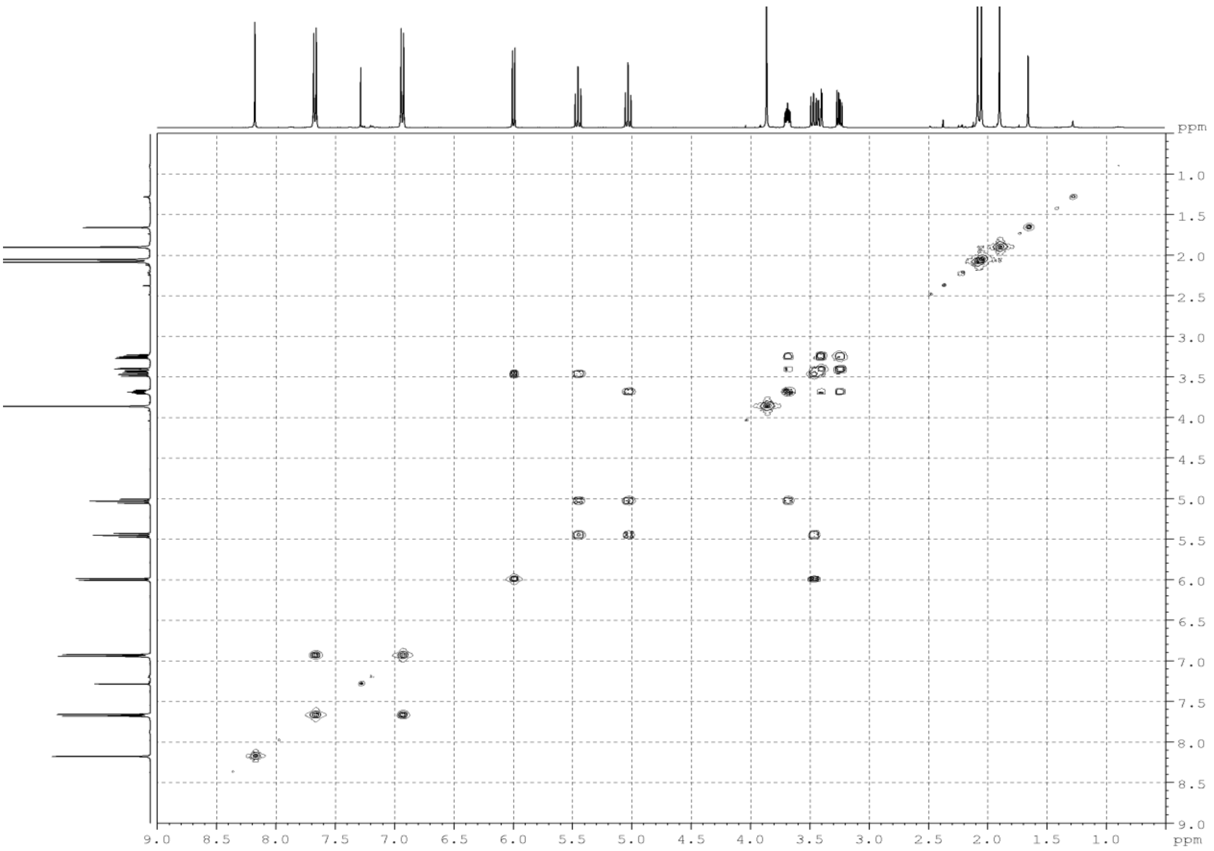

Figure. ^{1}H-^{1}H COSY NMR (400 MHz, $CDCl_3$) spectrum of **11**.

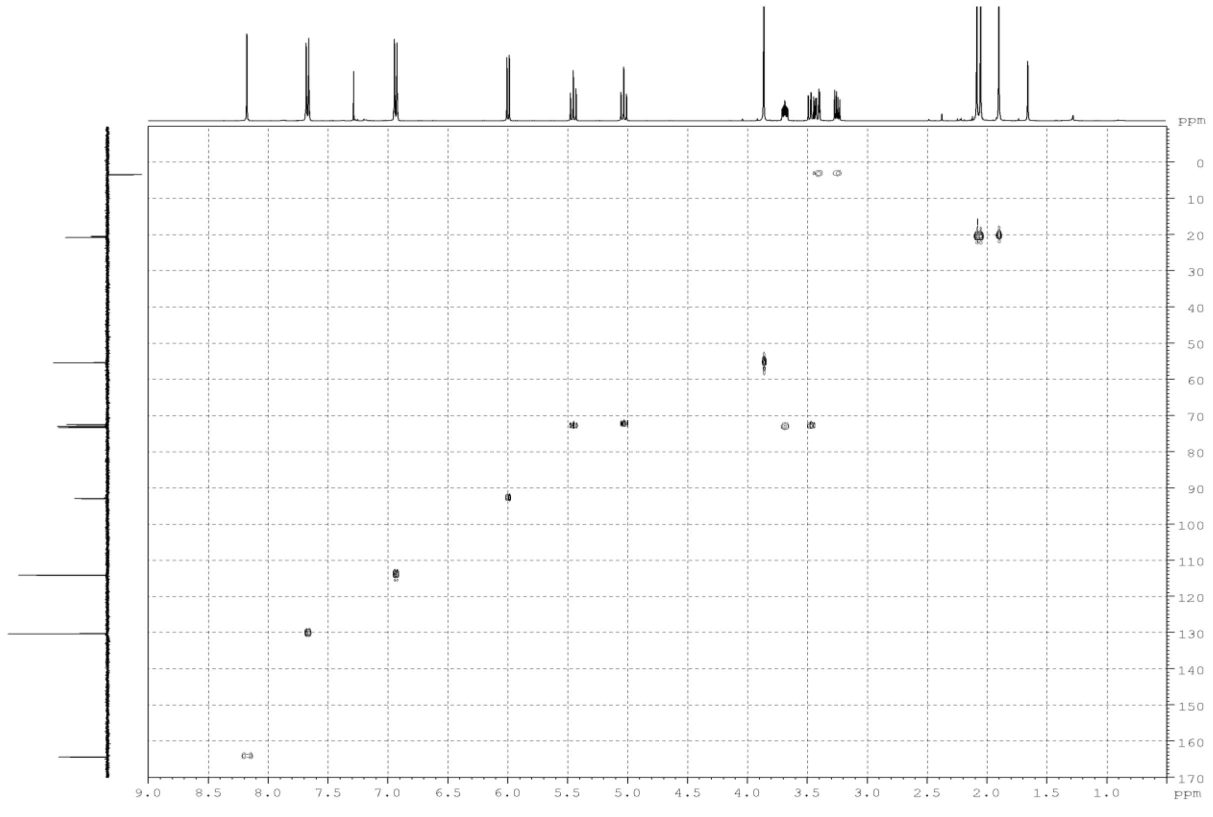

Figure. DEPT-HSQC NMR spectrum of **11**.

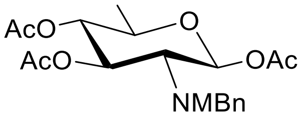

12

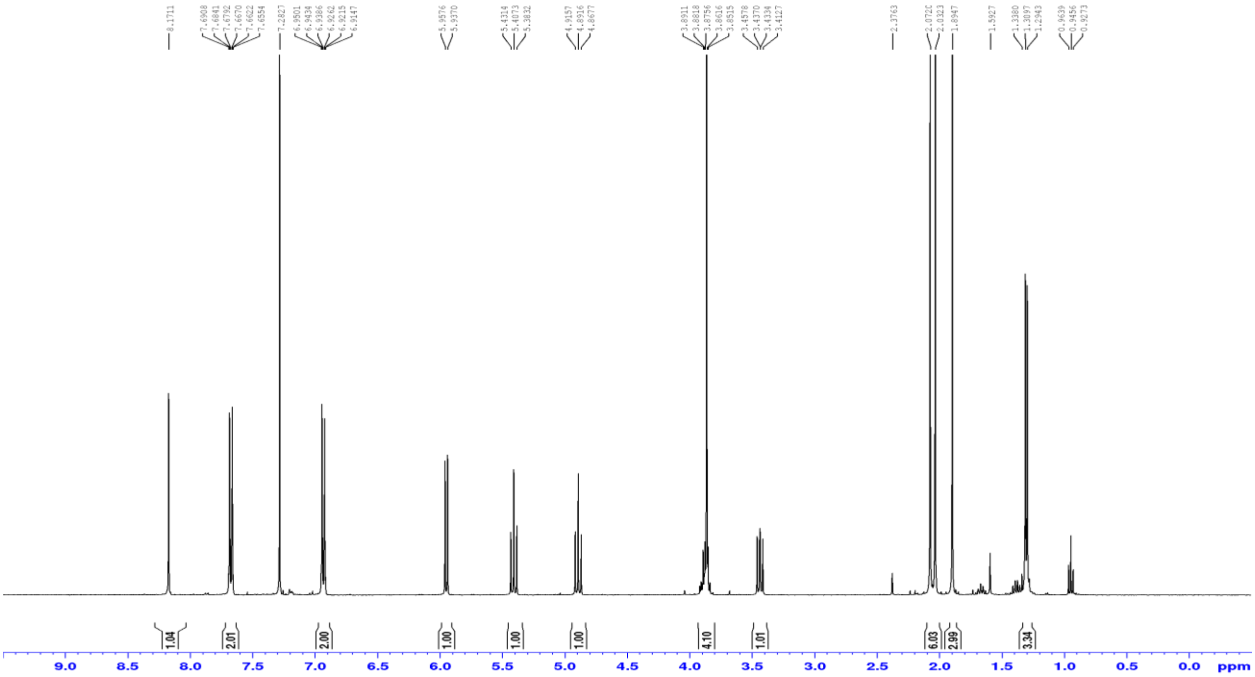

Figure. ^{1}H NMR (400 MHz, $CDCl_3$) spectrum of **12**.

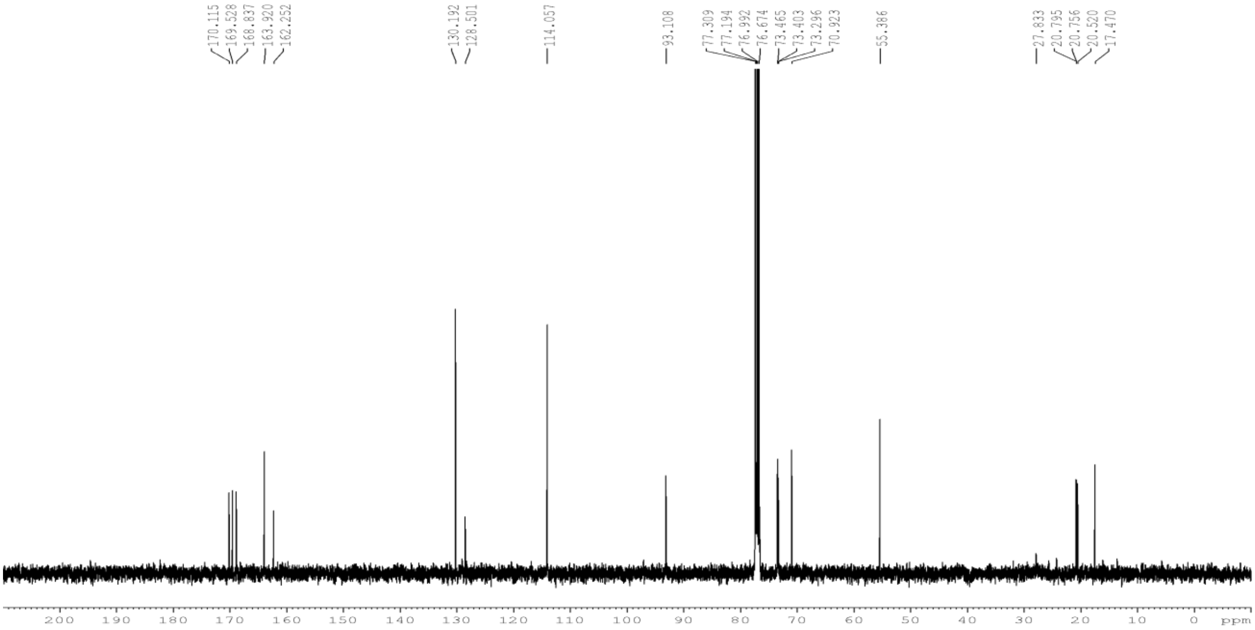

Figure. $^{13}C\{^{1}H\}$ NMR (100 MHz, $CDCl_3$) spectrum of **12**.

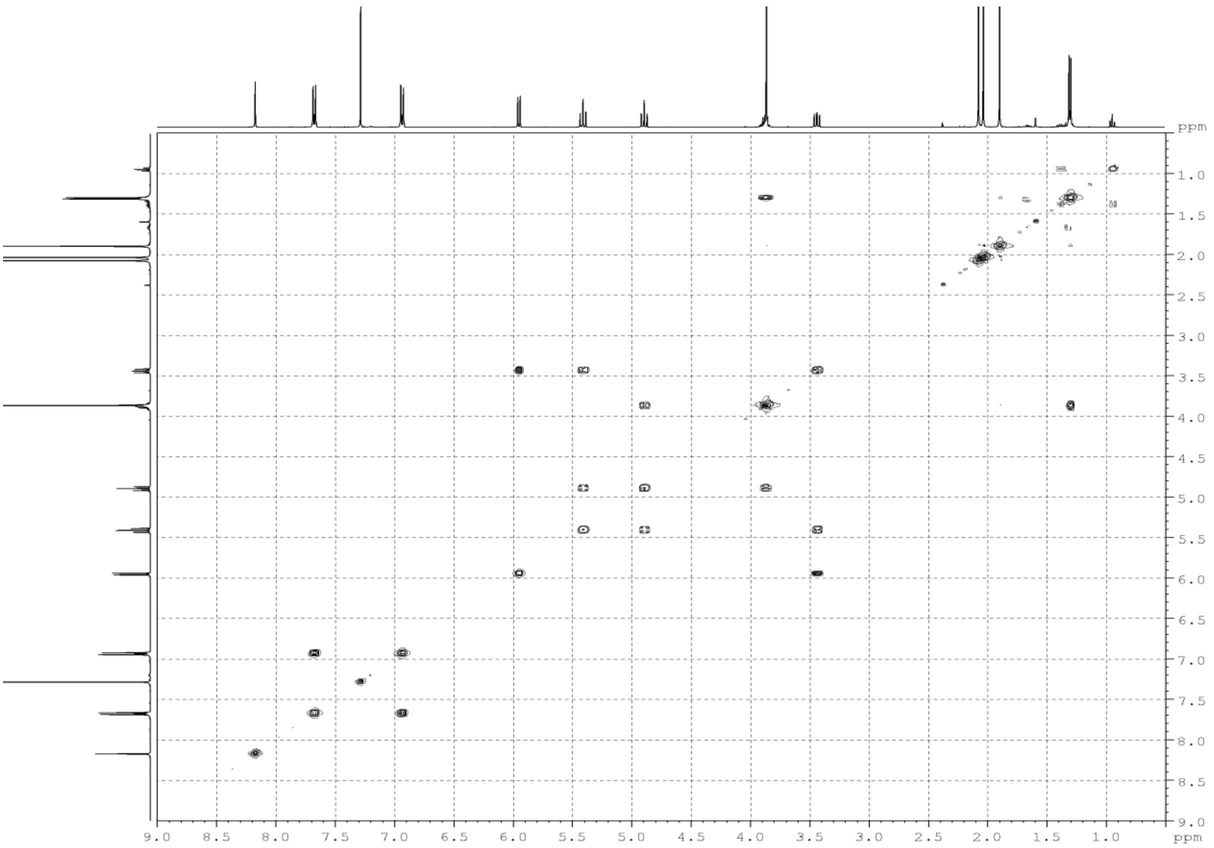

Figure. ^{1}H-^{1}H COSY NMR (400 MHz, $CDCl_3$) spectrum of **12**.

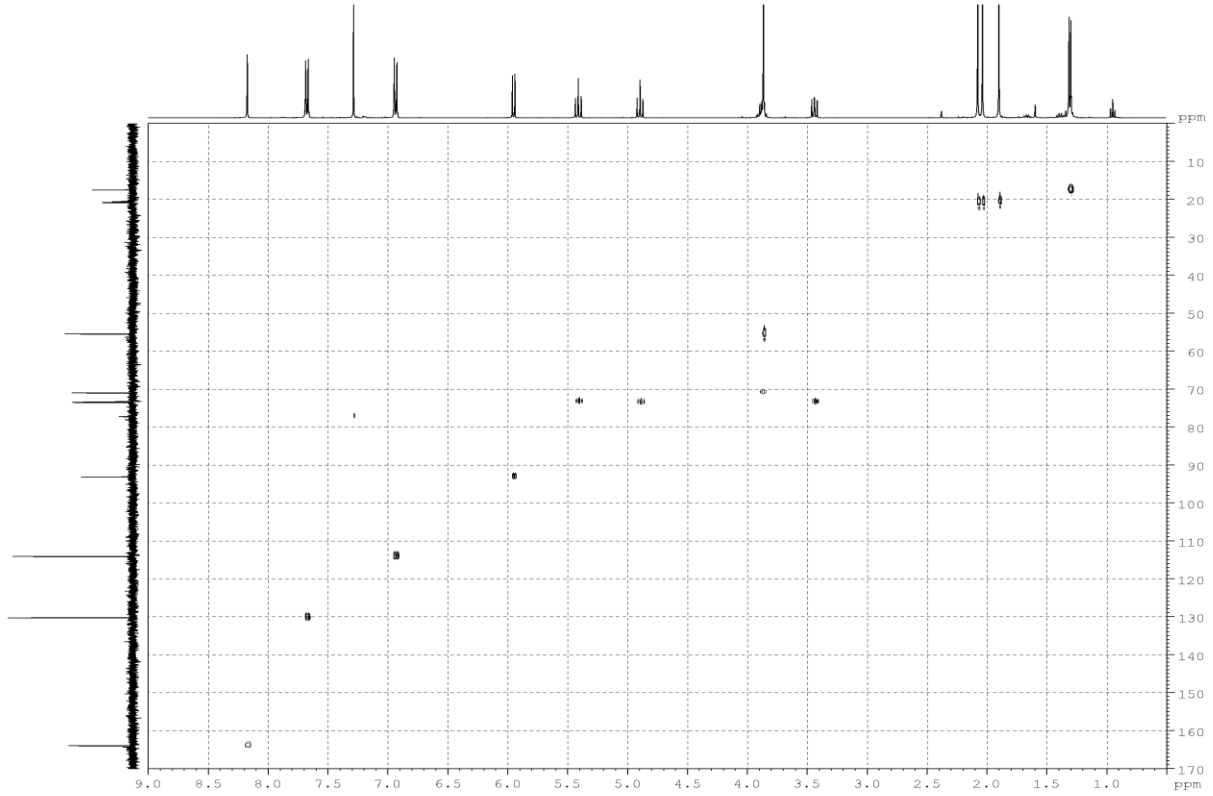

Figure. DEPT-HSQC NMR spectrum of **12**.

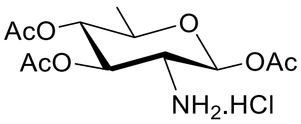

13

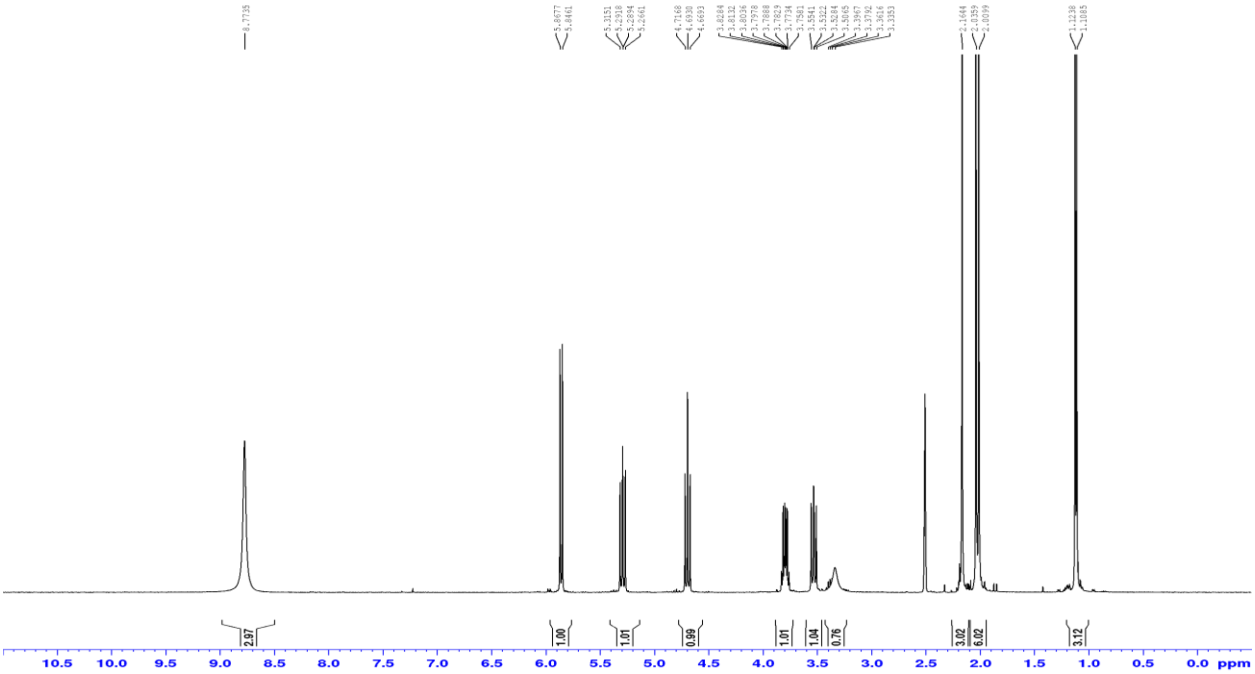

Figure. ^{1}H NMR (400 MHz, DMSO-d_6) spectrum of **13**.

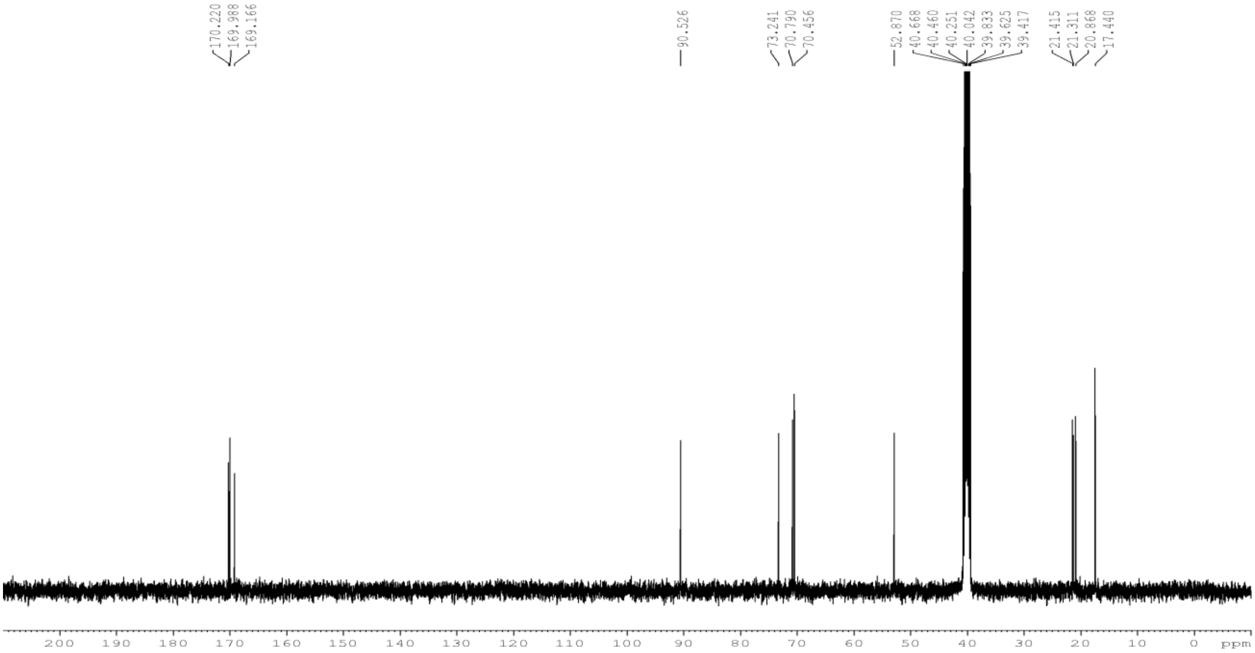

Figure. ^{13}C{^{1}H} NMR (100 MHz, DMSO-d_6) spectrum of **13**.

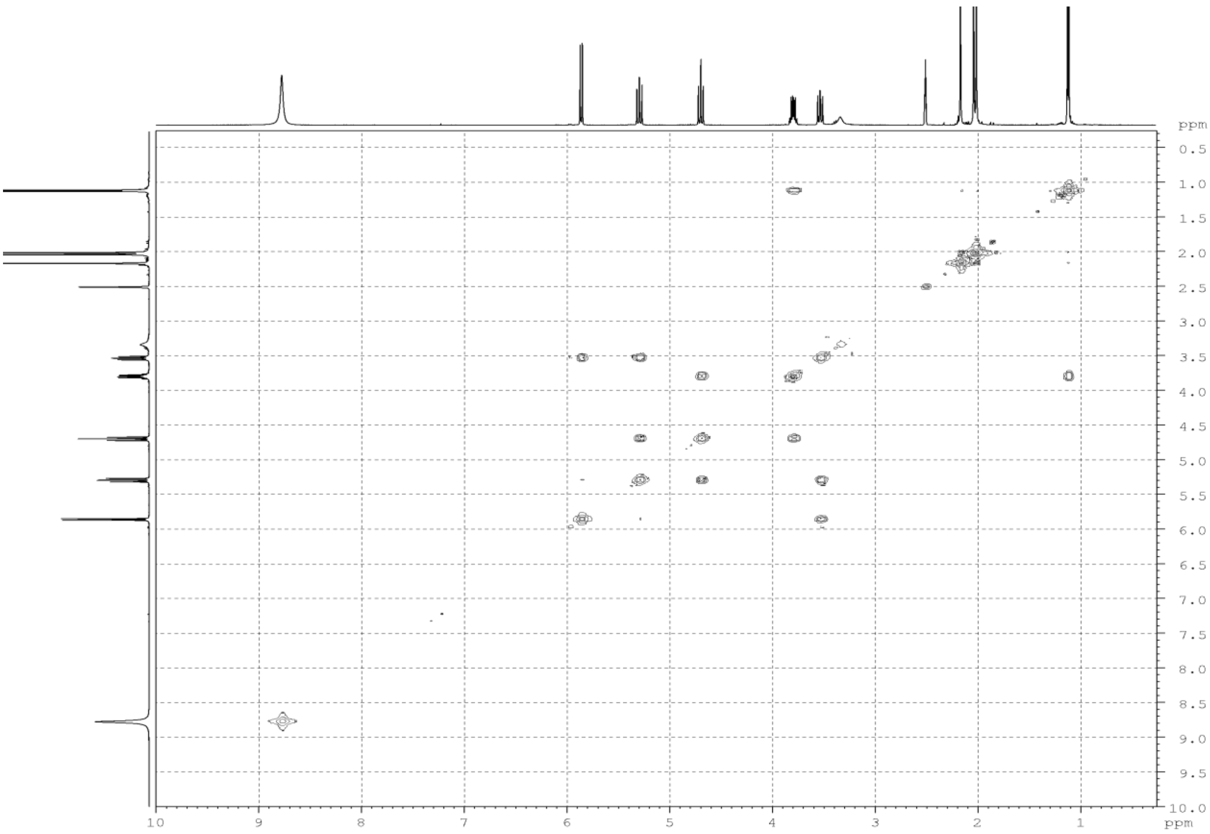

Figure. ^{1}H-^{1}H COSY NMR (400 MHz, DMSO-*d*$_6$) spectrum of **13**.

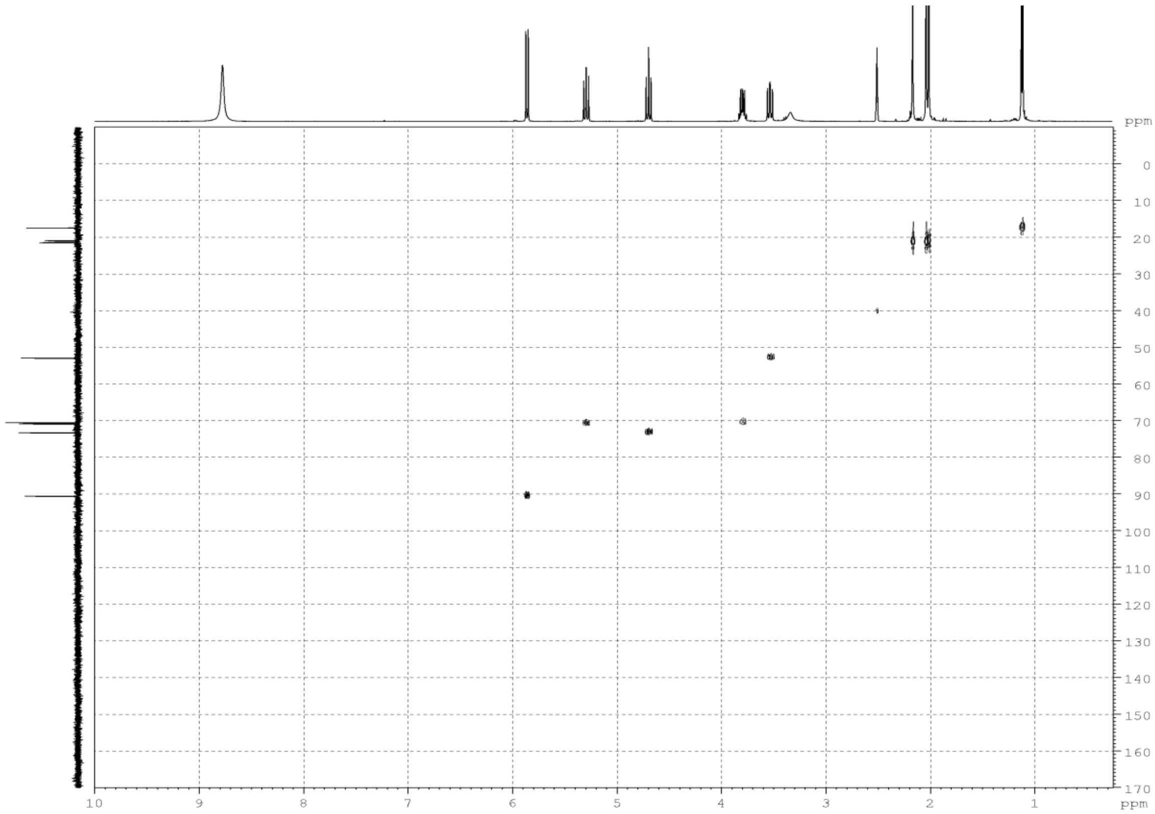

Figure. DEPT-HSQC NMR spectrum of **13**.

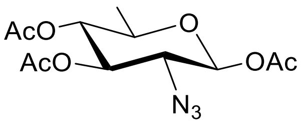

14

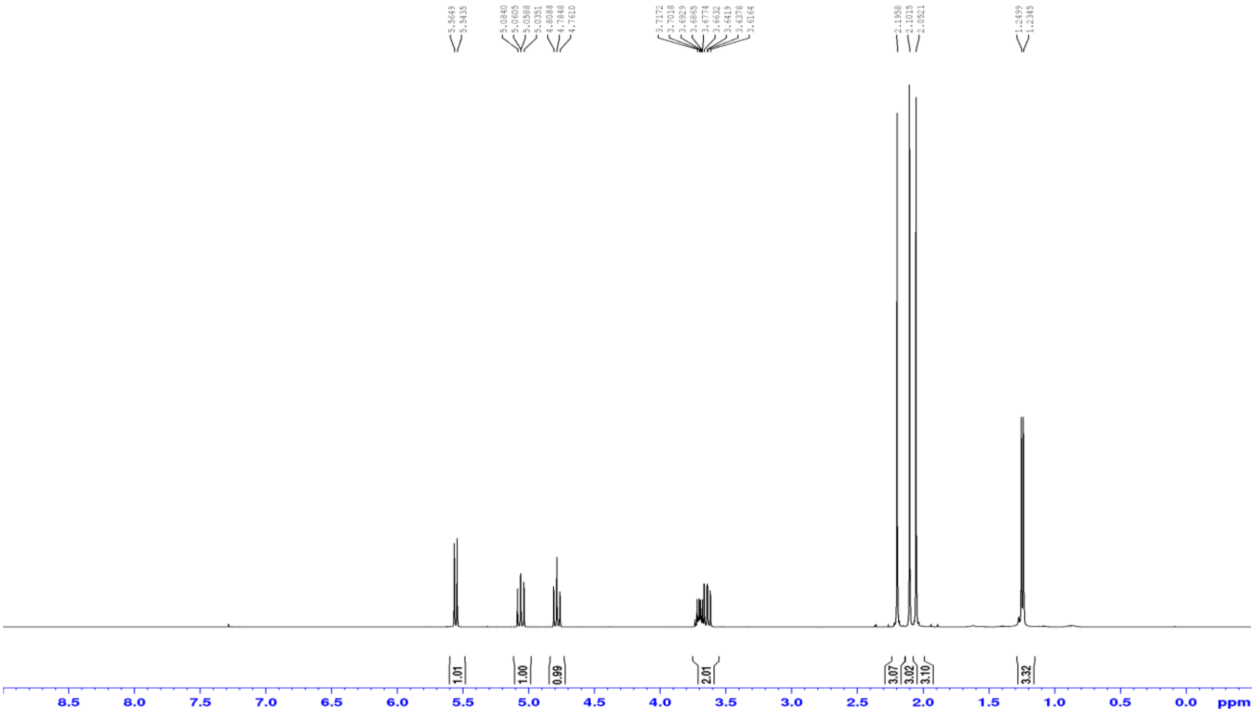

Figure. ^{1}H NMR (400 MHz, $CDCl_3$) spectrum of **14**.

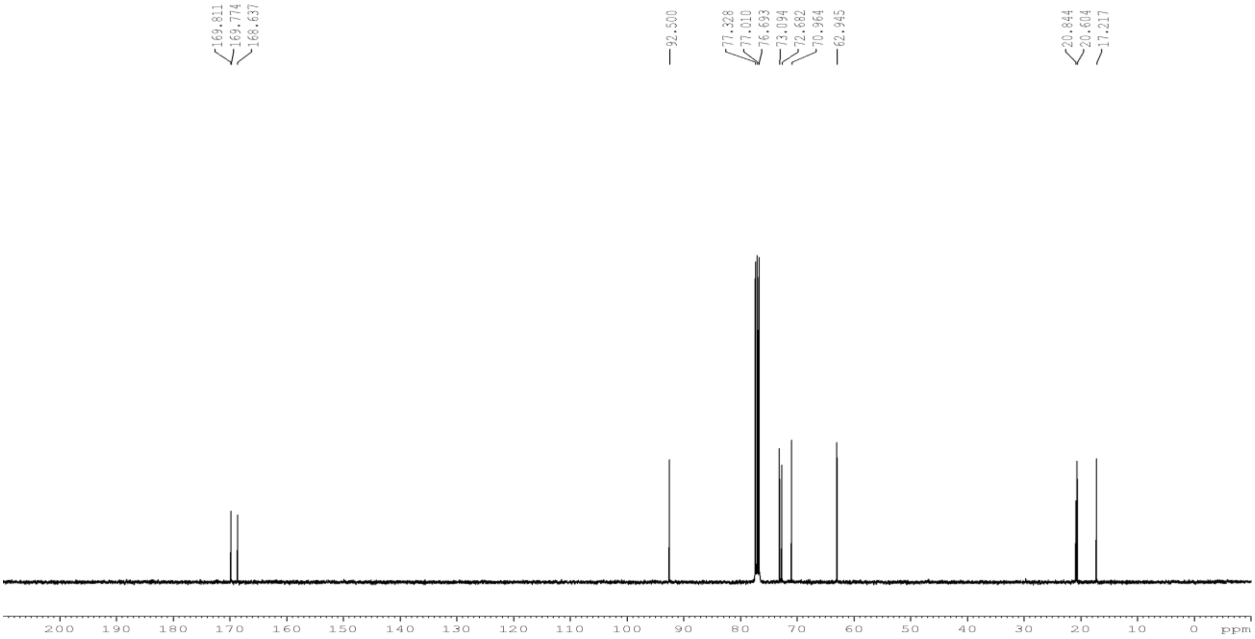

Figure. $^{13}C\{^{1}H\}$ NMR (100 MHz, $CDCl_3$) spectrum of **14**.

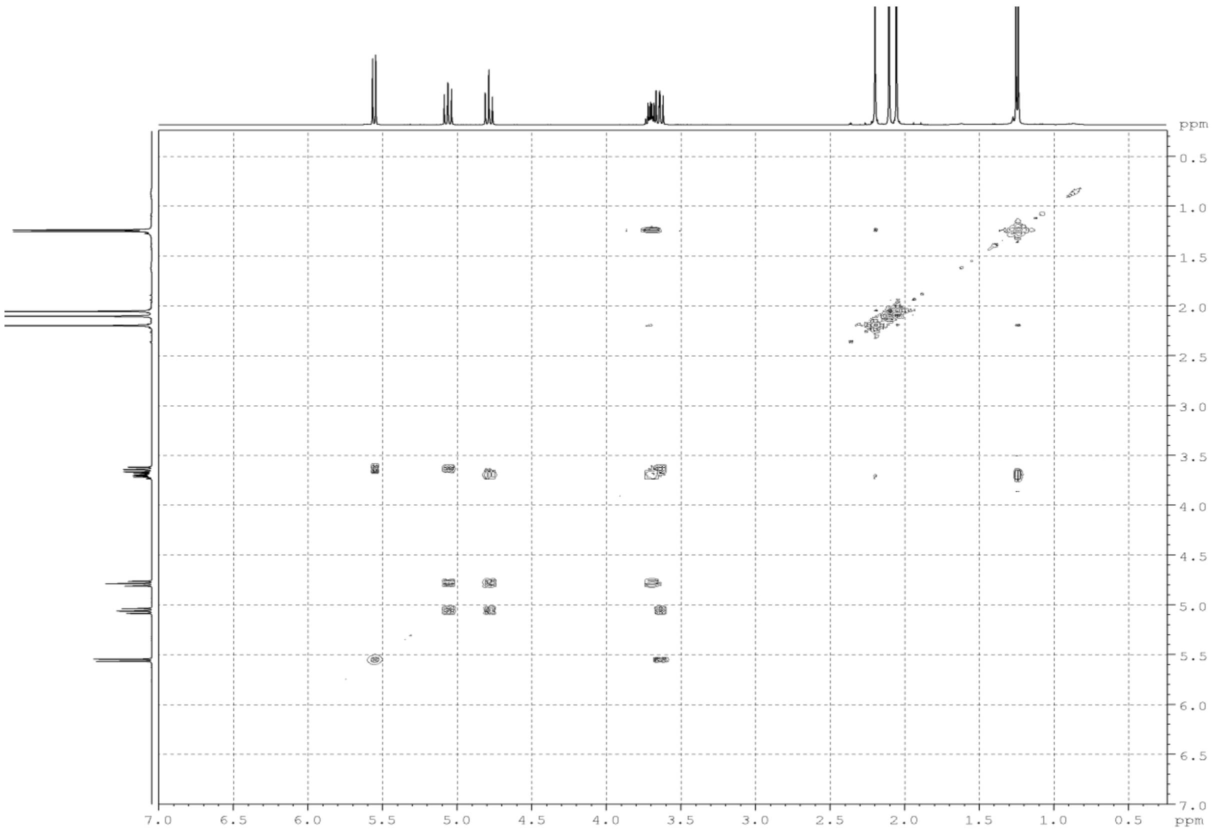

Figure. ^{1}H-^{1}H COSY NMR (400 MHz, $CDCl_3$) spectrum of **14**.

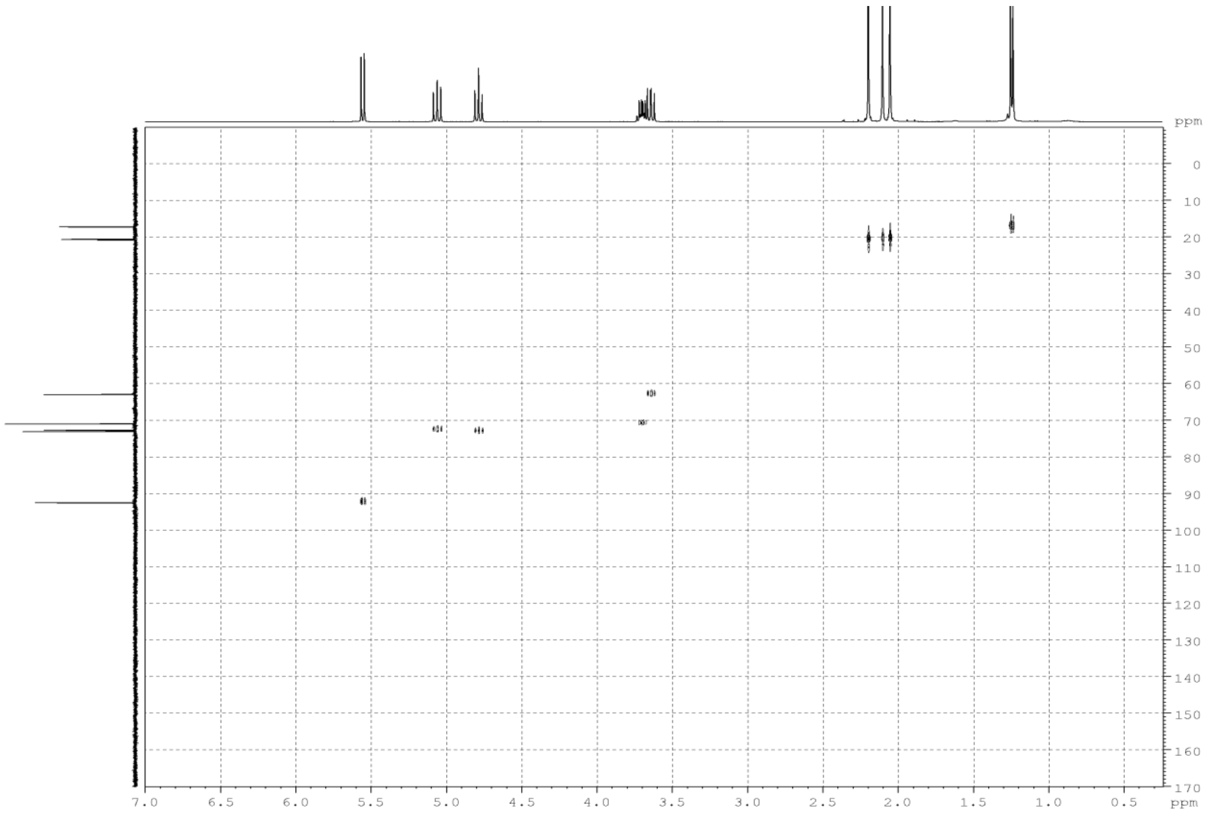

Figure. DEPT-HSQC NMR spectrum of **14**.

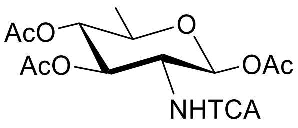

15

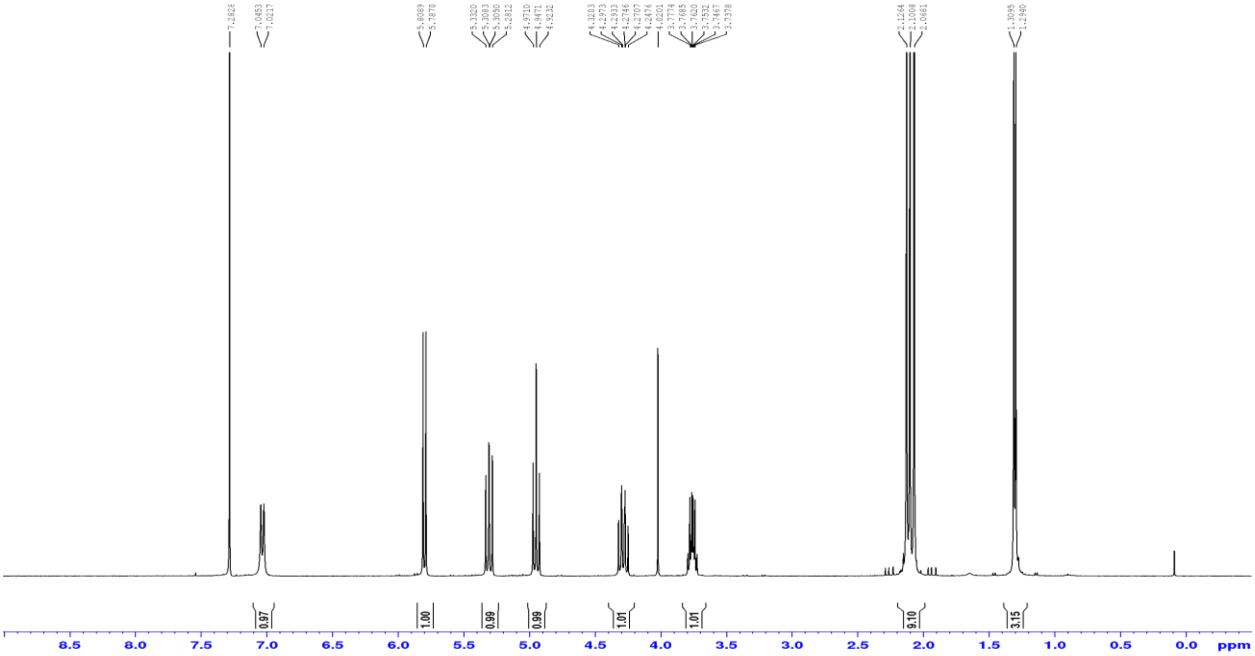

Figure. ^{1}H NMR (400 MHz, $CDCl_3$) spectrum of **15**.

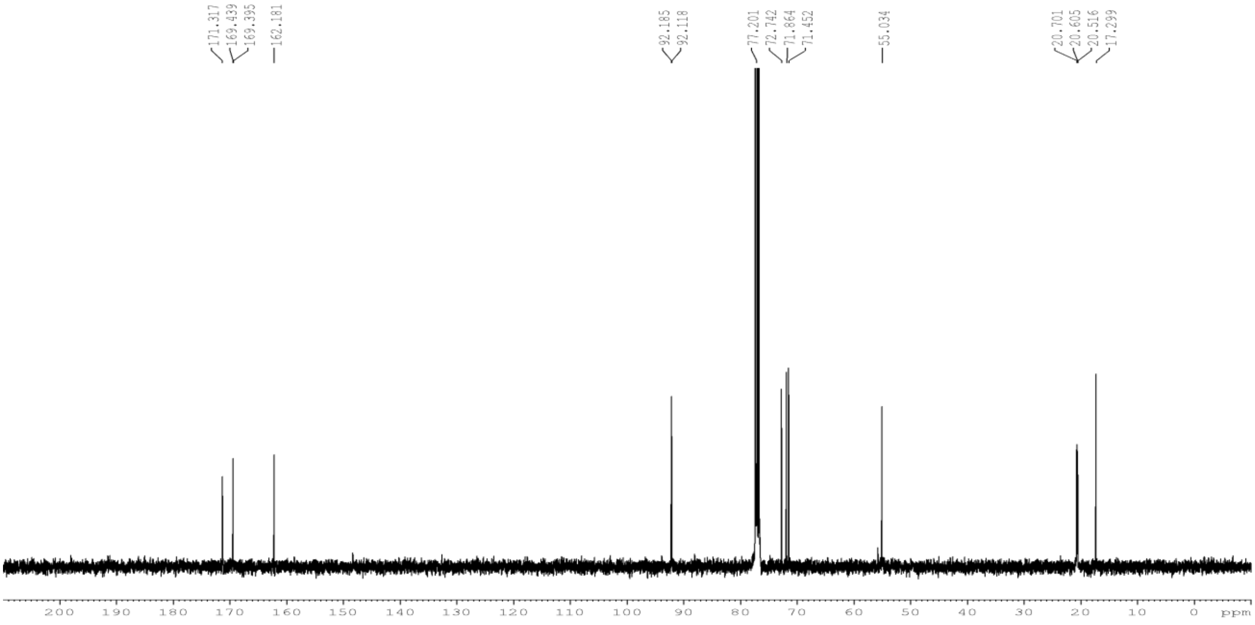

Figure. $^{13}C\{^{1}H\}$ NMR (100 MHz, $CDCl_3$) spectrum of **15**.

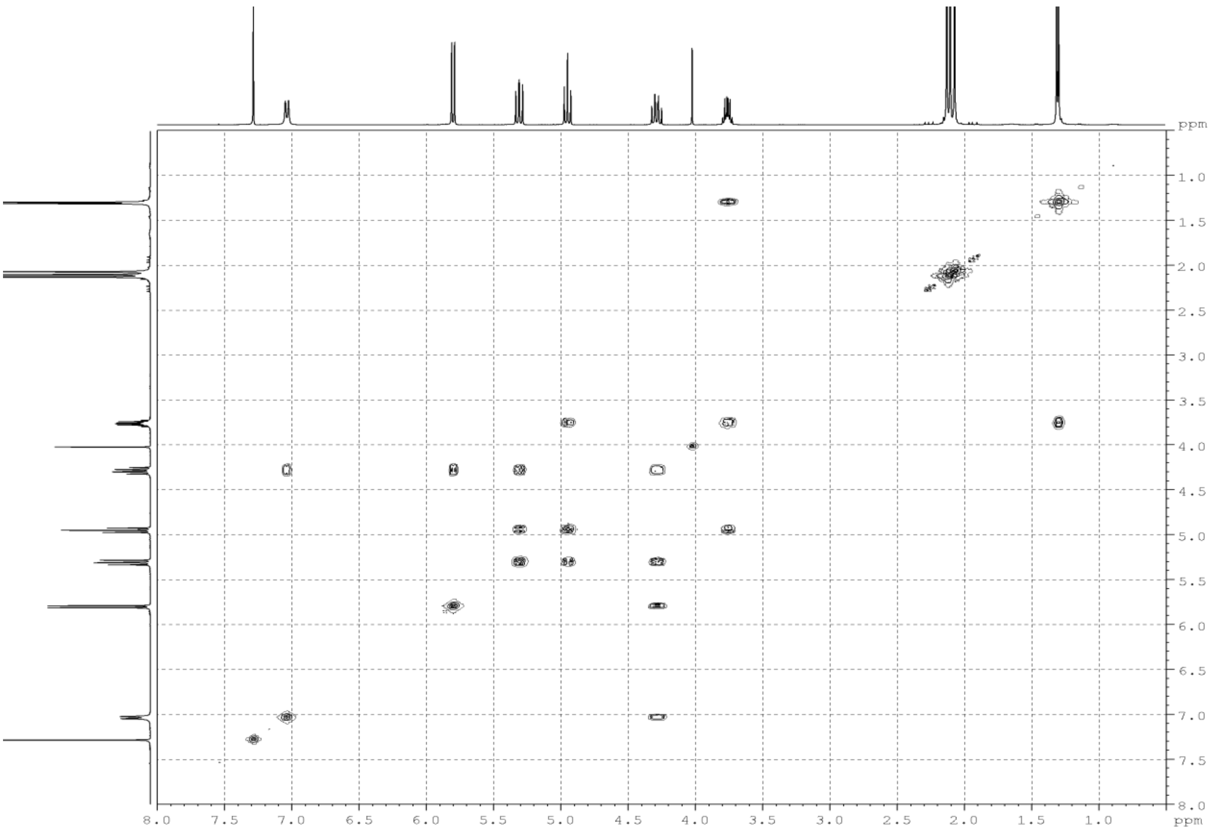

Figure. ^{1}H-^{1}H COSY NMR (400 MHz, $CDCl_3$) spectrum of **15**.

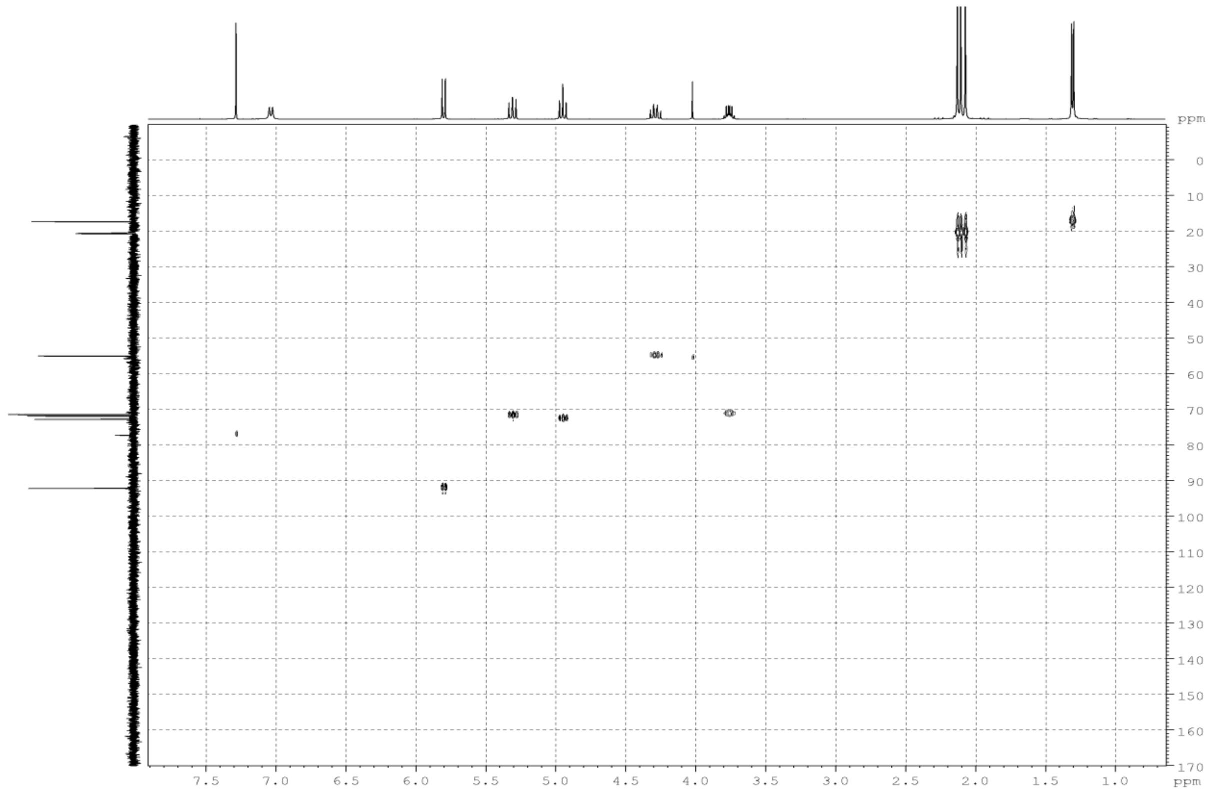

Figure. DEPT-HSQC NMR spectrum of **15**.

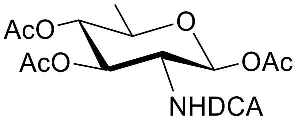

16

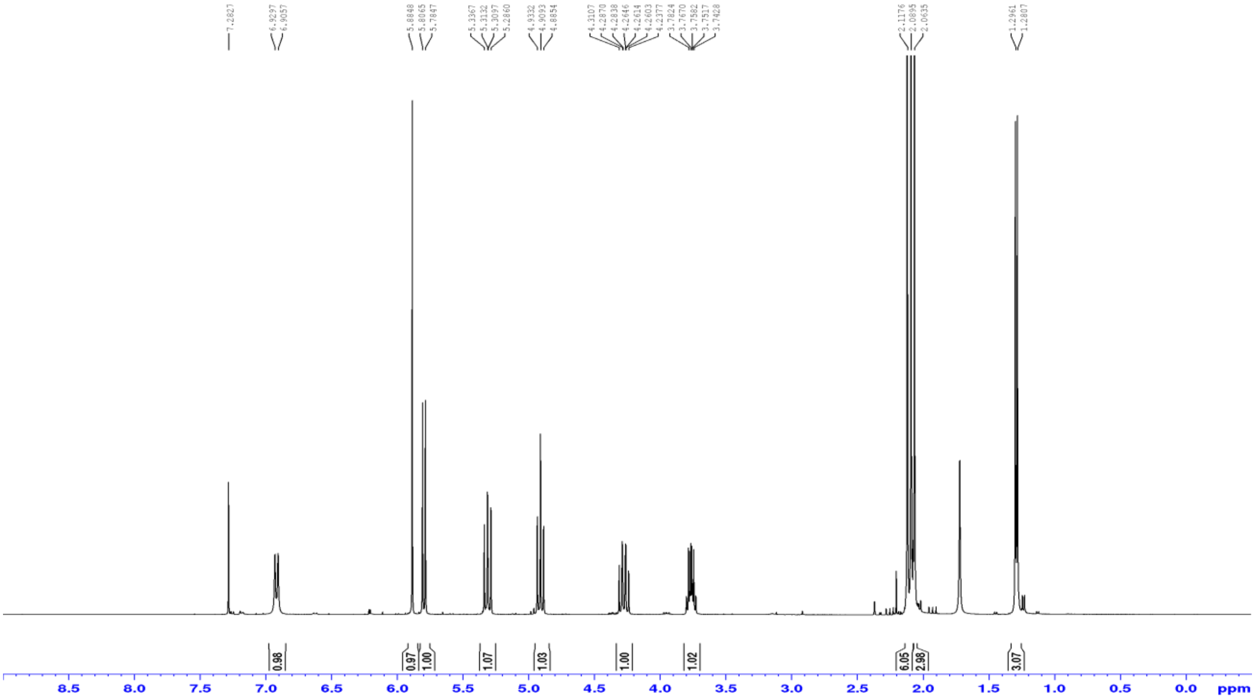

Figure. ^{1}H NMR (400 MHz, $CDCl_3$) spectrum of **16**.

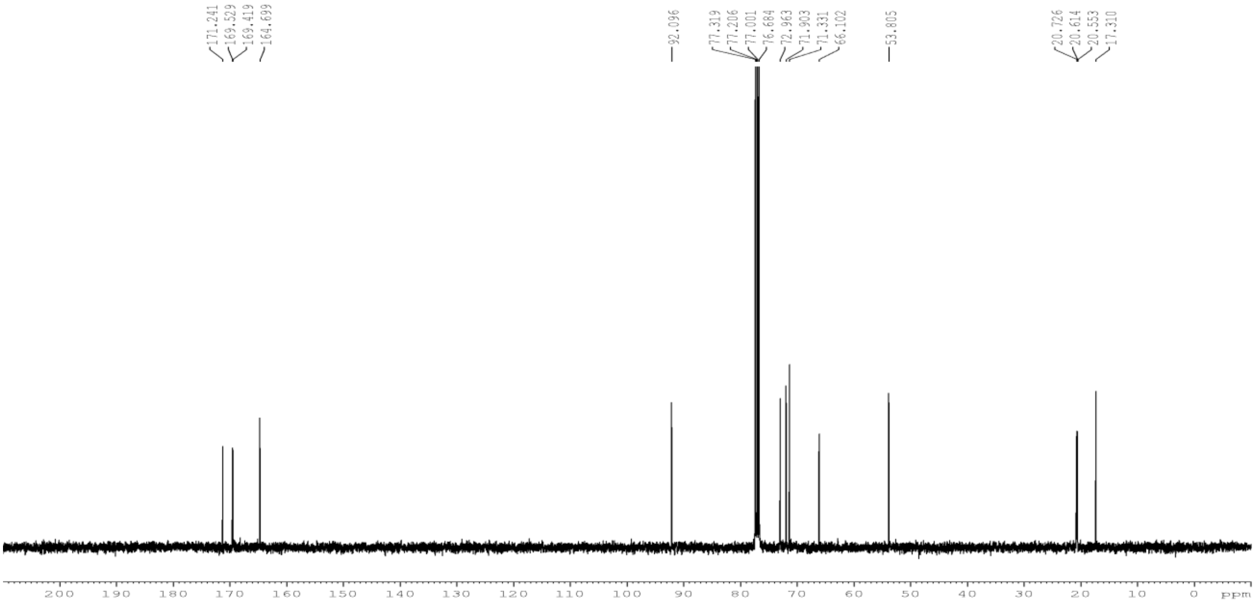

Figure. ^{13}C{^{1}H} NMR (100 MHz, $CDCl_3$) spectrum of **16**.

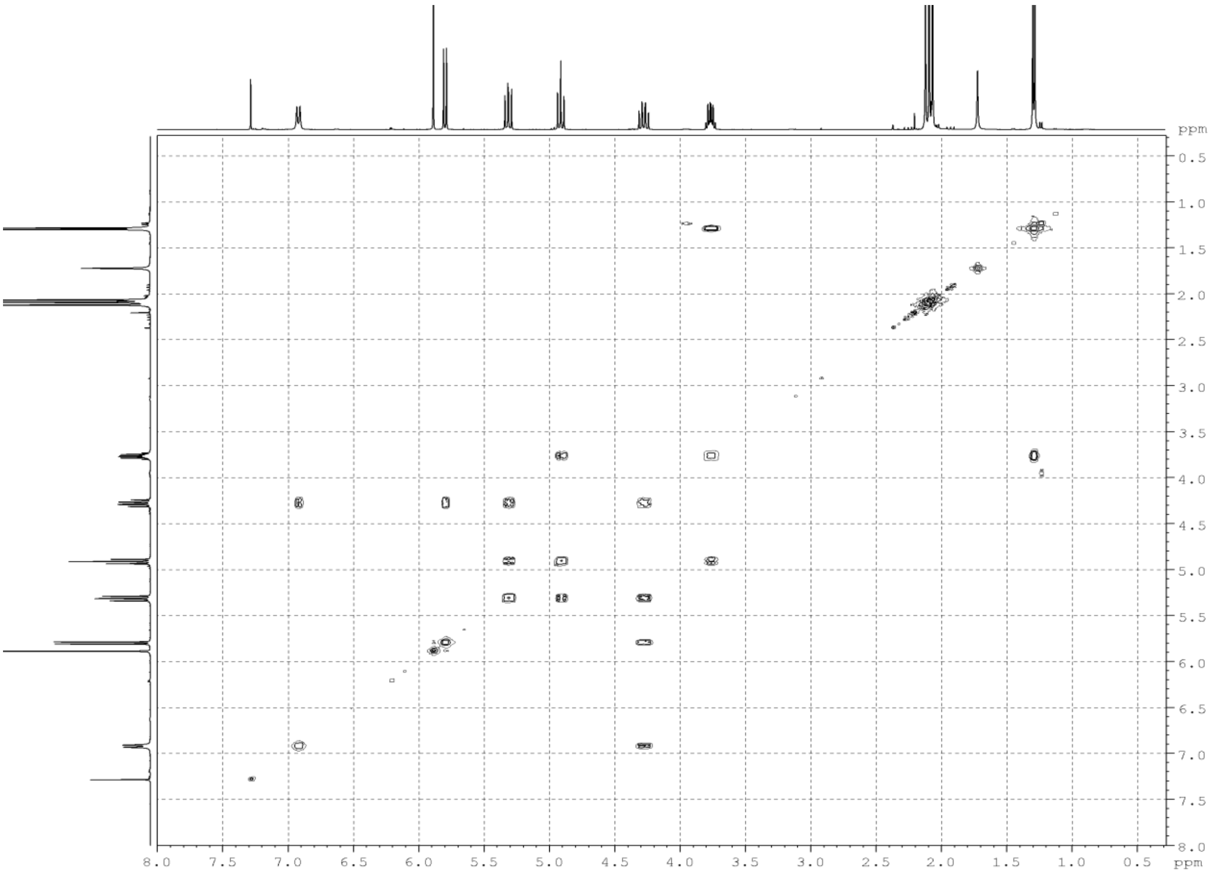

Figure. ^1H-^1H COSY NMR (400 MHz, $CDCl_3$) spectrum of **16**.

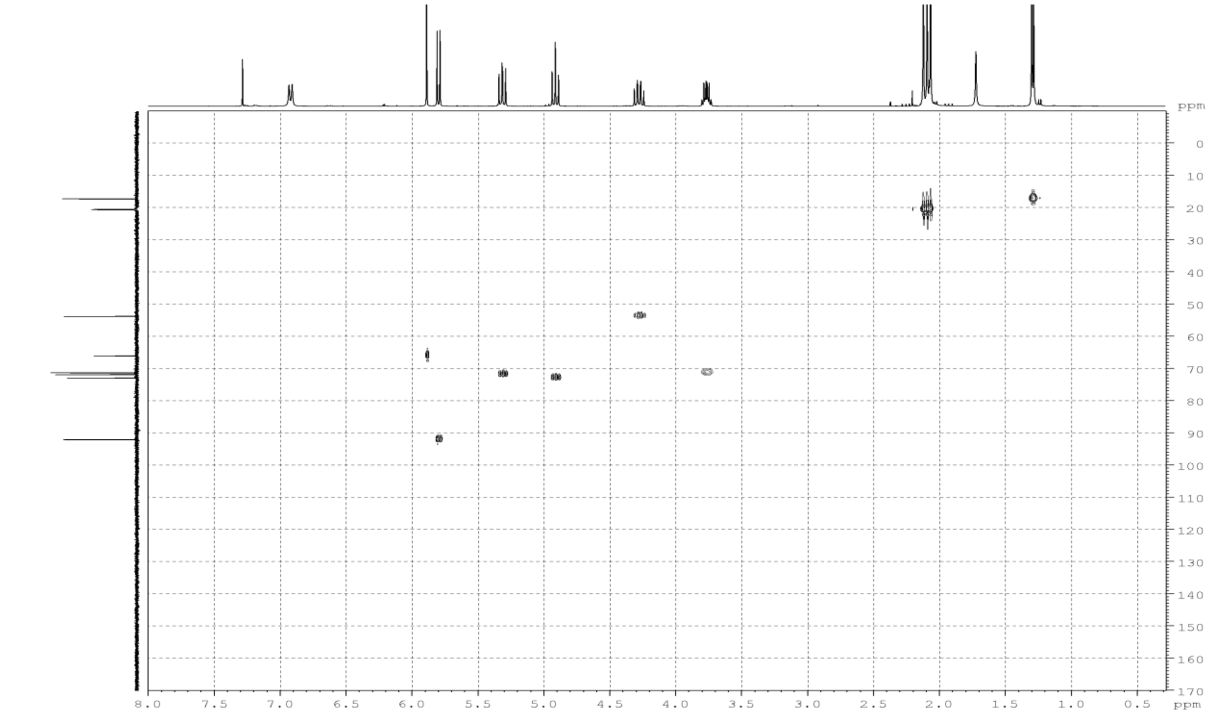

Figure. DEPT-HSQC NMR spectrum of **16**.

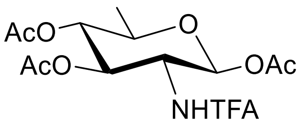

17

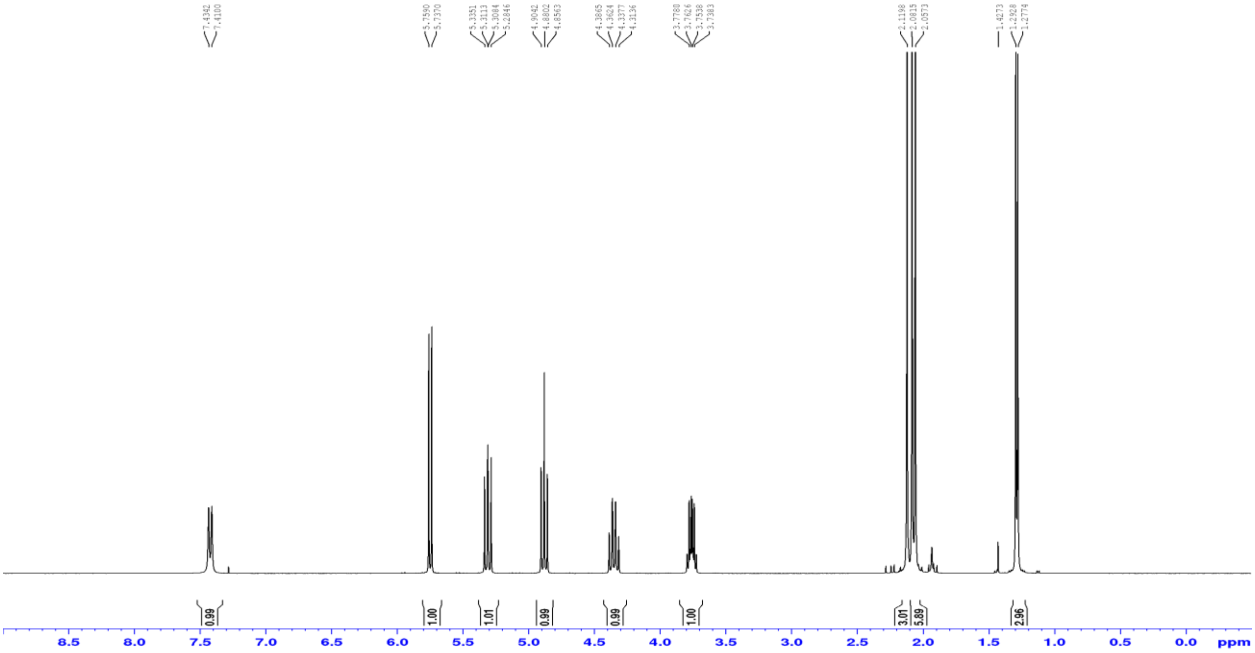

Figure. ^{1}H NMR (400 MHz, $CDCl_3$) spectrum of **17**.

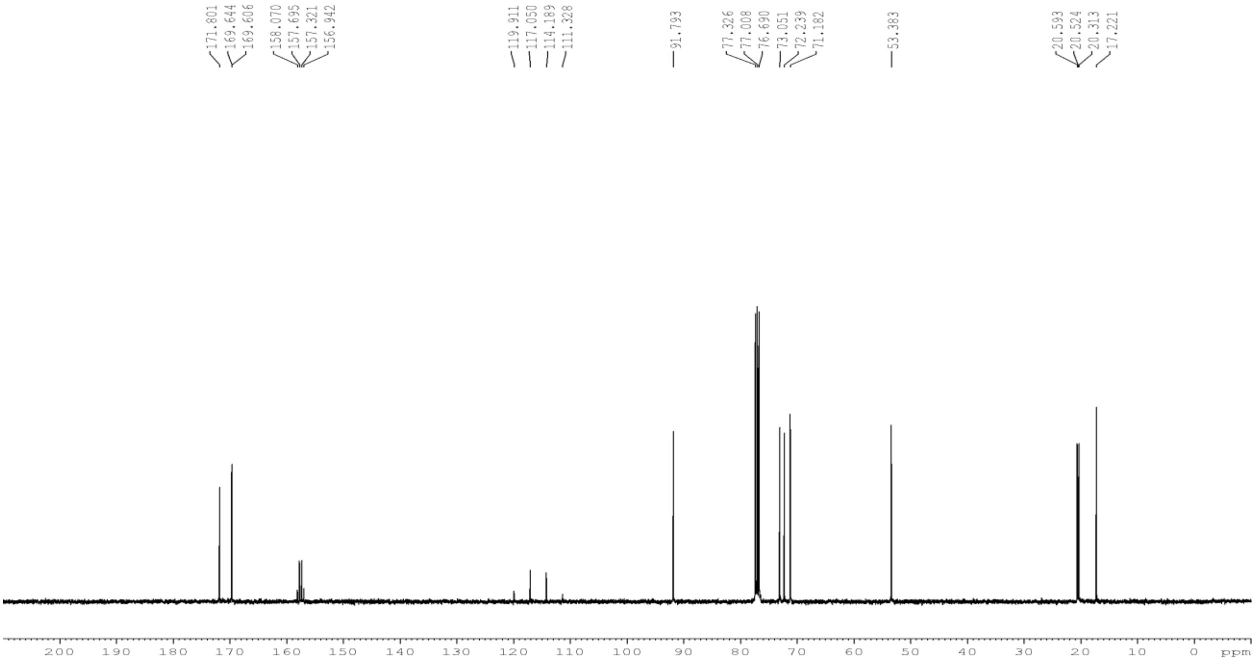

Figure. ^{13}C{^{1}H} NMR (100 MHz, $CDCl_3$) spectrum of **17**.

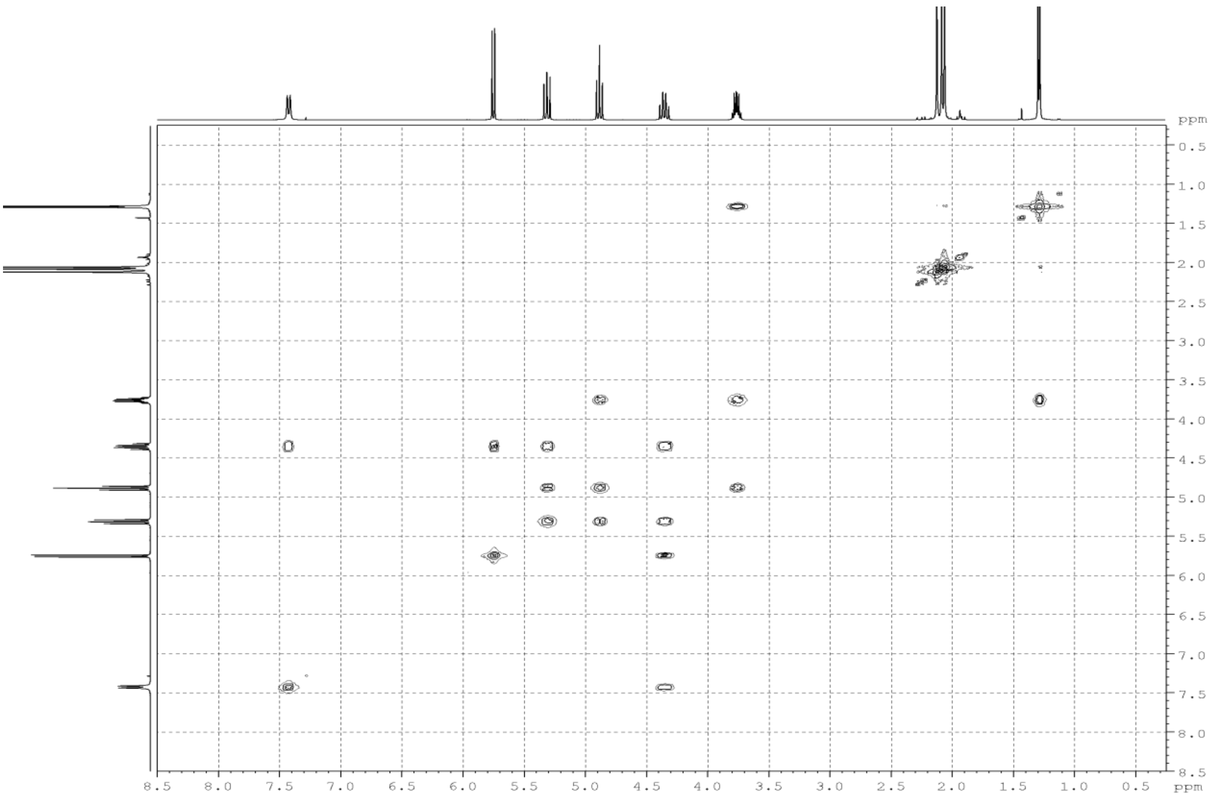

Figure. ^{1}H-^{1}H COSY NMR (400 MHz, $CDCl_3$) spectrum of **17**.

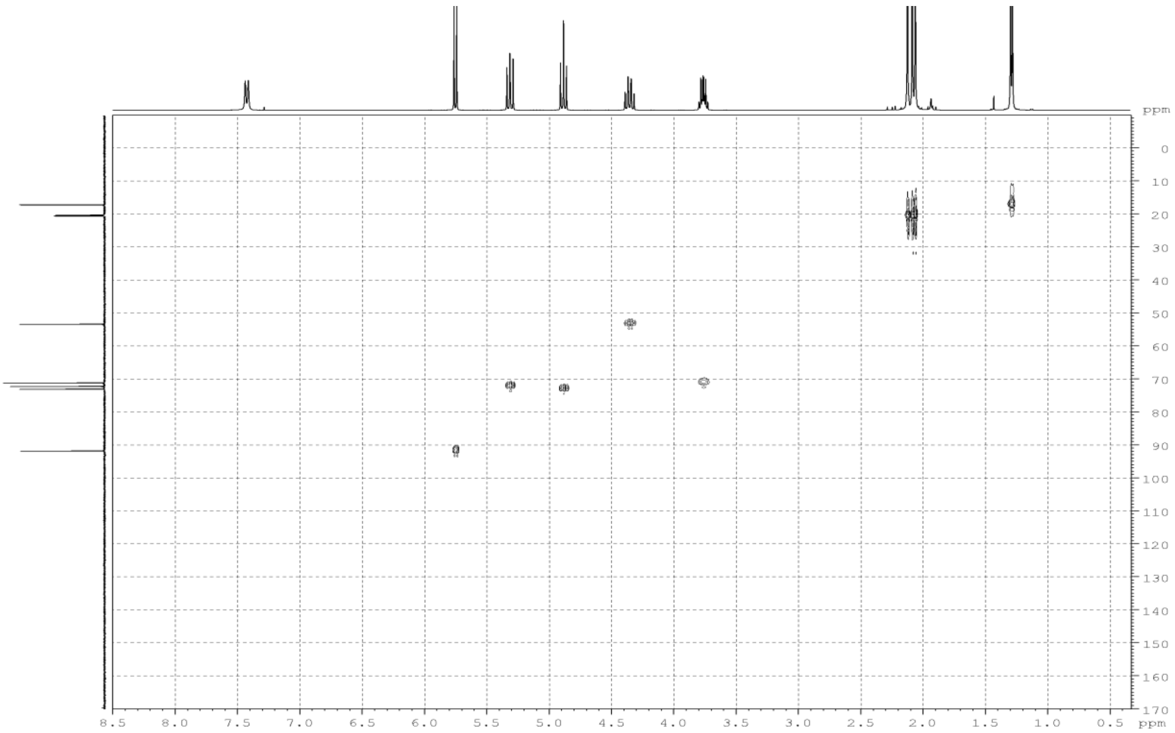

Figure. DEPT-HSQC NMR spectrum of **17**.

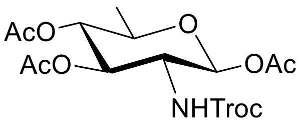

18

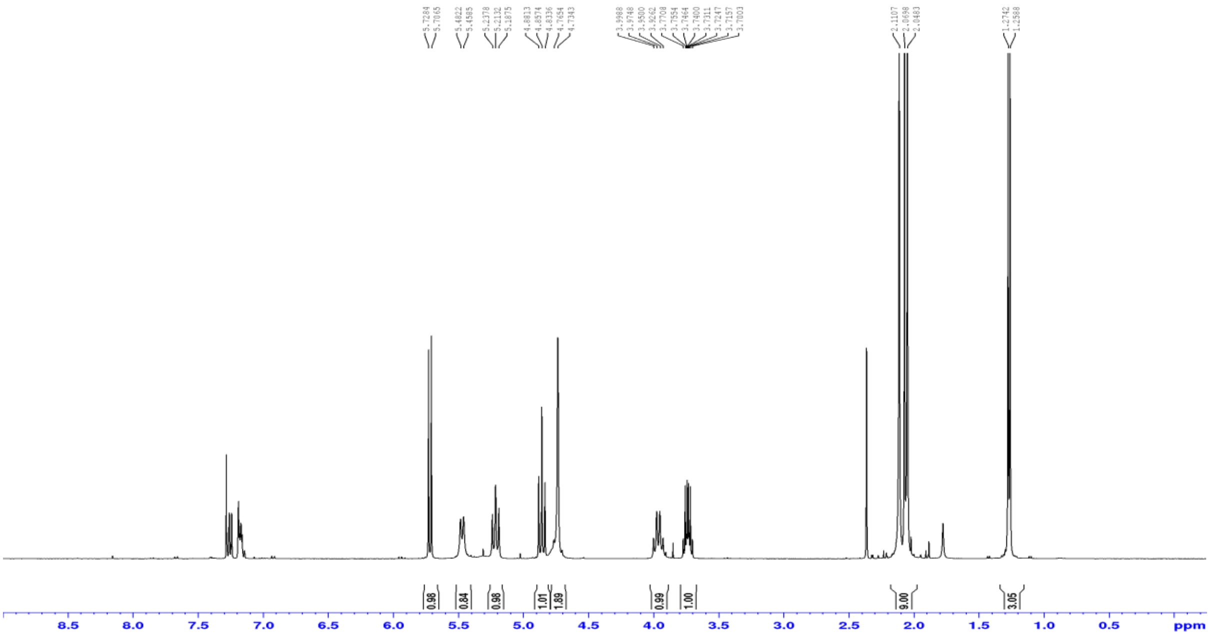

Figure. ^{1}H NMR (400 MHz, CDCl$_3$) spectrum of **18**.

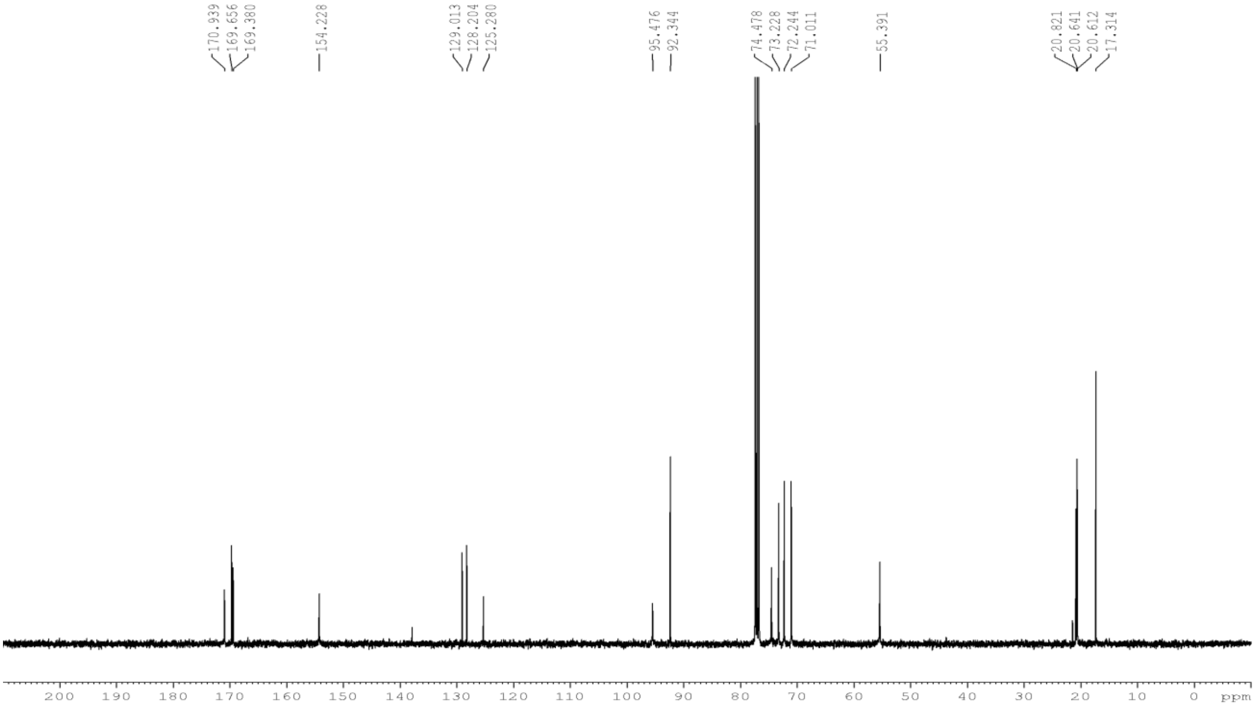

Figure. ^{13}C{^{1}H} NMR (100 MHz, CDCl$_3$) spectrum of **18**.

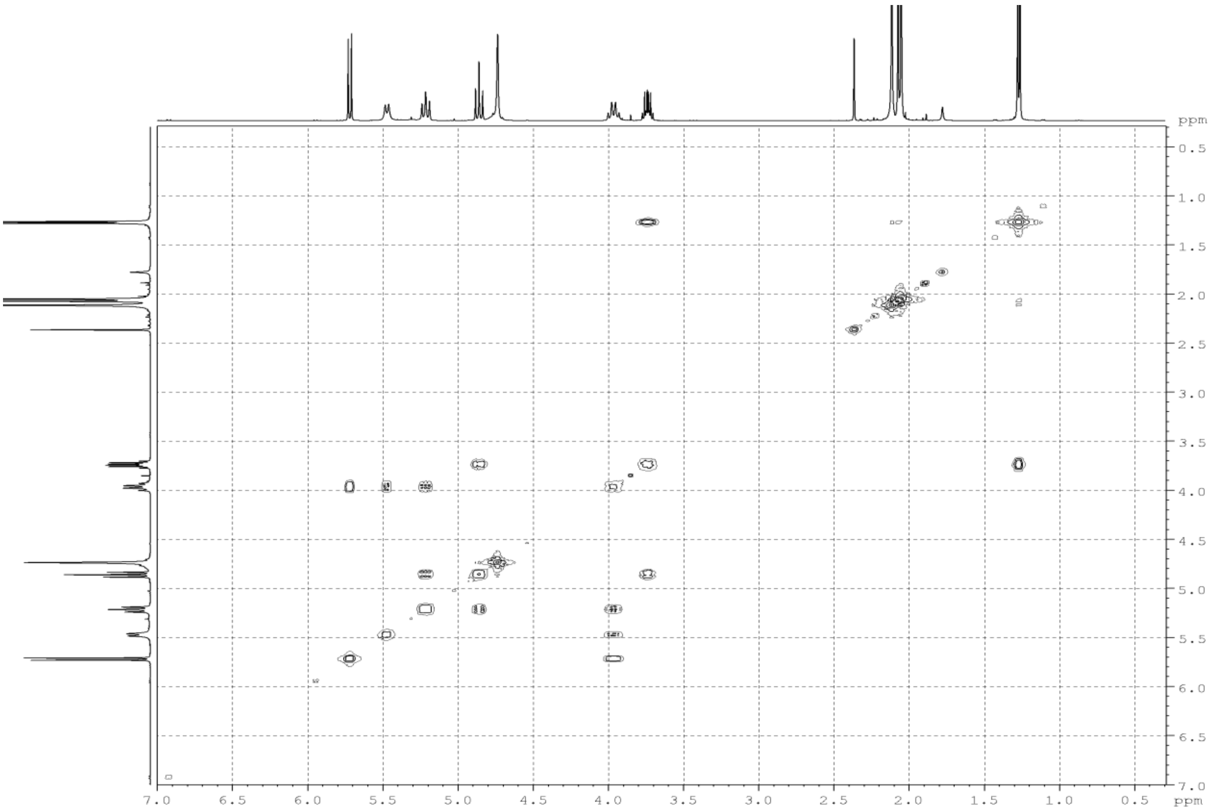

Figure. ^{1}H-^{1}H COSY NMR (400 MHz, $CDCl_3$) spectrum of **18**.

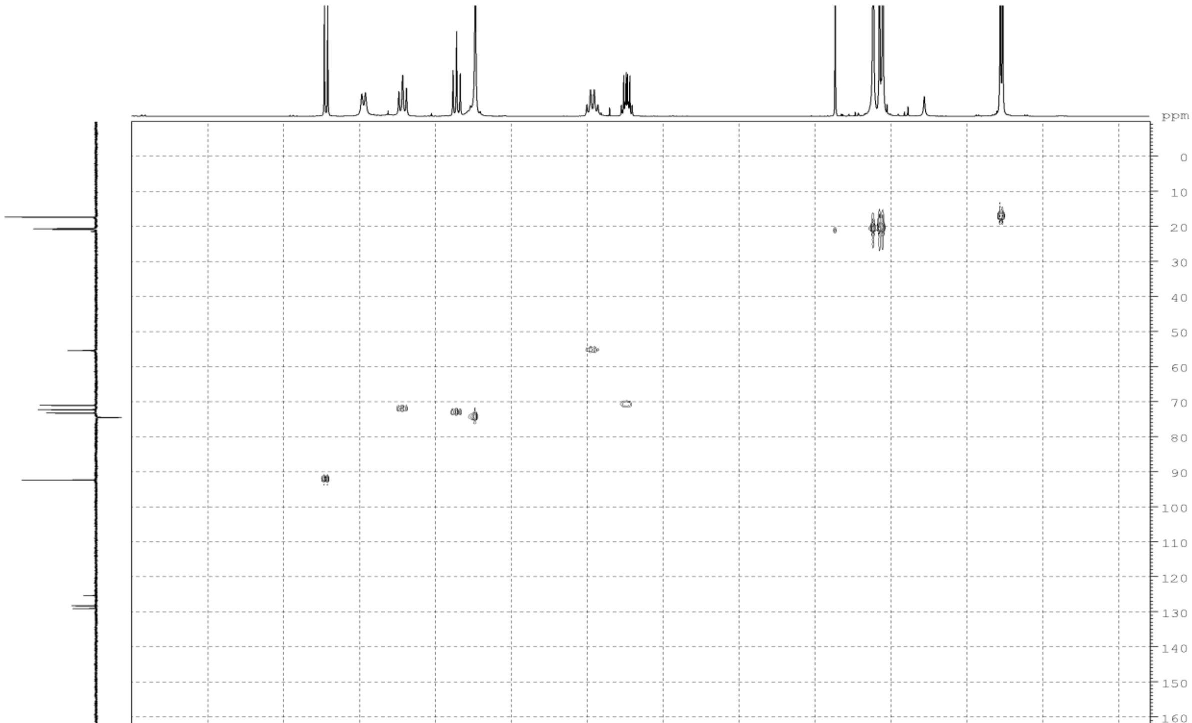

Figure. DEPT-HSQC NMR spectrum of **18**.

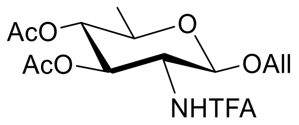

19

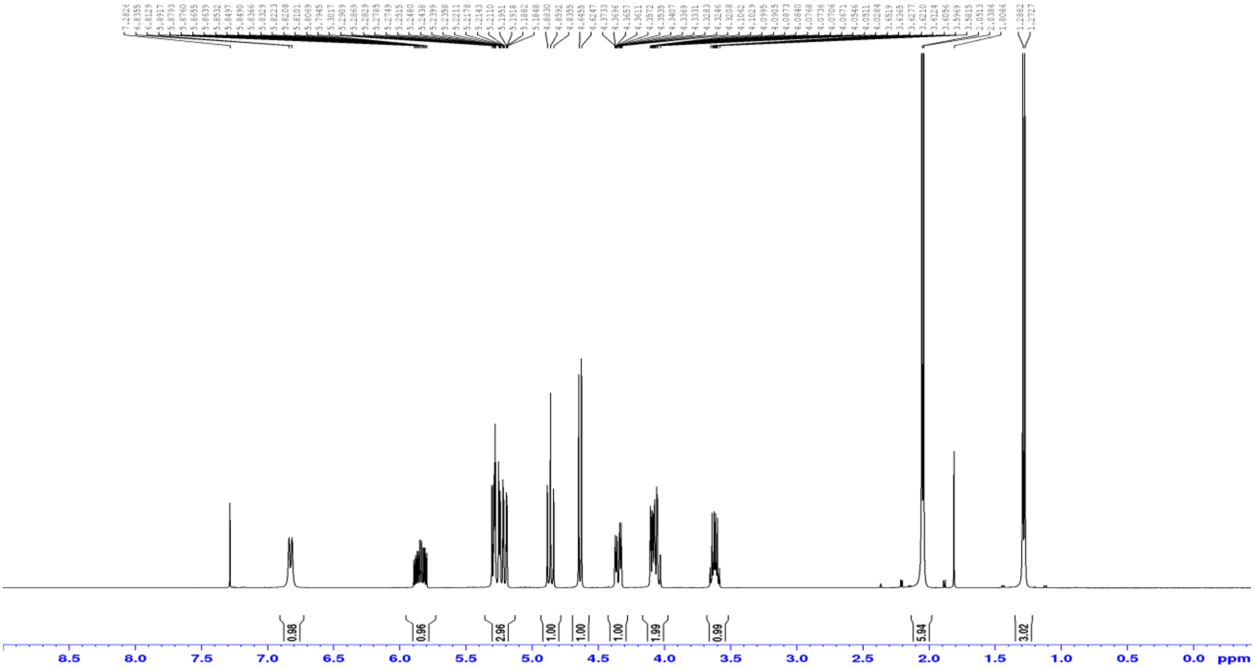

Figure. ^{1}H NMR (400 MHz, $CDCl_3$) spectrum of **19**.

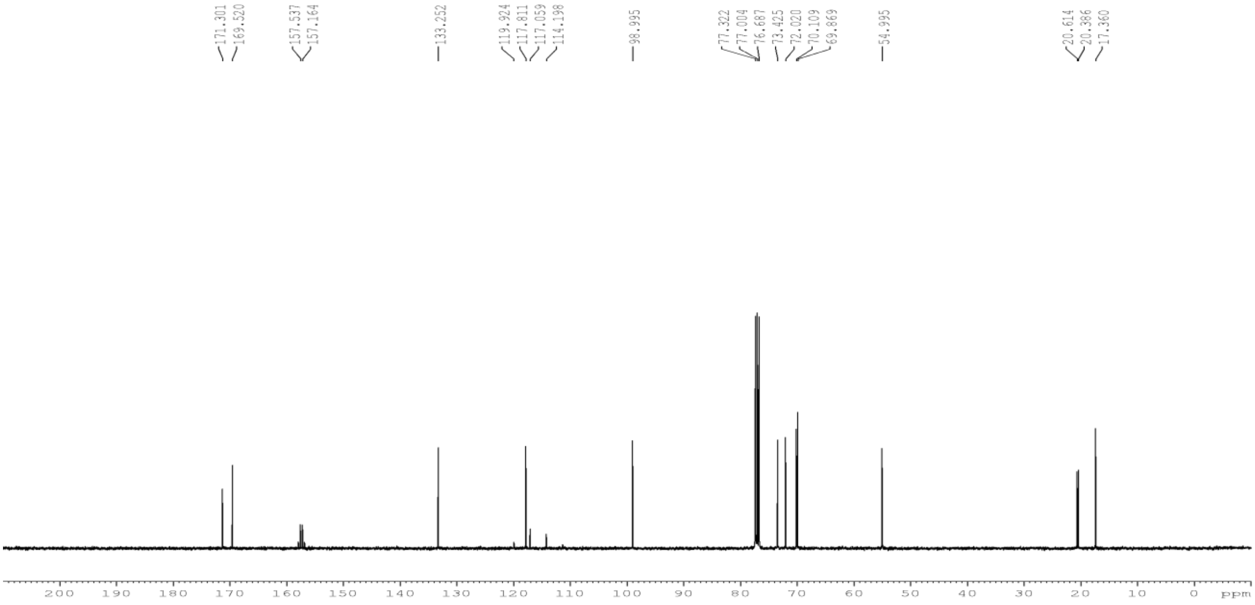

Figure. ^{13}C{^{1}H} NMR (100 MHz, $CDCl_3$) spectrum of **19**.

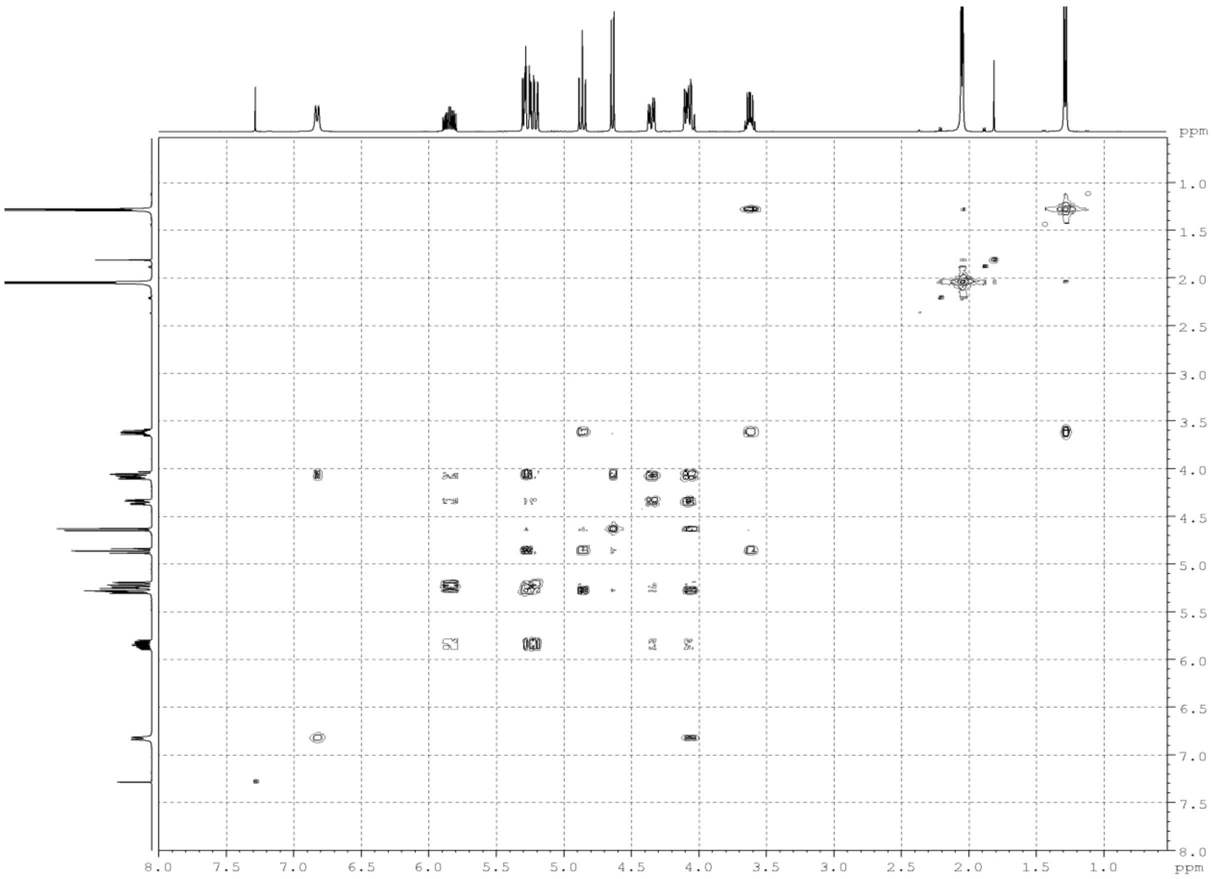

Figure. ^{1}H-^{1}H COSY NMR (400 MHz, $CDCl_3$) spectrum of **19**.

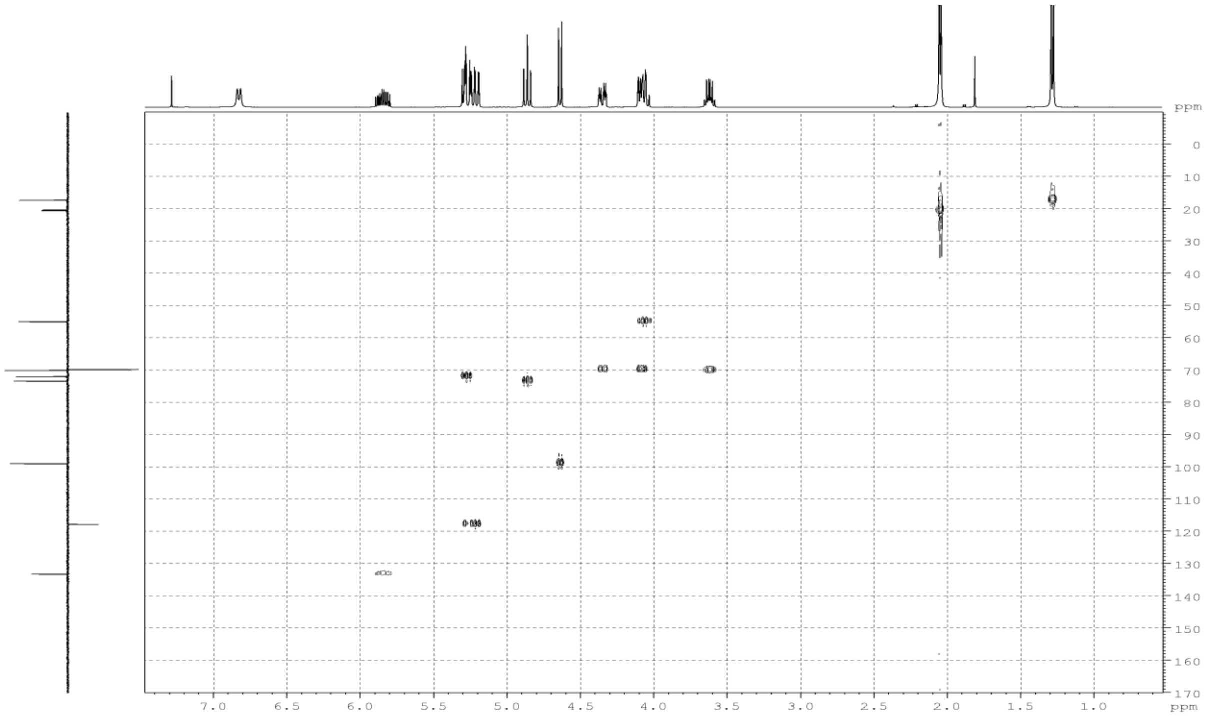

Figure. DEPT-HSQC NMR spectrum of **19**.

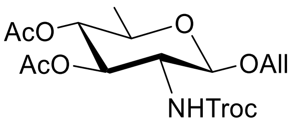

20

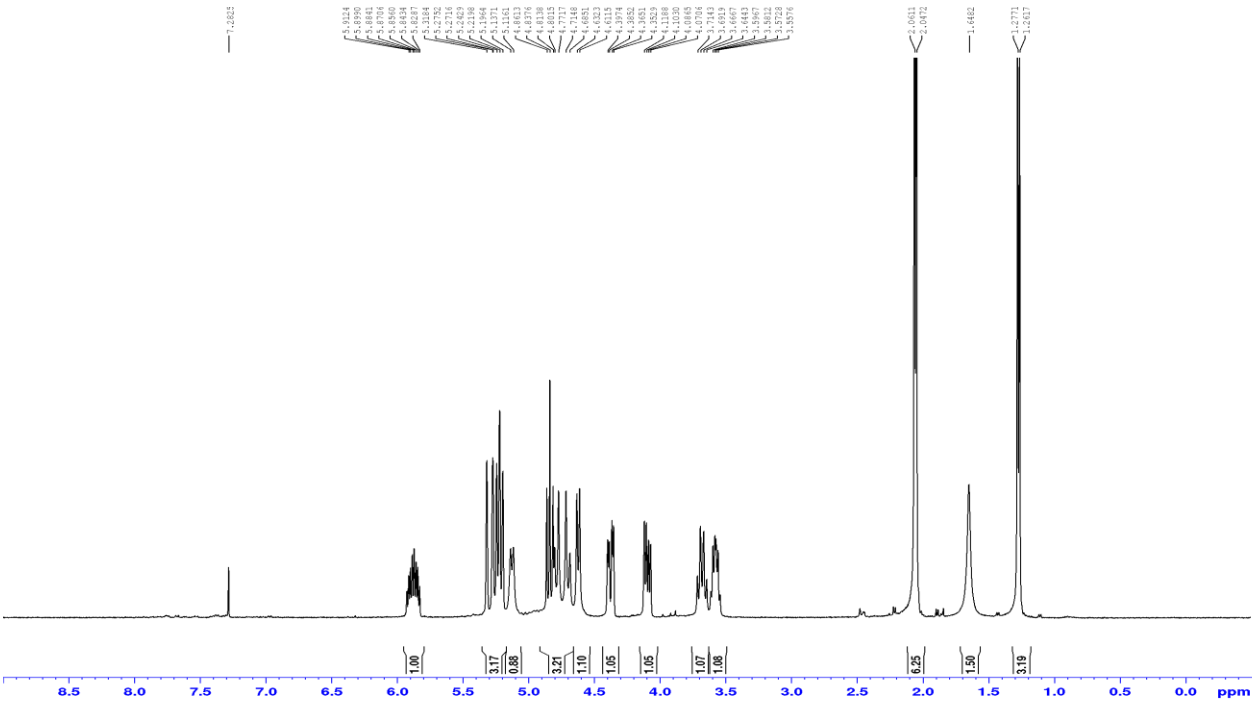

Figure. ^{1}H NMR (400 MHz, $CDCl_3$) spectrum of **20**.

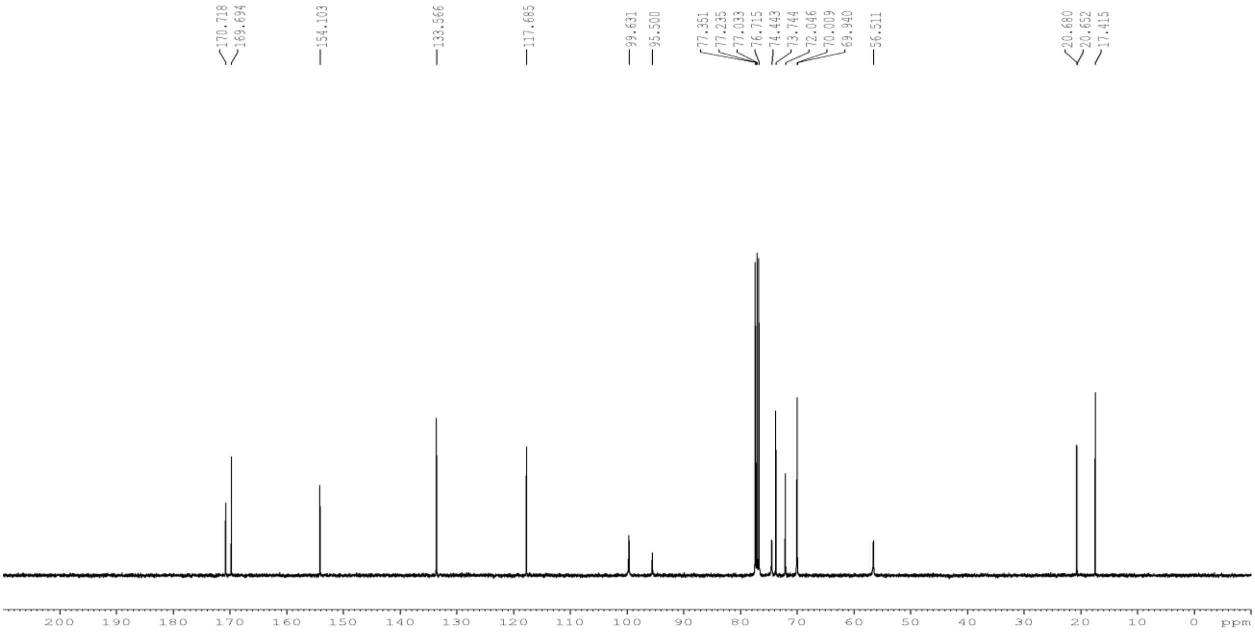

Figure. $^{13}C\{^{1}H\}$ NMR (100 MHz, $CDCl_3$) spectrum of **20**.

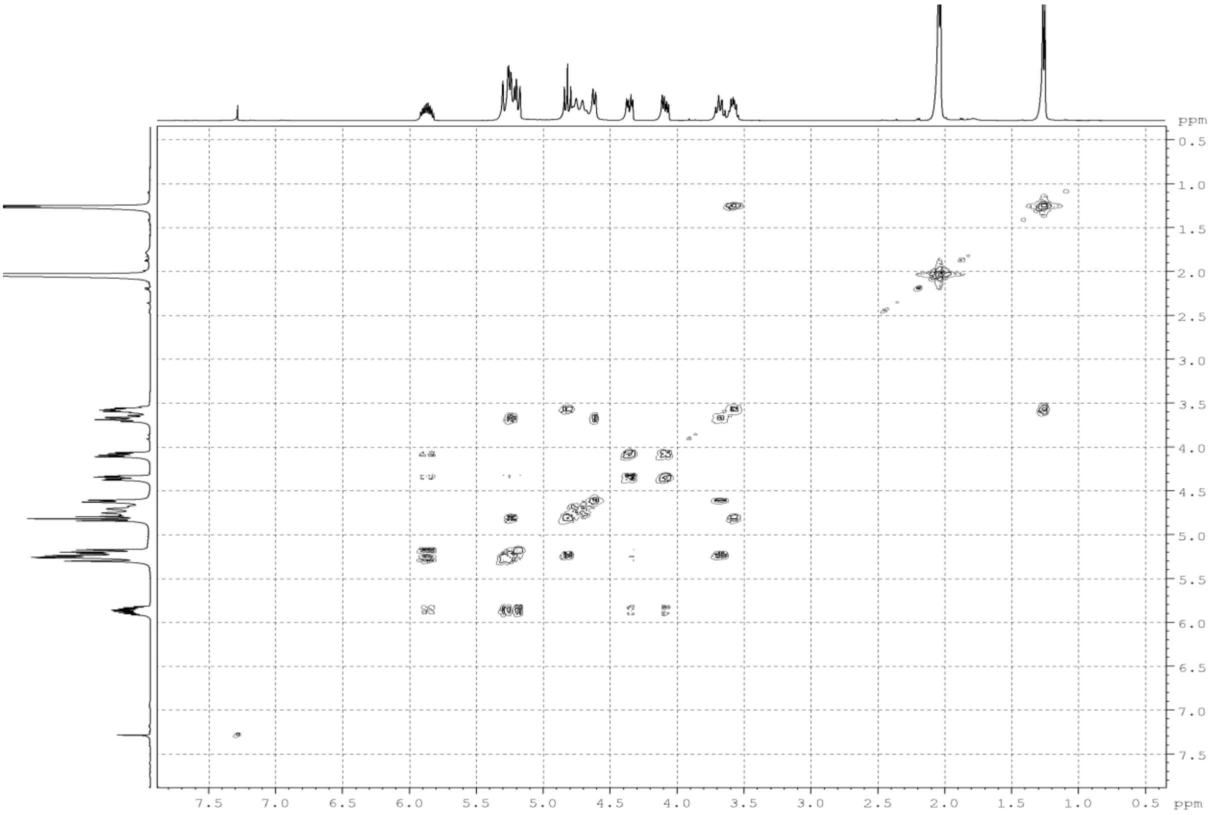

Figure. ^{1}H-^{1}H COSY NMR (400 MHz, $CDCl_3$) spectrum of **20**.

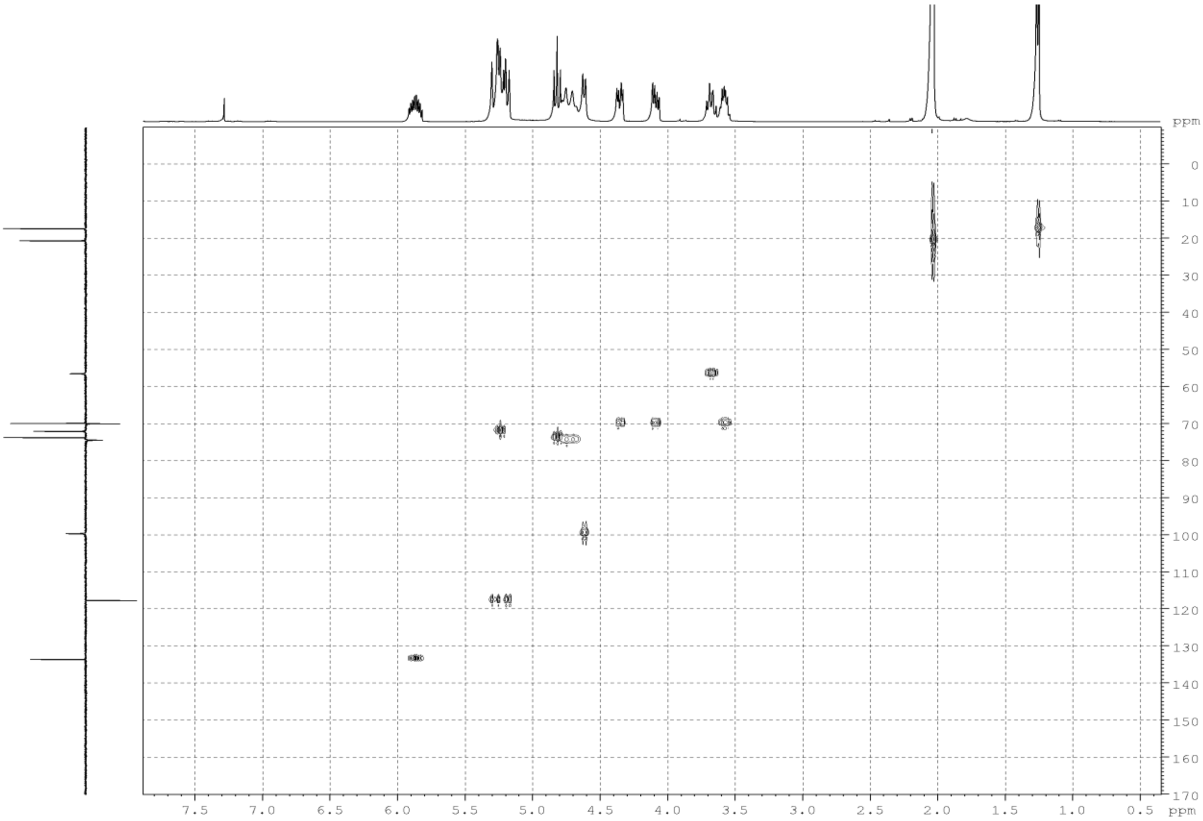

Figure. DEPT-HSQC NMR spectrum of **20**.

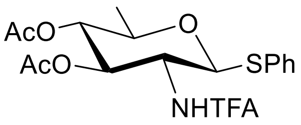

21

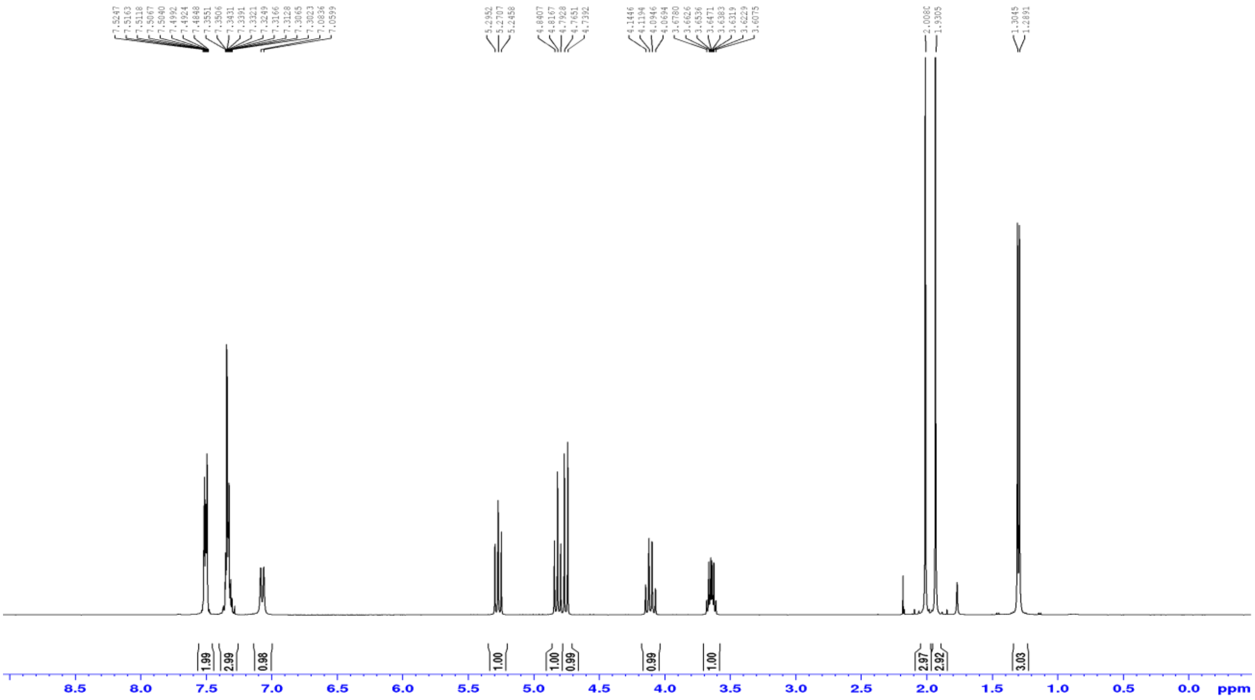

Figure. ^{1}H NMR (400 MHz, $CDCl_3$) spectrum of **21**.

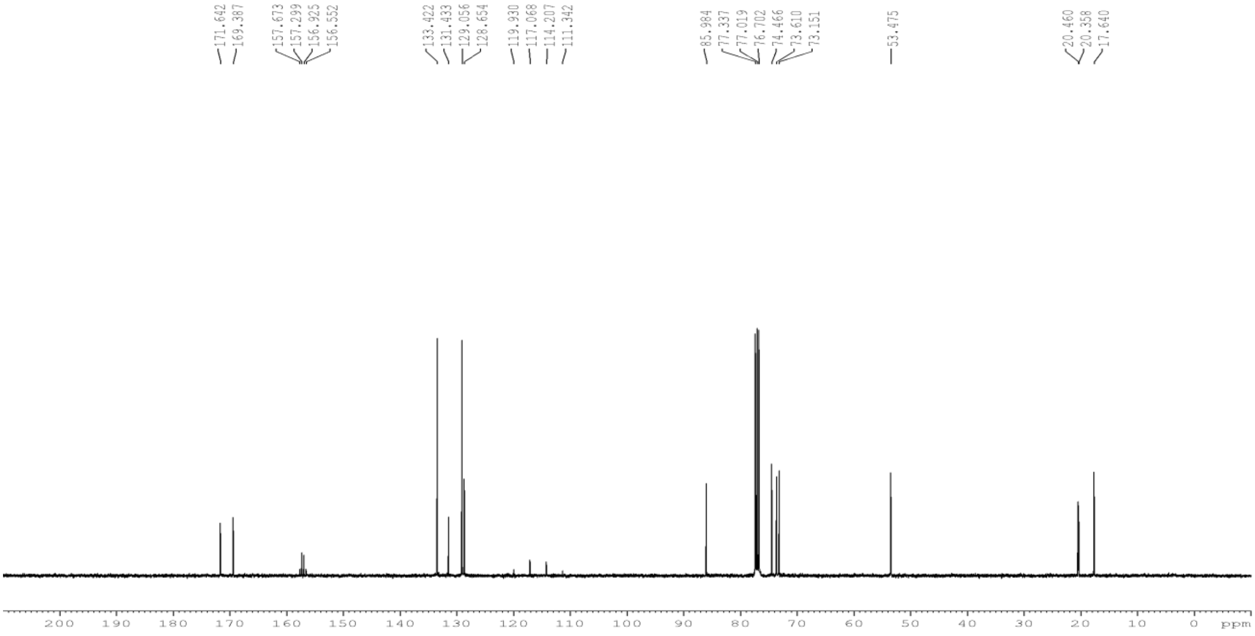

Figure. $^{13}C\{^{1}H\}$ NMR (100 MHz, $CDCl_3$) spectrum of **21**.

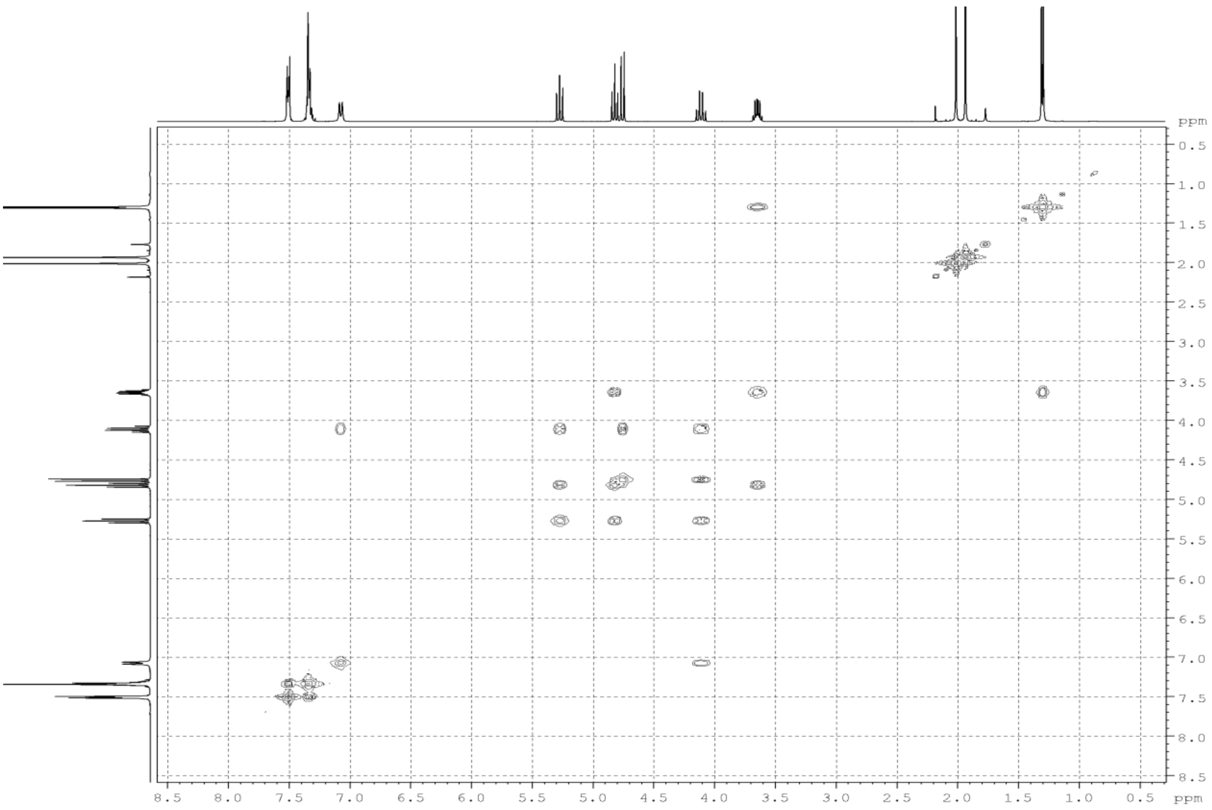

Figure. ^{1}H-^{1}H COSY NMR (400 MHz, $CDCl_3$) spectrum of **21**.

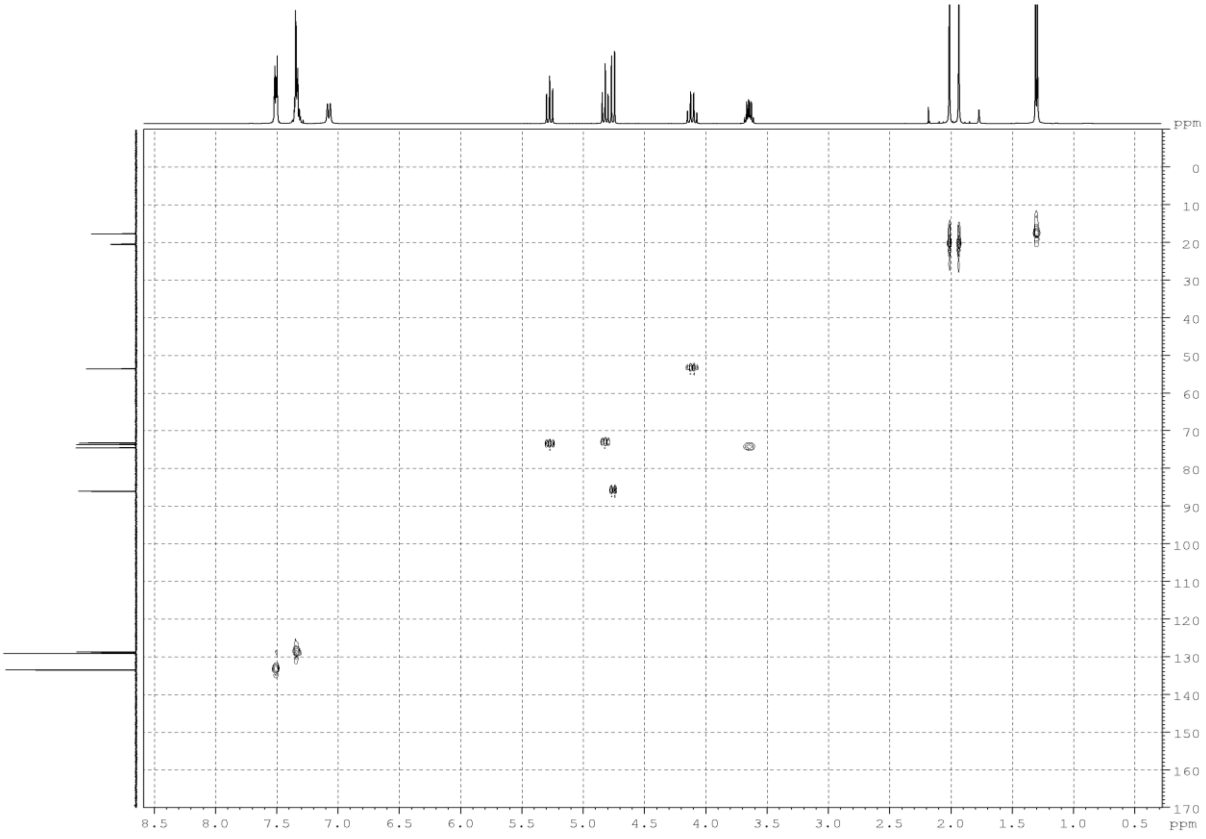

Figure. DEPT-HSQC NMR spectrum of **21**.

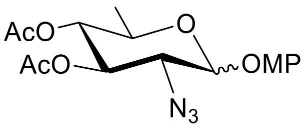

23

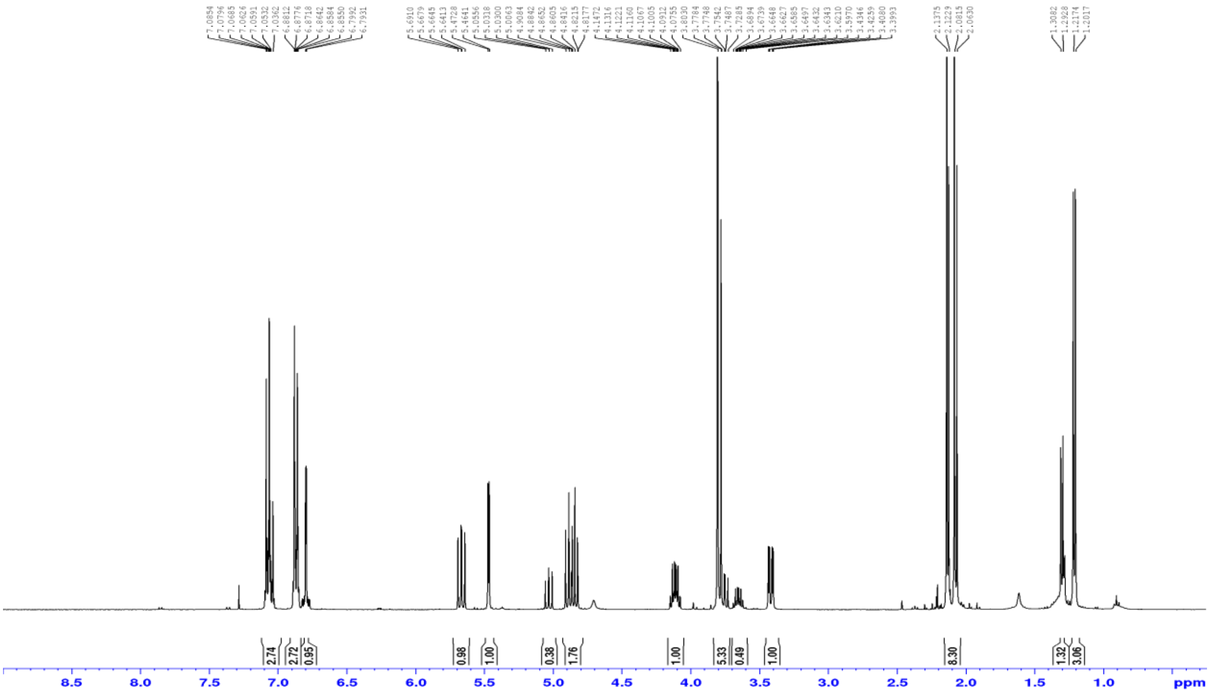

Figure. ^{1}H NMR (400 MHz, CDCl$_3$) spectrum of **23**.

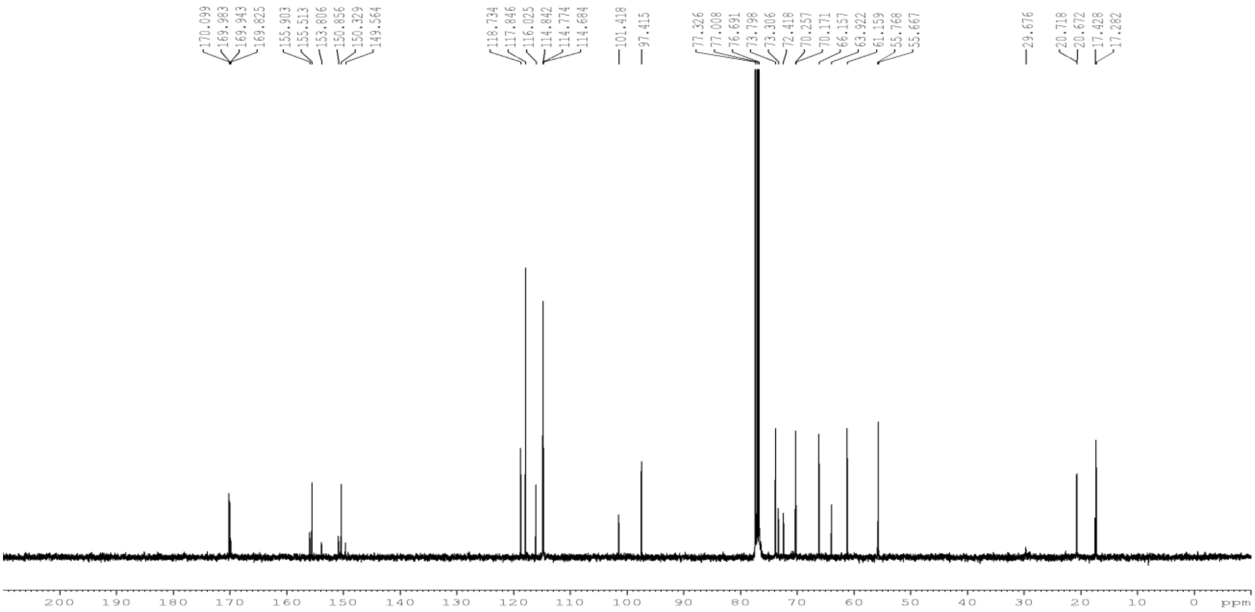

Figure. ^{13}C{^{1}H} NMR (100 MHz, CDCl$_3$) spectrum of **23**.

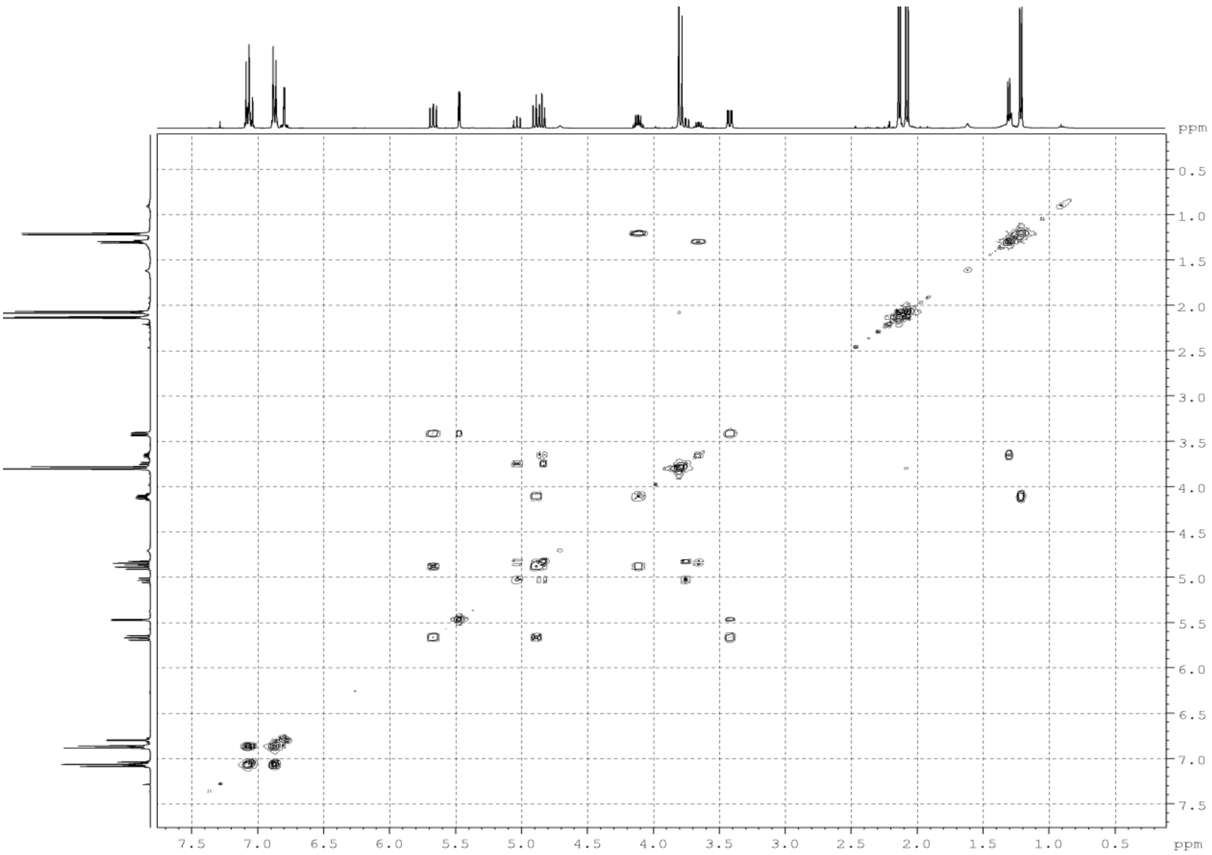

Figure. ^{1}H-^{1}H COSY NMR (400 MHz, $CDCl_3$) spectrum of **23**.

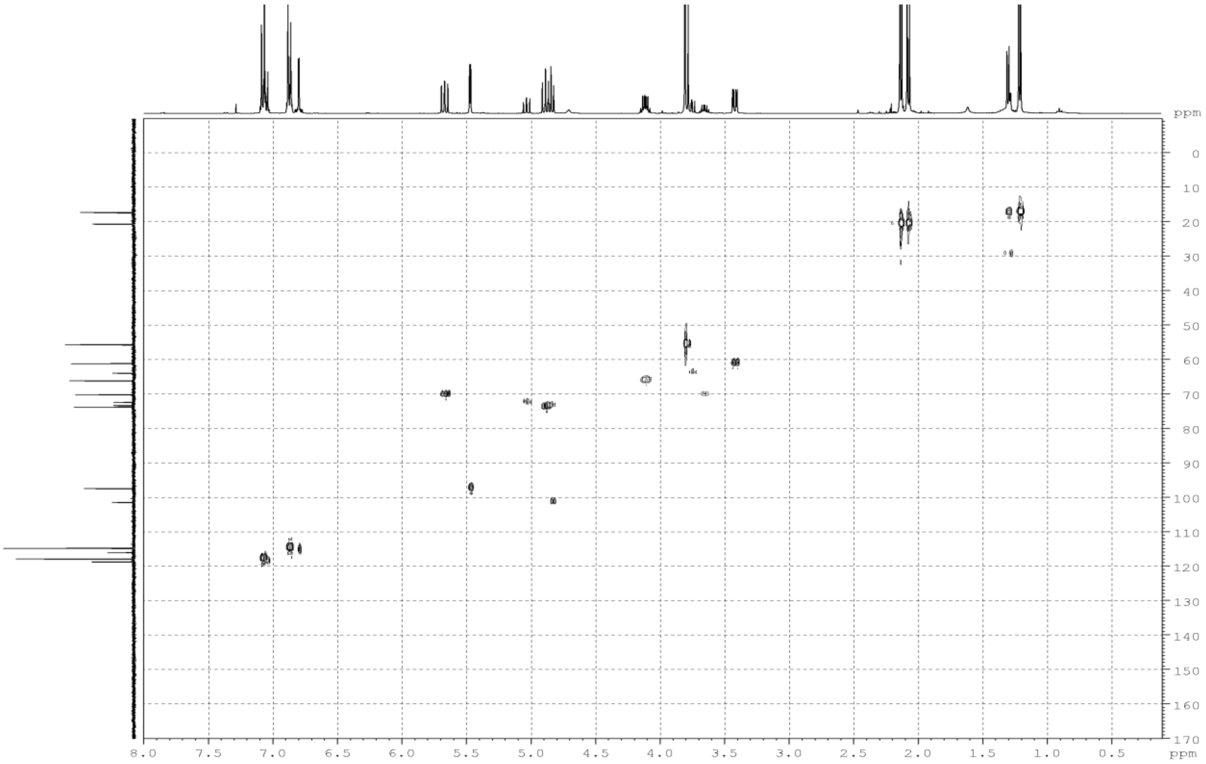

Figure. DEPT-HSQC NMR spectrum of **23**.

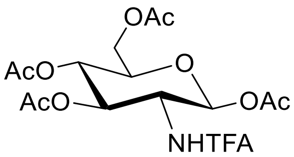

24

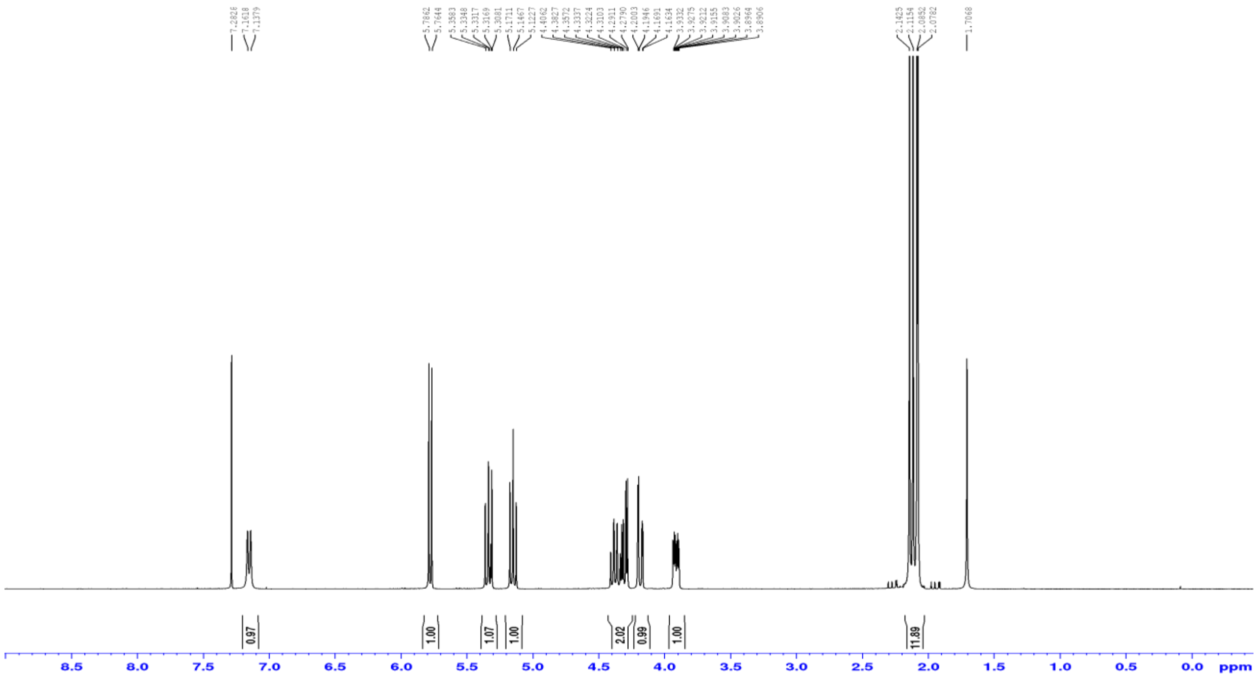

Figure. ^{1}H NMR (400 MHz, $CDCl_3$) spectrum of **24**.

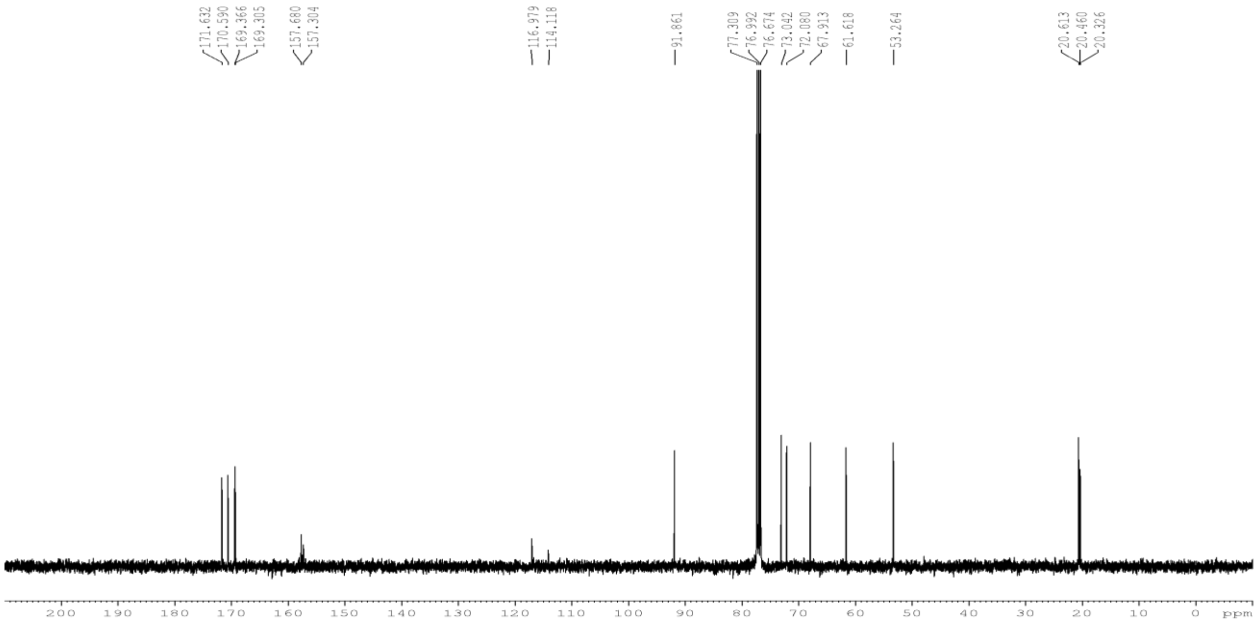

Figure. $^{13}C\{^{1}H\}$ NMR (100 MHz, $CDCl_3$) spectrum of **24**.

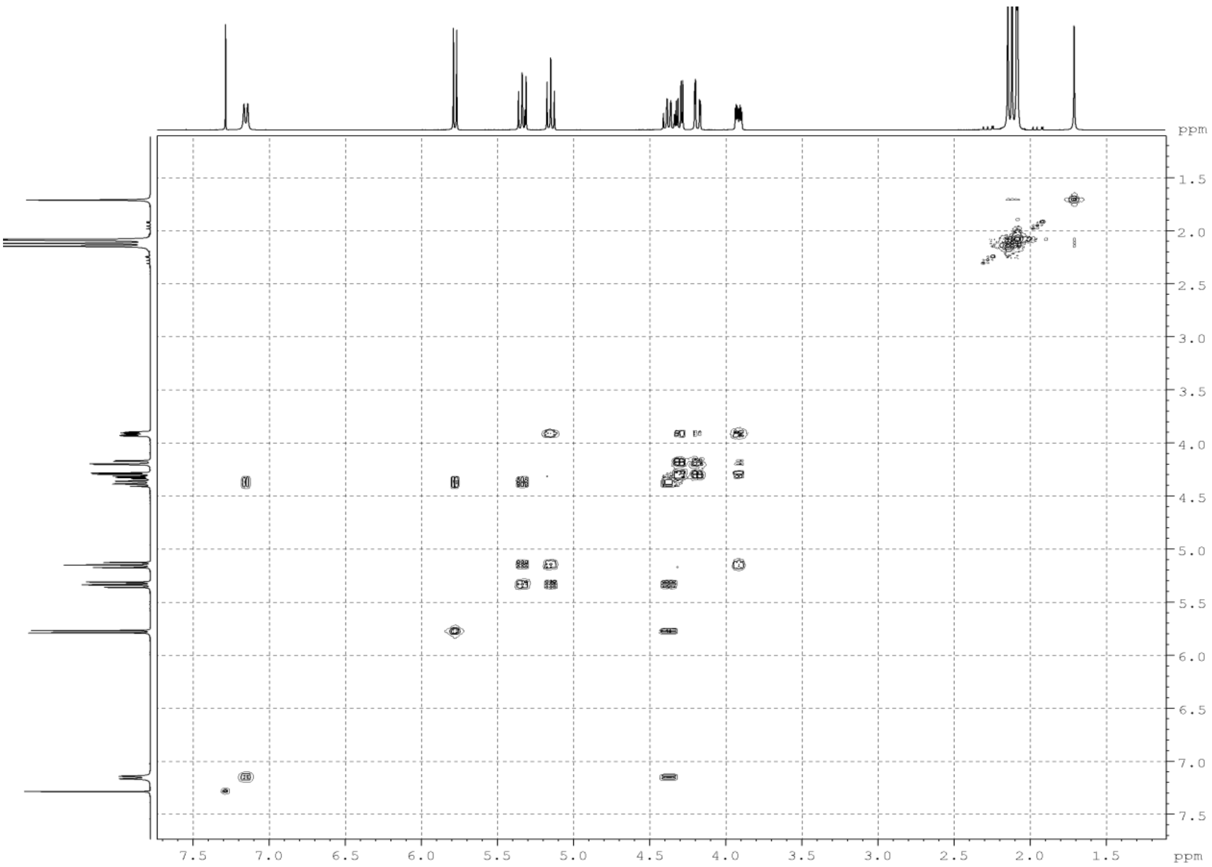

Figure. ^{1}H-^{1}H COSY NMR (400 MHz, $CDCl_3$) spectrum of **24**.

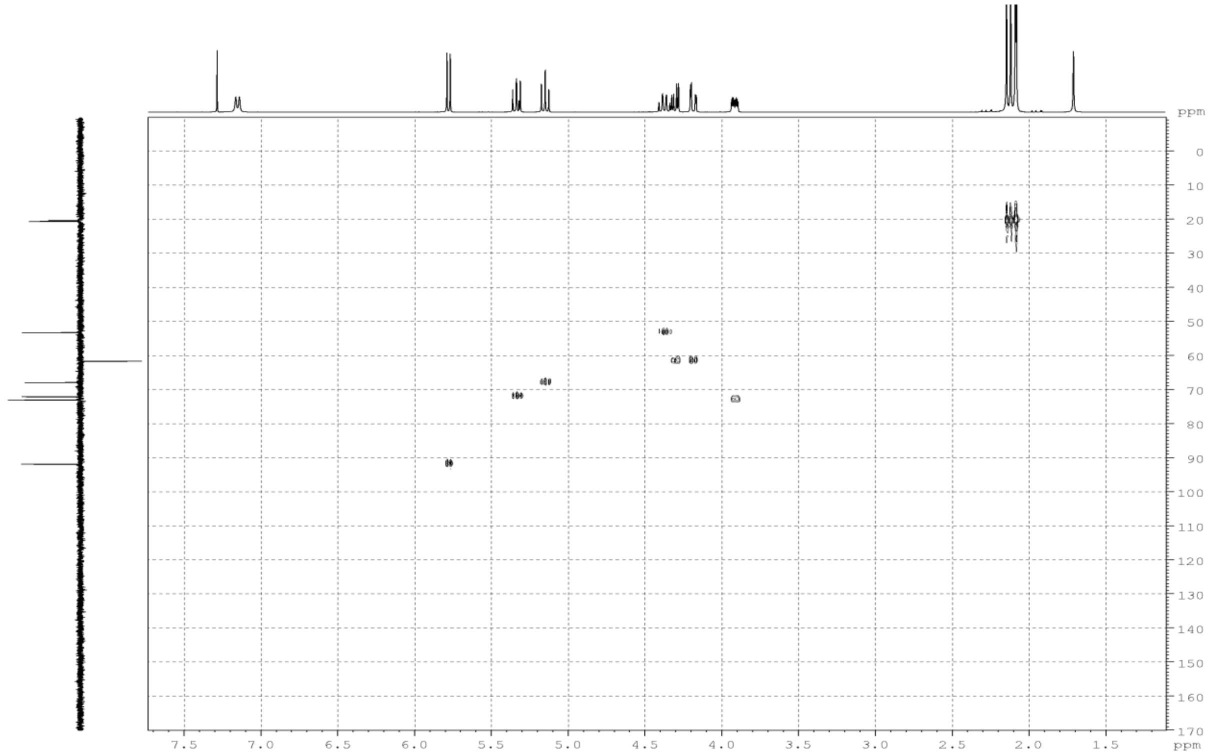

Figure. DEPT-HSQC NMR spectrum of **24**.

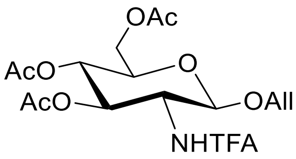

25

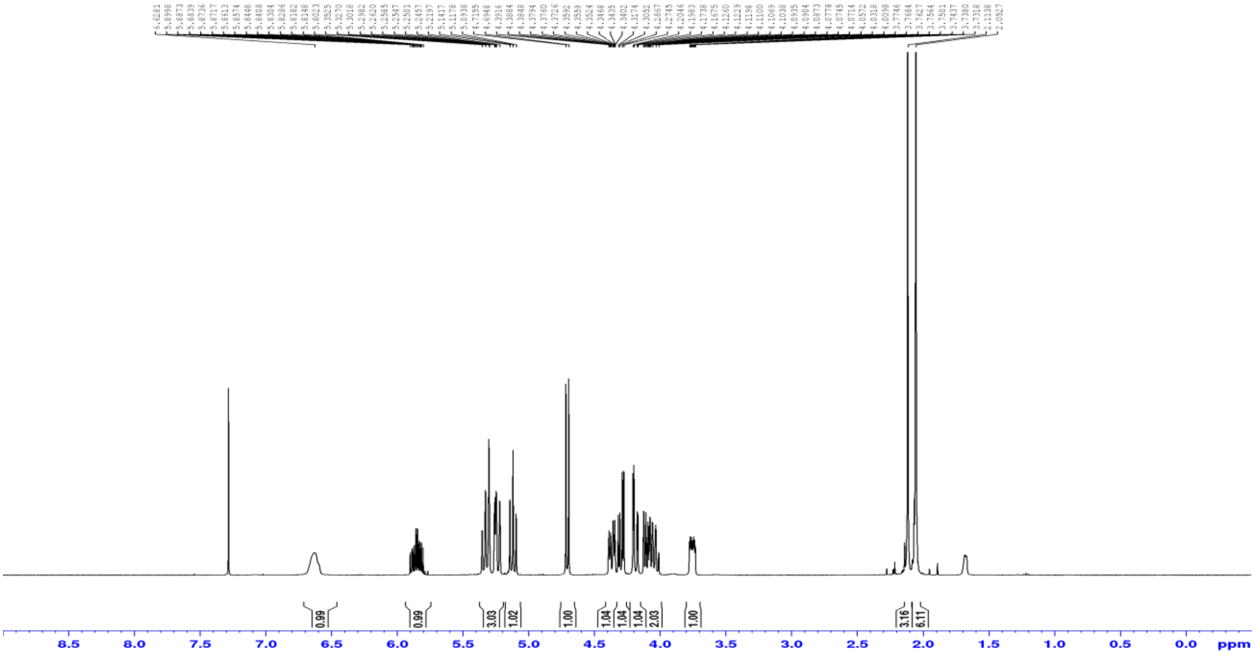

Figure. ^{1}H NMR (400 MHz, $CDCl_3$) spectrum of **25**.

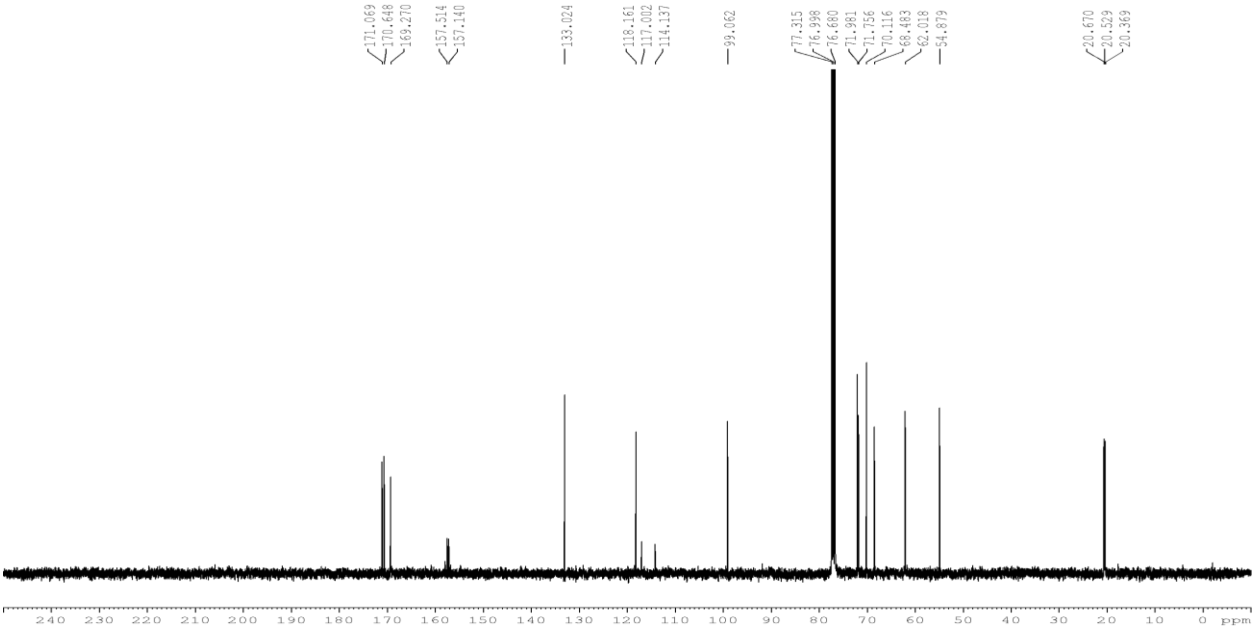

Figure. $^{13}C\{^{1}H\}$ NMR (100 MHz, $CDCl_3$) spectrum of **25**.

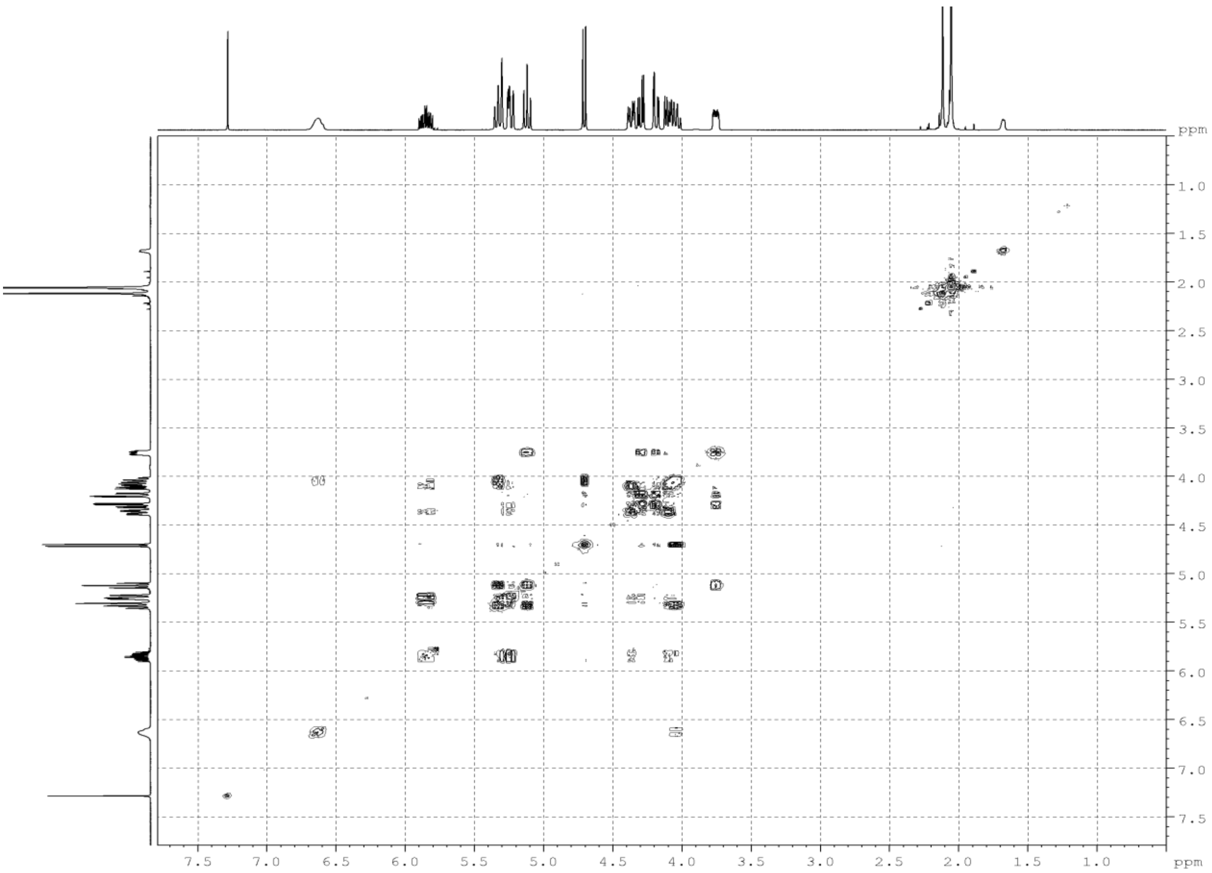

Figure. ^{1}H-^{1}H COSY NMR (400 MHz, $CDCl_3$) spectrum of **25**.

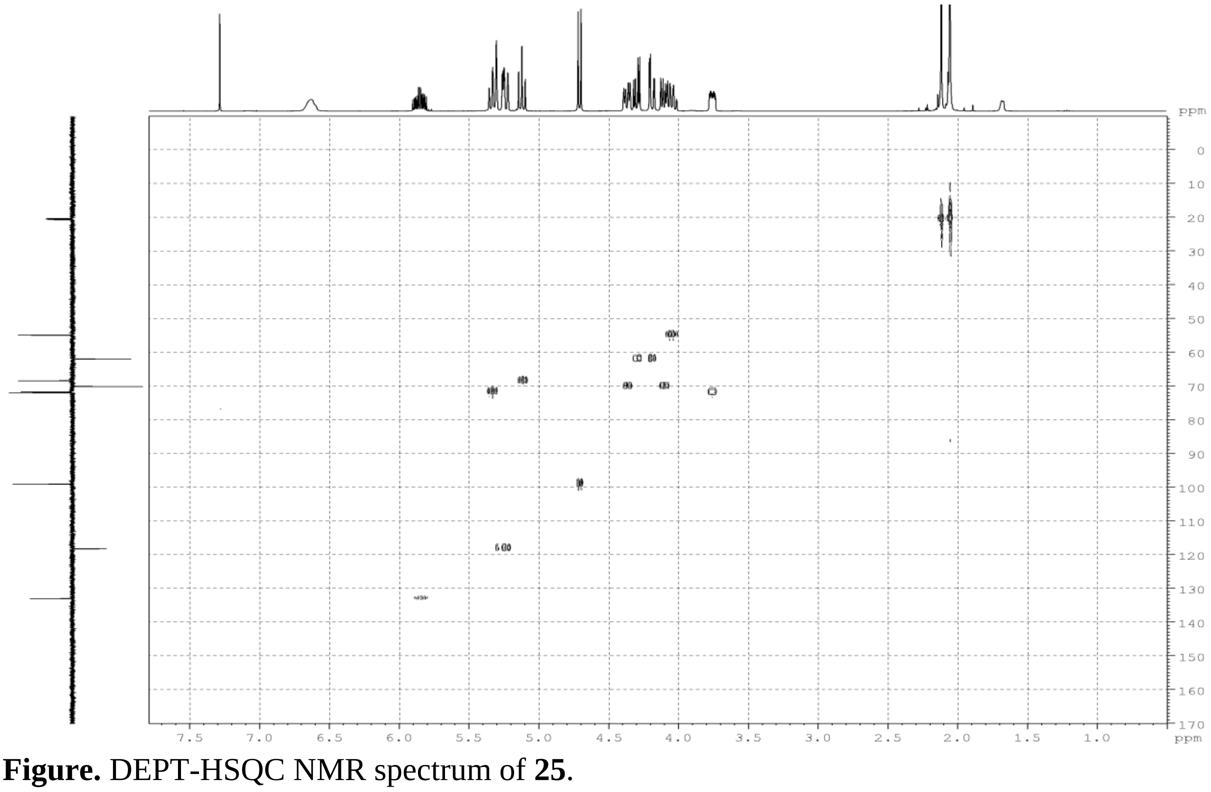

Figure. DEPT-HSQC NMR spectrum of **25**.

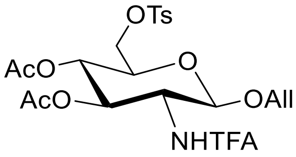

26

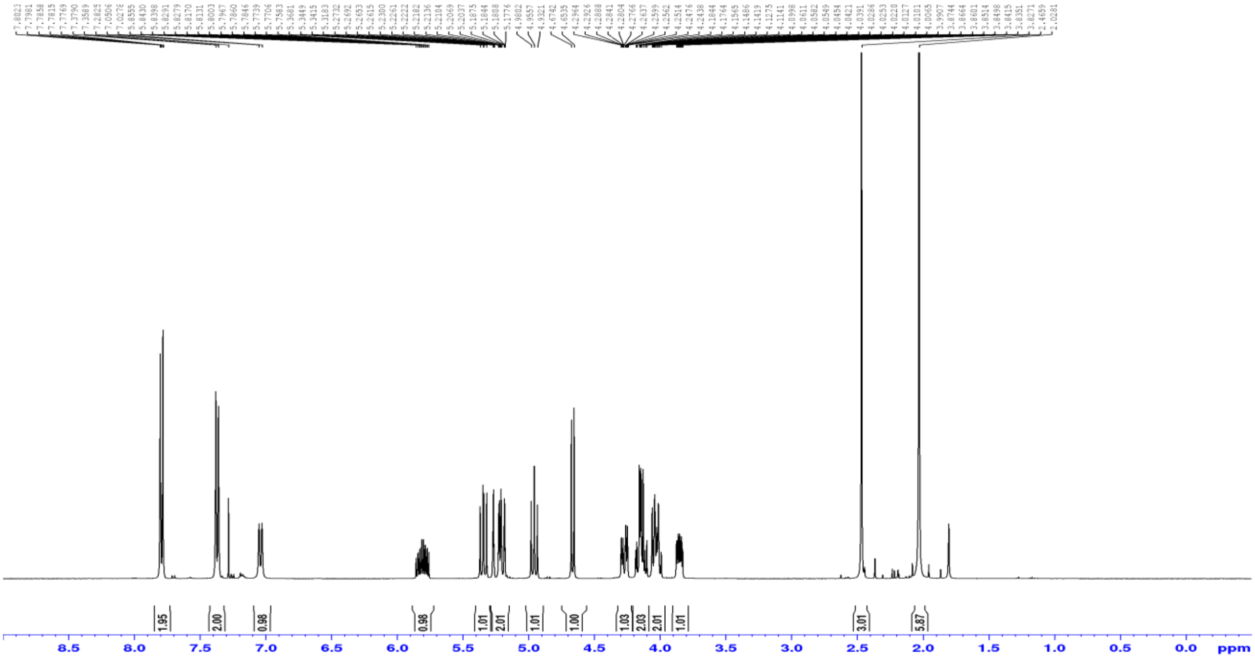

Figure. ^{1}H NMR (400 MHz, $CDCl_3$) spectrum of **26**.

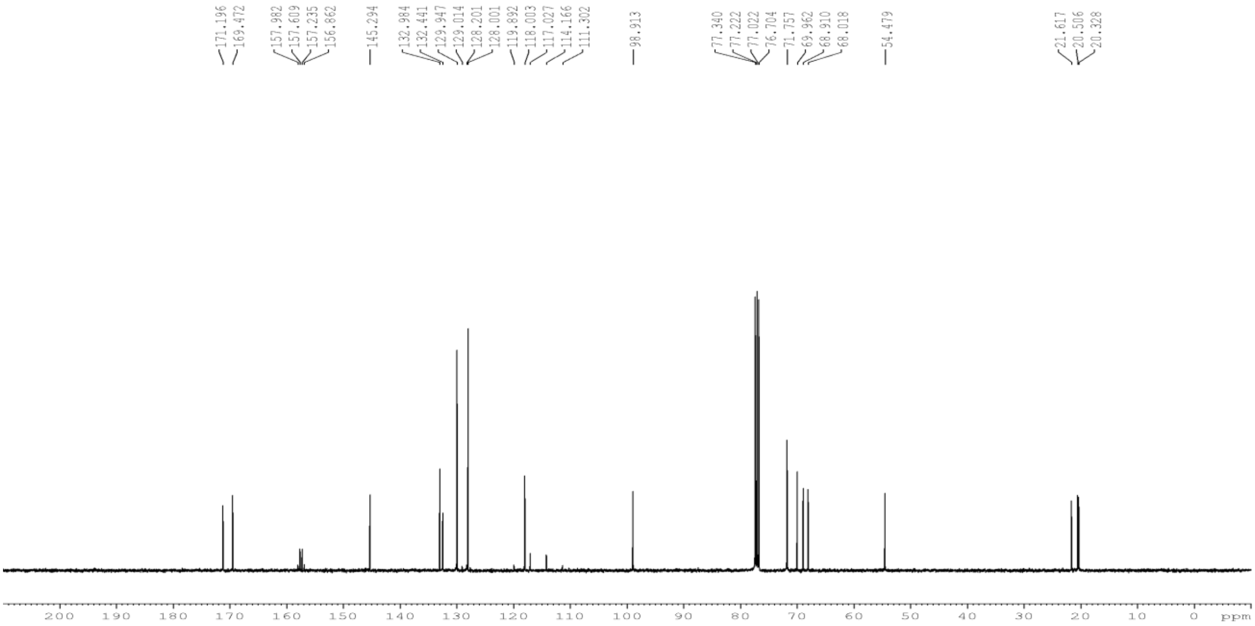

Figure. $^{13}C\{^{1}H\}$ NMR (100 MHz, $CDCl_3$) spectrum of **26**.

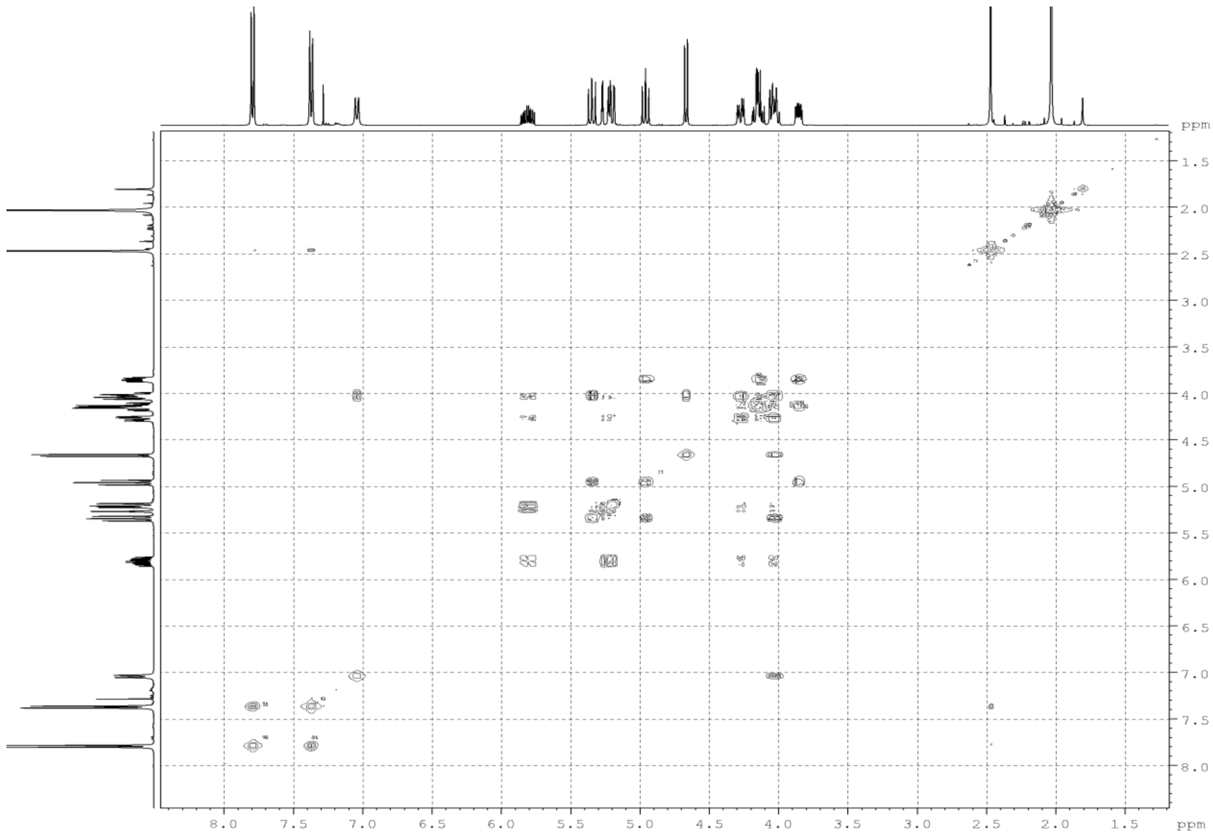

Figure. ^{1}H-^{1}H COSY NMR (400 MHz, $CDCl_3$) spectrum of **26**.

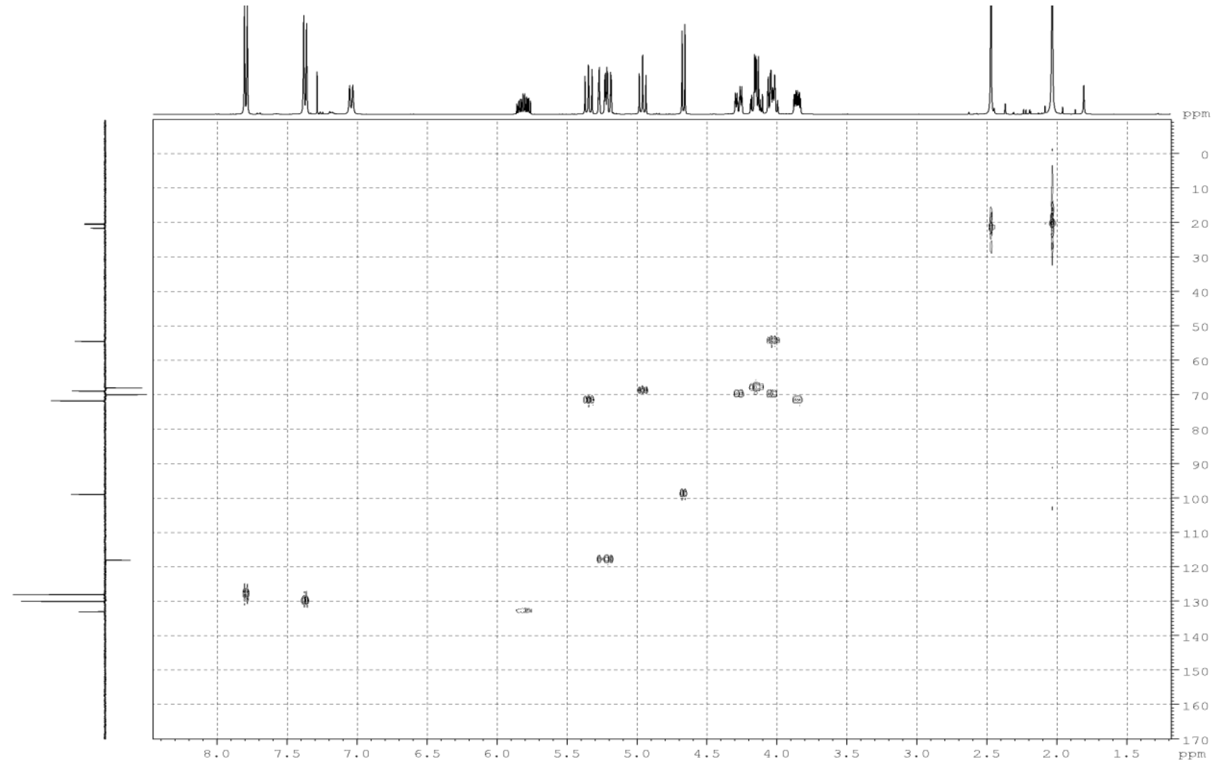

Figure. DEPT-HSQC NMR spectrum of **26**.

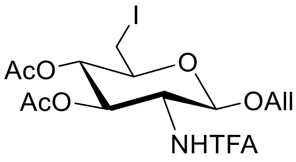

27

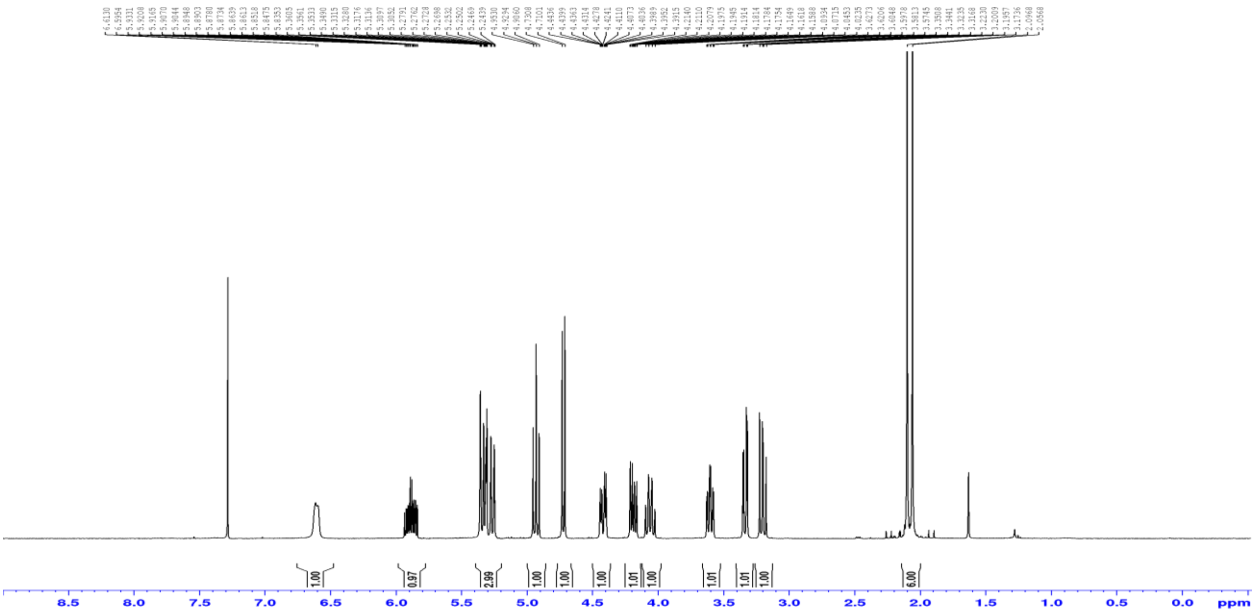

Figure. ^{1}H NMR (400 MHz, $CDCl_3$) spectrum of **27**.

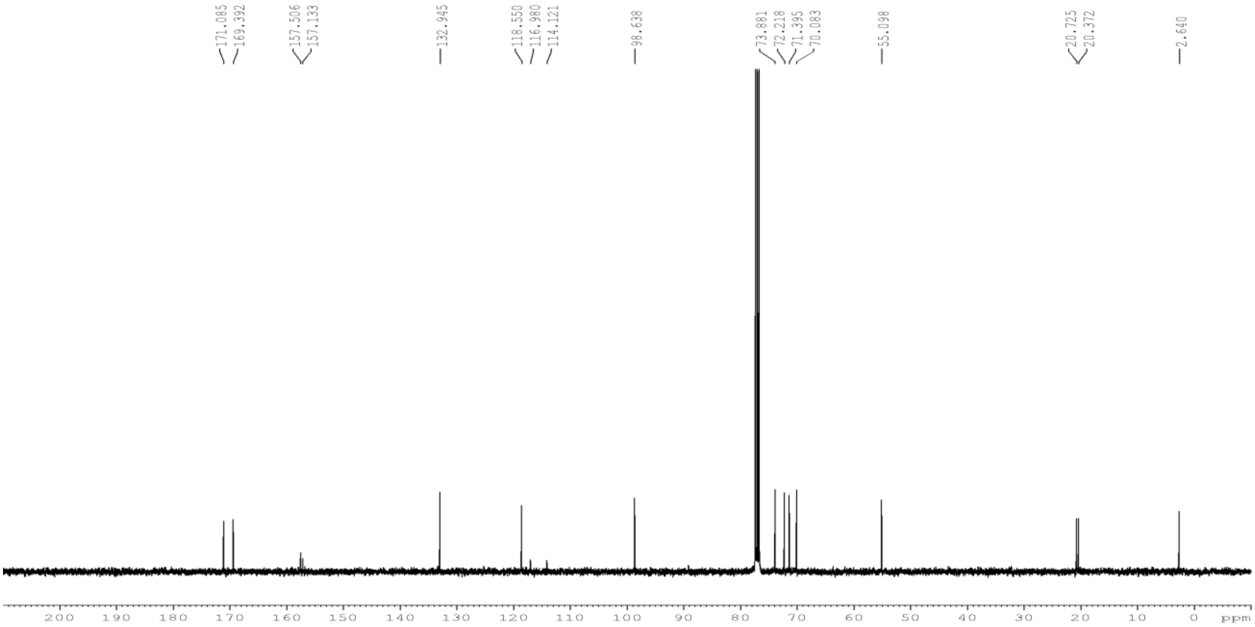

Figure. ^{13}C{^{1}H} NMR (100 MHz, $CDCl_3$) spectrum of **27**.

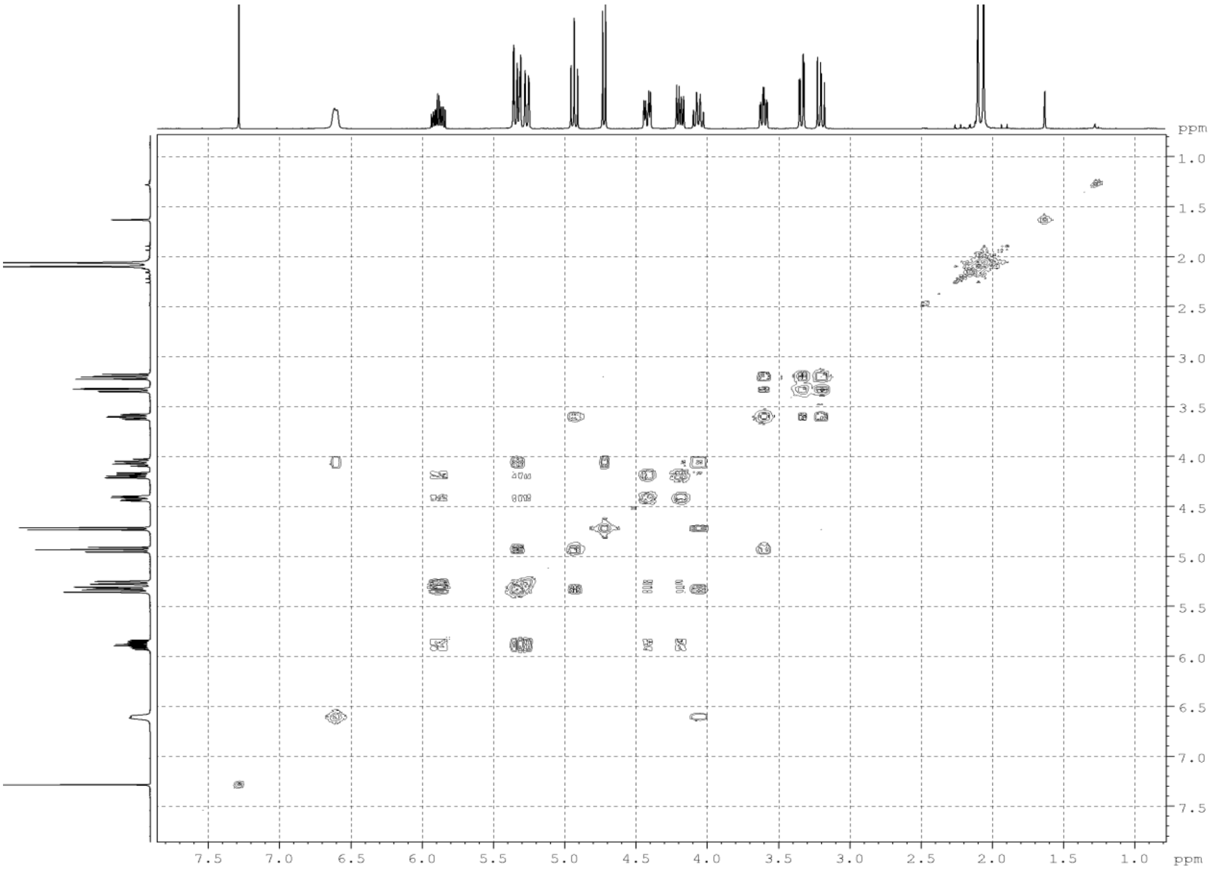

Figure. ^{1}H-^{1}H COSY NMR (400 MHz, $CDCl_3$) spectrum of **27**.

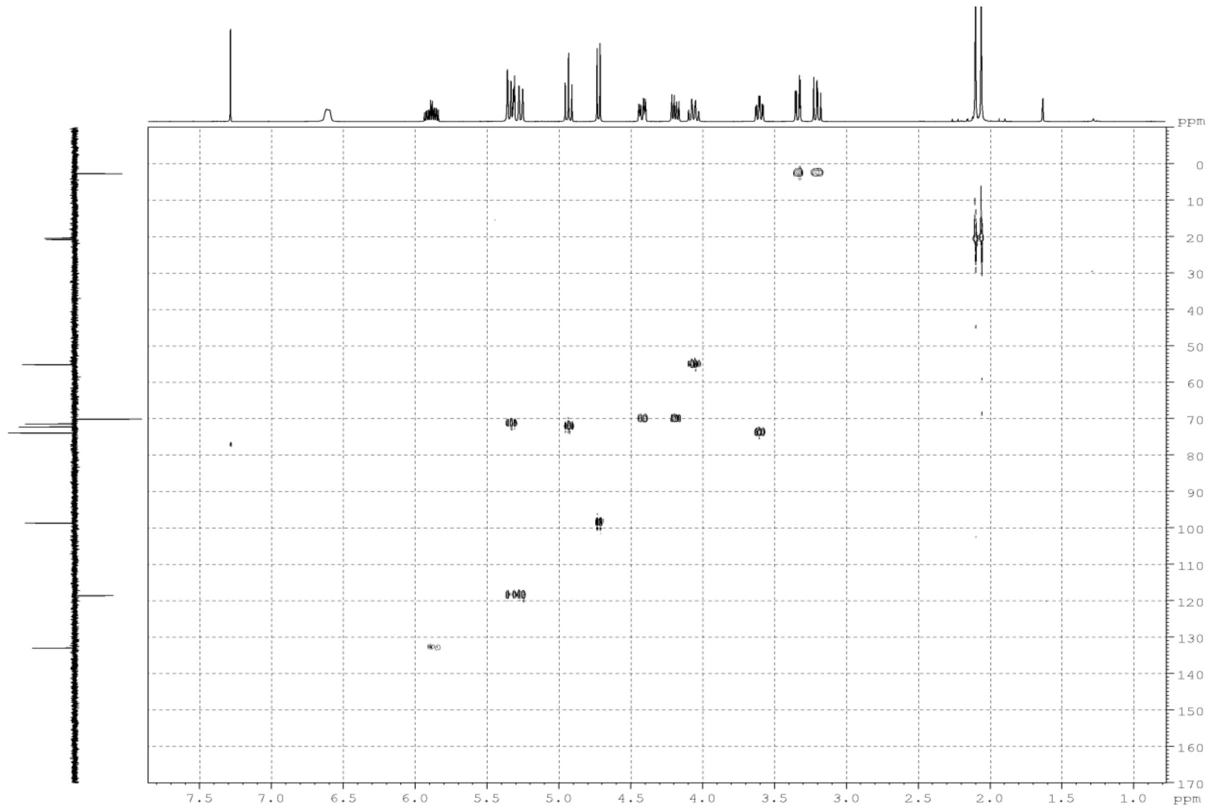

Figure. DEPT-HSQC NMR spectrum of **27**.

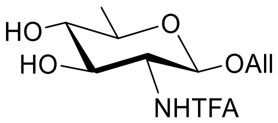

28

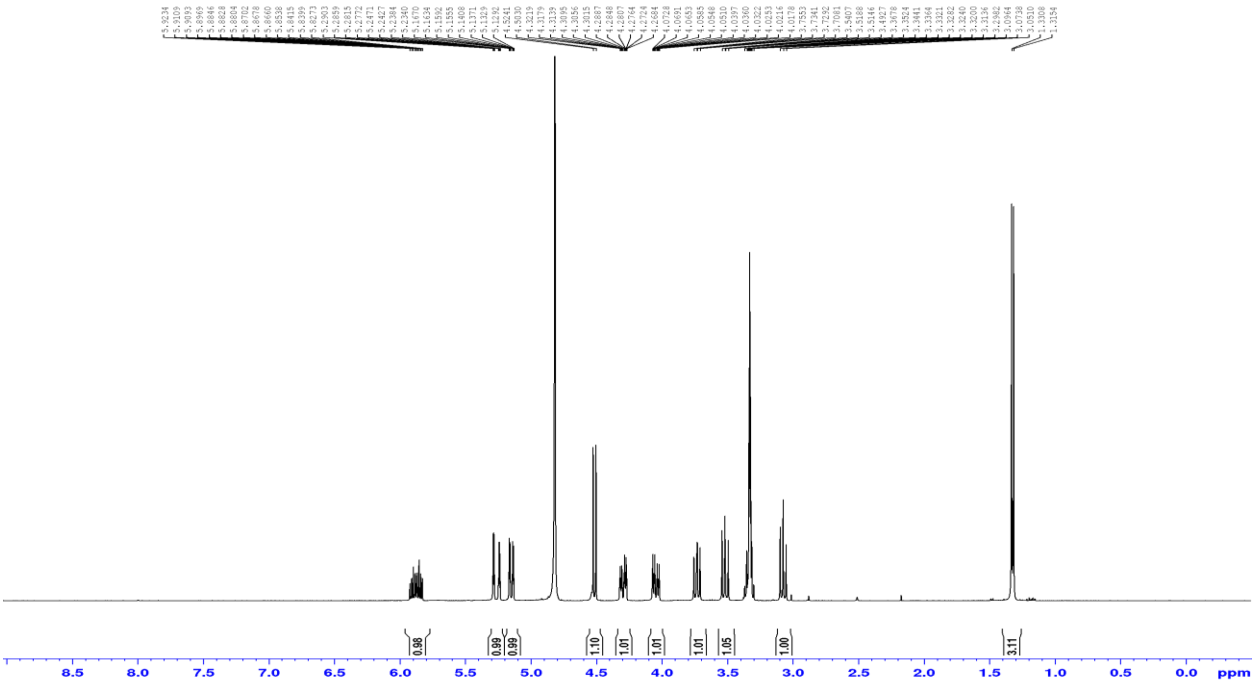

Figure. ^{1}H NMR (400 MHz, CD_3OD) spectrum of **28**.

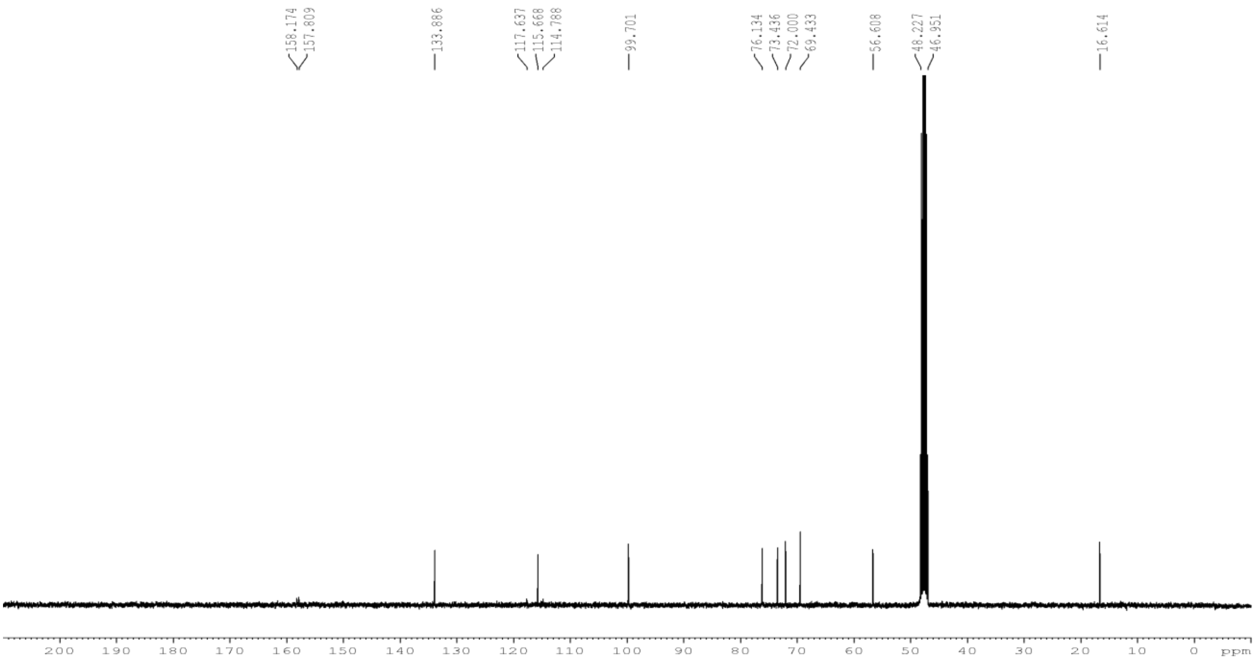

Figure. ^{13}C{^{1}H} NMR (100 MHz, CD_3OD) spectrum of **28**.

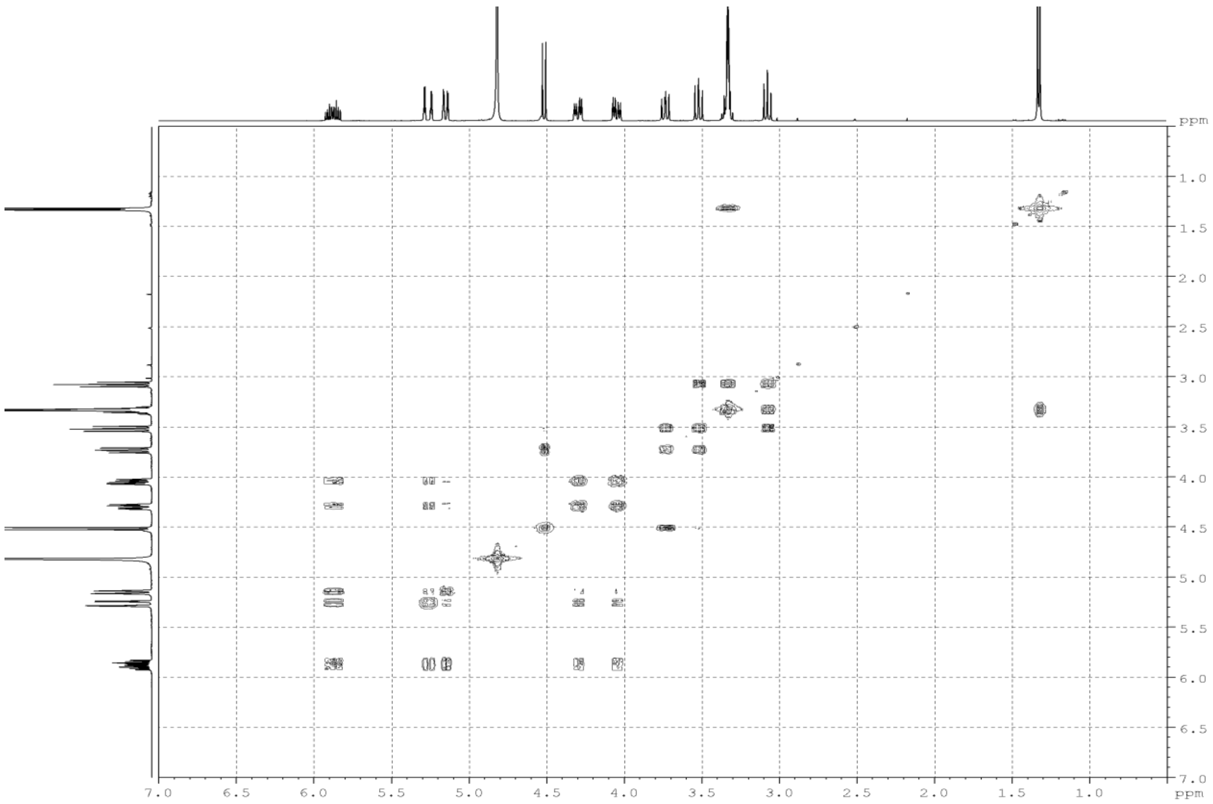

Figure. ^{1}H-^{1}H COSY NMR (400 MHz, CD_3OD) spectrum of **28**.

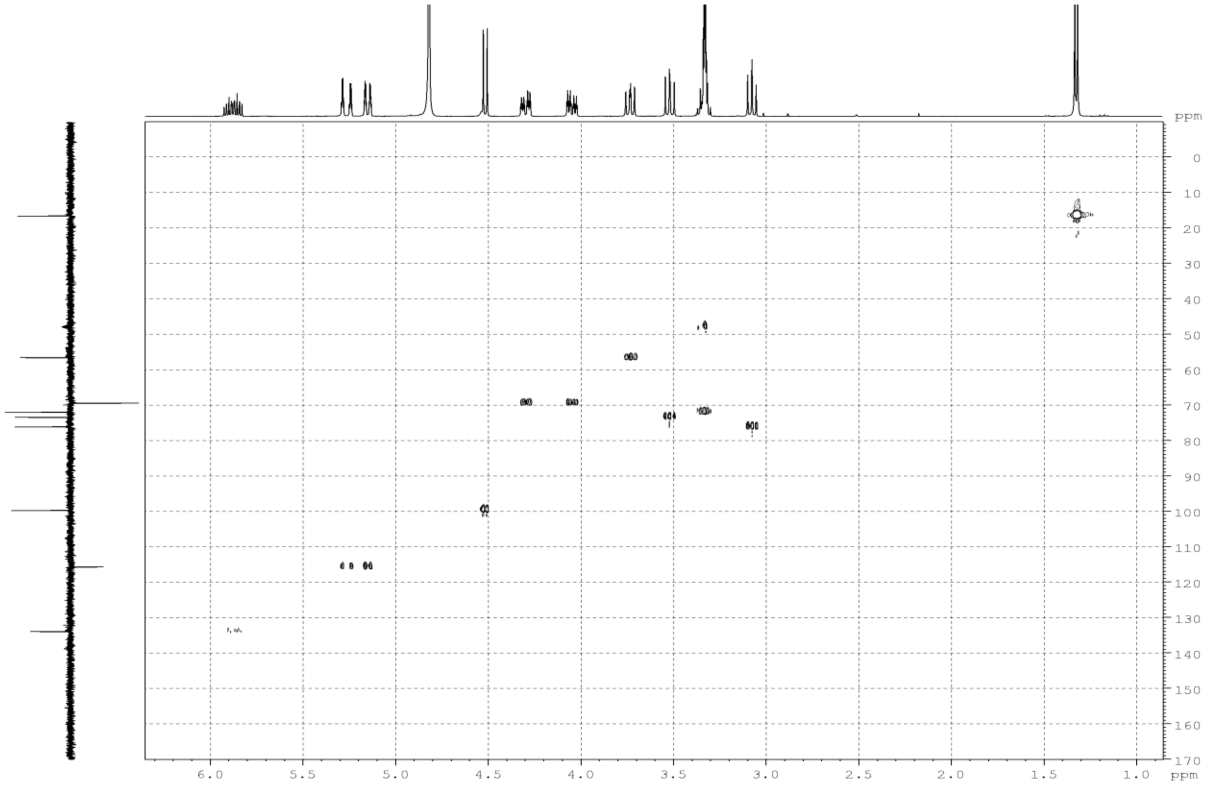

Figure. DEPT-HSQC NMR spectrum of **28**.

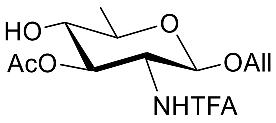

29

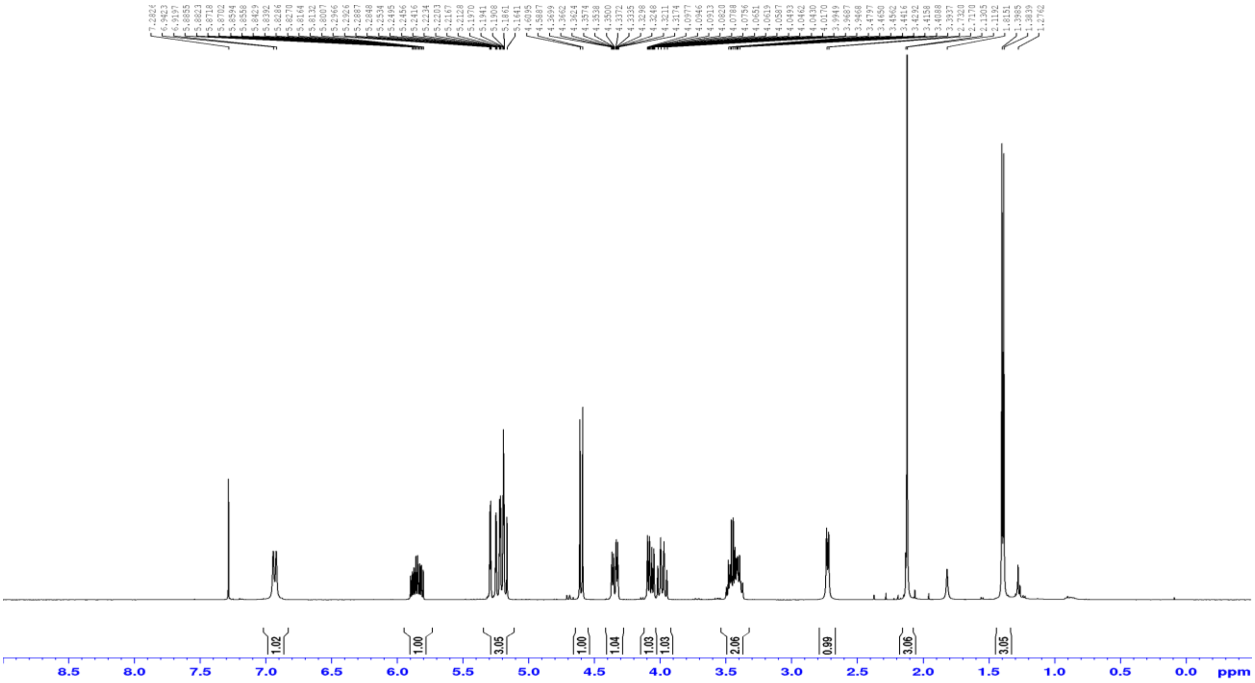

Figure. ^{1}H NMR (400 MHz, $CDCl_3$) spectrum of **29**.

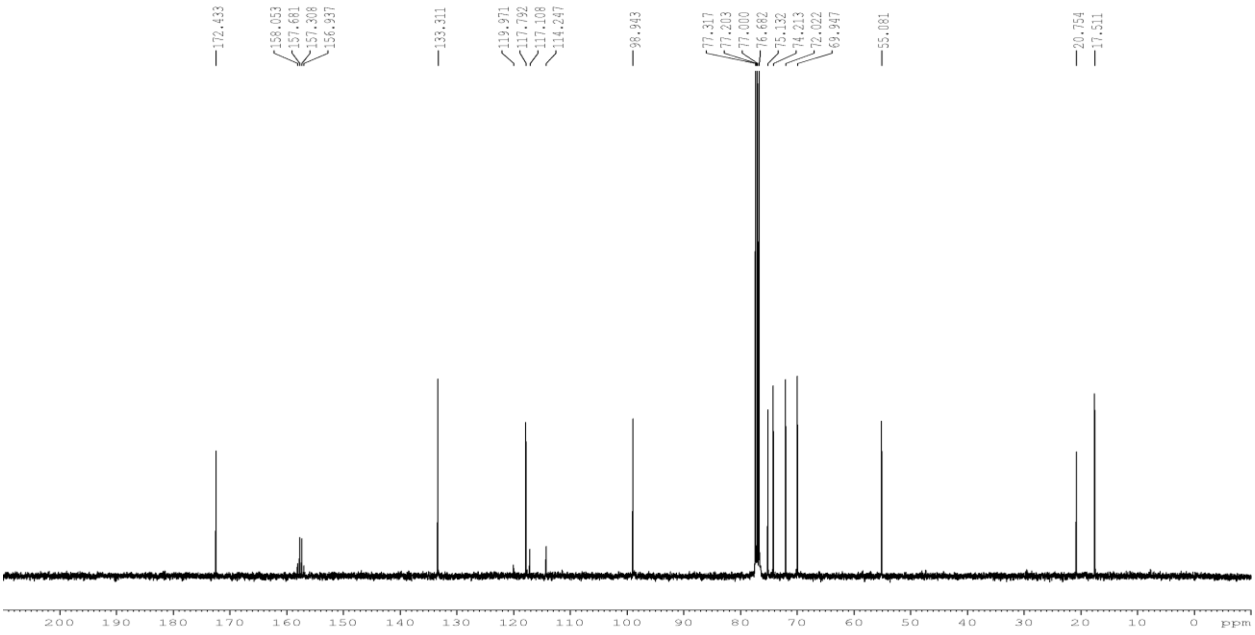

Figure. ^{13}C{^{1}H} NMR (100 MHz, $CDCl_3$) spectrum of **29**.

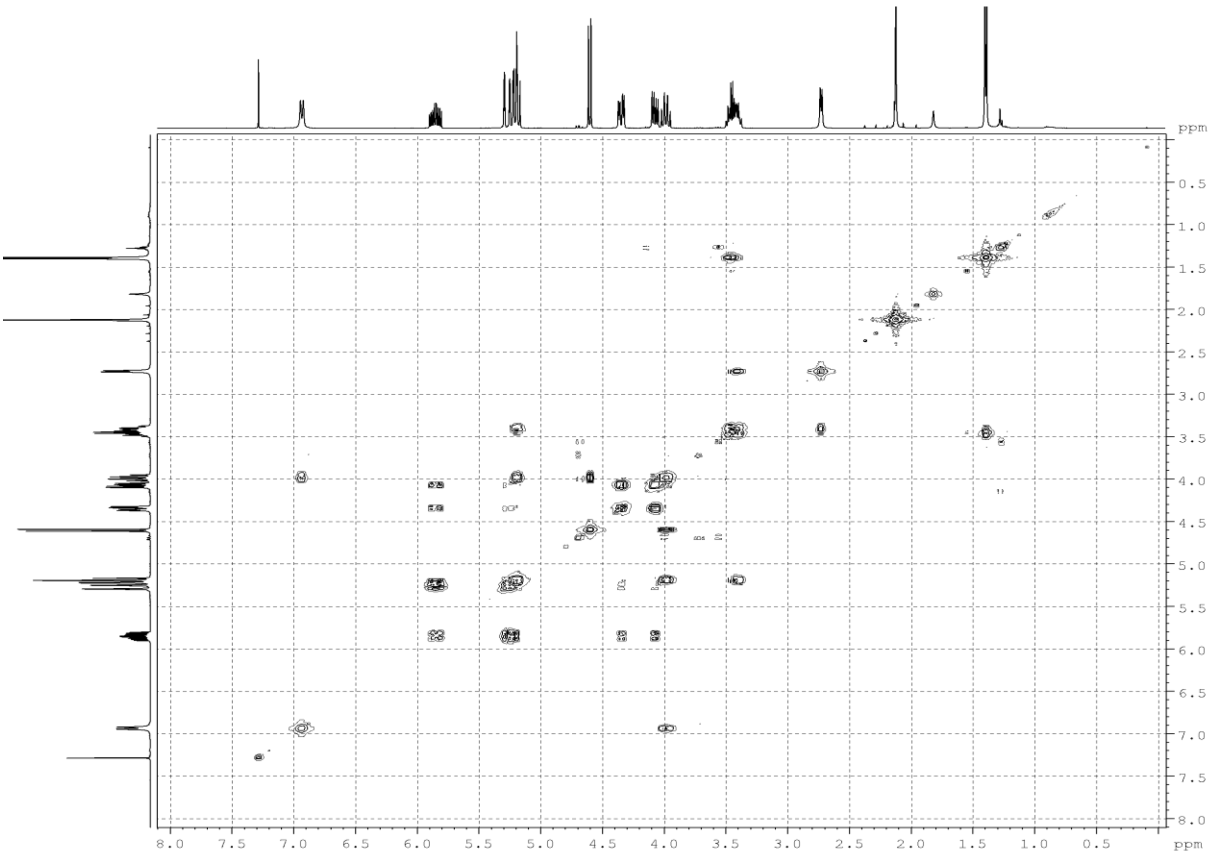

Figure. ^{1}H-^{1}H COSY NMR (400 MHz, $CDCl_3$) spectrum of **29**.

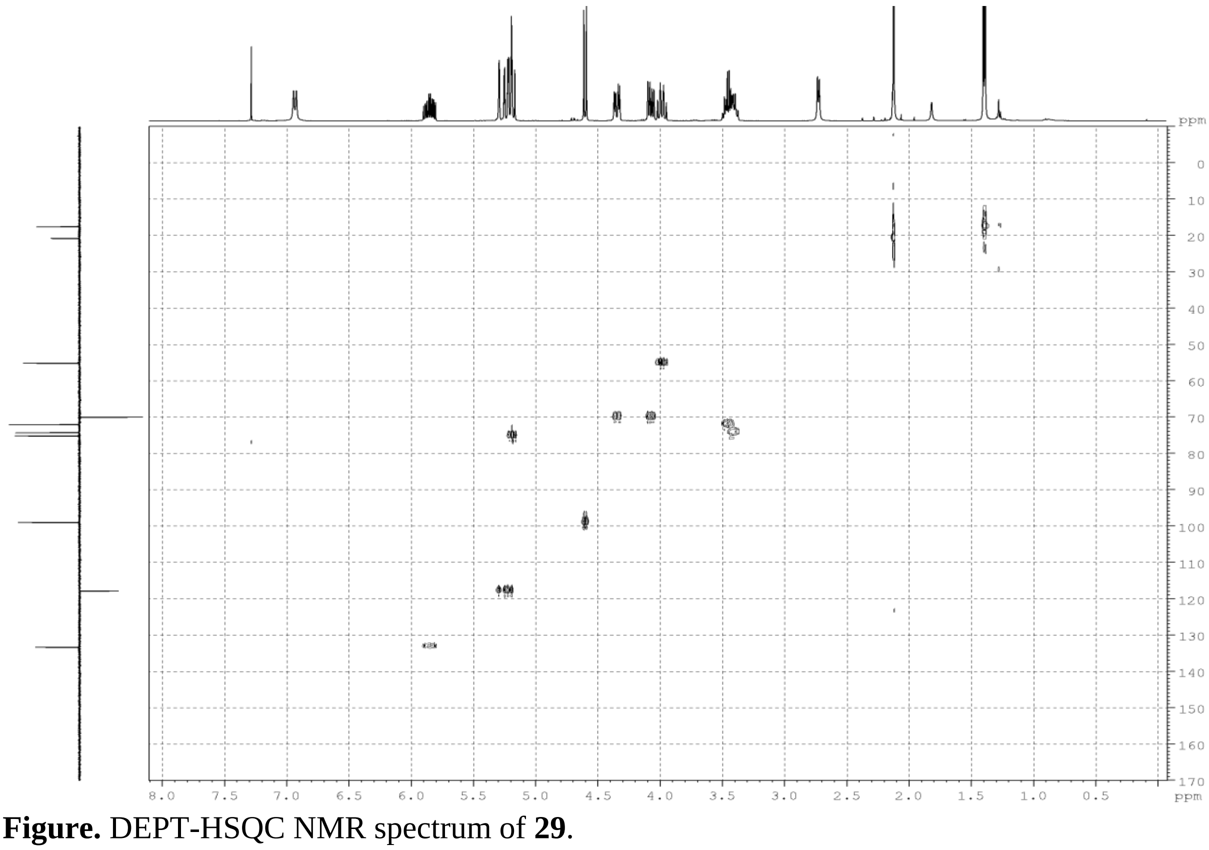

Figure. DEPT-HSQC NMR spectrum of **29**.

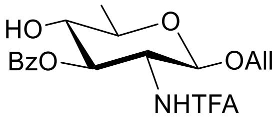

30

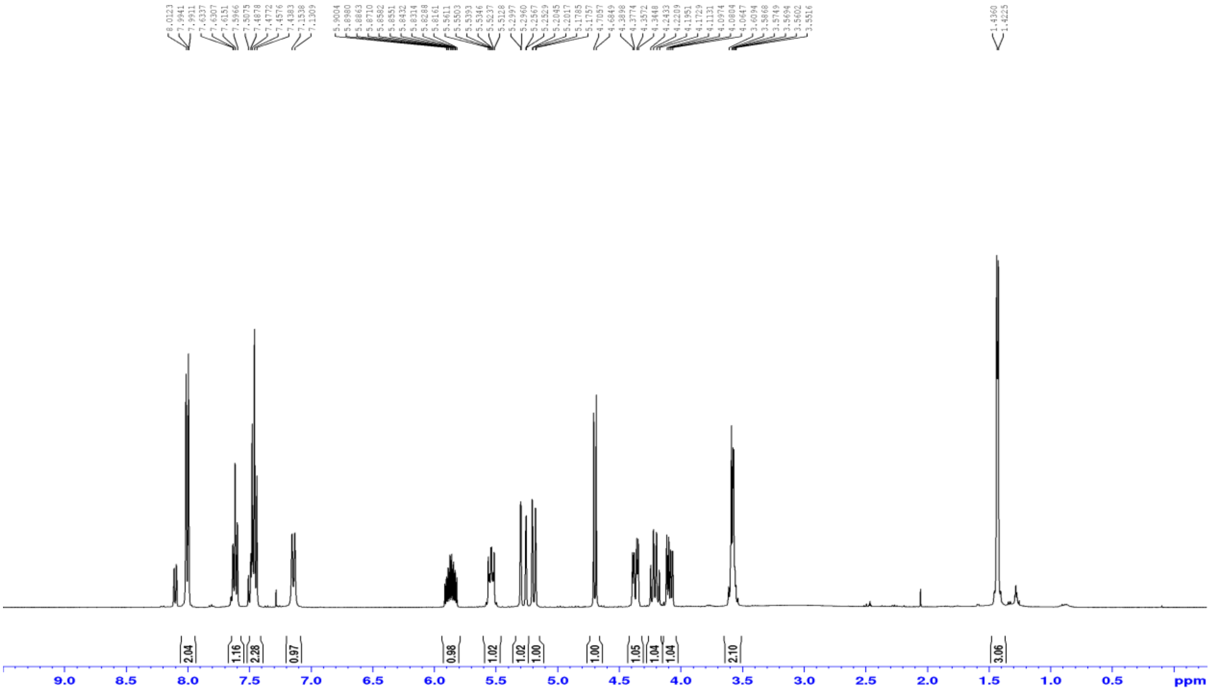

Figure. ^{1}H NMR (400 MHz, $CDCl_3$) spectrum of **30**.

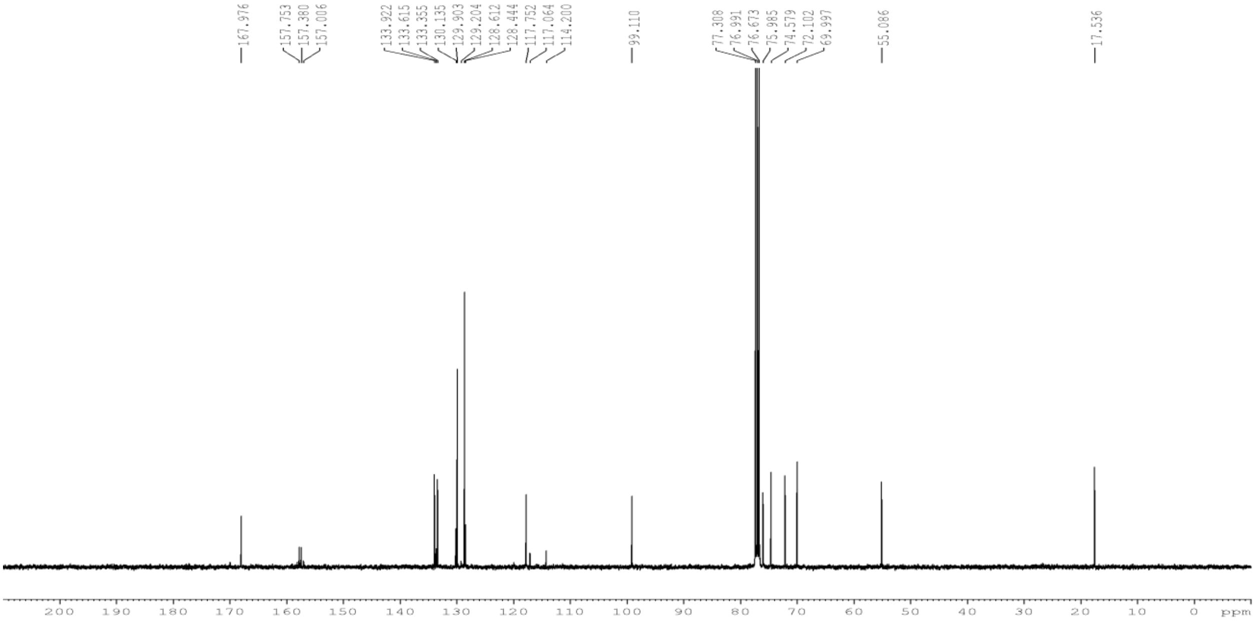

Figure. ^{13}C{^{1}H} NMR (100 MHz, $CDCl_3$) spectrum of **30**.

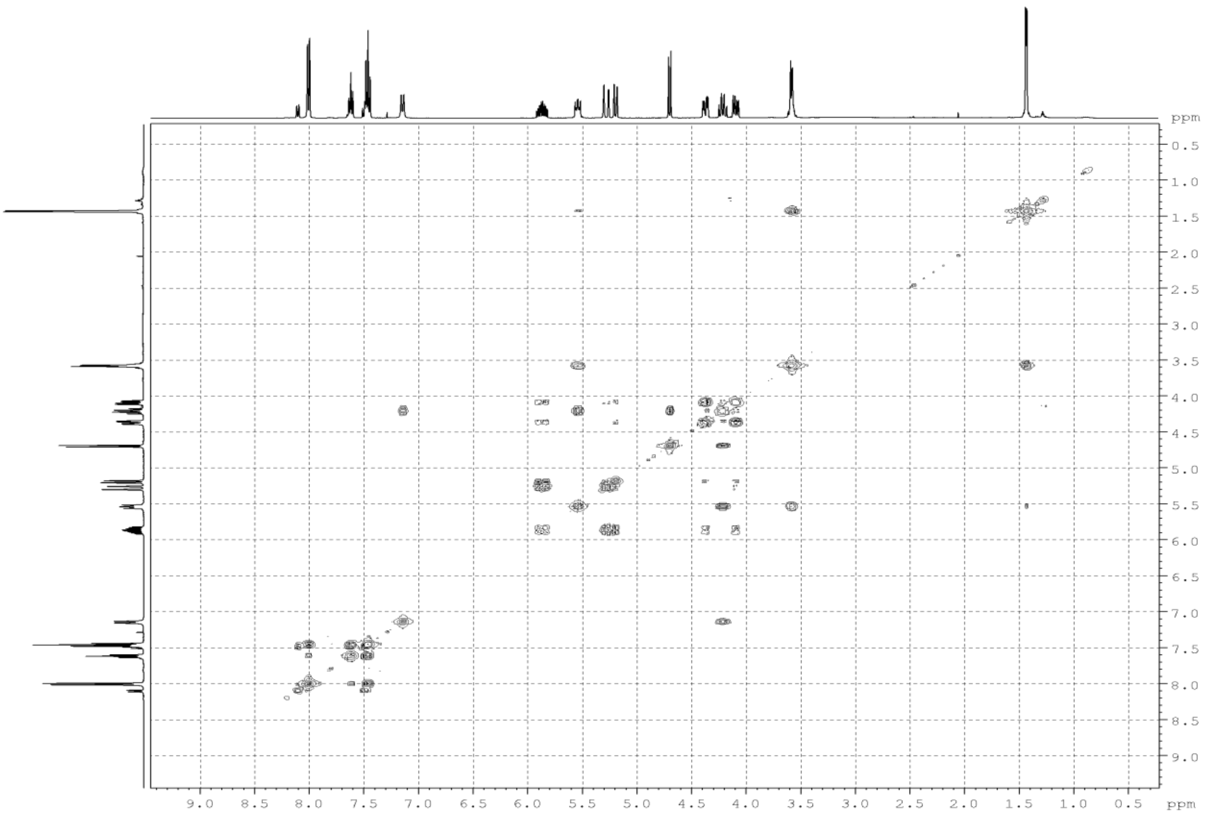

Figure. ^{1}H-^{1}H COSY NMR (400 MHz, $CDCl_3$) spectrum of **30**.

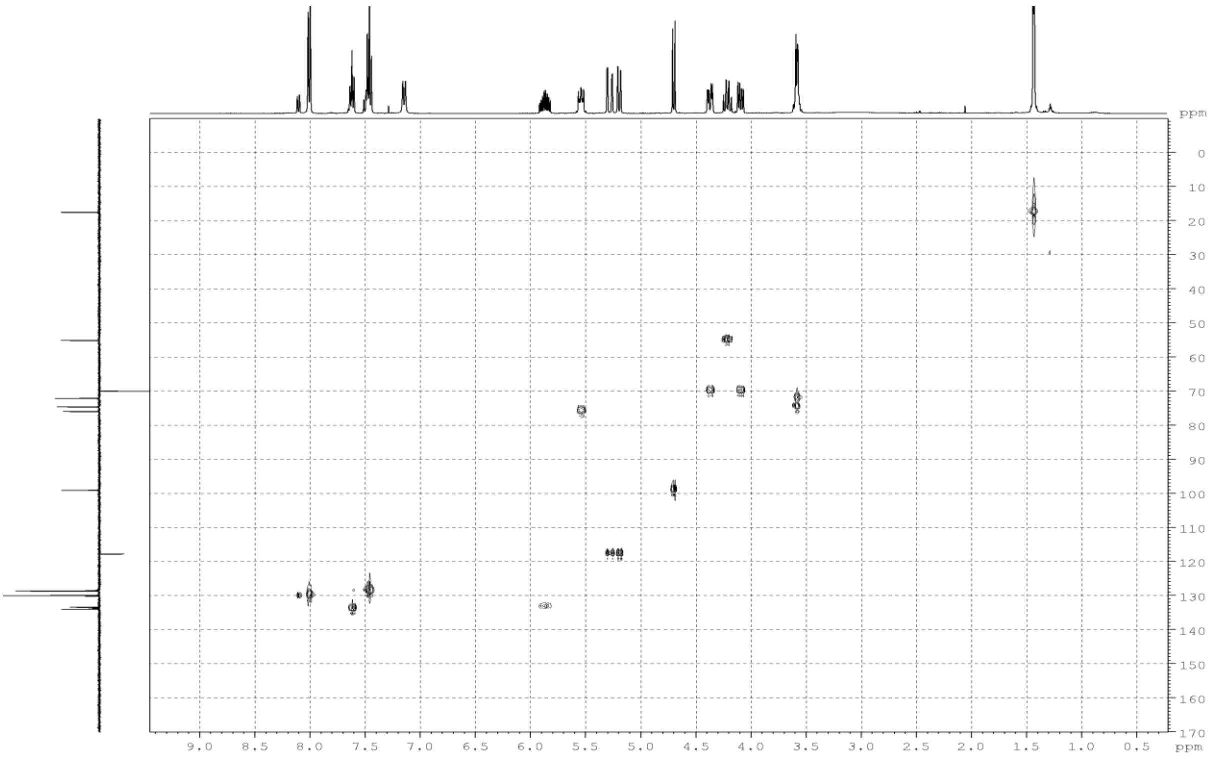

Figure. DEPT-HSQC NMR spectrum of **30**.

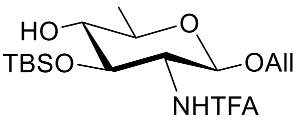

31

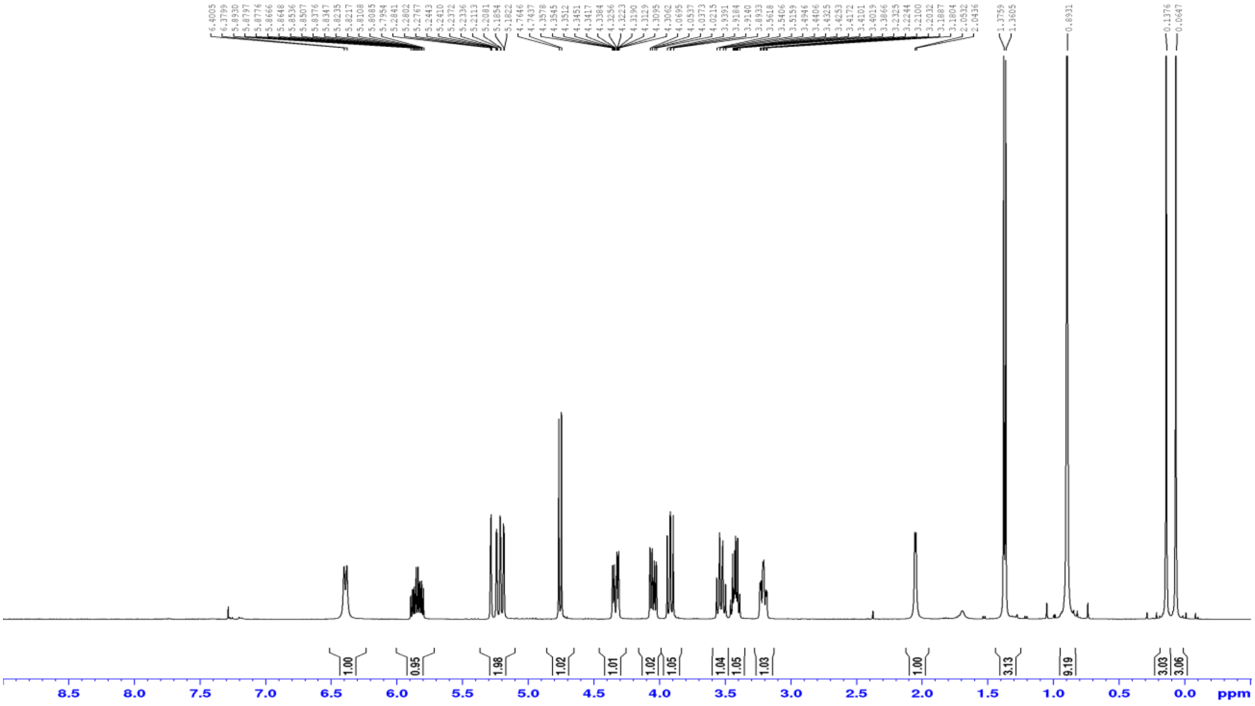

Figure. ^{1}H NMR (400 MHz, $CDCl_3$) spectrum of **31**.

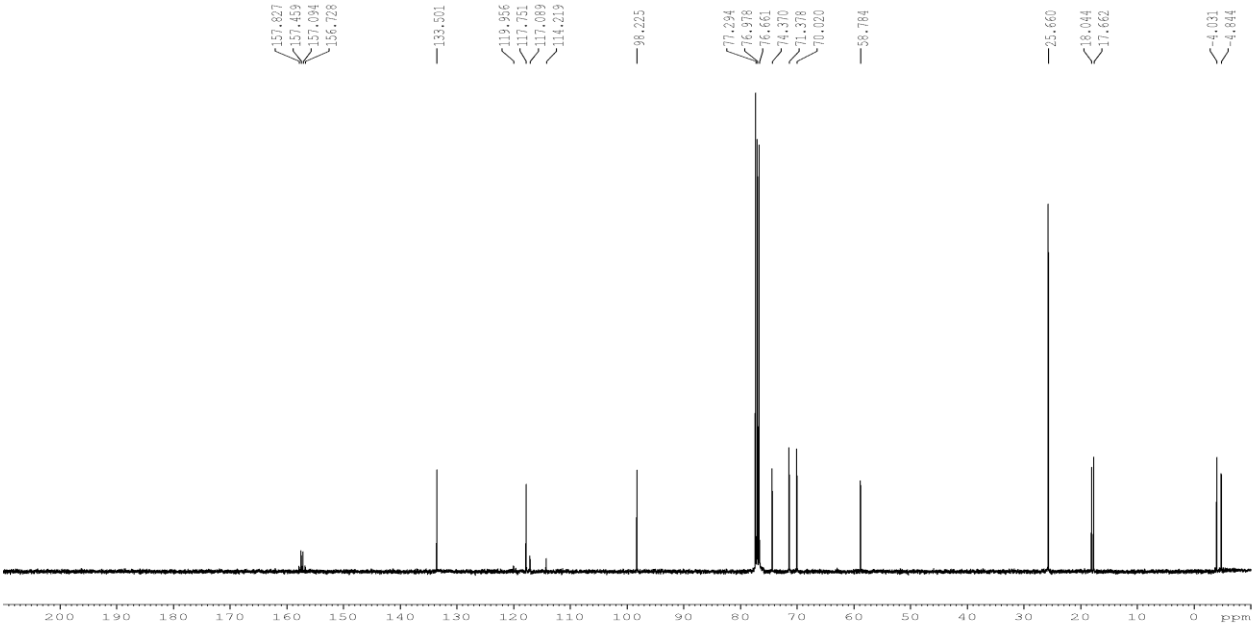

Figure. ^{13}C{^{1}H} NMR (100 MHz, $CDCl_3$) spectrum of **31**.

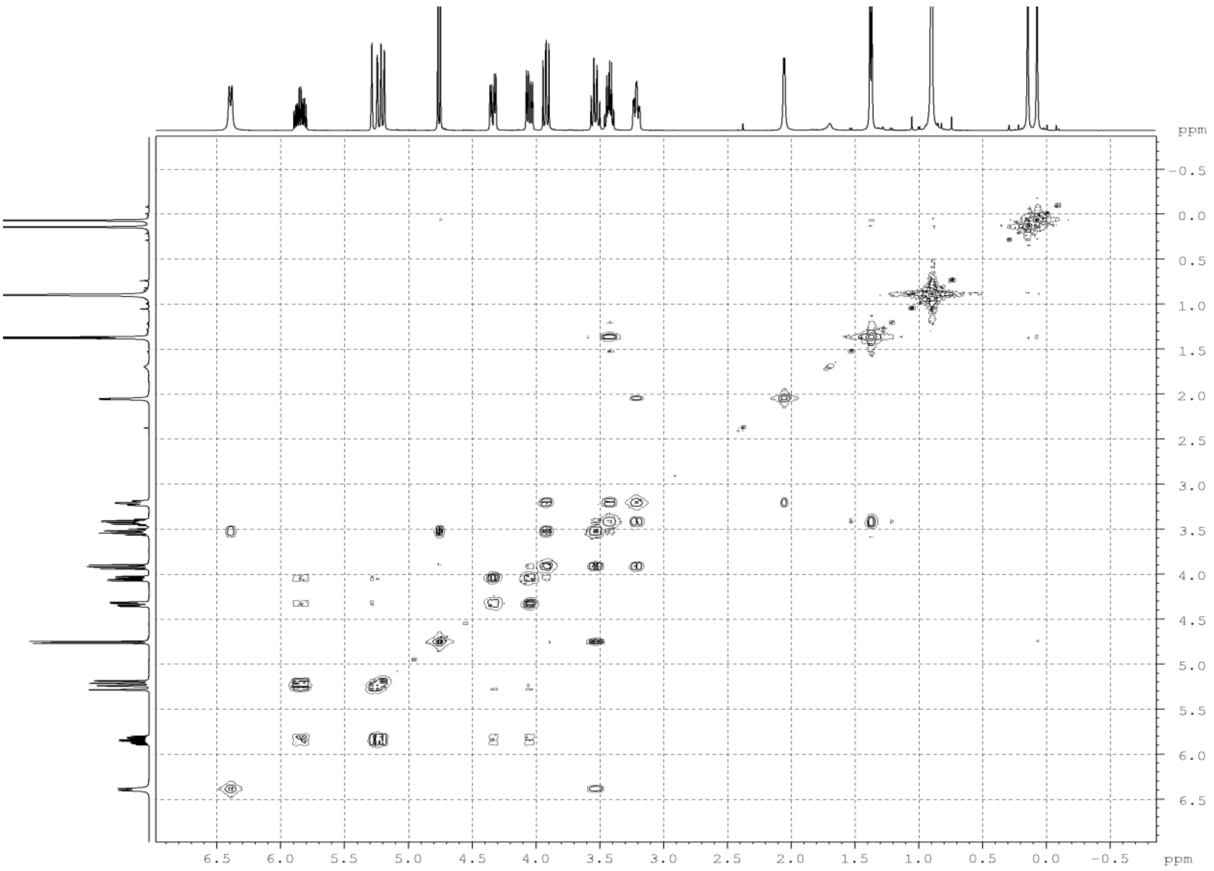

Figure. ^{1}H-^{1}H COSY NMR (400 MHz, $CDCl_3$) spectrum of **31**.

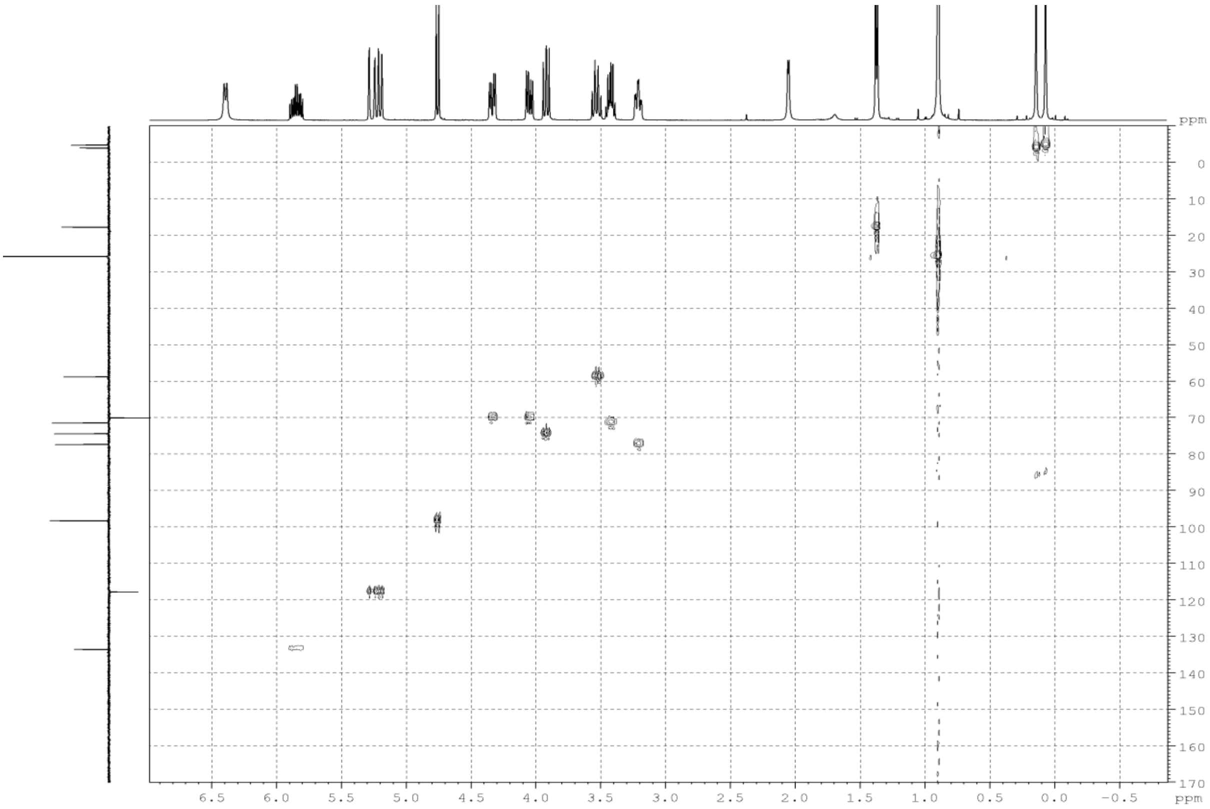

Figure. DEPT-HSQC NMR spectrum of **31**.

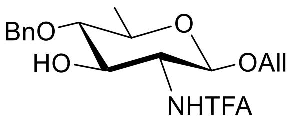

32

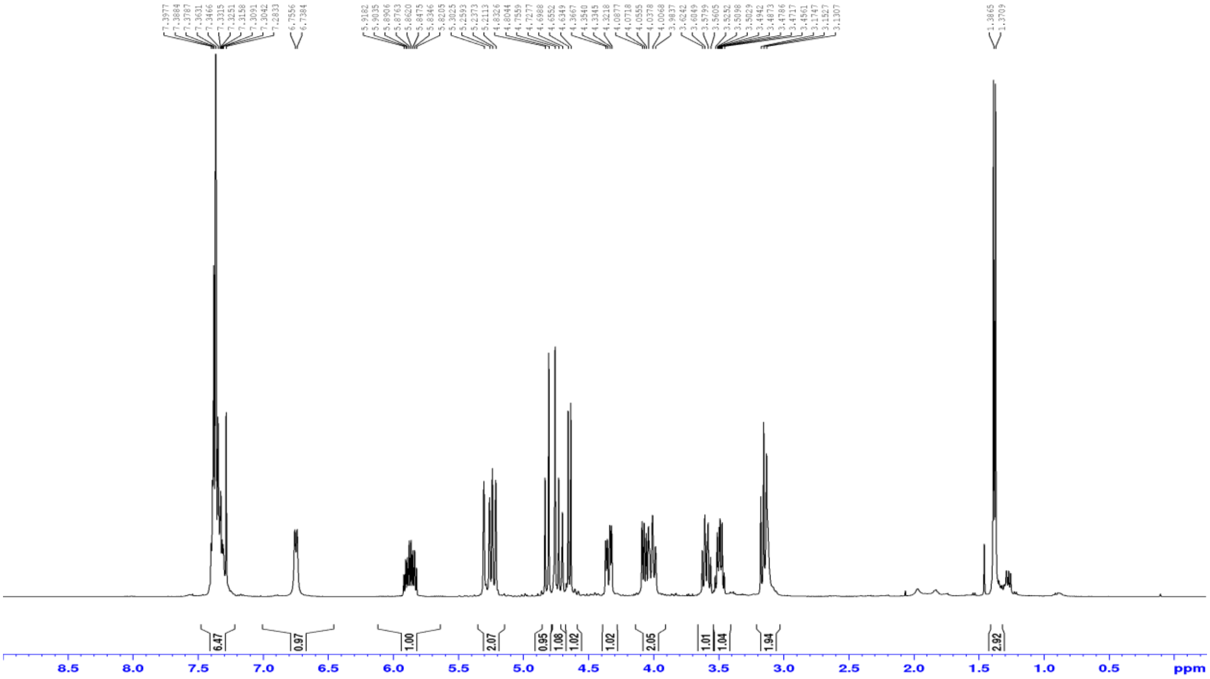

Figure. ^{1}H NMR (400 MHz, $CDCl_3$) spectrum of **32**.

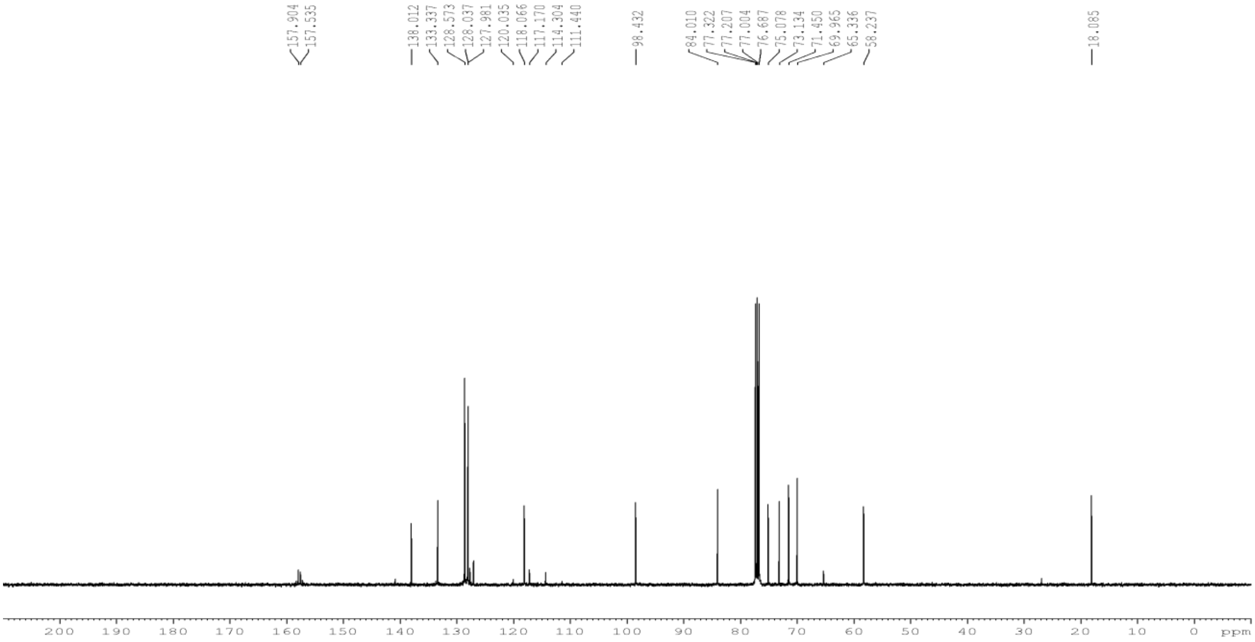

Figure. ^{13}C{^{1}H} NMR (100 MHz, $CDCl_3$) spectrum of **32**.

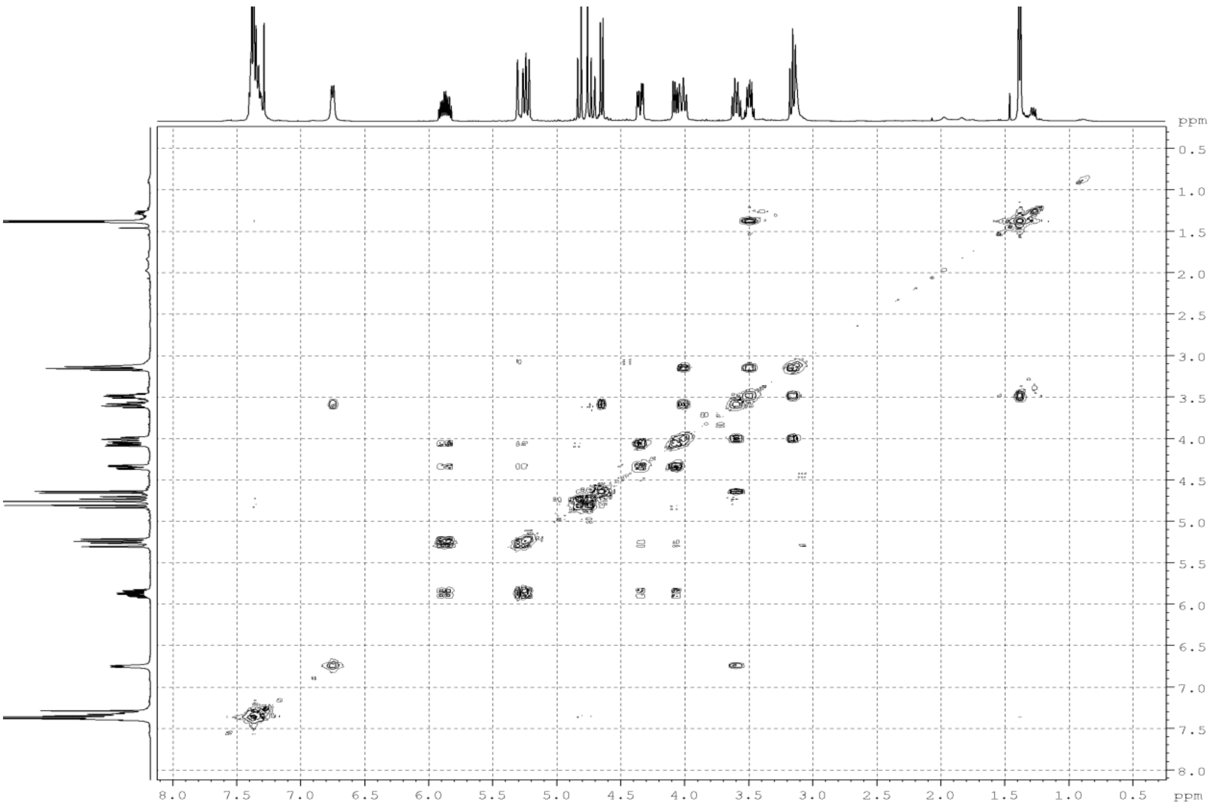

Figure. ^{1}H-^{1}H COSY NMR (400 MHz, $CDCl_3$) spectrum of **32**.

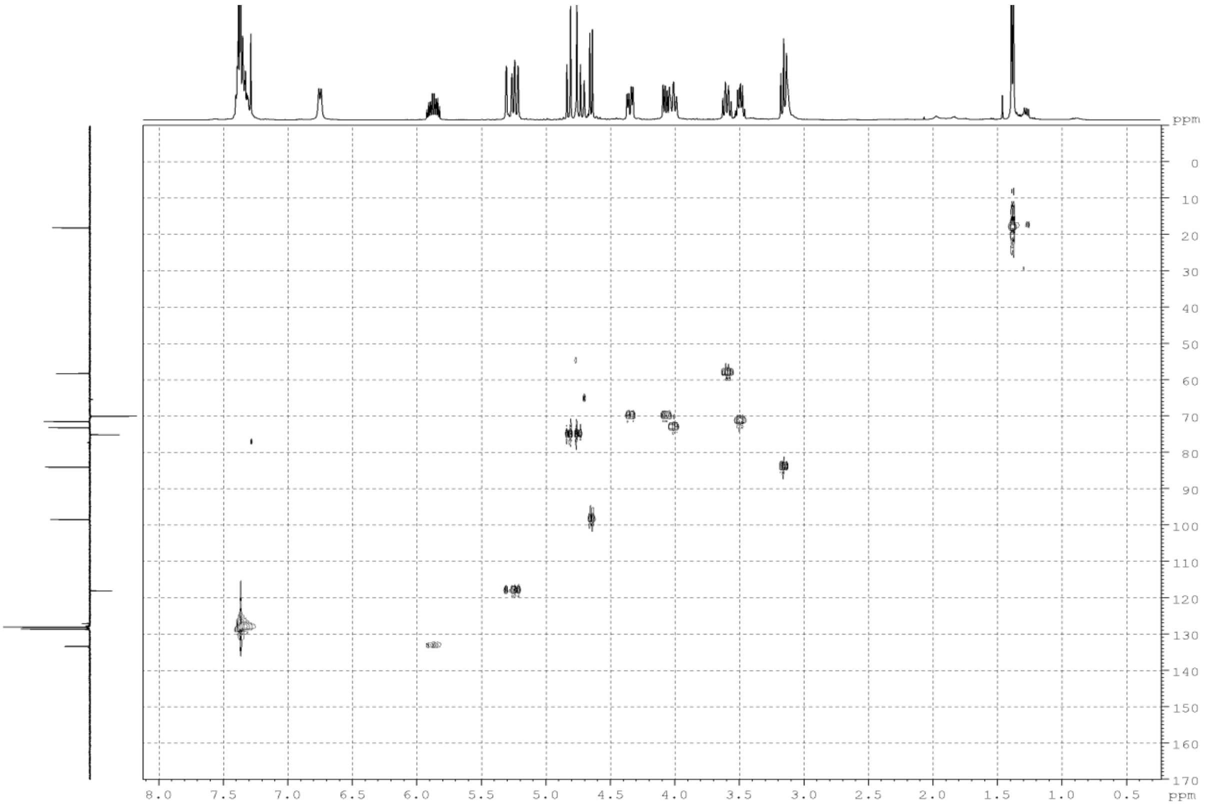

Figure. DEPT-HSQC NMR spectrum of **32**.

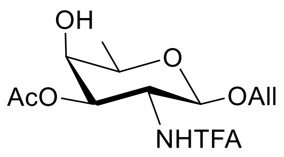

33

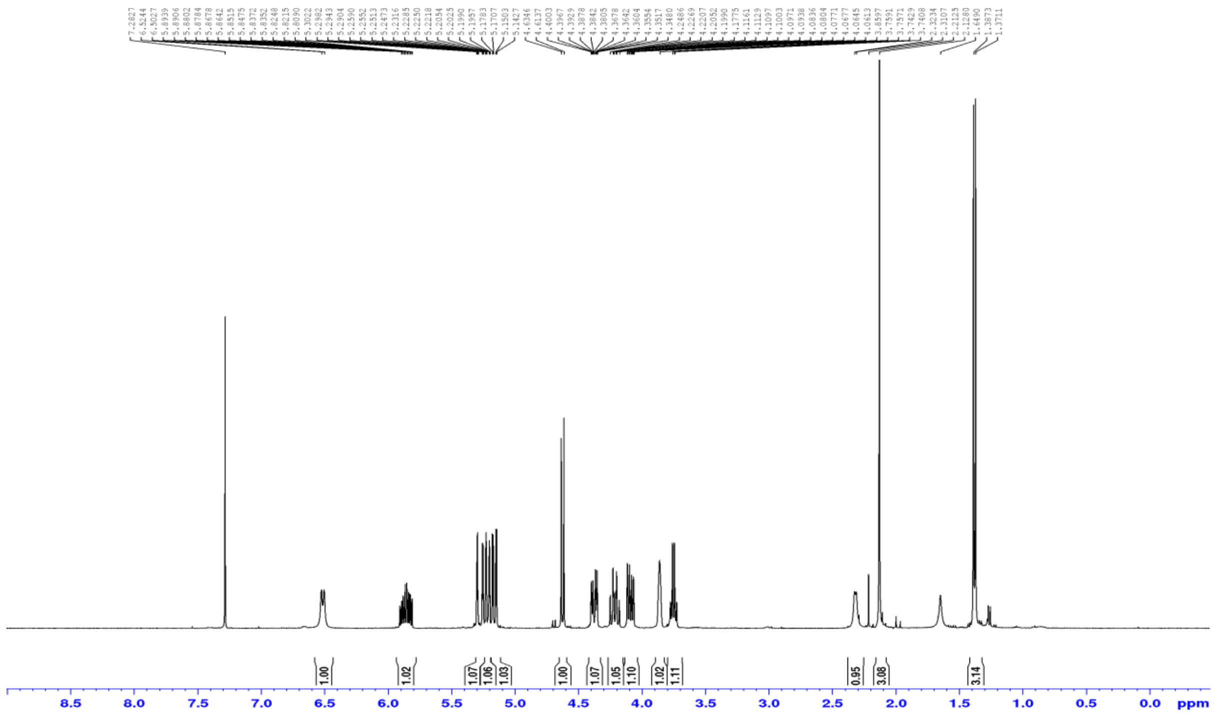

Figure. ^{1}H NMR (400 MHz, $CDCl_3$) spectrum of **33**.

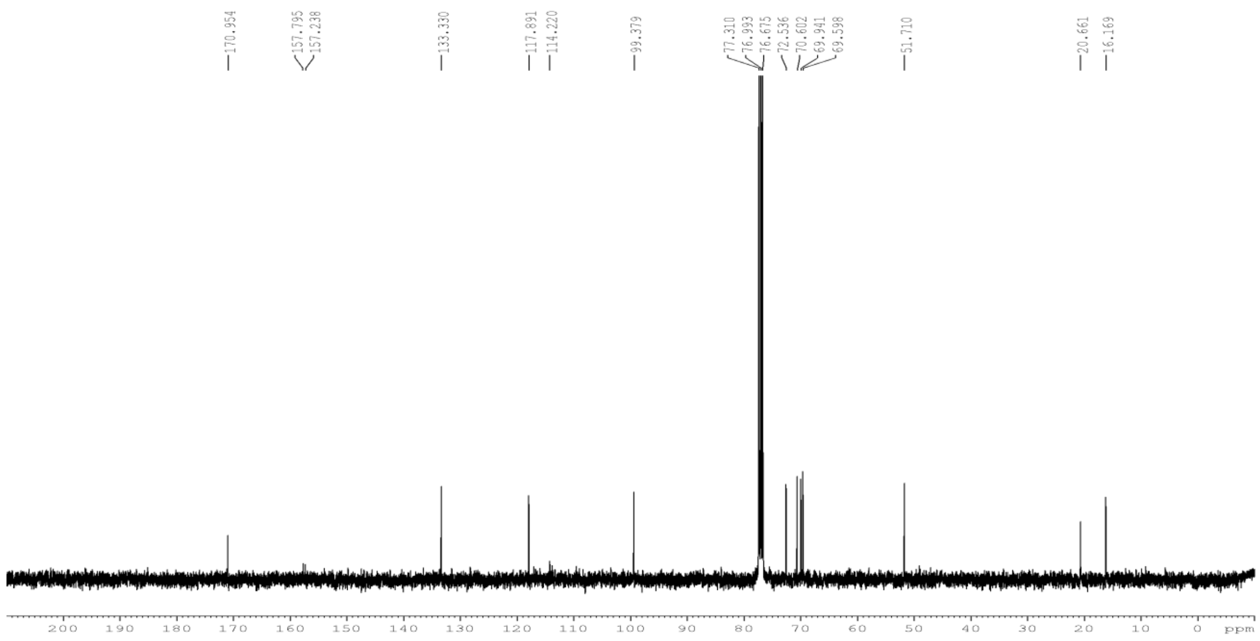

Figure. ^{13}C{^{1}H} NMR (100 MHz, $CDCl_3$) spectrum of **33**.

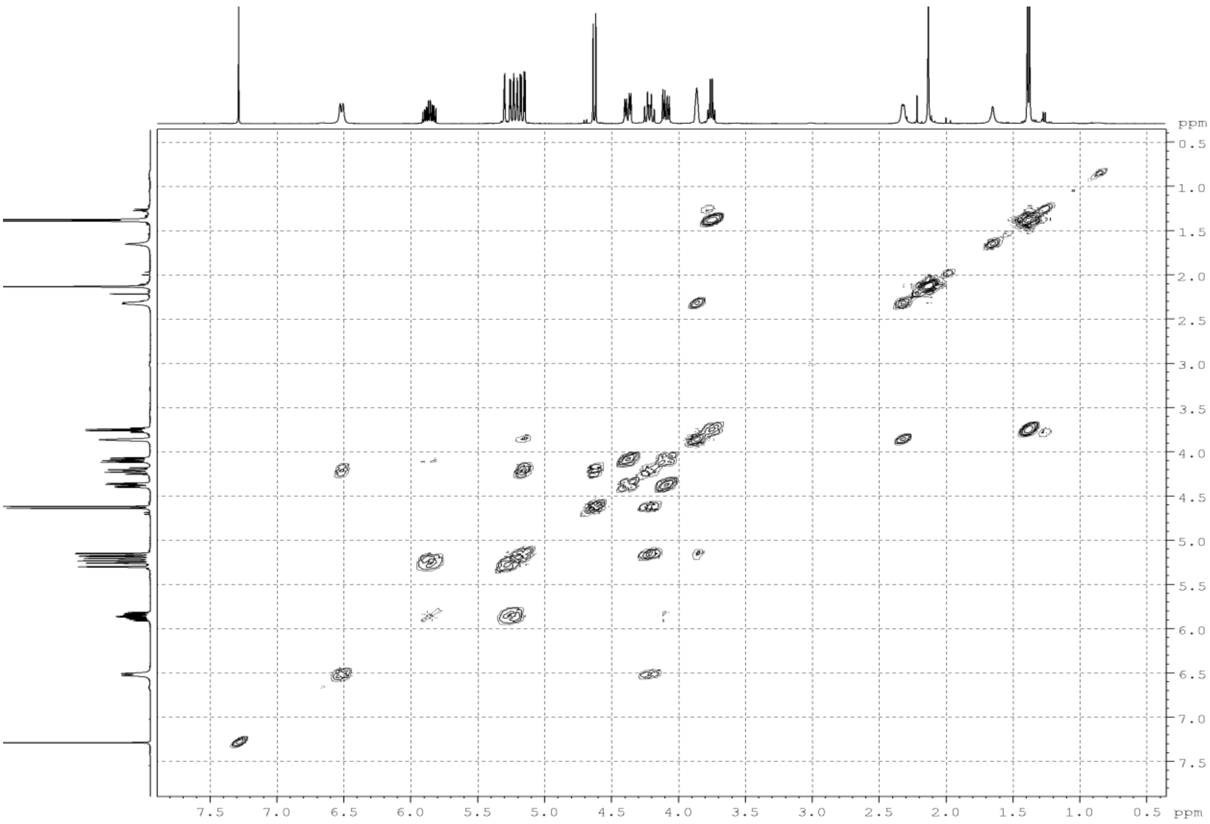

Figure. ^1H-^1H COSY NMR (400 MHz, $CDCl_3$) spectrum of **33**.

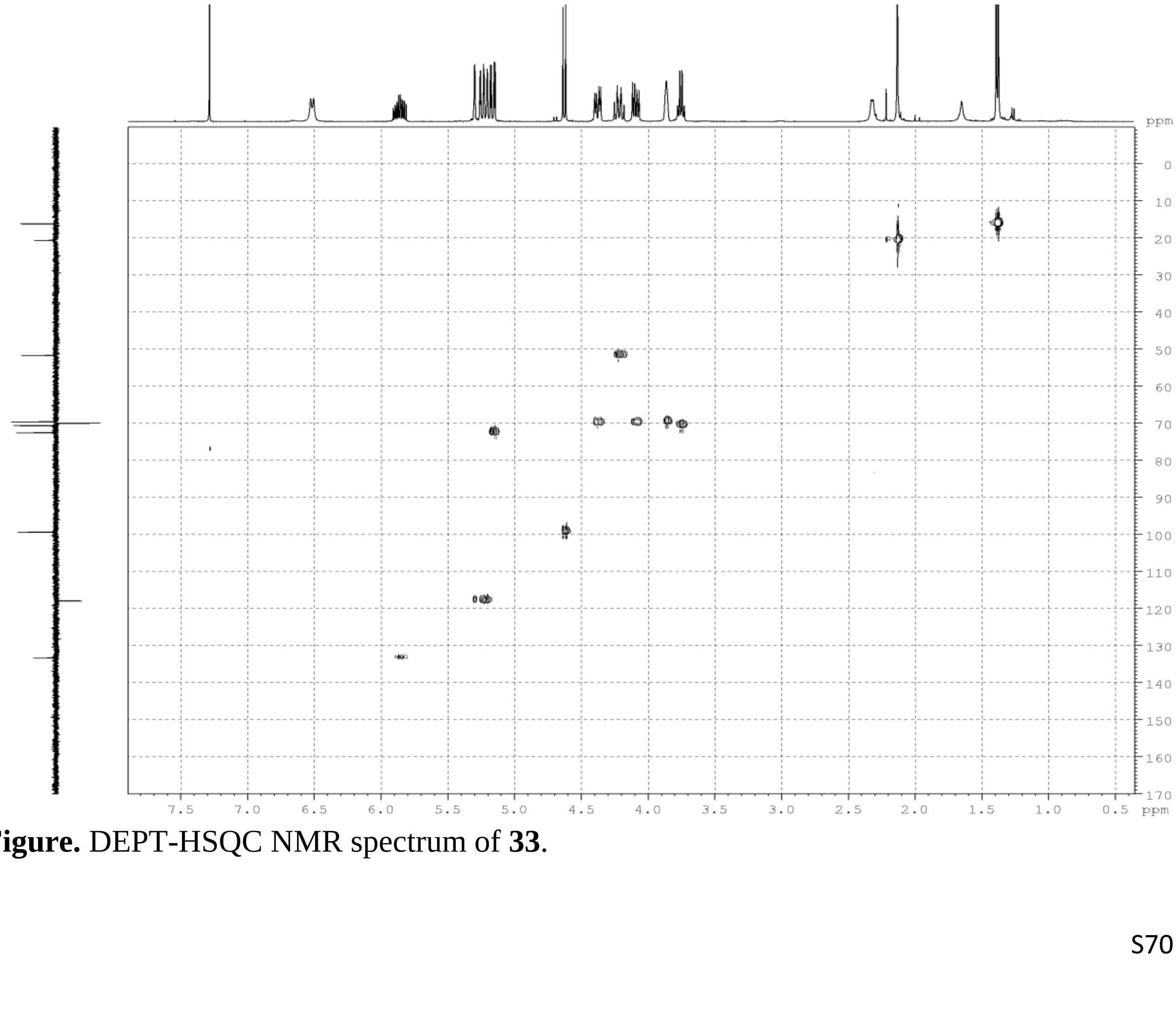

Figure. DEPT-HSQC NMR spectrum of **33**.

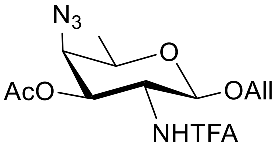

34

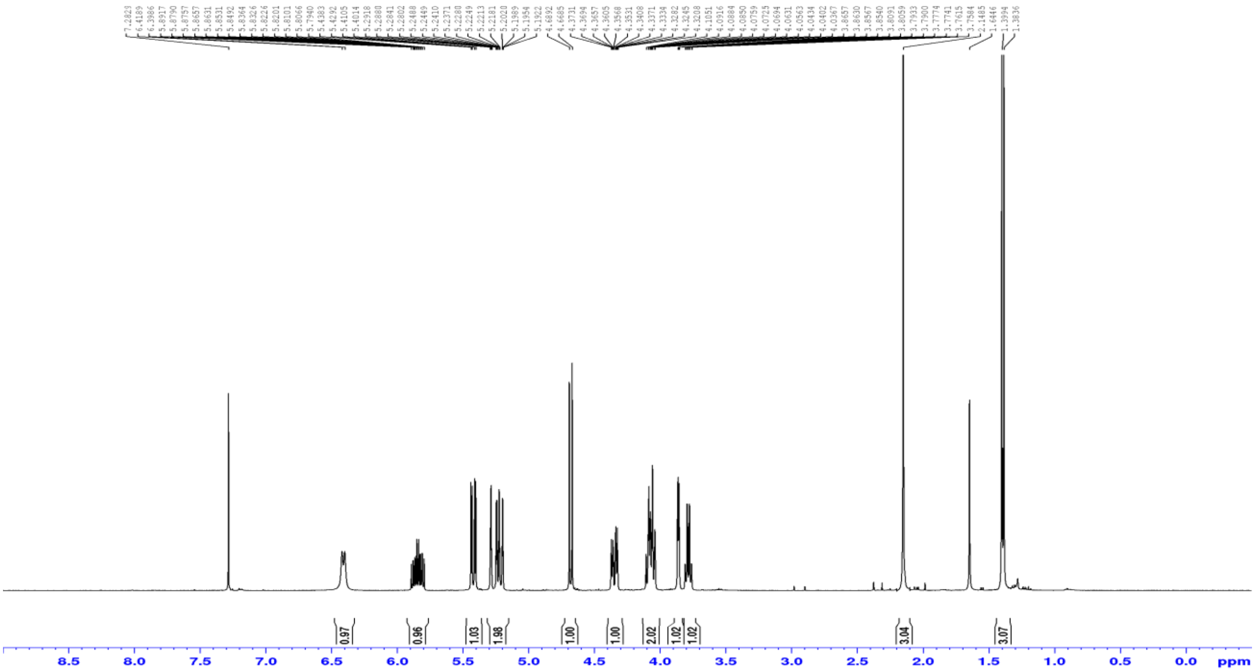

Figure. ^{1}H NMR (400 MHz, $CDCl_3$) spectrum of **34**.

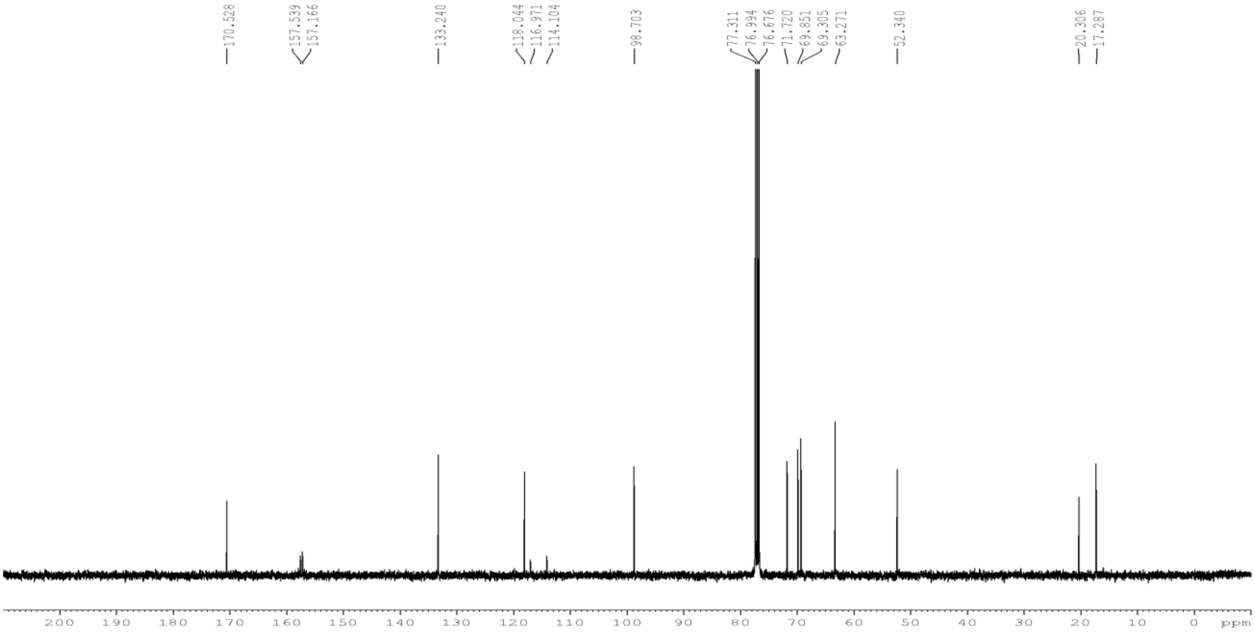

Figure. $^{13}C\{^{1}H\}$ NMR (100 MHz, $CDCl_3$) spectrum of **34**.

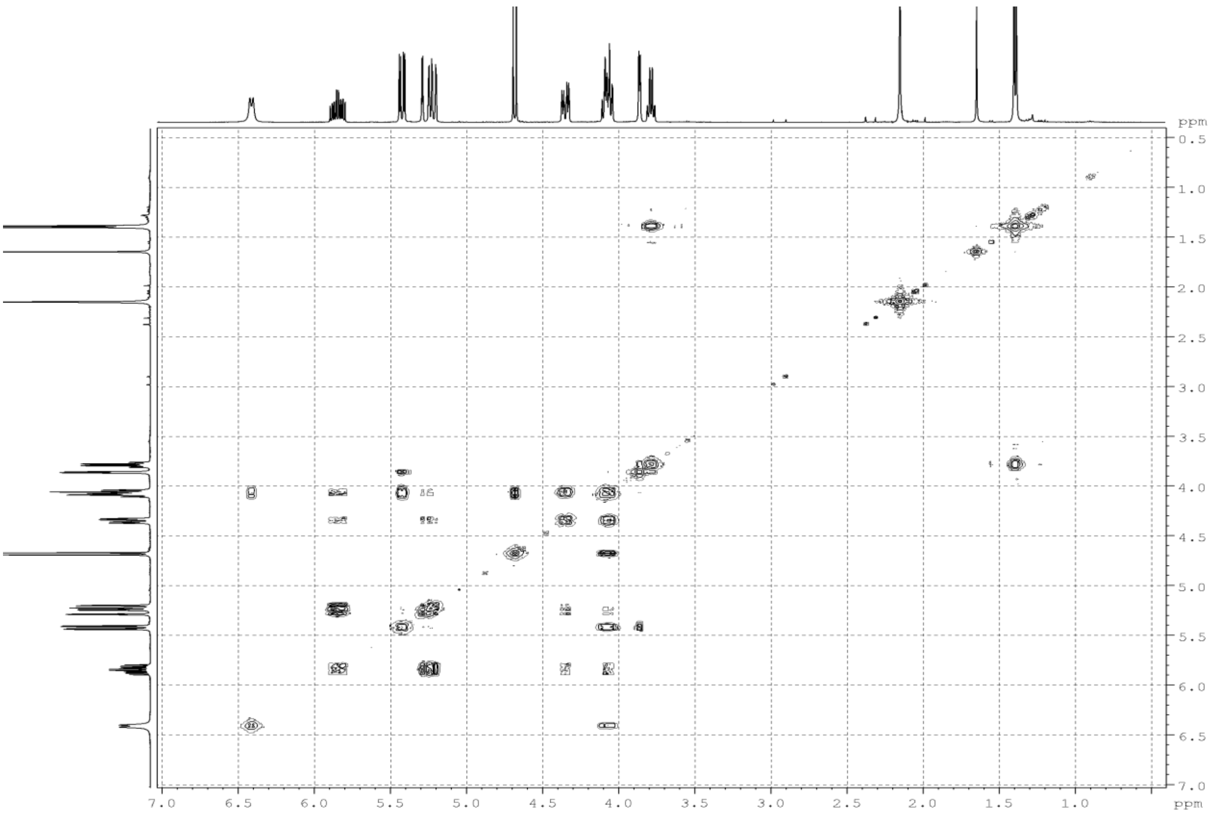

Figure. ^{1}H-^{1}H COSY NMR (400 MHz, $CDCl_3$) spectrum of **34**.

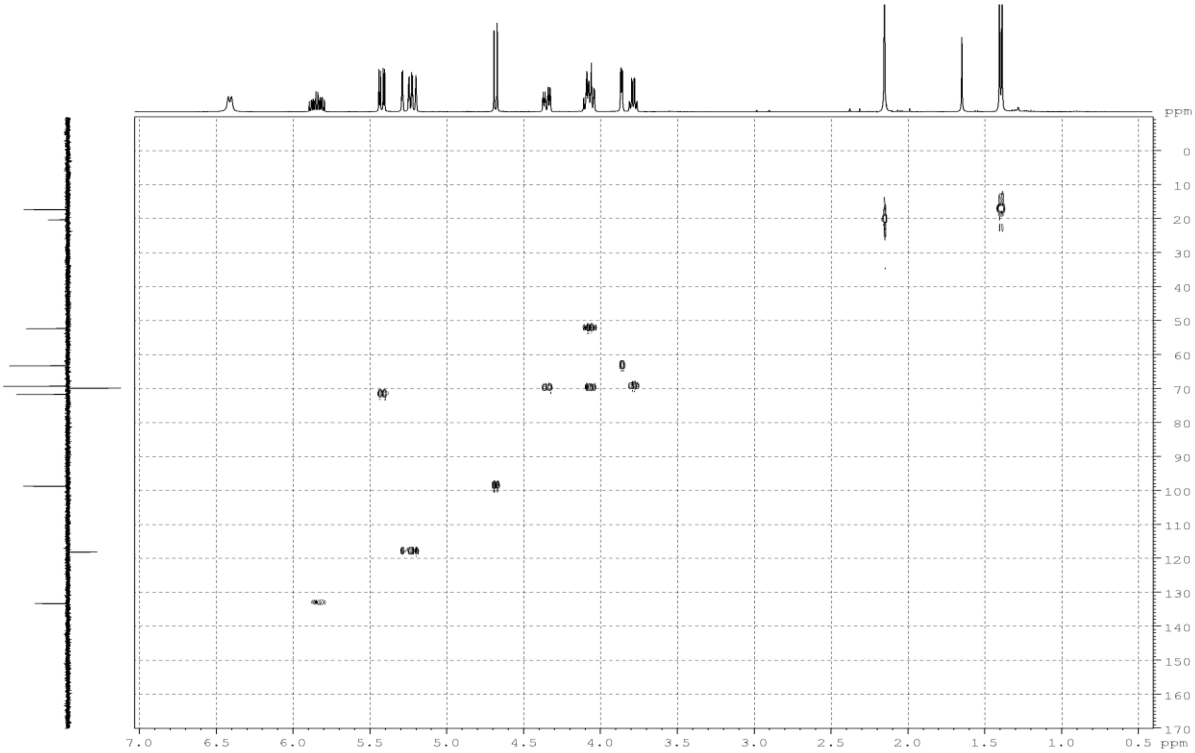

Figure. DEPT-HSQC NMR spectrum of **34**.

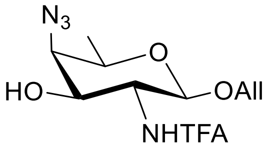

35

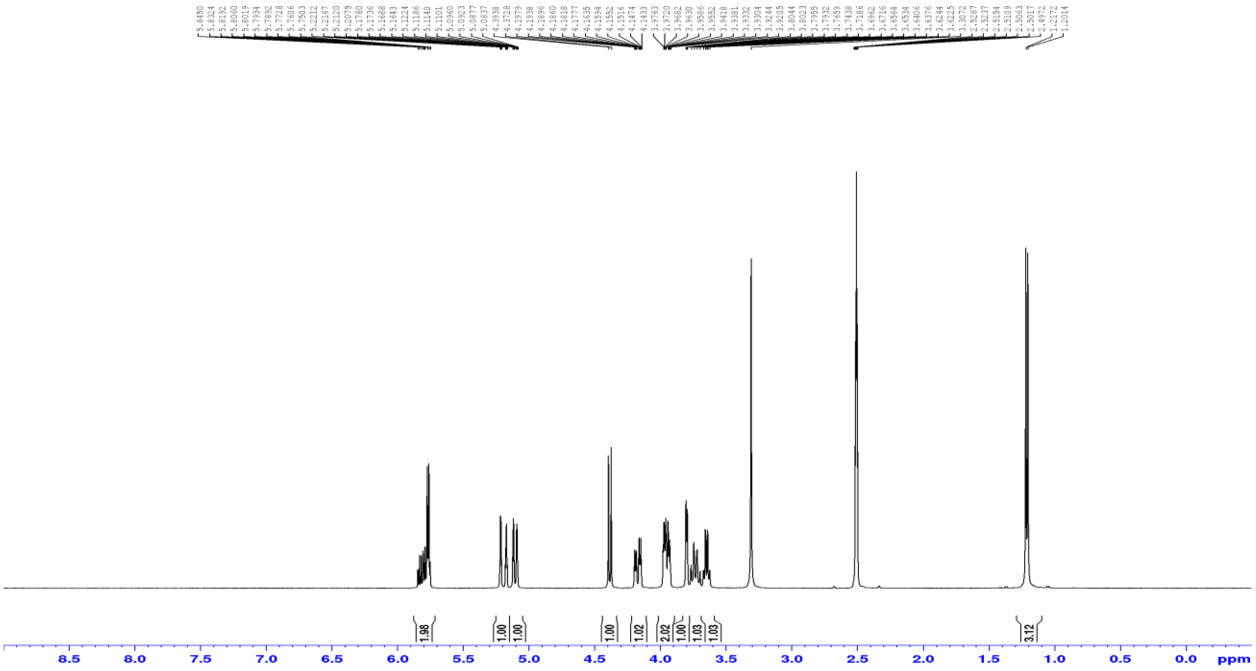

Figure. ^{1}H NMR (400 MHz, DMSO-d_6) spectrum of **35**.

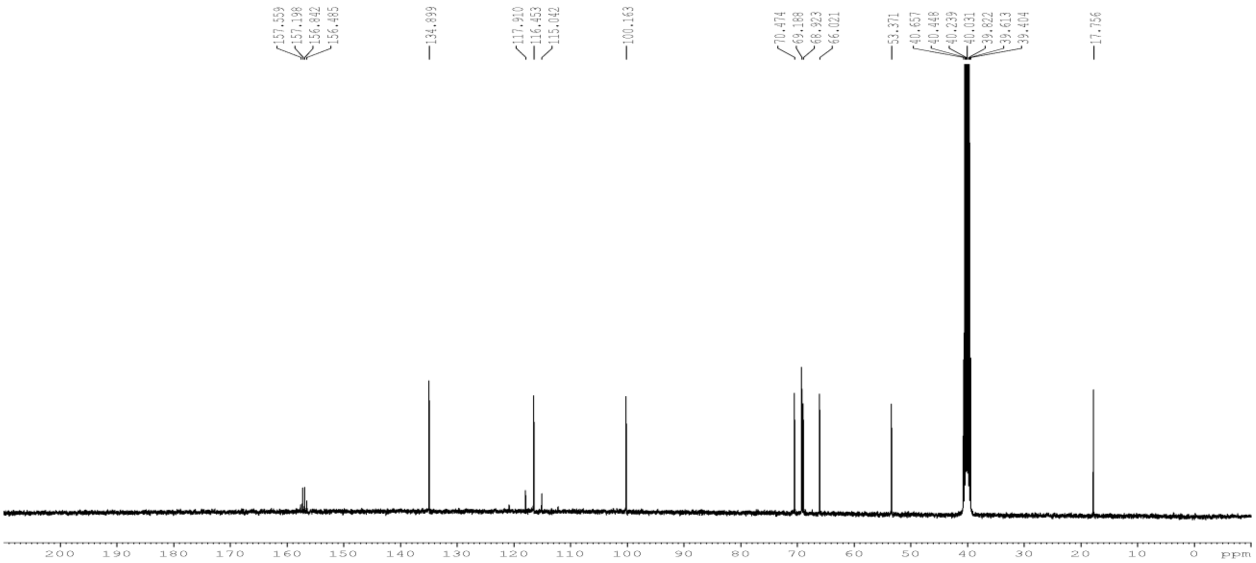

Figure. ^{13}C{^{1}H} NMR (100 MHz, DMSO-d_6) spectrum of **35**.

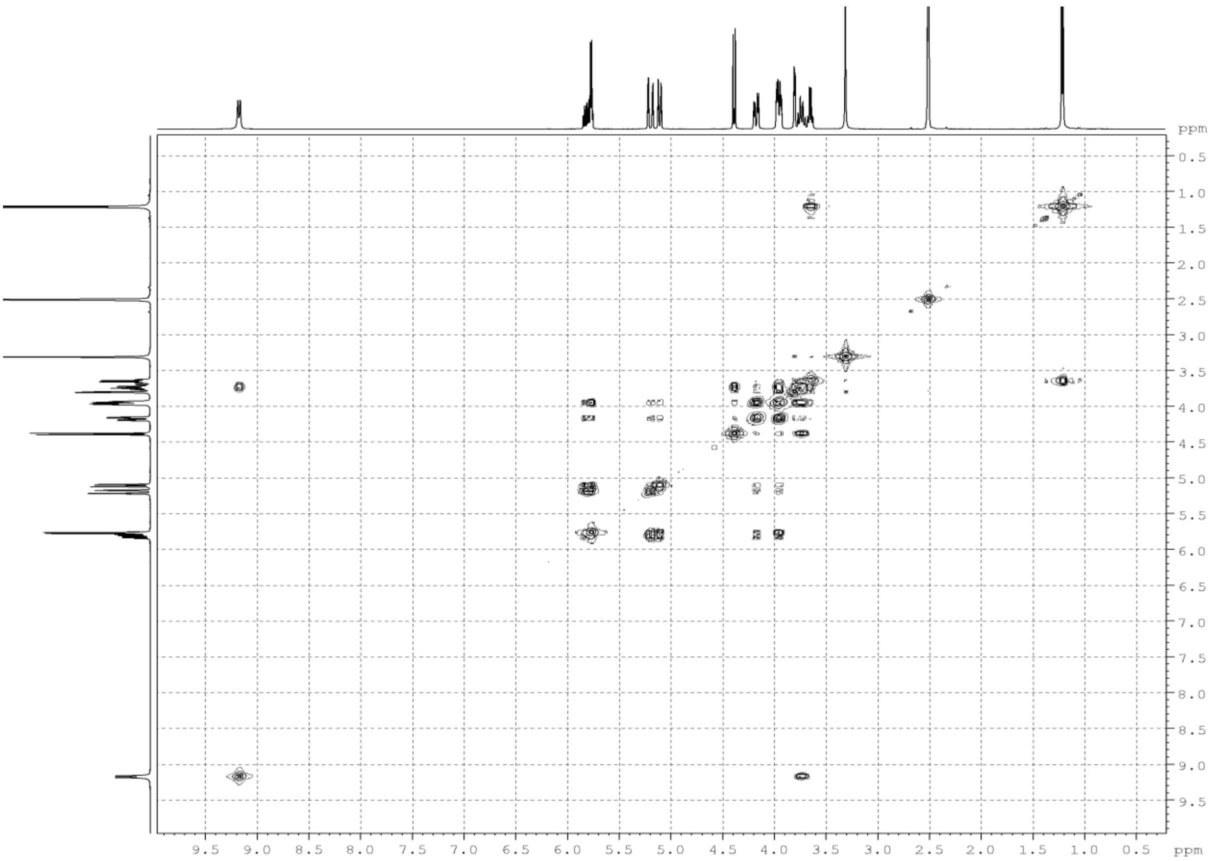

Figure. ^{1}H-^{1}H COSY NMR (400 MHz, DMSO-*d*$_6$) spectrum of **35**.

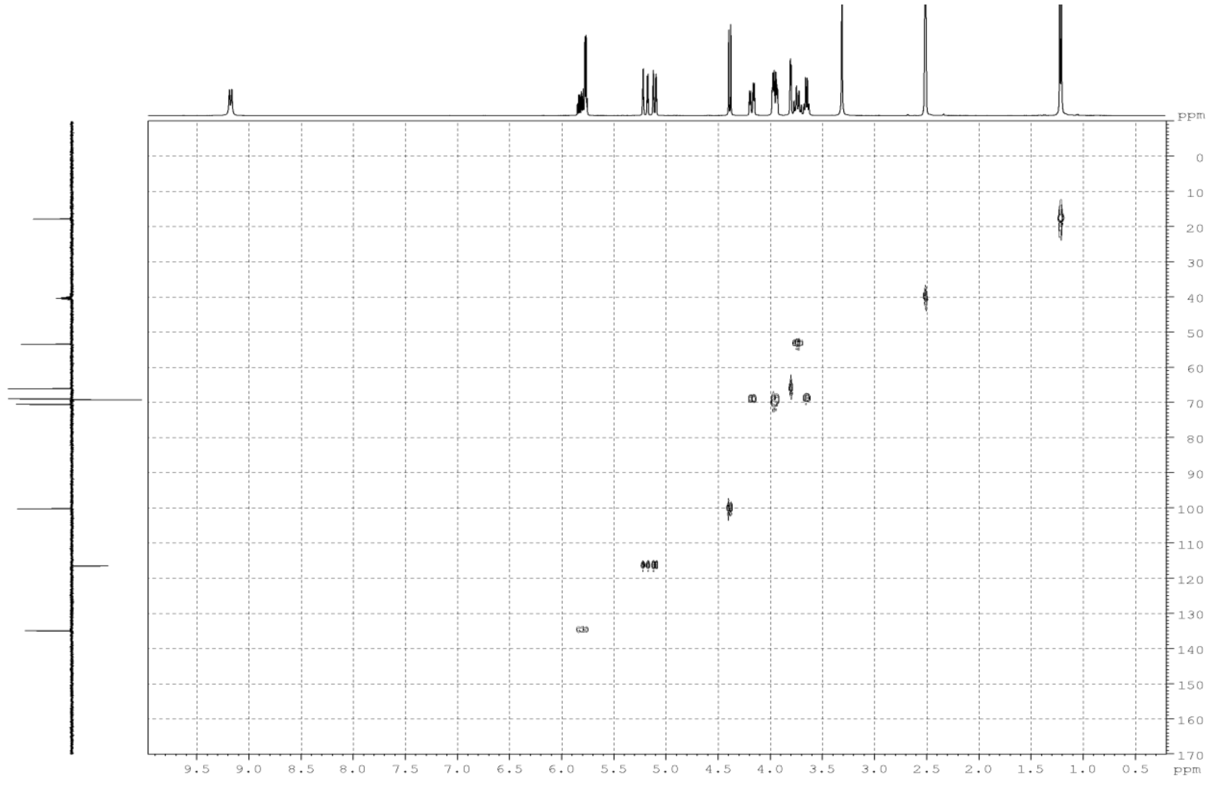

Figure. DEPT-HSQC NMR spectrum of **35**.

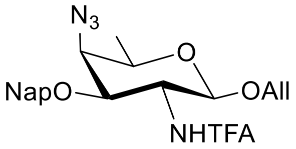

36

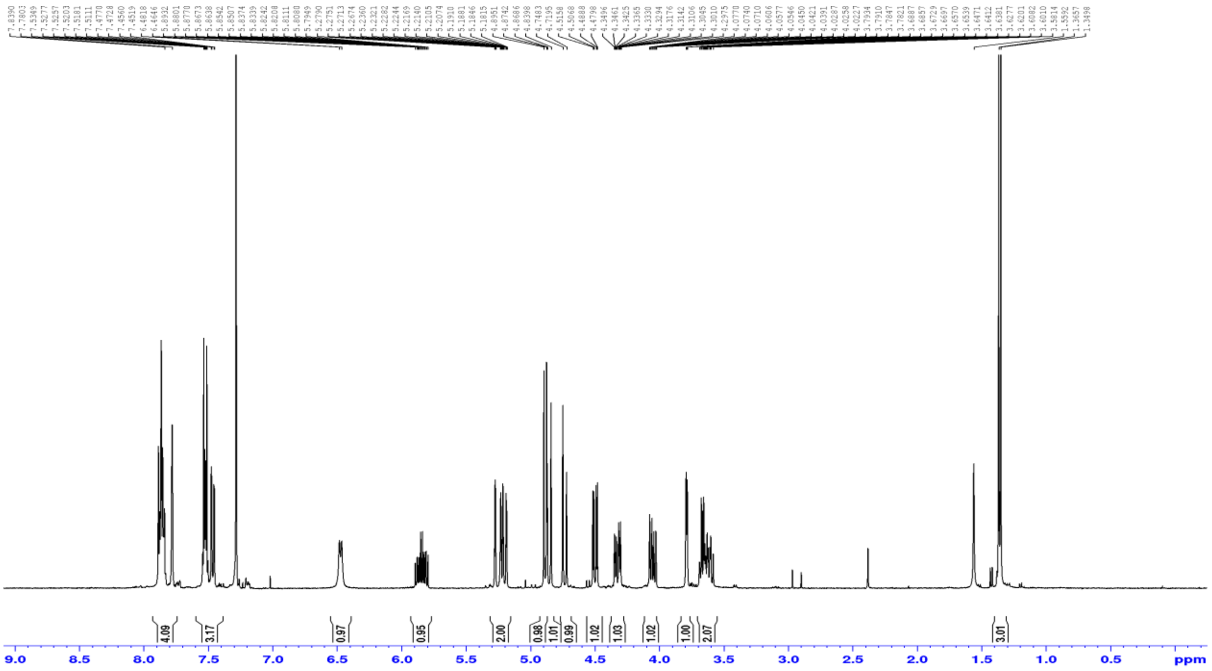

Figure. ^{1}H NMR (400 MHz, $CDCl_3$) spectrum of **36**.

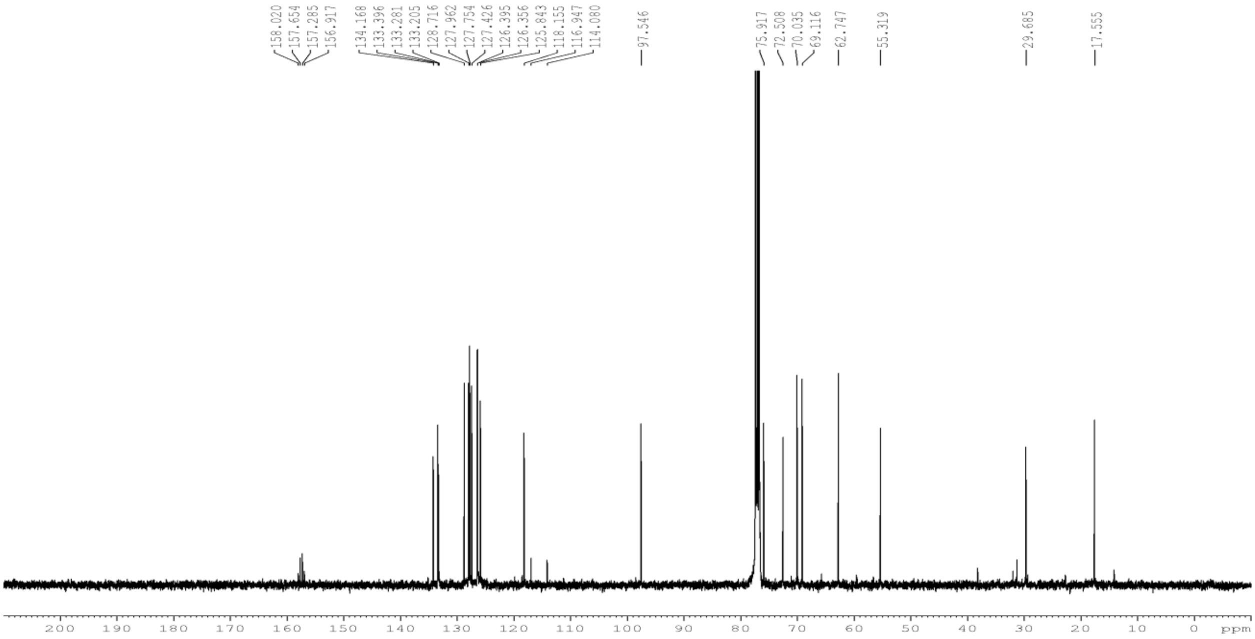

Figure. $^{13}C\{^{1}H\}$ NMR (100 MHz, $CDCl_3$) spectrum of **36**.

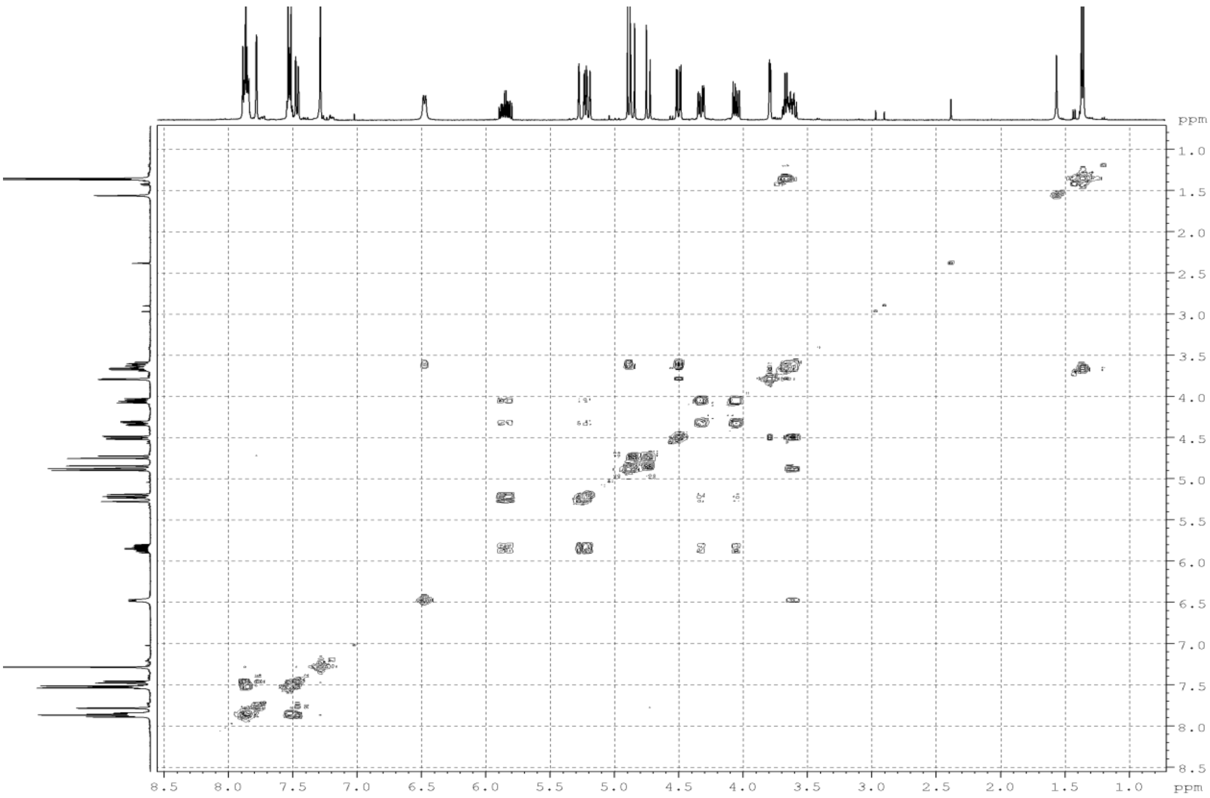

Figure. ^{1}H-^{1}H COSY NMR (400 MHz, $CDCl_3$) spectrum of **36**.

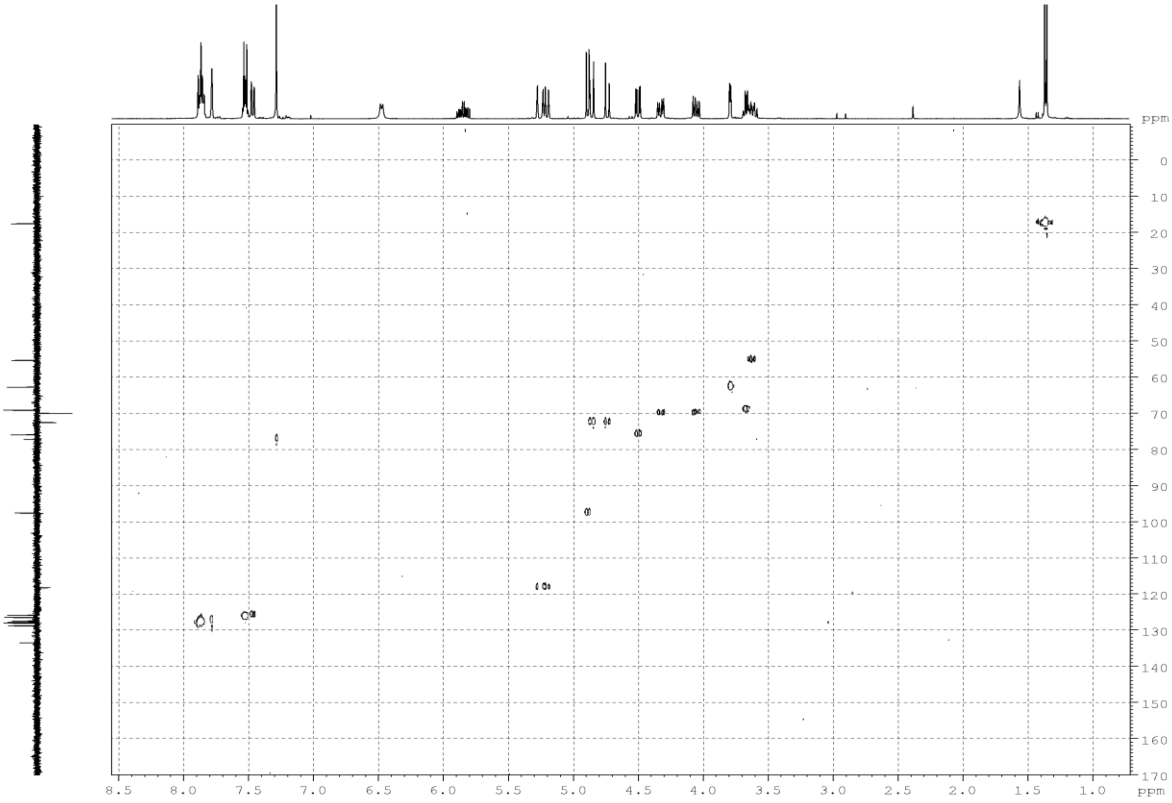

Figure. DEPT-HSQC NMR spectrum of **36**.

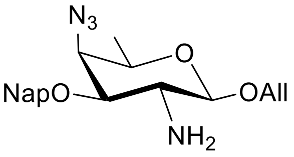

37

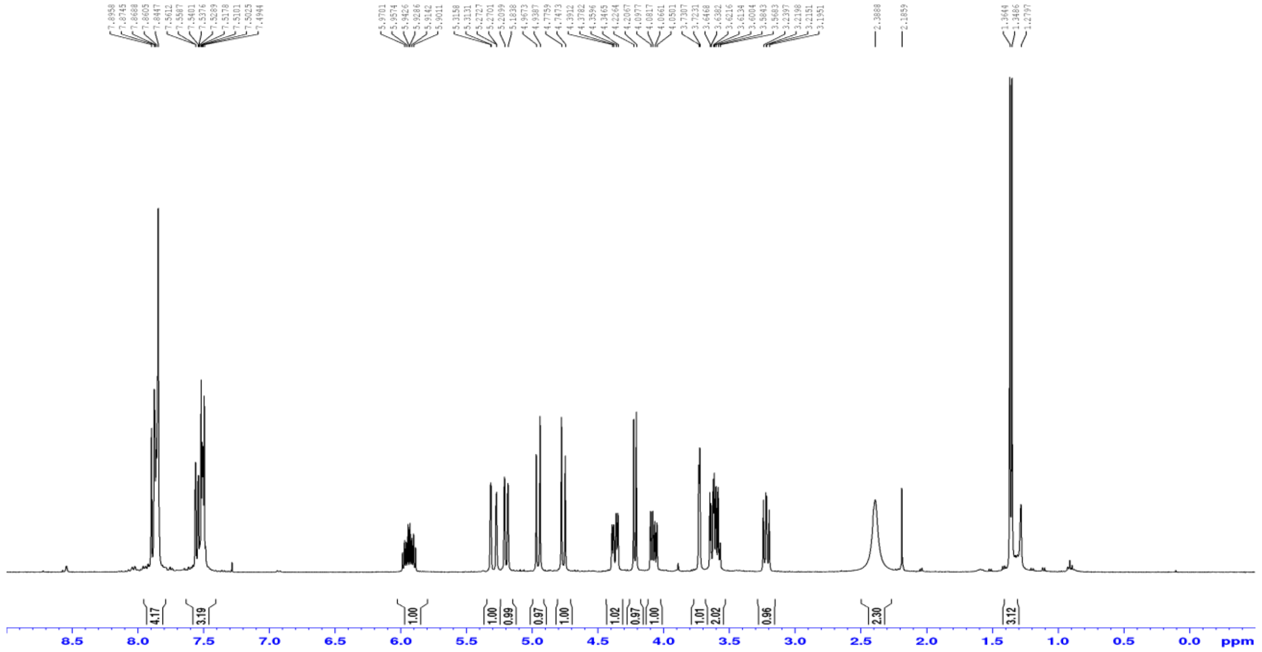

Figure. ^{1}H NMR (400 MHz, $CDCl_3$) spectrum of **37**.

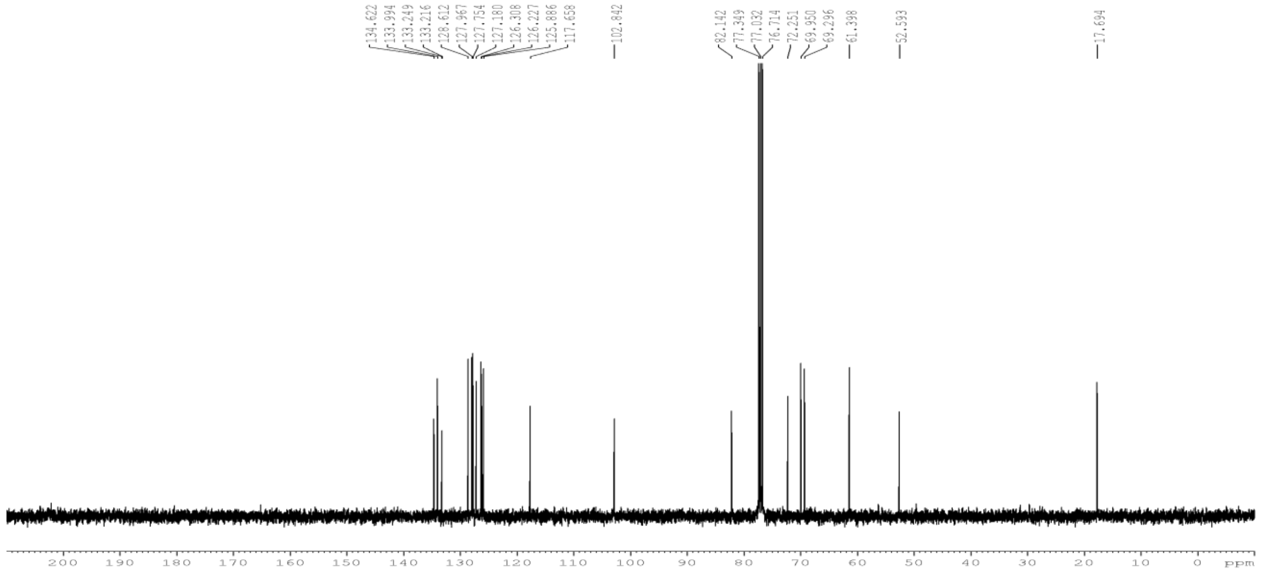

Figure. $^{13}C\{^{1}H\}$ NMR (100 MHz, $CDCl_3$) spectrum of **37**.

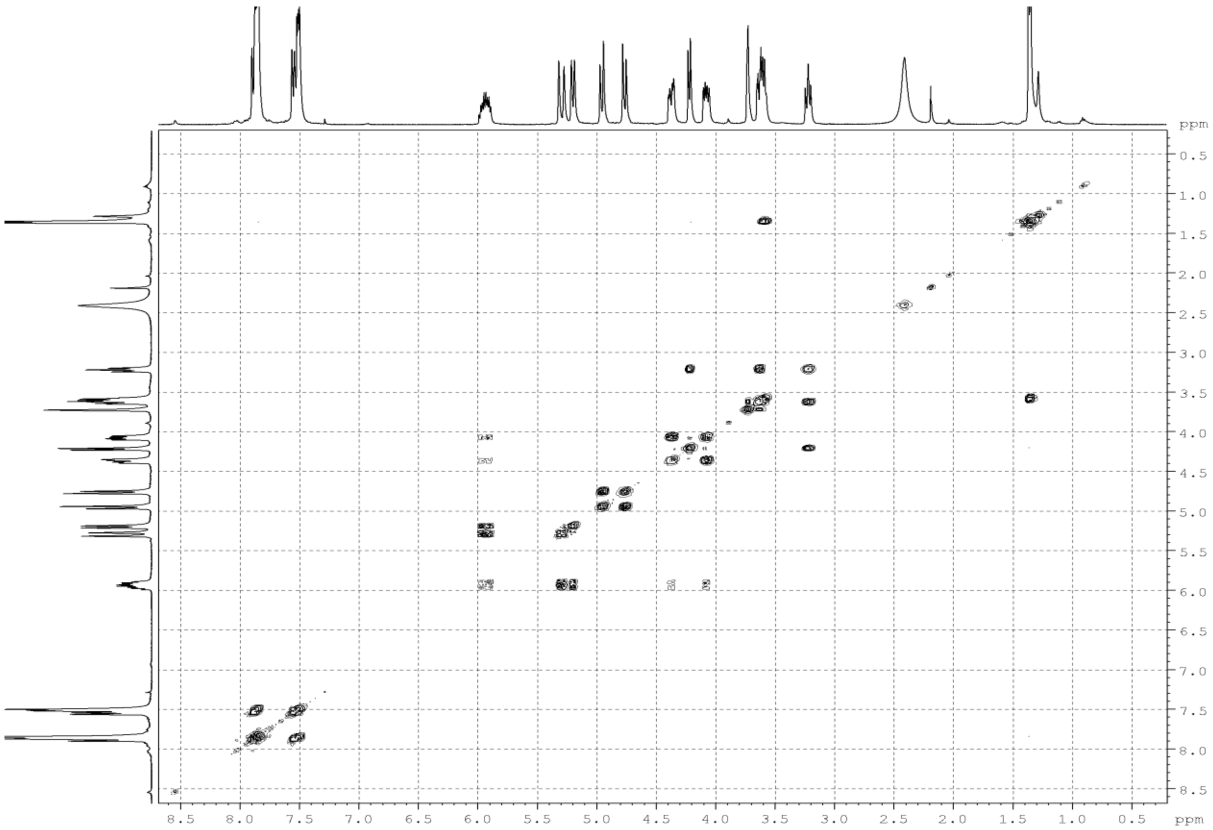

Figure. ^{1}H-^{1}H COSY NMR (400 MHz, $CDCl_3$) spectrum of **37**.

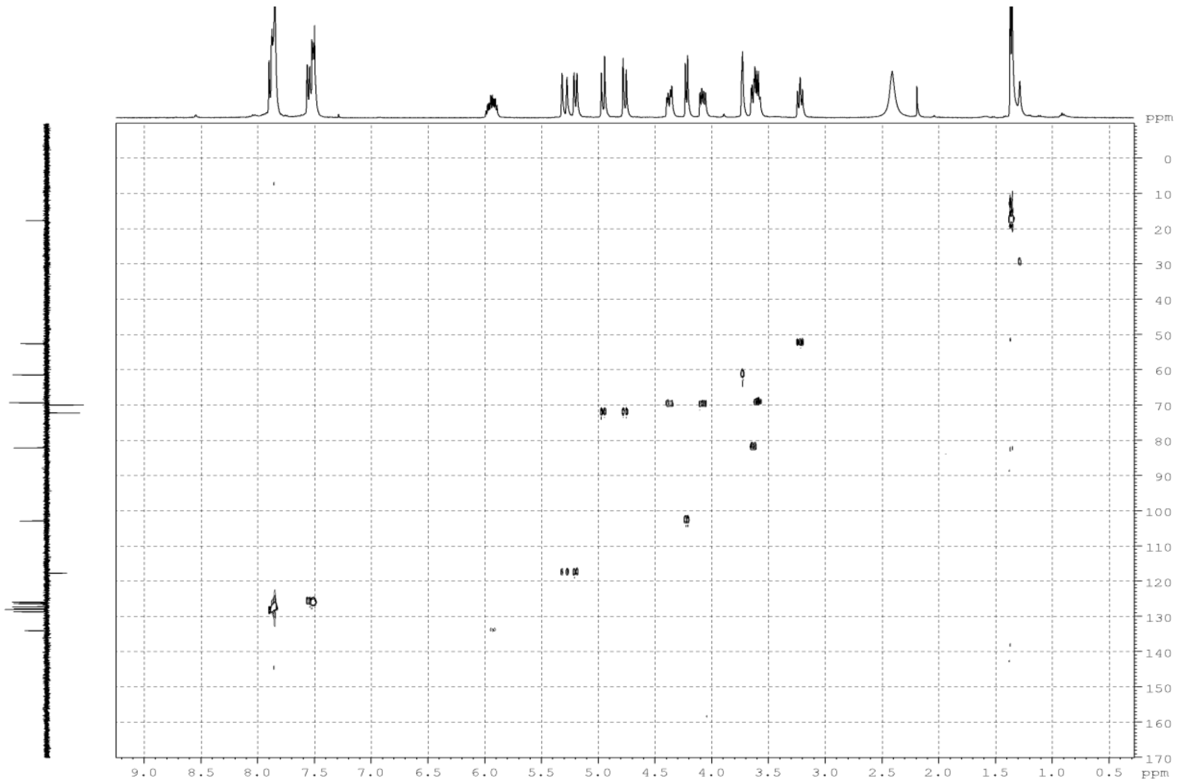

Figure. DEPT-HSQC NMR spectrum of **37**.

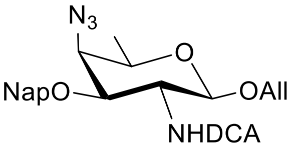

38

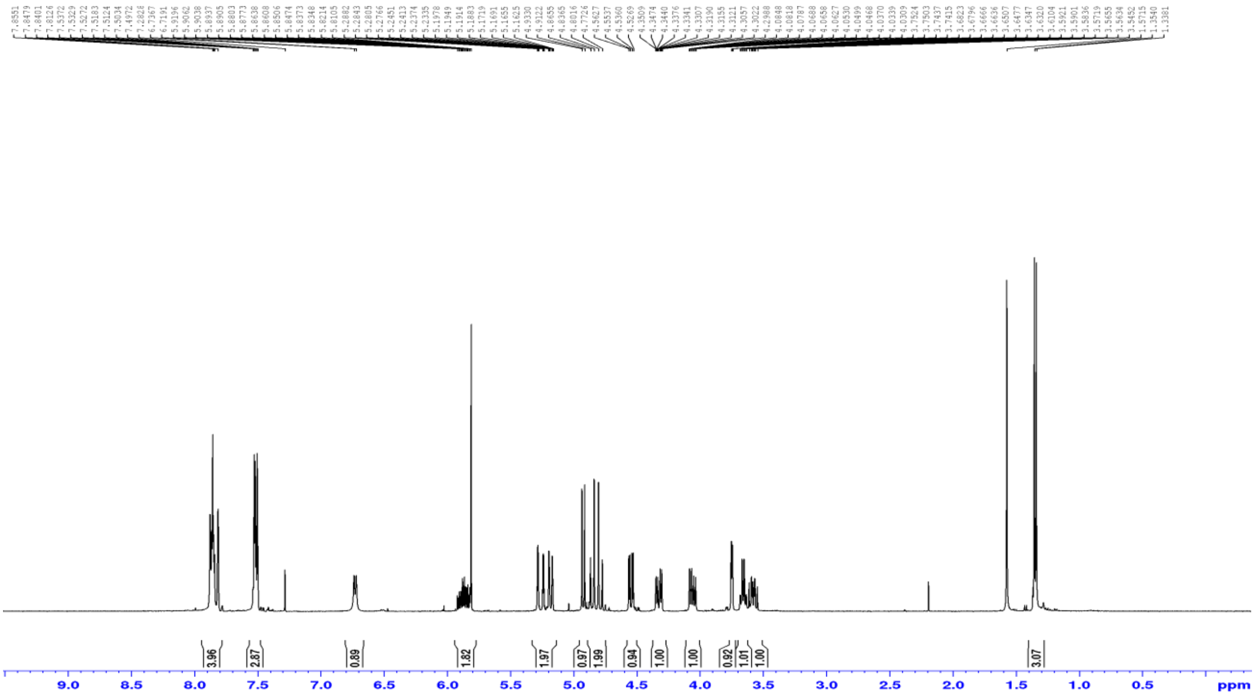

Figure. ^{1}H NMR (400 MHz, CDCl$_3$) spectrum of **38**.

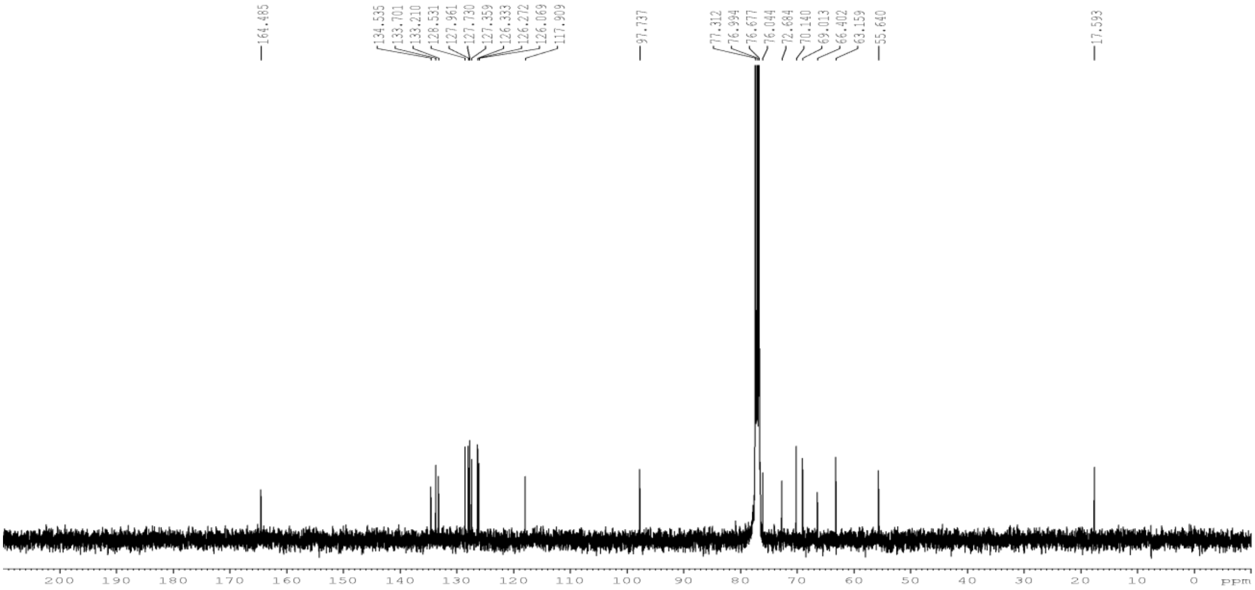

Figure. ^{13}C{^{1}H} NMR (100 MHz, CDCl$_3$) spectrum of **38**.

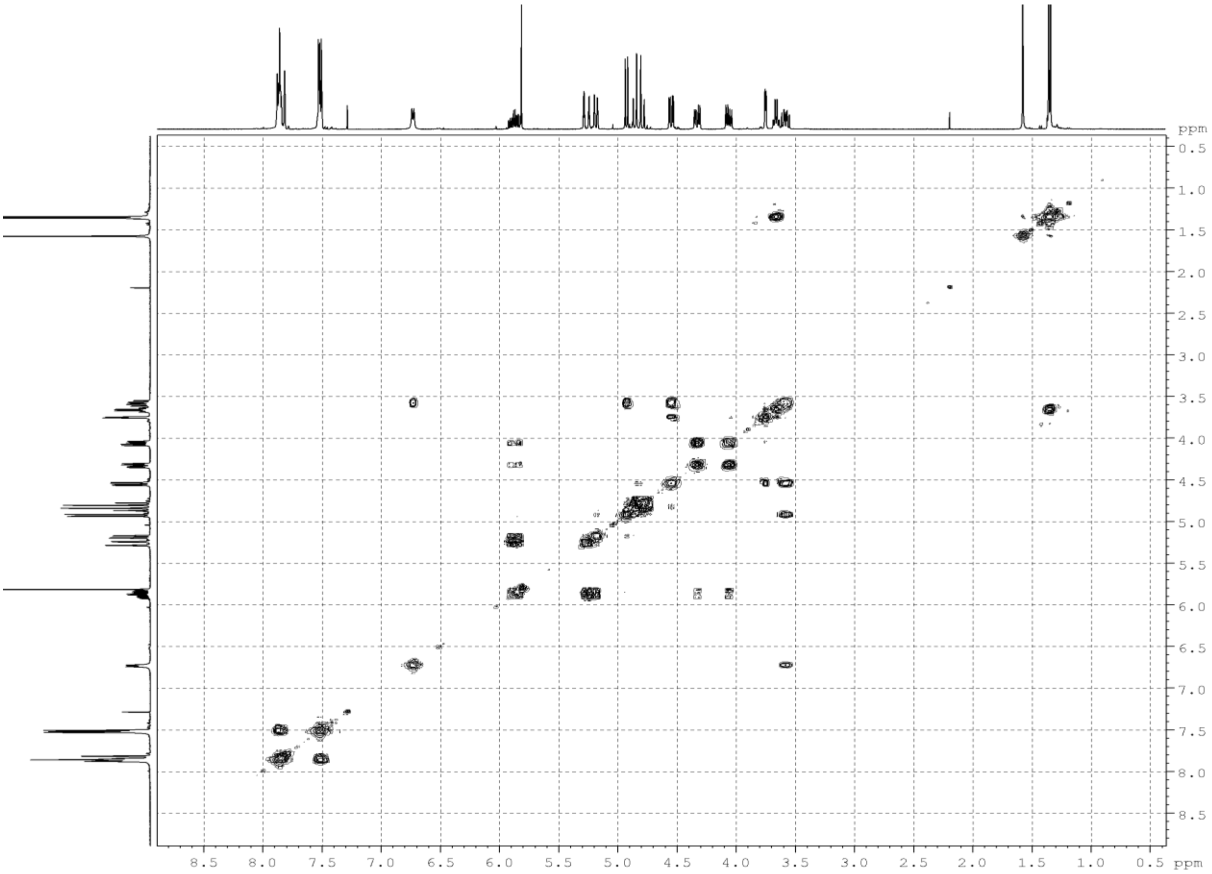

Figure. ^{1}H-^{1}H COSY NMR (400 MHz, $CDCl_3$) spectrum of **38**.

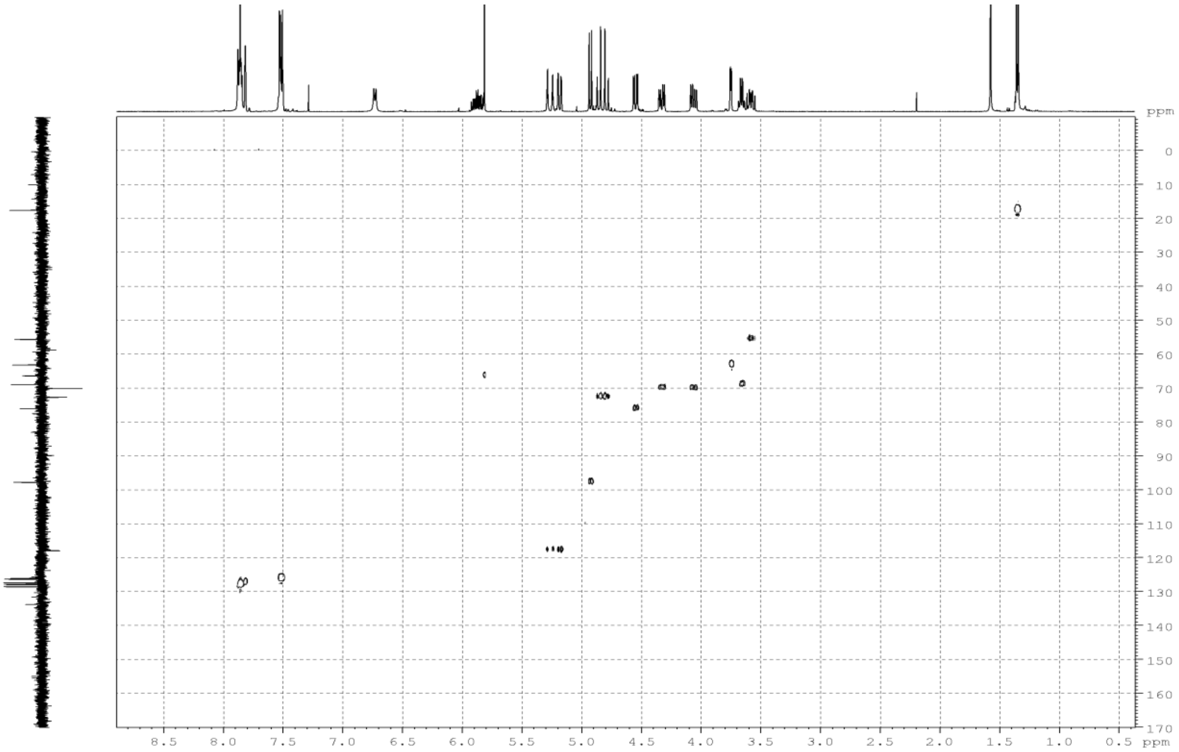

Figure. DEPT-HSQC NMR spectrum of **38**.

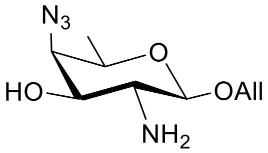

39

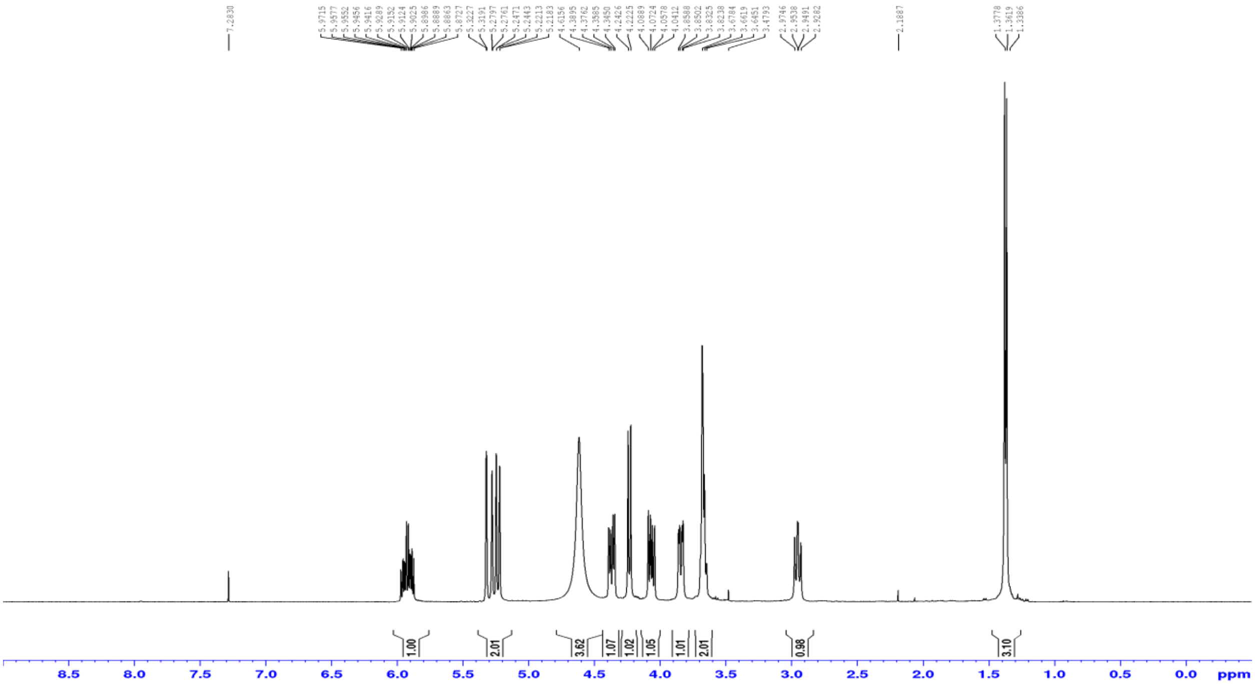

Figure. ^{1}H NMR (400 MHz, $CDCl_3$) spectrum of **39**.

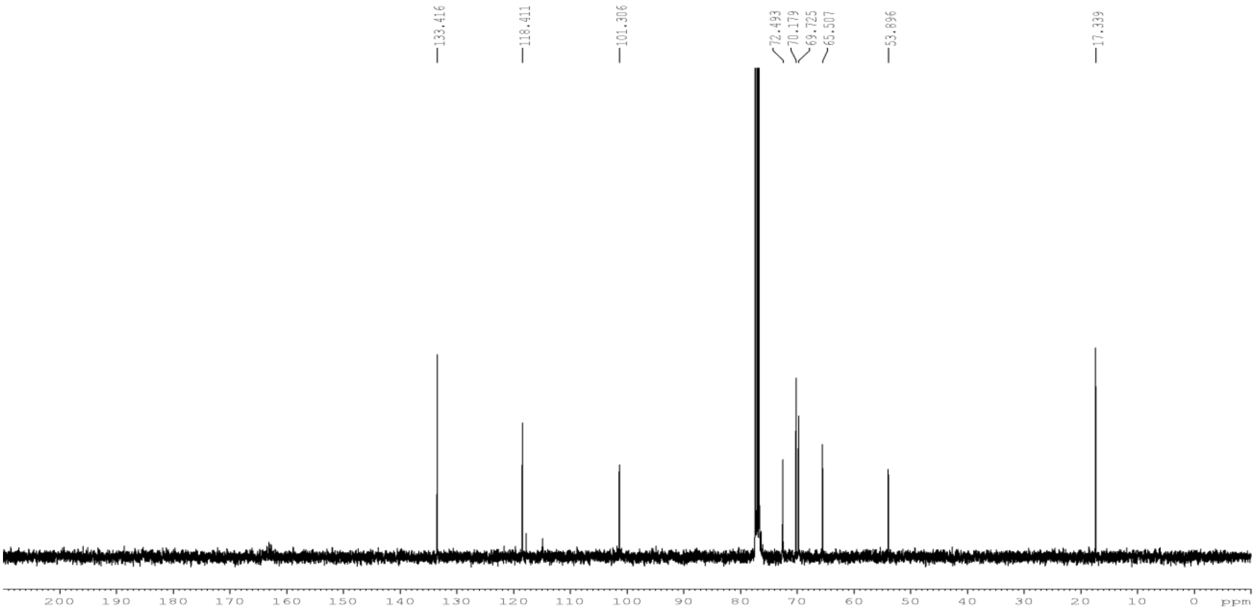

Figure. ^{13}C{^{1}H} NMR (100 MHz, $CDCl_3$) spectrum of **39**.

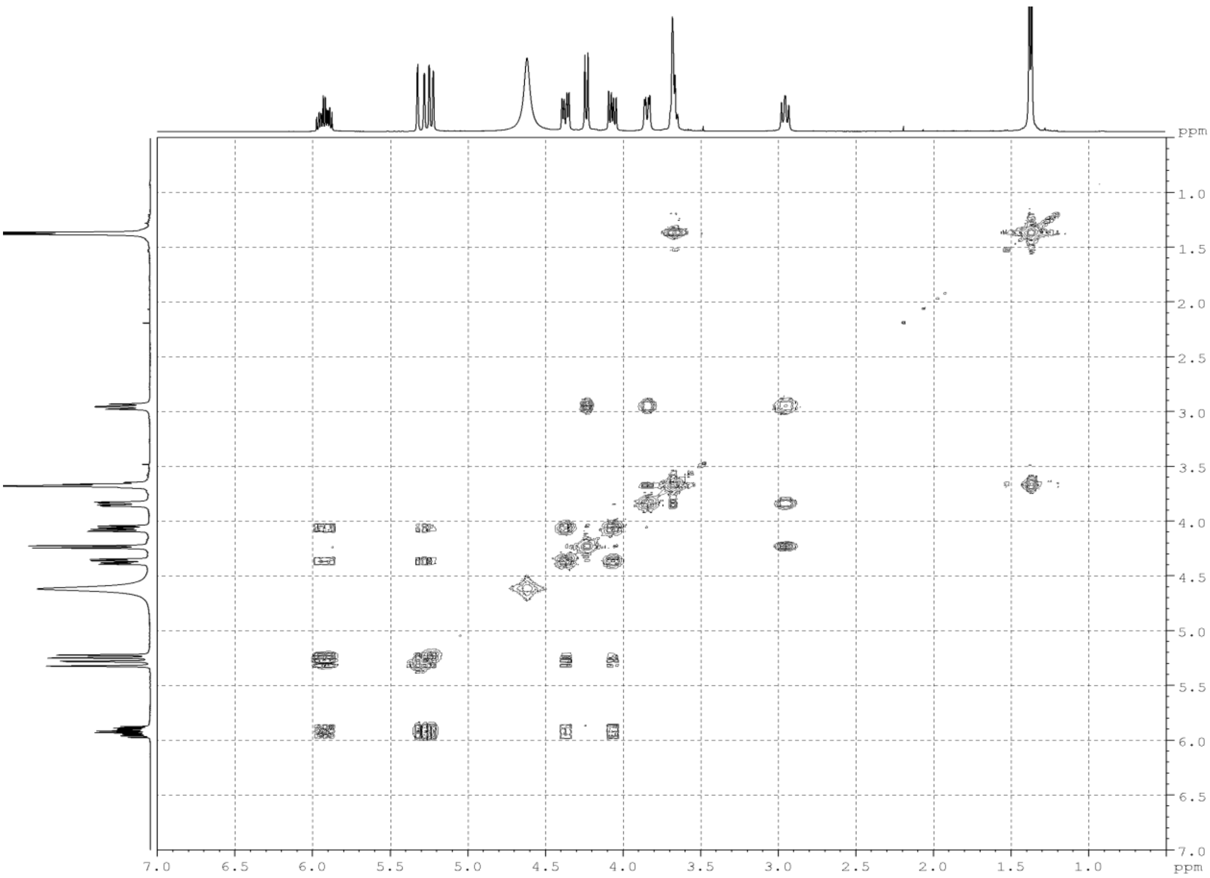

Figure. ^{1}H-^{1}H COSY NMR (400 MHz, $CDCl_3$) spectrum of **39**.

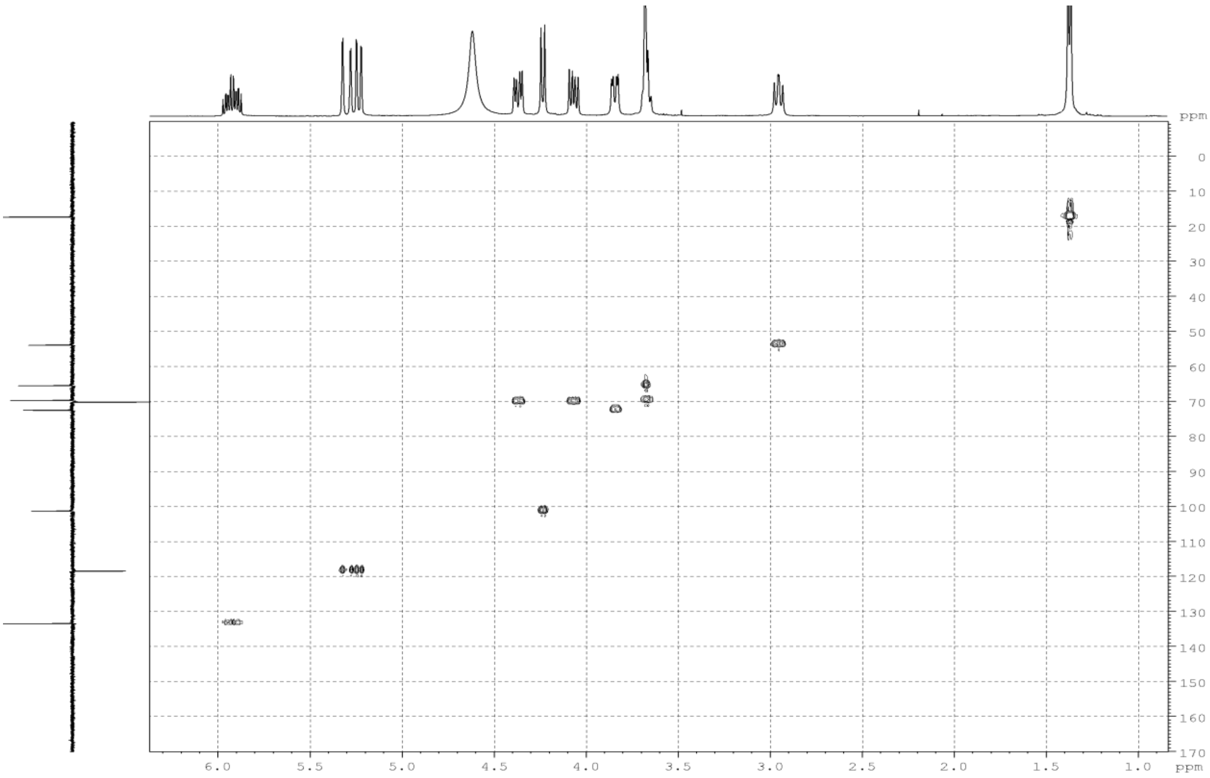

Figure. DEPT-HSQC NMR spectrum of **39**.

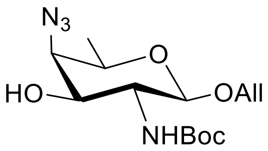

40

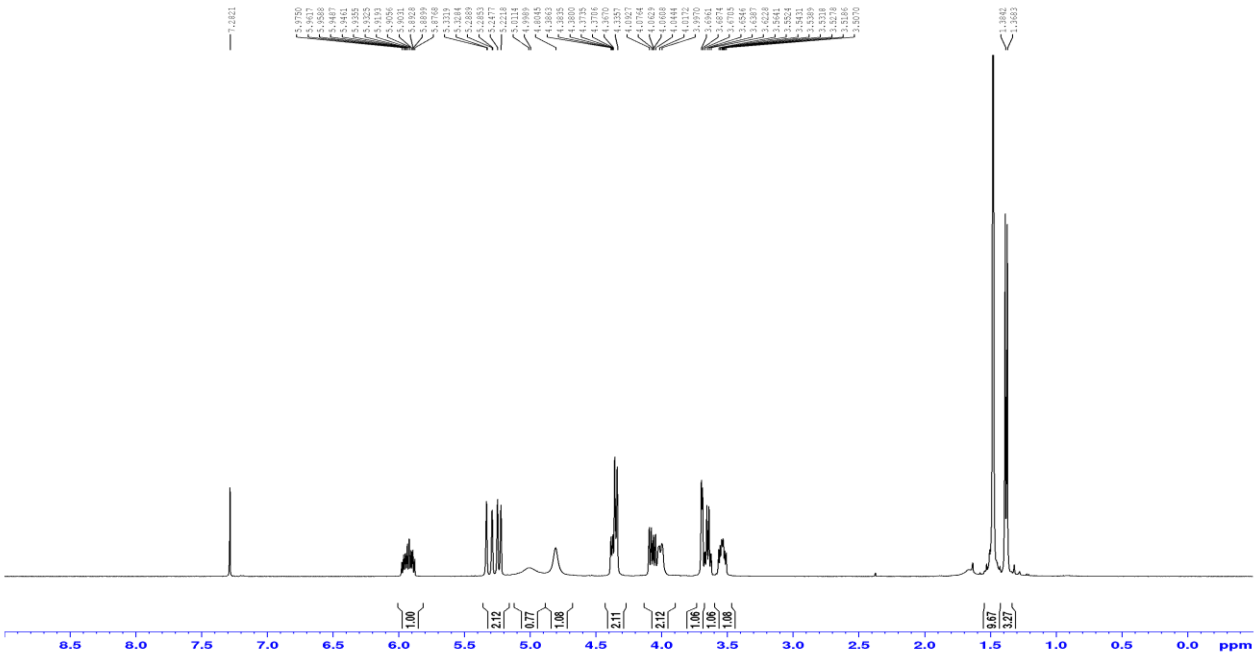

Figure. ^{1}H NMR (400 MHz, $CDCl_3$) spectrum of **40**.

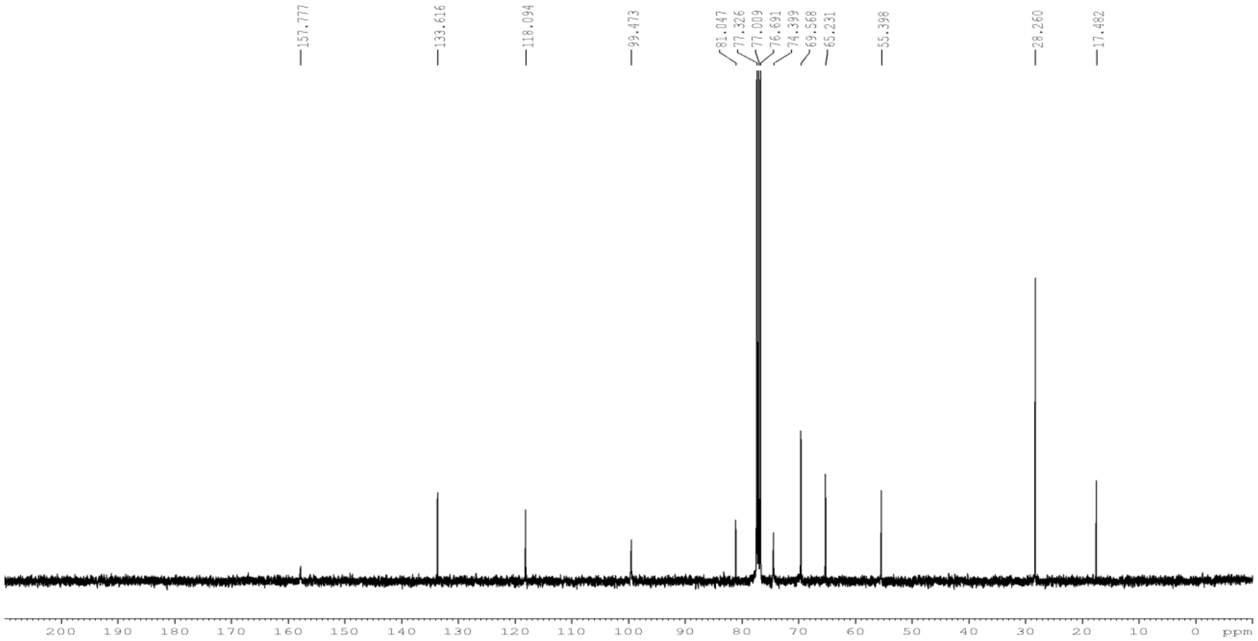

Figure. $^{13}C\{^{1}H\}$ NMR (100 MHz, $CDCl_3$) spectrum of **40**.

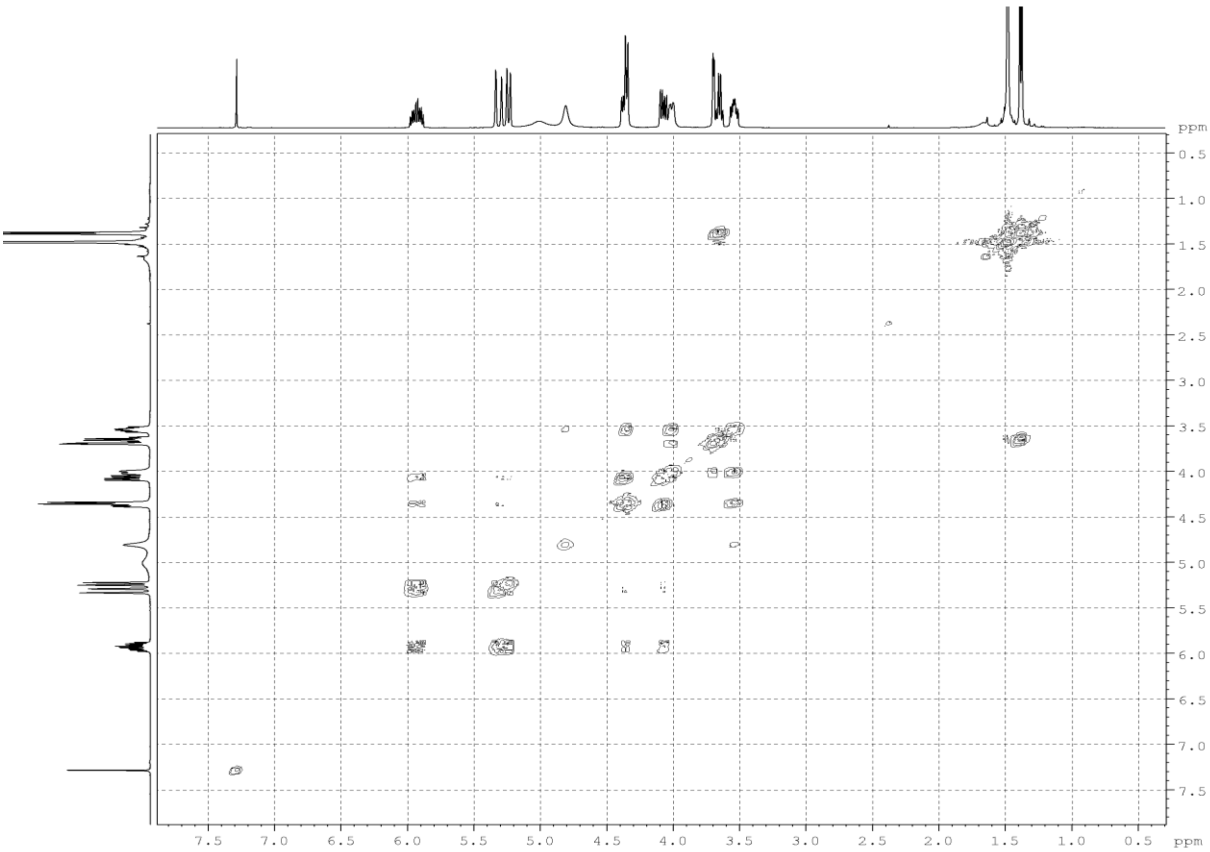

Figure. ^{1}H-^{1}H COSY NMR (400 MHz, $CDCl_3$) spectrum of **40**.

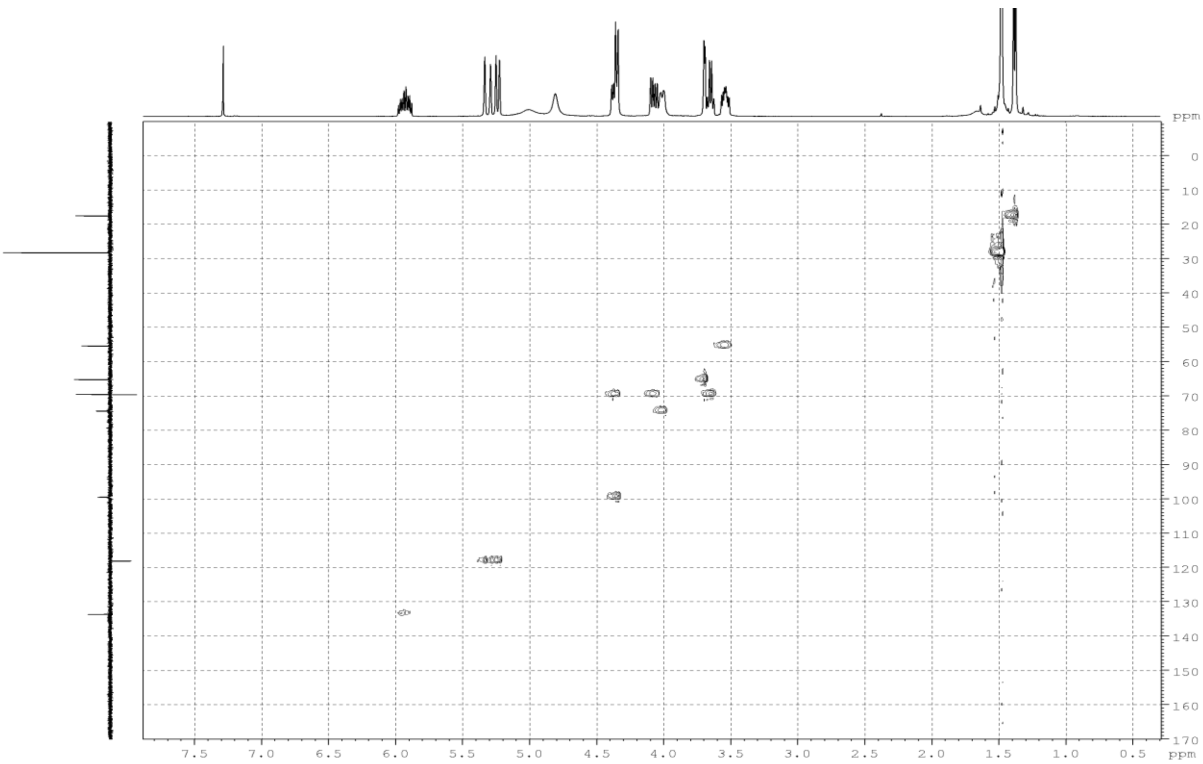

Figure. DEPT-HSQC NMR spectrum of **40**.

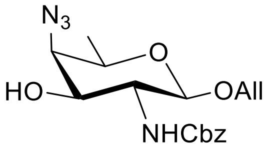

41

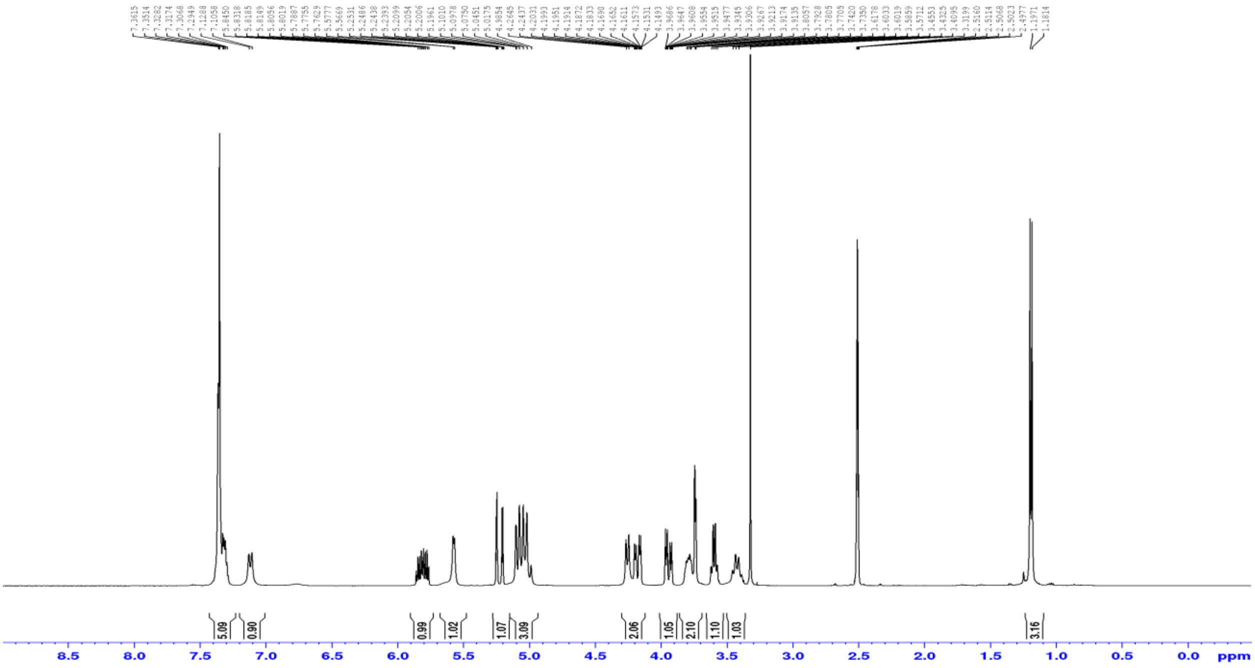

Figure. ^{1}H NMR (400 MHz, DMSO-d_6) spectrum of **41**.

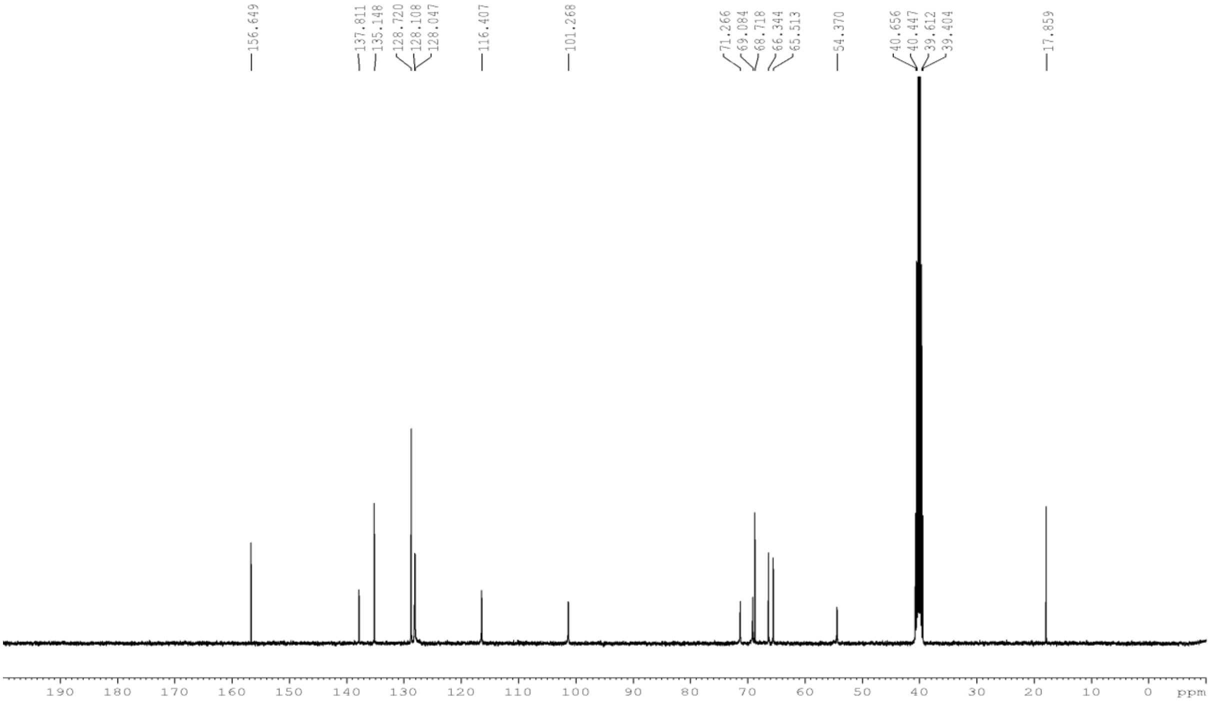

Figure. ^{13}C{^{1}H} NMR (100 MHz, DMSO-d_6) spectrum of **41**.

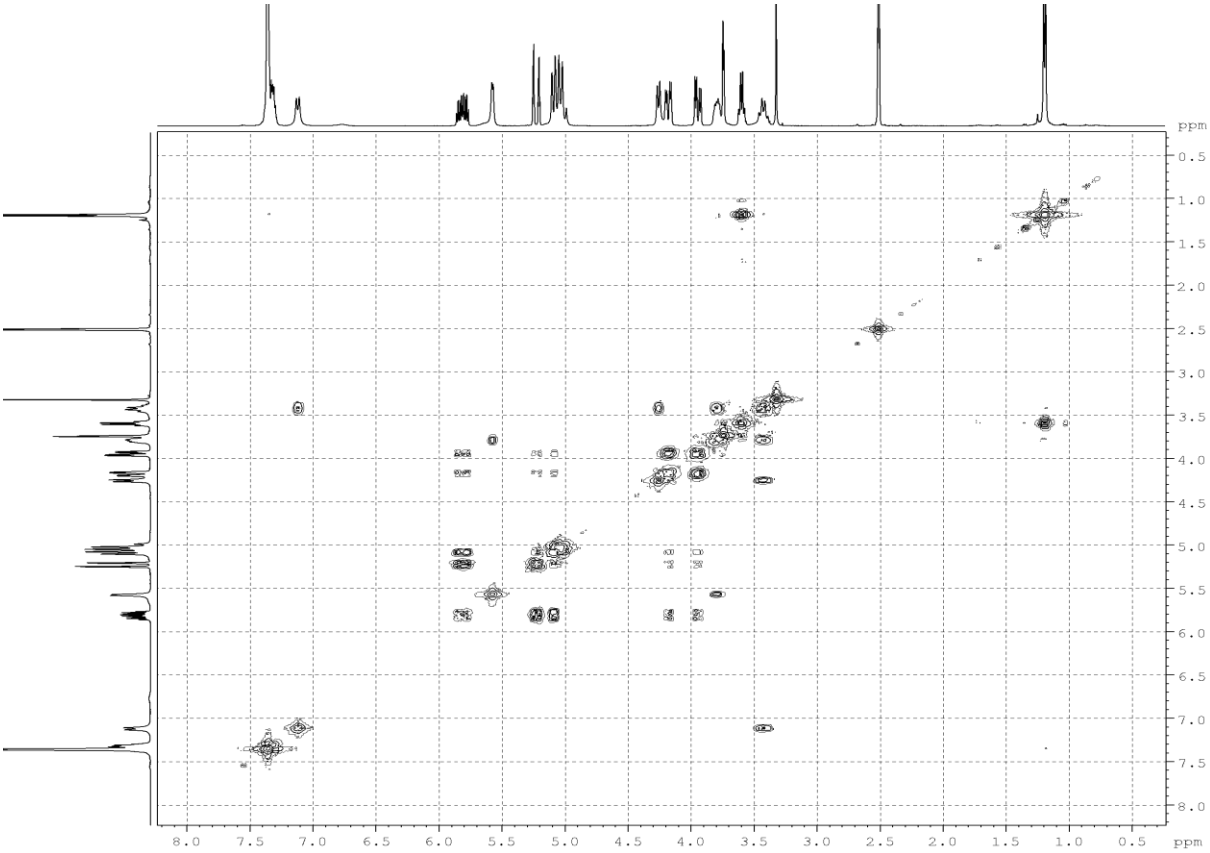

Figure. ^{1}H-^{1}H COSY NMR (400 MHz, DMSO-d_6) spectrum of **41**.

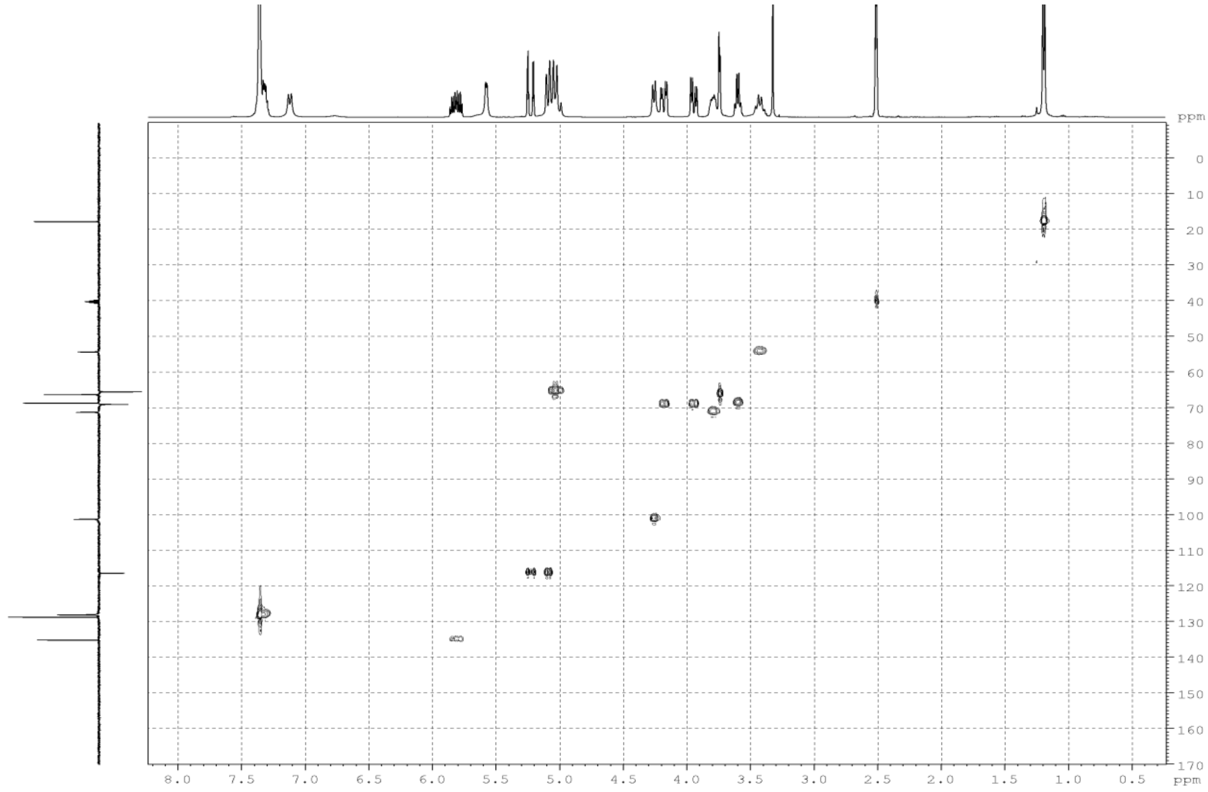

Figure. DEPT-HSQC NMR spectrum of **41**.

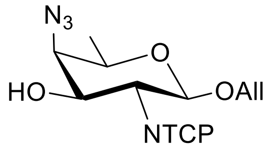

42

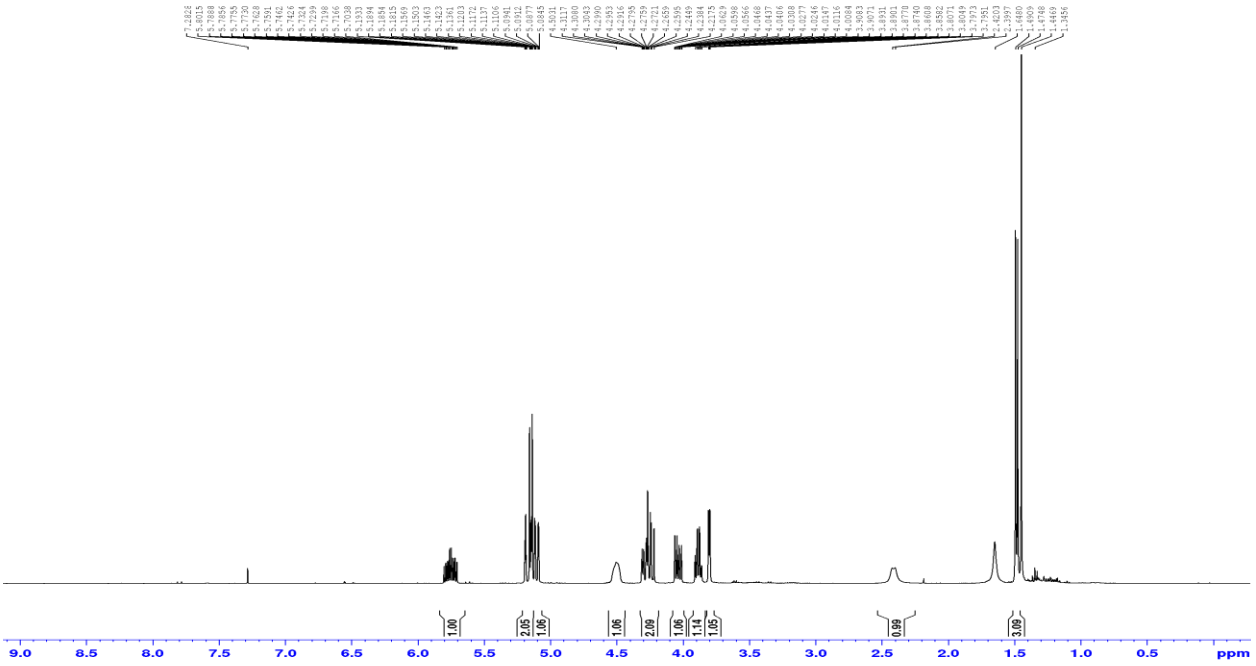

Figure. ^{1}H NMR (400 MHz, $CDCl_3$) spectrum of **42**.

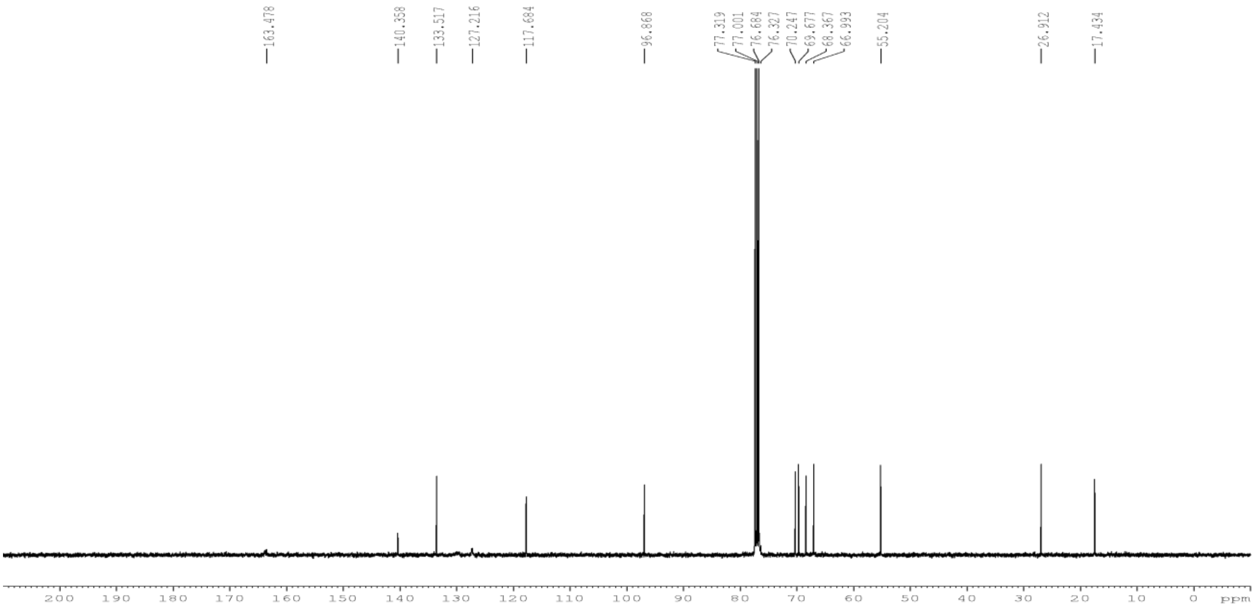

Figure. ^{13}C{^{1}H} NMR (100 MHz, $CDCl_3$) spectrum of **42**.

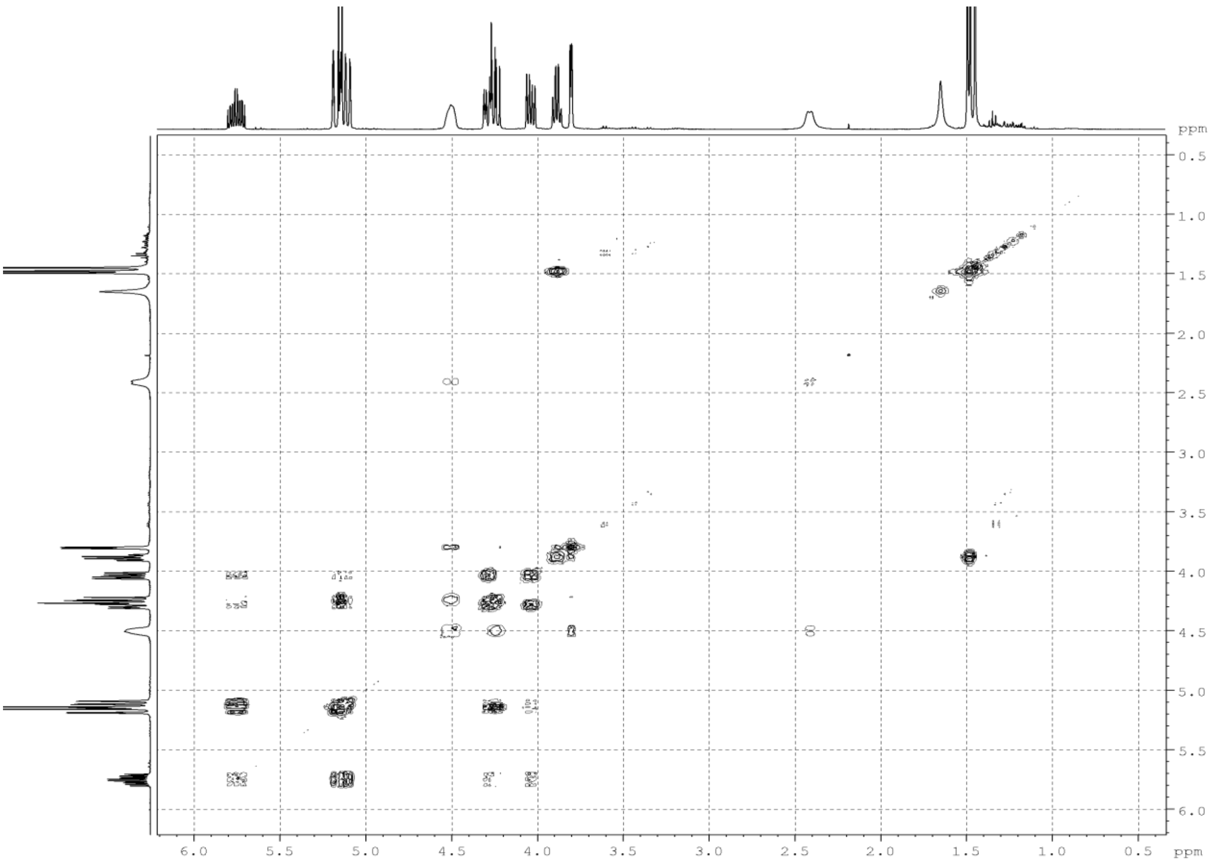

Figure. ^{1}H-^{1}H COSY NMR (400 MHz, $CDCl_3$) spectrum of **42**.

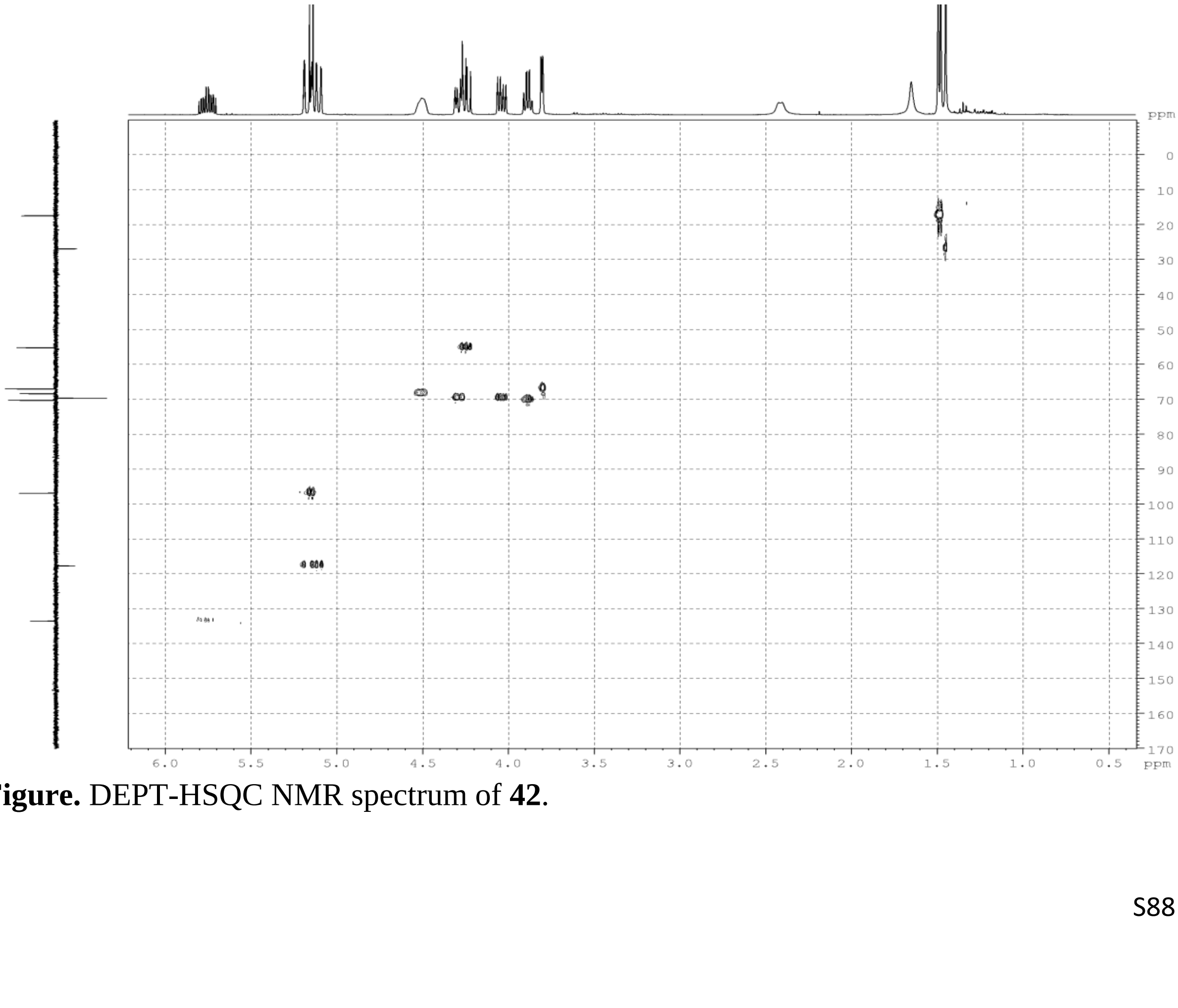

Figure. DEPT-HSQC NMR spectrum of **42**.

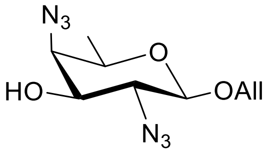

43

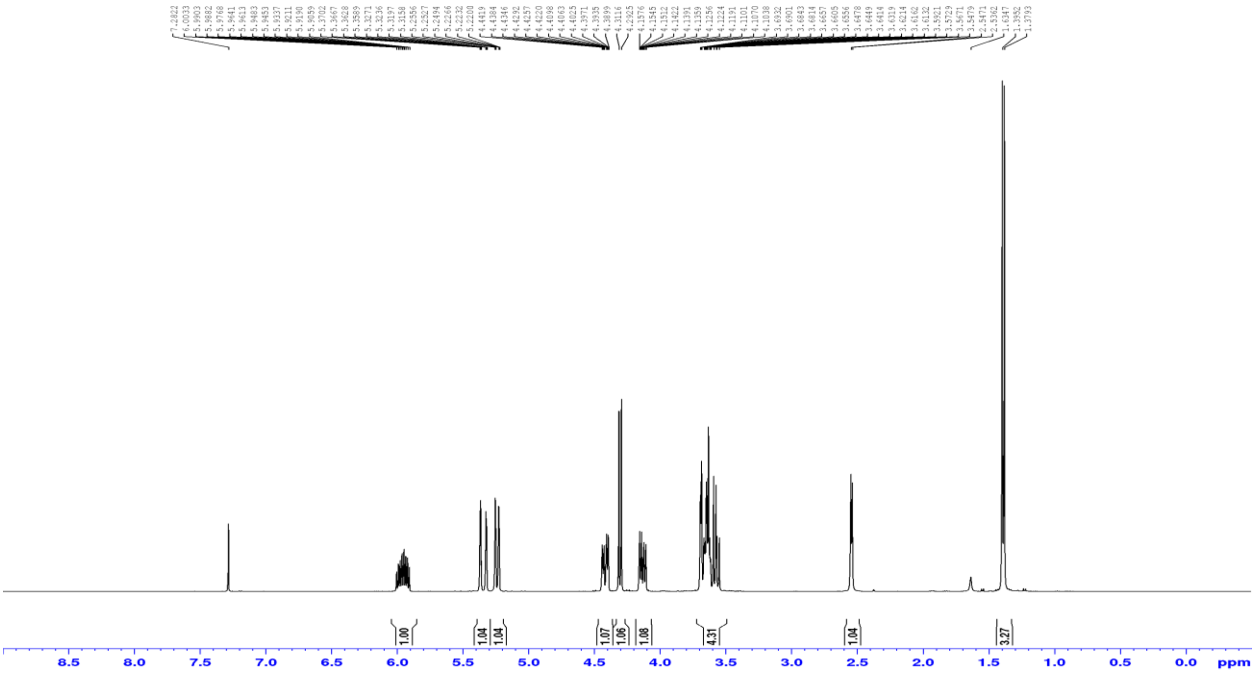

Figure. ^{1}H NMR (400 MHz, $CDCl_3$) spectrum of **43**.

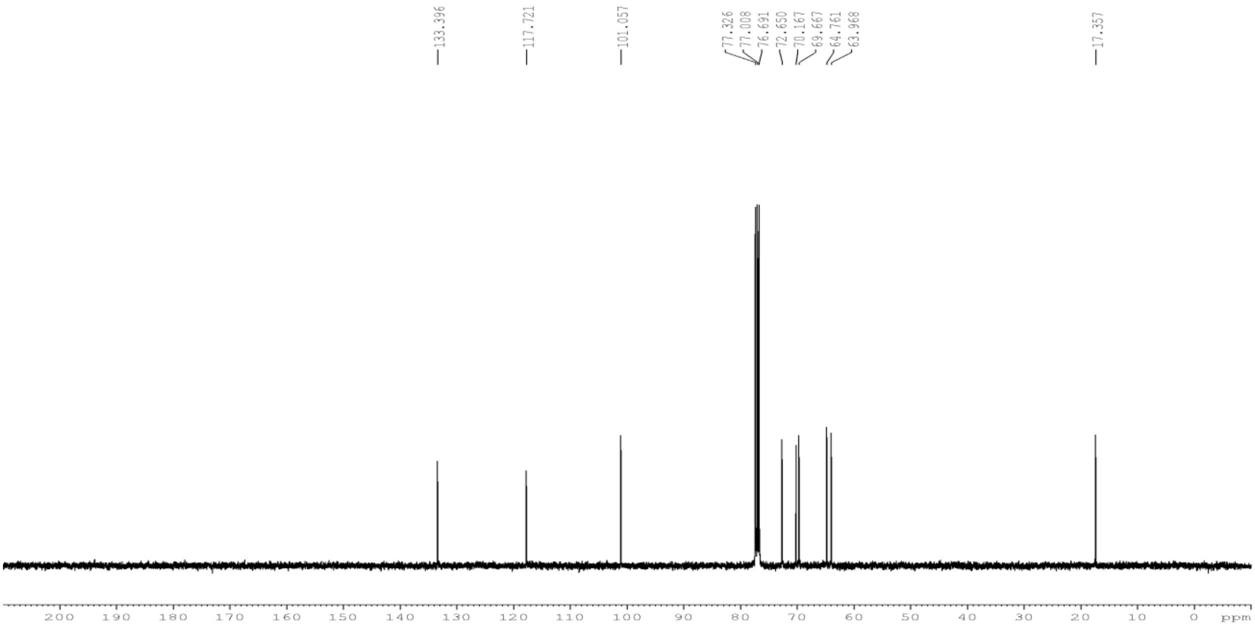

Figure. $^{13}C\{^{1}H\}$ NMR (100 MHz, $CDCl_3$) spectrum of **43**.

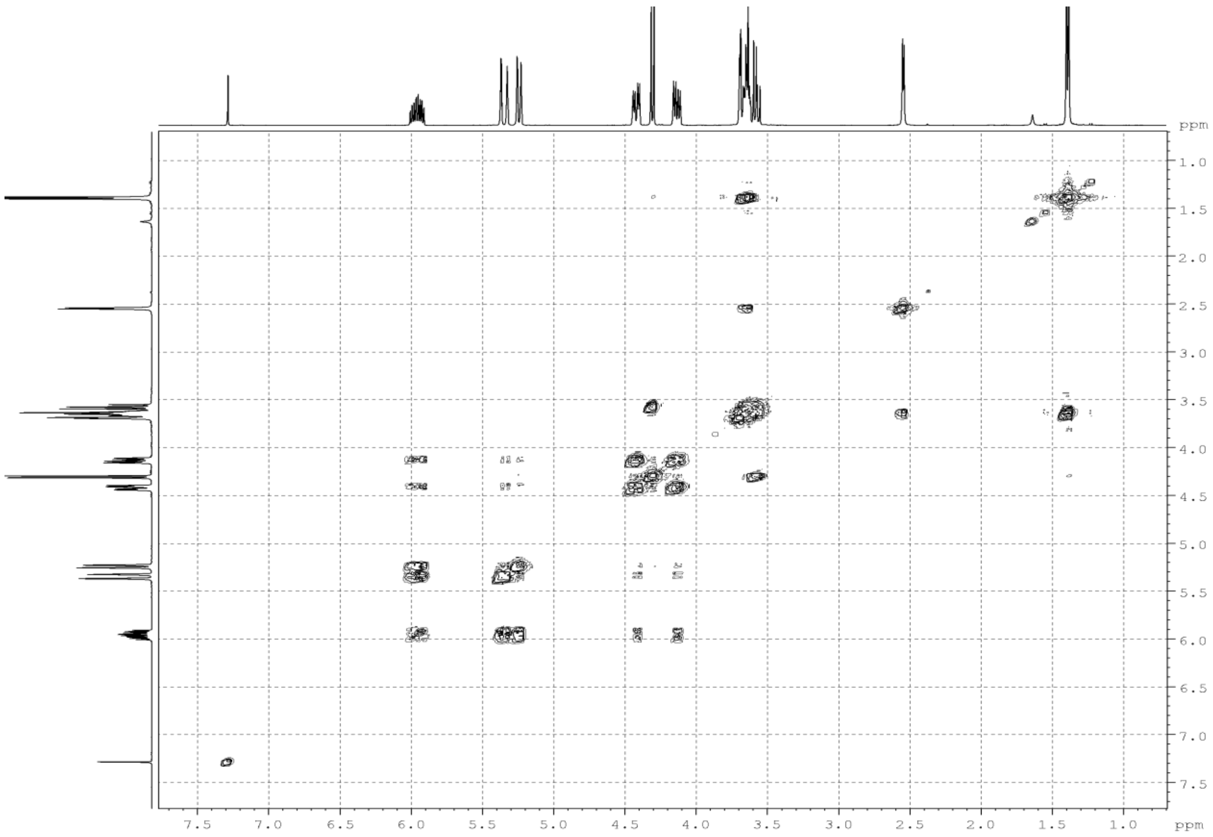

Figure. ^{1}H-^{1}H COSY NMR (400 MHz, $CDCl_3$) spectrum of **43**.

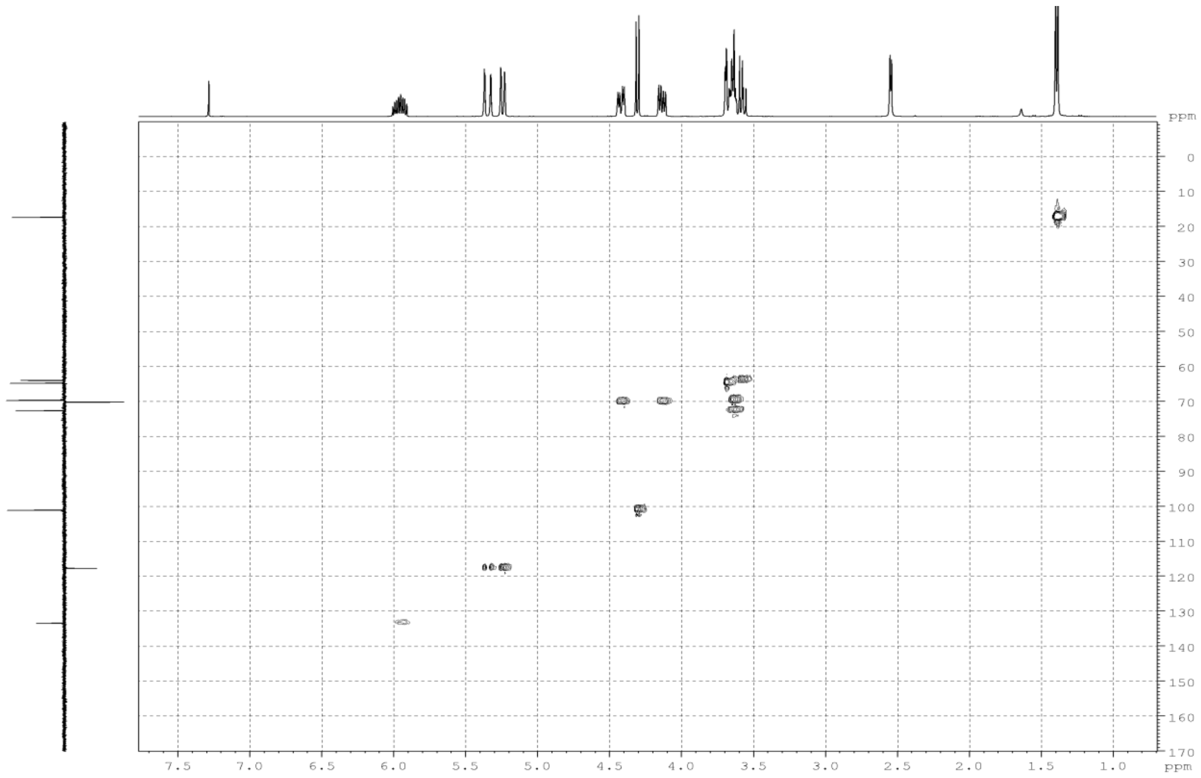

Figure. DEPT-HSQC NMR spectrum of **43**.

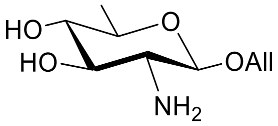

44

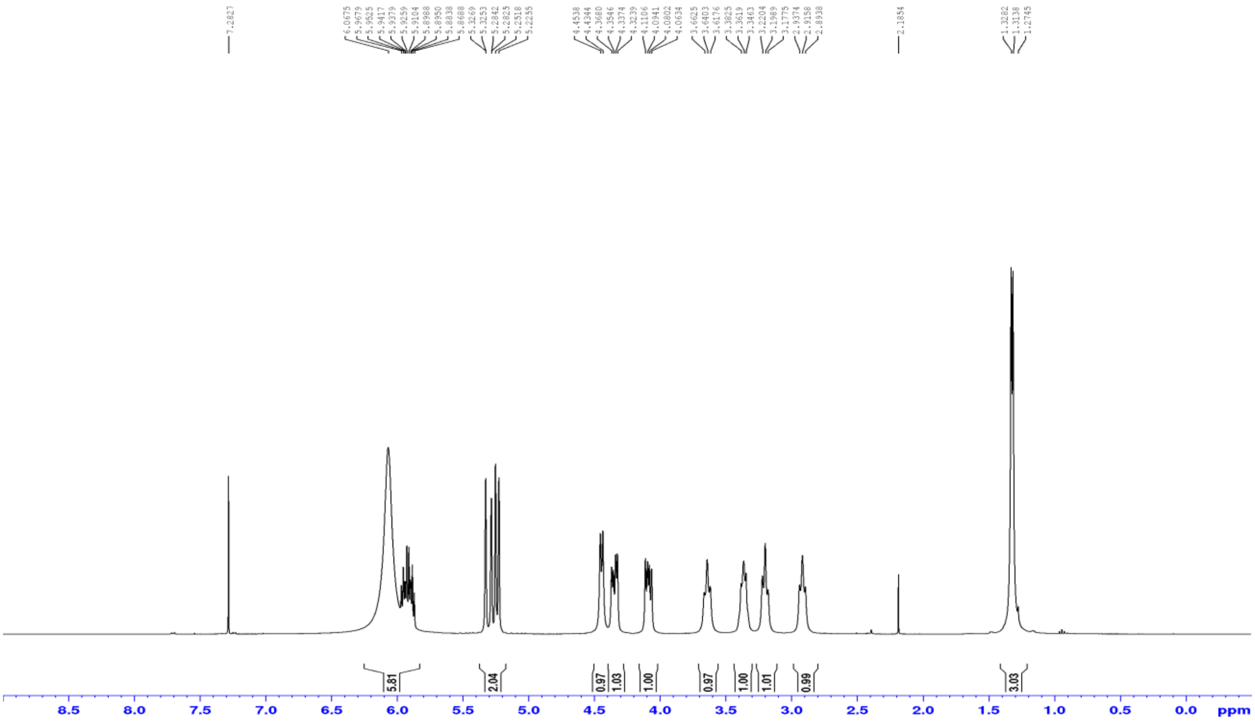

Figure. ^{1}H NMR (400 MHz, $CDCl_3$) spectrum of **44**.

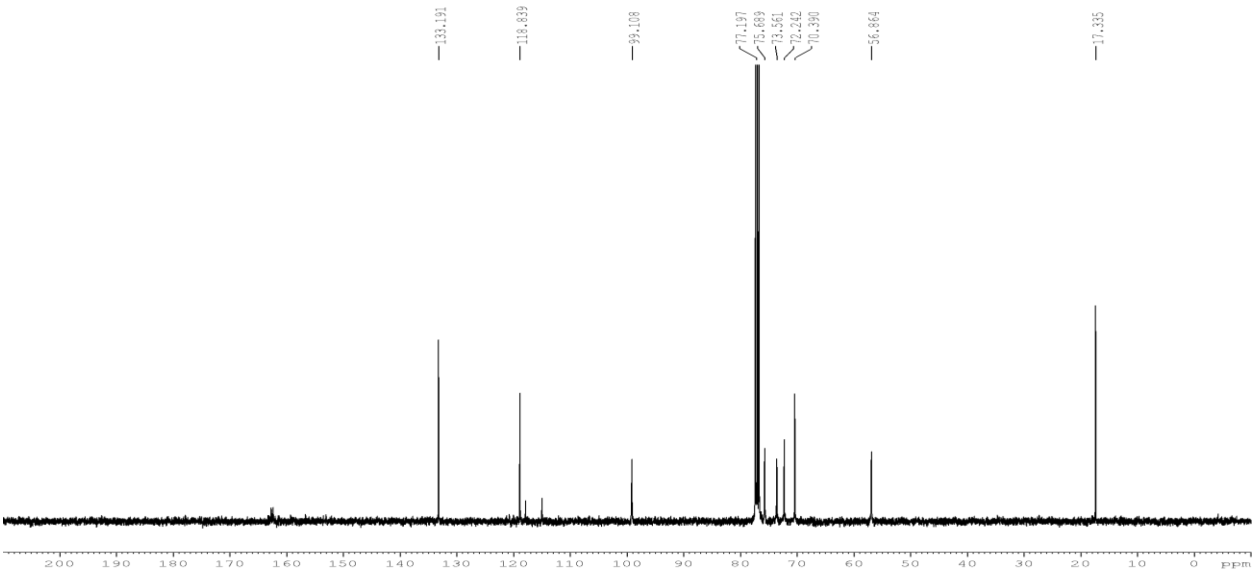

Figure. ^{13}C{^{1}H} NMR (100 MHz, $CDCl_3$) spectrum of **44**.

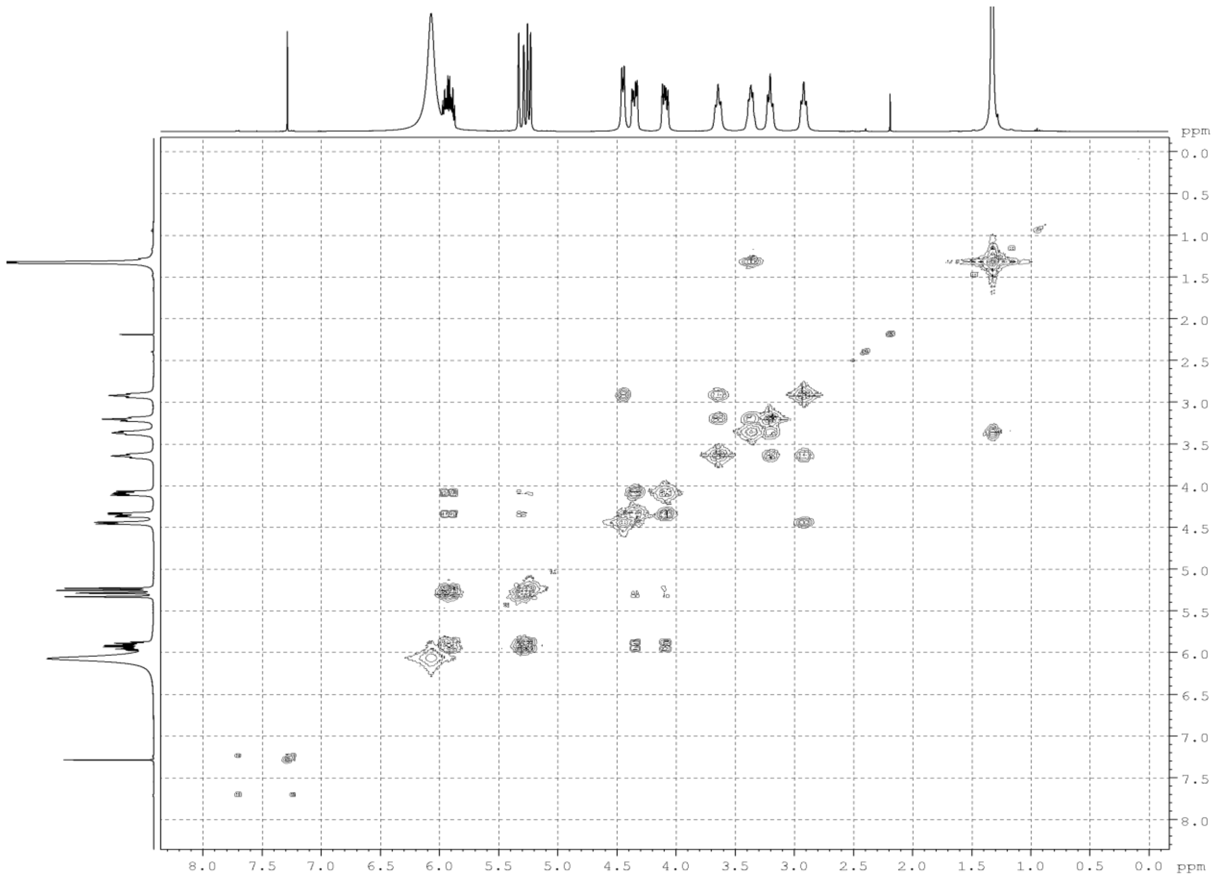

Figure. ^{1}H-^{1}H COSY NMR (400 MHz, $CDCl_3$) spectrum of **44**.

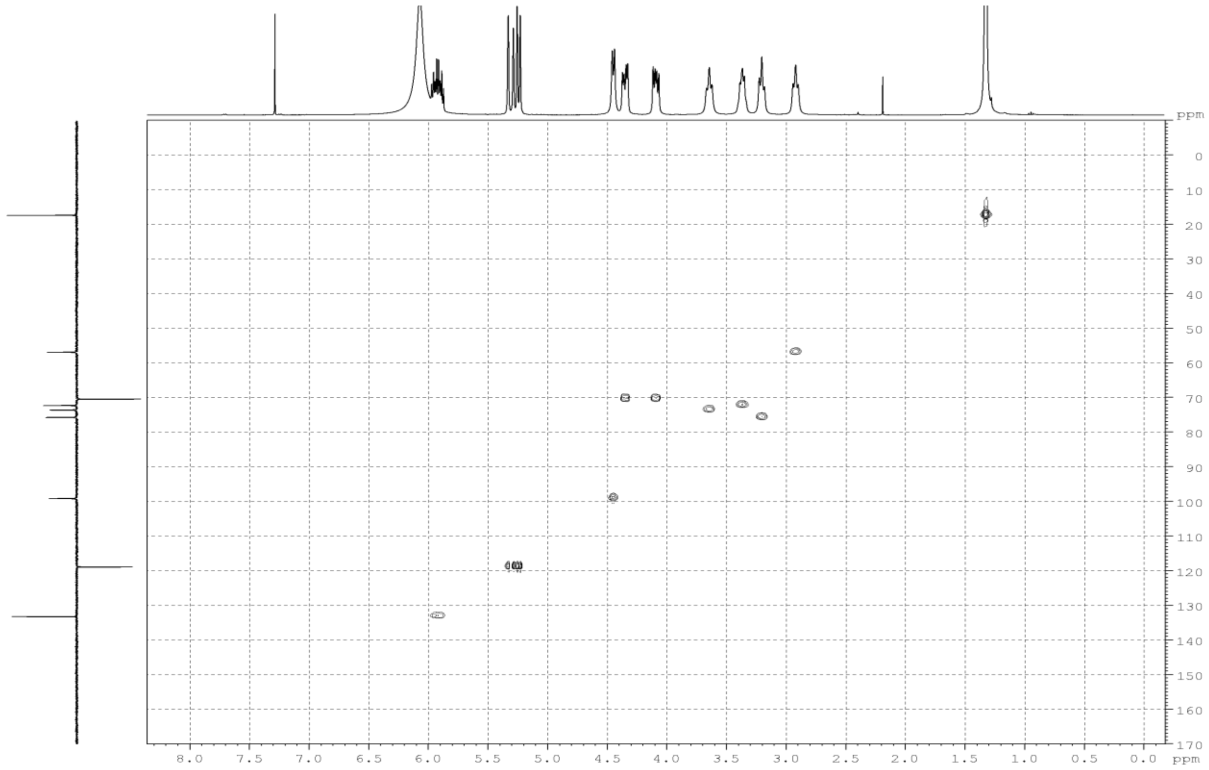

Figure. DEPT-HSQC NMR spectrum of **44**.

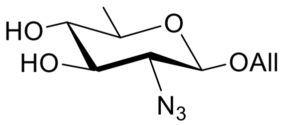

45

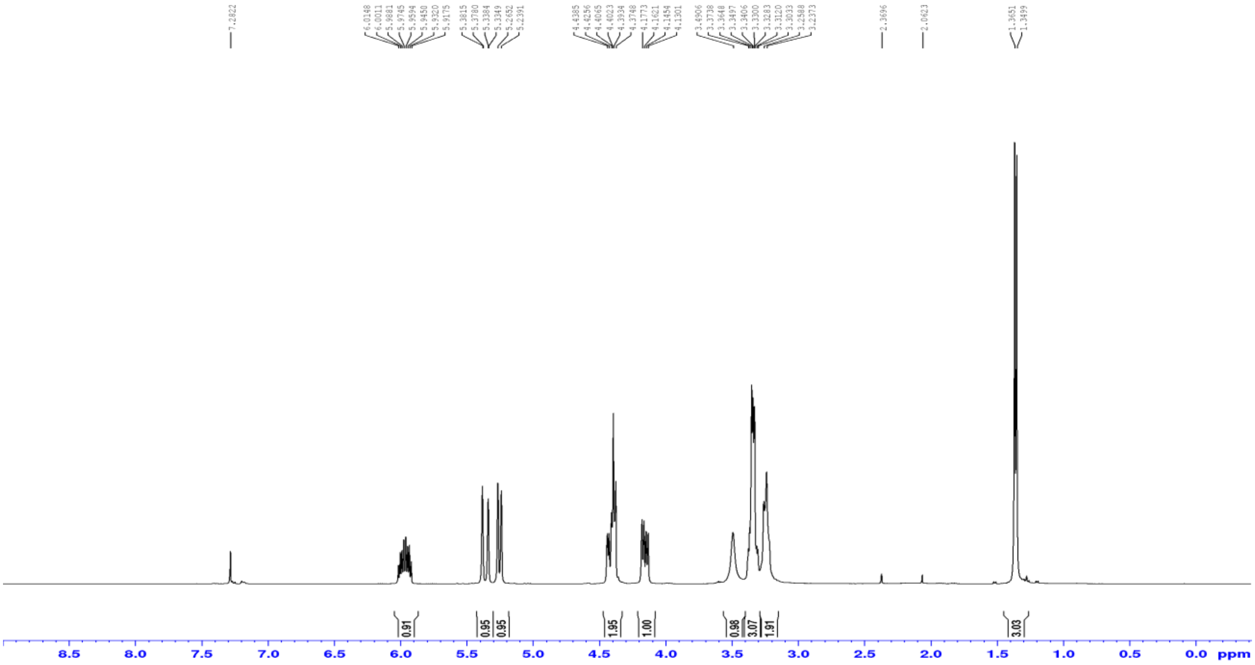

Figure. ^{1}H NMR (400 MHz, CDCl$_3$) spectrum of **45**.

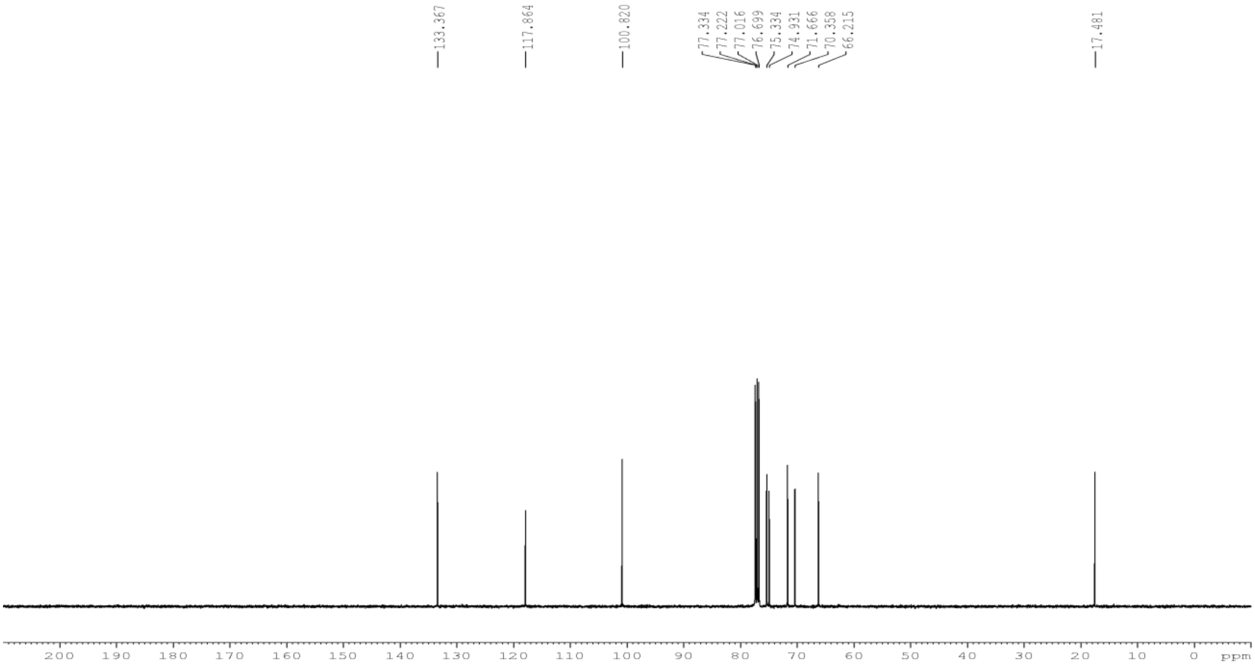

Figure. ^{13}C{^{1}H} NMR (100 MHz, CDCl$_3$) spectrum of **45**.

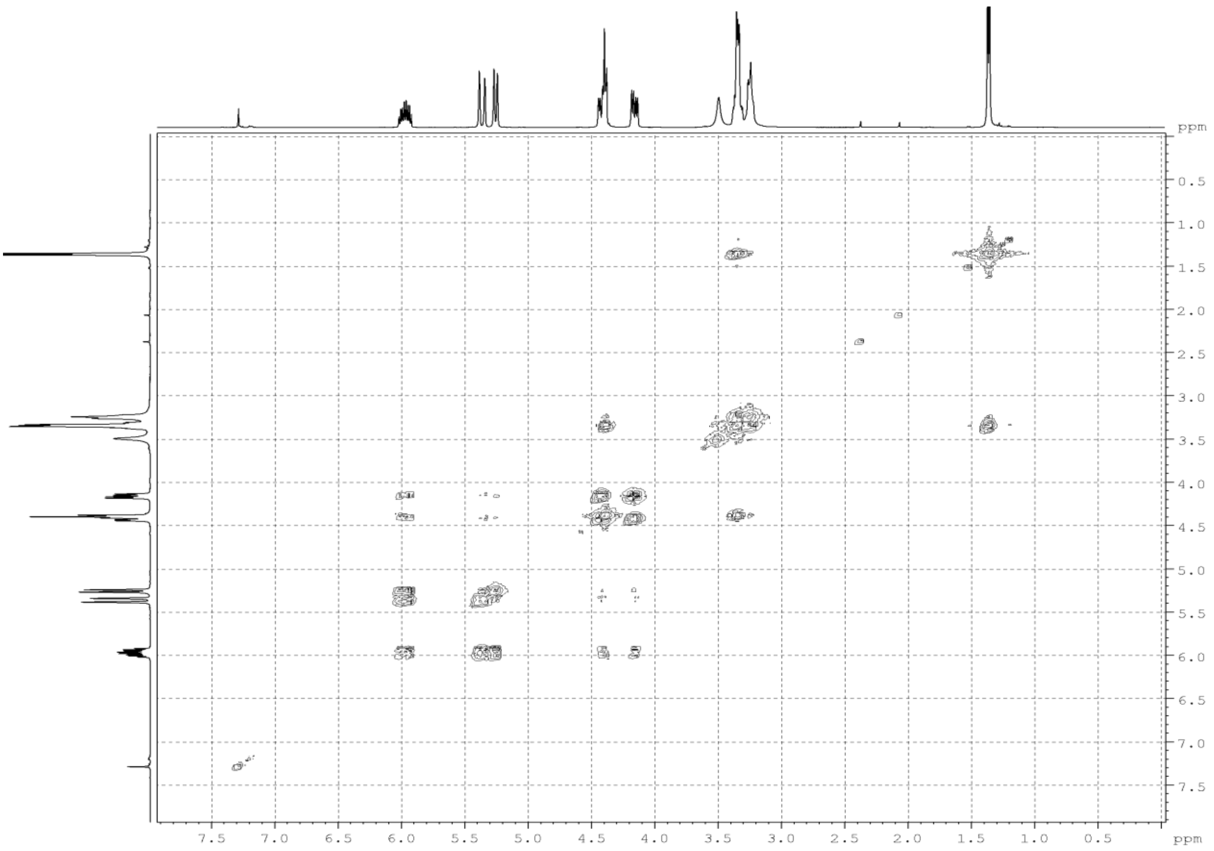

Figure. ^{1}H-^{1}H COSY NMR (400 MHz, $CDCl_3$) spectrum of **45**.

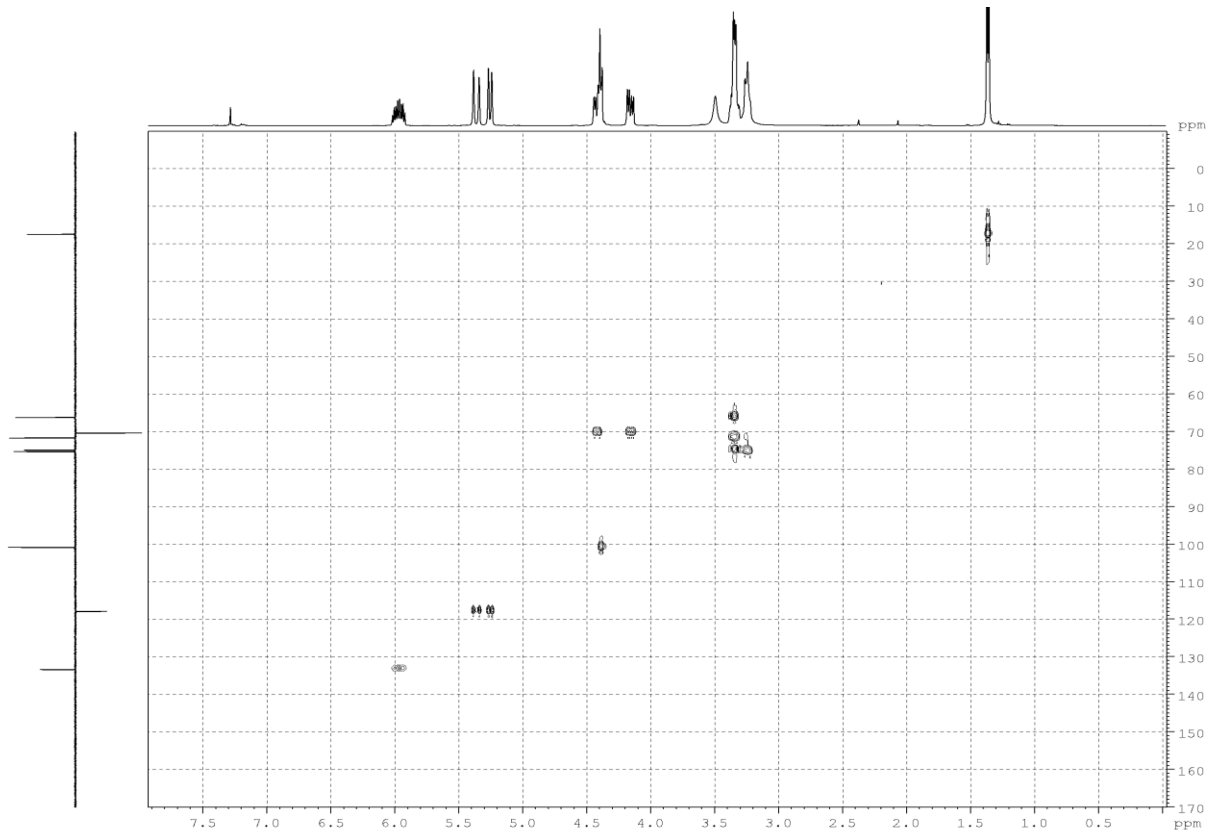

Figure. DEPT-HSQC NMR spectrum of **45**.

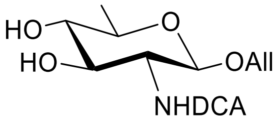

46

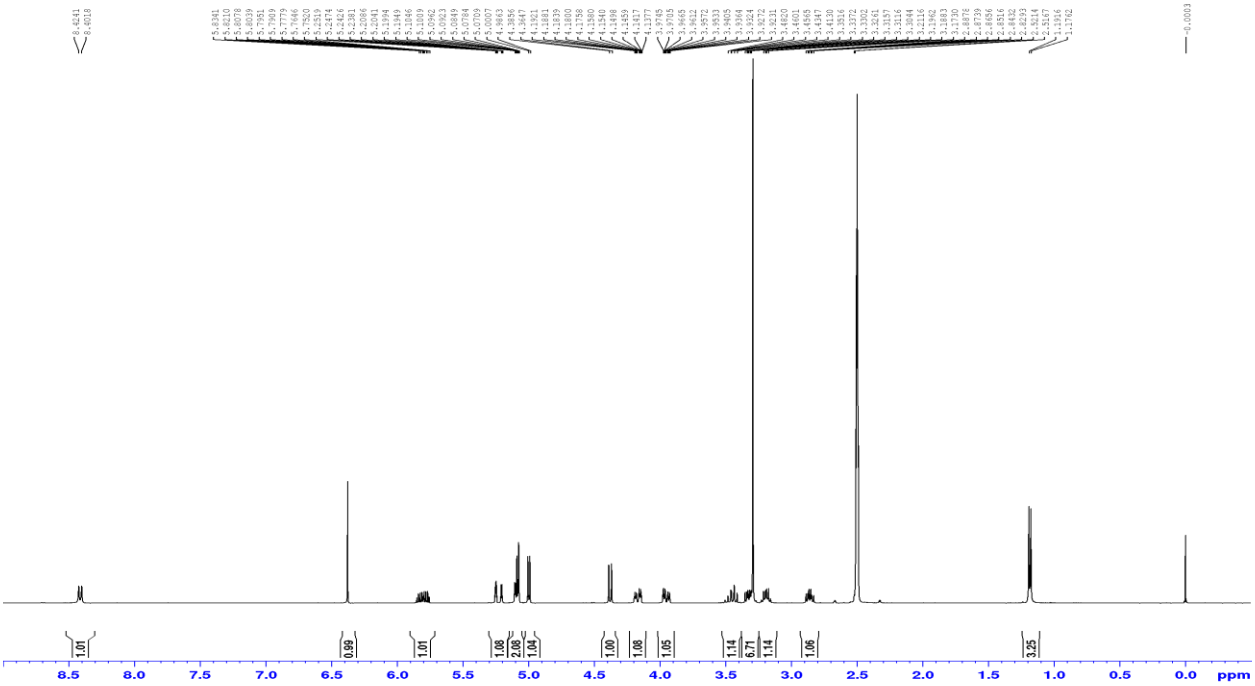

Figure. ^{1}H NMR (400 MHz, DMSO-d_6) spectrum of **46**.

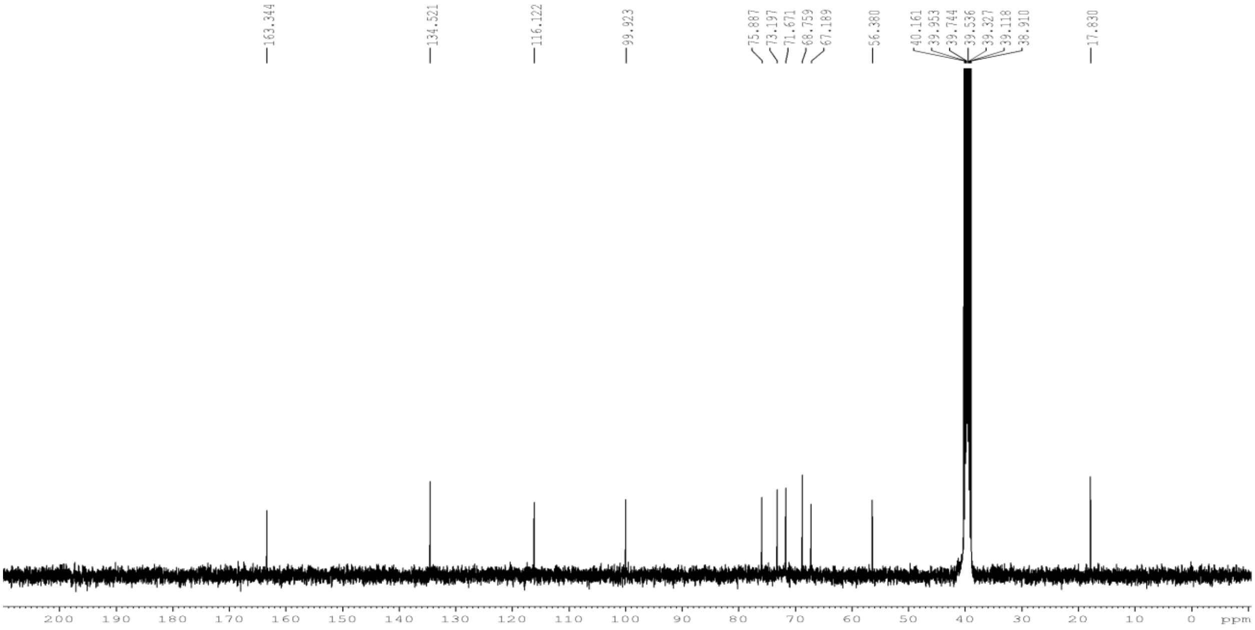

Figure. ^{13}C{^{1}H} NMR (100 MHz, DMSO-d_6) spectrum of **46**.

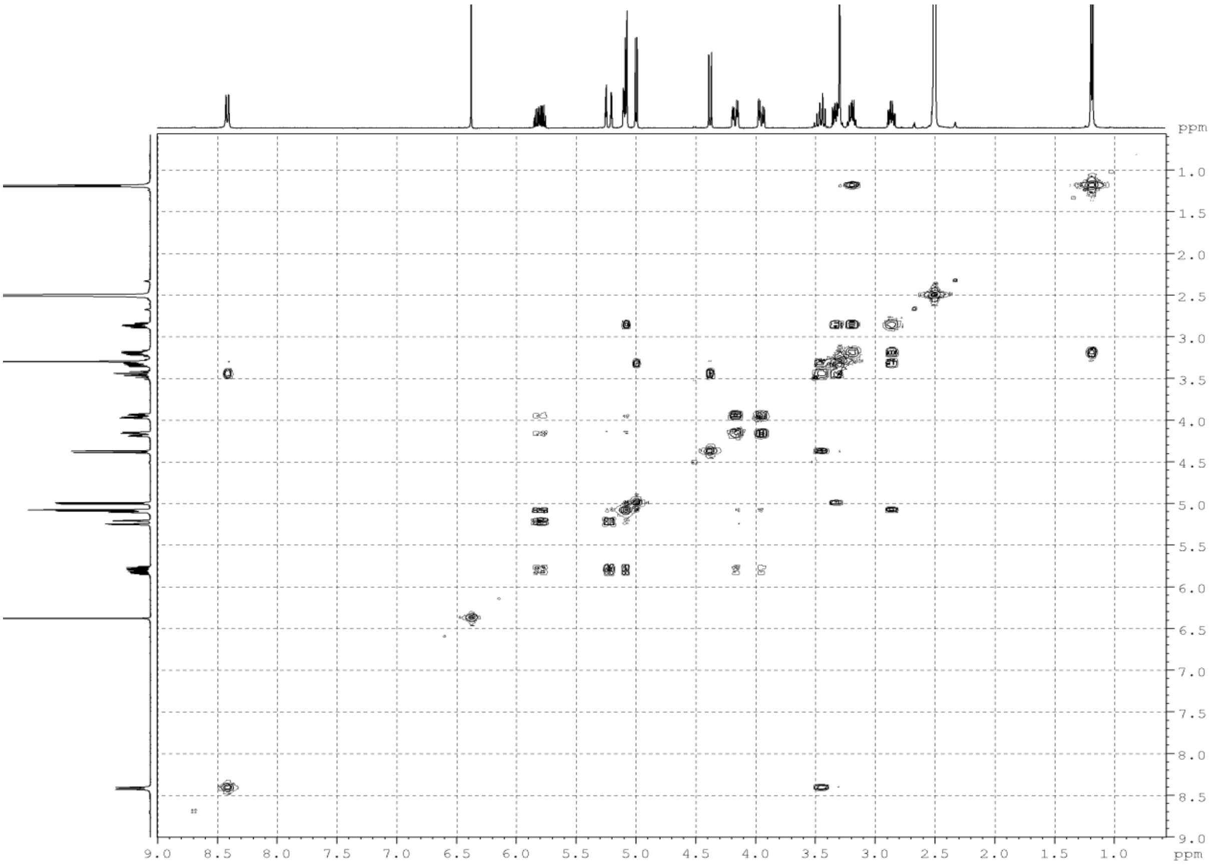

Figure. ^{1}H-^{1}H COSY NMR (400 MHz, DMSO-d_6) spectrum of **46**.

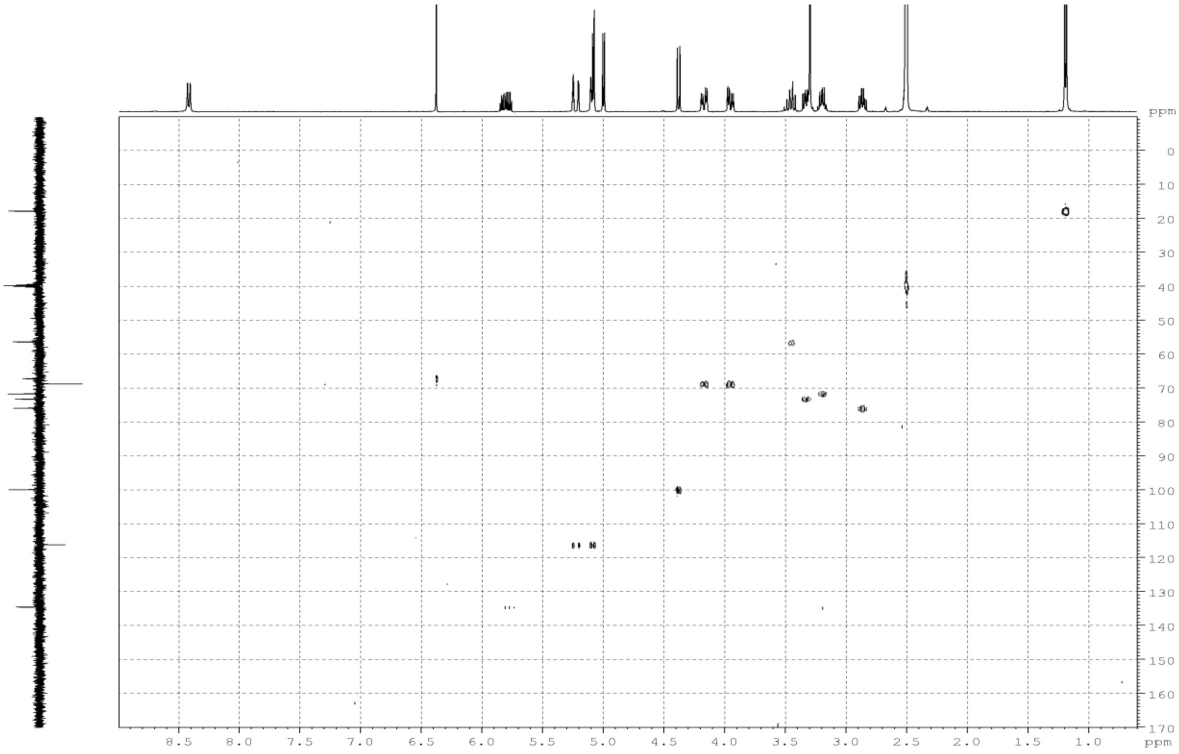

Figure. DEPT-HSQC NMR spectrum of **46**.

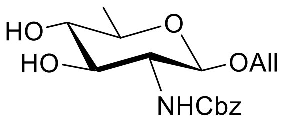

48

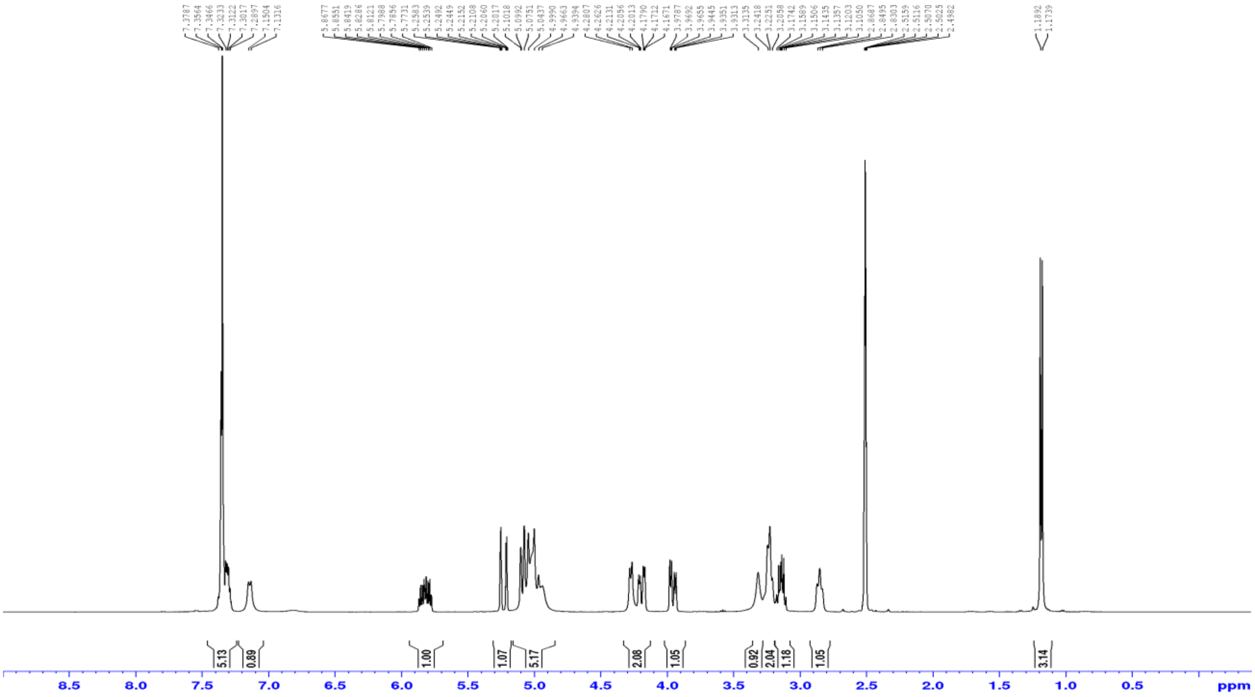

Figure. ^{1}H NMR (400 MHz, DMSO-d_6) spectrum of **48**.

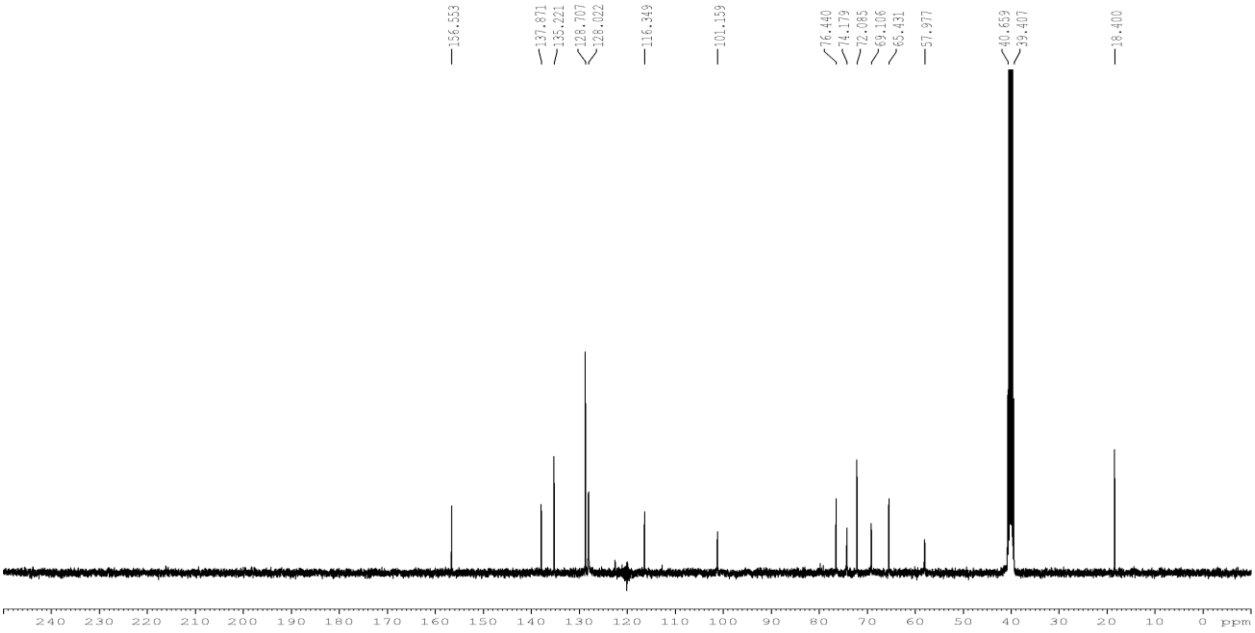

Figure. ^{13}C{^{1}H} NMR (100 MHz, DMSO-d_6) spectrum of **48**.

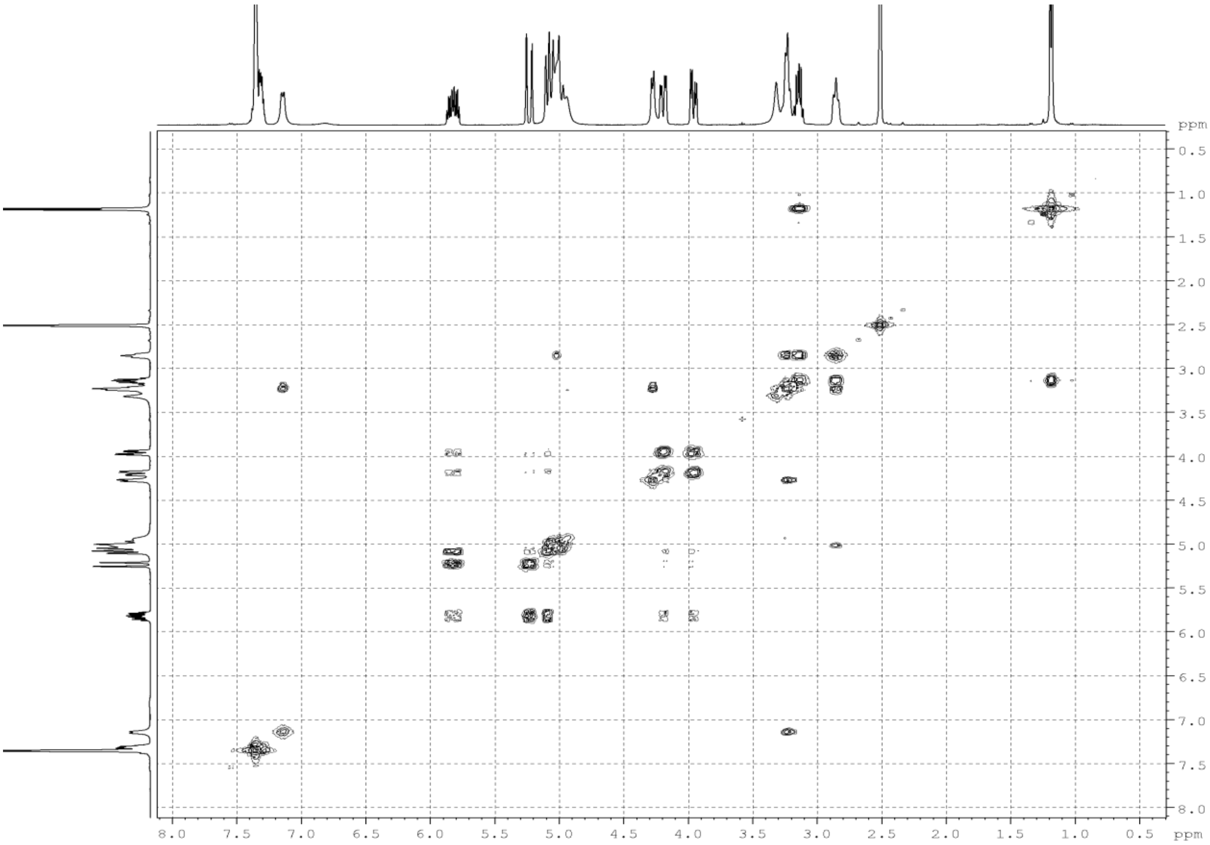

Figure. ^{1}H-^{1}H COSY NMR (400 MHz, DMSO-d_6) spectrum of **48**.

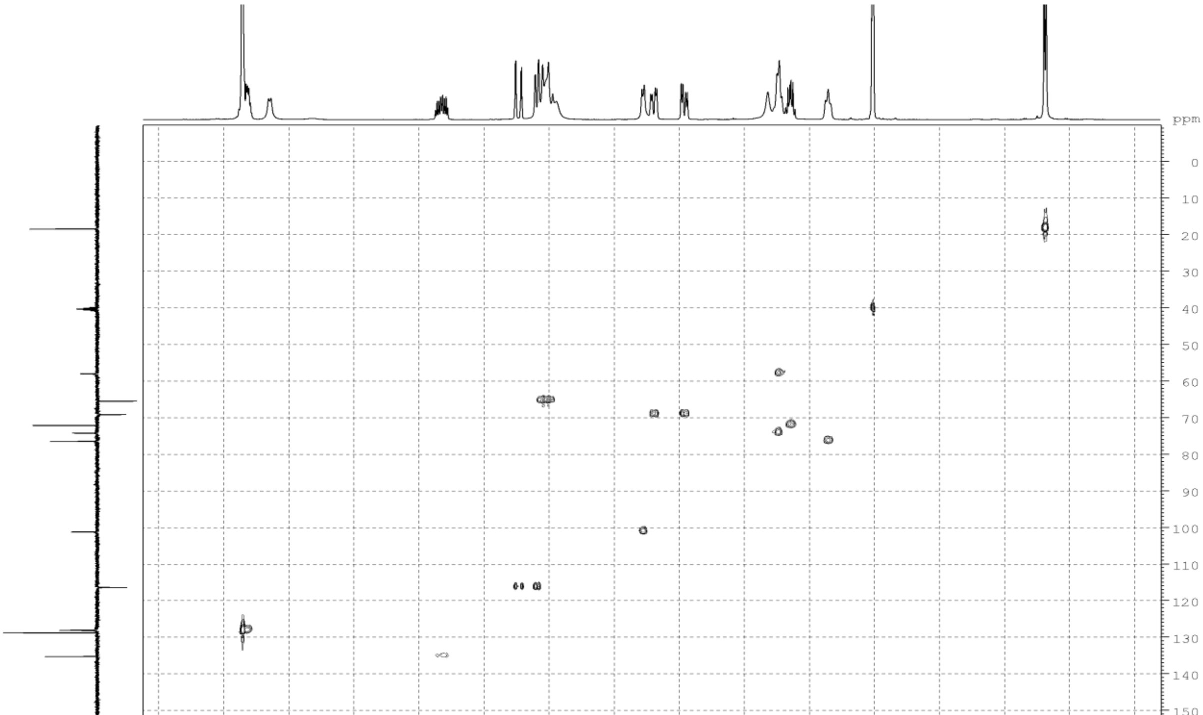

Figure. DEPT-HSQC NMR spectrum of **48**.

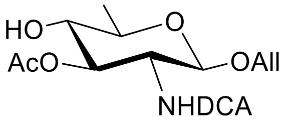

49

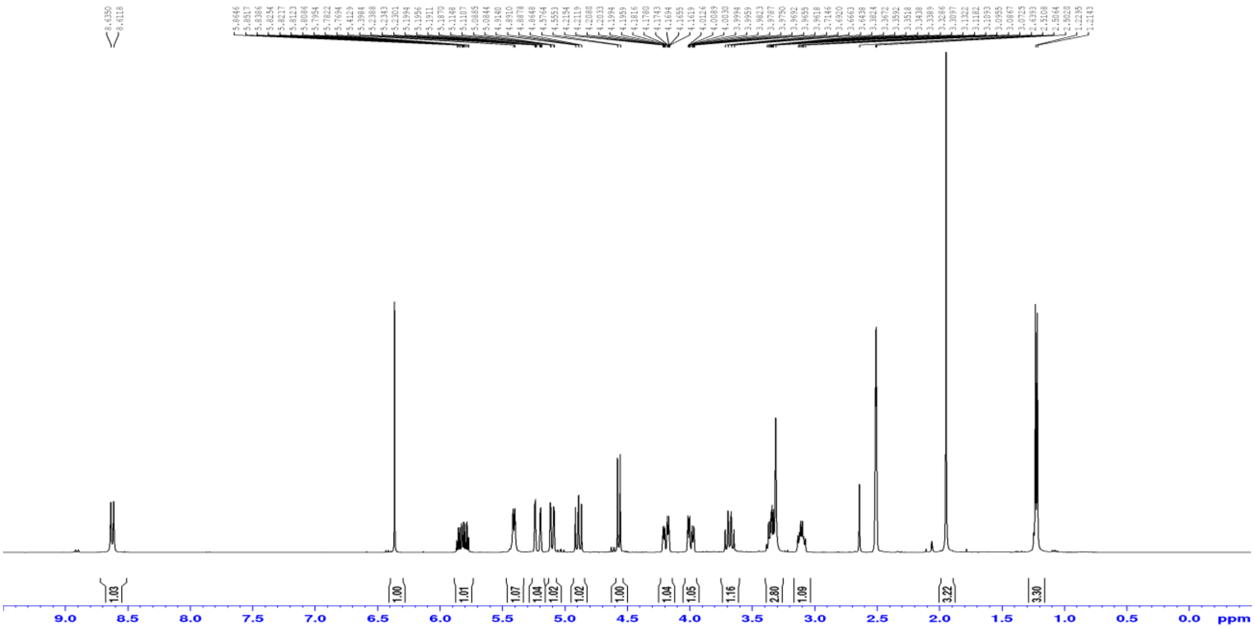

Figure. ^{1}H NMR (400 MHz, DMSO-d_6) spectrum of **49**.

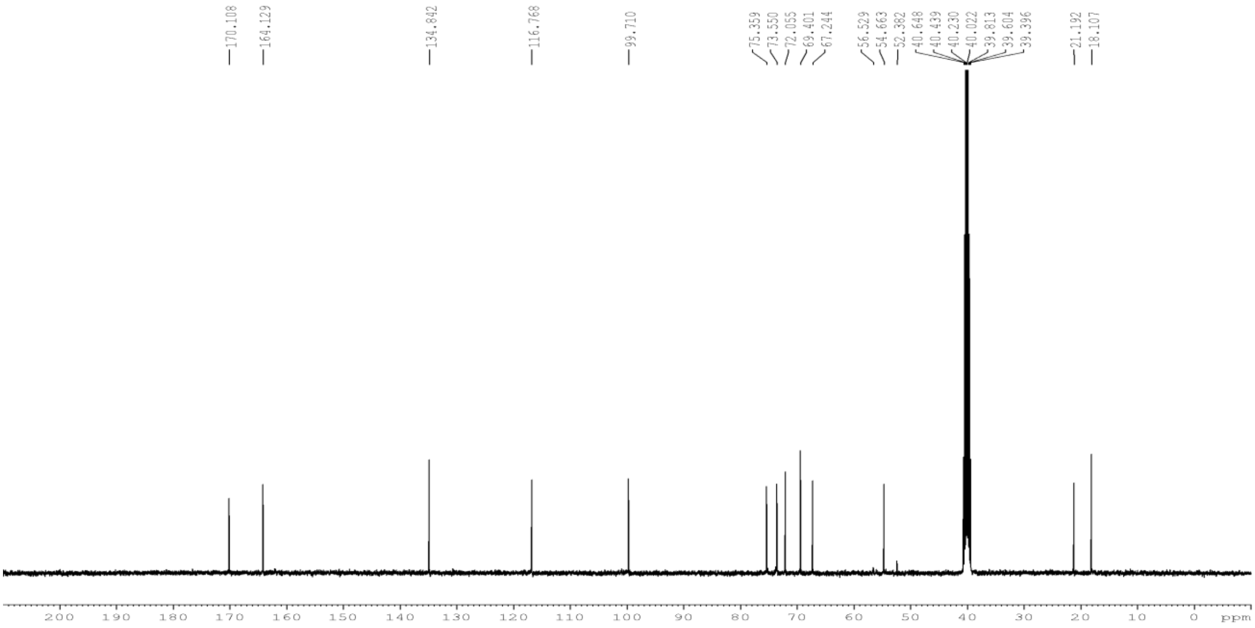

Figure. $^{13}C\{^{1}H\}$ NMR (100 MHz, DMSO-d_6) spectrum of **49**.

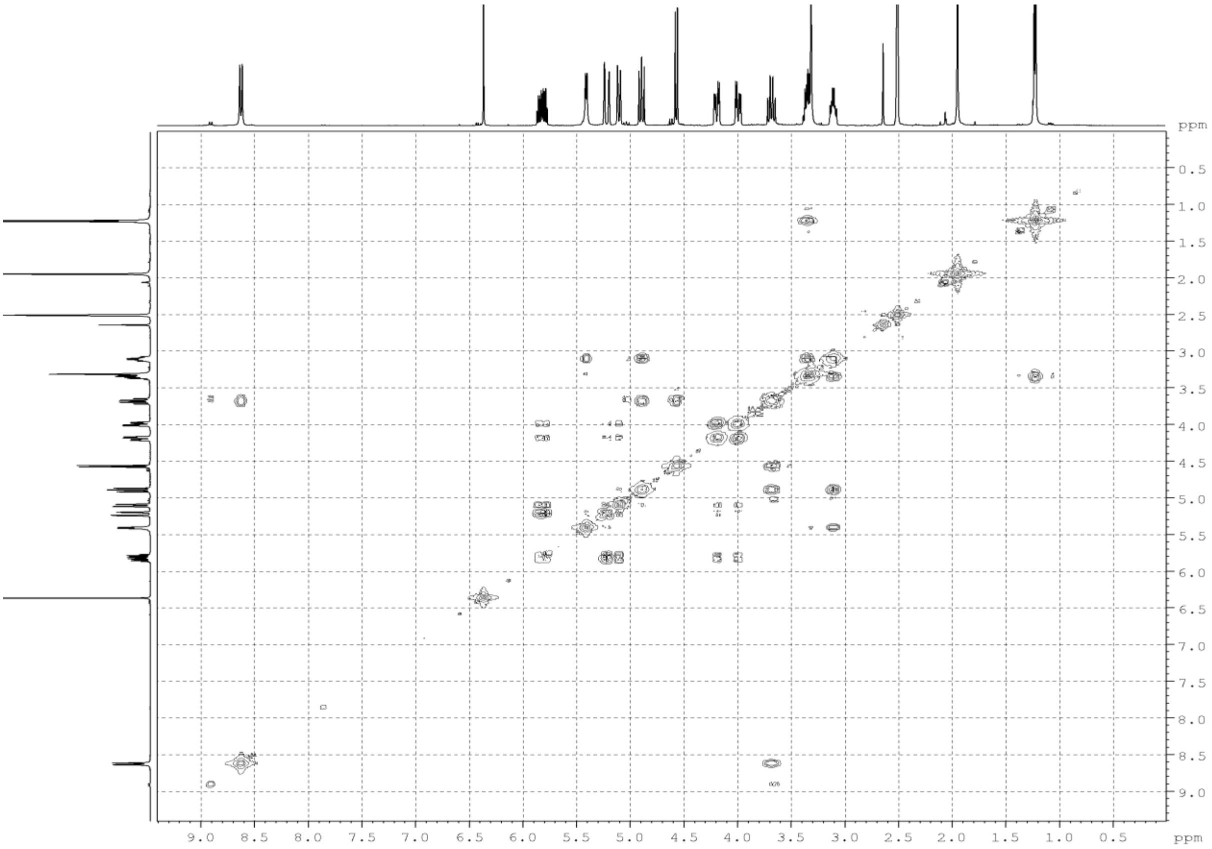

Figure. ¹H-¹H COSY NMR (400 MHz, DMSO-d_6) spectrum of **49**.

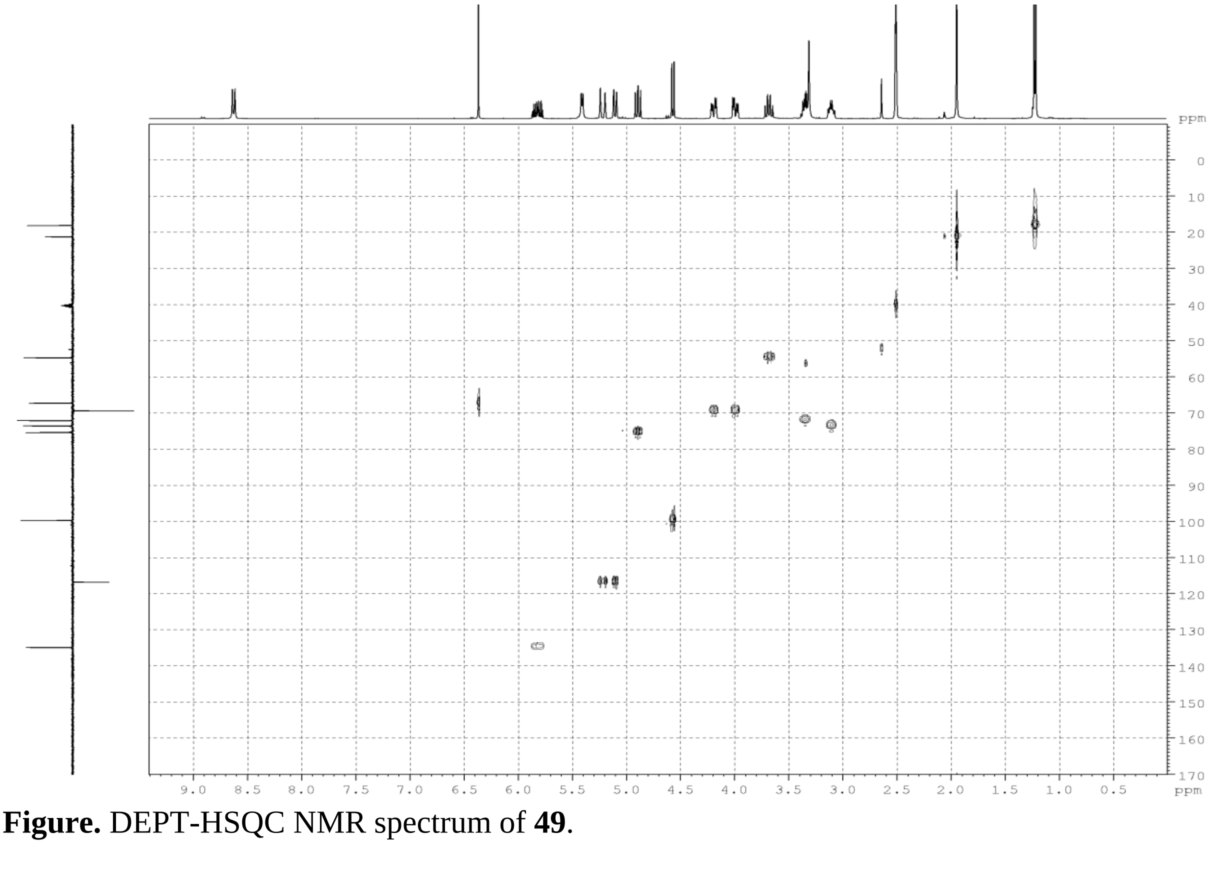

Figure. DEPT-HSQC NMR spectrum of **49**.

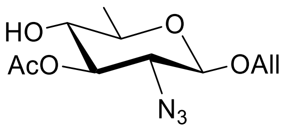

51

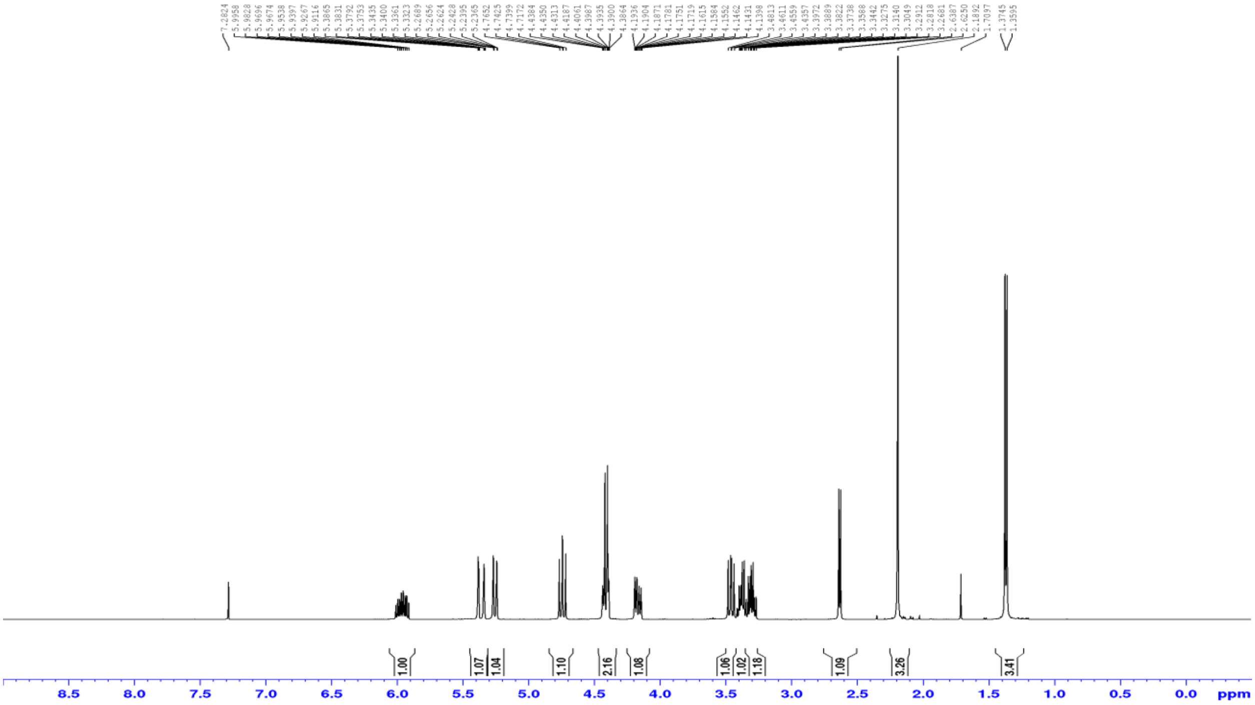

Figure. ^{1}H NMR (400 MHz, $CDCl_3$) spectrum of **51**.

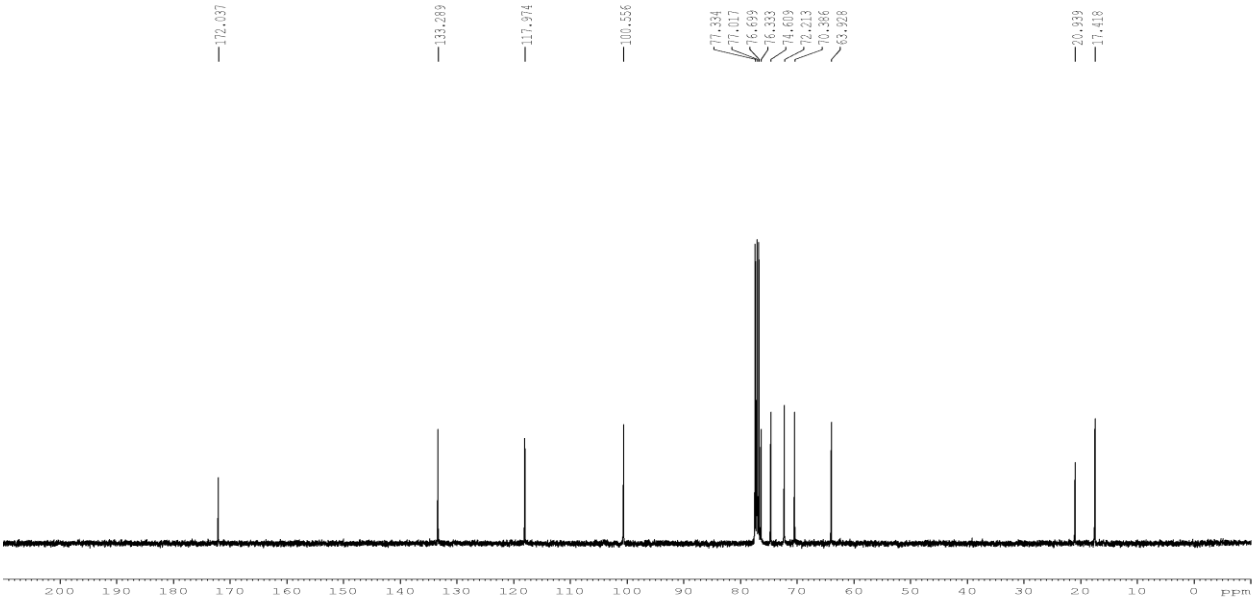

Figure. $^{13}C\{^{1}H\}$ NMR (100 MHz, $CDCl_3$) spectrum of **51**.

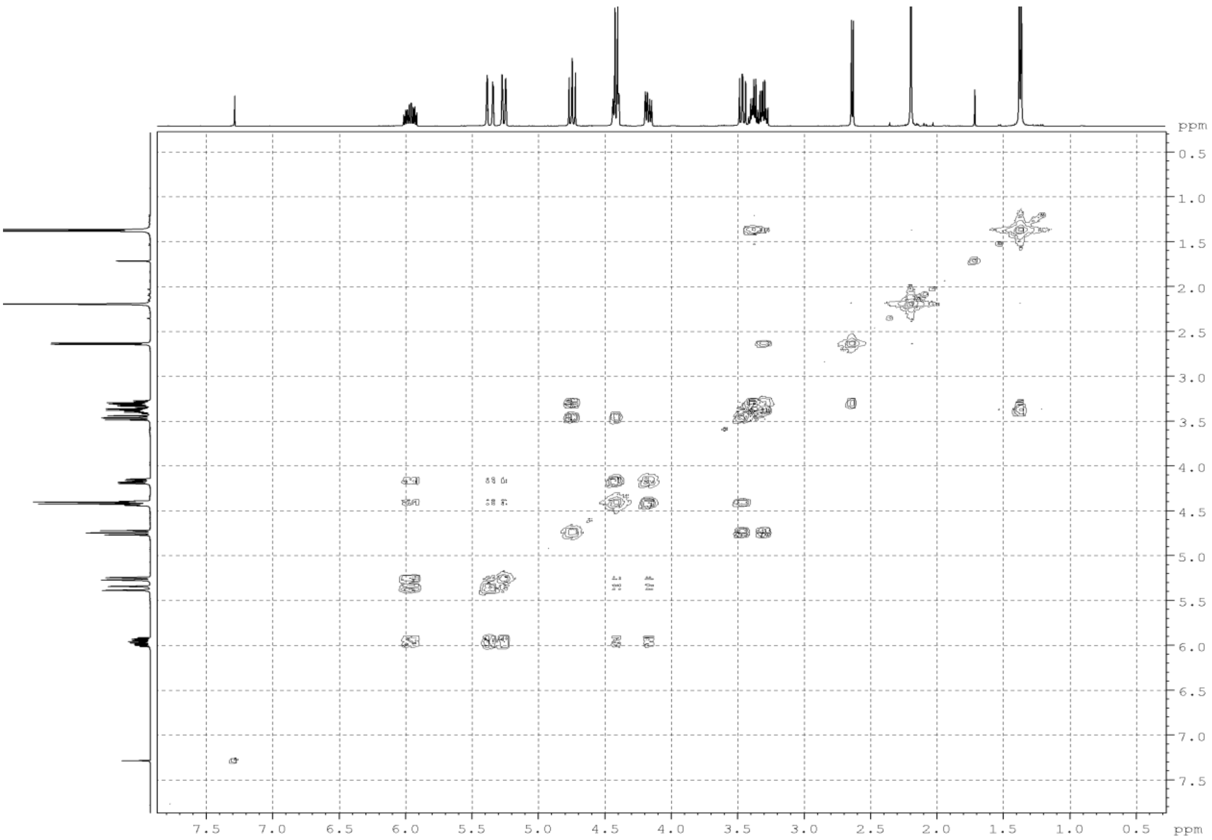

Figure. ^{1}H-^{1}H COSY NMR (400 MHz, $CDCl_3$) spectrum of **51**.

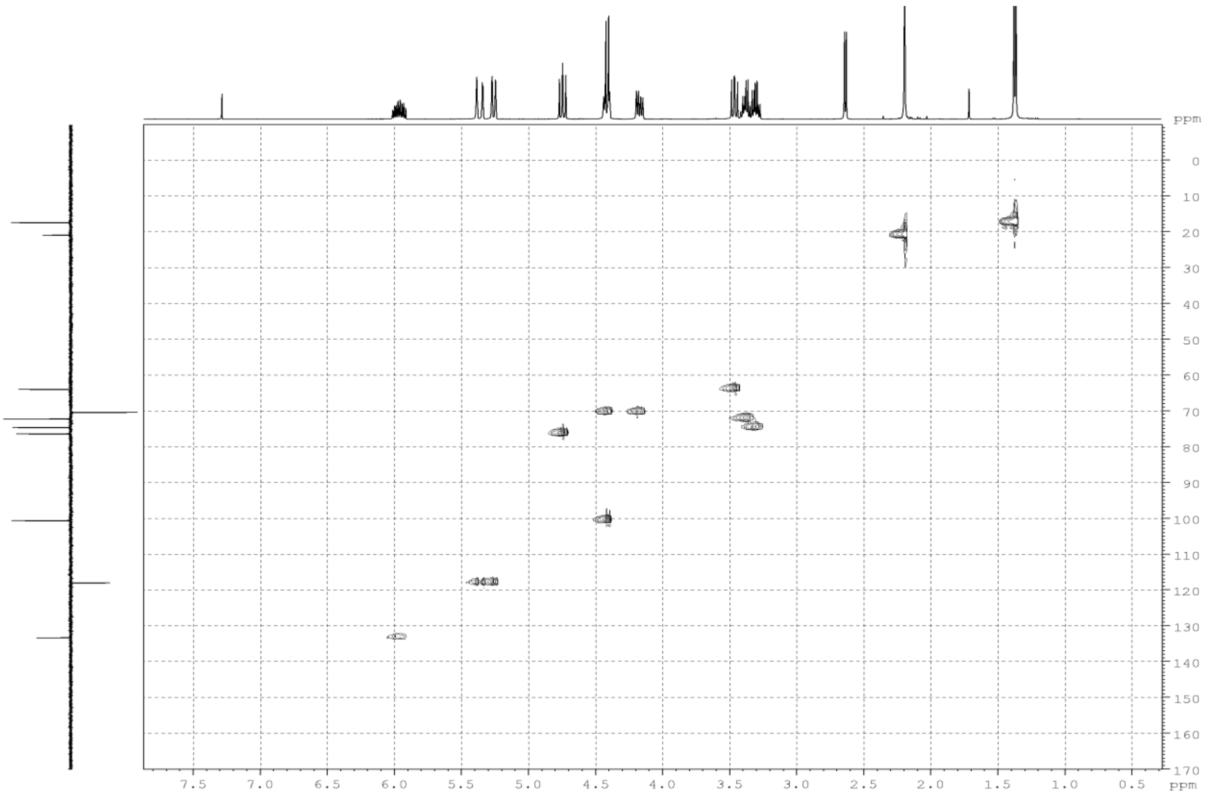

Figure. DEPT-HSQC NMR spectrum of **51**.

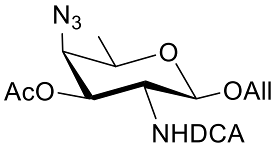

52

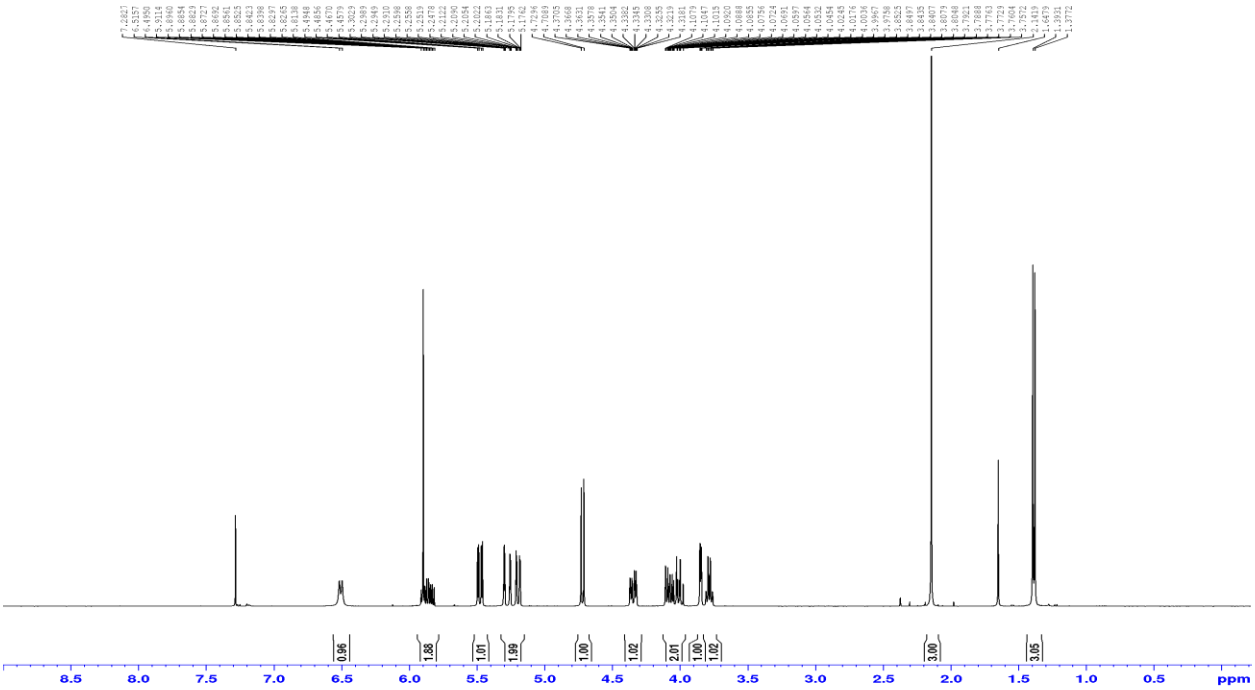

Figure. ^{1}H NMR (400 MHz, $CDCl_3$) spectrum of **52**.

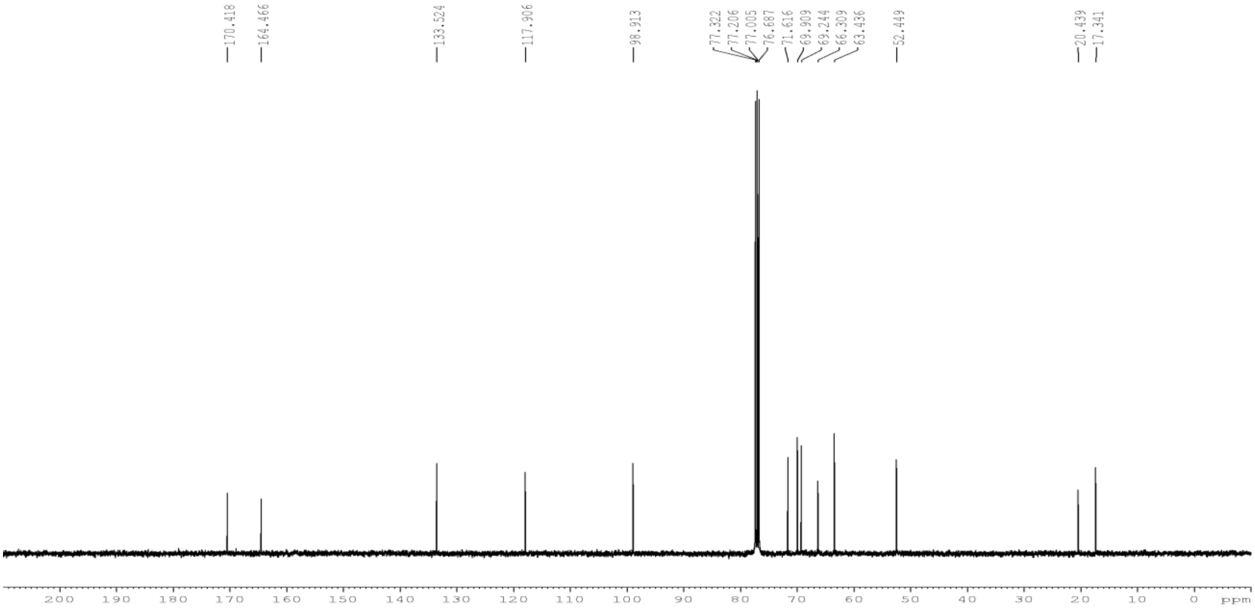

Figure. $^{13}C\{^{1}H\}$ NMR (100 MHz, $CDCl_3$) spectrum of **52**.

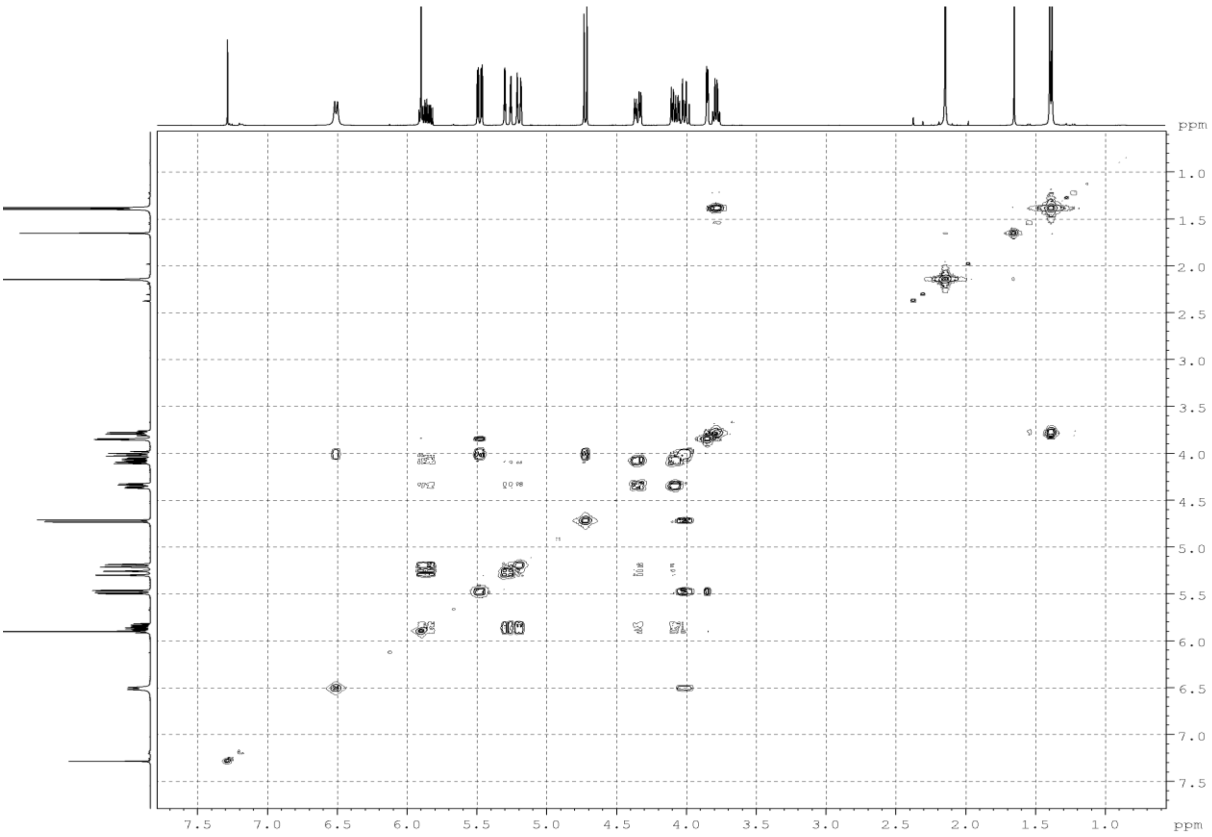

Figure. ^{1}H-^{1}H COSY NMR (400 MHz, $CDCl_3$) spectrum of **52**.

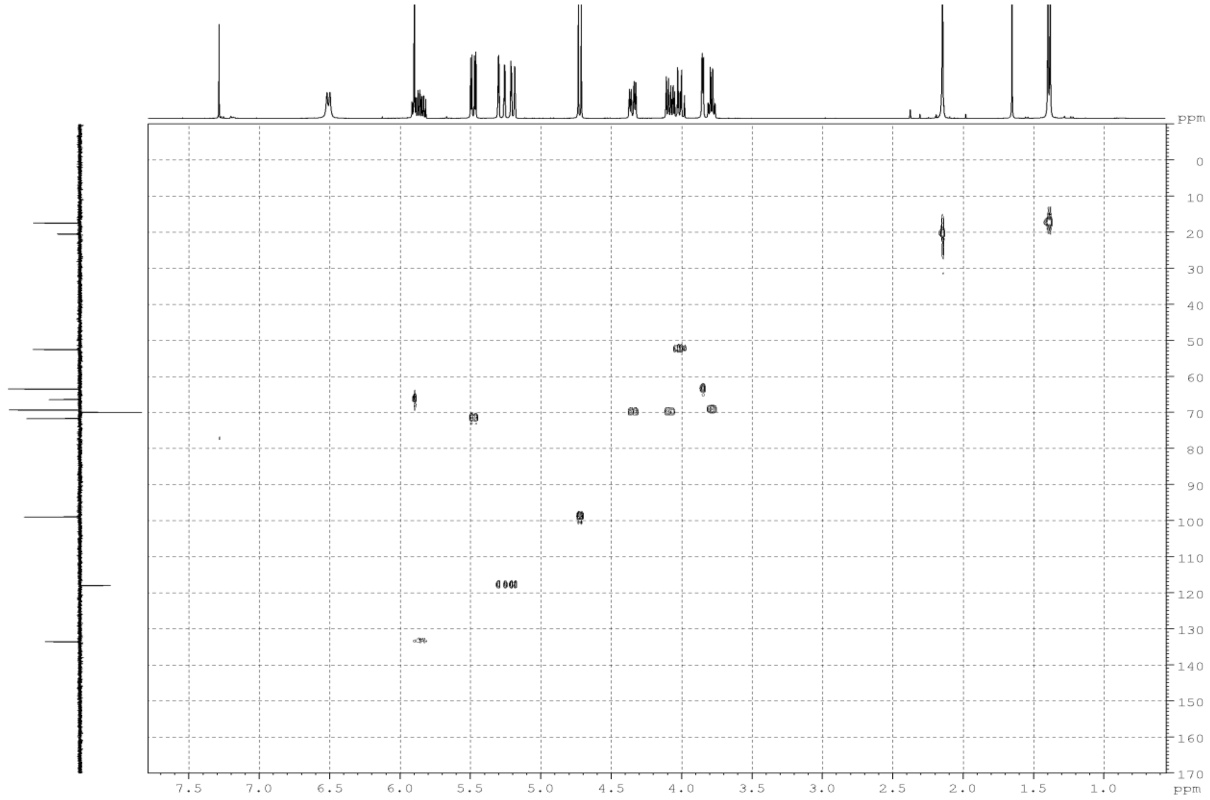

Figure. DEPT-HSQC NMR spectrum of **52**.

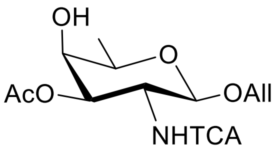

54

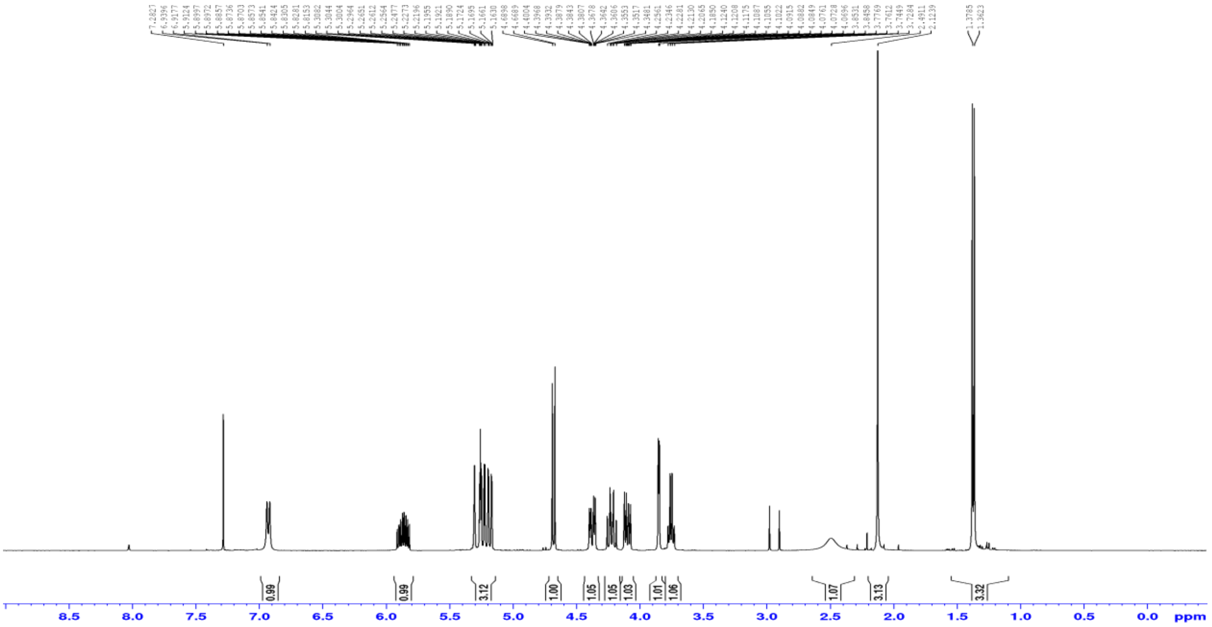

Figure. ^{1}H NMR (400 MHz, $CDCl_3$) spectrum of **54**.

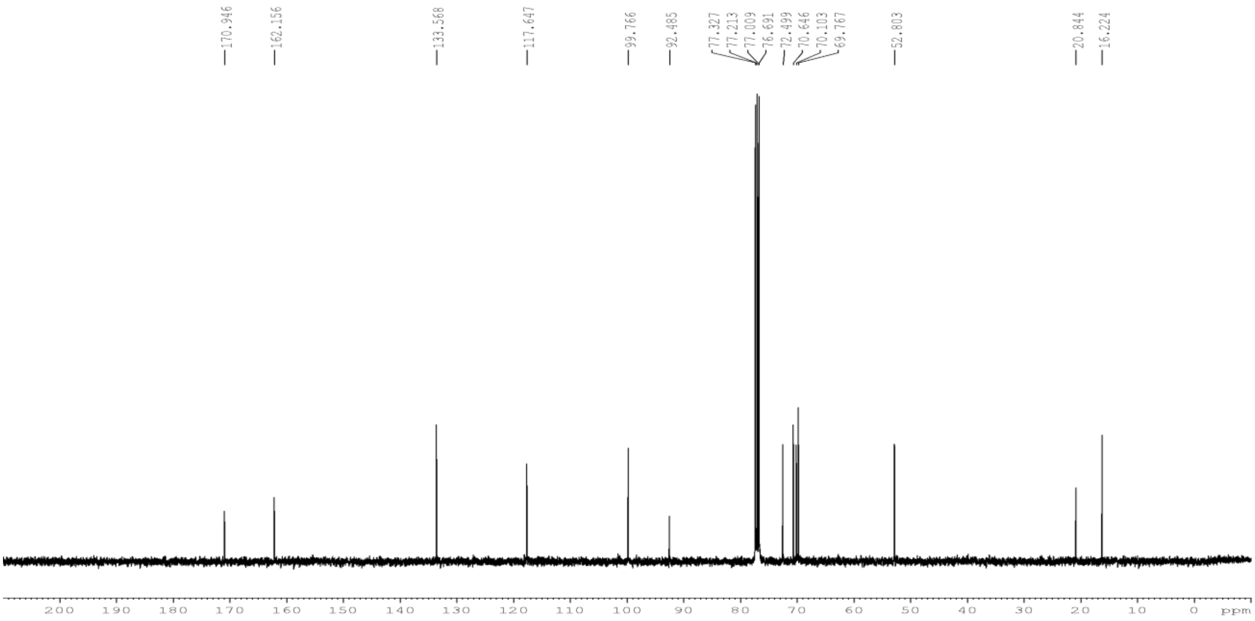

Figure. $^{13}C\{^{1}H\}$ NMR (100 MHz, $CDCl_3$) spectrum of **54**.

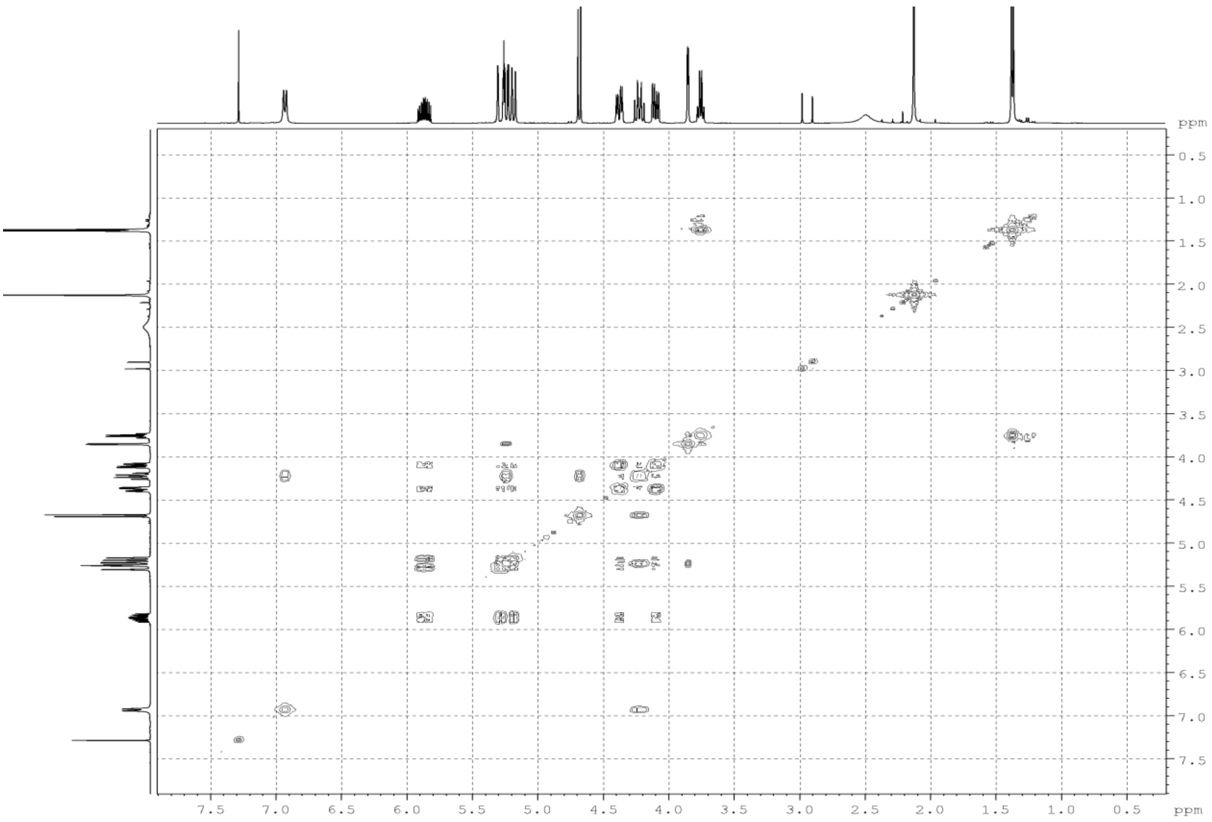

Figure. ^1H-^1H COSY NMR (400 MHz, $CDCl_3$) spectrum of **54**.

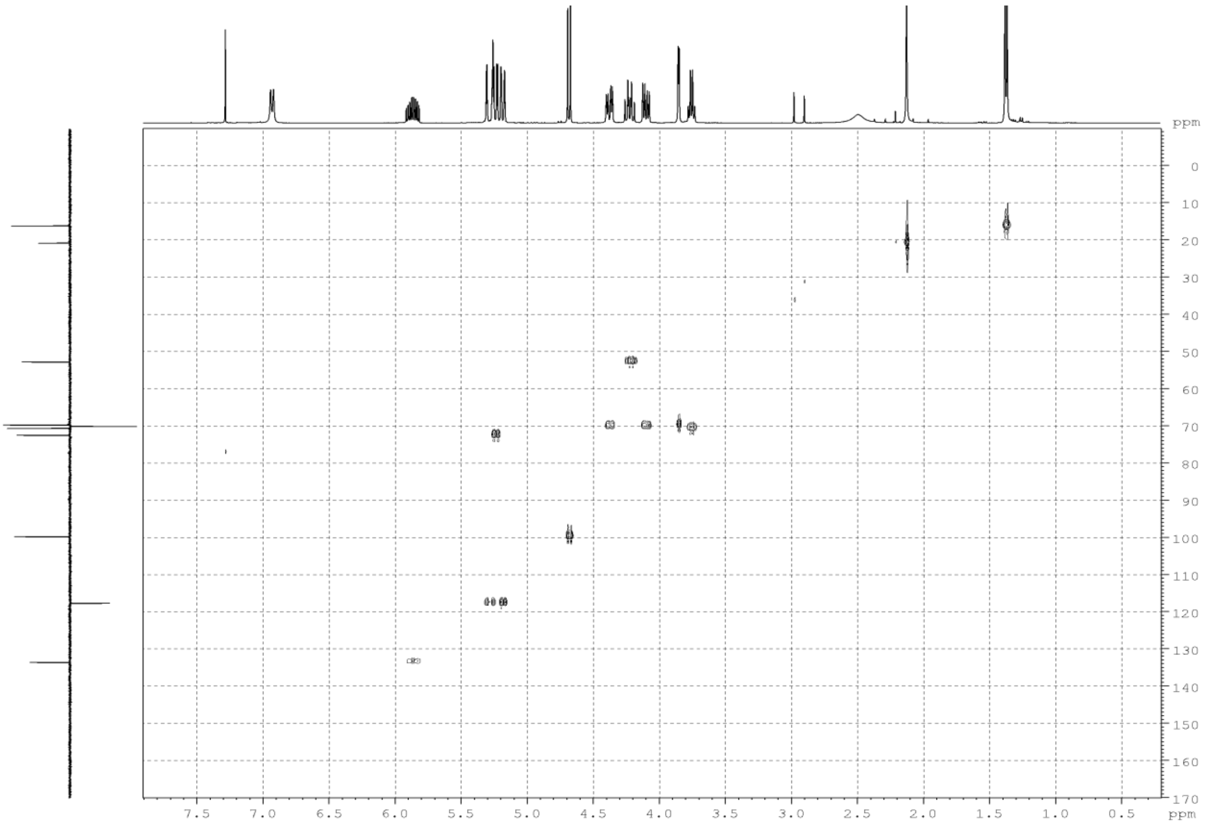

Figure. DEPT-HSQC NMR spectrum of **54**.

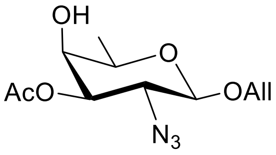

55

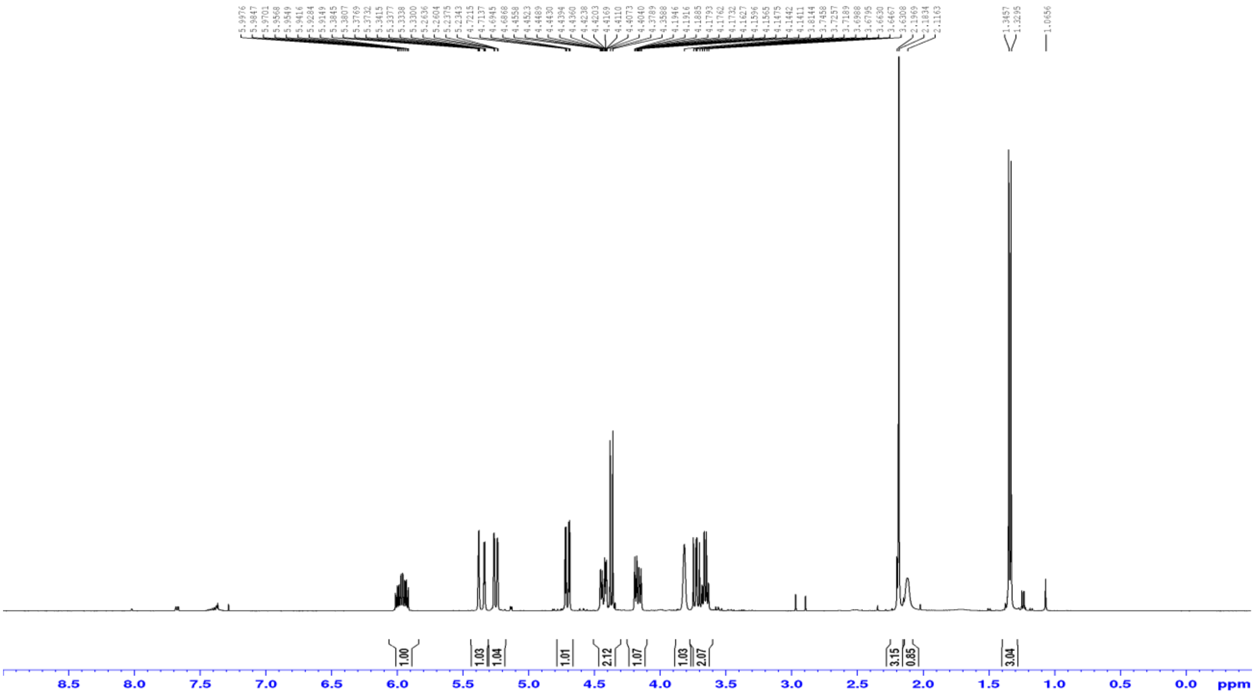

Figure. ^{1}H NMR (400 MHz, $CDCl_3$) spectrum of **55**.

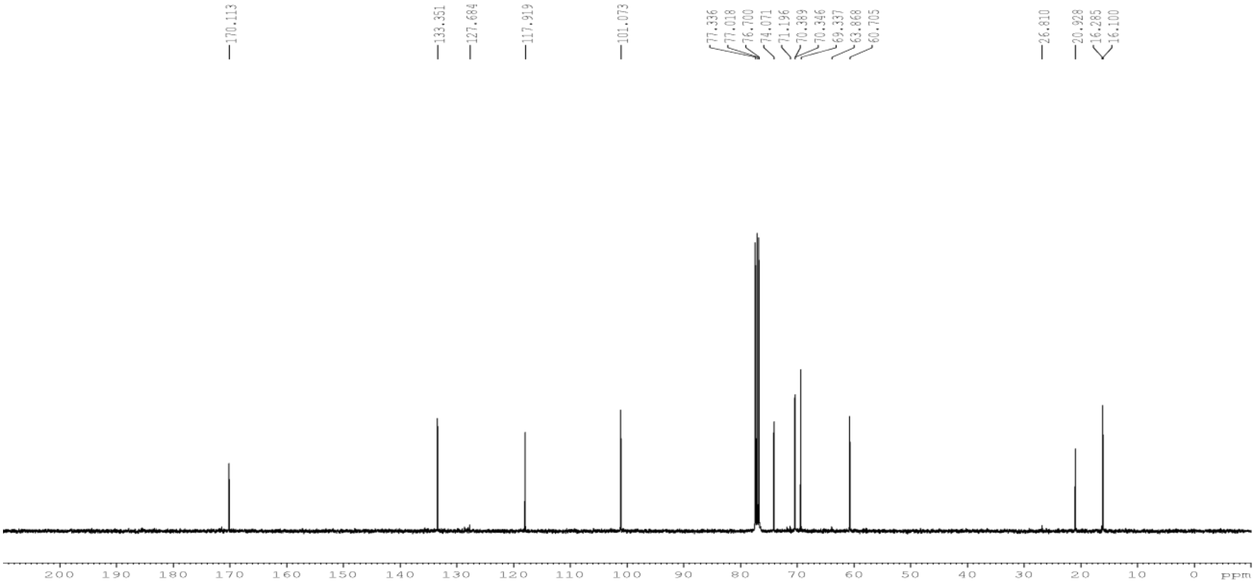

Figure. $^{13}C\{^{1}H\}$ NMR (100 MHz, $CDCl_3$) spectrum of **55**.

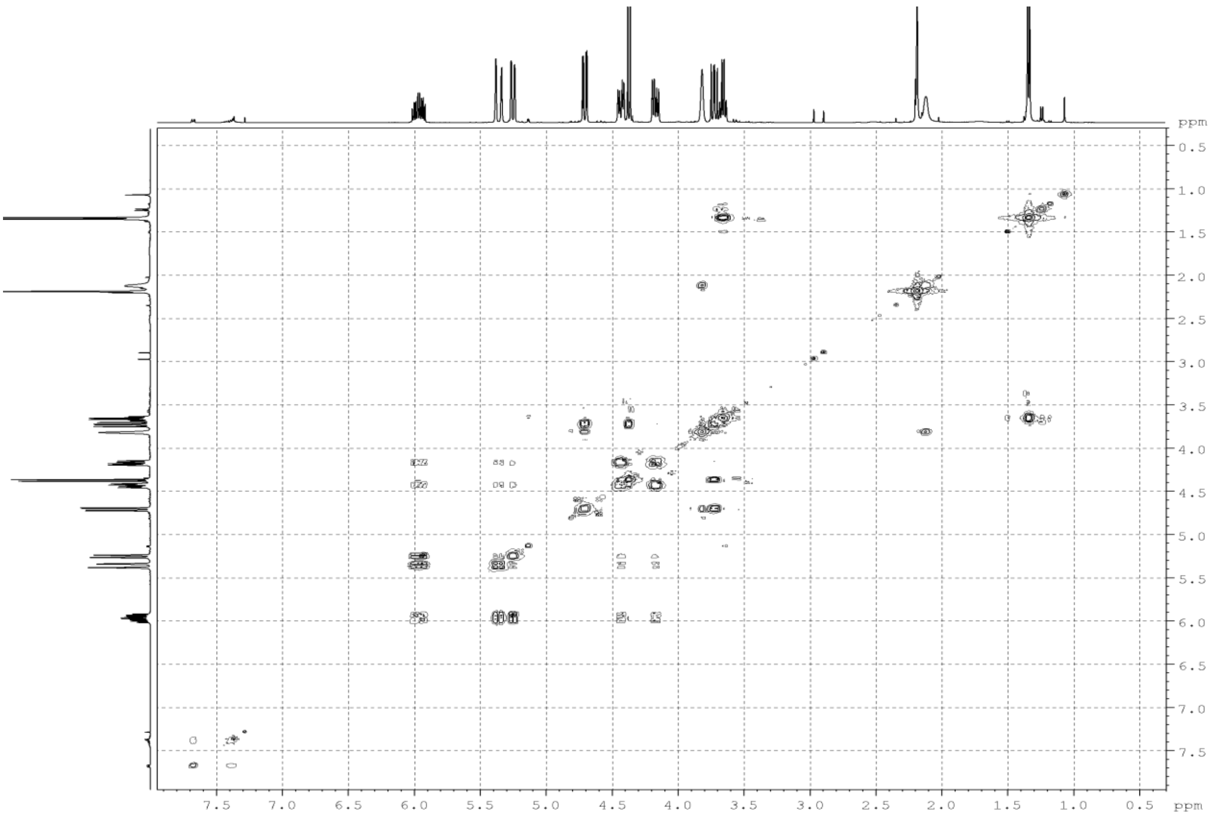

Figure. ^{1}H-^{1}H COSY NMR (400 MHz, $CDCl_3$) spectrum of **55**.

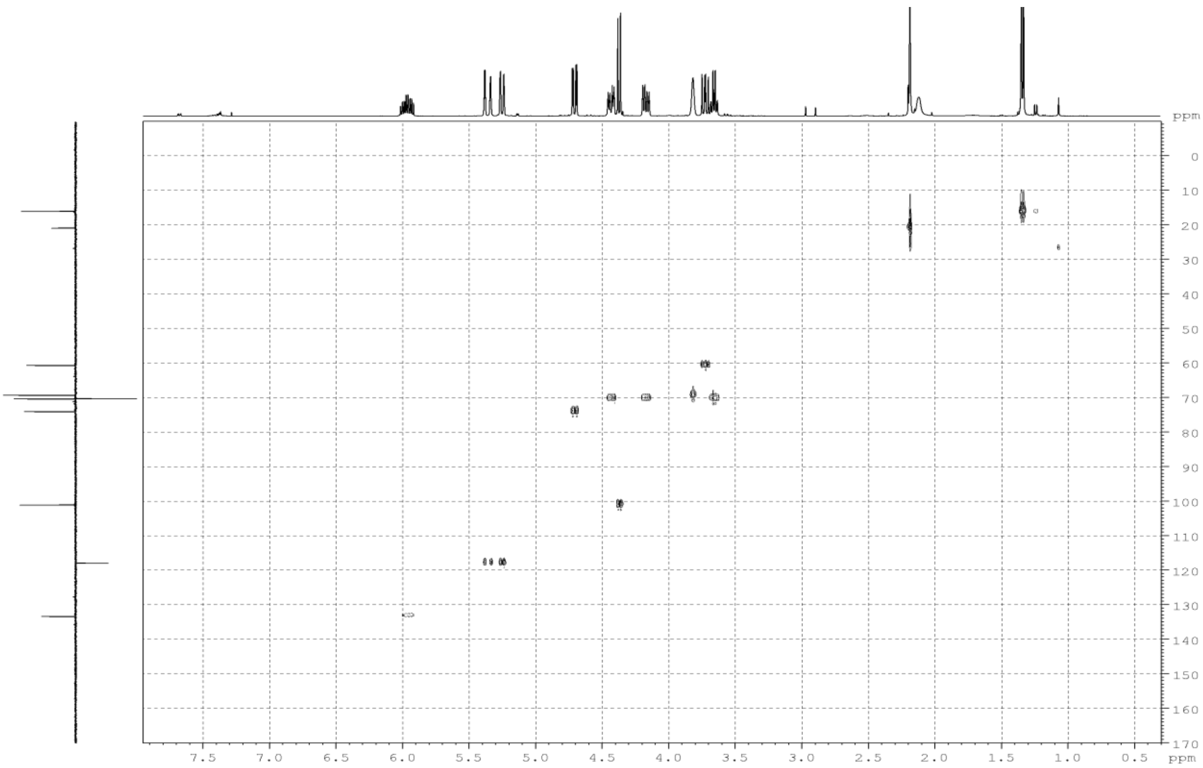

Figure. DEPT-HSQC NMR spectrum of **55**.

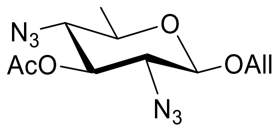

56

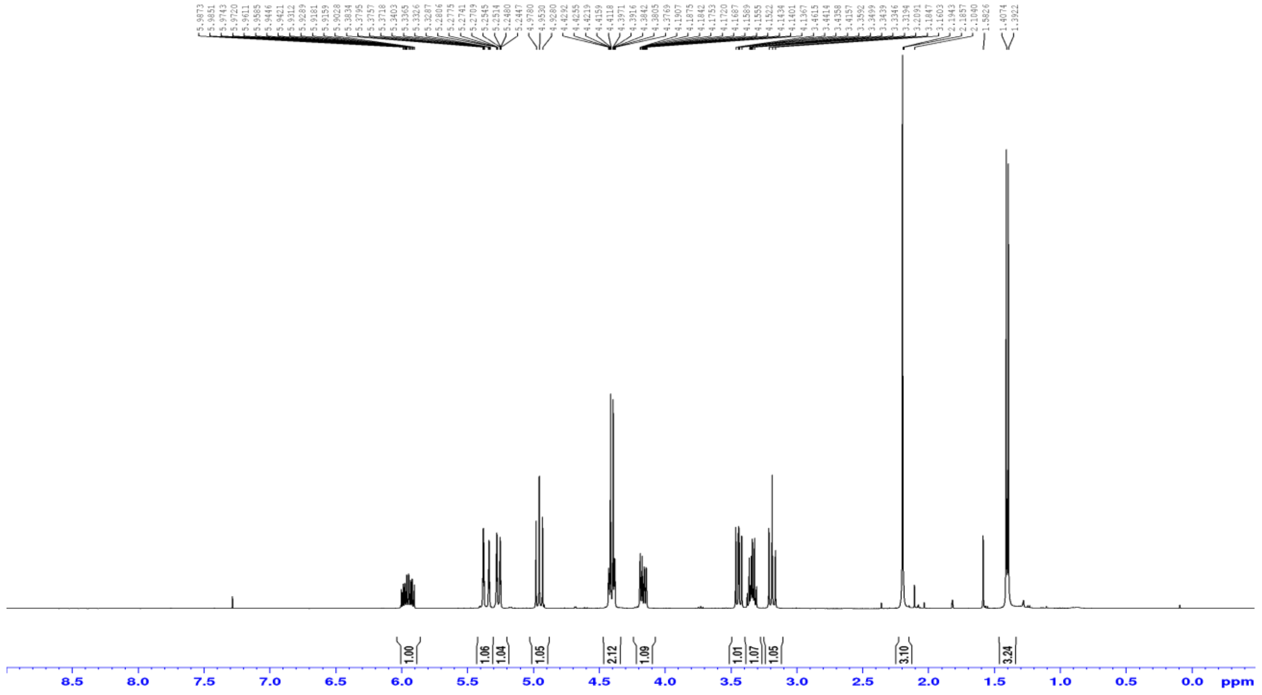

Figure. ^{1}H NMR (400 MHz, $CDCl_3$) spectrum of **56**.

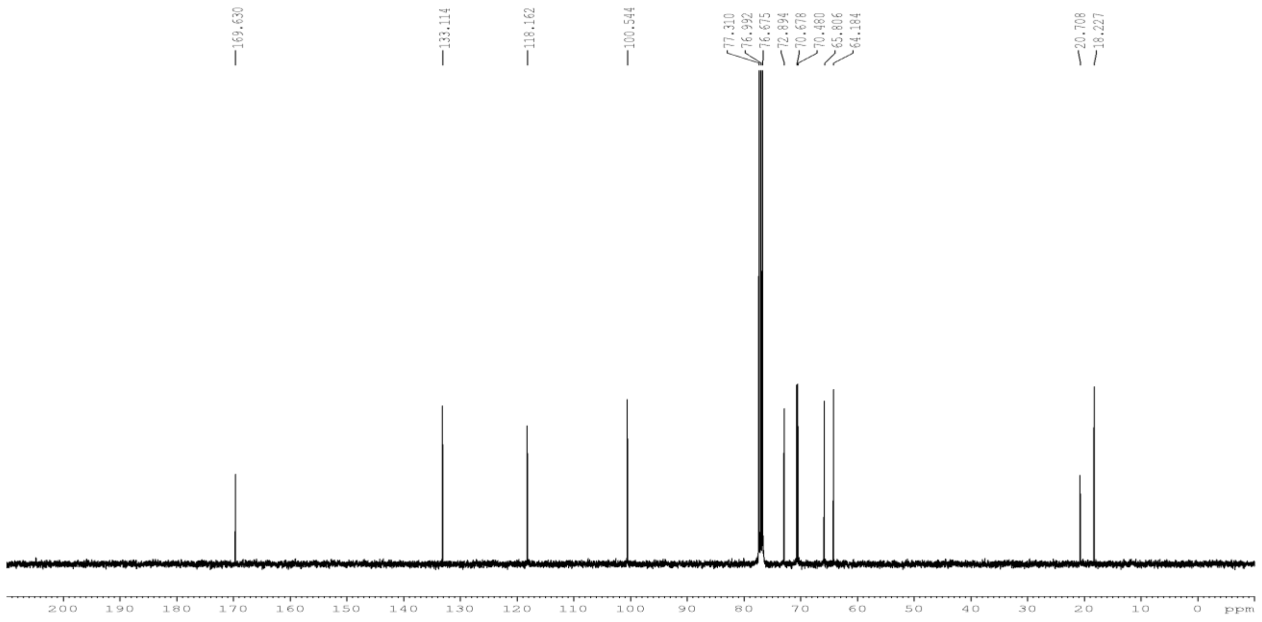

Figure. $^{13}C\{^{1}H\}$ NMR (100 MHz, $CDCl_3$) spectrum of **56**.

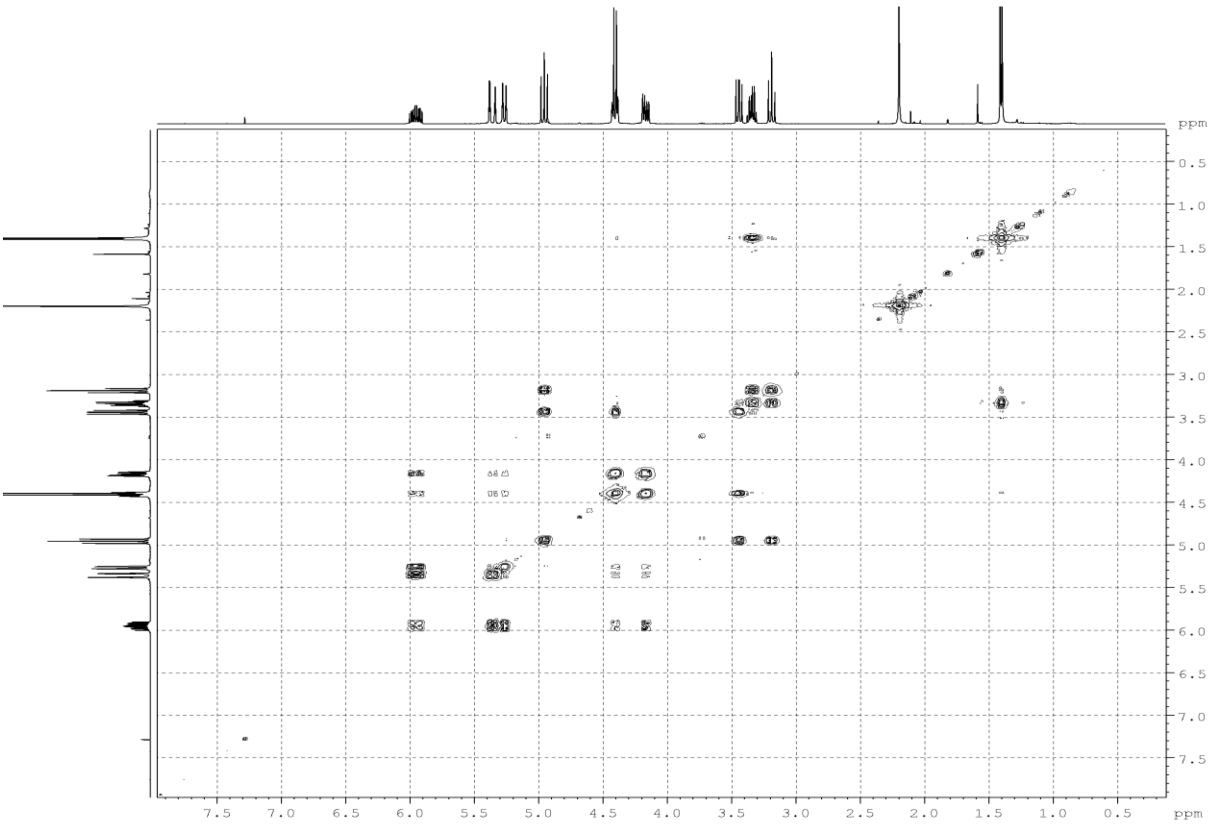

Figure. ^{1}H-^{1}H COSY NMR (400 MHz, $CDCl_3$) spectrum of **56**.

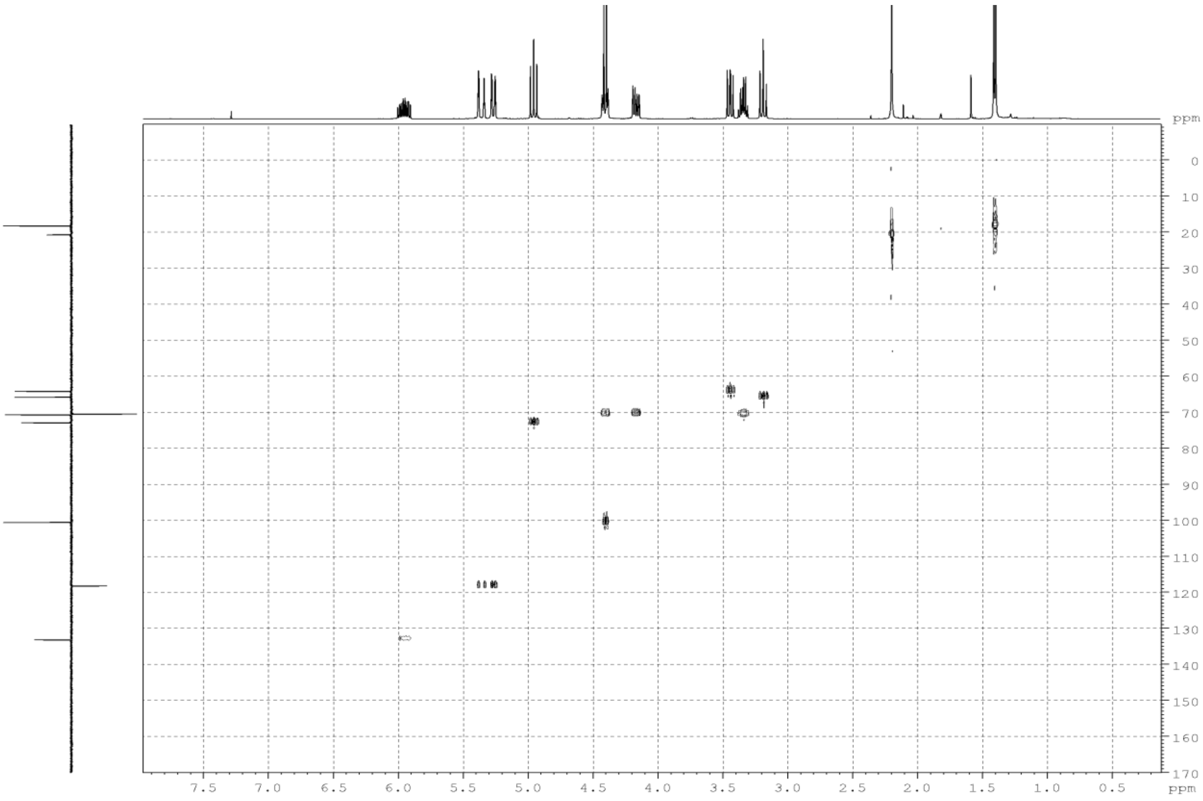

Figure. DEPT-HSQC NMR spectrum of **56**.

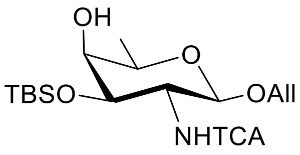

57

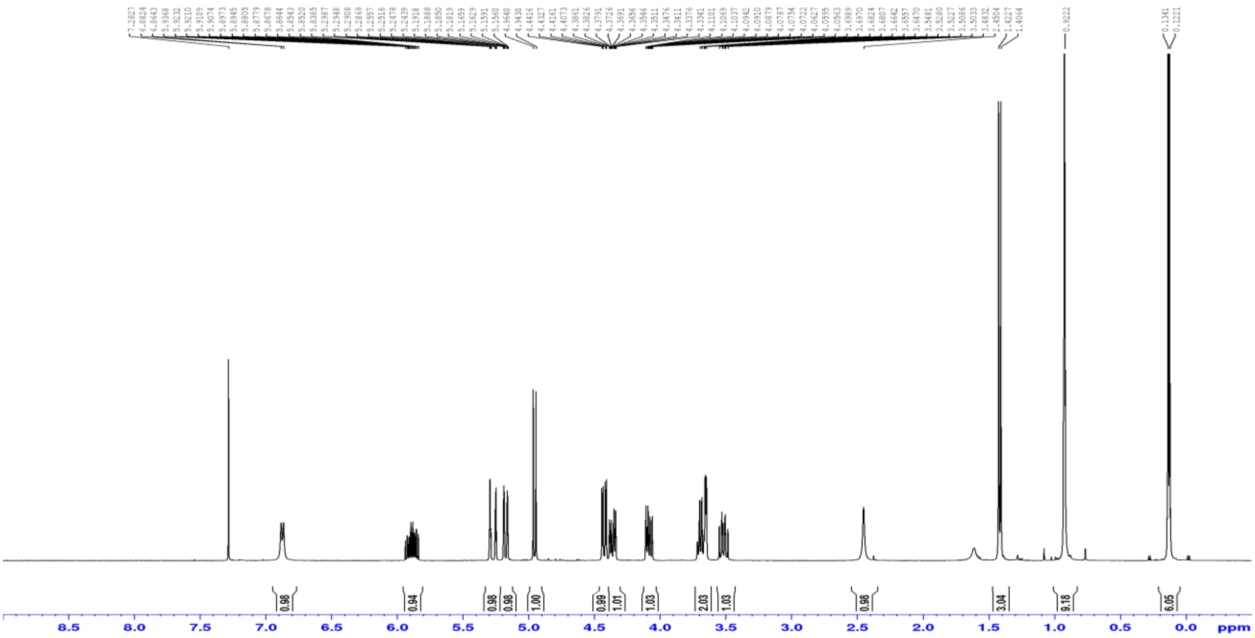

Figure. ^{1}H NMR (400 MHz, $CDCl_3$) spectrum of **57**.

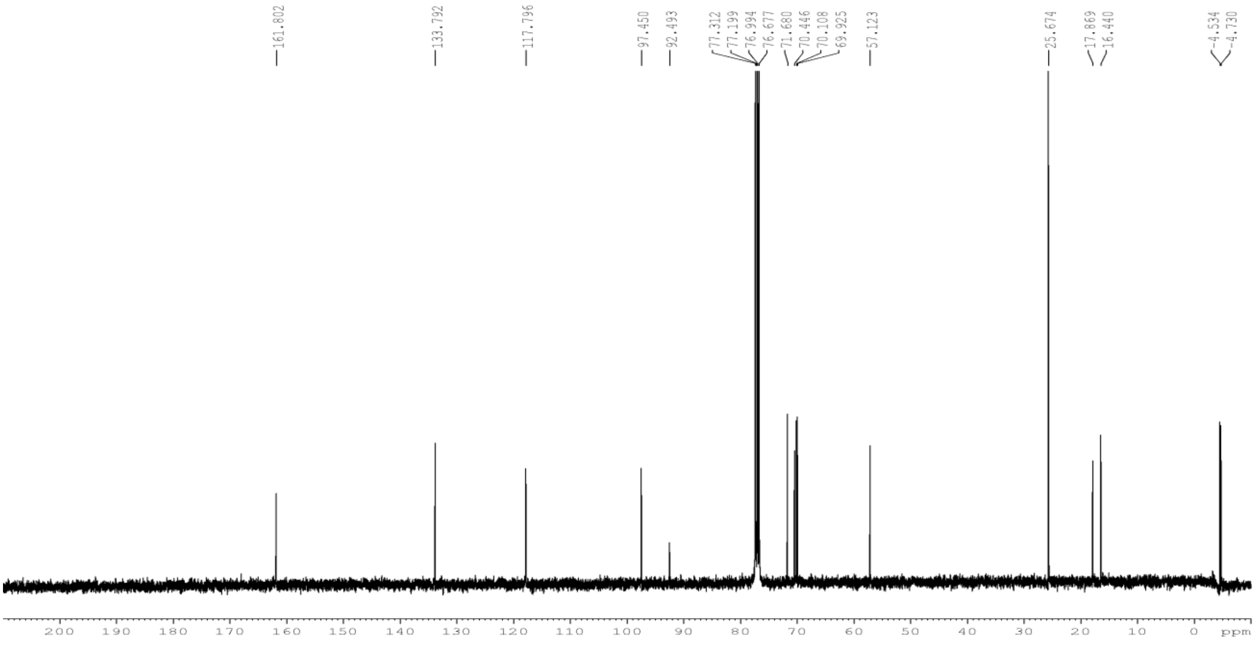

Figure. ^{13}C{^{1}H} NMR (100 MHz, $CDCl_3$) spectrum of **57**.

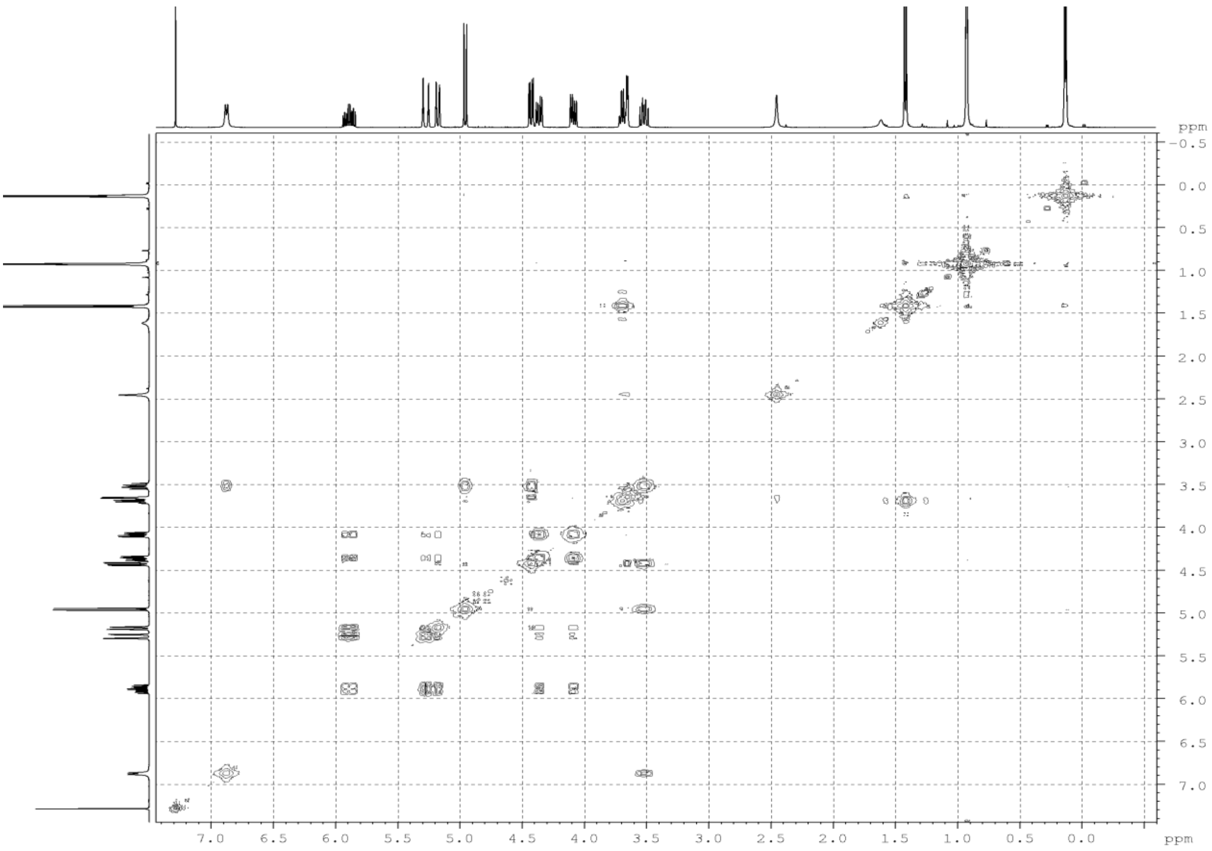

Figure. ^{1}H-^{1}H COSY NMR (400 MHz, $CDCl_3$) spectrum of **57**.

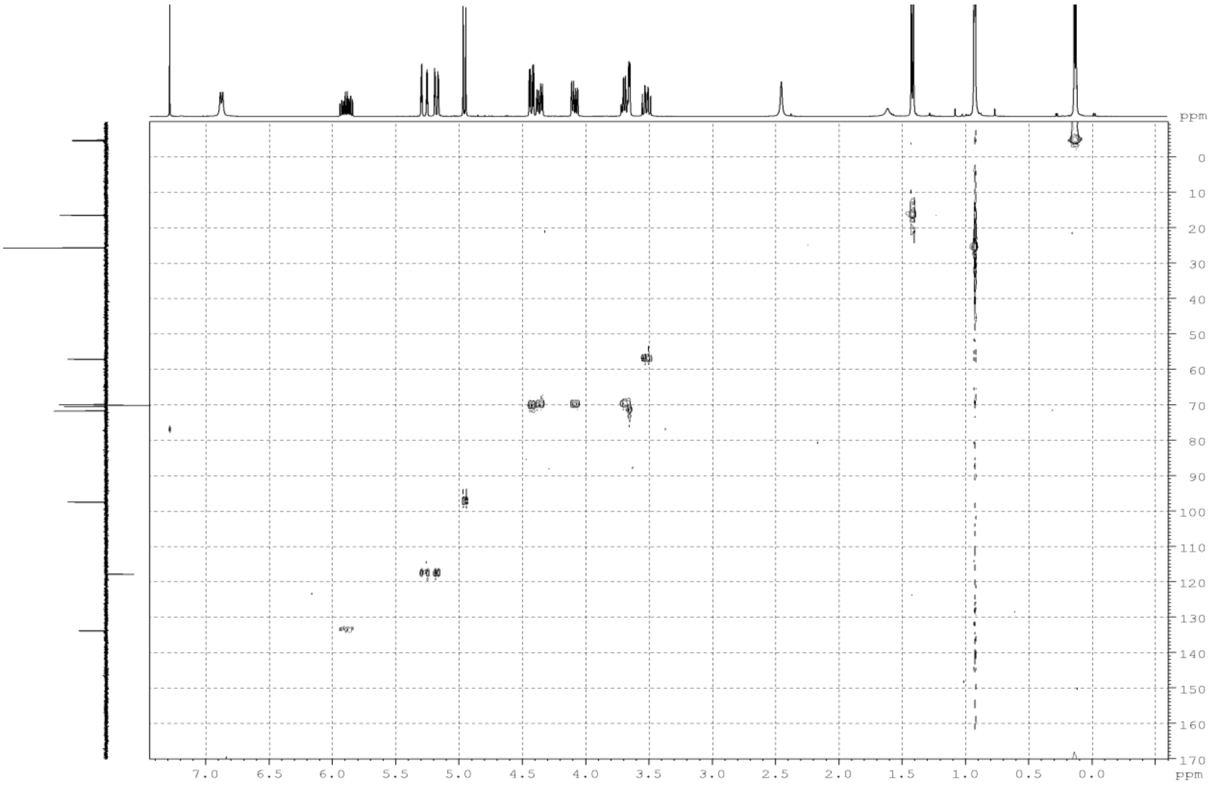

Figure. DEPT-HSQC NMR spectrum of **57**.

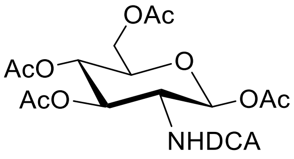

S2

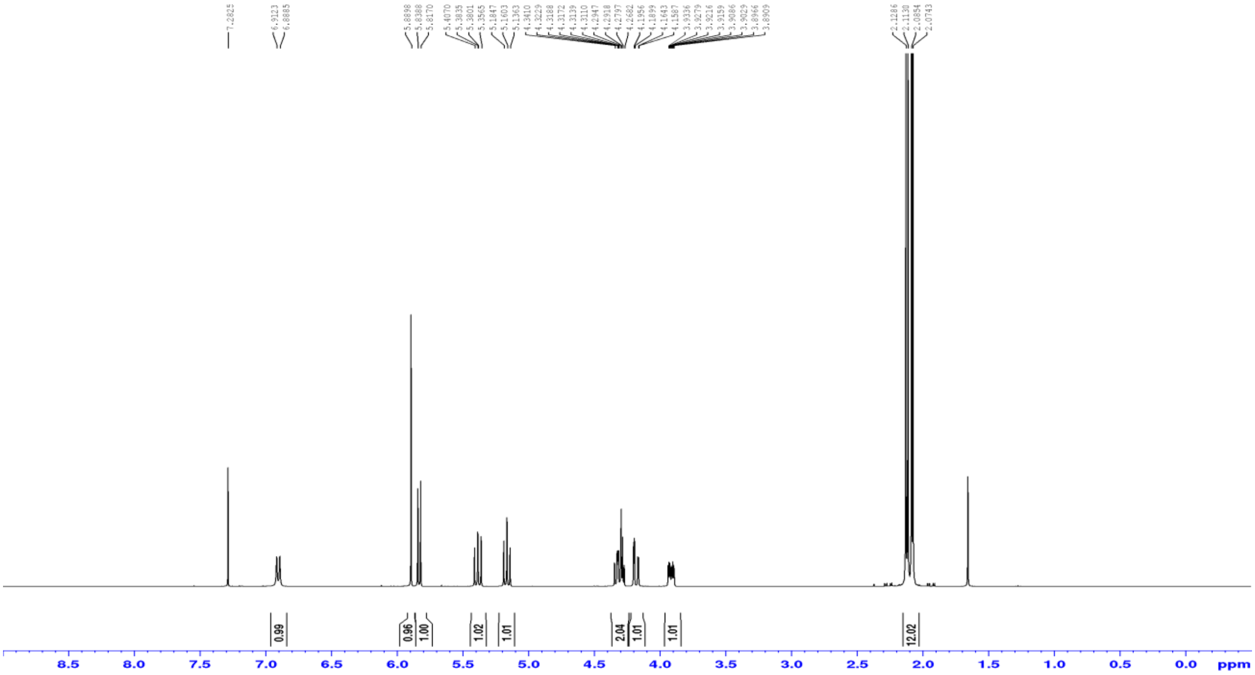

Figure. ^{1}H NMR (400 MHz, $CDCl_3$) spectrum of **S2**.

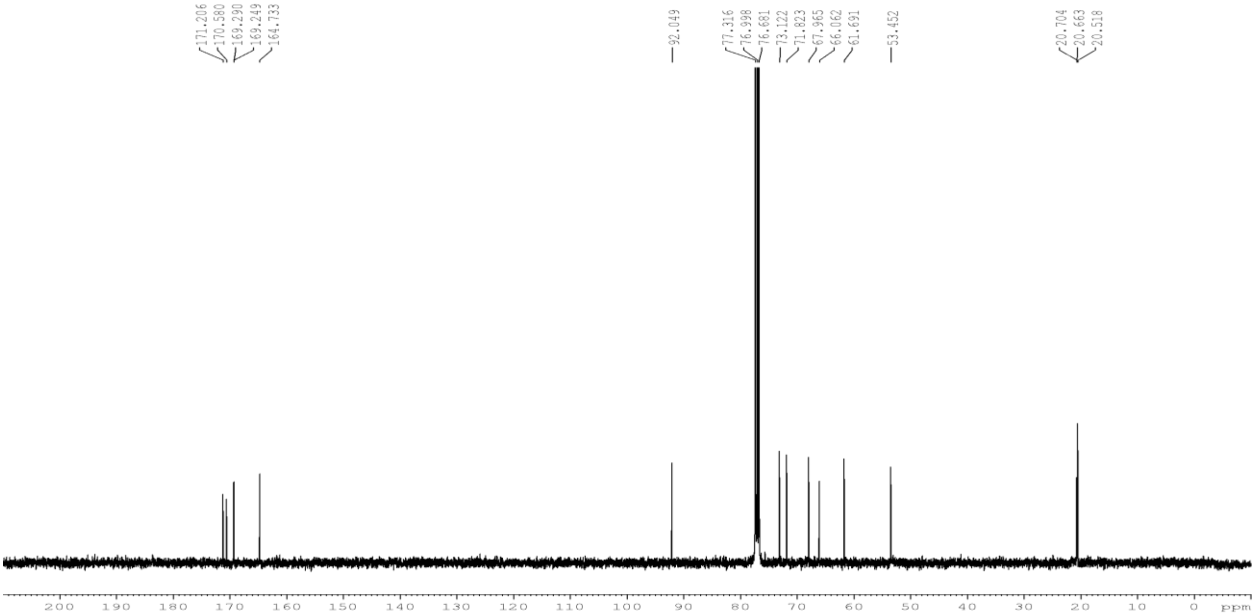

Figure. $^{13}C\{^{1}H\}$ NMR (100 MHz, $CDCl_3$) spectrum of **S2**.

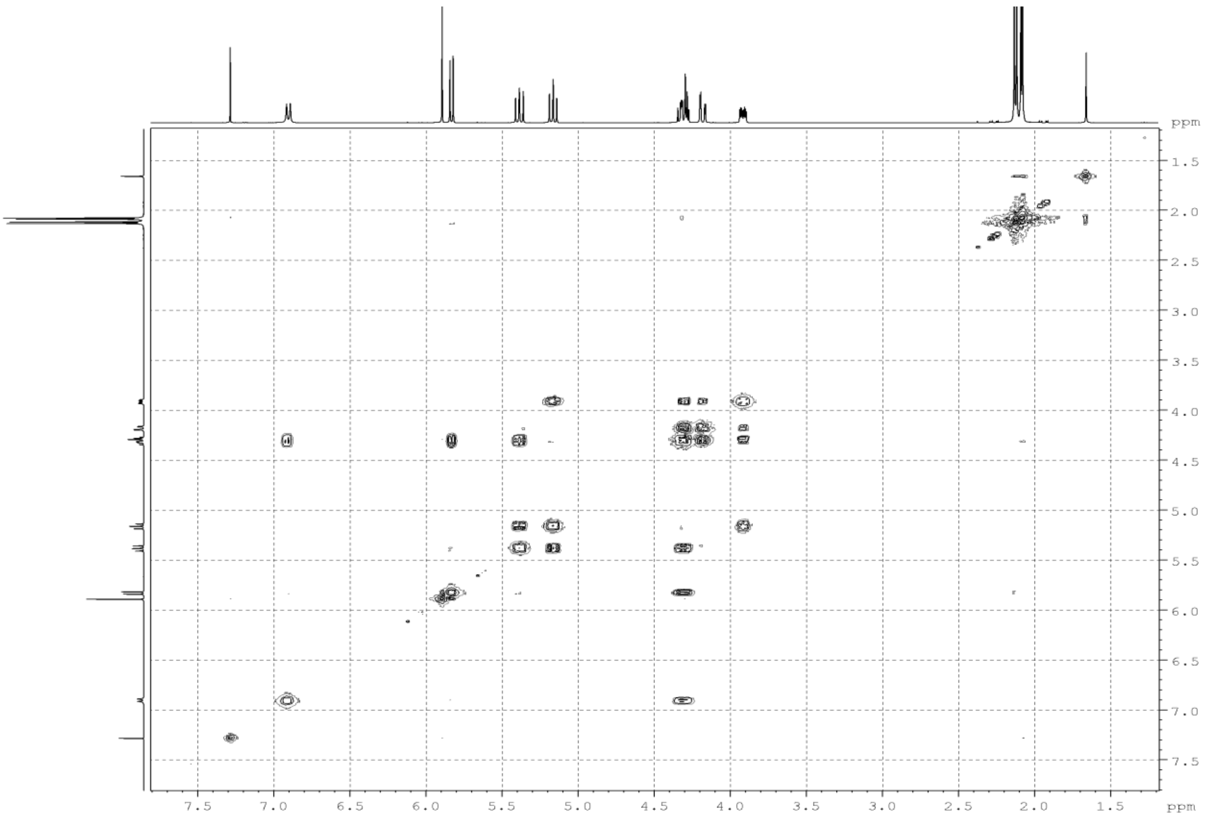

Figure. ^{1}H-^{1}H COSY NMR (400 MHz, $CDCl_3$) spectrum of **S2.**

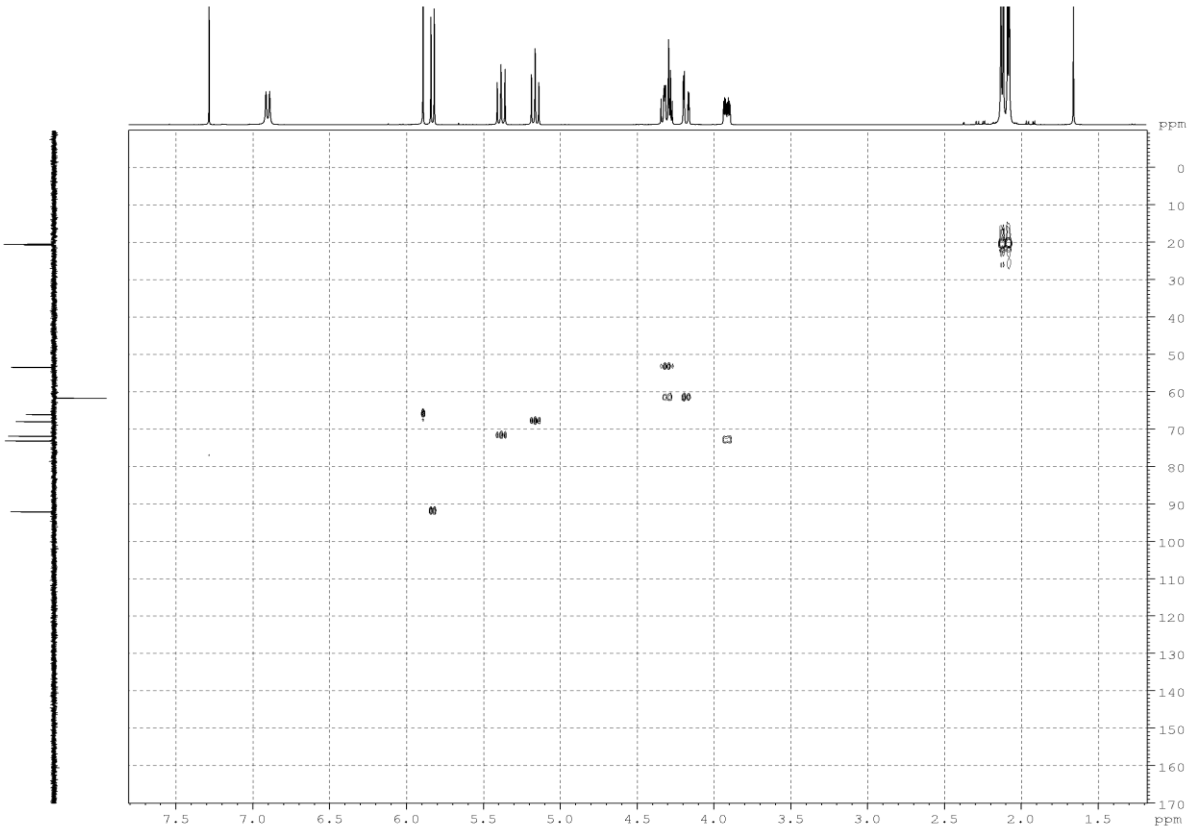

Figure. DEPT-HSQC NMR spectrum of **S2**.

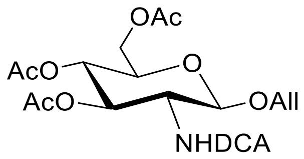

S4

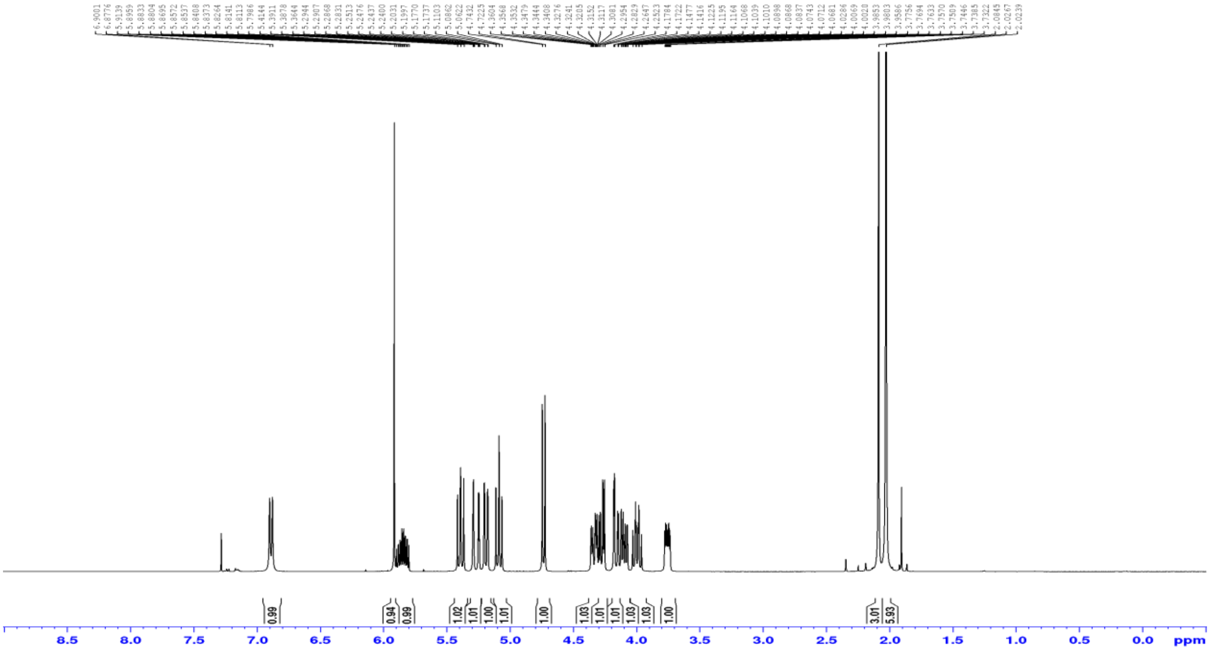

Figure. ^{1}H NMR (400 MHz, $CDCl_3$) spectrum of **S4**.

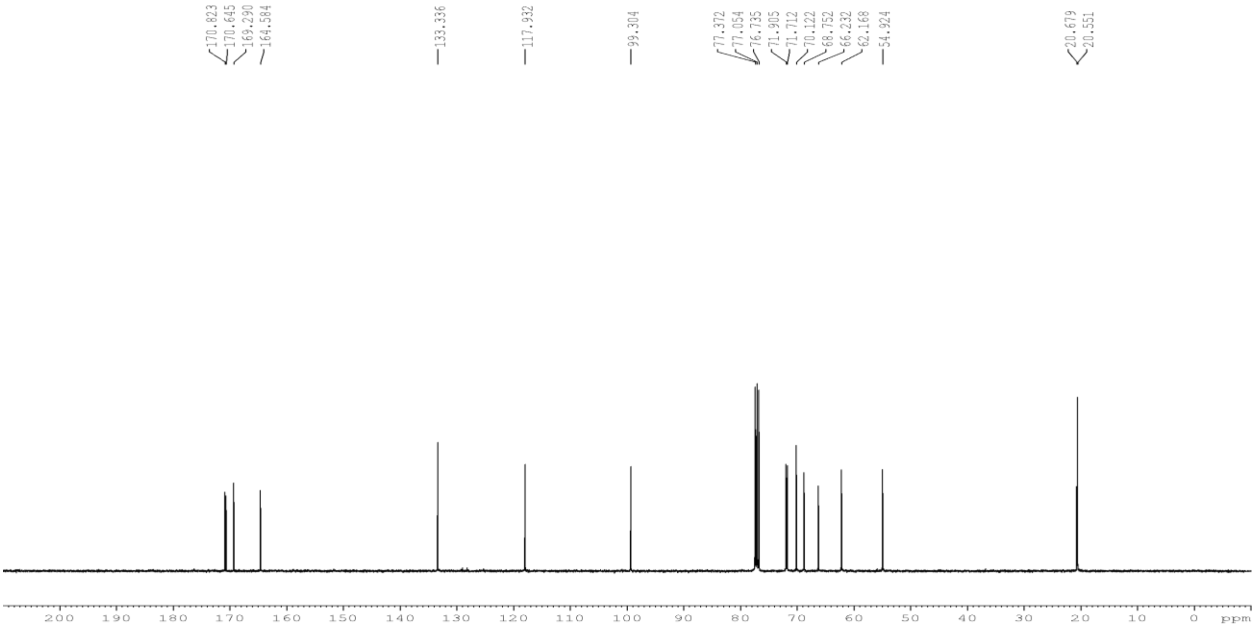

Figure. ^{13}C{^{1}H} NMR (100 MHz, $CDCl_3$) spectrum of **S4**.

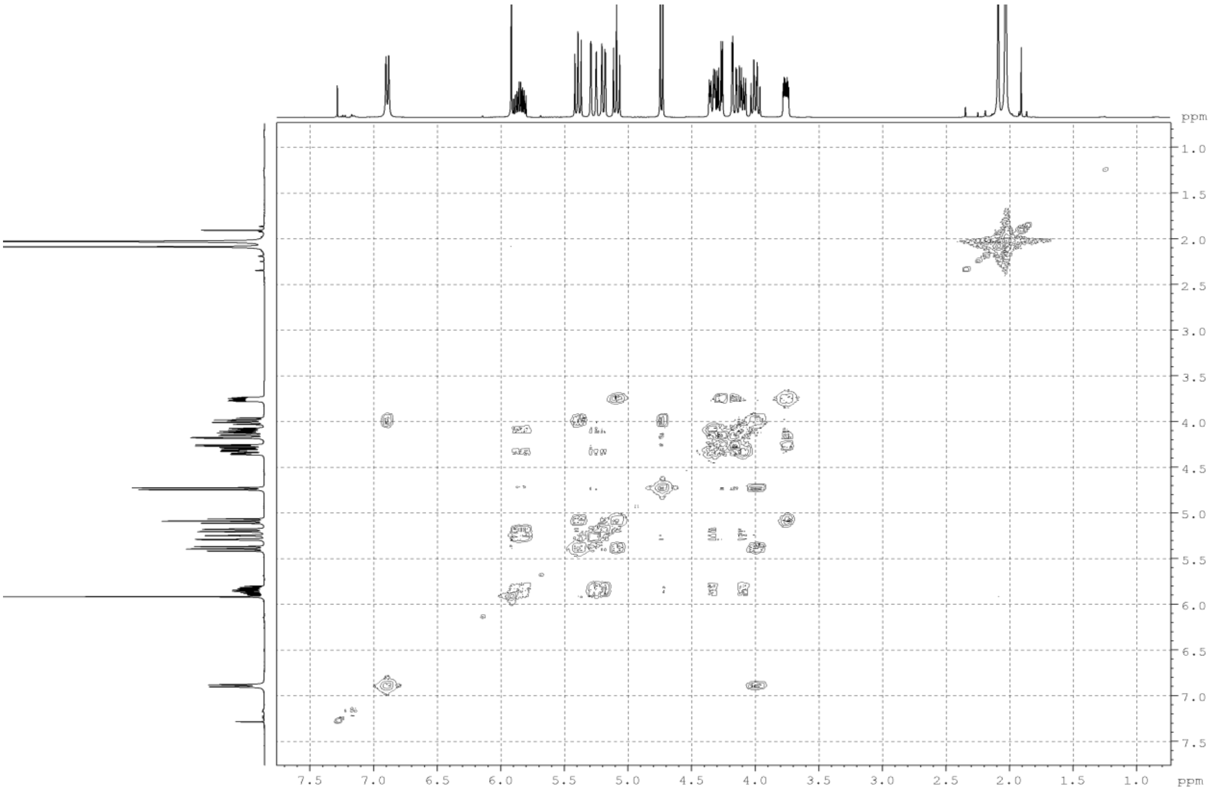

Figure. ^{1}H-^{1}H COSY NMR (400 MHz, $CDCl_3$) spectrum of **S4.**

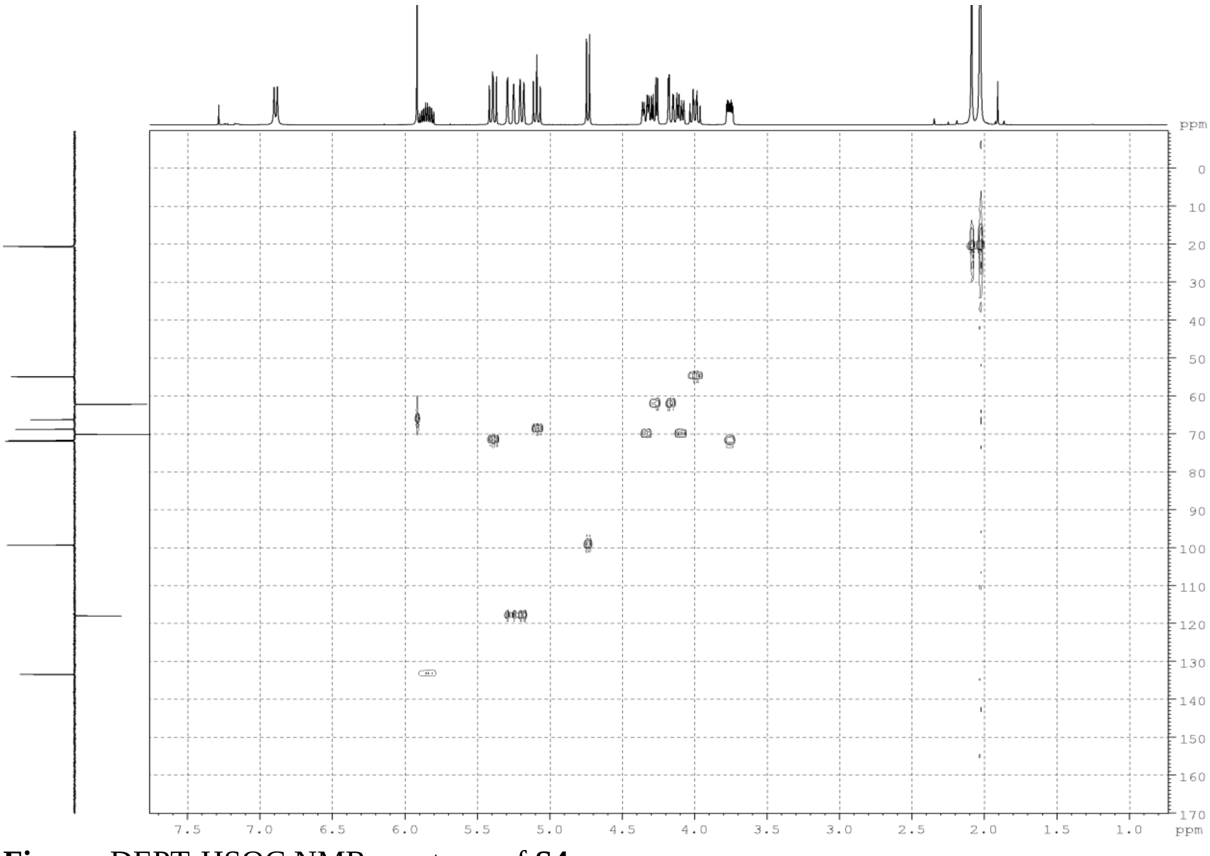

Figure. DEPT-HSQC NMR spectrum of **S4**.

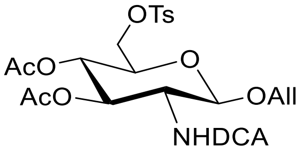

S6

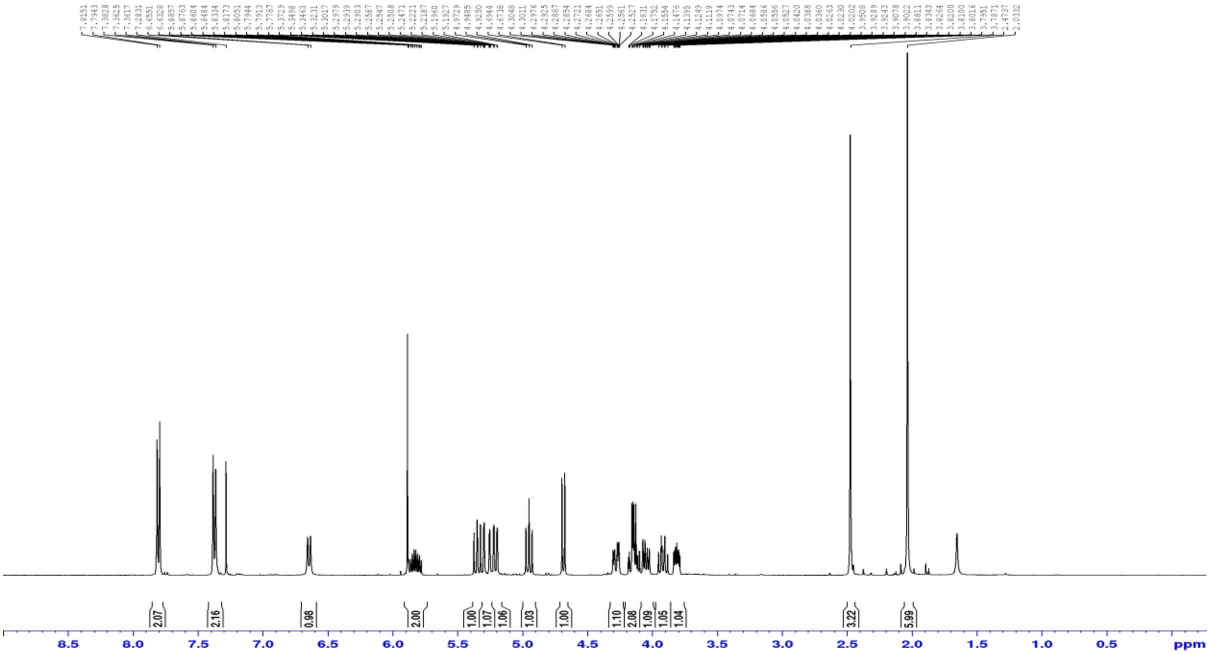

Figure. ^{1}H NMR (400 MHz, $CDCl_3$) spectrum of **S6**.

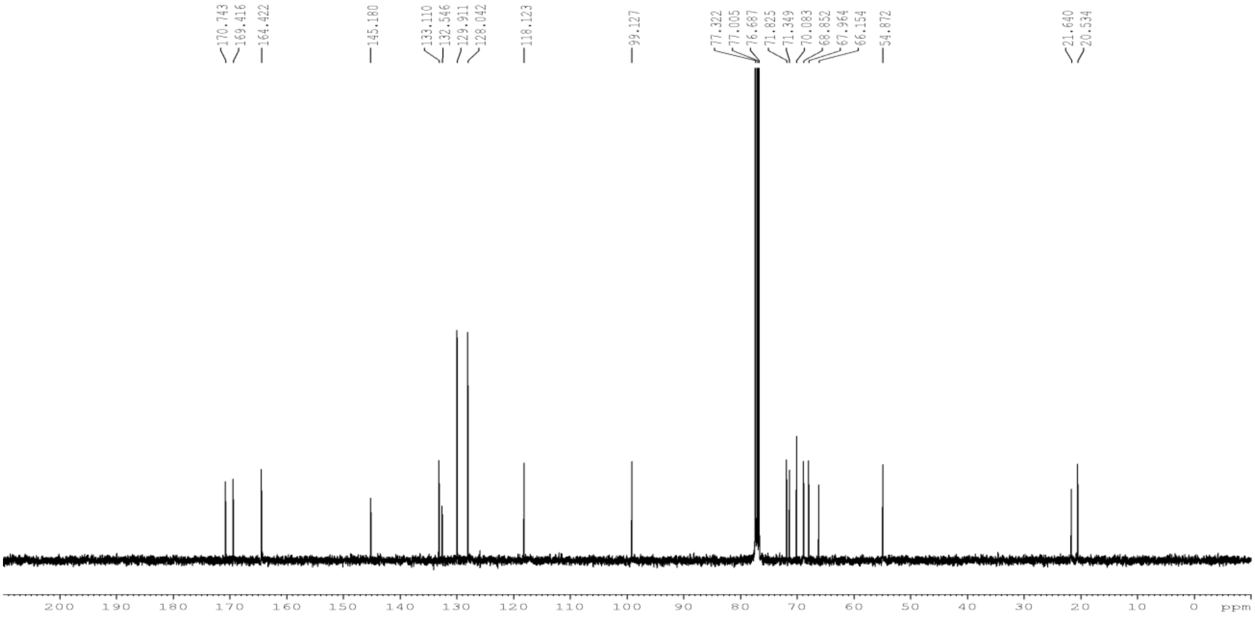

Figure. $^{13}C\{^{1}H\}$ NMR (100 MHz, $CDCl_3$) spectrum of **S6**.

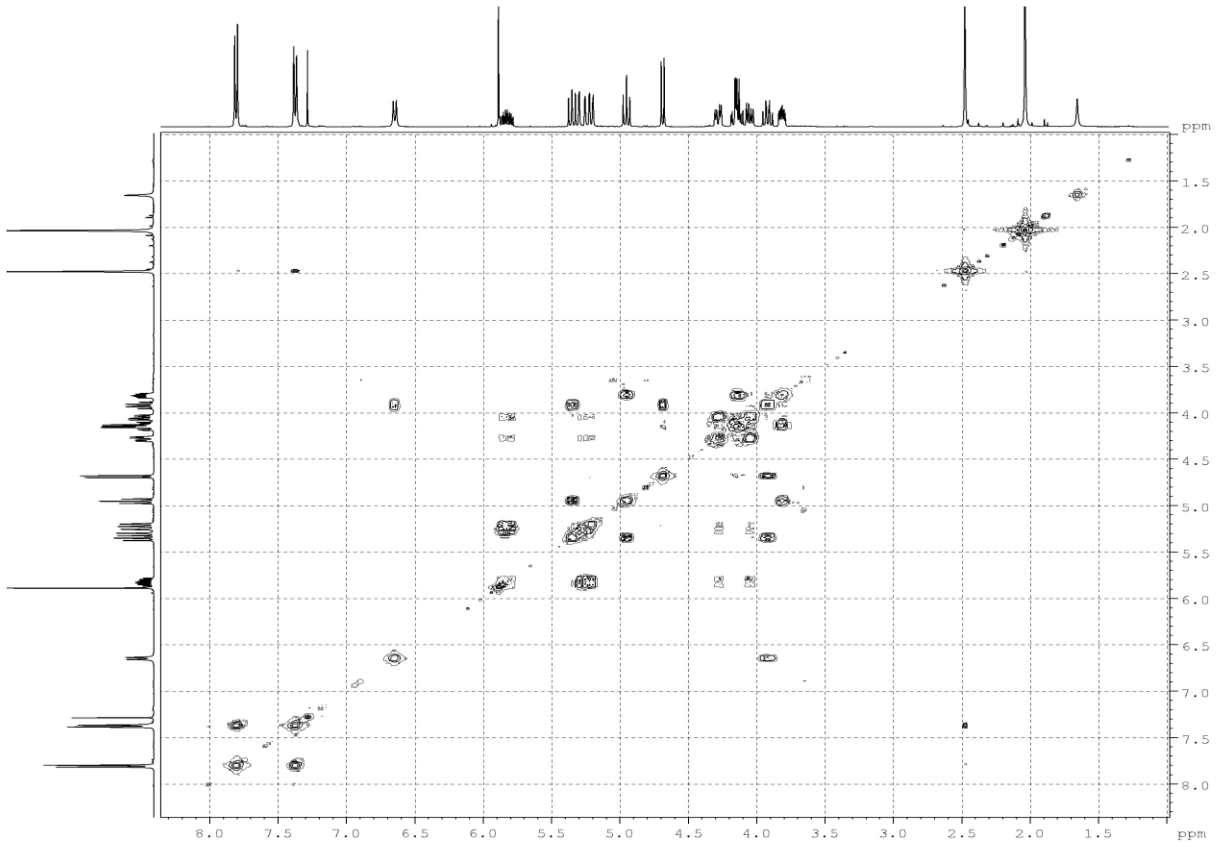

Figure. ^{1}H-^{1}H COSY NMR (400 MHz, $CDCl_3$) spectrum of **S6.**

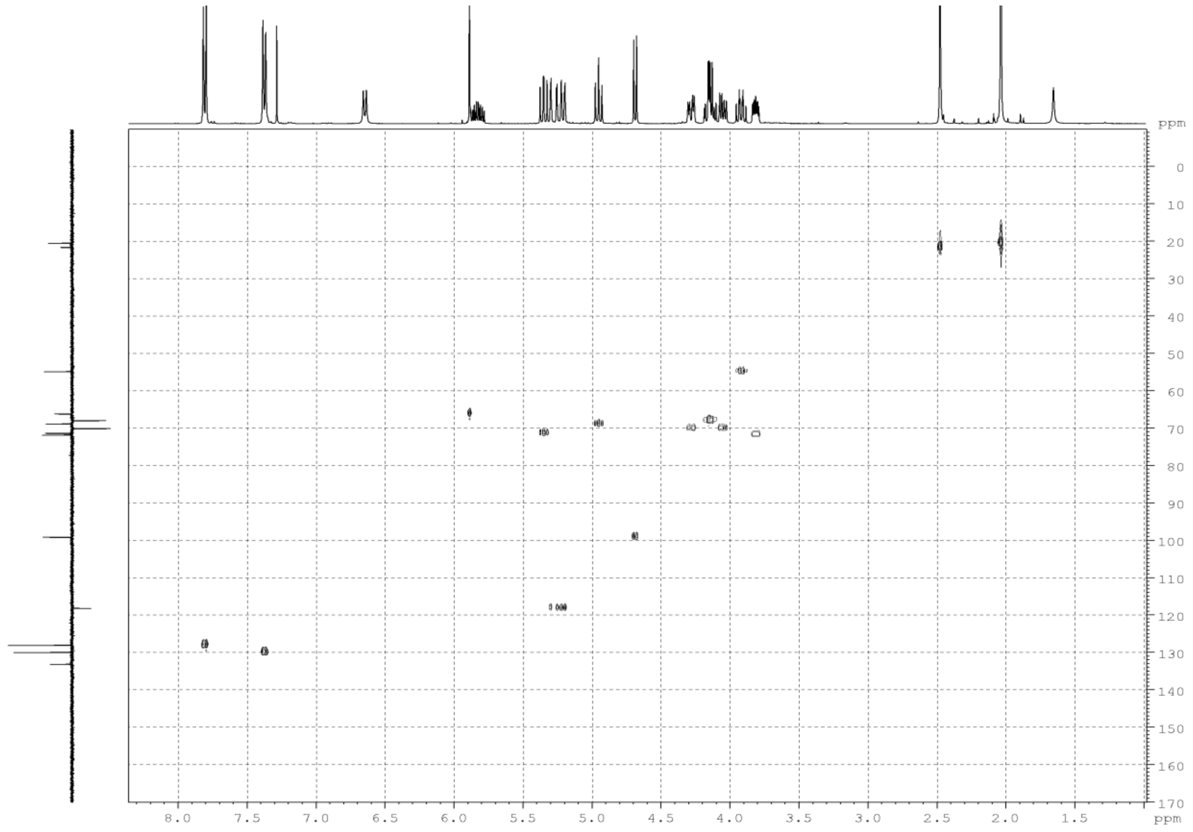

Figure. DEPT-HSQC NMR spectrum of **S6**.

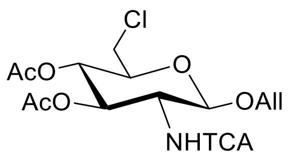

S7

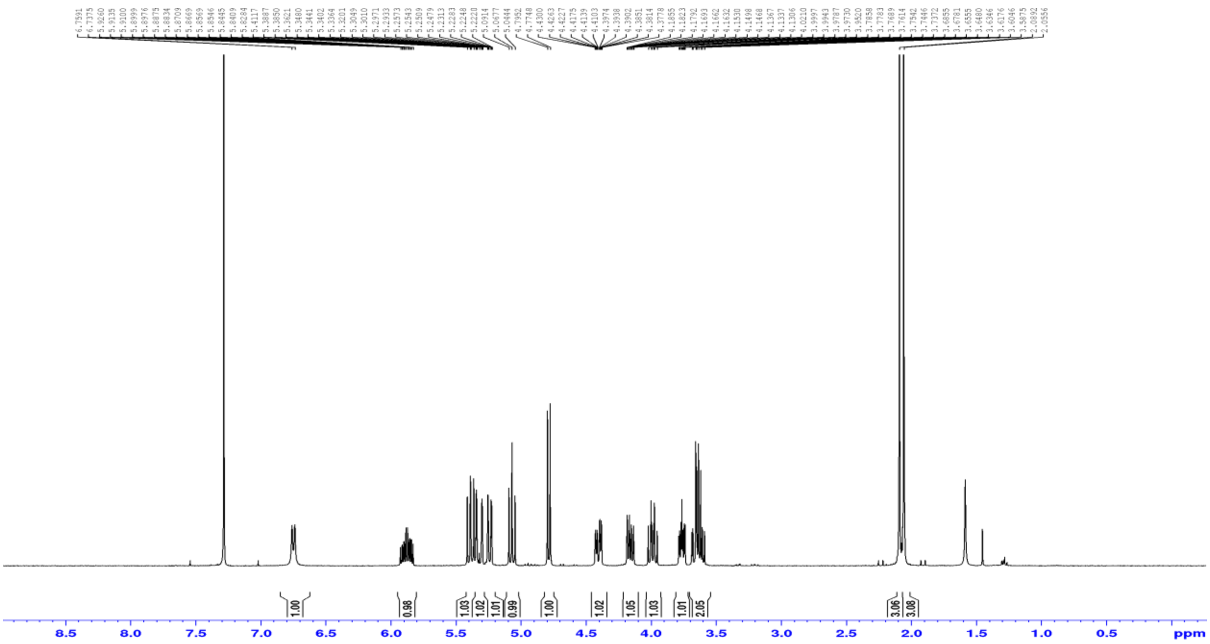

Figure. ^{1}H NMR (400 MHz, $CDCl_3$) spectrum of **S7**.

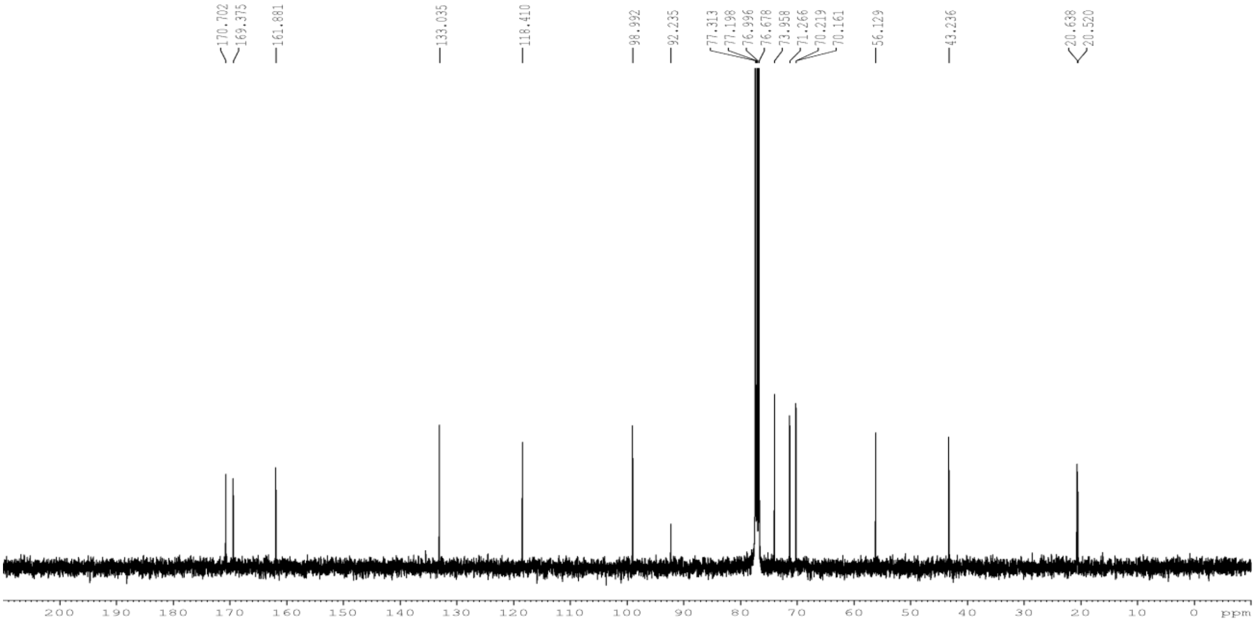

Figure. $^{13}C\{^{1}H\}$ NMR (100 MHz, $CDCl_3$) spectrum of **S7**.

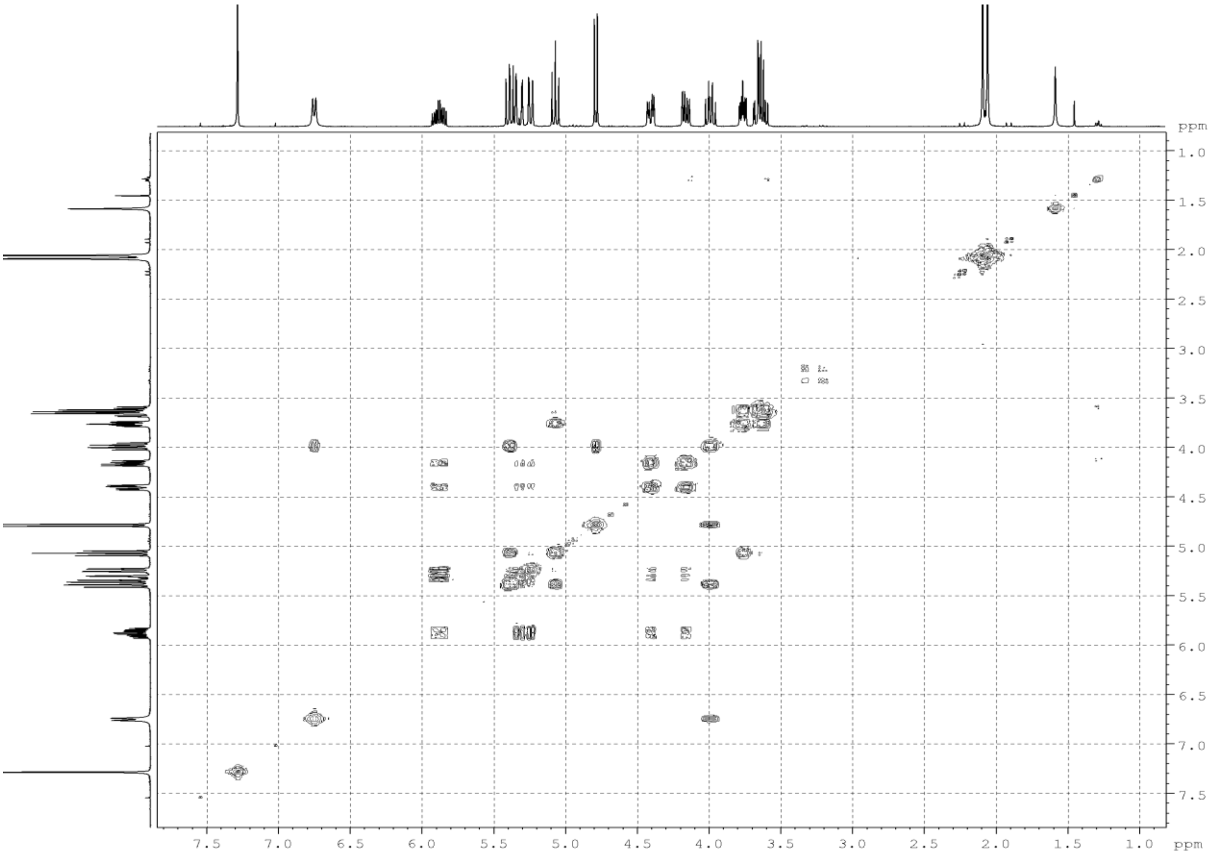

Figure. ^{1}H-^{1}H COSY NMR (400 MHz, $CDCl_3$) spectrum of **S7.**

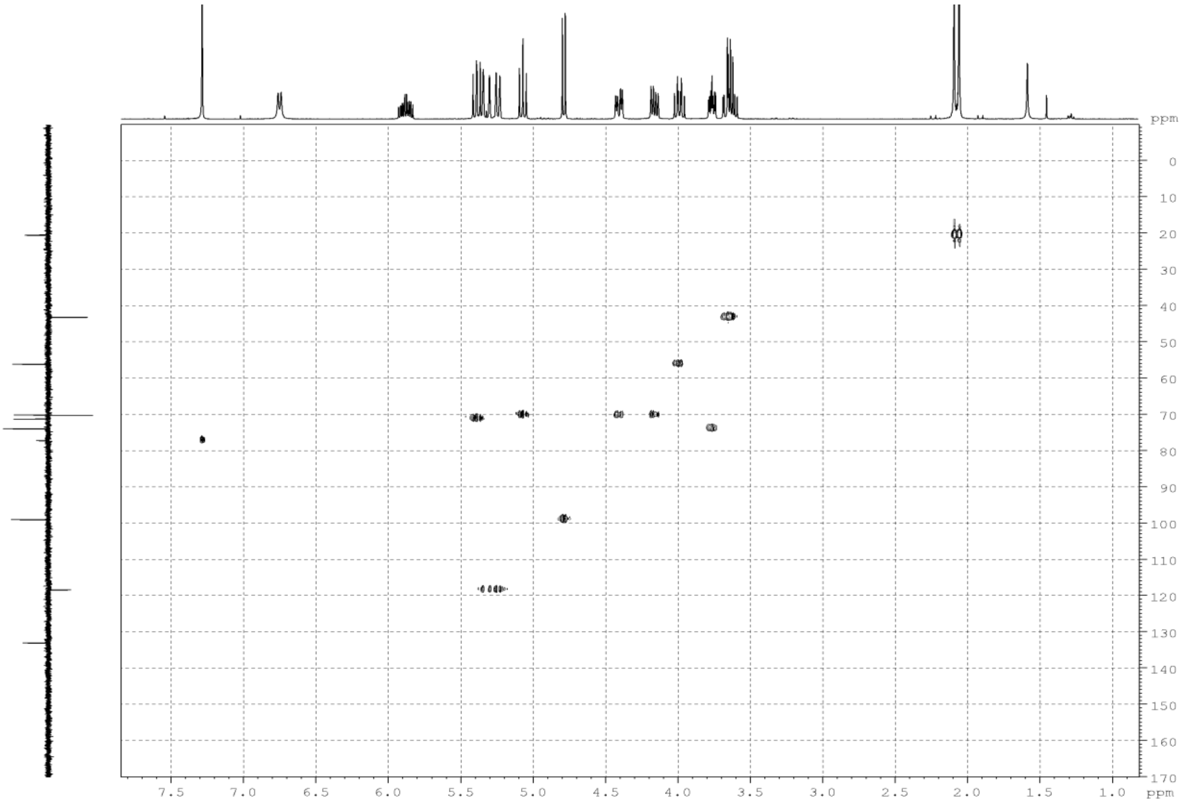

Figure. DEPT-HSQC NMR spectrum of **S7**.

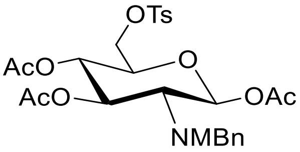

S9

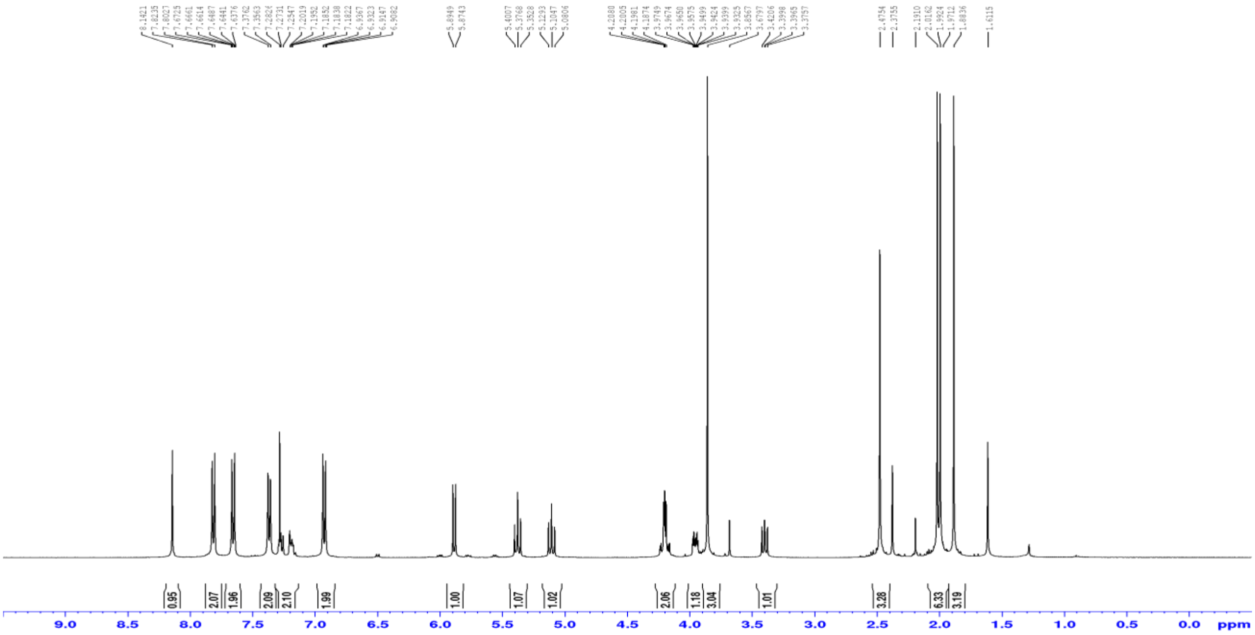

Figure. ^{1}H NMR (400 MHz, $CDCl_3$) spectrum of **S9**.

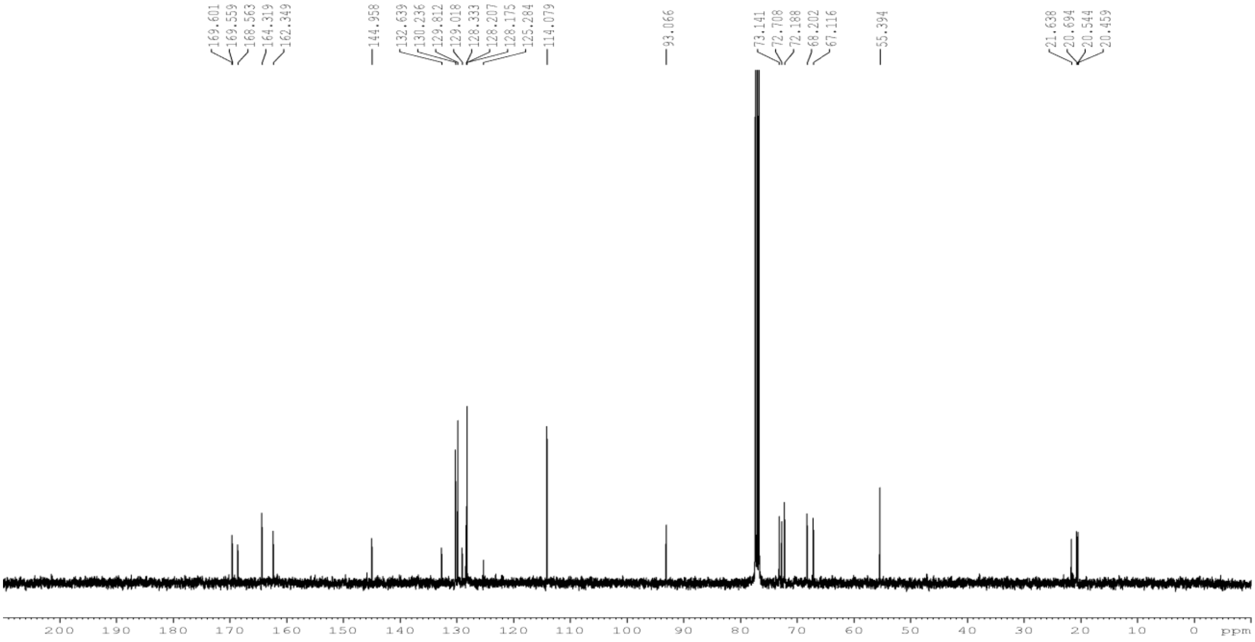

Figure. ^{13}C{^{1}H} NMR (100 MHz, $CDCl_3$) spectrum of **S9**.

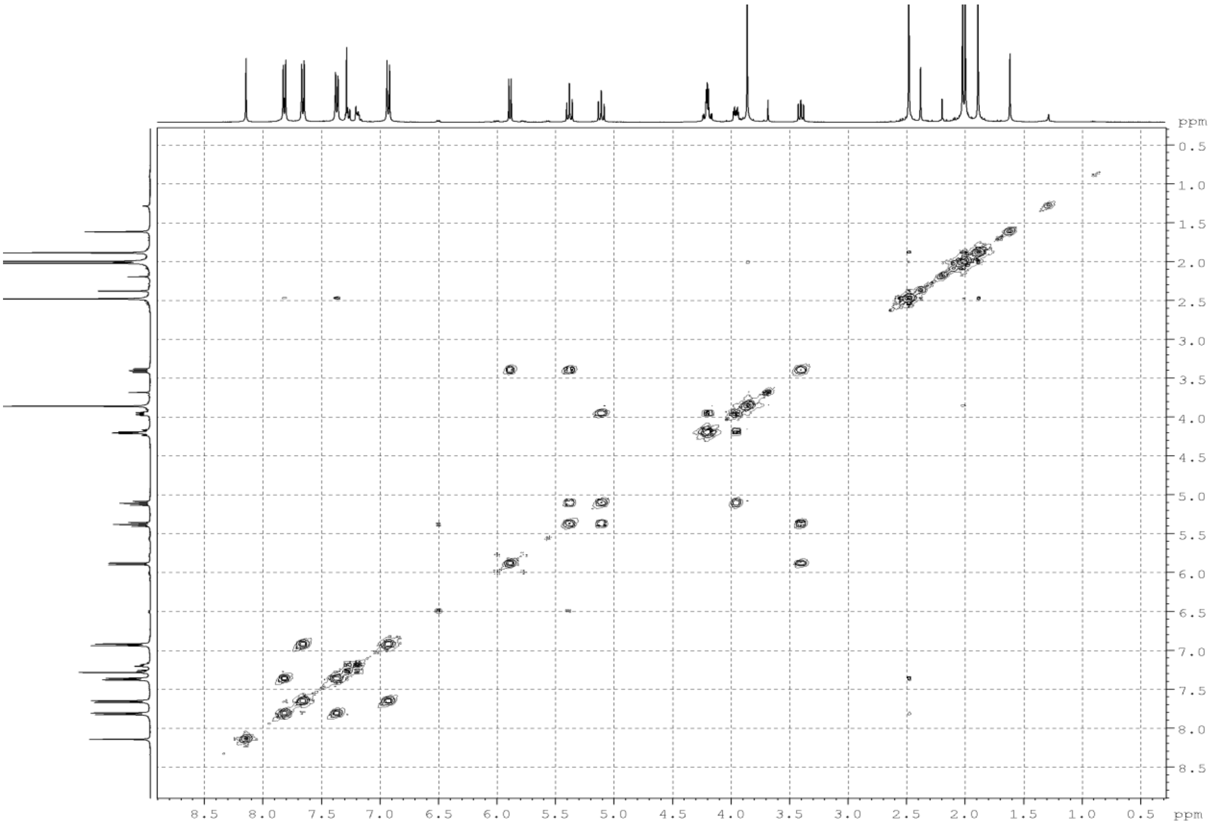

Figure. ^{1}H-^{1}H COSY NMR (400 MHz, $CDCl_3$) spectrum of **S9.**

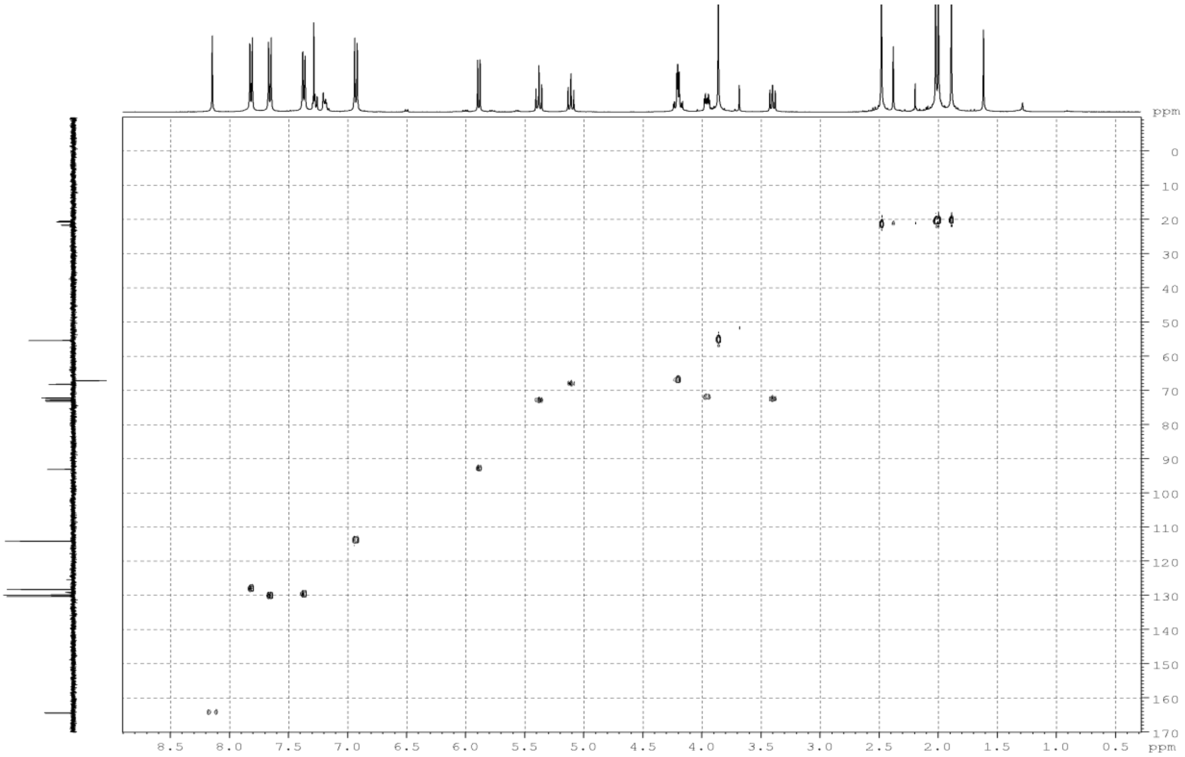

Figure. DEPT-HSQC NMR spectrum of **S9**.

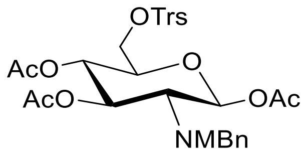

S10

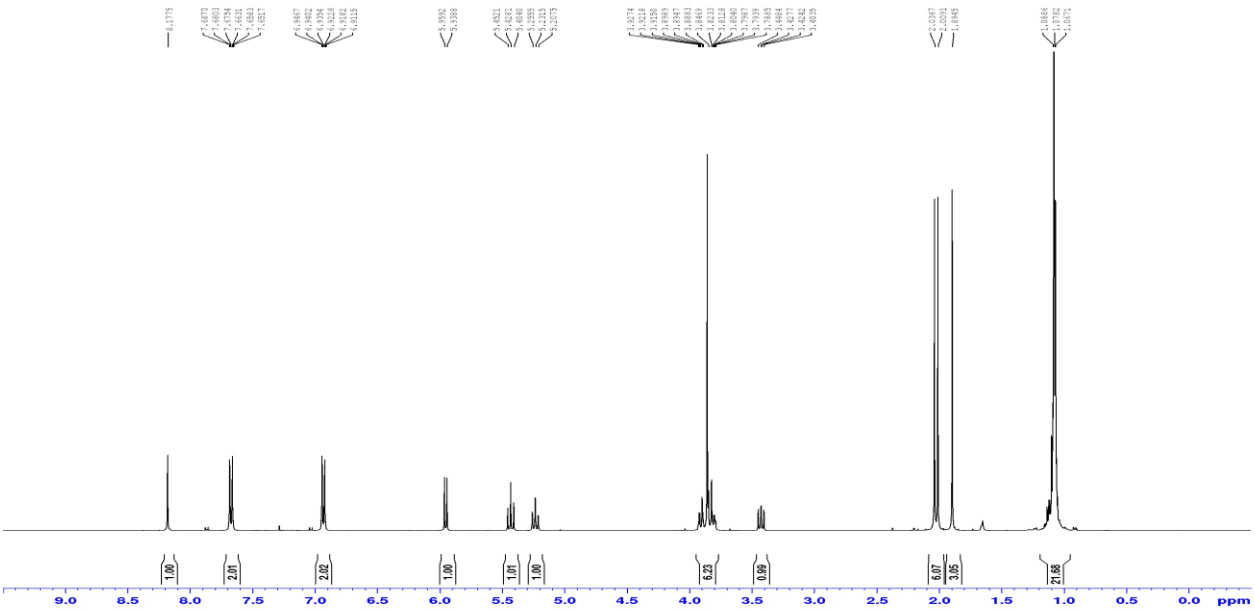

Figure. ^{1}H NMR (400 MHz, $CDCl_3$) spectrum of **S10**.

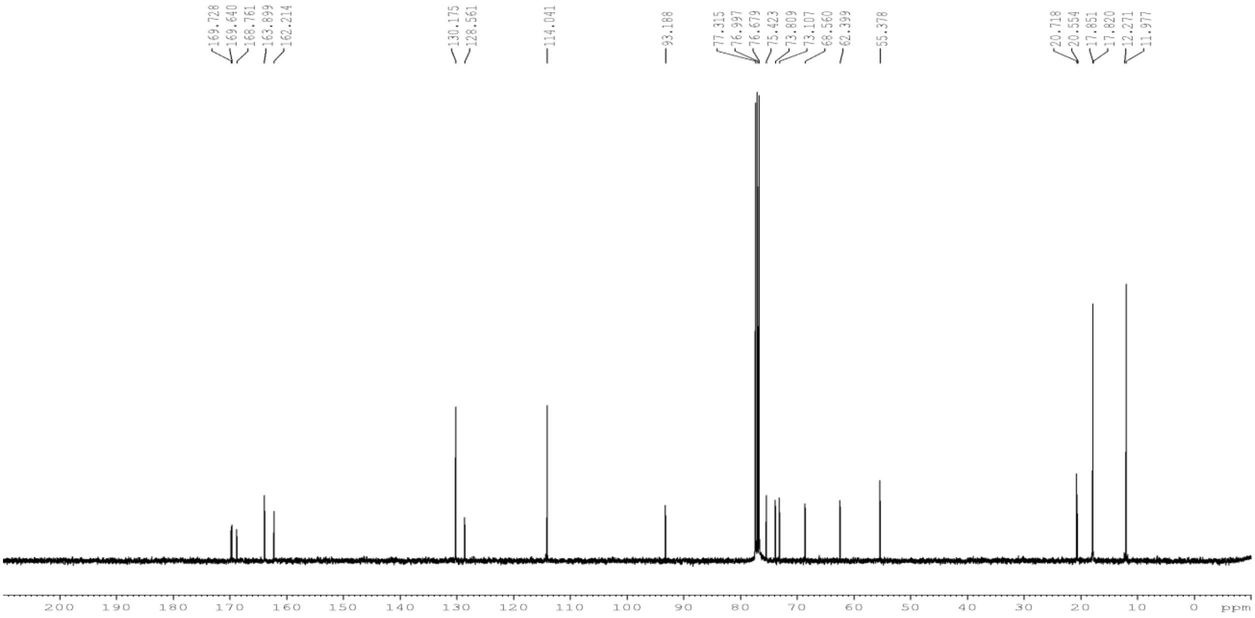

Figure. ^{13}C{^{1}H} NMR (100 MHz, $CDCl_3$) spectrum of **S10**.

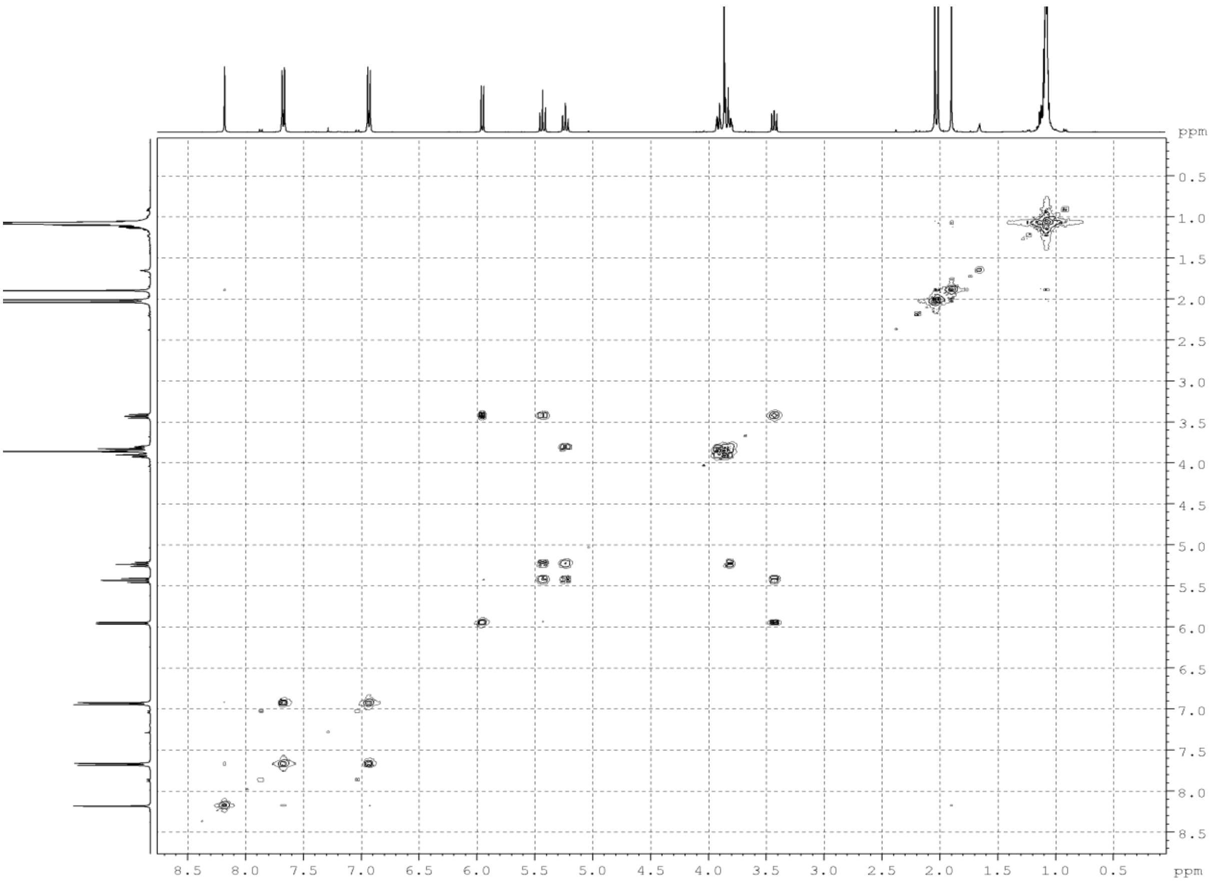

Figure. ^{1}H-^{1}H COSY NMR (400 MHz, $CDCl_3$) spectrum of **S10.**

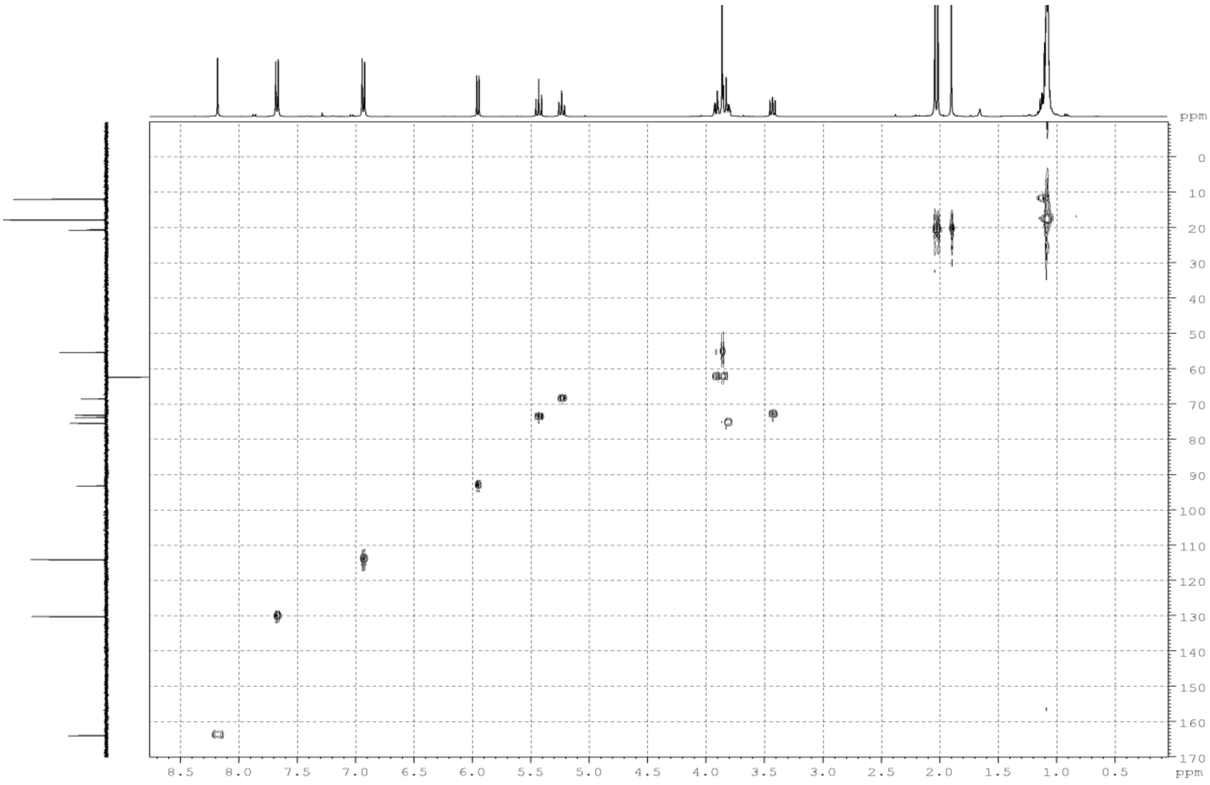

Figure. DEPT-HSQC NMR spectrum of **S10**.

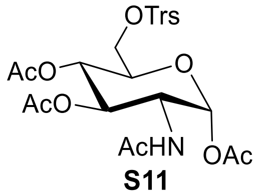

S11

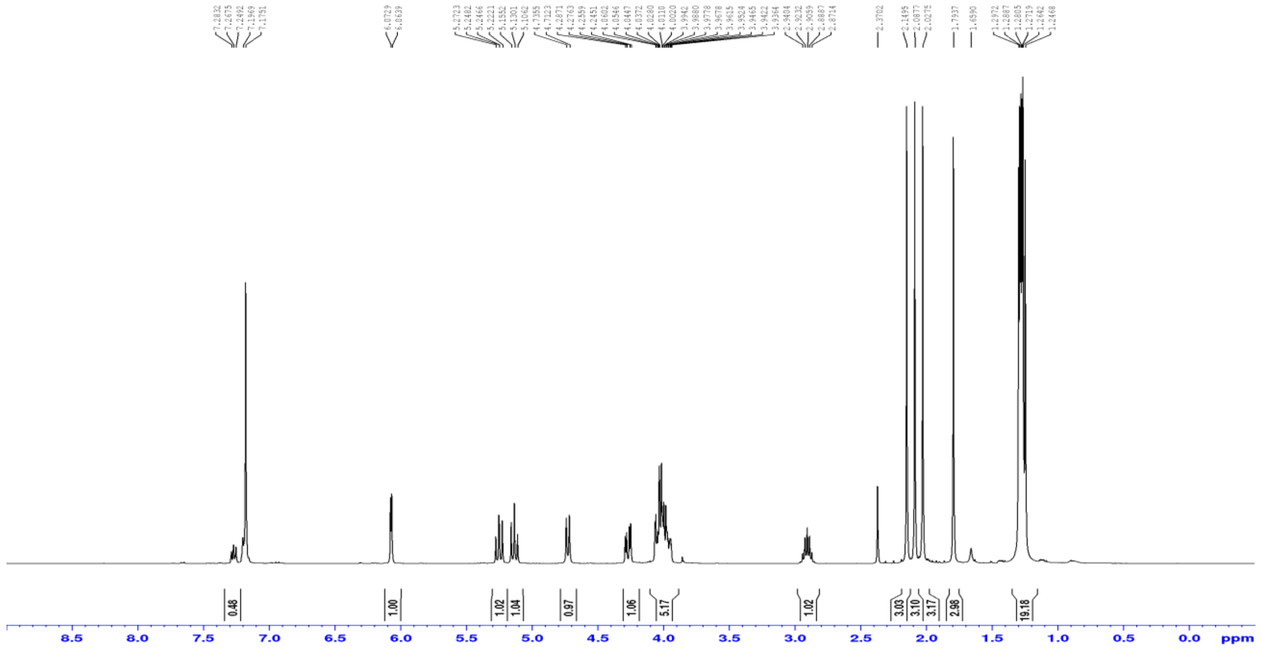

Figure. ^{1}H NMR (400 MHz, $CDCl_3$) spectrum of **S11**.

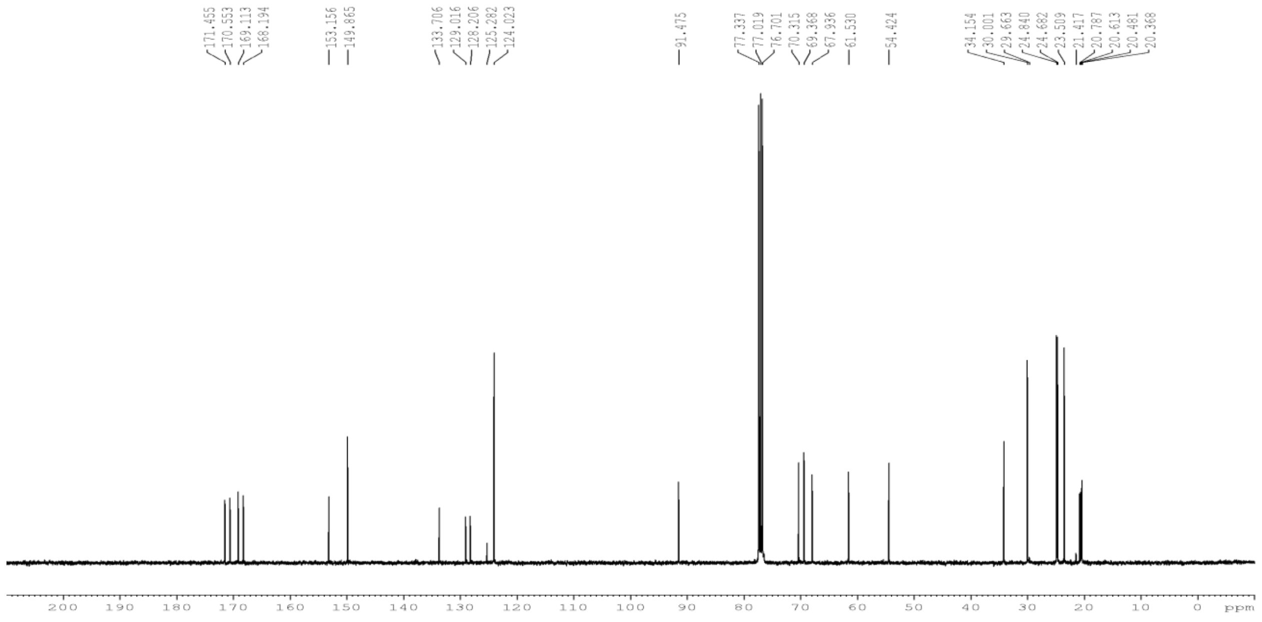

Figure. $^{13}C\{^{1}H\}$ NMR (100 MHz, $CDCl_3$) spectrum of **S11**.

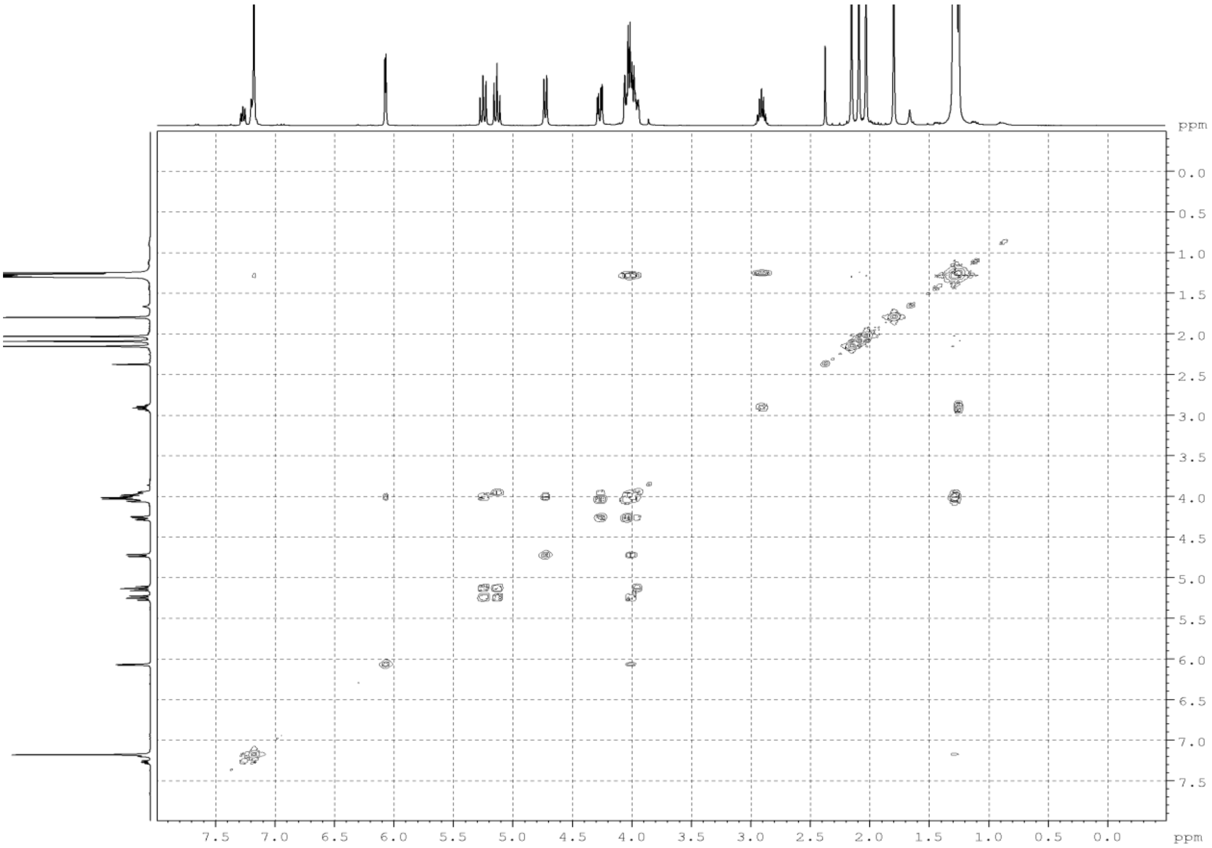

Figure. ^{1}H-^{1}H COSY NMR (400 MHz, $CDCl_3$) spectrum of **S11.**

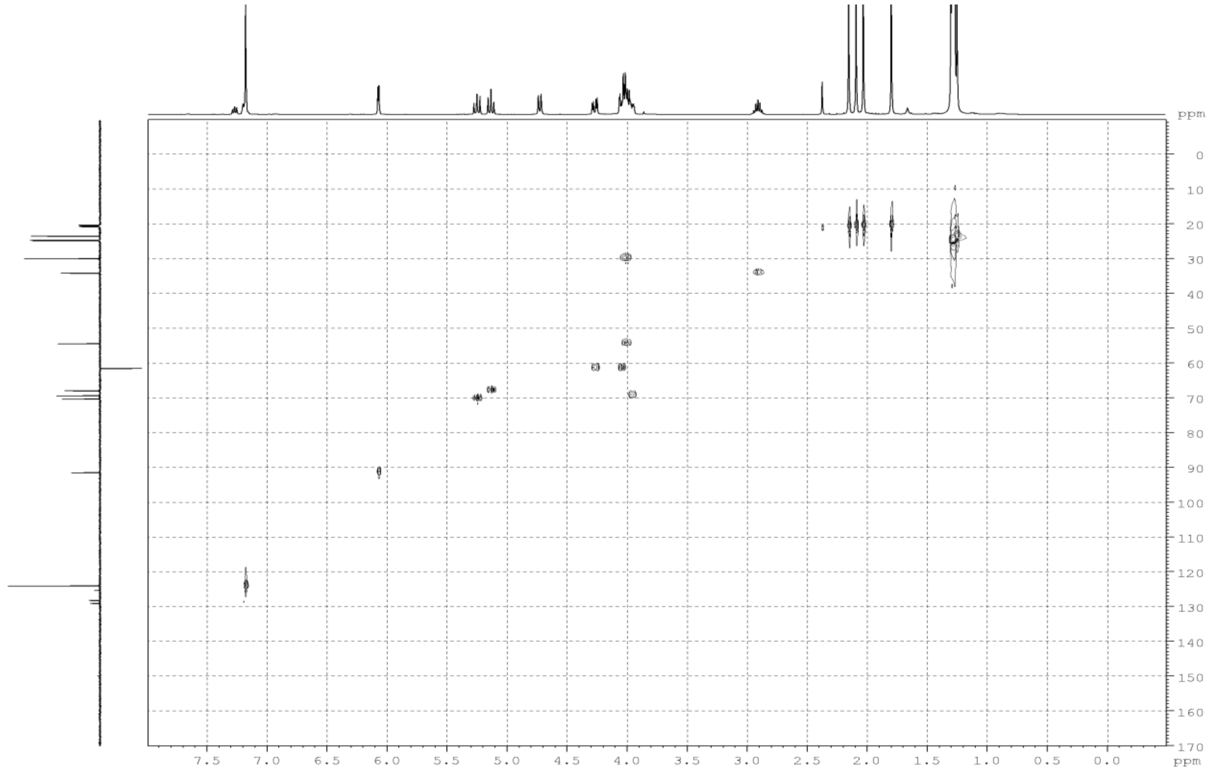

Figure. DEPT-HSQC NMR spectrum of **S11**.

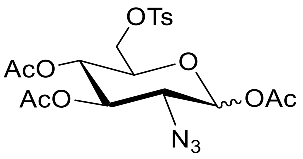

S12

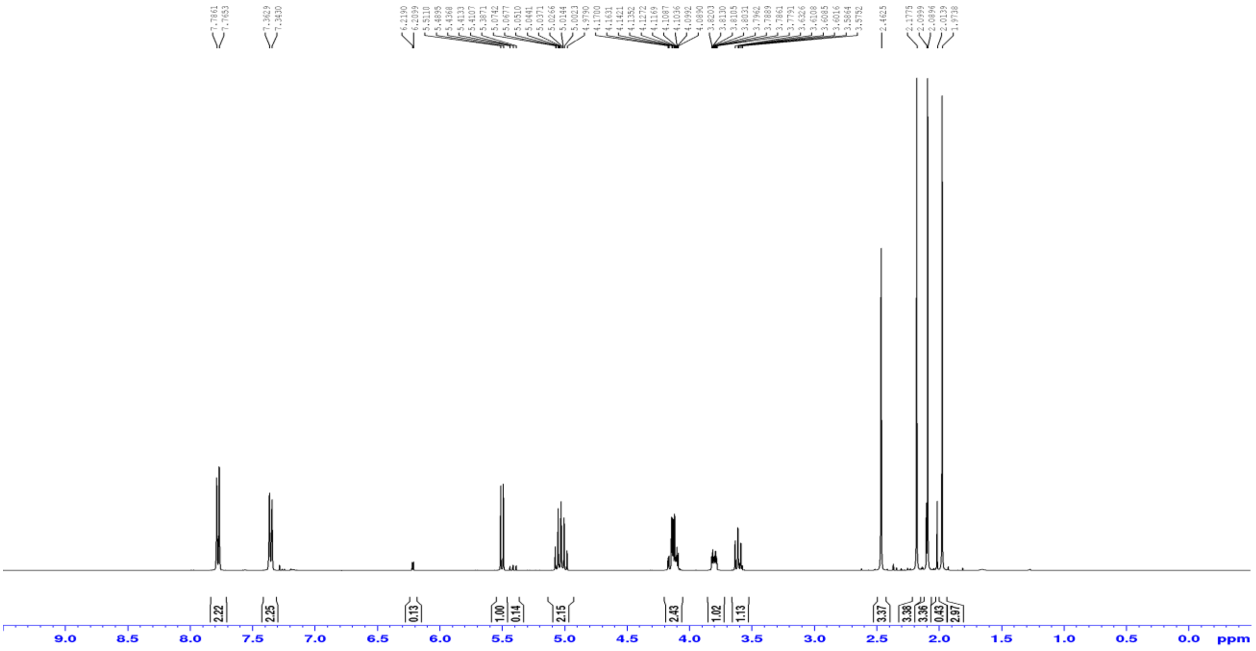

Figure. ^{1}H NMR (400 MHz, $CDCl_3$) spectrum of **S12**.

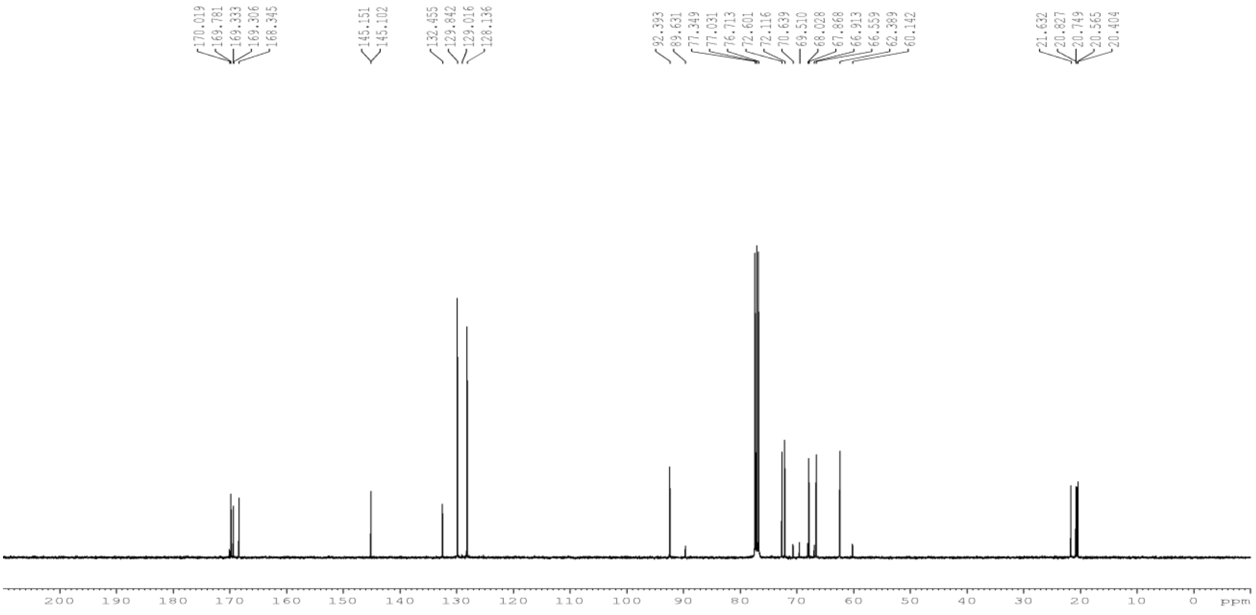

Figure. $^{13}C\{^{1}H\}$ NMR (100 MHz, $CDCl_3$) spectrum of **S12.**

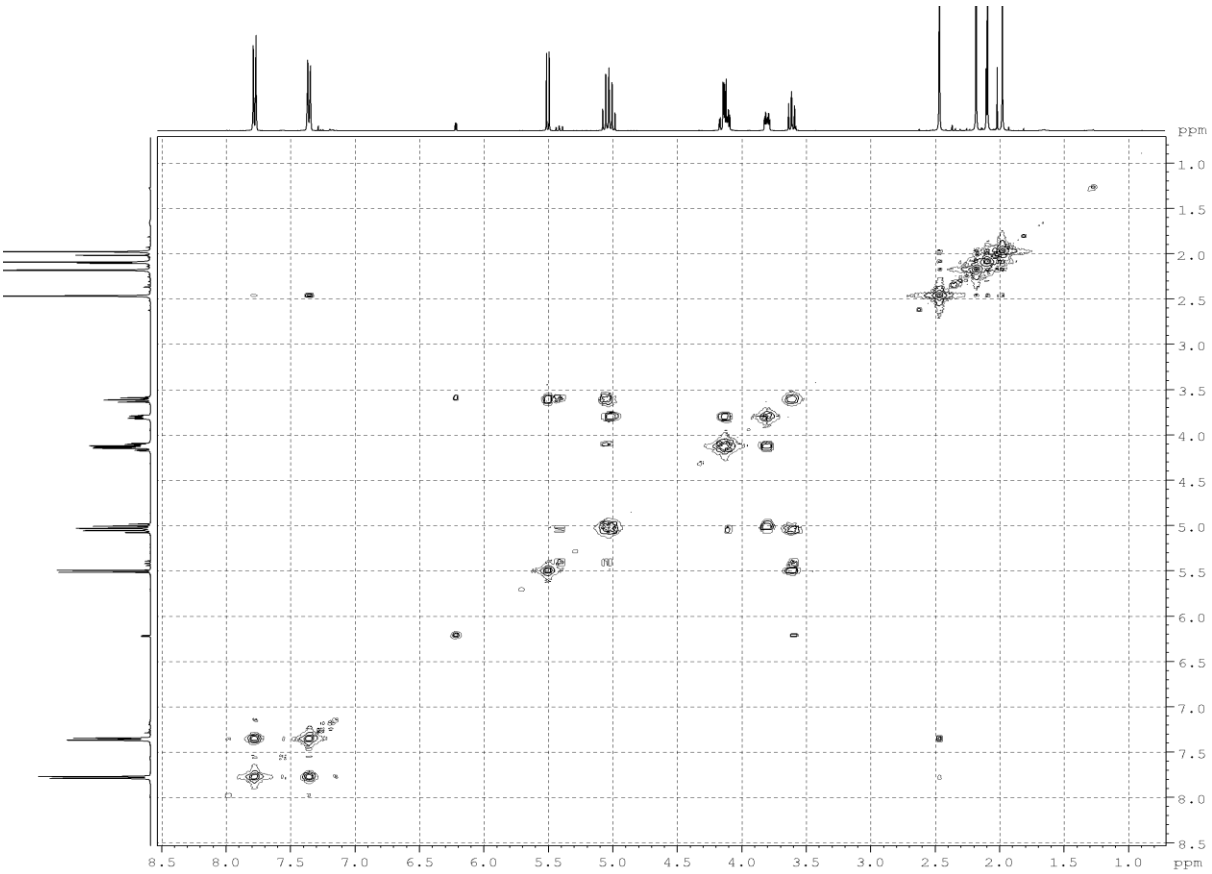

Figure. ^{1}H-^{1}H COSY NMR (400 MHz, $CDCl_3$) spectrum of **S12.**

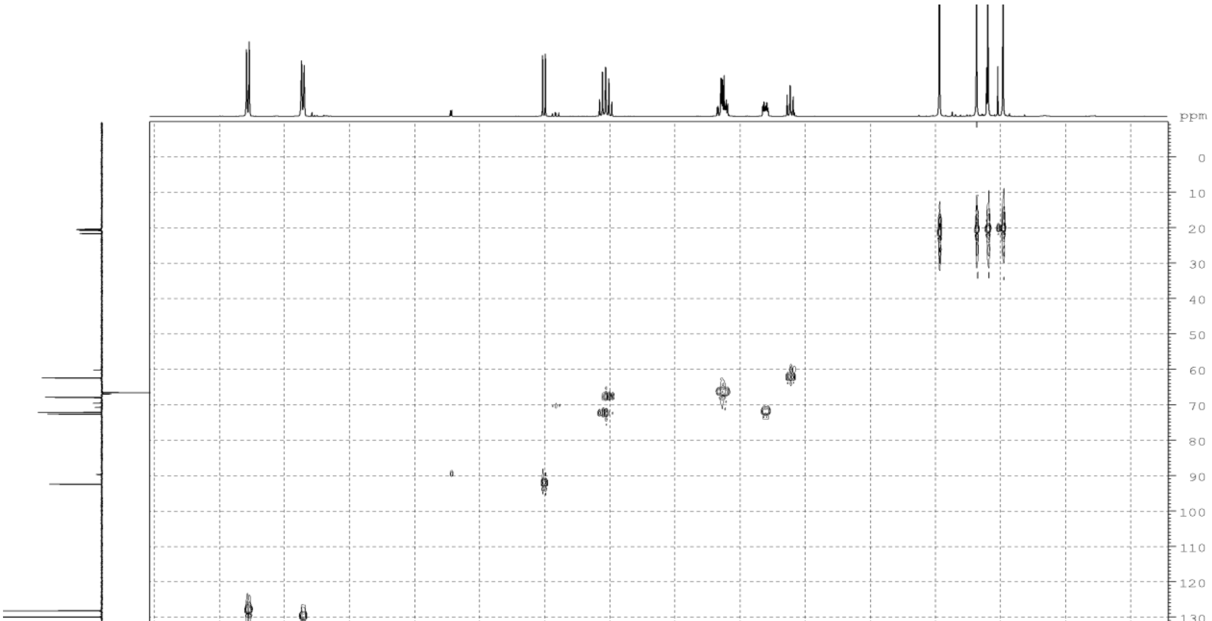

Figure. DEPT-HSQC NMR spectrum of **S12**.

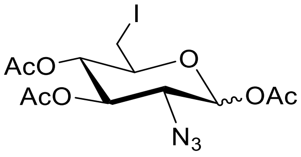

S13

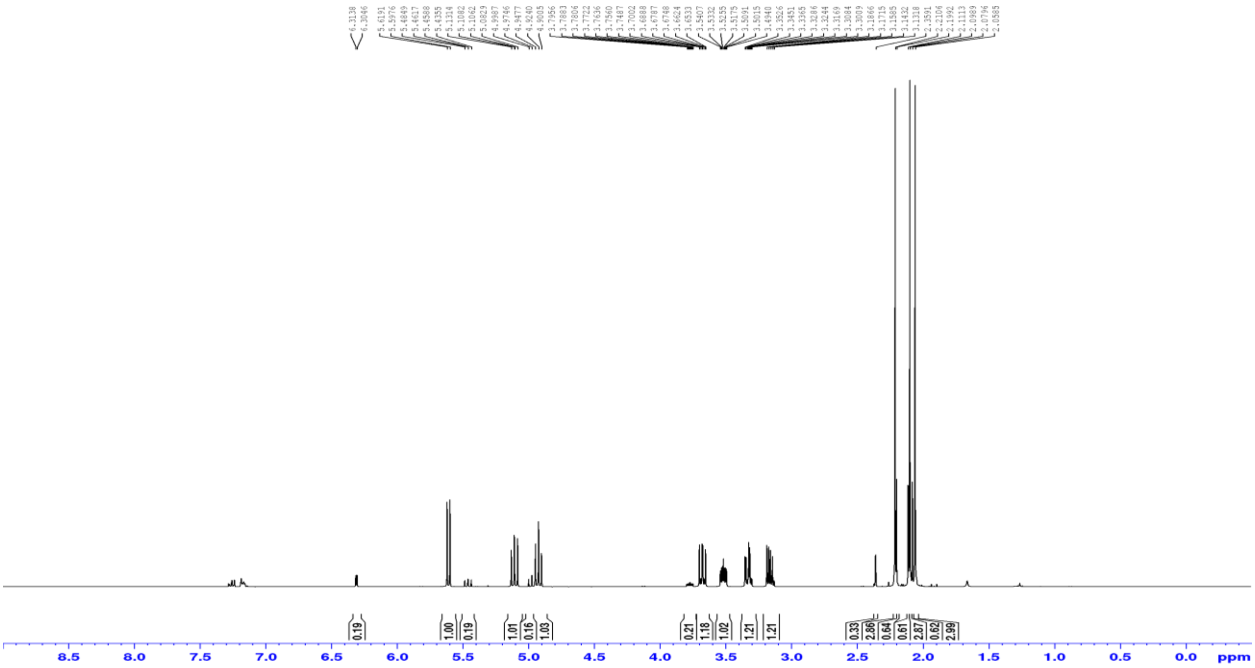

Figure. ^{1}H NMR (400 MHz, $CDCl_3$) spectrum of **S13**.

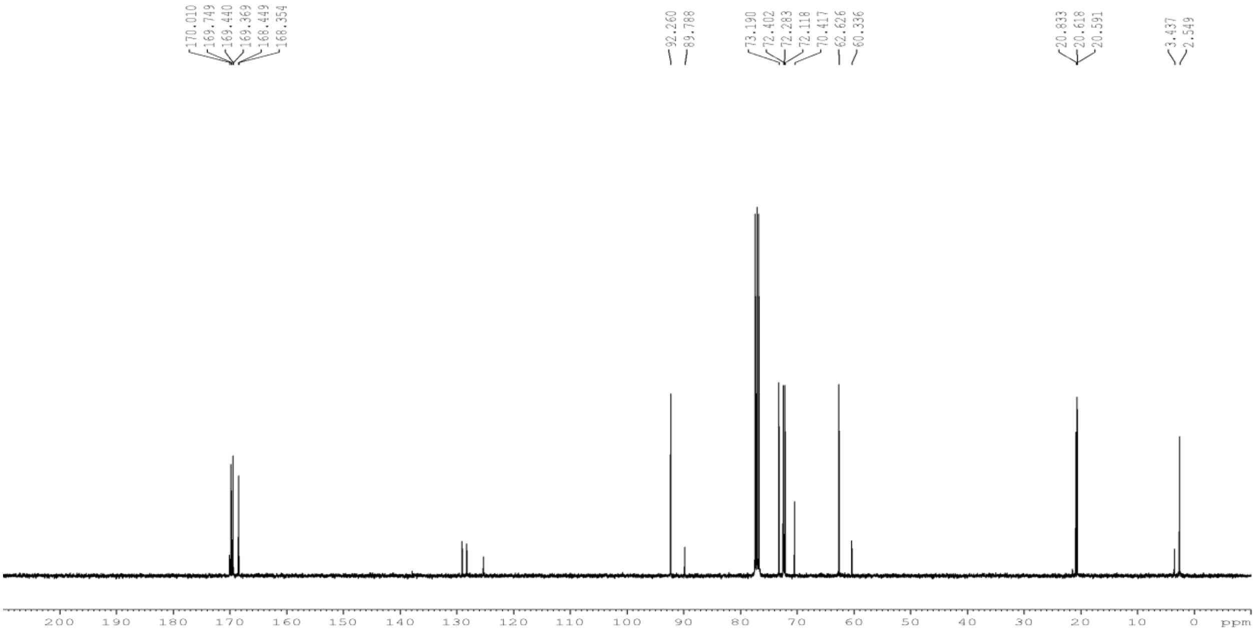

Figure. $^{13}C\{^1H\}$ NMR (100 MHz, $CDCl_3$) spectrum of **S13**.

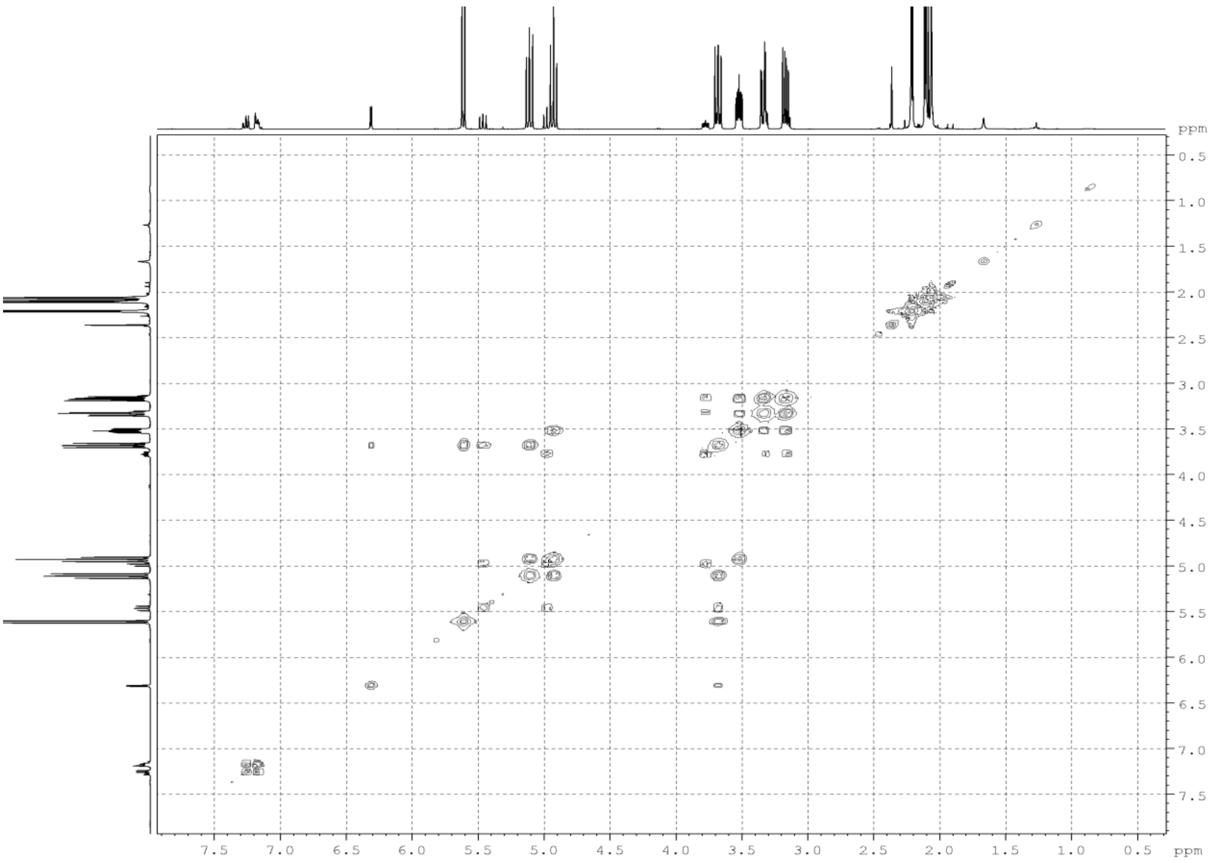

Figure. ^{1}H-^{1}H COSY NMR (400 MHz, $CDCl_3$) spectrum of **S13.**

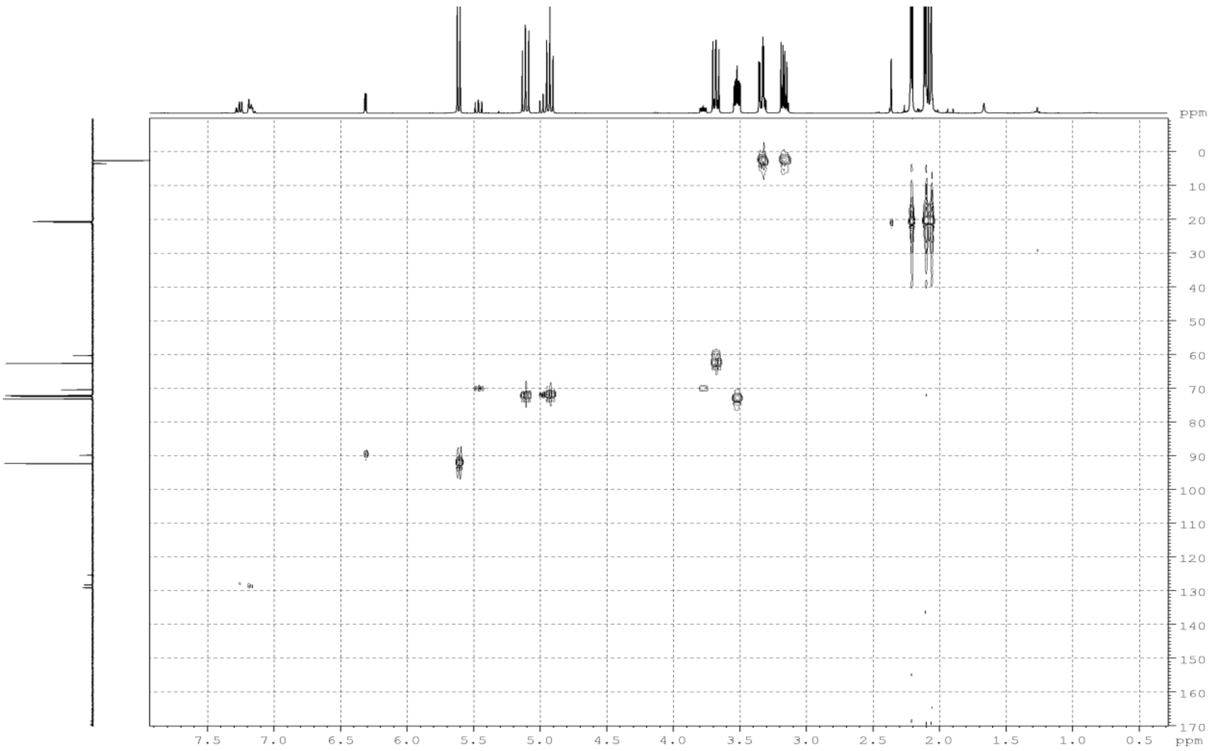

Figure. DEPT-HSQC NMR spectrum of **S13**.

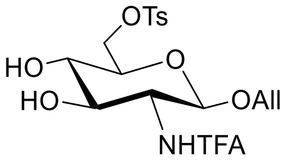

S14

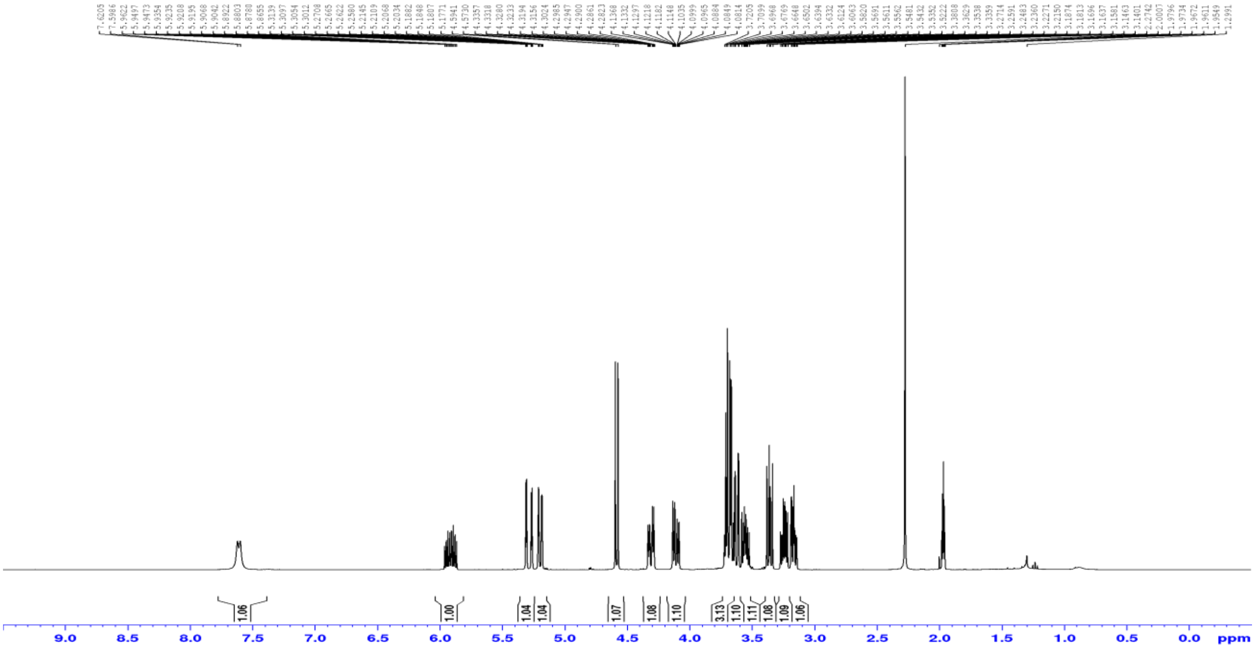

Figure. ^{1}H NMR (400 MHz, CD$_3$CN) spectrum of **S14**.

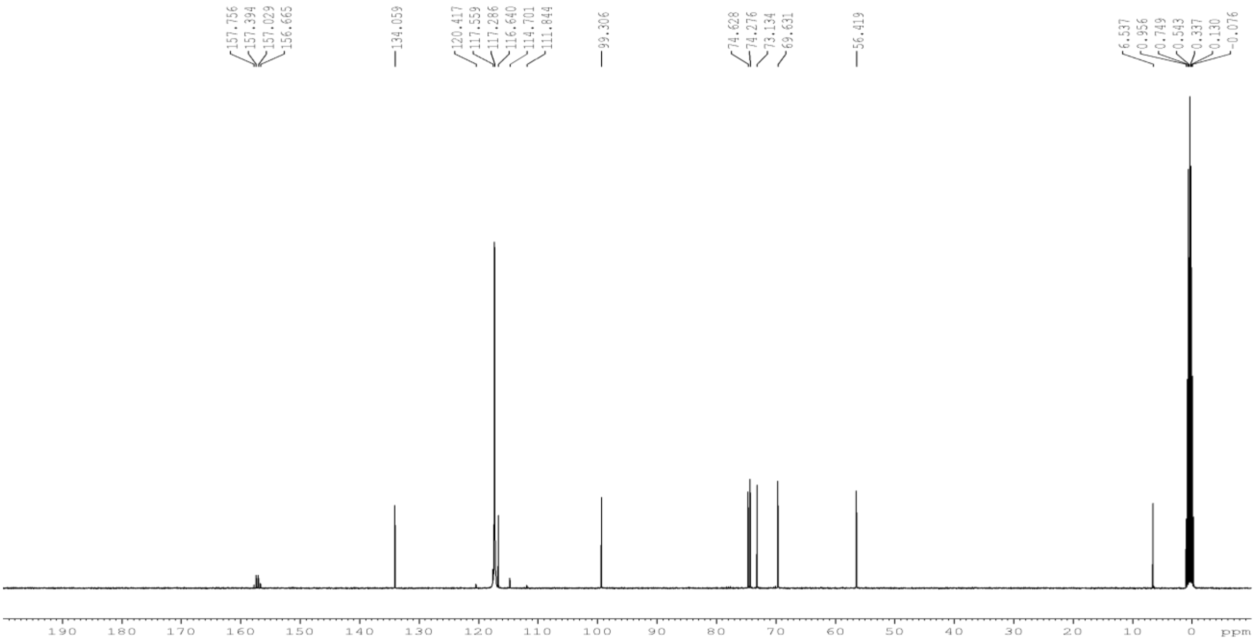

Figure. ^{13}C{^{1}H} NMR (100 MHz, CD$_3$CN) spectrum of **S14**.

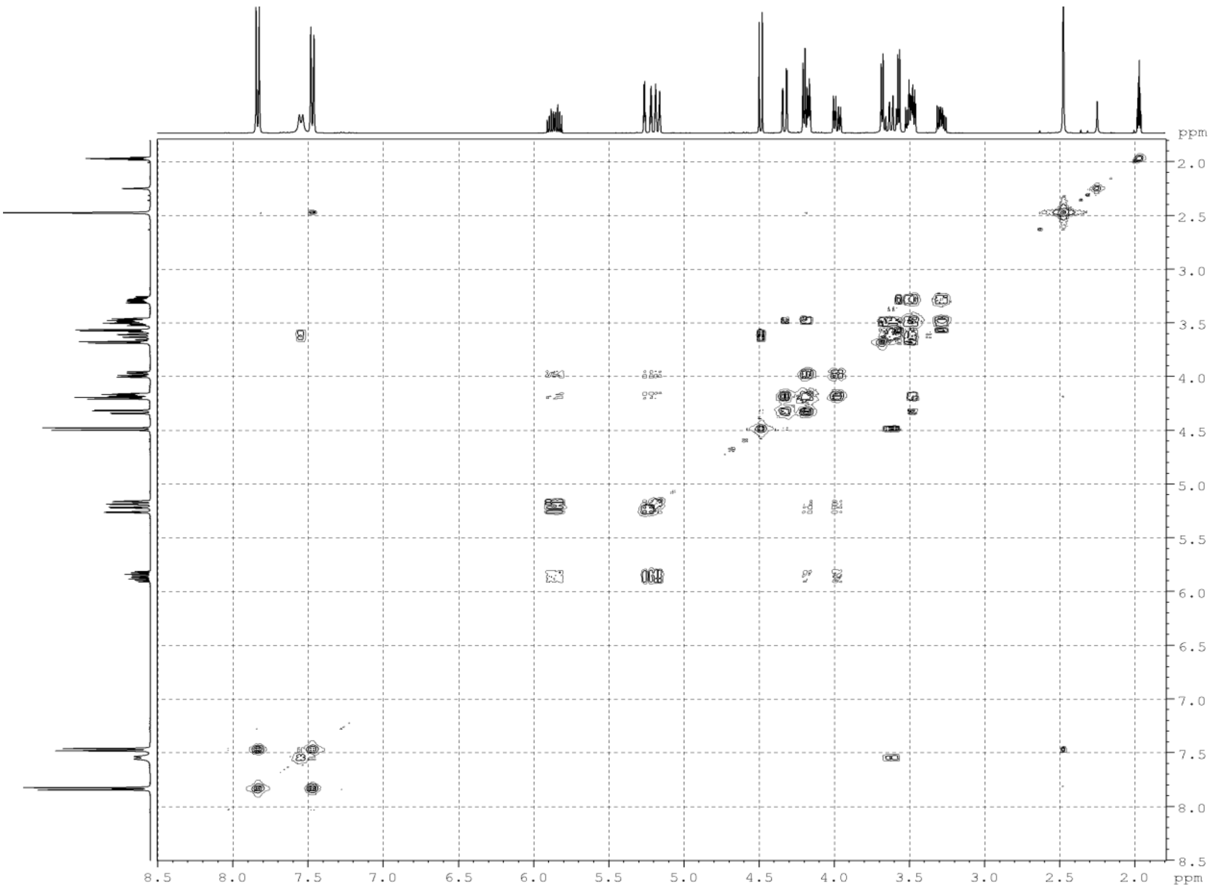

Figure. ^{1}H-^{1}H COSY NMR (400 MHz, CD_3CN) spectrum of **S14.**

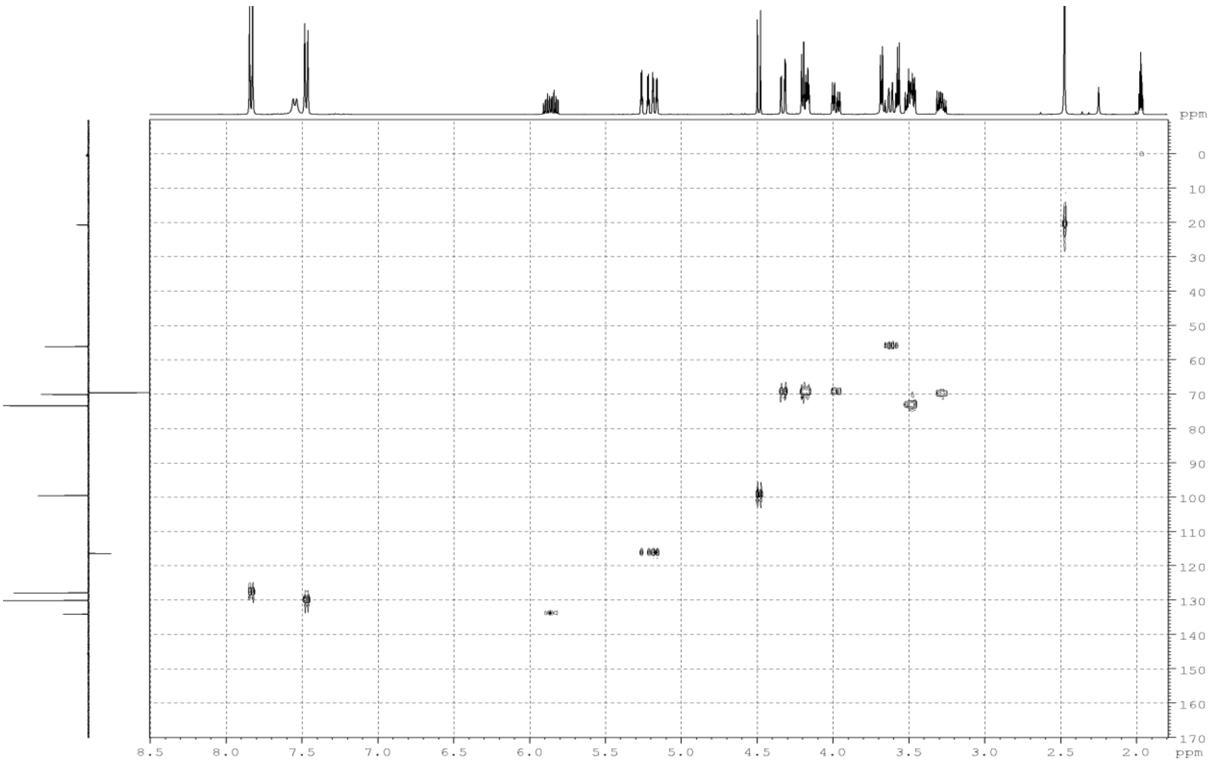

Figure. DEPT-HSQC NMR spectrum of **S14**.

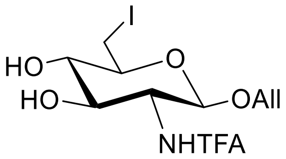

S15

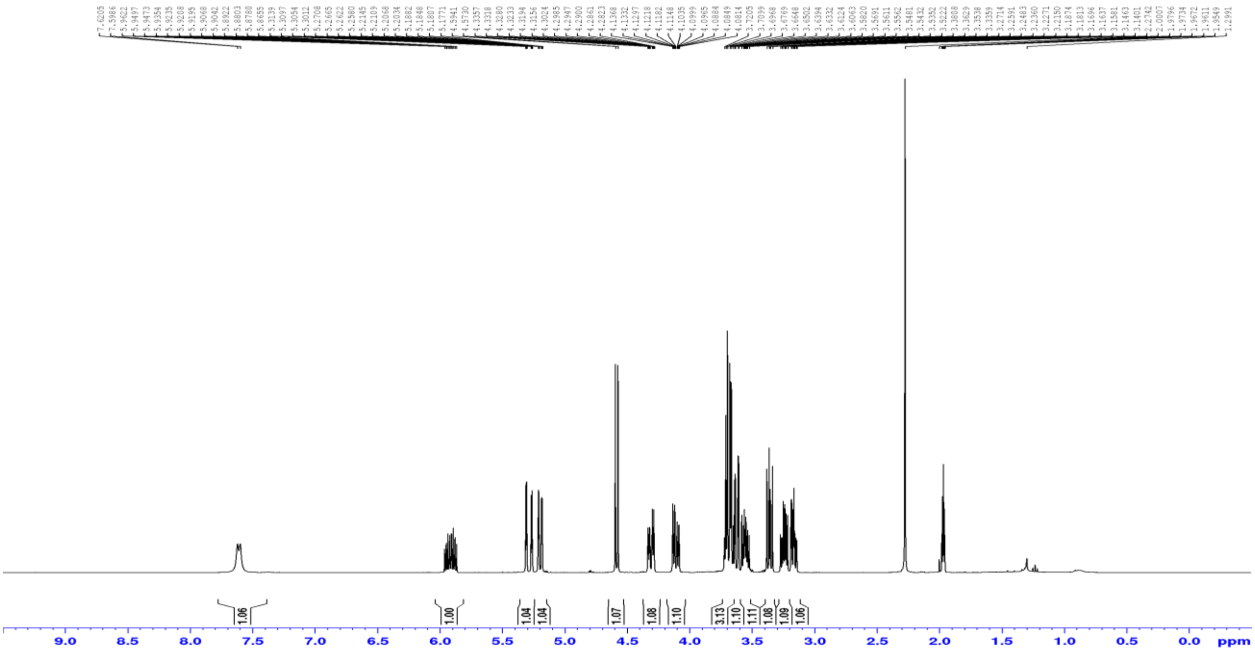

Figure. ^{1}H NMR (400 MHz, CD_3CN) spectrum of **S15**.

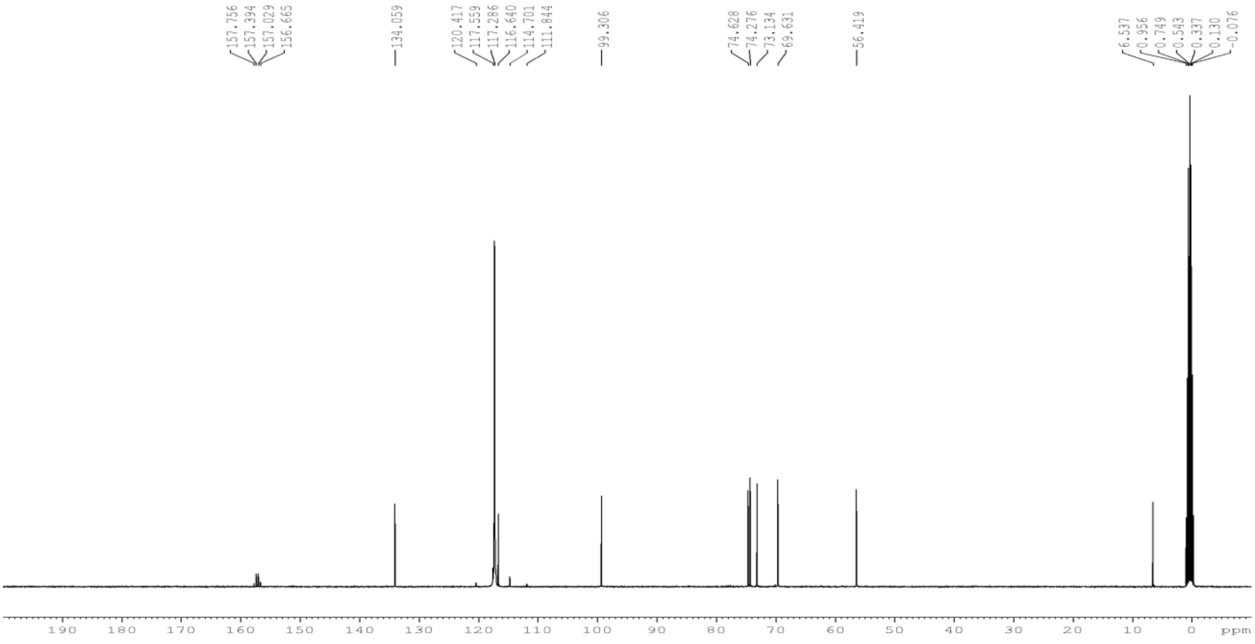

Figure. $^{13}C\{^{1}H\}$ NMR (100 MHz, CD_3CN) spectrum of **S15**.

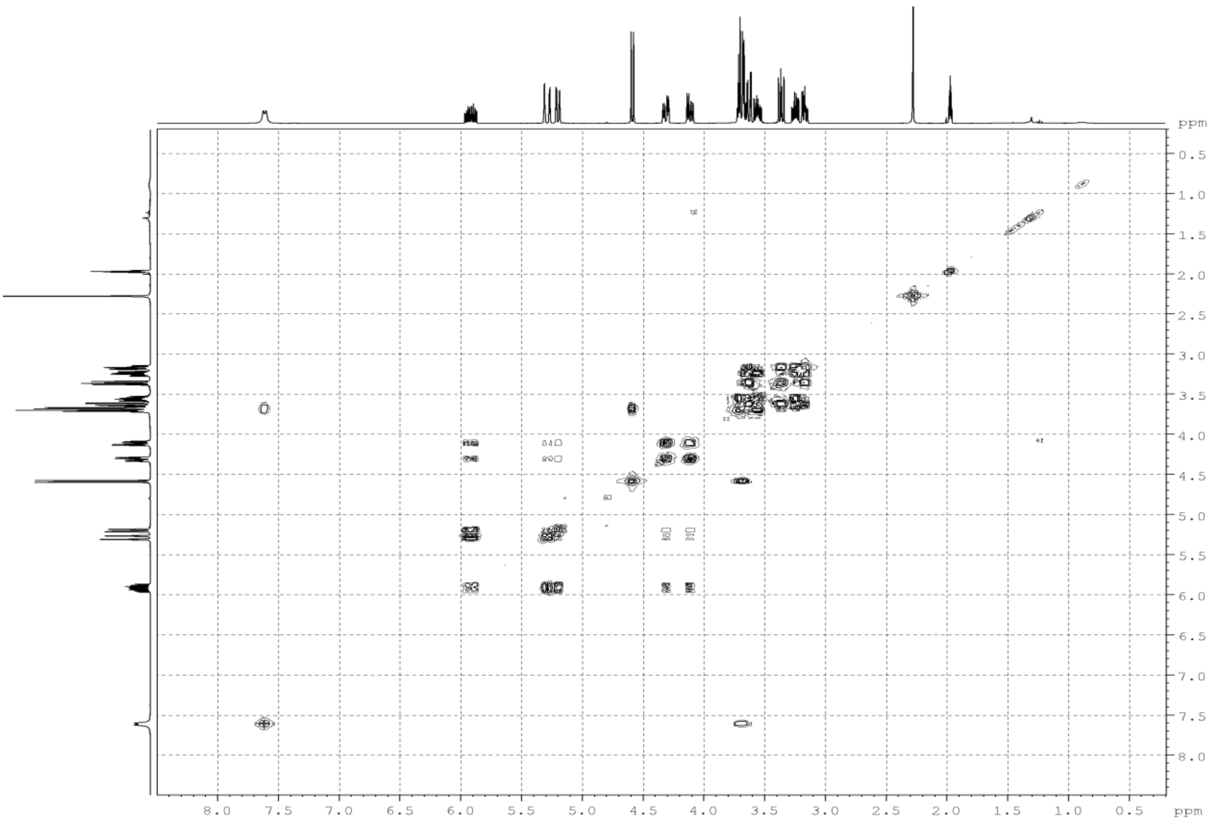

Figure. ^{1}H-^{1}H COSY NMR (400 MHz, CD_3CN) spectrum of **S15.**

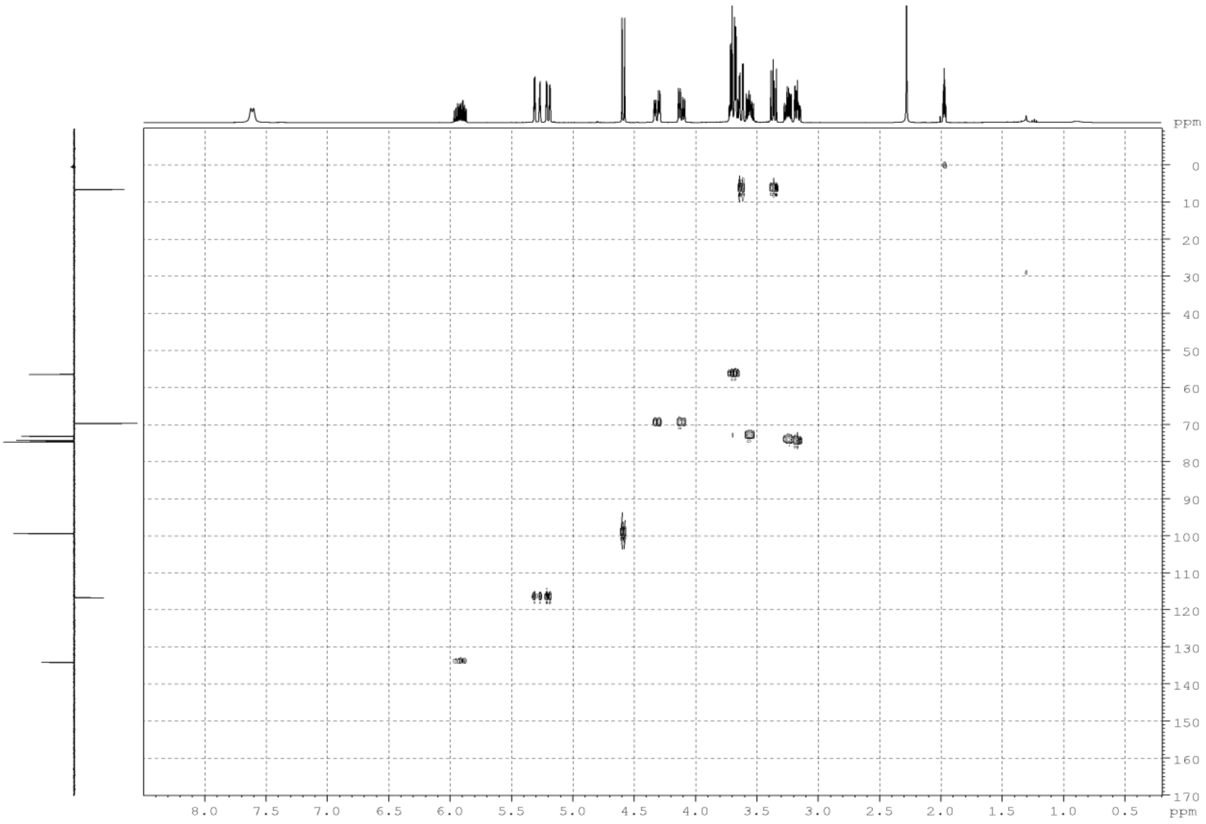

Figure. DEPT-HSQC NMR spectrum of **S15**.

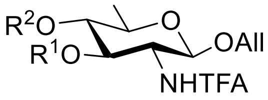

S16 R[1],R[2]: H, Ac
32 R[1],R[2]: Ac, H

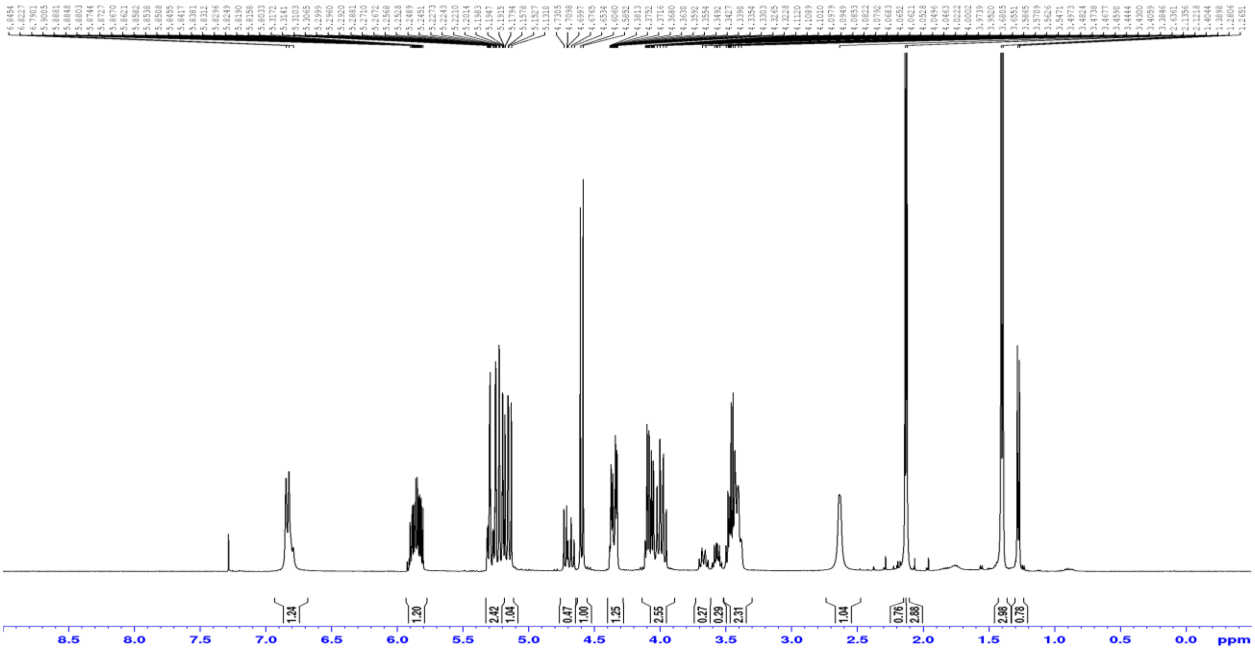

Figure. ^{1}H NMR (400 MHz, CDCl3) spectrum of **S16 + 32 (S16:32 ~1:4).**

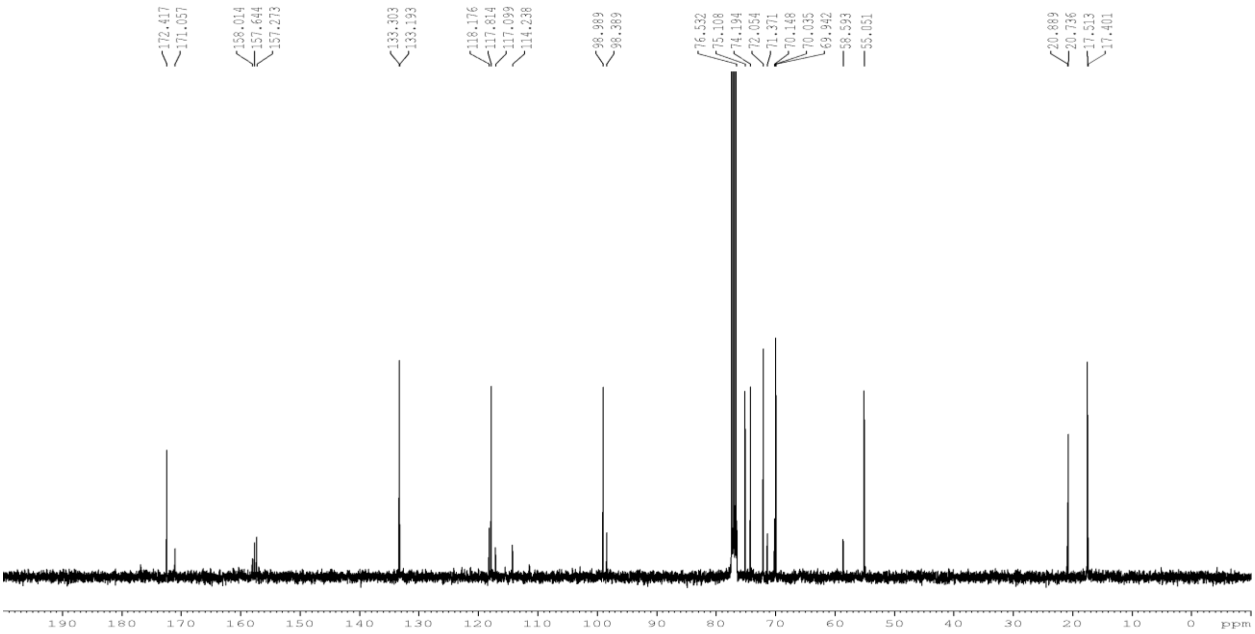

Figure. ^{13}C{^{1}H} NMR (100 MHz, CDCl3) spectrum of **S16 + 32 (S16:32 ~1:4)**.

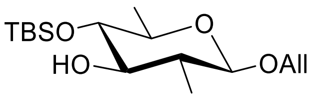

S17

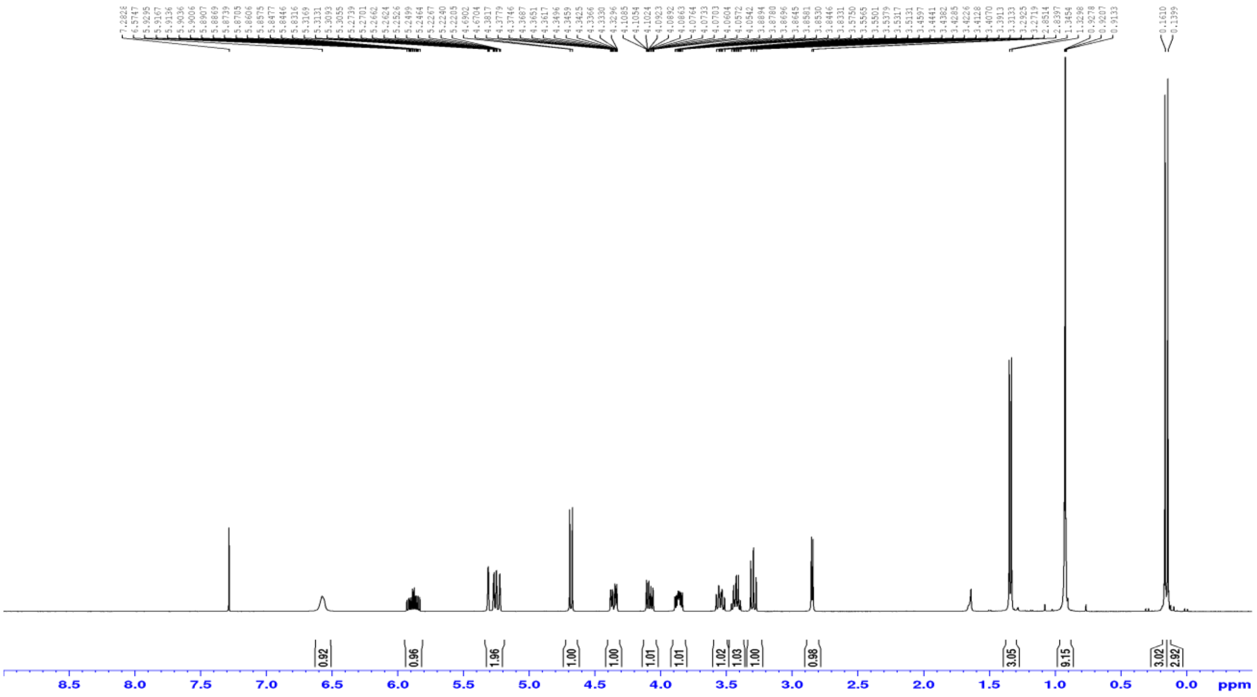

Figure. ^{1}H NMR (400 MHz, $CDCl_3$) spectrum of **S17**.

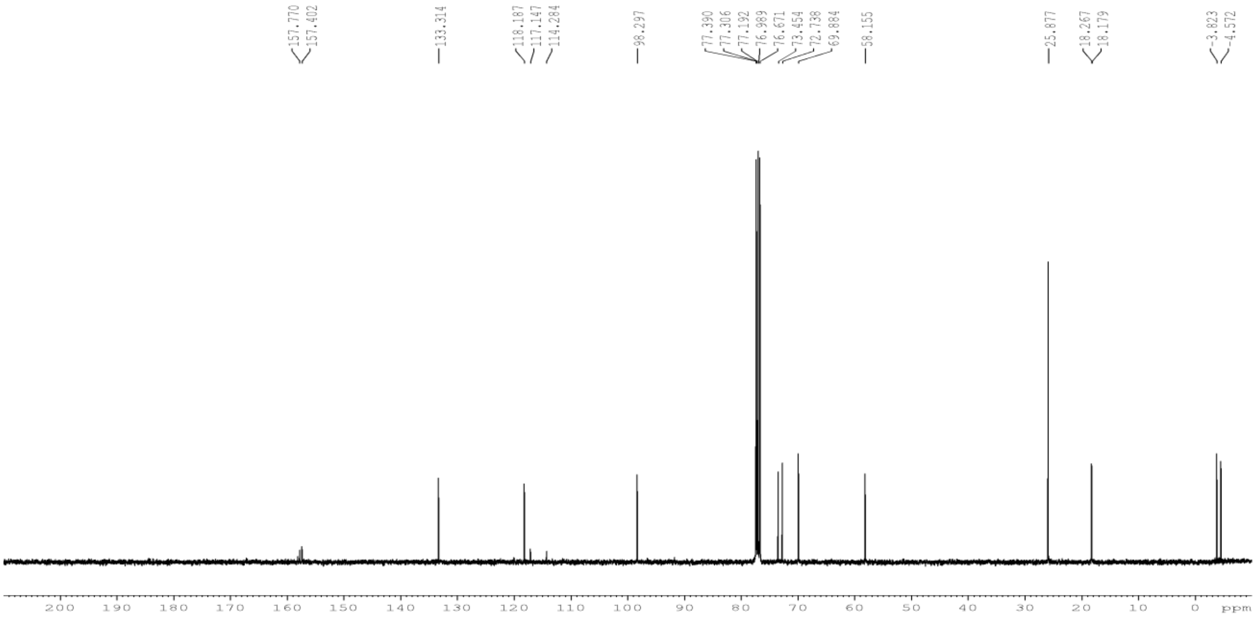

Figure. ^{13}C{^{1}H} NMR (100 MHz, $CDCl_3$) spectrum of **S17**.

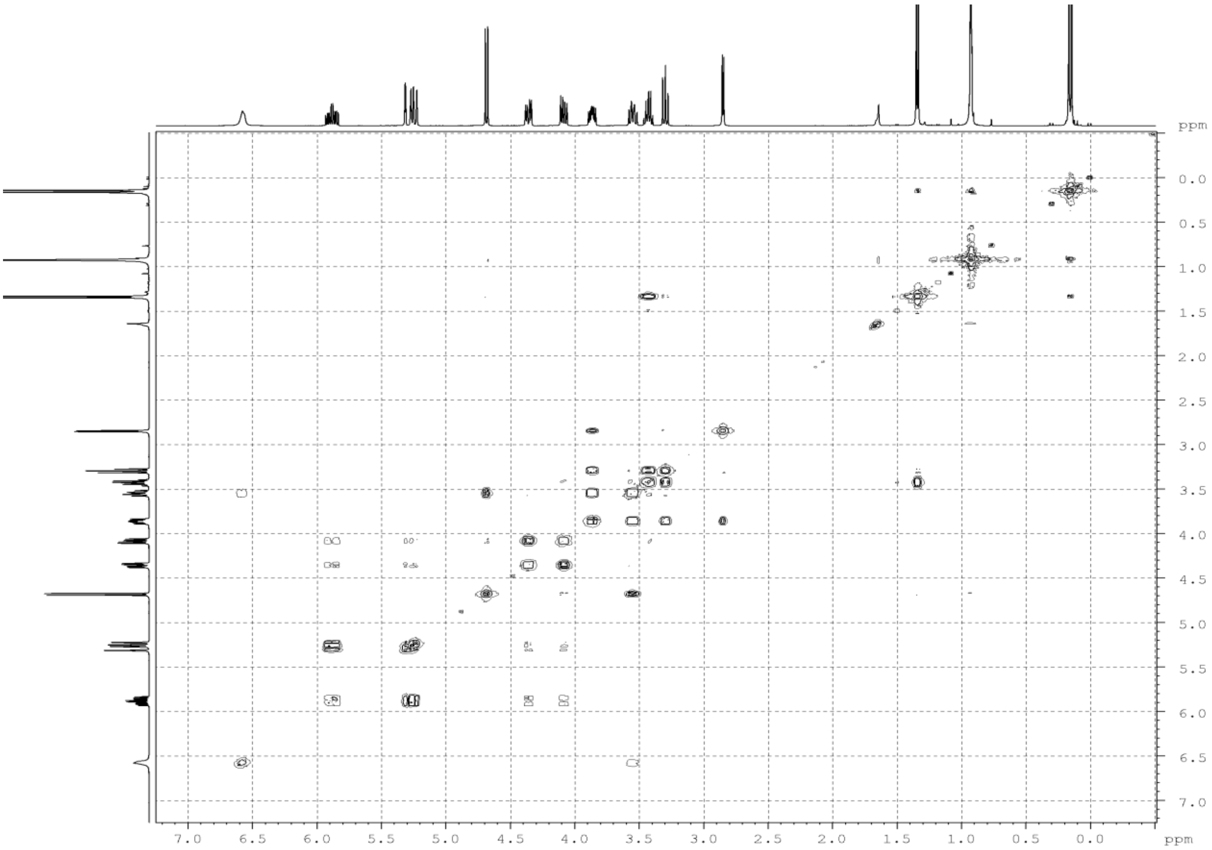

Figure. ^{1}H-^{1}H COSY NMR (400 MHz, $CDCl_3$) spectrum of **S17.**

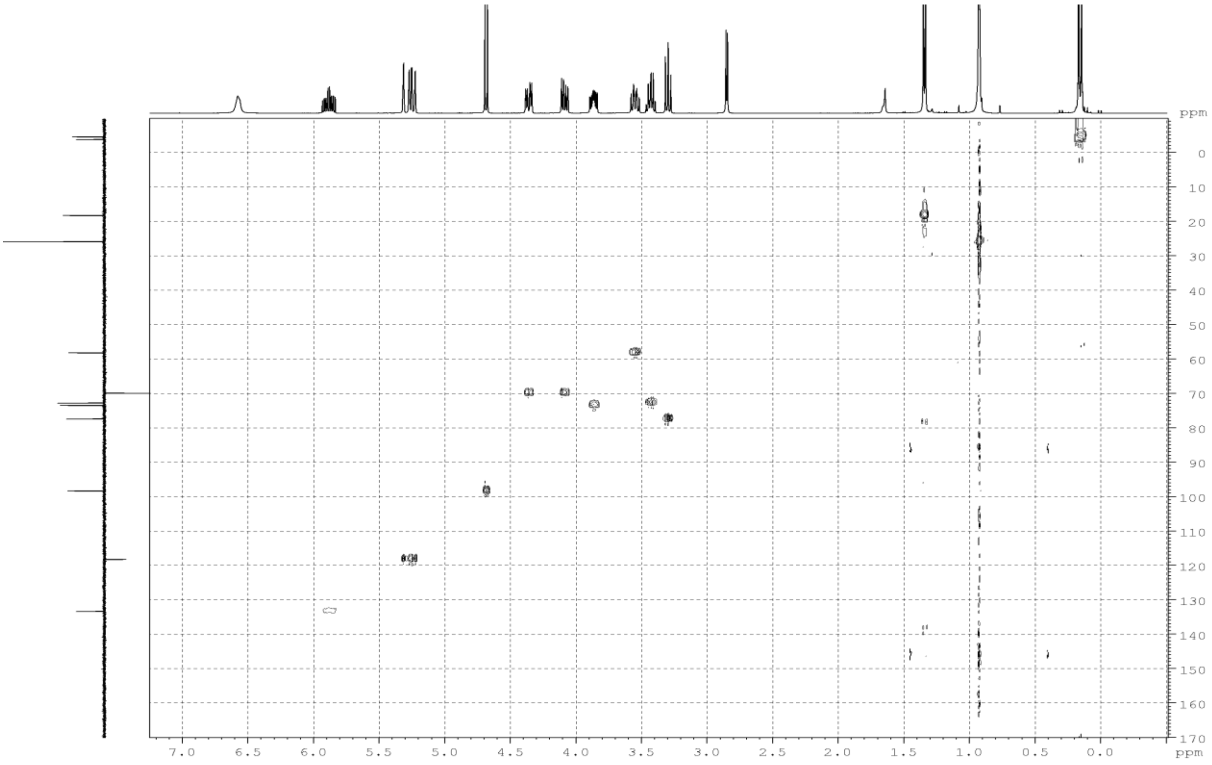

Figure. DEPT-HSQC NMR spectrum of **S17**.

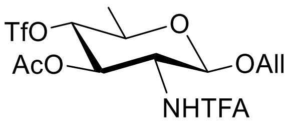

S18

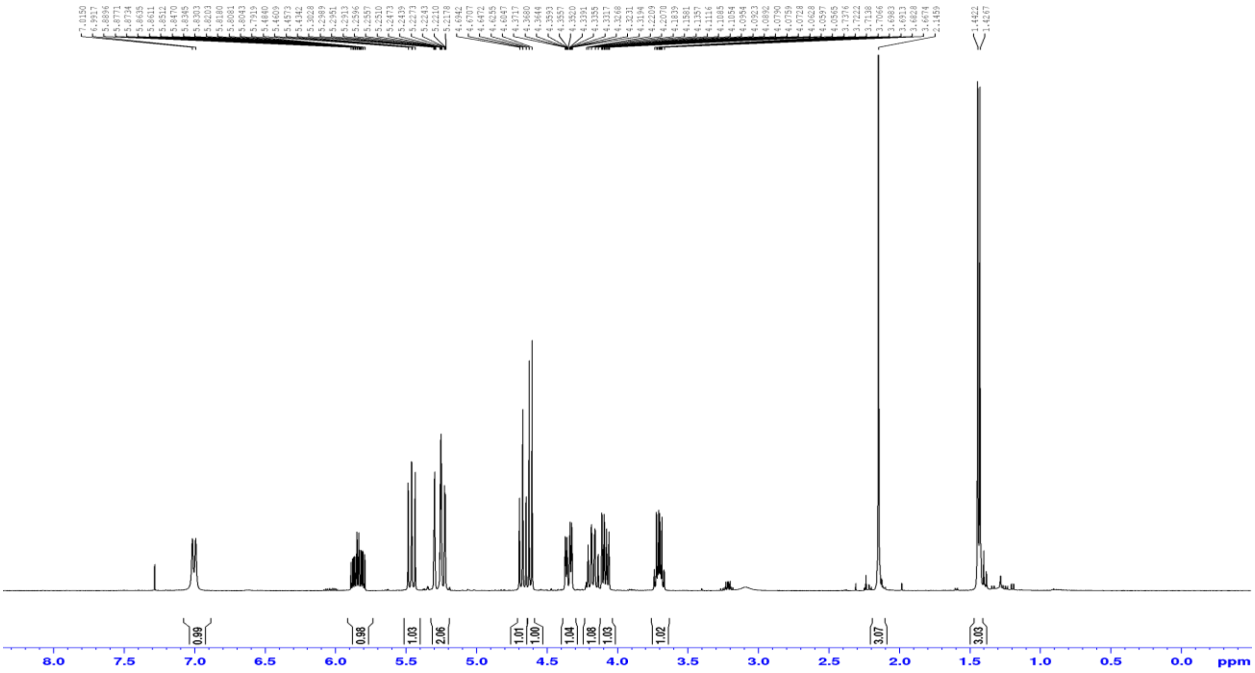

Figure. ^{1}H NMR (400 MHz, $CDCl_3$) spectrum of **S18**.

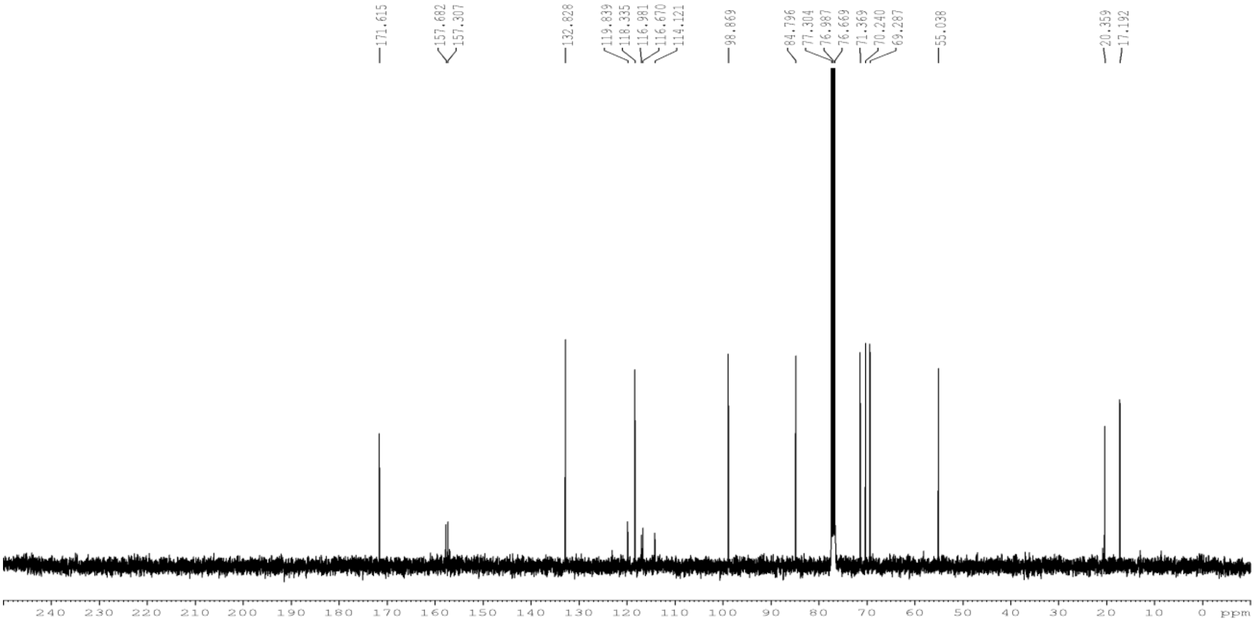

Figure. ^{13}C{^{1}H} NMR (100 MHz, $CDCl_3$) spectrum of **S18**.

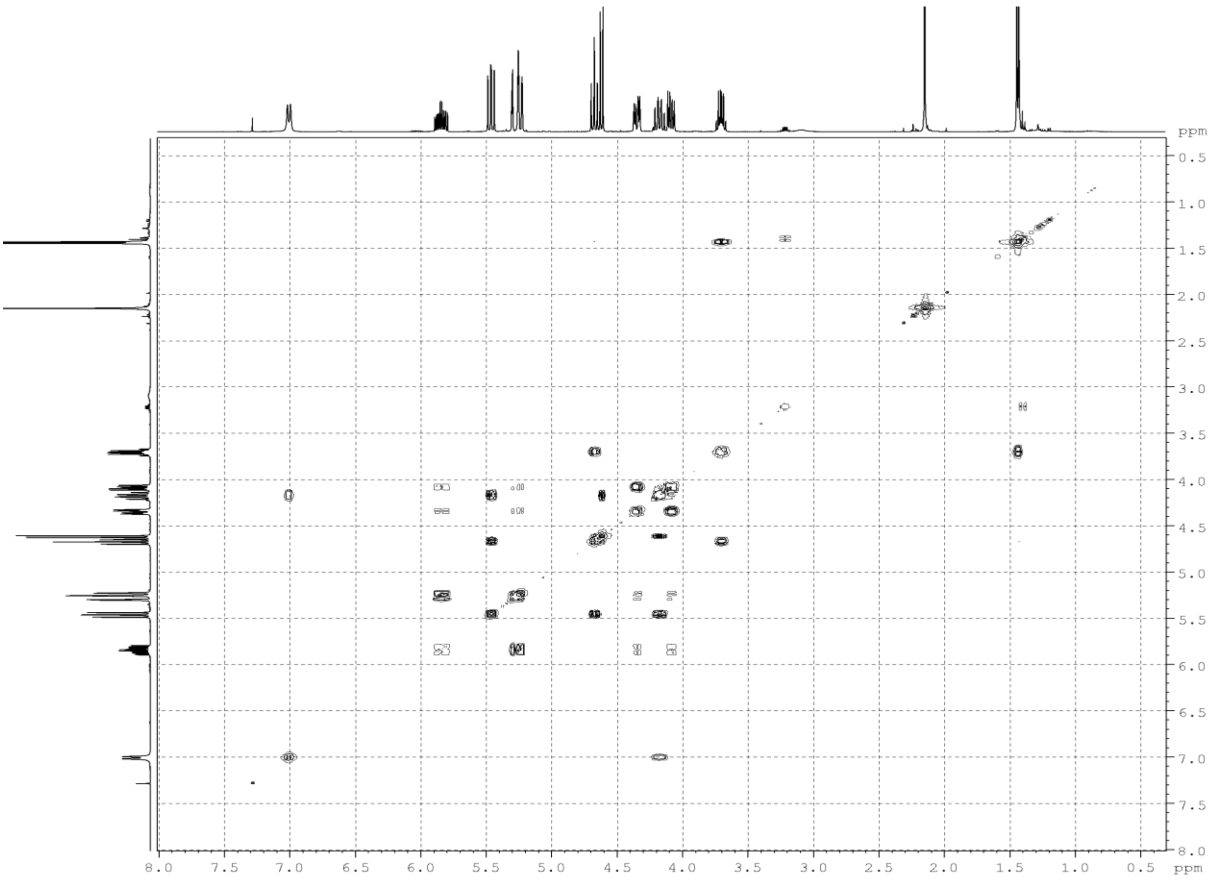

Figure. ^{1}H-^{1}H COSY NMR (400 MHz, $CDCl_3$) spectrum of **S18.**

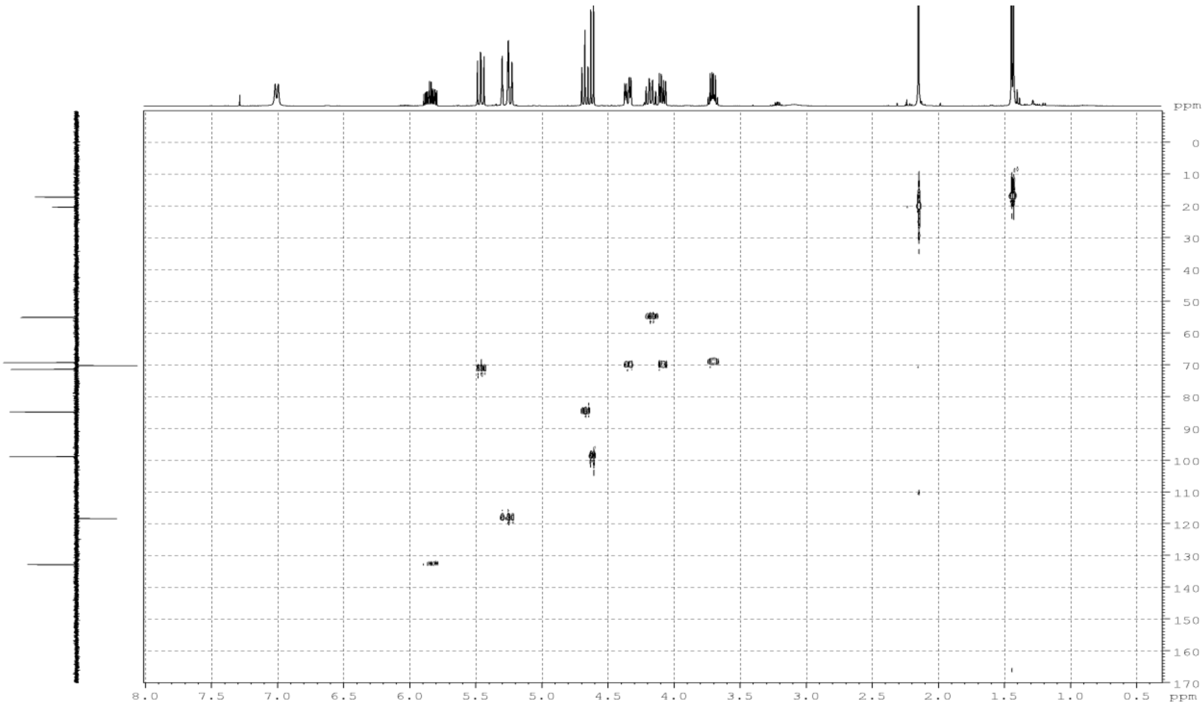

Figure. DEPT-HSQC NMR spectrum of **S18**.

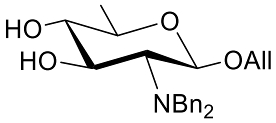

S19

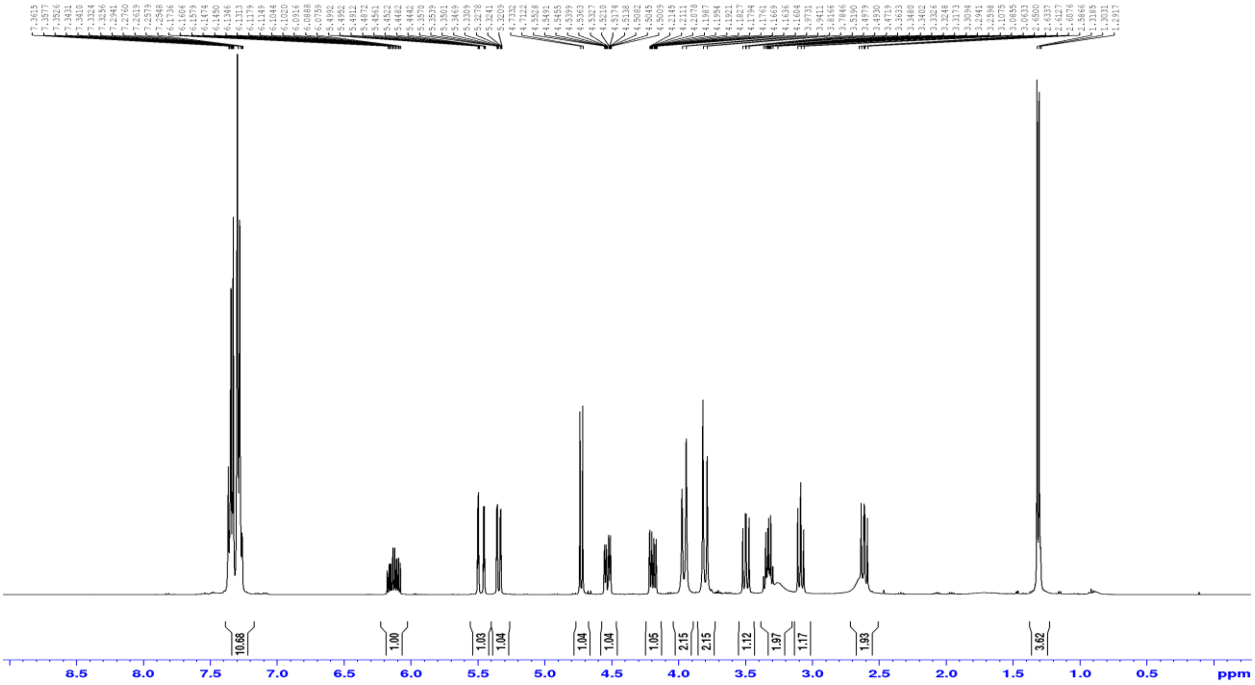

Figure. ^{1}H NMR (400 MHz, $CDCl_3$) spectrum of **S19**.

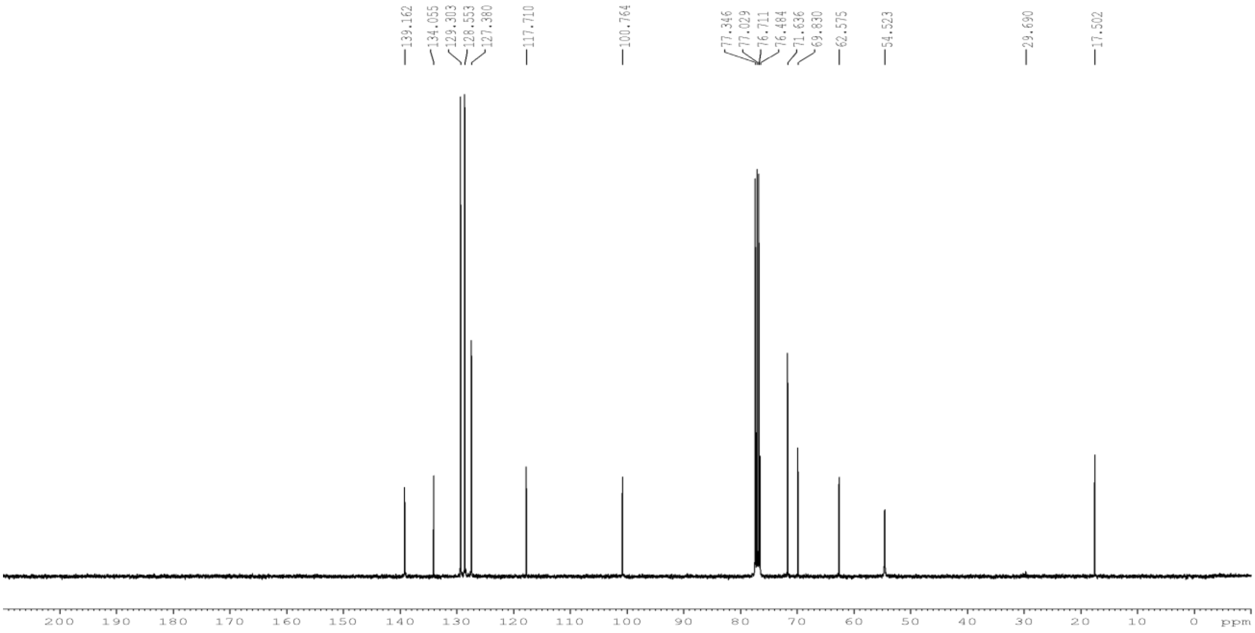

Figure. $^{13}C\{^{1}H\}$ NMR (100 MHz, $CDCl_3$) spectrum of **S19**.

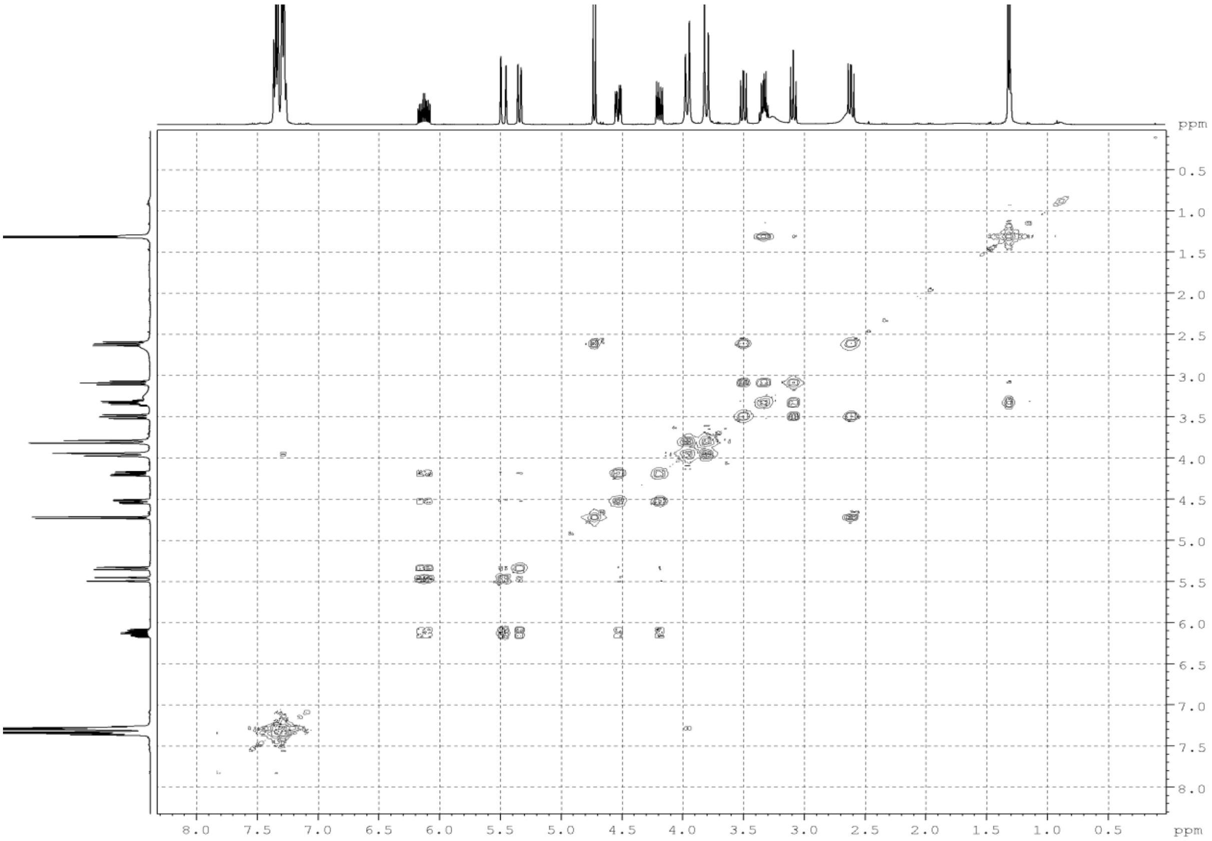

Figure. ^{1}H-^{1}H COSY NMR (400 MHz, $CDCl_3$) spectrum of **S19.**

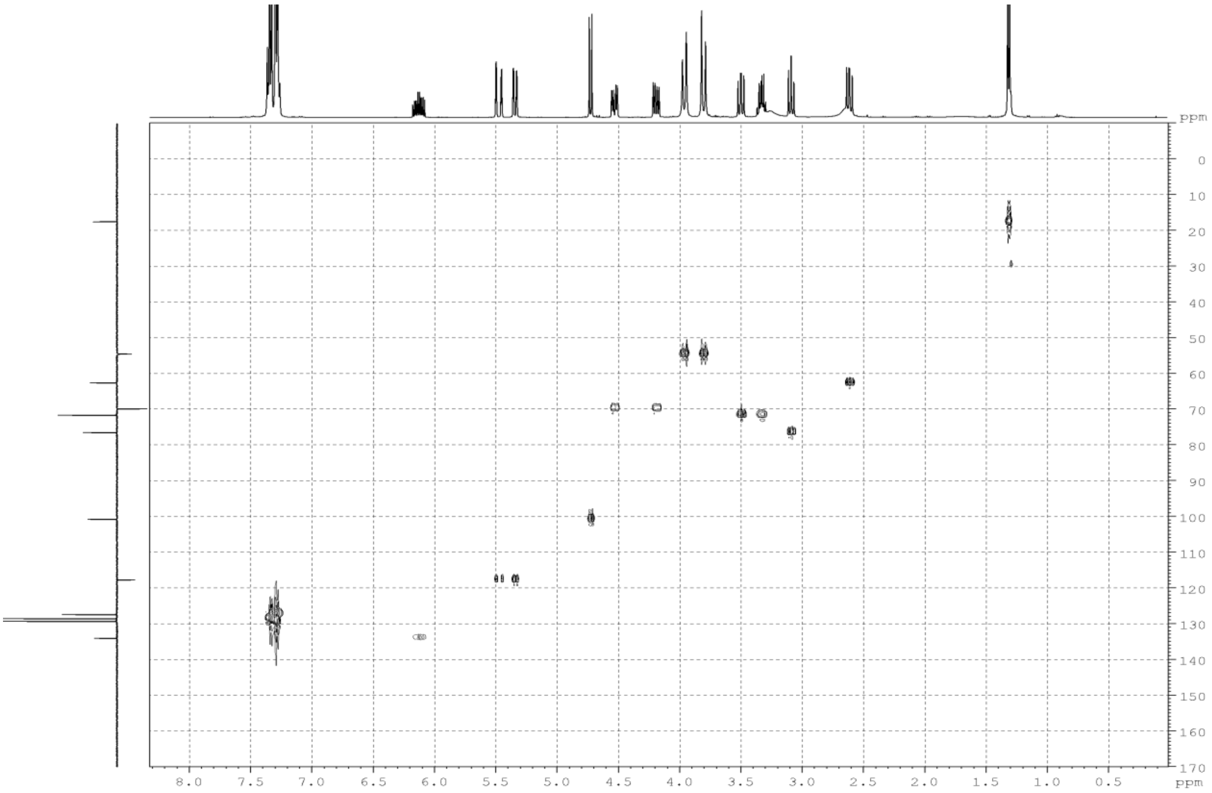

Figure. DEPT-HSQC NMR spectrum of **S19**.

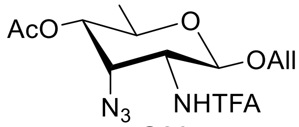

S20

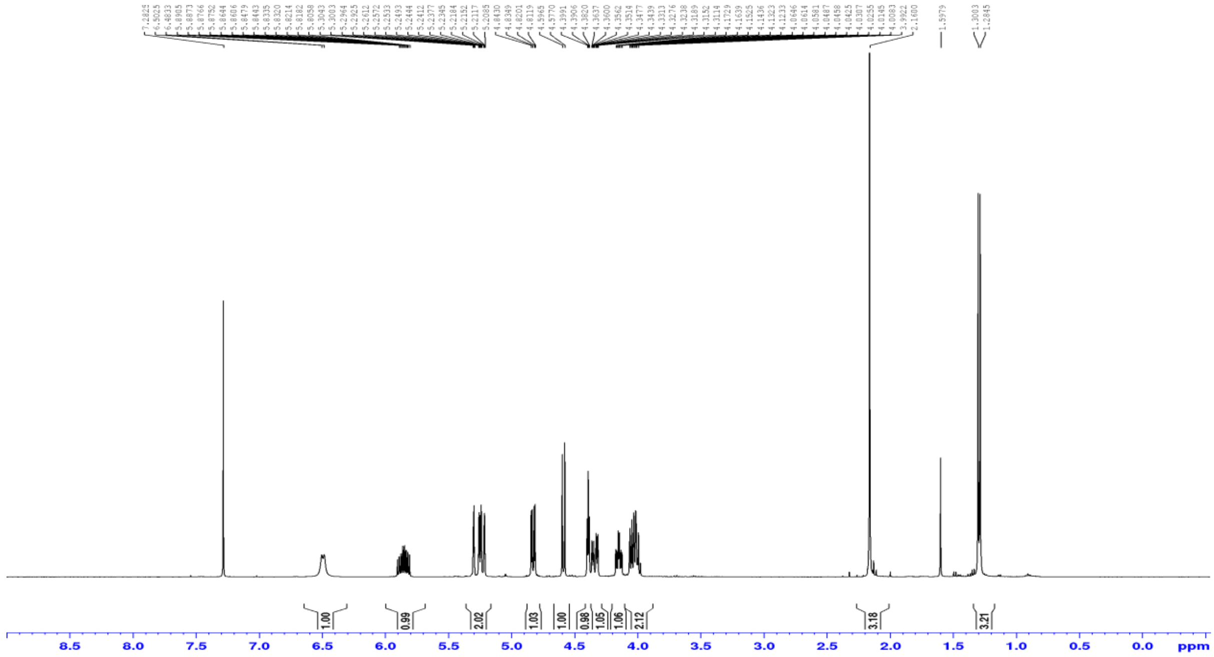

Figure. ^{1}H NMR (400 MHz, $CDCl_3$) spectrum of **S20**.

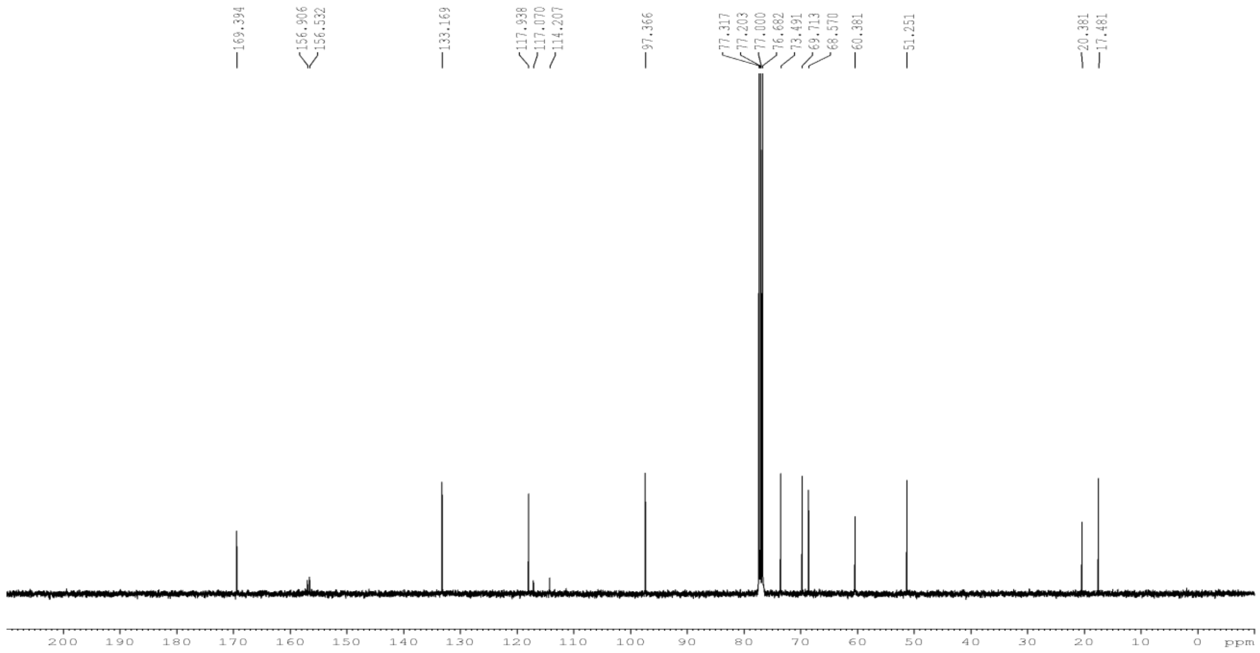

Figure. ^{13}C{^{1}H} NMR (100 MHz, $CDCl_3$) spectrum of **S20**.

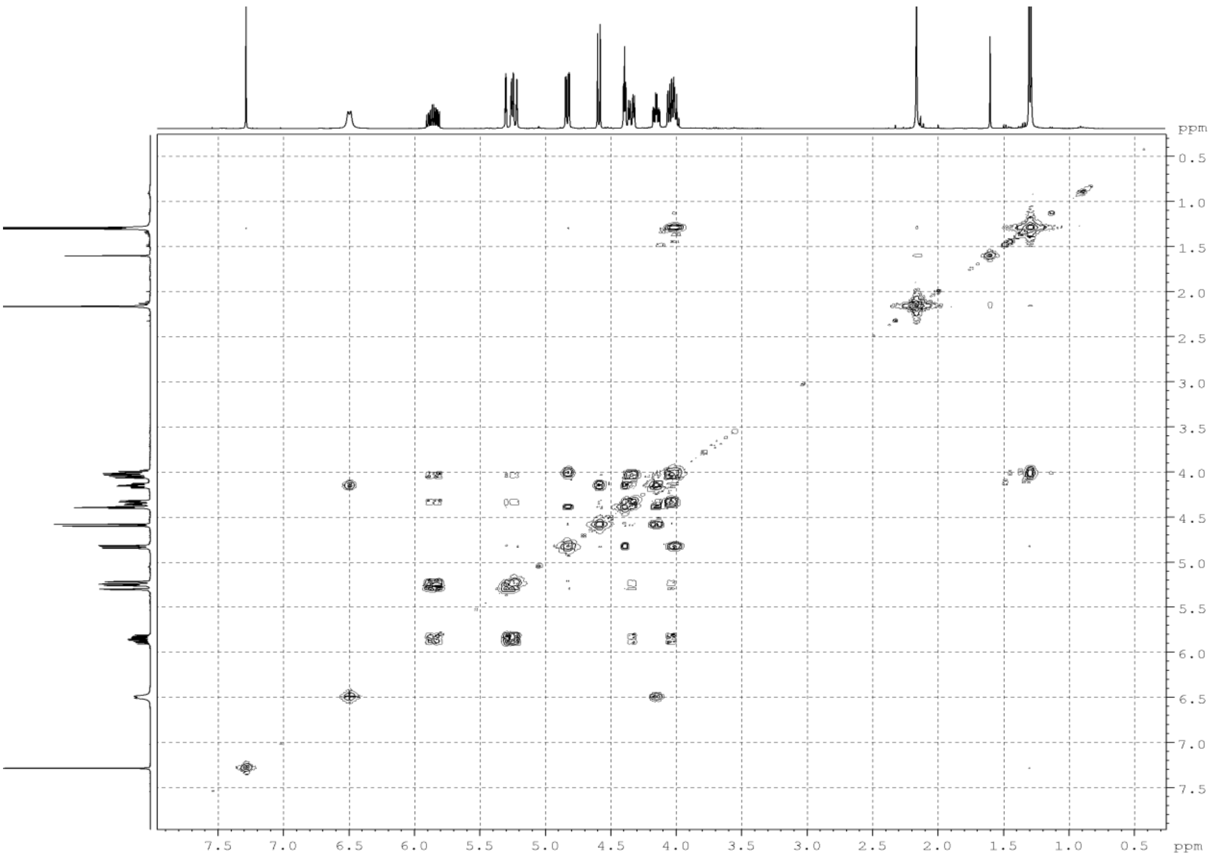

Figure. ^{1}H-^{1}H COSY NMR (400 MHz, $CDCl_3$) spectrum of **S20.**

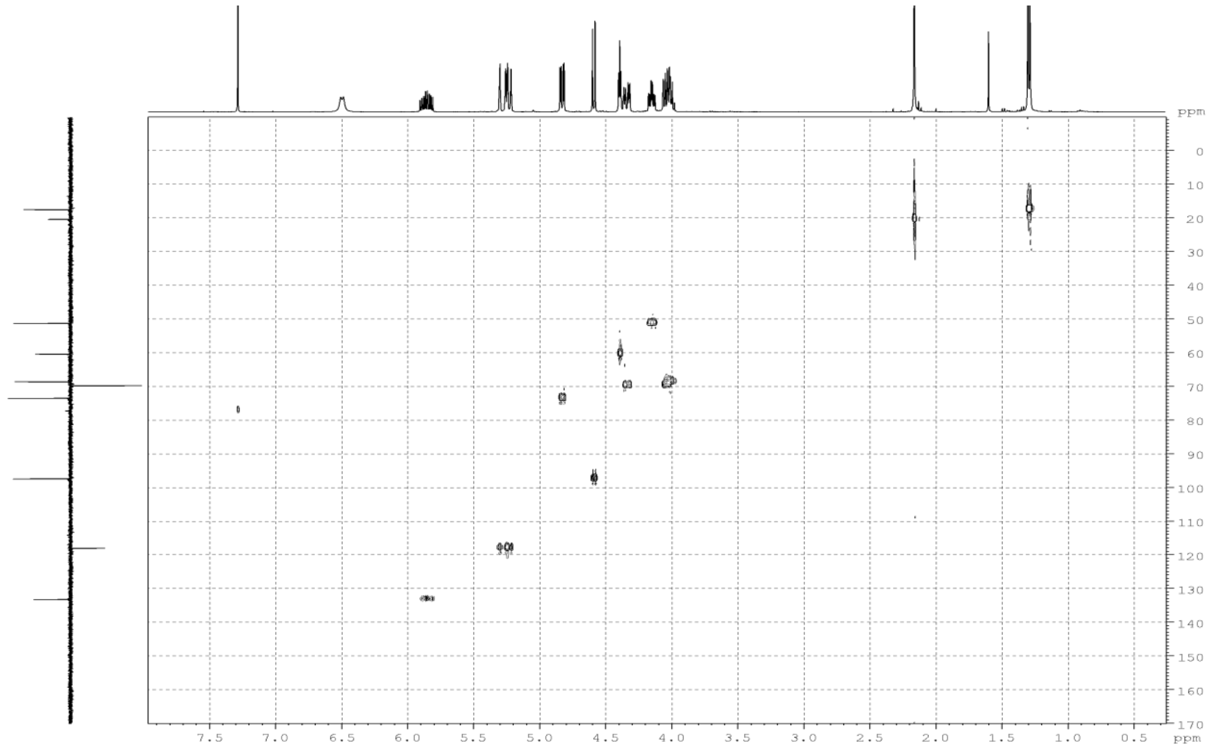

Figure. DEPT-HSQC NMR spectrum of **S20**.

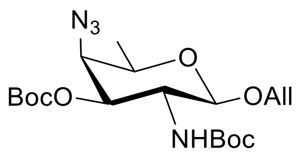

S21

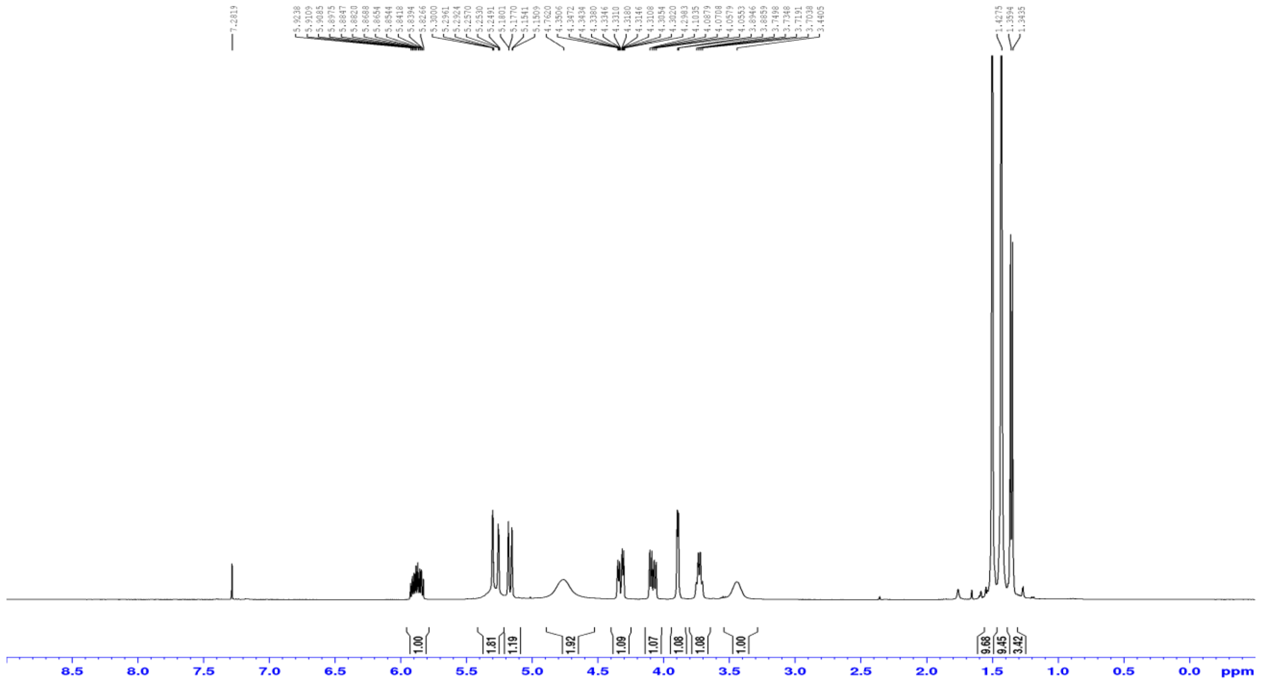

Figure. ^{1}H NMR (400 MHz, $CDCl_3$) spectrum of **S21**.

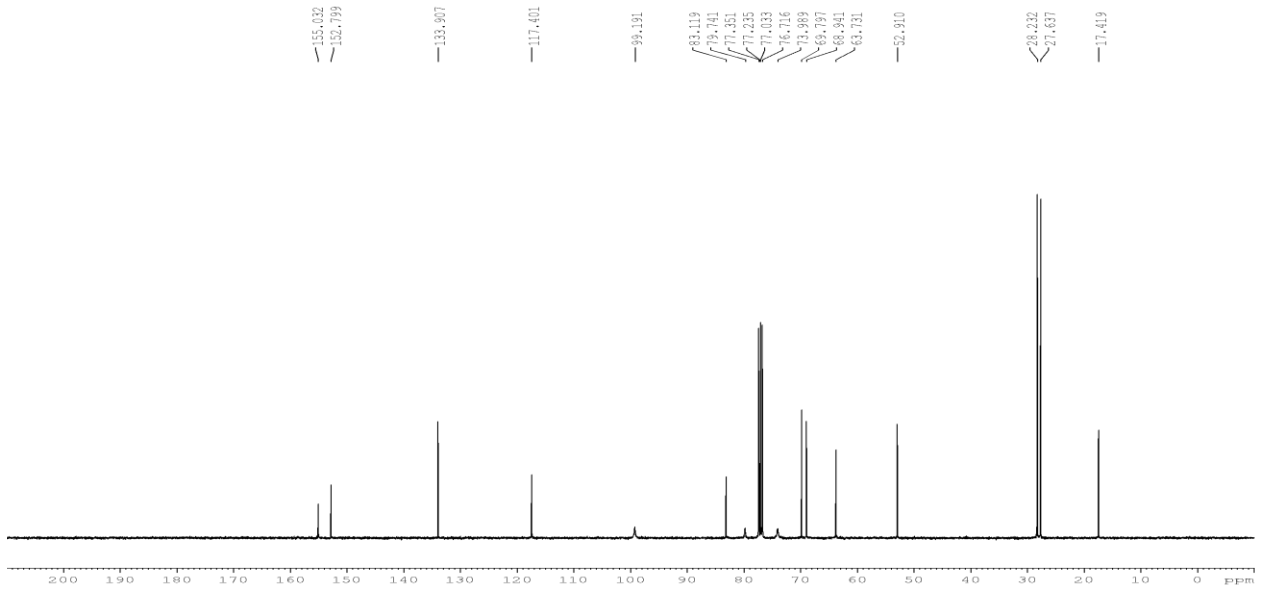

Figure. $^{13}C\{^{1}H\}$ NMR (100 MHz, $CDCl_3$) spectrum of **S21**.

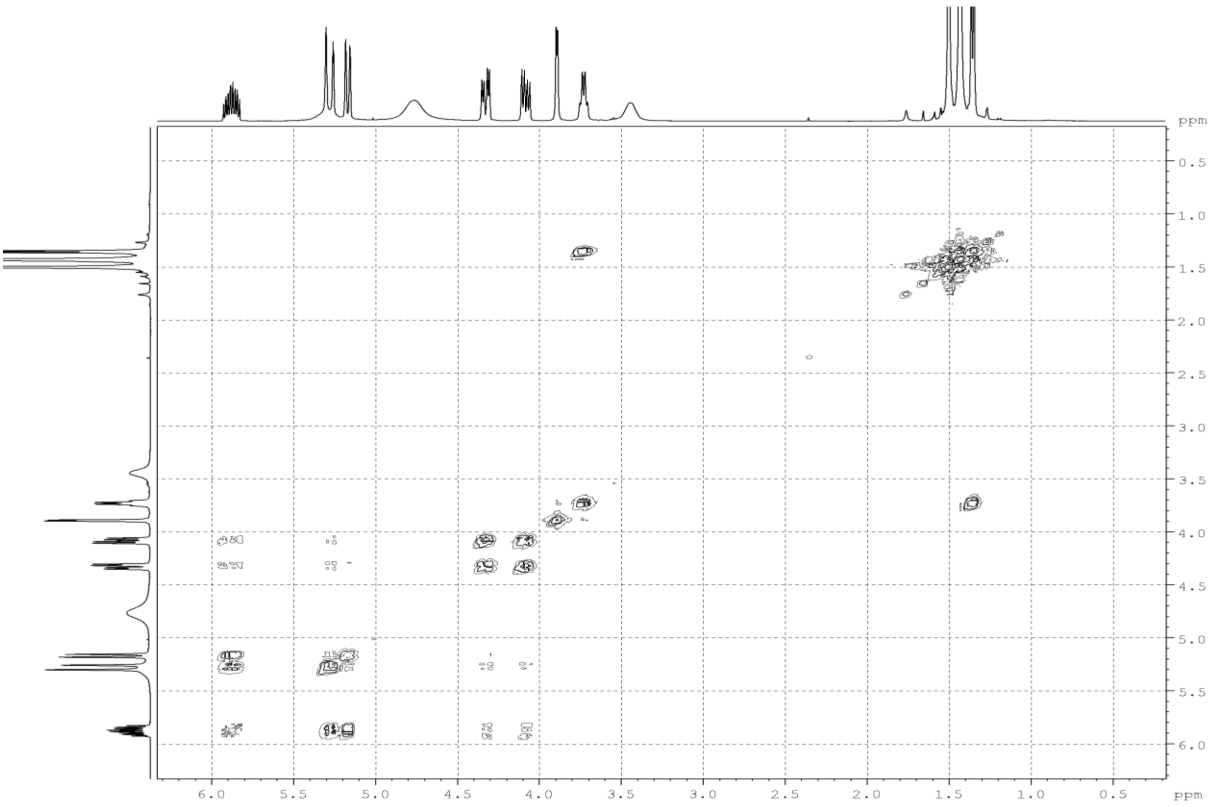

Figure. ^{1}H-^{1}H COSY NMR (400 MHz, $CDCl_3$) spectrum of **S21.**

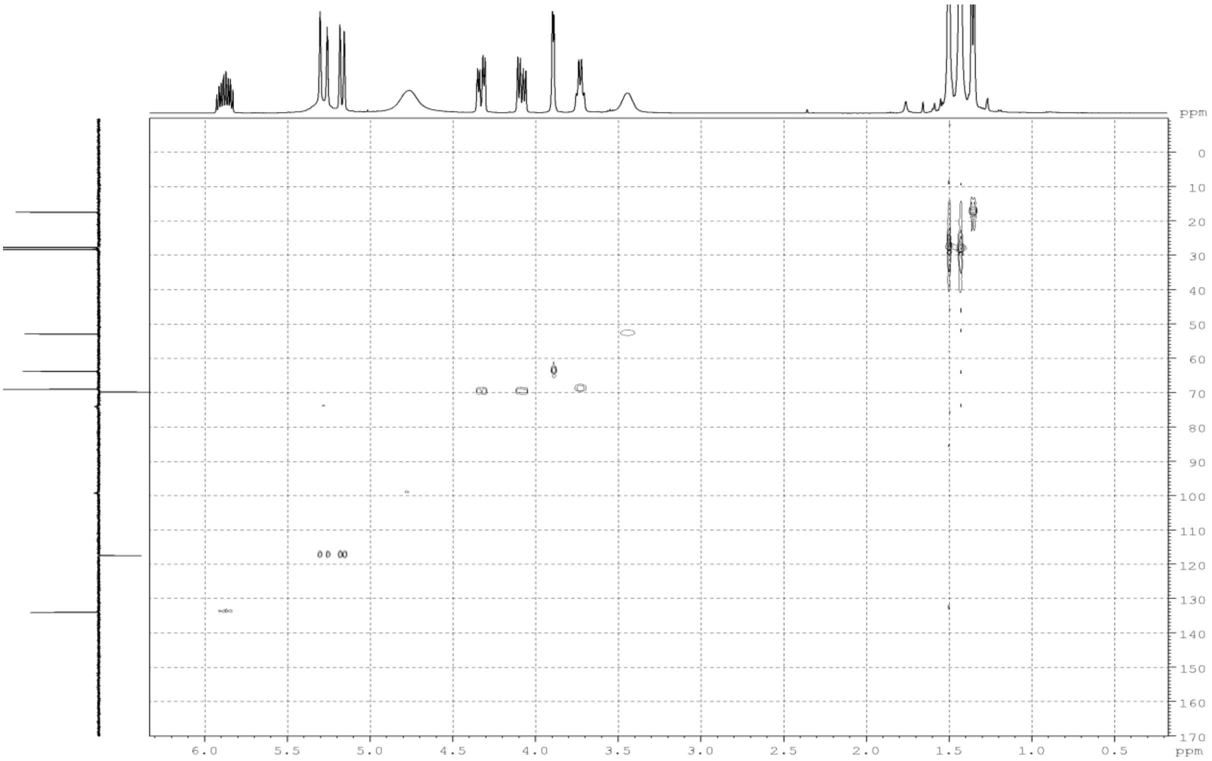

Figure. DEPT-HSQC NMR spectrum of **S21**.

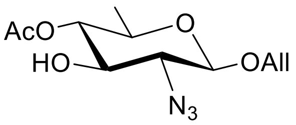

S22

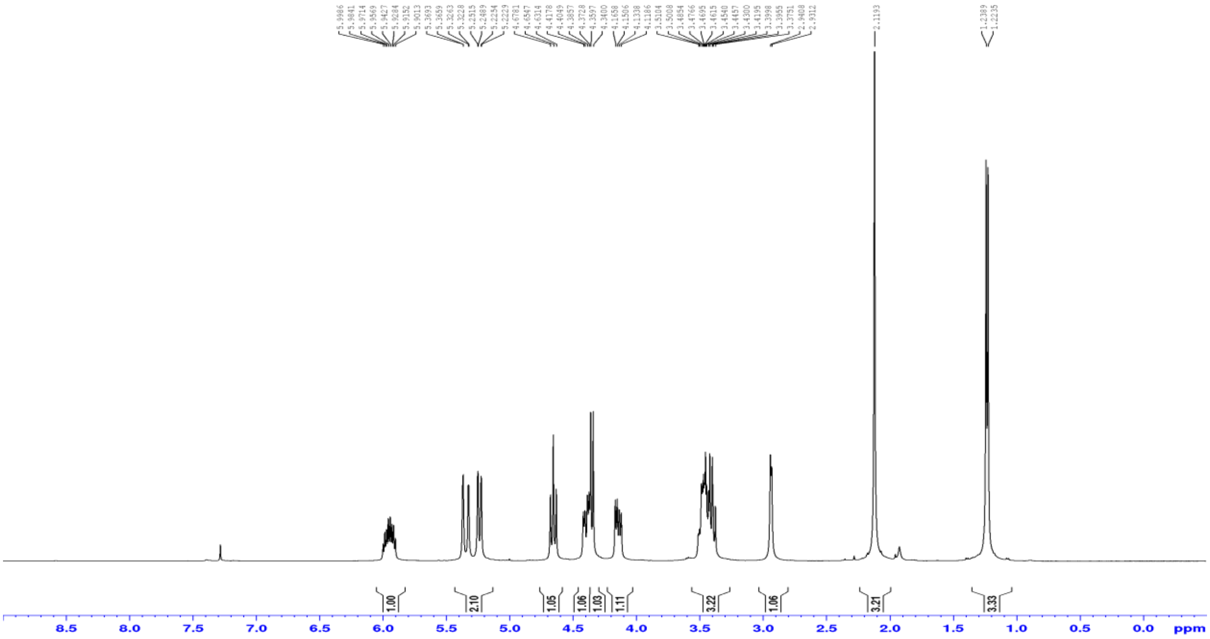

Figure. ^{1}H NMR (400 MHz, $CDCl_3$) spectrum of **S22**.

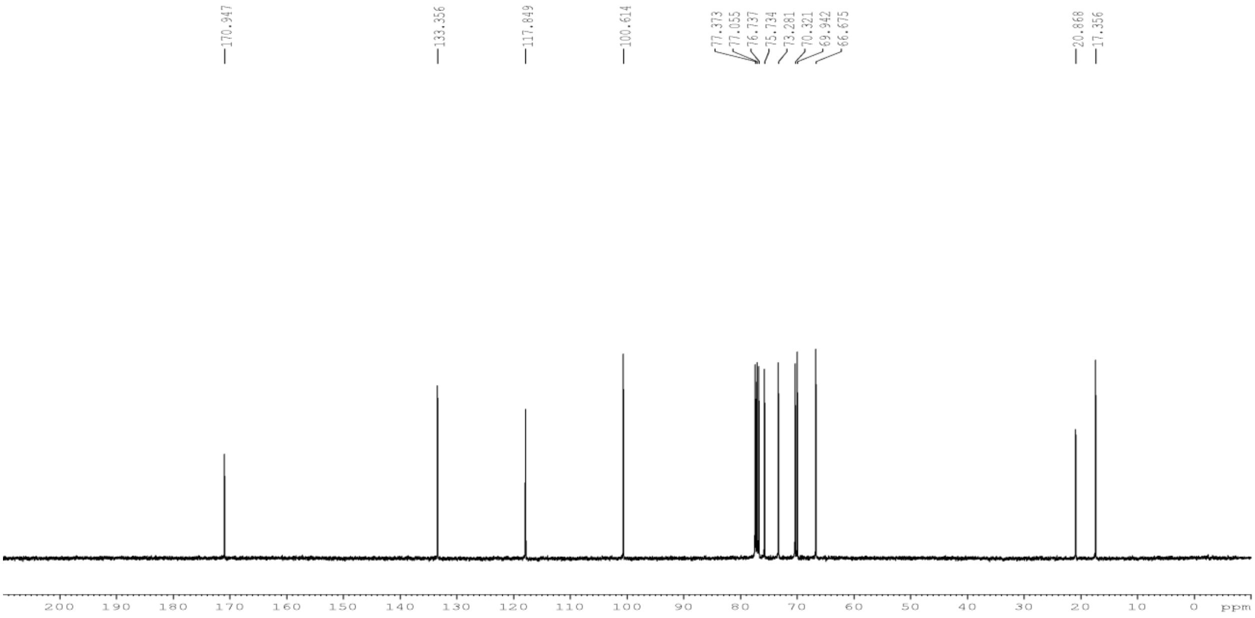

Figure. ^{13}C{^{1}H} NMR (100 MHz, $CDCl_3$) spectrum of **S22**.

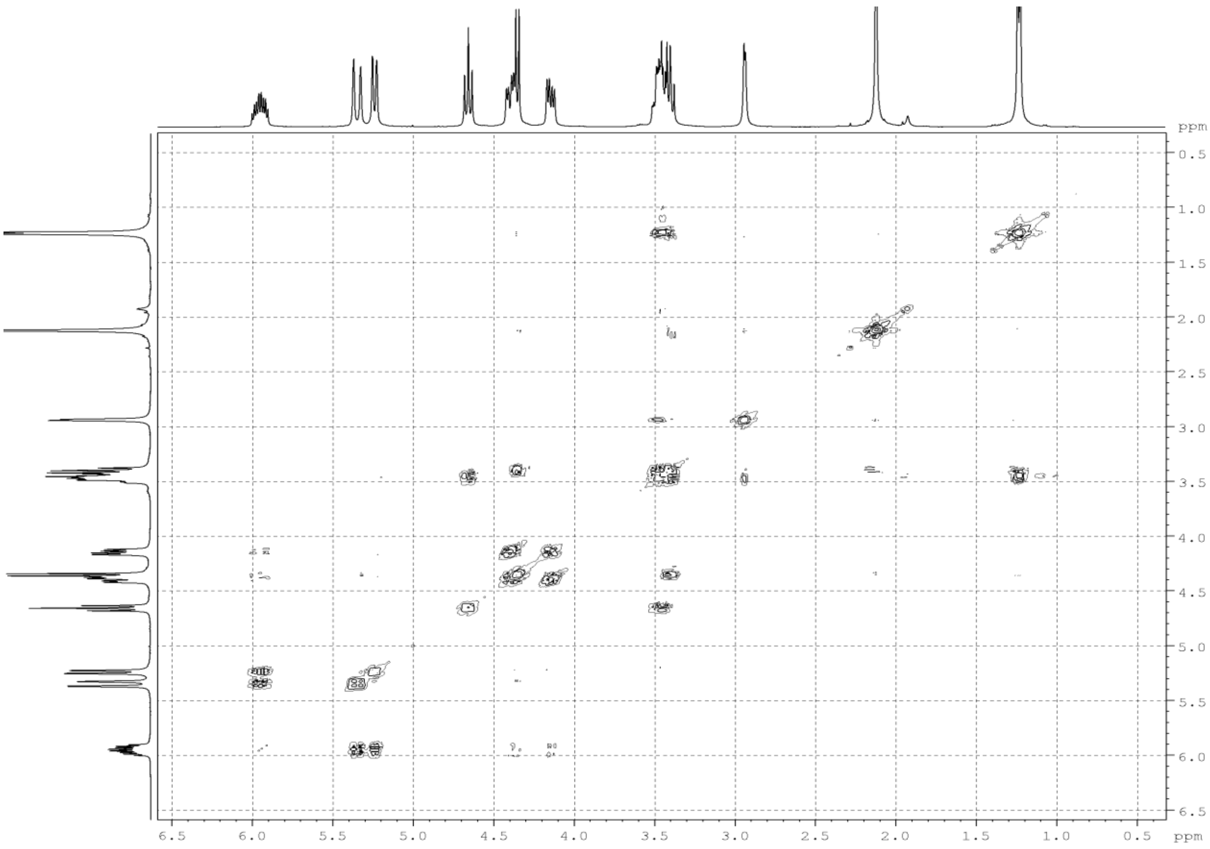

Figure. ^{1}H-^{1}H COSY NMR (400 MHz, $CDCl_3$) spectrum of **S22.**

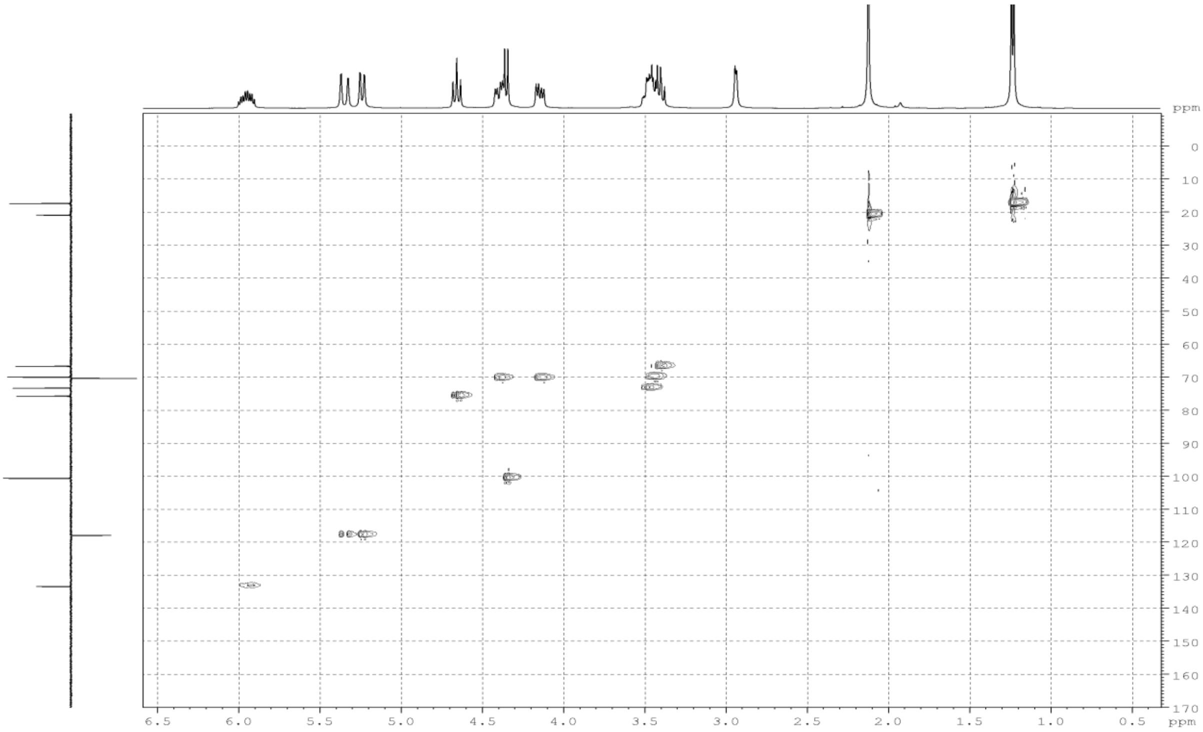

Figure. DEPT-HSQC NMR spectrum of **S22**.
